# Sex Differences in the Effects of Prenatal Bisphenol A Exposure on Genes Associated with Autism Spectrum Disorder in the Hippocampus

**DOI:** 10.1038/s41598-019-39386-w

**Published:** 2019-02-28

**Authors:** Surangrat Thongkorn, Songphon Kanlayaprasit, Depicha Jindatip, Tewin Tencomnao, Valerie W. Hu, Tewarit Sarachana

**Affiliations:** 1 The Ph.D. Program in Clinical Biochemistry and Molecular Medicine, Department of Clinical Chemistry, Faculty of Allied Health Sciences, Chulalongkorn University, Bangkok, Thailand; 2 Department of Anatomy, Faculty of Medicine, Chulalongkorn University, Bangkok, Thailand; 3 Age-related Inflammation and Degeneration Research Unit, Department of Clinical Chemistry, Faculty of Allied Health Sciences, Chulalongkorn University, Bangkok, Thailand; 4 Department of Biochemistry and Molecular Medicine, The George Washington University School of Medicine and Health Sciences, The George Washington University, Washington, DC USA

## Abstract

Autism spectrum disorder (ASD) is a neurodevelopmental disorder inexplicably biased towards males. Although prenatal exposure to bisphenol A (BPA) has recently been associated with the ASD risk, whether BPA dysregulates ASD-related genes in the developing brain remains unclear. In this study, transcriptome profiling by RNA-seq analysis of hippocampi isolated from neonatal pups prenatally exposed to BPA was conducted and revealed a list of differentially expressed genes (DEGs) associated with ASD. Among the DEGs, several ASD candidate genes, including *Auts2* and *Foxp2*, were dysregulated and showed sex differences in response to BPA exposure. The interactome and pathway analyses of DEGs using Ingenuity Pathway Analysis software revealed significant associations between the DEGs in males and neurological functions/disorders associated with ASD. Moreover, the reanalysis of transcriptome profiling data from previously published BPA studies consistently showed that BPA-responsive genes were significantly associated with ASD-related genes. The findings from this study indicate that prenatal BPA exposure alters the expression of ASD-linked genes in the hippocampus and suggest that maternal BPA exposure may increase ASD susceptibility by dysregulating genes associated with neurological functions known to be negatively impacted in ASD, which deserves further investigations.

## Introduction

Autism spectrum disorder (ASD) is an early-onset neurodevelopmental disorder characterized by 2 main symptoms: i) social interaction and communication impairments, and ii) restricted interests and stereotyped behaviors. The Centers for Disease Control and Prevention (CDC) recently reported that the prevalence of ASD is as high as 1 in 59 children in the United States^1^. ASD is inexplicably biased towards males, with a prevalence in males approximately four times higher than that in females^1^. Although there is accumulating evidence that genetic factors are associated with ASD etiology or susceptibility, the majority (80–90%) of ASD cases remain idiopathic. Moreover, recent studies have reported that epigenetic regulatory mechanisms, including DNA methylation^2–5^, histone modifications^6,7^, and RNA-associated mechanisms^8^, are associated with ASD. Epigenetic mechanisms play an important role in gene-environment interactions and susceptibility to environmental stresses, and environmental factors are also thought to be associated with ASD etiology and/or susceptibility. In fact, several recent studies have reported that exposure to certain environmental pollutants and industrial chemicals is associated with increased risk of ASD^5,9,10^. Examples of environmental chemicals that have been associated with ASD include endocrine-disrupting compounds (EDCs)^11^, lead^12^, mercury^13^, pesticides^14^, and cigarette smoke^15^.

EDCs are a group of chemicals that can be found in various products widely used in daily life. With chemical structures similar to sex hormones, particularly estrogen, EDCs are thought to disrupt hormone regulatory systems in the body by interfering in several processes, including hormone synthesis, secretion, transport, metabolism, binding process, and elimination of natural hormones that are present in the body^16^. Given that sex hormones are known to play critical roles in homeostasis, reproduction, and developmental processes, EDCs are thought to disrupt hormone-related biological functions and pose a risk for many diseases/disorders, including ASD^5,17^. EDCs that have been associated with ASD include bisphenol A (BPA), phthalates, polybrominated diphenyl ethers (PBDEs), and polychlorinated biphenyls (PCBs).

BPA ((CH_3_)_2_C(C_6_H_4_OH)_2_) is an organic compound consisting of two hydroxyphenyl groups. It is widely used in polycarbonate plastic and epoxy resin products, including linings inside beverage and food cans, plastic bottles, and dental sealants. Under high heat and alkaline conditions, BPA can be hydrolyzed and leach from products, posing a risk of exposure to consumers^18–20^. After ingestion, BPA is metabolized by UDP glucuronosyltransferases in the liver to BPA-glucuronide which is then excreted in urine^21^. The U.S. Food and Drug Administration (FDA) and European Food Safety Authority (EFSA) have determined the No-Observed-Adverse-Effect Level (NOAEL) for BPA to be 5,000 µg/kg body weight/day and established the Tolerable Daily Intake (TDI) of 50 µg/kg body weight/day which is derived by applying a 100-fold uncertainty factor to the NOAEL^22^.

BPA can circulate throughout the body and pass through the placenta and blood-brain barrier^23,24^. Although the safety of exposure to BPA remains unclear, accumulating evidence indicates that BPA alters synaptic plasticity^25^, neonatal brain development^26^, neurogenesis^27,28^, learning^29^, memory^30^, anxiety^31,32^, and social interaction^33^, all of which have been implicated in ASD^34–36^. Moreover, recent studies have reported that BPA exposure is associated with the risk of ASD^37–40^. Stein *et al*. (2015) determined the concentrations of free and total BPA in urine obtained from 46 children with ASD and 52 typically developing children using liquid chromatography-mass spectrometry (LC-MS/MS) analysis. They found that approximately 20% of the ASD children had BPA levels beyond the 90th percentile (>50 ng/ml) of the frequency distribution for the total sample of 98 children^39^. Moreover, Kardas *et al*. (2016) measured the levels of BPA, mono-(2-ethylhexyl)-phthalate (MEHP), and di-(2-ethylhexyl)-phthalate (DEHP) in the sera of 48 children with ASD and 41 typically developing children using high-performance liquid chromatography (HPLC). Interestingly, the levels of serum BPA, MEHP, and DEHP were significantly increased in children with ASD compared with controls^37^. Similar to Kardas *et al*. (2016), Kondolot *et al*. (2016) measured the plasma concentrations of BPA and phthalates in 51 ASD children and 50 age-/sex-matched typically developing children using HPLC analysis^38^. They found that the average plasma BPA level of children with a specific disorder in the autism spectrum called PDD-NOS (pervasive developmental disorder not otherwise specified) was significantly higher than that of the control, and the average level of BPA detected in the plasma was as high as 6.91 ng/ml^38^. Furthermore, it was reported that *in vitro* BPA exposure induced oxidative stress and mitochondrial dysfunctions in lymphoblastoid cell lines derived from individuals with ASD and unaffected siblings, suggesting that BPA may act as an environmental risk factor for ASD^40^. Nevertheless, whether BPA exposure can cause changes at the molecular level reminiscent of those observed in individuals with ASD has not been investigated.

In this study, we therefore sought to determine the effects of prenatal BPA exposure on transcriptome profiles in the context of ASD. First, we conducted a transcriptome profiling analysis of hippocampal tissues isolated from neonatal rats prenatally exposed to BPA or control vehicle to investigate the effects of BPA exposure on gene expression profiles in the hippocampus. The lists of differentially expressed genes (DEGs) in males and females were compared with autism candidate genes to determine the associations between the DEGs and ASD and the sex differences in the effects of BPA on the expression of ASD candidate genes. Biological pathways and interactome analysis were predicted using Ingenuity Pathway Analysis (IPA) software. DEGs that were associated with ASD were selected for further confirmation by qRT-PCR analysis. Moreover, we conducted a data-mining analysis of transcriptome profiles published in the NCBI GEO DataSets database to identify genes that were differentially expressed in response to BPA exposure. The list of significant genes was then overlapped with lists of ASD candidate genes obtained from two different ASD databases to predict whether BPA-responsive genes were also significantly associated with ASD candidate genes.

## Results

### Prenatal BPA exposure alters hippocampal transcriptome profiles in a sex-dependent manner

To examine whether prenatal BPA exposure could lead to dysregulation of ASD candidate genes in the developing brain *in vivo*, we conducted an RNA-seq analysis of hippocampal tissues isolated from male and female neonatal rats exposed to 5,000 µg/kg·maternal BW of BPA *in utero* or vehicle control. Notably, the dose of BPA used to treat rats in this study is equal to the No-Observed-Adverse-Effect Level (NOAEL) in humans as determined by the FDA and ESFA. We found that when all male and female rat pups under the same treatment condition were combined into one group, as many as 5,624 transcripts corresponding to 4,525 genes were significantly differentially expressed in the hippocampi of BPA-treated rats compared with the controls. In addition, to determine whether prenatal BPA exposure alters hippocampal transcriptome profiles in a sex-dependent manner, DEGs in each sex were identified. We found that 2,496 transcripts (corresponding to 2,078 genes) and 4,021 transcripts (corresponding to 3,522 genes) were significantly differentially expressed in the hippocampi of BPA-treated male and female pups, respectively, compared to controls (P-value < 0.05 and FDR < 0.05). This finding indicates that the brain transcriptome profiles of males and females were unequally disturbed by prenatal BPA exposure. The lists of DEGs are shown in Supplementary Table S1.

### BPA-responsive DEGs in the hippocampus exhibit sex differences in ASD-associated genes

To determine whether DEGs in response to prenatal BPA exposure are associated with ASD, the lists of BPA-responsive genes were overlapped with the lists of ASD candidate genes from two ASD databases, including the SFARI and AutismKB databases. When all male and female pups were combined, a total of 298 and 700 genes among the DEGs were found to be ASD candidate genes in the SFARI and AutismKB databases, respectively. We next performed hypergeometric distribution analyses to assess the over-representation of ASD candidate genes among DEGs responsive to BPA. Hypergeometric distribution analysis of the list of DEGs in the combined male and female pups with respect to autism candidate genes showed no significant association. However, when each sex was analyzed separately, DEGs from male and female hippocampal tissues exhibited significant enrichment in ASD-related genes from the SFARI database (Table 1, Supplementary Table S2). Notably, DEGs in male hippocampal tissues tended to exhibit stronger associations with ASD genes than those in female tissues. This male bias was also observed when the list of DEGs was analyzed for enrichment of syndromic ASD genes in the AutismKB database. These results indicated that DEGs due to BPA exposure showed sex differences in their associations with ASD genes. In addition, to determine whether enrichment of ASD-related genes exists on the X chromosome in these DEG lists, we conducted hypergeometric distribution analyses between ASD-related genes on the X chromosome and each of these lists. Interestingly, we found significant enrichment of ASD-related genes on the X chromosome in the list of ASD-related DEGs in both sexes (9 from 298 genes; P-value = 6.45E-06), DEGs in males only (11 from 183 genes; P-value = 8.46E-10), and DEGs in females only (15 from 266 genes; P-value = 1.20E-12), suggesting that the X chromosome may be involved in the underlying mechanism of BPA-associated risk for ASD.

**Table 1:** Association analysis between differentially expressed genes in hippocampi of offspring prenatally exposed to BPA and ASD-related genes.

Overlap with (number of genes)	Gene list category	P-value from hypergeometric analysis (number of overlapping genes from both sexes)	P-value from hypergeometric analysis (number of overlapping genes from males)	P-value from hypergeometric analysis (number of overlapping genes from females)
**SFARI database** (1,007 genes)	All	0.50 (298)	**1**.**32E-05** (183)	**3**.**83E-03** (266)
	Syndromic	0.57 (39)	**1**.**82E-11** (51)	6.34E-02 (41)
	Score 1	0.12 (10)	**5**.**40E-06** (13)	**1**.**57E-02** (11)
	Score 2	0.58 (16)	**5**.**73E-05** (20)	**1**.**84E-02** (21)
	Score 3	**1**.**07E-02** (63)	**1**.**22E-02** (35)	**4**.**49E-04** (60)
	Score 4	0.48 (113)	0.13 (63)	8.57E-02 (105)
	Score 5	0.75 (40)	0.95 (15)	0.95 (28)
**AutismKB database** (3,055 genes)	All	0.99 (700)	0.99 (322)	0.99 (562)
	Syndromic	0.80 (24)	**1**.**23E-02** (22)	8.38E-02 (29)
	Non-syndromic	0.99 (694)	0.99 (317)	0.99 (558)

We overlapped the lists of significantly differentially expressed BPA-responsive genes in the neonatal hippocampus and ASD-related genes (SFARI and AutismKB databases). The lists of significantly differentially expressed genes in both sexes were analyzed using MeV software with a standard Bonferroni test (P-value < 0.05), and the lists of sex-specific significantly differentially expressed genes from the RNA-seq process were analyzed using Poisson distribution (FDR < 0.05, P-value < 0.05). P-values of association were calculated using hypergeometric distribution analysis and are shown in the table. SFARI scores represent the level of confidence. Score 1 = High confidence; Score 2 = Strong candidates; Score 3 = Suggestive evidence; Score 4 = Minimal evidence; Score 5 = Hypothesized; Syndromic: all syndromic genes associated with ASD.

### BPA-responsive genes in the hippocampus are involved in biological functions, canonical pathways, and networks associated with ASD

To predict biological functions, pathways, and interactome networks associated with BPA-responsive genes in the hippocampus, the lists of DEGs were analyzed using IPA software. DEGs in the hippocampus were associated with several functions impacted in ASD, including “nervous system development and function”, “inflammatory response”, and “digestive system development and function”. Interestingly, the top canonical pathways significantly associated with DEGs in the male hippocampus included “glutamate receptor signaling”, “axonal guidance signaling”, and “circadian rhythm signaling”, all of which have been associated with ASD. Similarly, “glutamate receptor signaling” and “axonal guidance signaling” were also present among the top canonical pathways significantly associated with DEGs in the female hippocampus (P-value < 0.05; Supplementary Table S3). Neurological diseases/disorders associated with DEGs included “autism or intellectual disability”, “mental retardation”, and “developmental delay”. It was interesting to note that several neurological functions, including “morphogenesis of neurons”, “neuritogenesis”, and “formation of brain”, were significantly associated with DEGs in males only (P-value < 0.05; Table 2). Additionally, the IPA comparison analysis between canonical pathways associated with DEGs in males and in females revealed several pathways that exhibited significant associations in a sex-dependent manner. Such canonical pathways included “DNA methylation and transcriptional repression signaling”, “IGF-1 signaling”, “synaptic long-term potentiation”, and “androgen signaling”, all of which have been associated with ASD (Supplementary Table S4).

**Table 2:** Comparison of neurological diseases/disorders and nervous system development functions between both sexes and males and females separately. NS = Not significant.

Disease or Function Annotation	P-values (number of genes)		
	Both Sexes	Males	Females
Autism or intellectual disability	5.19E-04 (144)	4.71E-16 (97)	9.30E-10 (120)
Mental retardation	9.66E-04 (133)	1.49E-14 (89)	1.11E-09 (113)
Familial syndromic intellectual disability	6.51E-03 (84)	1.40E-10 (58)	3.09E-07 (73)
Disorder of stature	5.78E-03 (55)	5.05E-03 (25)	8.70E-06 (47)
Autism	NS	5.77E-03 (15)	NS
Global developmental delay	NS	4.40E-04 (4)	NS
Developmental delay	9.55E-04 (20)	NS	NS
Development of central nervous system	NS	4.06E-03 (21)	NS
Development of neurons	NS	5.14E-03 (21)	NS
Morphogenesis of neurons	NS	4.18E-04 (19)	NS
Neuritogenesis	NS	9.01E-04 (18)	NS
Formation of brain	NS	1.60E-03 (14)	NS
Migration of neurons	NS	NS	1.83E-05 (11)

**Figure 1:**
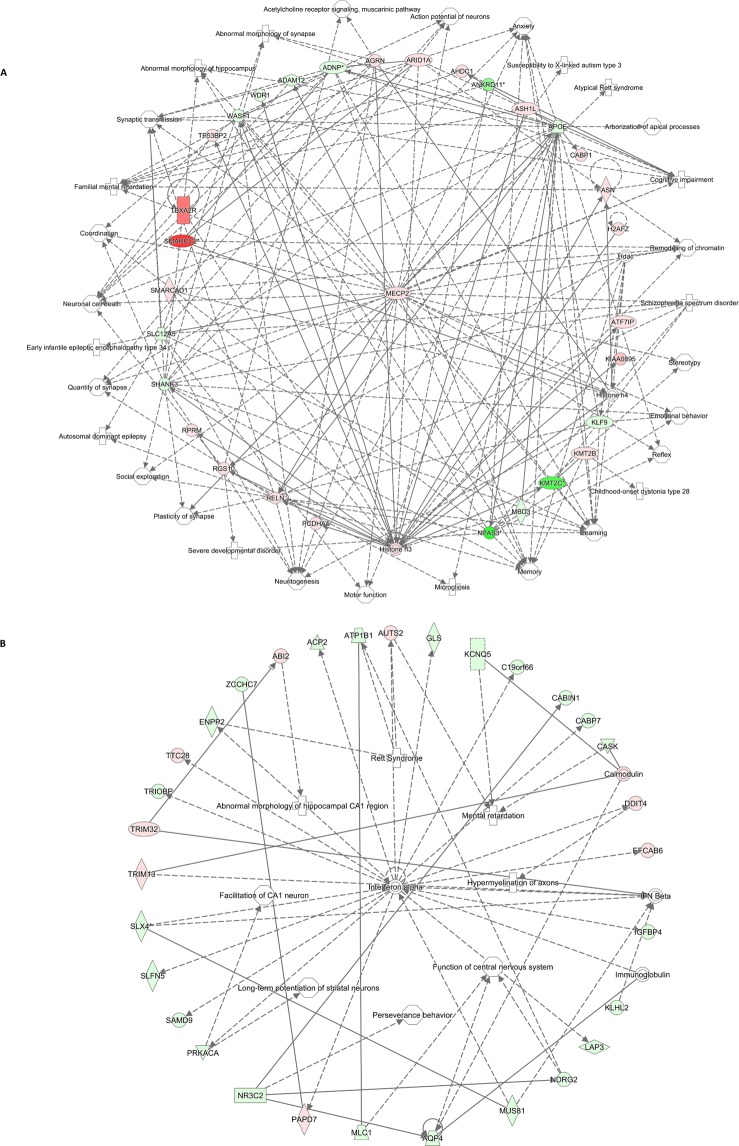
The regulatory network of DEGs in hippocampal tissues is related to neurological diseases/disorders and functions that are impacted in ASD. The gene regulatory network was predicted by IPA software using the list of DEGs from RNA-seq (colored; red = up-regulation; green = down-regulation), and the IPA showed that these genes are associated with functions that are impacted in individuals with ASD. (**A**) male (**B**) female.

Interactome networks, which are collections of genes that interact with each other or with specific biological functions, were created using the lists of significant DEGs in males and females (Fig. 1). A representative interactome network of DEGs in the male hippocampus revealed gene interactions among DEGs and associations with disorders/diseases, neurological functions, and behaviors, including mental retardation, neuritogenesis, social exploration, learning, and motor functions (Fig. 1). Similarly, the interactome network of DEGs in the female hippocampus showed associations with Rett syndrome, perseverance behavior, and mental retardation (Fig. 1). Interestingly, the hub gene in the interactome generated using DEGs from the male hippocampus is MeCP2, which is the key gene responsible for Rett syndrome. These findings suggest that prenatal BPA exposure alters the expression of genes in the brain, which may in turn disrupt gene regulatory networks/pathways and neurological functions underlying the pathobiology of ASD.

In addition, to investigate whether BPA-responsive genes have divergent effects on biological pathways and networks in males and females, the separate lists of DEGs in males and females were used to predict disorders/diseases associated with ASD using IPA. Interestingly, we found that the DEGs in male but not female hippocampal tissues exclusively associated with autism (P-value = 1.18E-02, 10 genes) (Table 3**)**. However, DEGs in both males and females were significantly associated with pervasive developmental disorder (P-value = 2.20E-02, 17 genes, and P-value = 1.44E-04, 41 genes, respectively), which is currently considered a component of ASD.

**Table 3:** Comparison of neurological diseases/disorders of DEGs uniquely found in males or females.

Diseases or Functions Annotation	P-values (number of genes)	Diseases or Functions Annotation	P-values (number of genes)
	Males		Females
Huntington’s Disease	6.67E-03 (40)	Disorder of basal ganglia	2.72E-04 (122)
Pervasive developmental disorder	2.20E-02 (17)	Dementia	1.90E-04 (118)
Autism	1.18E-02 (10)	Tauopathy	3.82E-05 (117)
Alcohol withdrawal syndrome	3.15E-02 (4)	Alzheimer disease	1.66E-04 (110)
Susceptibility to Alzheimer disease	7.03E-03 (3)	Pervasive developmental disorder	1.44E-04 (41)

The lists of genes that were dysregulated only in males or females were used to predict the neurological diseases/disorders associated with ASD using IPA. Significance was determined by the Fisher’ exact test, with a P-value = 0.05 as the cutoff.

To determine whether the gene expression profiles in the hippocampi of rats prenatally exposed to BPA reflect those in the brains of ASD individuals, we obtained the lists of genes that are differentially expressed in post-mortem brain tissues of ASD individuals from two previously published ASD brain transcriptome profiling studies^41,42^, and overlapped them with our list of BPA-responsive genes. Interestingly, we found that as many as 206, 159, and 80 genes differentially expressed due to BPA exposure in both sexes, in females, and in males, respectively, were also dysregulated in ASD post-mortem brain tissues identified by Voineague, I., *et al*.^41^. In addition, as many as 1,045, 690, and 393 genes differentially expressed by BPA exposure in both sexes, in females, and in males, respectively, were also dysregulated in ASD post-mortem brain tissues identified by Parikshak, N. N., *et al*.^42^. The lists of DEGs identified by both ASD brain transcriptome studies and genes overlapping with BPA-responsive genes are shown in Supplementary Table S5. This finding suggests that prenatal BPA exposure may result in dysregulation of at least some genes reminiscent of those altered in the brains of ASD individuals.

### Quantitative RT-PCR analysis of BPA-responsive genes

**Figure 2:**
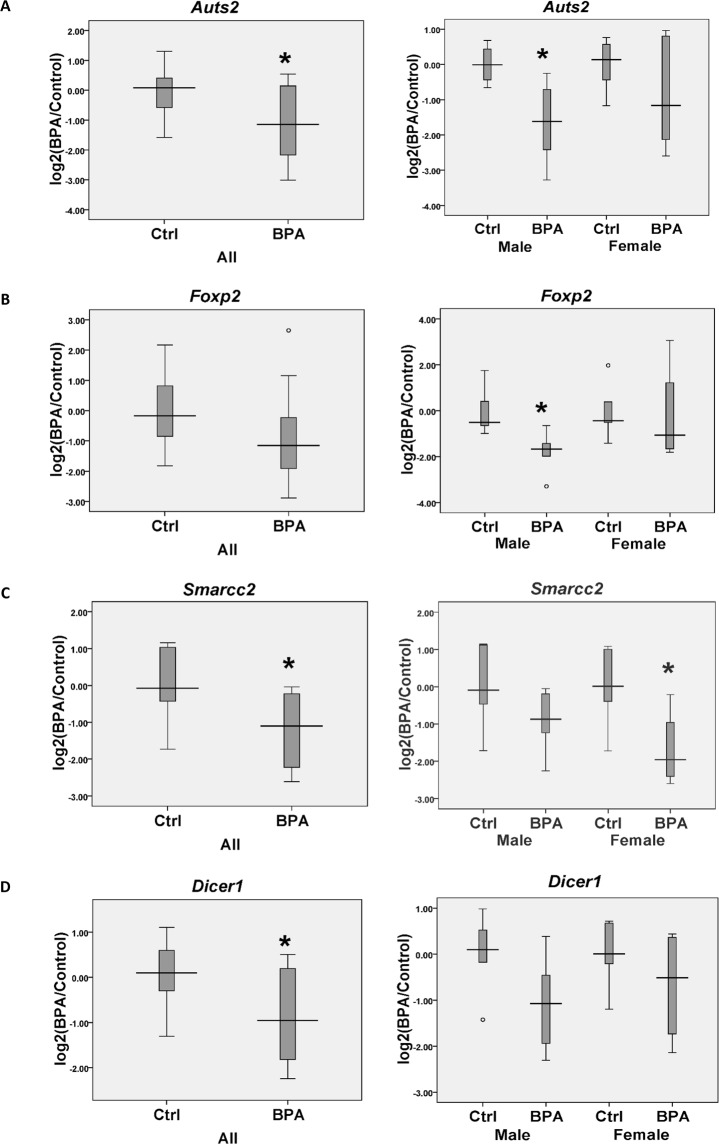
Box plot of ASD-related gene expression in hippocampal tissues. The expression levels of *Auts2* (**A**), *Foxp2* (**B**), *Smarcc2* (**C**), and *Dicer1* (**D**) were determined in both sexes and separately in males and females. The qRT-PCR analyses revealed that *Auts2* and *Foxp2* were significantly down-regulated in the hippocampi of both sexes and males that were prenatally exposed to BPA. In contrast, *Smarcc2* was significantly reduced in both sexes and in females, and *Dicer1* was significantly reduced in both sexes. * P-value < 0.05.

To further examine whether prenatal BPA exposure causes the dysregulation of genes in the hippocampus, four DEGs (i.e., *Auts2*, *Foxp2*, *Smarcc2*, and *Dicer1*) identified by RNA-seq analysis were selected for further confirmation by qRT-PCR analysis in another set of hippocampal tissue samples (Fig. 2). *Auts2* (Autism Susceptibility Gene 2), *Foxp2* (Forkhead Box P2), and *Smarcc2* (SWI/SNF Related, Matrix Associated, Actin Dependent Regulator of Chromatin subfamily C member 2) have been identified as ASD candidate genes, whereas *Dicer1* (Dicer 1, Ribonuclease III) is involved in a post-transcriptional gene silencing mechanism that has been associated with ASD. We found that when both males and females were combined, the expression levels of the *Auts2*, *Smarcc2*, and *Dicer1* genes were significantly reduced in the hippocampi of rats prenatally exposed to BPA (Fig. 2). *Foxp2* expression tended to decrease in the BPA group, although the difference was not statistically significant. Interestingly, sex-specific dysregulation of genes was observed when qRT-PCR data from each sex were analyzed separately. The expression levels of *Auts2* and *Foxp2* were significantly decreased in males but not in females (Fig. 2), whereas *Smarcc2* expression was significantly decreased in females but not in males (Fig. 2). These results indicate that prenatal BPA exposure causes the dysregulation of genes associated with ASD in the hippocampus in a sex-dependent manner.

### DEGs in response to BPA exposure based on the integration of data from multiple transcriptomic studies revealed an association with ASD candidate genes

To determine whether BPA-responsive genes identified by other independent investigators were also associated with ASD, transcriptome profiling data from cell lines, primary cells, or tissues from animal models treated with BPA were obtained from six independent transcriptomic studies previously deposited in the NCBI GEO DataSets database (https://www.ncbi.nlm.nih.gov/gds/). The details of each study, including the title, sample size, and sample type, are shown in Supplementary Table S6. Significantly differentially expressed genes in the BPA treatment group compared with the corresponding control group from each transcriptomic study were then identified using a common statistical program for large-scale expression analyses. The lists of BPA-responsive genes from the transcriptomic studies are shown in Supplementary Table S7. We next overlapped the list of DEGs from each study with ASD candidate genes previously deposited in two different ASD databases: SFARI (https://gene.sfari.org/) and AutismKB (http://autismkb.cbi.pku.edu.cn/). Furthermore, hypergeometric distribution analyses were performed to determine whether ASD candidate genes were associated with the BPA-responsive genes from each study. Interestingly, several to hundreds of ASD candidate genes were found to be differentially expressed in response to BPA, and the hypergeometric distribution analyses revealed that the ASD candidate genes obtained from each ASD database were significantly enriched (P-value < 0.05) in the lists of BPA-responsive genes identified from four of six transcriptomic studies (Table 4).

**Table 4:** Hypergeometric distribution analyses between significantly differentially expressed genes from BPA studies and autism candidate genes.

Overlap with	GSE44387	GSE63852	GSE58642	GSE50527	GSE58516	GSE86923
**SFARI database** (1,007 genes)	44	26	2	75	1	88
*P-value from hypergeometric distribution analysis	**5**.**60E-10**	**1**.**15E-07**	0.26	**1**.**42E-15**	0.73	**4**.**35E-11**
**AutismKB database** (3,055 genes)	142	76	3	168	7	289
*P-value from hypergeometric distribution analysis	**4**.**60E-24**	**1**.**23E-17**	0.59	**1**.**36E-19**	0.09	**5**.**23E-41**

Hypergeometric distribution analyses were used to analyze associations between differentially expressed genes from six previously published BPA transcriptome studies and autism candidate genes. Statistically significant associations were determined by hypergeometric distribution analysis (P-value < 0.05).

To determine whether the BPA-responsive genes identified by our study were also dysregulated in the independent studies, the lists of BPA-responsive genes in the hippocampi of rats prenatally exposed to BPA were overlapped with the BPA-responsive genes from the previously published transcriptome studies. The numbers of overlapping genes are shown in Table 5. When the DEGs from the published studies were combined, as many as 914 DEGs identified by our study were also found to be dysregulated in at least one of the independent studies (Supplementary Table S8). IPA revealed that this set of genes was significantly associated with several canonical pathways, including “Aldosterone Signaling in Epithelial Cells” (P-value = 2.14E-04), “PTEN Signaling” (P-value = 1.62E-03), “PPARα/RXRα Activation” (P-value = 5.75E-03), “Dendritic Cell Maturation” (P-value = 1.02E-02), and “Circadian Rhythm Signaling” (P-value = 1.78E-02) (Table 6). Taken together, the results of these bioinformatic analyses suggest that BPA exposure may cause dysregulation of genes associated with ASD-related biological functions in the brain as well as other tissues.

**Table 5:** Numbers of overlapping genes between our list of DEGs in the hippocampus and the lists of BPA-responsive genes identified by other transcriptome profiling studies.

Overlap with	GSE44387 (840 genes)	GSE63852 (366 genes)	GSE58642 (35 genes)	GSE50527 (1,250 genes)	GSE58516 (43 genes)	GSE86923 (1,869 genes)
Both sexes (4,525 genes)	228	84	5	251	6	429
Males (2,078 genes)	95	31	3	95	1	188
Females (3,522 genes)	152	67	1	156	3	325

**Table 6:** Significant canonical pathways associated with our DEGs that were also identified as BPA-responsive genes in other independent studies.

Canonical Pathways	P-values	Genes
Aldosterone Signaling in Epithelial Cells	2.14E-04	RAF1, HSPH1, SLC12A2, TRAP1, DNAJC13, HSPD1, HSPA2, HSPA8, PIK3R3, HSP90B1, PLCB4, PIK3C3, PRKCD, PIK3CD, DNAJB6, PRKD3, HSPB6, HSPA4L, HSPB1
PTEN Signalingop	1.62E-03	RAF1, YWHAH, BAD, ITGA5, NFKB2, CCND1, SYNJ2, PIK3R3, GHR, PIK3CD, INSR, FGFRL1, FASLG, PDGFRB
PPARα/RXRα Activation	5.75E-03	RAF1, IL1RL1, MED1, NFKB2, TGS1, PRKAG1, HSP90B1, PLCB4, GHR, GPD2, LPL, SMAD4, NCOR1, INSR, NFKBIB, MED24
Dendritic Cell Maturation	1.02E-02	LEP, FCGR2A, HLA-A, TYROBP, HLA-DQA1, NFKB2, MAPK11, PIK3R3, PLCB4, PIK3C3, CD86, ATF4, PIK3CD, TLR3, IL23A, NFKBIB
Circadian Rhythm Signaling	1.78E-02	PER1, GRIN2A, ATF4, VIP, PER2

The list of DEGs in the hippocampus was overlapped with the list of BPA-responsive genes identified by other studies. Canonical pathways associated with the overlapping genes were analyzed by IPA software. P-values were calculated using Fisher’s exact test (P < 0.05).

## Discussion

Accumulating evidence from both *in vitro* and *in vivo* studies indicates that exposure to BPA, even at low doses, disrupts the expression of multiple genes in the brain and alters the behaviors of offspring from exposed females^43,44^. Increased BPA levels have been reported in the blood and urine of ASD children compared with typically developing children^37–39^, prompting the hypothesis that BPA may be an environmental risk factor for ASD and that exposure to BPA, especially during pregnancy, may cause and/or increase the risk of ASD. However, whether prenatal BPA exposure causes the dysregulation of genes associated with ASD in the brain that could lead to the pathobiological conditions associated with ASD has never been investigated.

This is the first study to demonstrate that BPA exposure can cause sex-dependent changes in the transcriptome profiles of many genes involved in biological functions known to be negatively impacted in ASD, and that significant associations exists between BPA-responsive genes and dysregulated genes observed in individuals with ASD. Using rats as an experimental model, we demonstrated that prenatal BPA exposure in pregnant dams dysregulated the transcriptome profiles of ASD candidate genes in the brains of the offspring. Specifically, RNA-seq analysis of hippocampal tissues isolated from prenatally exposed neonatal rats showed sex differences in the response to BPA exposure, with 2,078 and 3,522 DEGs in the hippocampi of males and females, respectively, indicating that prenatal BPA exposure affects brain transcriptome profiles in a sex-dependent manner. Sex differences in the effects of prenatal BPA exposure on brain transcriptome profiles have also been reported in recent studies^43,45^. Arambula *et al*. (2016) conducted a transcriptome profiling analysis of hypothalami and hippocampi isolated from neonatal rats prenatally exposed to BPA^43^ and found that BPA induced sex-specific effects on hypothalamic ERα and ERβ (*Esr1* and *Esr2*) expression and hippocampal and hypothalamic oxytocin (*Oxt*) expression. Moreover, prenatal BPA exposure was reported to disrupt the transcriptome of the neonate amygdala in a sex-specific manner^45^.

Interestingly, when overlapped with the lists of ASD candidate genes, the list of DEGs in males identified in this study exhibited stronger associations with ASD genes than the DEGs in females. Moreover, we found significant enrichment of ASD genes on the X chromosome in the lists of ASD-related DEGs in both males and females, suggesting that BPA exerts its effect on the brain partly through X-linked genes, which provides a plausible explanation for the sex difference in BPA effects on the brain transcriptome. Notably, the X chromosome theory of ASD^46–48^ posits that the male bias of ASD partly involves genes on the X chromosome, the dysregulation of which increases susceptibility to ASD. This result suggests that prenatal BPA exposure may elevate the risk of ASD in males and may help explain the higher male prevalence of ASD, which deserves further study. Additionally, IPA showed that DEGs in the hippocampus were significantly associated with ASD and mental retardation. Canonical pathways associated with DEGs in both males and females included glutamate receptor signaling, axonal guidance signaling, and circadian rhythm signaling, all of which have been associated with ASD^49–51^. Interestingly, several neuro/biological functions and disorders, including “autism”, “global developmental delay”, “formation of brain”, “neuritogenesis”, and “inflammatory response”, were associated with DEGs in the male hippocampus only. The canonical pathway analysis also revealed significant associations of DEGs with “DNA methylation and transcriptional repression signaling” and “4-aminobutyrate degradation” in male only, both of which have been associated with ASD^3,52–54^. We then overlapped the DEGs in males together with those in females, and the lists of genes that were found to be dysregulated in only males or females were separately analyzed to demonstrate diseases/disorders specific to male and female DEGs. The results revealed that genes that were dysregulated in males were significantly associated with “Autism” (P-value = 1.18E-02) while the dysregulated genes in females were associated with “Pervasive developmental disorder” (P- value = 1.44E-04). Pervasive development disorder is a group of disorders characterized by developmental delays of socialization and communication skills, consisting of autism, Asperger syndrome, Rett syndrome, childhood disintegrative disorder, and pervasive developmental disorder-not other wised specified (PDD-NOS). In the DSM-5, all of these neurodevelopmental conditions, except for Rett syndrome, were grouped into the new classification for autism spectrum disorder (ASD) which has an overall prevalence of approximately 1 in 59 children and is 4 times higher in males than females^1^. This result suggests that exposure to BPA during pregnancy can cause divergent effects on the expression of genes associated with ASD in both sexes, but may be more directly associated with classic autism (typically considered the most severe subtype) in males. Interactome analysis showed that *Mecp2*, a gene located on the X chromosome encoding the methyl-CpG binding protein 2, served as the hub gene in a biological network of DEGs in the hippocampus. This protein mediates transcriptional repression through interaction with histone deacetylase^55,56^ and plays a role in the maintenance of synapses and normal brain function^57,58^. Loss-of-function mutations of *MeCP2* in humans are known to cause Rett syndrome, a childhood neurodevelopmental disorder with some ASD-related symptoms that affects females almost exclusively. An increased *MeCP2* gene copy number was reported in males with neurodevelopmental delay who exhibited autistic-like features, absent speech, stereotypic movements, and infantile hypotonia^59^. Moreover, increased binding of MeCP2 to the promoters of *GAD1* and *RELN* which are candidate genes for ASD was also found in the ASD cerebellum^60^. This evidence suggests that the up-regulation of *Mecp2* due to prenatal exposure to BPA may lead to ASD-like symptoms, which should be further studied.

We then conducted quantitative RT-PCR analyses to further investigate the expression levels of four DEGs (*Auts2*, *Foxp2*, *Smarcc2*, and *Dicer1*) in the hippocampi of neonatal rats prenatally exposed to BPA compared with vehicle control. *Auts2*, *Smarcc2*, and *Dicer1* were significantly reduced in the hippocampi of the BPA group compared with the control, whereas *Foxp2* tended to decrease but did not show a statistically significant difference. Although the expression levels of these four genes seemed to be reduced in rats of both sexes exposed to BPA, there were some sex differences in the effects of BPA exposure on the expression levels of these genes. *Auts2* and *Foxp2* were significantly decreased in the hippocampi of male rats exposed to BPA compared with sex-matched controls, but these differences were not observed in females. *Smarcc2*, in contrast, was significantly decreased in females prenatally exposed to BPA, but not in males. These findings suggest that prenatal BPA exposure may pose an increased risk of ASD in males and females by disrupting the expression profiles of ASD-related genes, providing a plausible explanation for how an environmental factor can contribute to ASD susceptibility. The molecular mechanisms underlying how BPA affects differential gene expression between males and females should be studied further, but evidence indicates that exposure to BPA can alter genes related to global DNA methylation and histone modification processes^44,61^.

*Auts2* (Autism Susceptibility Candidate 2) is an ASD candidate gene that has been associated with ASD and other neurodevelopmental disorders that are comorbid with ASD, including intellectual disability^62^ and developmental delay^62^. *Auts2* is abundantly expressed in the developing brain and is mostly expressed in the hippocampus, prefrontal cortex, and cerebellum^63^, which are brain regions known to be impacted in individuals with ASD^64^. Recent studies have revealed that *Auts2* is important for neuronal development. Knockout of both coding and noncoding sequences of the *Auts2* gene in zebrafish caused microcephaly and a decreased number of neuronal cells^65^, both of which are consistently found in ASD patients^66^.

*Foxp2* (Forkhead Box P2) encodes a member of the forkhead/winged-helix (FOX) family of transcription factors that is widely reported as a candidate gene associated with language development^67^. Foxp2 is expressed in the fetal and adult brain and is required for the development of speech and language regions of the brain during embryogenesis. Mutation of this gene has been reported in speech-language disorder 1 (SPCH1), also known as autosomal dominant speech and language disorder with orofacial dyspraxia. A single-nucleotide polymorphism (SNP) in the *FOXP2* gene has been associated with social deficits in ASD patients^68,69^. Moreover, the disruption of *Foxp2* in mice caused altered ultrasonic vocalization^70^.

*Smarcc2* (SWI/SNF Related, Matrix Associated, Actin Dependent Regulator of Chromatin subfamily C member 2) encodes a member of the SWI/SNF family of proteins. The functions of this gene include transcriptional activation and repression by chromatin remodeling process^71^. *Smarcc2* is highly expressed in the brain and is required for the differentiation of stem/progenitor cells into mature neural cells during neural development. Recent studies reported that mutation of *Smarcc2* resulted in alteration of chromatin remodeling complexes in ASD^6^. A *de novo* splice-site variant in this gene was also observed in ASD cases^72^.

The *Dicer1* (Dicer 1, Ribonuclease III) gene encodes a protein involved in the repression of gene expression. The protein acts as a ribonuclease that is required for RNA interference and small temporal RNA (stRNA) in the small RNA component production pathway. There is evidence that post-transcriptional mechanisms are associated with ASD. Recent studies revealed dysregulated miRNAs in the ASD brain^73^ and in lymphoblastoid cell lines derived from individuals with ASD^8,74^.

To further understand the systemic effects of BPA, we identified BPA-responsive genes using the transcriptome profiles of cells/tissues isolated from animals exposed to BPA because of the limitation of brain transcriptome data in the GEO DataSets database. In addition, we attempted to use several statistical tests, such as t-test with standard Bonferroni correction, to identify the DEGs, but we were unable to identify any DEGs from the studies under these stringent conditions for multiple testing correction. We then used student’s t-test to re-analyze the significant DEGs from other studies with the goal of identifying some genes that are dysregulated due to BPA exposure in other cells/tissues. Hypergeometric distribution analyses were then performed using BPA-responsive genes from each transcriptomic study and lists of ASD candidate genes obtained from two different ASD bioinformatic databases. We found that ASD candidate genes were significantly enriched in BPA-responsive genes in four transcriptomic studies. Interestingly, one of these four transcriptomic studies investigated the effects BPA exposure on the transcriptome profiles of mouse placenta^75^. That study found that *in utero* exposure to BPA disrupted blood vessel development and morphology in the placenta. BPA exposure caused narrowing of blood vessels and disrupted the embryonic head and forelimb structures^76^. A recent study revealed that altered maternal vascular malperfusion was significantly associated with the pathobiology of ASD and increased the risk of ASD^77^.

Moreover, we overlapped the DEGs from our study with DEGs from other BPA studies in different cell types or tissues. Interestingly, we found some overlapping genes among these sets of genes, suggesting that genes that are found to be differentially expressed in the brain also show differential expression in response to BPA in other tissues. The set of overlapping genes was significantly associated with pathways impacted in ASD. There is some evidence showing that “Aldosterone Signaling”^78^, “PTEN signaling”^79^ and “Circadian Rhythm”^51^ are implicated in ASD patients. These findings suggest that BPA exposure may cause changes in the transcriptome profiles of genes involved in biological functions known to be impacted in ASD.

In addition to changes in transcriptome profiles, recent studies have shown that prenatal BPA exposure altered neurological functions, including neurogenesis in the hippocampus and hypothalamus and synaptic density in mouse models^31,80,81^. Moreover, prenatal BPA exposure induced behavioral impairments in offspring, such as in learning and memory^82^ and in social interaction^82^, along with anxiety-like behavior^31^. Whether the changes in the transcriptome profiles observed in this study could lead to altered neurological functions and behaviors should be investigated further. Moreover, in this study we used oral administration of BPA at 5,000 µg/kg of maternal BW/day which is equal to the NOAEL in humans determined by the FDA and ESFA. The TDI in humans is 50 µg/kg BW/day, and the estimated BPA exposure levels from use in food-contacting materials in infants and adults are 2.42 µg/kg BW/day and 0.185 µg/kg BW/day, respectively^22^. The effects of prenatal BPA exposure at the TDI and these estimated daily doses in humans on the brain transcriptome and functions warrant further investigation. Moreover, the molecular mechanisms through which BPA disrupts the expression of genes associated with ASD deserve further study.

## Conclusions

In this study, transcriptomic profiling analysis of hippocampi isolated from rats prenatally exposed to BPA revealed sex-dependent dysregulation of gene expression, with a greater number of differentially expressed genes in females. However, the genes that were disrupted in the male hippocampus showed more significant association with ASD than those in females. Interestingly, the expression of ASD candidate genes selected for validation by quantitative RT-PCR, including *Auts2*, *Foxp2*, and *Smarcc2*, was also sex-dependent in response to prenatal BPA exposure. Finally, re-analyses of transcriptomic data obtained from multiple published studies on the effects of BPA in various cellular, tissue, and animal models support our current findings that BPA-responsive genes are significantly associated with ASD candidate genes as well as ASD-related neurological functions and disorders. Taken together, this study shows that prenatal BPA exposure causes changes in the hippocampal expression of genes associated with ASD in a sex-specific fashion, supporting the hypothesis that BPA is an environmental risk factor for ASD, and thus providing a plausible explanation for how BPA exposure may contribute to the sex bias of ASD.

## Methods

### Animal husbandry and treatment

Eight-week-old female and male Wistar rats were purchased from the National Laboratory Animal Center (NLAC), Thailand. All animals were housed at the Chulalongkorn University Laboratory Animal Center (CULAC) under standard temperature (21 ± 1 °C) and humidity (30–70%) conditions in a 12-h light/dark cycle with food and RO-UV water available *ad libitum*. Female rats (gestational day 1 (GD1); n = 8) were divided into 2 groups (control group and BPA treatment group) with a total of 4 rats per group. The weight of each rat was measured daily and used to calculate the amount of BPA or vehicle control needed to treat each rat. For BPA treatment, BPA (Sigma-Aldrich, USA) was dissolved in absolute ethanol (Merck Millipore, USA) to a final concentration of 250 mg/ml to make a stock BPA solution. Then, the stock solution was further diluted with corn oil to a final concentration of 5,000 µg/kg·maternal BW of BPA to treat each rat. The vehicle control treatment was prepared by mixing absolute ethanol with corn oil in amounts equivalent to those used for preparing BPA. After mating, each rat was intragastrically administered either BPA or the vehicle control from GD1 until parturition. To prevent cross-contamination of the treatment conditions, rats in the BPA and control groups were raised separately in individual ventilated cages in a biohazard containment housing system. Separate sets of stainless steel needles and all consumable products were used for oral gavage. All reusable materials were cleaned with ethanol and rinsed with copious amounts of Milli-Q deionized water before use. All experimental procedures were approved by the Chulalongkorn University Animal Care and Use Committee (Animal Use Protocol No. 1673007 and No. 1773011), Chulalongkorn University. We confirm that all experiments were performed in accordance with the relevant guidelines and regulations.

### RNA isolation and transcriptome profiling analysis

Male and female neonatal pups were euthanized (BPA n = 6; control n = 6), and the hippocampi were isolated as previously described with slight modifications^83^. Briefly, neonatal pups were euthanized by decapitation on ice following intraperitoneal injection of 100 mg/kg·BW sodium pentobarbital. The brain was quickly removed from the head and placed in a pre-chilled tube containing ice-cold, freshly prepared 1X HBSS (Invitrogen, USA) containing 30 mM glucose (Sigma-Aldrich, USA), 2 mM HEPES (GE Healthcare Bio-Sciences, USA), and 26 mM NaHCO_3_ (Sigma-Aldrich, USA). The brain was then dissected, and the hippocampus was isolated under a Nikon SMZ18 Stereo Microscope (Nikon, Japan). Meninges were removed completely, and the hippocampal tissues were immediately placed in a tube with RNAlater (Ambion, USA) and stored at −80 °C, according to the manufacturer’s protocol, until use.

Total RNA from the hippocampus was isolated and purified using the mirVana miRNA Isolation Kit (Ambion, USA) according to the manufacturer’s protocol. The RNA integrity was assessed using an Agilent Bioanalyzer (BGI, Hong Kong). To identify DEGs in the hippocampus in response to prenatal BPA exposure, a transcriptome profiling analysis of total RNA isolated from the hippocampi of neonatal rats from six independent litters prenatally exposed to BPA or vehicle control was performed by BGI Genomics Co., Ltd using the Illumina HiSeq 4000 next-generation sequencing platform with 4 G reads (Illumina, Inc.) according to the manufacturer’s protocol. Briefly, total RNA was treated with DNase I, and oligo(dT) treatment was used for mRNA isolation. Next, the RNA was mixed with fragmentation buffer to fragment the mRNA. Then, cDNA was synthesized using the mRNA fragments as templates. Subsequently, sequencing reads were filtered and subjected to quality control. Clean reads in a FASTQ file were mapped to the rat reference genome (RefSeq ID: 1174938) using Bowtie 2^84^ and gene expression levels were then calculated using RSEM^85^. We then compared the transcriptome profiles between the BPA and the control groups with Poisson distribution. Comparisons were performed with all male and female pups with the same treatment condition combined into one group and separately for each sex. P-values were calculated using a Poisson distribution method. DEGs with a P-value < 0.05 and FDR < 0.05 were considered statistically significant.

### Quantitative RT-PCR analysis

Four DEGs in the hippocampus identified by RNA-seq transcriptomic analysis were selected for further confirmation by quantitative RT-PCR analysis. These four DEGs were selected for further validation based on differential expression between males and females as well as known association with ASD. Total RNA was used for cDNA synthesis with the AccuPower® RT PreMix (Bioneer, Korea) according to the manufacturer’s protocol. Briefly, 0.5 µg total RNA was mixed with 0.5 µg (100 pmol) oligo dT_18_ primer, and DEPC-treated water was added to 15 µl. Then, the reaction was incubated at 70 °C for 5 min and placed on ice. To perform the cDNA synthesis, the mixture (15 µl) was then transferred to an AccuPower® RT PreMix tube, and DEPC-treated water was added to 20 µl. The cDNA synthesis reaction was performed by incubating the reaction at 42 °C for 60 min, followed by 94 °C for 5 min. The cDNA reaction mixture was further diluted to a volume of 50 μl with nuclease-free water and was used as a template for subsequent qPCR analyses. Quantitative PCR analysis was conducted in triplicate using AccuPower® 2X GreenStar™ qPCR MasterMix (Bioneer, Korea) according to the manufacturer’s instructions. Briefly, 1 μl of the cDNA was mixed with 2X Greenstar Master Mix, forward primer, reverse primer, and nuclease-free water. The reaction was then incubated in a Bio-Rad CFX Connect Real-Time System (Bio-Rad, USA). The PCR amplification conditions were set as follows: an initial denaturing step at 95 °C for 15 min, followed by 40 cycles of 10 s at 95 °C for denaturing and 30 s at 55 °C for annealing/extension. Product formation was confirmed by melting curve analysis (65 to 95 °C). The expression levels were calculated by the 2^−ΔΔCt^ method using the 18 S ribosomal RNA (*Rn18s*) gene as an endogenous control. The specific primers in the qPCR analyses were designed using the UCSC Genome Browser (https://genome.ucsc.edu/), Ensembl (https://asia.ensembl.org/index.html), and Primer3 software (http://bioinfo.ut.ee/primer3-0.4.0/). Forward and reverse primers were designed for rat *Auts2*, *Foxp2*, *Smarcc2*, *and Dicer1*, and *Rn18s*. The sequences of the qPCR primers are shown in Supplementary Table S9.

### Prediction of biological functions and interactome analysis

Biological functions, disorders, canonical pathways, and interactome networks associated with DEGs were predicted using IPA software (Qiagen Inc., USA, https://www.qiagenbioinformatics.com/products/ingenuity-pathway-analysis/). The list of DEGs was overlapped with the list of genes experimentally validated to be associated with each function/disorder/canonical pathway in the Ingenuity’s Knowledge Base database. Fisher’s exact test was then performed to calculate P-values, and a P-value < 0.05 was considered statistically significant.

### Transcriptome data collection

Transcriptome profiling data of cells/tissues dissected from animals exposed to BPA or vehicle controls were obtained from the NCBI Gene Expression Omnibus database (GEO DataSets: http://www.ncbi.nlm.nih.gov/gds) in a search performed on May 13, 2017, using the keyword “bisphenol A” and the following criteria: i) the experimental models were animals, primary cells, or cell lines; and ii) each treatment group consisted of more than three samples. Transcriptome profiling data of cells exposed to chemicals other than BPA, when present in any selected study, were excluded prior to subsequent differential expression analyses.

### Identification of BPA-responsive genes and association with ASD candidate genes

To identify significant BPA-responsive genes in cells/tissues exposed to BPA, the transcriptome profile from each BPA study was analyzed separately using Multiple Experiment Viewer (MeV) (http://mev.tm4.org/)^86^. All transcriptome profiling data were filtered using a 70% cutoff, which removed transcripts for which intensity values were missing in >30% of the samples. The available transcripts were then used for identifying DEGs in the BPA group with two-tailed t-tests. Lists of ASD-related genes were obtained from two different ASD databases: the SFARI database (updated on April 17, 2018) (https://gene.sfari.org/) and the AutismKB database (from May 25, 2012) (http://autismkb.cbi.pku.edu.cn/). To determine whether the BPA-responsive genes identified in each transcriptomic study were significantly associated with ASD candidate genes, the list of BPA-responsive genes was overlapped with the list of ASD candidate genes from each ASD database, and a hypergeometric distribution analysis was conducted using the Hypergeometric Distribution Calculator program in the Keisan Online Calculator package (http://keisan.casio.com/exec/system/1180573201). There are four variables in the Hypergeometric Distribution Calculator: number of overlapping genes, total number of DEGs in the experiment, total number of ASD-candidate genes, and total number of genes from RNA-seq analysis.

### Statistical analyses

Statistical analyses were conducted using SPSS version 16.0. The criterion for statistical significance was a P-value < 0.05. A two-tailed Student’s t*-*test was used to determine the statistical significance of differences between the mean values of two groups. A hypergeometric distribution analysis was performed to determine the association of DEGs with ASD candidate genes obtained from the SFARI (https://gene.sfari.org/) and AutismKB (http://autismkb.cbi.pku.edu.cn/) databases using the Hypergeometric Distribution Calculator in the Keisan Online Calculator program (http://keisan.casio.com/exec/system/1180573201). A P-value < 0.05 was considered statistically significant.

### Ethics approval and informed consent

All animal experimental procedures were approved by the Chulalongkorn University Animal Care and Use Committee (Animal Use Protocol No. 1673007 and No. 1773011), Chulalongkorn University.

## Supplementary information

Supplementary Information

## Data Availability

The transcriptome profiling data used in this study have been published in the NCBI GEO DataSets database (GSE44387, GSE63852, GSE58642, GSE50527, GSE58516, and GSE86923). The RNA-seq data will be made publicly available in the GEO upon acceptance of this manuscript for publication.
