# Supplementary material for: Sex Differences in the Effects of Prenatal Bisphenol A Exposure on Genes Associated with Autism Spectrum Disorder in the Hippocampus
Source: Sci Rep. 2019 Feb 28;9:3038. doi: 10.1038/s41598-019-39386-w (PMC6395584; doi:10.1038/s41598-019-39386-w)
Supplement: Supplementary file 1 — Supplementary Information [file 41598_2019_39386_MOESM1_ESM.pdf]

Sex Differences in the Effects of Prenatal Bisphenol A Exposure on Genes Associated with Autism Spectrum Disorder in the Hippocampus

Surangrat Thongkorn[1], Songphon Kanlayaprasit[1], Depicha Jindatip[2], Tewin Tencomnao[3], Valerie W. Hu[4], Tewarit Sarachana[3,*]

[1]The Ph.D. Program in Clinical Biochemistry and Molecular Medicine, Department of Clinical Chemistry, Faculty of Allied Health Sciences, Chulalongkorn University, Bangkok, Thailand.

[2]Department of Anatomy, Faculty of Medicine, Chulalongkorn University, Bangkok, Thailand.

[3]Age-related Inflammation and Degeneration Research Unit, Department of Clinical Chemistry, Faculty of Allied Health Sciences, Chulalongkorn University, Bangkok, Thailand.

[4]Department of Biochemistry and Molecular Medicine, The George Washington University School of Medicine and Health Sciences, The George Washington University, Washington, DC, USA.

*Corresponding author:

Tewarit Sarachana, Ph.D.

Age-related Inflammation and Degeneration Research Unit, Department of Clinical Chemistry, Faculty of Allied Health Sciences, Chulalongkorn University

154 Soi Chula 12, Rama 1 Road, Wangmai, Pathumwan, Bangkok, 10330, Thailand

Tel. 662-218-1081 ext. 313

Fax. 662-218-1082

E-mail: tewarit.sa@chula.ac.th

Supplementary Table S1. The lists of DEGs from RNA-seq analysis of the hippocampi of rats prenatally exposed to BPA

DEGs in Both Sexes			
**ID**	**Symbol**	**Entrez Gene Name**	**Expr Log Ratio**
148675846	FAM114A2	family with sequence similarity 114 member A2	-9.453
672027854	APBB2	amyloid beta precursor protein binding family B member 2	-8.723
564316241	CEP170	centrosomal protein 170	-8.600
149055308	N/A	N/A	-8.147
293347435	PTPRD	protein tyrosine phosphatase, receptor type D	-7.731
149029159	N/A	N/A	-7.551
154090947	NPAS3	neuronal PAS domain protein 3	-7.476
672039093	N/A	N/A	-7.388
564375502	Mxra7	matrix-remodelling associated 7	-7.209
672088045	N/A	N/A	-7.124
293344558	PCNX3	pecanex homolog 3	-7.103
149057336	ZSCAN2	zinc finger and SCAN domain containing 2	-7.087
148694630	N/A	N/A	-6.948
149042655	N/A	N/A	-6.937
392339806	CFAP69	cilia and flagella associated protein 69	-6.820
564302768	KIAA1755	KIAA1755	-6.820
672084304	N/A	N/A	-6.801
564307173	HEATR5A	HEAT repeat containing 5A	-6.735
564310188	IGDCC4	immunoglobulin superfamily DCC subclass member 4	-6.728
564314389	DZIP3	DAZ interacting zinc finger protein 3	-6.700
149026222	N/A	N/A	-6.484
672031167	C19orf57	chromosome 19 open reading frame 57	-6.476
149020633	TAF1D	TATA-box binding protein associated factor, RNA polymerase I subunit D	-6.476
66911867	PHF20L1	PHD finger protein 20 like 1	-6.451
671034187	N/A	N/A	-6.150
672024381	N/A	N/A	-6.150
293341811	PRR14L	proline rich 14 like	-6.129
564320728	Fbxo38	F-box protein 38	-6.109
672017191	N/A	N/A	-6.077
672031484	PCNX2	pecanex homolog 2	-6.044
672062394	N/A	N/A	-5.907
149052470	ZNF454	zinc finger protein 454	-5.700

157818475	SMIM22	small integral membrane protein 22	-5.615
567315993	LOC102550396	LRRGT00188	-5.600
148684403	N/A	N/A	-5.600
355559972	N/A	N/A	-5.476
672063876	MGC116197 (includes others)	similar to RIKEN cDNA 1700001E04	-5.443
564312230	LOC100912948	multidrug resistance-associated protein 1-like	-5.285
568941572	IQSEC1	IQ motif and Sec7 domain 1	-5.248
672029704	ZCCHC6	zinc finger CCHC-type containing 6	-5.211
564313676	FBF1	Fas binding factor 1	-5.170
754169051	EPPIN	epididymal peptidase inhibitor	-5.066
28972369	TOMM70	translocase of outer mitochondrial membrane 70	-5.066
350537755	LYZL4	lysozyme like 4	-5.044
197384778	RGD1311447	LOC363276	-5.044
672035060	CIC	capicua transcriptional repressor	-4.863
157817392	PIRT	phosphoinositide interacting regulator of transient receptor potential channels	-4.833
149053275	N/A	N/A	-4.807
320498033	CD300LD	CD300 molecule like family member d	-4.672
564323305	LOC681300	similar to CXXC finger 5	-4.672
209447125	Ctf2	cardiotrophin 2	-4.392
201860265	NRN1L	neuritin 1 like	-4.358
300798104	IFNLR1	interferon lambda receptor 1	-4.248
966979994	N/A	N/A	-4.248
51591901	MPIG6B	megakaryocyte and platelet inhibitory receptor G6b	-4.170
564298047	GDPD5	glycerophosphodiester phosphodiesterase domain containing 5	-4.163
672075812	N/A	N/A	-4.129
913486723	N/A	N/A	-4.112
53850626	TINAG	tubulointerstitial nephritis antigen	-4.087
71043922	TNFRSF9	TNF receptor superfamily member 9	-4.087
114145538	ARSJ	arylsulfatase family member J	-4.044
300798035	NRG4	neuregulin 4	-4.044
61556838	Raet1d/Raet1e	retinoic acid early transcript 1E	-4.044
564352824	Ago4	argonaute 4, RISC catalytic component	-3.940
114145748	LOC680227	LRRGT00193	-3.907
157819037	MBNL3	muscleblind like splicing regulator 3	-3.907
6978515	APOA1	apolipoprotein A1	-3.807

117647210	CTRC	chymotrypsin C	-3.807
194474016	SLC30A8	solute carrier family 30 member 8	-3.807
564342627	TP53BP1	tumor protein p53 binding protein 1	-3.779
157820153	6030498E09Rik	RIKEN cDNA 6030498E09 gene	-3.755
564315358	N/A	N/A	-3.725
8392926	ASGR2	asialoglycoprotein receptor 2	-3.700
48040447	SUCNR1	succinate receptor 1	-3.700
532078357	N/A	N/A	-3.700
72255533	ANXA8/ANXA8L1	annexin A8 like 1	-3.644
61556786	CCL24	C-C motif chemokine ligand 24	-3.585
194473646	UPK3A	uroplakin 3A	-3.585
149036438	N/A	N/A	-3.546
625217108	N/A	N/A	-3.498
164518908	RAB25	RAB25, member RAS oncogene family	-3.459
27465609	GNAT3	G protein subunit alpha transducin 3	-3.392
157823827	S1PR4	sphingosine-1-phosphate receptor 4	-3.392
564342632	TP53BP1	tumor protein p53 binding protein 1	-3.392
285026465	HS3ST3A1	heparan sulfate-glucosamine 3-sulfotransferase 3A1	-3.322
148710035	PITX3	paired like homeodomain 3	-3.322
148675704	TBX15	T-box 15	-3.268
564324736	L3MBTL3	L3MBTL3, histone methyl-lysine binding protein	-3.262
9506451	CA5A	carbonic anhydrase 5A	-3.248
564375434	CEP295NL	CEP295 N-terminal like	-3.248
156231014	IRX6	iroquois homeobox 6	-3.248
56788780	KRT19	keratin 19	-3.248
564305562	PTPRD	protein tyrosine phosphatase, receptor type D	-3.248
56090299	ODF4	outer dense fiber of sperm tails 4	-3.170
157822159	CCDC42	coiled-coil domain containing 42	-3.170
123173794	GSG1	germ cell associated 1	-3.170
62078965	SLC47A1	solute carrier family 47 member 1	-3.135
157817652	BNC2	basonuclin 2	-3.129
148707849	C1QL2	complement C1q like 2	-3.038
12408310	N5	DNA binding protein N5	-3.000
795466258	N/A	N/A	-3.000
392339806	CFAP69	cilia and flagella associated protein 69	-2.947
149054807	N/A	N/A	-2.925
148704897	MEOX2	mesenchyme homeobox 2	-2.907

57527617	OAS2	2'-5'-oligoadenylate synthetase 2	-2.907
20302091	PLB1	phospholipase B1	-2.907
157821903	Slc7a15	solute carrier family 7 (cationic amino acid transporter, y+ system), member 15	-2.907
564355126	ADGRF3	adhesion G protein-coupled receptor F3	-2.907
77917534	CBLC	Cbl proto-oncogene C	-2.807
347800717	ILDR1	immunoglobulin like domain containing receptor 1	-2.807
20302109	SLC22A9	solute carrier family 22 member 9	-2.807
672022282	KIF21A	kinesin family member 21A	-2.790
564310551	N/A	N/A	-2.785
157822121	LRMDA	leucine rich melanocyte differentiation associated	-2.747
291327518	AVP	arginine vasopressin	-2.734
157822377	ERICH4	glutamate rich 4	-2.716
156071424	Vom2r18 (includes others)	vomeronasal 2 receptor, 18	-2.700
51948496	SLC22A18	solute carrier family 22 member 18	-2.687
672032217	REPS2	RALBP1 associated Eps domain containing 2	-2.683
564313782	Tha1	threonine aldolase 1	-2.683
68063179	N/A	N/A	-2.651
295391779	LOC100363112	Aa2-296-like	-2.644
8567332	CALCB	calcitonin related polypeptide beta	-2.636
293351303	METTL22	methyltransferase like 22	-2.628
306482632	BPIFB4	BPI fold containing family B member 4	-2.585
298566276	Ces1a	carboxylesterase 1A	-2.585
57222300	Klra2	killer cell lectin-like receptor, subfamily A, member 2	-2.585
157786772	KREMEN2	kringle containing transmembrane protein 2	-2.585
157822463	Nkx6-3	NK6 homeobox 3	-2.585
57222328	PFN4	profilin family member 4	-2.585
148703035	CLDN11	claudin 11	-2.549
56090537	Ccl7	chemokine (C-C motif) ligand 7	-2.524
157822853	GUCA1A	guanylate cyclase activator 1A	-2.509
884945546	N/A	N/A	-2.507
6978679	COMP	cartilage oligomeric matrix protein	-2.503
13928714	Ccl2	chemokine (C-C motif) ligand 2	-2.497
157822321	KCNV2	potassium voltage-gated channel modifier subfamily V member 2	-2.459

162417984	Vom2r12 (includes others)	vomeronasal 2 receptor, 80	-2.459
672082610	N/A	N/A	-2.457
157818895	RNF225	ring finger protein 225	-2.451
564391680	MAP3K8	mitogen-activated protein kinase kinase kinase 8	-2.426
149053857	LOC100363423	rCG35357-like	-2.421
157821307	BNC1	basonuclin 1	-2.415
11120724	Sult1d1	sulfotransferase family 1D, member 1	-2.415
68163381	CALHM5	calcium homeostasis modulator family member 5	-2.392
672023922	VWA3B	von Willebrand factor A domain containing 3B	-2.390
564297387	Zfp658	zinc finger protein 658	-2.369
187469451	CLEC7A	C-type lectin domain containing 7A	-2.322
62945342	LAX1	lymphocyte transmembrane adaptor 1	-2.322
187282171	SLC22A13	solute carrier family 22 member 13	-2.322
392334411	ANKRD11	ankyrin repeat domain 11	-2.301
112983998	C10orf62	chromosome 10 open reading frame 62	-2.273
568914626	GARNL3	GTPase activating Rap/RanGAP domain like 3	-2.261
148679862	SLC35F3	solute carrier family 35 member F3	-2.261
672053062	FKBP15	FK506 binding protein 15	-2.254
387157880	CLRN1	clarin 1	-2.248
157820151	ERAS	ES cell expressed Ras	-2.248
62079023	ADTRP	androgen dependent TFPI regulating protein	-2.246
672028646	N/A	N/A	-2.233
564320724	Fbxo38	F-box protein 38	-2.220
13242279	GJA3	gap junction protein alpha 3	-2.209
8393418	GAPDH	glyceraldehyde-3-phosphate dehydrogenase	-2.197
198278465	DYDC2	DPY30 domain containing 2	-2.190
157822087	ACTRT3	actin related protein T3	-2.170
39930539	MYOCD	myocardin	-2.170
40786453	ABCC12	ATP binding cassette subfamily C member 12	-2.170
300798413	FSD2	fibronectin type III and SPRY domain containing 2	-2.170
404247470	OLR1	oxidized low density lipoprotein receptor 1	-2.170
66730349	SPIB	Spi-B transcription factor	-2.170
254675145	STPG4	sperm-tail PG-rich repeat containing 4	-2.170
60223047	DNTT	DNA nucleotidylexotransferase	-2.147
392334596	RSPH3	radial spoke head 3 homolog	-2.138
88759335	IRX2	iroquois homeobox 2	-2.138

149046043	N/A	N/A	-2.130
25742828	SCN7A	sodium voltage-gated channel alpha subunit 7	-2.129
148702471	N/A	N/A	-2.127
148669850	GFRA1	GDNF family receptor alpha 1	-2.118
58331126	GJB6	gap junction protein beta 6	-2.104
62078837	ABHD16B	abhydrolase domain containing 16B	-2.100
672088045	N/A	N/A	-2.097
300797305	TMEM45A	transmembrane protein 45A	-2.093
672023280	N/A	N/A	-2.092
398650658	TG	thyroglobulin	-2.087
148672128	SMAGP	small cell adhesion glycoprotein	-2.077
187937026	NCF4	neutrophil cytosolic factor 4	-2.062
19923094	OSGIN1	oxidative stress induced growth inhibitor 1	-2.059
564388803	SLC18A1	solute carrier family 18 member A1	-2.059
675294847	ATP12A	ATPase H+/K+ transporting non-gastric alpha2 subunit	-2.051
672089449	N/A	N/A	-2.050
149041058	RCOR3	REST corepressor 3	-2.026
55741882	ZPBP2	zona pellucida binding protein 2	-2.022
19424304	CHRNB3	cholinergic receptor nicotinic beta 3 subunit	-2.000
117647206	DDX4	DEAD-box helicase 4	-2.000
156119589	FOXC2	forkhead box C2	-2.000
157822587	PDE6B	phosphodiesterase 6B	-2.000
296483047	SIX1	SIX homeobox 1	-2.000
157818091	TMEM182	transmembrane protein 182	-2.000
987396306	N/A	N/A	-2.000
672023560	Pot1b	protection of telomeres 1B	-2.000
47577151	Olfr1441	olfactory receptor 1441	-1.972
672014371	N/A	N/A	-1.963
56676350	PRSS35	protease, serine 35	-1.962
18543365	FGF21	fibroblast growth factor 21	-1.948
18543367	FGF19	fibroblast growth factor 19	-1.945
157820583	ANKRD34C	ankyrin repeat domain 34C	-1.939
84781670	MCOLN2	mucolipin 2	-1.939
149066014	PVALB	parvalbumin	-1.939
564321260	N/A	N/A	-1.938
564378170	PAN3	PAN3 poly(A) specific ribonuclease subunit	-1.932
157820217	Gsta4	glutathione S-transferase, alpha 4	-1.930
157818655	MPZL2	myelin protein zero like 2	-1.922
21326469	SLC15A3	solute carrier family 15 member 3	-1.921

564380050	2410141K09Rik	RIKEN cDNA 2410141K09 gene	-1.920
13540671	CNMD	chondromodulin	-1.918
58000421	Ggnbp1	gametogenetin binding protein 1	-1.918
117647214	EDN3	endothelin 3	-1.913
157823875	EPS8L1	EPS8 like 1	-1.907
114052553	RGS18	regulator of G protein signaling 18	-1.902
81295367	Abcg3	ATP binding cassette subfamily G member 3	-1.898
19705467	Cyp2t4	cytochrome P450, family 2, subfamily t, polypeptide 4	-1.898
157820331	CAPNS2	calpain small subunit 2	-1.893
672068485	N/A	N/A	-1.890
672035210	PRR19	proline rich 19	-1.888
6981154	Lgals5	lectin, galactose binding, soluble 5	-1.884
157787076	SLAMF8	SLAM family member 8	-1.883
454526968	PDE4C	phosphodiesterase 4C	-1.874
71043614	SDCBP2	syndecan binding protein 2	-1.874
149040268	N/A	N/A	-1.874
71043672	FAM166A	family with sequence similarity 166 member A	-1.865
11067389	BMP15	bone morphogenetic protein 15	-1.858
149060001	N/A	N/A	-1.858
672029702	ZCCHC6	zinc finger CCHC-type containing 6	-1.854
564309742	IGSF9B	immunoglobulin superfamily member 9B	-1.850
13592031	PTGER2	prostaglandin E receptor 2	-1.845
297374767	TPSAB1/TPSB2	tryptase alpha/beta 1	-1.834
16758014	HPX	hemopexin	-1.830
18543363	FGF22	fibroblast growth factor 22	-1.830
149054577	CD79B	CD79b molecule	-1.826
569009290	TENM1	teneurin transmembrane protein 1	-1.824
970699122	N/A	N/A	-1.824
149064260	PRDM6	PR/SET domain 6	-1.816
226698394	UNC80	unc-80 homolog, NALCN channel complex subunit	-1.781
300797073	CCDC27	coiled-coil domain containing 27	-1.778
157819477	GLOD5	glyoxalase domain containing 5	-1.768
672079259	N/A	N/A	-1.758
57222306	Oas1f	2'-5' oligoadenylate synthetase 1F	-1.755
19424240	PCSK4	proprotein convertase subtilisin/kexin type 4	-1.740
480306394	Mcpt4	mast cell protease 4	-1.739
149029798	Tmc4	transmembrane channel-like 4	-1.737
300795532	PLSCR5	phospholipid scramblase family member 5	-1.727

6981344	PDC	phosducin	-1.722
71043698	SYTL1	synaptotagmin like 1	-1.716
157820179	TSNAXIP1	translin associated factor X interacting protein 1	-1.713
54019438	PCDHAC1	protocadherin alpha subfamily C, 1	-1.704
31745164	HAX1	HCLS1 associated protein X-1	-1.703
300796107	PROX2	prospero homeobox 2	-1.700
672020363	N/A	N/A	-1.694
157816965	DKK2	dickkopf WNT signaling pathway inhibitor 2	-1.694
564296517	N/A	N/A	-1.688
157819871	EPB42	erythrocyte membrane protein band 4.2	-1.678
157822811	Fmo9	flavin containing monooxygenase 9	-1.678
817473312	GATA6	GATA binding protein 6	-1.678
9910234	IFIT1B	interferon induced protein with tetratricopeptide repeats 1B	-1.678
392331668	HAGHL	hydroxyacylglutathione hydrolase like	-1.665
564306888	N/A	N/A	-1.661
300795403	ODF3B	outer dense fiber of sperm tails 3B	-1.659
153792385	Vom2r34	vomeronasal 2 receptor, 34	-1.648
672019578	MYSM1	Myb like, SWIRM and MPN domains 1	-1.645
564301284	Ttf1	transcription termination factor, RNA polymerase I	-1.633
6981098	IL2RB	interleukin 2 receptor subunit beta	-1.632
505771867	N/A	N/A	-1.624
148671981	NFE2	nuclear factor, erythroid 2	-1.619
31542125	ALOX15	arachidonate 15-lipoxygenase	-1.611
58331159	GSTA3	glutathione S-transferase alpha 3	-1.603
76443687	SLC4A1	solute carrier family 4 member 1 (Diego blood group)	-1.599
157821527	RHOD	ras homolog family member D	-1.597
158711755	C17orf97	chromosome 17 open reading frame 97	-1.592
568964944	EPB41L2	erythrocyte membrane protein band 4.1 like 2	-1.590
295391913	LOC100366054	Da1-10-like	-1.590
117647198	CFD	complement factor D	-1.585
71896590	AOC3	amine oxidase, copper containing 3	-1.585
815890866	HNF1B	HNF1 homeobox B	-1.585
209870037	INSRR	insulin receptor related receptor	-1.585
62079089	MALL	mal, T cell differentiation protein like	-1.585
281604096	MYH8	myosin heavy chain 8	-1.585
157819393	NNMT	nicotinamide N-methyltransferase	-1.585

564328425	TPH1	tryptophan hydroxylase 1	-1.585
19745192	ATP2C2	ATPase secretory pathway Ca2+ transporting 2	-1.585
194294538	CACNA1S	calcium voltage-gated channel subunit alpha1 S	-1.585
157818603	CLCA2	chloride channel accessory 2	-1.585
685156911	NLRP4	NLR family pyrin domain containing 4	-1.585
194473618	SCX	scleraxis bHLH transcription factor	-1.585
149041966	N/A	N/A	-1.585
564378315	Zfp853	zinc finger protein 853	-1.574
392332008	SLC38A10	solute carrier family 38 member 10	-1.568
564312886	DNAH2	dynein axonemal heavy chain 2	-1.566
194239635	Tpsab1	tryptase alpha/beta 1	-1.563
564379810	TMEM119	transmembrane protein 119	-1.562
149034165	GALNT15	polypeptide N-acetylgalactosaminyltransferase 15	-1.561
672082610	N/A	N/A	-1.557
13929066	CPZ	carboxypeptidase Z	-1.549
6978837	FGF10	fibroblast growth factor 10	-1.548
16758884	Cd52	CD52 antigen	-1.546
28972714	KLHL13	kelch like family member 13	-1.539
157820611	INSC	INSC, spindle orientation adaptor protein	-1.536
6981326	S100A4	S100 calcium binding protein A4	-1.532
21426773	ASPG	asparaginase	-1.524
300798371	MCTP2	multiple C2 and transmembrane domain containing 2	-1.524
149050087	N/A	N/A	-1.524
157819105	C2orf73	chromosome 2 open reading frame 73	-1.515
149025029	SUSD6	sushi domain containing 6	-1.512
158186711	F13A1	coagulation factor XIII A chain	-1.510
157821867	BMP8A	bone morphogenetic protein 8a	-1.509
281371353	IL17B	interleukin 17B	-1.507
840084406	Lypd2	Ly6/Plaur domain containing 2	-1.506
155369702	ECHDC3	enoyl-CoA hydratase domain containing 3	-1.497
74202463	EYA2	EYA transcriptional coactivator and phosphatase 2	-1.497
72255569	Abca17	ATP-binding cassette, sub-family A (ABC1), member 17	-1.495
157822647	CD70	CD70 molecule	-1.492
153791855	Vmn2r116 (includes others)	vomeronasal 2, receptor 116	-1.485
157820579	Lrrc51	leucine rich repeat containing 51	-1.481

157820541	RGS9BP	regulator of G protein signaling 9 binding protein	-1.480
13929126	GALNT5	polypeptide N-acetylgalactosaminyltransferase 5	-1.478
194474002	MEI1	meiotic double-stranded break formation protein 1	-1.476
672028287	Kat6b	K(lysine) acetyltransferase 6B	-1.476
94400795	PRLHR	prolactin releasing hormone receptor	-1.474
162138928	SLC13A3	solute carrier family 13 member 3	-1.467
71043750	SYNGR4	synaptogyrin 4	-1.464
242397466	CTXN2	cortexin 2	-1.461
16758434	DAO	D-amino acid oxidase	-1.459
13994171	SLC22A2	solute carrier family 22 member 2	-1.459
564305934	BTBD19	BTB domain containing 19	-1.458
18677739	CDKN2B	cyclin dependent kinase inhibitor 2B	-1.454
157823445	PABPC4L	poly(A) binding protein cytoplasmic 4 like	-1.453
81295349	SLC52A3	solute carrier family 52 member 3	-1.453
148704234	GJB2	gap junction protein beta 2	-1.451
564357330	N/A	N/A	-1.446
157820117	FBXL8	F-box and leucine rich repeat protein 8	-1.441
210032999	MBOAT4	membrane bound O-acyltransferase domain containing 4	-1.441
675801992	N/A	N/A	-1.441
149052949	N/A	N/A	-1.436
672065933	DOCK10	dedicator of cytokinesis 10	-1.436
6981148	LEP	leptin	-1.436
564317562	CFAP70	cilia and flagella associated protein 70	-1.433
157818865	INCA1	inhibitor of CDK, cyclin A1 interacting protein 1	-1.433
283806636	ZNF831	zinc finger protein 831	-1.433
31377521	S1PR5	sphingosine-1-phosphate receptor 5	-1.426
672019901	LOC100361036	rCG31267-like	-1.420
1698696	CMA1	chymase 1	-1.413
77917586	GRAP2	GRB2-related adaptor protein 2	-1.412
157818369	Hils1	histone H1-like protein in spermatids 1	-1.411
752993027	HSPB1	heat shock protein family B (small) member 1	-1.410
672042718	ZBBX	zinc finger B-box domain containing	-1.409
990011509	N/A	N/A	-1.409
19924087	Akr1c14	aldo-keto reductase family 1, member C14	-1.406
158187526	TFEC	transcription factor EC	-1.402

157820935	Prss32	protease, serine 32	-1.392
157822105	SLC49A3	solute carrier family 49 member 3	-1.389
293341722	N/A	N/A	-1.387
564314671	VPS8	VPS8, CORVET complex subunit	-1.386
157818431	PPL	periplakin	-1.385
157823809	CD163	CD163 molecule	-1.385
149065466	ARHGEF5	Rho guanine nucleotide exchange factor 5	-1.382
18426832	IL23A	interleukin 23 subunit alpha	-1.381
564352410	ARTN	artemin	-1.379
157952196	Tmem125	transmembrane protein 125	-1.379
149038394	N/A	N/A	-1.379
157822063	Gm6377	predicted gene 6377	-1.379
48675870	PPP1R3B	protein phosphatase 1 regulatory subunit 3B	-1.379
166157470	PSTPIP1	proline-serine-threonine phosphatase interacting protein 1	-1.376
209870097	NLRP6	NLR family pyrin domain containing 6	-1.375
157818677	ALX4	ALX homeobox 4	-1.372
57526854	IFI35	interferon induced protein 35	-1.369
119709837	SLC15A1	solute carrier family 15 member 1	-1.364
568994911	PHLDB2	pleckstrin homology like domain family B member 2	-1.350
148702636	SPHK1	sphingosine kinase 1	-1.348
340523096	IL10RA	interleukin 10 receptor subunit alpha	-1.347
167830415	Dcdc5	doublecortin domain containing 5	-1.346
827012496	NLRC4	NLR family CARD domain containing 4	-1.346
672025117	MBTD1	mbt domain containing 1	-1.344
6978717	CTRB2	chymotrypsinogen B2	-1.342
149020413	Zfp599	zinc finger protein 599	-1.342
20806113	BAMBI	BMP and activin membrane bound inhibitor	-1.338
157786780	MELTF	melanotransferrin	-1.333
29789044	SNAI2	snail family transcriptional repressor 2	-1.328
148704682	N/A	N/A	-1.326
158187533	ABCA13	ATP binding cassette subfamily A member 13	-1.322
157786628	ALOX12	arachidonate 12-lipoxygenase, 12S type	-1.322
672070295	BAHCC1	BAH domain and coiled-coil containing 1	-1.322
157823345	LRR1	leucine rich repeat protein 1	-1.322
282847351	LRRC36	leucine rich repeat containing 36	-1.322
307746876	Pzp	PZP, alpha-2-macroglobulin like	-1.322
71043730	VNN1	vanin 1	-1.322
149047788	ACVR1C	activin A receptor type 1C	-1.322

672071361	CFAP44	cilia and flagella associated protein 44	-1.322
293346251	TMEM62	transmembrane protein 62	-1.322
300794534	WDR93	WD repeat domain 93	-1.322
149037646	N/A	N/A	-1.316
226958688	RBP4	retinol binding protein 4	-1.310
148689488	SYN3	synapsin III	-1.308
157824214	FAM46B	family with sequence similarity 46 member B	-1.301
1236083	LSR	lipolysis stimulated lipoprotein receptor	-1.290
61556810	ADPRHL1	ADP-ribosylhydrolase like 1	-1.285
58865654	EFEMP1	EGF containing fibulin extracellular matrix protein 1	-1.284
57114286	HLA-DRB5	major histocompatibility complex, class II, DR beta 5	-1.284
816197606	HTR6	5-hydroxytryptamine receptor 6	-1.283
66730461	Clec2d (includes others)	C-type lectin domain family 2, member D	-1.282
209364566	COL4A3	collagen type IV alpha 3 chain	-1.280
16758506	SLFN12	schlafen family member 12	-1.280
568950651	CYP2R1	cytochrome P450 family 2 subfamily R member 1	-1.277
148694035	SENP8	SUMO/sentrin peptidase family member, NEDD8 specific	-1.276
409264670	DHRS7C	dehydrogenase/reductase 7C	-1.273
71043878	PROCR	protein C receptor	-1.270
62656582	KIAA0100	KIAA0100	-1.266
2231145	N/A	N/A	-1.264
13162326	SLC27A5	solute carrier family 27 member 5	-1.263
672065139	N/A	N/A	-1.263
162287073	CRYBB1	crystallin beta B1	-1.263
38303879	LIPH	lipase H	-1.263
157821835	SCEL	sciellin	-1.263
149034697	N/A	N/A	-1.263
672034215	ZNF729	zinc finger protein 729	-1.256
158186720	SLC12A3	solute carrier family 12 member 3	-1.254
564312627	ZFP62	ZFP62 zinc finger protein	-1.253
40018582	LARGE2	LARGE xylosyl- and glucuronyltransferase 2	-1.252
157821877	ATP8B1	ATPase phospholipid transporting 8B1	-1.248
9910536	RNASE4	ribonuclease A family member 4	-1.245
6981068	ICAM1	intercellular adhesion molecule 1	-1.240
9506461	CAPN1	calpain 1	-1.237

672074150	N/A	N/A	-1.230
8392900	RUNX1	runt related transcription factor 1	-1.222
157819007	TBXT	T-box transcription factor T	-1.222
67078412	LRRC63	leucine rich repeat containing 63	-1.222
672019544	N/A	N/A	-1.222
187281975	DENND1C	DENN domain containing 1C	-1.216
672029435	N/A	N/A	-1.216
224500890	C1orf228	chromosome 1 open reading frame 228	-1.209
16758338	FTCD	formimidoyltransferase cyclodeaminase	-1.208
157824077	CCRL2	C-C motif chemokine receptor like 2	-1.206
157819399	NOXO1	NADPH oxidase organizer 1	-1.206
157819313	RGD1561661	similar to Ferritin light chain (Ferritin L subunit)	-1.206
672038049	N/A	N/A	-1.206
56119141	BTK	Bruton tyrosine kinase	-1.205
157820267	MEI4	meiotic double-stranded break formation protein 4	-1.205
58865664	SH2D4A	SH2 domain containing 4A	-1.204
50979278	IL22RA2	interleukin 22 receptor subunit alpha 2	-1.202
8393742	MAG	myelin associated glycoprotein	-1.202
62945350	C4orf36	chromosome 4 open reading frame 36	-1.198
672040664	CFAP43	cilia and flagella associated protein 43	-1.198
155369293	AEBP1	AE binding protein 1	-1.198
218505769	Zfp7	zinc finger protein 7	-1.191
564300462	DCHS2	dachsous cadherin-related 2	-1.190
157820725	SUN5	Sad1 and UNC84 domain containing 5	-1.187
672033933	N/A	N/A	-1.186
568924481	COL25A1	collagen type XXV alpha 1 chain	-1.185
6981332	SERPINE1	serpin family E member 1	-1.184
564393142	WDR36	WD repeat domain 36	-1.184
13928752	PTGIS	prostaglandin I2 synthase	-1.183
149408137	DHX58	DExH-box helicase 58	-1.183
300797201	ENDOU	endonuclease, poly(U) specific	-1.181
58866008	TMC5	transmembrane channel like 5	-1.180
672037923	N/A	N/A	-1.178
392334475	RGD1560020_predicted	similar to Myb proto-oncogene protein (C-myb) (predicted)	-1.175
674082951	N/A	N/A	-1.172
157820699	ANGPT4	angiopoietin 4	-1.170
6981590	IL1RL1	interleukin 1 receptor like 1	-1.170

300795079	STOX1	storkhead box 1	-1.170
71795615	UPP1	uridine phosphorylase 1	-1.167
281332082	THBS2	thrombospondin 2	-1.164
58865684	MCOLN3	mucolipin 3	-1.158
256574780	TMEM212	transmembrane protein 212	-1.158
11968076	RHCE/RHD	Rh blood group D antigen	-1.158
406035319	KIRREL2	kirre like nephrin family adhesion molecule 2	-1.154
564311678	PLEKHM3	pleckstrin homology domain containing M3	-1.152
564353313	N/A	N/A	-1.144
282721071	4931409K22Rik	RIKEN cDNA 4931409K22 gene	-1.138
157818125	CYP39A1	cytochrome P450 family 39 subfamily A member 1	-1.138
126723022	NEU2	neuraminidase 2	-1.138
157820223	TBX4	T-box 4	-1.138
157823801	SLC50A1	solute carrier family 50 member 1	-1.136
60223053	SEPT1	septin 1	-1.133
148664561	DTNA	dystrobrevin alpha	-1.133
218156285	CFB	complement factor B	-1.130
51948494	ITGB6	integrin subunit beta 6	-1.129
57527560	TMEM140	transmembrane protein 140	-1.128
392331829	ATAD5	ATPase family, AAA domain containing 5	-1.122
157823789	PIK3AP1	phosphoinositide-3-kinase adaptor protein 1	-1.113
672017085	N/A	N/A	-1.111
125347412	FAM72A	family with sequence similarity 72 member A	-1.106
672034189	N/A	N/A	-1.100
300798739	MYO3B	myosin IIIB	-1.100
594061361	N/A	N/A	-1.098
149053909	COL1A1	collagen type I alpha 1 chain	-1.092
564315753	N/A	N/A	-1.092
197385083	C1orf194	chromosome 1 open reading frame 194	-1.090
67078444	EPN3	epsin 3	-1.090
156347833	N/A	N/A	-1.090
24308484	SLC17A8	solute carrier family 17 member 8	-1.089
157819247	CPA4	carboxypeptidase A4	-1.087
281371494	LAMC2	laminin subunit gamma 2	-1.087
564310671	NBEAL2	neurobeachin like 2	-1.084
320089574	FAM161A	family with sequence similarity 161 member A	-1.080
300797017	ATG16L2	autophagy related 16 like 2	-1.079
157818961	UBA7	ubiquitin like modifier activating enzyme 7	-1.078
70608161	REEP4	receptor accessory protein 4	-1.076

9910378	CDC42SE2	CDC42 small effector 2	-1.069
564365960	KIF9	kinesin family member 9	-1.066
18959230	SLC6A20	solute carrier family 6 member 20	-1.066
197384727	Smco4	single-pass membrane protein with coiled-coil domains 4	-1.065
197386139	SSC5D	scavenger receptor cysteine rich family member with 5 domains	-1.060
564358455	RASAL3	RAS protein activator like 3	-1.052
672020326	MTA3	metastasis associated 1 family member 3	-1.047
676272056	N/A	N/A	-1.046
189011701	PPP1R42	protein phosphatase 1 regulatory subunit 42	-1.044
58865636	ART3	ADP-ribosyltransferase 3	-1.042
66730473	DNAI1	dynein axonemal intermediate chain 1	-1.042
164448680	HBB	hemoglobin subunit beta	-1.042
157819141	C6orf141	chromosome 6 open reading frame 141	-1.040
11560040	PTGDR	prostaglandin D2 receptor	-1.037
157817410	ZNF474	zinc finger protein 474	-1.034
672015005	N/A	N/A	-1.033
149064010	LTB4R2	leukotriene B4 receptor 2	-1.032
157822059	HACD4	3-hydroxyacyl-CoA dehydratase 4	-1.031
672085160	N/A	N/A	-1.029
32189322	ADGRG2	adhesion G protein-coupled receptor G2	-1.027
7106240	AKR7A3	aldo-keto reductase family 7 member A3	-1.026
68226711	LOC310926	hypothetical protein LOC310926	-1.023
564310624	CDHR4	cadherin related family member 4	-1.021
297206838	ARNTL2	aryl hydrocarbon receptor nuclear translocator like 2	-1.020
58865898	LIMS2	LIM zinc finger domain containing 2	-1.018
157821423	TBX6	T-box 6	-1.017
672033554	LOC102557335	uncharacterized LOC102557335	-1.015
300797728	MGST3	microsomal glutathione S-transferase 3	-1.011
564296180	CEP72	centrosomal protein 72	-1.005
68163493	DAW1	dynein assembly factor with WD repeats 1	-1.005
189181712	CFAP161	cilia and flagella associated protein 161	-1.003
1778213	HSPD1	heat shock protein family D (Hsp60) member 1	-1.002
564317722	N/A	N/A	-1.002
62078849	USP18	ubiquitin specific peptidase 18	-1.000
25453414	ASS1	argininosuccinate synthase 1	-1.000
564380463	CDK18	cyclin dependent kinase 18	-1.000
13929148	CNGB1	cyclic nucleotide gated channel beta 1	-1.000

197384591	CYTL1	cytokine like 1	-1.000
149025238	ESRRB	estrogen related receptor beta	-1.000
392342053	GK5	glycerol kinase 5 (putative)	-1.000
198386353	MYO1G	myosin IG	-1.000
156231008	PRND	prion like protein doppel	-1.000
149056745	N/A	N/A	-1.000
672067248	N/A	N/A	-1.000
765826426	Acot6	acyl-CoA thioesterase 6	-1.000
59676595	FAM20A	FAM20A, golgi associated secretory pathway pseudokinase	-1.000
149054281	CNTNAP1	contactin associated protein 1	-0.989
537234259	N/A	N/A	-0.988
563404456	CCL3L3	C-C motif chemokine ligand 3 like 3	-0.986
569009290	TENM1	teneurin transmembrane protein 1	-0.985
672076451	NCAPG	non-SMC condensin I complex subunit G	-0.984
880889841	N/A	N/A	-0.983
300795496	LAYN	layilin	-0.983
158341649	FAM227B	family with sequence similarity 227 member B	-0.981
148679699	N/A	N/A	-0.981
564324583	N/A	N/A	-0.980
407228396	THEMIS2	thymocyte selection associated family member 2	-0.980
157817157	C2orf70	chromosome 2 open reading frame 70	-0.979
672085160	N/A	N/A	-0.978
942065067	N/A	N/A	-0.977
215276950	PKP2	plakophilin 2	-0.973
20302089	GABRR3	gamma-aminobutyric acid type A receptor rho3 subunit (gene/pseudogene)	-0.972
68163517	CCDC146	coiled-coil domain containing 146	-0.971
149053793	TSPOAP1	TSPO associated protein 1	-0.970
564356749	N/A	N/A	-0.969
564340633	OLFML2A	olfactomedin like 2A	-0.968
157819465	CLEC9A	C-type lectin domain containing 9A	-0.967
564349125	TEAD4	TEA domain transcription factor 4	-0.967
157819701	Ctla2a	cytotoxic T lymphocyte-associated protein 2 alpha	-0.966
157819493	Igbp1b	immunoglobulin (CD79A) binding protein 1b	-0.966
20806163	CKLF	chemokine like factor	-0.962
672033256	LOC100912904	disks large homolog 5-like	-0.960
395826410	N/A	N/A	-0.959

157820951	PRSS53	protease, serine 53	-0.959
564326123	CDC42EP5	CDC42 effector protein 5	-0.958
157817989	MDFI	MyoD family inhibitor	-0.958
16758344	SEC16B	SEC16 homolog B, endoplasmic reticulum export factor	-0.958
672052120	RBM12B	RNA binding motif protein 12B	-0.957
564392197	LOC684327	similar to inter-alpha (globulin) inhibitor H5	-0.954
156231010	CLDN6	claudin 6	-0.954
157818989	LRRC71	leucine rich repeat containing 71	-0.952
157818275	KCNG4	potassium voltage-gated channel modifier subfamily G member 4	-0.952
157820109	SLC43A1	solute carrier family 43 member 1	-0.950
24638442	RLN3	relaxin 3	-0.949
157823055	EGFL6	EGF like domain multiple 6	-0.947
672062795	N/A	N/A	-0.944
9506907	MSTN	myostatin	-0.943
392339435	WDR76	WD repeat domain 76	-0.943
124486586	AUTS2	AUTS2, activator of transcription and developmental regulator	-0.941
564299864	N/A	N/A	-0.940
148680846	HIC1	HIC ZBTB transcriptional repressor 1	-0.940
157823079	RBKS	ribokinase	-0.939
149035008	Grifin	galectin-related inter-fiber protein	-0.936
198442873	CDC14A	cell division cycle 14A	-0.936
672071361	CFAP44	cilia and flagella associated protein 44	-0.934
16758094	FABP4	fatty acid binding protein 4	-0.933
564315207	TMEM270	transmembrane protein 270	-0.933
672088045	N/A	N/A	-0.932
71043648	LOC499742	LRRG00137	-0.931
6981176	MAK	male germ cell associated kinase	-0.931
40254742	NCF1	neutrophil cytosolic factor 1	-0.929
62945330	SLC8B1	solute carrier family 8 member B1	-0.929
564395350	N/A	N/A	-0.927
532048106	N/A	N/A	-0.926
149043385	N/A	N/A	-0.925
50657416	C1RL	complement C1r subcomponent like	-0.918
58372166	HAPLN2	hyaluronan and proteoglycan link protein 2	-0.918
157819487	TACO1	translational activator of cytochrome c oxidase I	-0.917
77993372	PQLC3	PQ loop repeat containing 3	-0.917

47059114	LTB	lymphotoxin beta	-0.916
29789140	GRM6	glutamate metabotropic receptor 6	-0.916
293347270	OSGIN2	oxidative stress induced growth inhibitor family member 2	-0.915
564345430	LOC100910079	actin-related protein 3B-like	-0.913
164663906	PDIA2	protein disulfide isomerase family A member 2	-0.909
157821719	CPM	carboxypeptidase M	-0.908
148677539	N/A	N/A	-0.908
207446698	FA2H	fatty acid 2-hydroxylase	-0.907
564325648	Zfp54	zinc finger protein 54	-0.907
564318923	WDR17	WD repeat domain 17	-0.904
149050847	POMC	proopiomelanocortin	-0.904
58865836	SP140	SP140 nuclear body protein	-0.903
589937133	N/A	N/A	-0.902
16758080	COL1A2	collagen type I alpha 2 chain	-0.900
157819945	ARPIN/C15orf38-AP3S2	C15orf38-AP3S2 readthrough	-0.899
672016875	LOC103690320	FERM and PDZ domain-containing protein 3	-0.897
564382020	TMEM63A	transmembrane protein 63A	-0.896
564342055	Apip	APAF1 interacting protein	-0.894
47576123	Olr1387/Olr1388	olfactory receptor 1387	-0.893
157822605	OTOR	otoraplin	-0.893
14861868	Ptprv	protein tyrosine phosphatase, receptor type, V	-0.893
13540697	NOV	nephroblastoma overexpressed	-0.893
38454316	TLR3	toll like receptor 3	-0.891
329755323	ATP13A4	ATPase 13A4	-0.889
157824138	LURAP1	leucine rich adaptor protein 1	-0.888
12621098	EPHX2	epoxide hydrolase 2	-0.887
157821417	KERA	keratocan	-0.885
13540656	EMP3	epithelial membrane protein 3	-0.883
564377419	THPO	thrombopoietin	-0.882
149069514	N/A	N/A	-0.881
657940868	PALLD	palladin, cytoskeletal associated protein	-0.881
407228403	EVI2B	ecotropic viral integration site 2B	-0.880
149056256	FXYD5	FXYD domain containing ion transport regulator 5	-0.880
148670853	BBOF1	basal body orientation factor 1	-0.878
392351290	DNAH9	dynein axonemal heavy chain 9	-0.875
19424166	FBXO32	F-box protein 32	-0.874
564312627	ZFP62	ZFP62 zinc finger protein	-0.871

209870105	GPR37L1	G protein-coupled receptor 37 like 1	-0.870
8393123	CHRM5	cholinergic receptor muscarinic 5	-0.869
564321163	CHD9	chromodomain helicase DNA binding protein 9	-0.868
157817185	PPCDC	phosphopantothenoylcysteine decarboxylase	-0.866
672036437	ACP4	acid phosphatase 4	-0.862
3420792	N/A	N/A	-0.862
57114338	SCN4B	sodium voltage-gated channel beta subunit 4	-0.859
672022227	RGS22	regulator of G protein signaling 22	-0.855
148747412	CBWD1	COBW domain containing 1	-0.855
62945324	LCA5L	LCA5L, lebercilin like	-0.853
149031998	ACVRL1	activin A receptor like type 1	-0.852
672076690	N/A	N/A	-0.851
6978791	EDN1	endothelin 1	-0.850
119226204	CFAP206	cilia and flagella associated protein 206	-0.849
201861483	LOC102548396 (includes others)	zinc finger protein 951	-0.848
148690904	NTF4	neurotrophin 4	-0.848
261337179	TMPRSS7	transmembrane protease, serine 7	-0.848
77020254	GPR182	G protein-coupled receptor 182	-0.845
672029530	NEK5	NIMA related kinase 5	-0.842
672087474	REPS2	RALBP1 associated Eps domain containing 2	-0.841
672087474	REPS2	RALBP1 associated Eps domain containing 2	-0.841
61557366	SELPLG	selectin P ligand	-0.841
672064087	N/A	N/A	-0.841
392340768	DISP3	dispatched RND transporter family member 3	-0.840
227116255	P2RX6	purinergic receptor P2X 6	-0.838
38454288	P4HA3	prolyl 4-hydroxylase subunit alpha 3	-0.838
564311658	N/A	N/A	-0.837
6980958	SLC2A4	solute carrier family 2 member 4	-0.837
157822283	HS3ST6	heparan sulfate-glucosamine 3-sulfotransferase 6	-0.835
564387882	IL17RB	interleukin 17 receptor B	-0.835
392334002	CCDC3	coiled-coil domain containing 3	-0.833
564366187	LOC100361039 (includes others)	similar to nidogen 2	-0.833
157822457	SYNC	syncoilin, intermediate filament protein	-0.833
564373460	SLFN13	schlafen family member 13	-0.831
157821851	C5orf52	chromosome 5 open reading frame 52	-0.830
157821541	ACSS3	acyl-CoA synthetase short chain family member 3	-0.830

56605720	GADD45B	growth arrest and DNA damage inducible beta	-0.828
61740621	RARRES2	retinoic acid receptor responder 2	-0.826
6980992	GSTT2/GSTT2B	glutathione S-transferase theta 2 (gene/pseudogene)	-0.824
149038682	Srgn	serglycin	-0.823
672063869	MGC116197 (includes others)	similar to RIKEN cDNA 1700001E04	-0.823
564372514	SHBG	sex hormone binding globulin	-0.822
157823295	OLFM4	olfactomedin 4	-0.817
672067229	N/A	N/A	-0.816
148675846	FAM114A2	family with sequence similarity 114 member A2	-0.814
392333201	CFAP70	cilia and flagella associated protein 70	-0.813
564342320	FSIP1	fibrous sheath interacting protein 1	-0.813
149042883	LOC100365365	rCG32328-like	-0.812
58865784	GPR157	G protein-coupled receptor 157	-0.812
564343851	BPIFB1	BPI fold containing family B member 1	-0.807
16758272	CPN1	carboxypeptidase N subunit 1	-0.807
53791211	PHOX2A	paired like homeobox 2a	-0.807
37591183	SLC10A6	solute carrier family 10 member 6	-0.807
300795865	SLC15A5	solute carrier family 15 member 5	-0.807
157817065	KCNK16	potassium two pore domain channel subfamily K member 16	-0.807
880882276	N/A	N/A	-0.807
259089426	AGER	advanced glycosylation end-product specific receptor	-0.804
672044124	N/A	N/A	-0.803
564300659	CGN	cingulin	-0.801
672045595	RIF1	replication timing regulatory factor 1	-0.799
564396113	ZCCHC14	zinc finger CCHC-type containing 14	-0.797
157821153	ECHDC2	enoyl-CoA hydratase domain containing 2	-0.796
58865924	RSPH1	radial spoke head 1 homolog	-0.795
61556945	MOAP1	modulator of apoptosis 1	-0.795
564388219	ARHGAP22	Rho GTPase activating protein 22	-0.795
109480098	SMARCC2	SWI/SNF related, matrix associated, actin dependent regulator of chromatin subfamily c member 2	-0.795
148701441	N/A	N/A	-0.794
8393053	CASR	calcium sensing receptor	-0.794
199561637	PSD4	pleckstrin and Sec7 domain containing 4	-0.794
672015213	N/A	N/A	-0.794

564314685	EIF4G1	eukaryotic translation initiation factor 4 gamma 1	-0.793
157817819	CCDC103	coiled-coil domain containing 103	-0.791
16924012	TNFSF11	TNF superfamily member 11	-0.791
564345556	CROT	carnitine O-octanoyltransferase	-0.790
300798598	MYOF	myoferlin	-0.790
402692079	PFKFB1	6-phosphofructo-2-kinase/fructose-2,6-biphosphatase 1	-0.788
300798165	ZBTB40	zinc finger and BTB domain containing 40	-0.788
18426812	ADA	adenosine deaminase	-0.787
62078891	ZC2HC1C	zinc finger C2HC-type containing 1C	-0.786
16758318	PDK4	pyruvate dehydrogenase kinase 4	-0.783
67846074	EHD2	EH domain containing 2	-0.782
281306773	Htr5b	5-hydroxytryptamine (serotonin) receptor 5B	-0.782
162287322	LSP1	lymphocyte-specific protein 1	-0.781
829944637	N/A	N/A	-0.780
307548437	NYAP2	neuronal tyrosine-phosphorylated phosphoinositide-3-kinase adaptor 2	-0.780
149059246	N/A	N/A	-0.778
74178753	DENND2D	DENN domain containing 2D	-0.778
194474096	Gpihbp1	GPI-anchored HDL-binding protein 1	-0.778
157786718	OTOP2	otopetrin 2	-0.778
157819203	TECTA	tectorin alpha	-0.778
148702528	OTOP3	otopetrin 3	-0.776
157818565	CFAP126	cilia and flagella associated protein 126	-0.775
942523340	CAPRIN2	caprin family member 2	-0.774
392352101	LRCH3	leucine rich repeats and calponin homology domain containing 3	-0.773
157819205	EFHC2	EF-hand domain containing 2	-0.772
55741827	TERT	telomerase reverse transcriptase	-0.771
818015	HBB	hemoglobin subunit beta	-0.770
157819467	C19orf71	chromosome 19 open reading frame 71	-0.769
197386131	Acad10	acyl-CoA dehydrogenase family, member 10	-0.768
564298020	KCTD14	potassium channel tetramerization domain containing 14	-0.767
61097937	VEGFB	vascular endothelial growth factor B	-0.766
197385174	ENO4	enolase family member 4	-0.766
157820387	SLC17A9	solute carrier family 17 member 9	-0.766
56090397	CYB5D2	cytochrome b5 domain containing 2	-0.764
13928758	CTSK	cathepsin K	-0.761

74177759	RPS2	ribosomal protein S2	-0.761
57528269	ABHD14A	abhydrolase domain containing 14A	-0.760
625217421	N/A	N/A	-0.760
148664646	GYPC	glycophorin C (Gerbich blood group)	-0.760
58865632	ARHGAP24	Rho GTPase activating protein 24	-0.758
672023936	N/A	N/A	-0.758
27465577	Cyp4f16/Cyp4f37	cytochrome P450, family 4, subfamily f, polypeptide 16	-0.758
71361637	STRA6	stimulated by retinoic acid 6	-0.757
18158435	TACR2	tachykinin receptor 2	-0.757
13591914	ANPEP	alanyl aminopeptidase, membrane	-0.757
293349343	MYO6	myosin VI	-0.755
16758804	ACACB	acetyl-CoA carboxylase beta	-0.755
66730425	MGC105567	similar to cDNA sequence BC023105	-0.755
589918901	N/A	N/A	-0.755
309319796	COL18A1	collagen type XVIII alpha 1 chain	-0.754
564307792	TECPR2	tectonin beta-propeller repeat containing 2	-0.753
157822593	NEIL2	nei like DNA glycosylase 2	-0.753
189011669	FERMT3	fermitin family member 3	-0.751
319009550	PPM1N	protein phosphatase, Mg2+/Mn2+ dependent 1N (putative)	-0.750
30794230	TNFRSF1B	TNF receptor superfamily member 1B	-0.749
672069802	C1QTNF1	C1q and TNF related 1	-0.749
157821431	GAL3ST1	galactose-3-O-sulfotransferase 1	-0.748
255652942	EFCC1	EF-hand and coiled-coil domain containing 1	-0.747
194473640	FAM109B	family with sequence similarity 109 member B	-0.746
281427214	TRADD	TNFRSF1A associated via death domain	-0.746
149056609	DEDD2	death effector domain containing 2	-0.745
564312886	DNAH2	dynein axonemal heavy chain 2	-0.740
157786850	TUBD1	tubulin delta 1	-0.740
46485412	ABCA7	ATP binding cassette subfamily A member 7	-0.739
13242287	HES5	hes family bHLH transcription factor 5	-0.737
149057856	ADRB3	adrenoceptor beta 3	-0.737
53850642	AKAP3	A-kinase anchoring protein 3	-0.737
12621132	FAT2	FAT atypical cadherin 2	-0.737
149032986	MFSD4B	major facilitator superfamily domain containing 4B	-0.737
585191689	N/A	N/A	-0.737
157822391	OTOG	otogelin	-0.737
13929084	THBD	thrombomodulin	-0.732

56119147	ARRDC3	arrestin domain containing 3	-0.732
672089518	N/A	N/A	-0.728
672022615	N/A	N/A	-0.728
13929156	MYBPH	myosin binding protein H	-0.728
68163370	CARNMT1	carnosine N-methyltransferase 1	-0.724
208973286	RBM46	RNA binding motif protein 46	-0.724
402534539	C2orf40	chromosome 2 open reading frame 40	-0.723
76253906	CASP4	caspase 4	-0.722
16758232	PLCB2	phospholipase C beta 2	-0.721
19173756	ERG	ERG, ETS transcription factor	-0.718
568939712	KCP	kielin/chordin-like protein	-0.718
157821145	DQX1	DEAQ-box RNA dependent ATPase 1	-0.718
148669751	SMNDC1	survival motor neuron domain containing 1	-0.717
568927637	ADAMTSL1	ADAMTS like 1	-0.716
149062459	MS4A2	membrane spanning 4-domains A2	-0.716
58219500	SLC10A7	solute carrier family 10 member 7	-0.716
149047075	Spaca6	sperm acrosome associated 6	-0.715
148704596	L3HYPDH	trans-L-3-hydroxyproline dehydratase	-0.715
27465603	AKR1B10	aldo-keto reductase family 1 member B10	-0.714
6981108	ITGB4	integrin subunit beta 4	-0.712
68341959	CASTOR1	cytosolic arginine sensor for mTORC1 subunit 1	-0.712
148701845	RACK1	receptor for activated C kinase 1	-0.710
672076581	N/A	N/A	-0.707
672080683	Tmem255b	transmembrane protein 255B	-0.707
564378828	TFR2	transferrin receptor 2	-0.707
564400341	N/A	N/A	-0.706
66730475	Tpm2	tropomyosin 2, beta	-0.705
157818511	CCDC190	coiled-coil domain containing 190	-0.705
8393922	PCTP	phosphatidylcholine transfer protein	-0.705
672057367	N/A	N/A	-0.704
54312100	DNAJB13	DnaJ heat shock protein family (Hsp40) member B13	-0.703
62644808	ADAMTSL2	ADAMTS like 2	-0.699
55742713	ECM1	extracellular matrix protein 1	-0.699
157816997	BDH2	3-hydroxybutyrate dehydrogenase 2	-0.698
40018618	CBX7	chromobox 7	-0.698
281332190	APBB1IP	amyloid beta precursor protein binding family B member 1 interacting protein	-0.696
148671621	VIP	vasoactive intestinal peptide	-0.696

157786678	Cisd3	CDGSH iron sulfur domain 3	-0.695
401461786	CP	ceruloplasmin	-0.695
672064415	LOC103693202	uncharacterized LOC103693202	-0.693
293343546	C5orf49	chromosome 5 open reading frame 49	-0.693
564393107	TMEM173	transmembrane protein 173	-0.693
48675865	PDP2	pyruvate dehyrogenase phosphatase catalytic subunit 2	-0.692
113206040	LRRC34	leucine rich repeat containing 34	-0.690
564317714	Ktn1	kinectin 1	-0.687
149066381	DSCC1	DNA replication and sister chromatid cohesion 1	-0.687
157823213	CAPSL	calcyphosine like	-0.687
300794452	PTPRH	protein tyrosine phosphatase, receptor type H	-0.687
803217203	N/A	N/A	-0.686
157821197	C9orf135	chromosome 9 open reading frame 135	-0.683
149053566	N/A	N/A	-0.683
672072376	N/A	N/A	-0.683
77917598	TMCO4	transmembrane and coiled-coil domains 4	-0.682
149047375	CCDC96	coiled-coil domain containing 96	-0.682
46485501	CDH15	cadherin 15	-0.681
672046728	N/A	N/A	-0.681
300797913	PALB2	partner and localizer of BRCA2	-0.679
71043800	STPG1	sperm tail PG-rich repeat containing 1	-0.679
62821825	OPALIN	oligodendrocytic myelin paranodal and inner loop protein	-0.678
38454234	COL27A1	collagen type XXVII alpha 1 chain	-0.677
11993952	SRPX	sushi repeat containing protein, X-linked	-0.677
33286888	GJA1	gap junction protein alpha 1	-0.675
564320335	TMEM241	transmembrane protein 241	-0.675
148706223	ZNRF4	zinc and ring finger 4	-0.675
148691168	N/A	N/A	-0.675
402745263	COL11A1	collagen type XI alpha 1 chain	-0.672
293349725	AMER3	APC membrane recruitment protein 3	-0.672
189491877	MYADML2	myeloid associated differentiation marker like 2	-0.671
149039662	LAMA2	laminin subunit alpha 2	-0.670
568986424	Prrxl1	paired related homeobox protein-like 1	-0.667
74142284	DSE	dermatan sulfate epimerase	-0.667
157818909	Zim1	zinc finger, imprinted 1	-0.665
67514566	POLA2	DNA polymerase alpha 2, accessory subunit	-0.665
58866014	CATSPER2	cation channel sperm associated 2	-0.664

672038314	N/A	N/A	-0.664
672057459	DGKA	diacylglycerol kinase alpha	-0.664
281427186	CHST9	carbohydrate sulfotransferase 9	-0.661
13592057	RPL18	ribosomal protein L18	-0.660
305682588	PDZD7	PDZ domain containing 7	-0.660
672081701	N/A	N/A	-0.660
70912395	C3orf67	chromosome 3 open reading frame 67	-0.659
201861690	TPK1	thiamin pyrophosphokinase 1	-0.657
114145782	MORN5	MORN repeat containing 5	-0.657
158262033	PAH	phenylalanine hydroxylase	-0.655
157824216	RRAS	RAS related	-0.655
514450311	N/A	N/A	-0.655
9845261	LGALS1	galectin 1	-0.655
300794803	SYNPO2	synaptopodin 2	-0.655
164565435	SYNJ2	synaptojanin 2	-0.654
8392864	ADORA2B	adenosine A2b receptor	-0.654
281306821	HEY2	hes related family bHLH transcription factor with YRPW motif 2	-0.653
157823283	Coch	cochlin	-0.653
82617598	SLC5A3	solute carrier family 5 member 3	-0.652
564350366	N/A	N/A	-0.652
149066324	LOC690120	hypothetical protein LOC690120	-0.652
157817426	MISP	mitotic spindle positioning	-0.652
281332078	TTLL2	tubulin tyrosine ligase like 2	-0.652
157822365	LAMC3	laminin subunit gamma 3	-0.651
119226202	CDC42EP1	CDC42 effector protein 1	-0.651
672064676	N/A	N/A	-0.649
47059173	IER3	immediate early response 3	-0.649
157819783	IRF6	interferon regulatory factor 6	-0.648
77020281	CD55	CD55 molecule (Cromer blood group)	-0.648
672039742	TKFC	triokinase and FMN cyclase	-0.646
62079183	CCT6B	chaperonin containing TCP1 subunit 6B	-0.646
68534736	ERAP1	endoplasmic reticulum aminopeptidase 1	-0.646
260593702	Slc26a10	solute carrier family 26, member 10	-0.646
564348231	RPUSD3	RNA pseudouridylate synthase domain containing 3	-0.645
197333844	LLPH	LLP homolog, long-term synaptic facilitation	-0.644
13928928	NAPSA	napsin A aspartic peptidase	-0.644
157816915	ZNF438	zinc finger protein 438	-0.643
564316927	FRYL	FRY like transcription coactivator	-0.643

13591940	DPYD	dihydropyrimidine dehydrogenase	-0.642
13786136	PDGFC	platelet derived growth factor C	-0.642
187469467	SMPD5	sphingomyelin phosphodiesterase 5	-0.642
293340128	MIEF2	mitochondrial elongation factor 2	-0.642
25742776	MC4R	melanocortin 4 receptor	-0.641
148665664	PHLDB2	pleckstrin homology like domain family B member 2	-0.641
158508544	DDR2	discoidin domain receptor tyrosine kinase 2	-0.640
300794353	FANCL	Fanconi anemia complementation group L	-0.639
157786864	PHOSPHO1	phosphoethanolamine/phosphocholine phosphatase	-0.638
392355027	TANGO6	transport and golgi organization 6 homolog	-0.638
149061352	ADAM12	ADAM metallopeptidase domain 12	-0.638
71043760	RRM2	ribonucleotide reductase regulatory subunit M2	-0.637
672060362	ELFN2	extracellular leucine rich repeat and fibronectin type III domain containing 2	-0.636
187282311	ISLR	immunoglobulin superfamily containing leucine rich repeat	-0.635
19424350	GBP2	guanylate binding protein 2	-0.635
58865948	CREB3L2	cAMP responsive element binding protein 3 like 2	-0.634
31542804	FCGR2A	Fc fragment of IgG receptor IIa	-0.633
50657355	TOP1MT	DNA topoisomerase I mitochondrial	-0.632
672076564	DDC	dopa decarboxylase	-0.632
157821105	SLC4A11	solute carrier family 4 member 11	-0.632
34734058	HCK	HCK proto-oncogene, Src family tyrosine kinase	-0.631
880911340	N/A	N/A	-0.630
672053201	N/A	N/A	-0.630
392334060	LAMA3	laminin subunit alpha 3	-0.630
11968122	PLLP	plasmolipin	-0.630
8394221	Rps3a1	ribosomal protein S3A1	-0.630
397529557	C8orf58	chromosome 8 open reading frame 58	-0.629
13591916	ABCC6	ATP binding cassette subfamily C member 6	-0.628
157824208	NTNG1	netrin G1	-0.628
300797715	NDST4	N-deacetylase and N-sulfotransferase 4	-0.627
58865854	SCRN2	secernin 2	-0.627
16758622	IFT172	intraflagellar transport 172	-0.627
149038530	CCDC170	coiled-coil domain containing 170	-0.626

564395215	LOC100909409 (includes others)	RGD1562660	-0.626
672031392	N/A	N/A	-0.626
74203836	PNPLA7	patatin like phospholipase domain containing 7	-0.626
1763306	UNC13C	unc-13 homolog C	-0.626
157818465	P2ry10b	purinergic receptor P2Y, G-protein coupled 10B	-0.624
731457978	N/A	N/A	-0.624
672013308	CCER2	coiled-coil glutamate rich protein 2	-0.621
157817396	MIS18BP1	MIS18 binding protein 1	-0.621
760997729	SYNPO2L	synaptopodin 2 like	-0.621
197927137	WDR63	WD repeat domain 63	-0.621
157819949	ITGA4	integrin subunit alpha 4	-0.620
402744047	SLC25A18	solute carrier family 25 member 18	-0.620
157786914	OGFOD2	2-oxoglutarate and iron dependent oxygenase domain containing 2	-0.618
685536628	N/A	N/A	-0.618
16758284	SLC5A7	solute carrier family 5 member 7	-0.618
672058561	N/A	N/A	-0.618
169234854	SPATC1	spermatogenesis and centriole associated 1	-0.617
166157468	CLMN	calmin	-0.616
62945382	SERTAD3	SERTA domain containing 3	-0.616
478732983	MAP3K5	mitogen-activated protein kinase kinase kinase 5	-0.616
62078563	CD302	CD302 molecule	-0.613
672042306	N/A	N/A	-0.613
672078695	N/A	N/A	-0.612
281427233	WISP3	WNT1 inducible signaling pathway protein 3	-0.611
672020846	N/A	N/A	-0.610
157821551	EBI3	Epstein-Barr virus induced 3	-0.609
655832893	N/A	N/A	-0.608
402478640	HTRA3	HtrA serine peptidase 3	-0.608
149068766	PLEKHB1	pleckstrin homology domain containing B1	-0.608
157819065	ADAMTS15	ADAM metallopeptidase with thrombospondin type 1 motif 15	-0.604
672014740	MAMDC2	MAM domain containing 2	-0.603
401709944	MPP7	membrane palmitoylated protein 7	-0.603
157818181	GPR146	G protein-coupled receptor 146	-0.602
157818491	DUS2	dihydrouridine synthase 2	-0.602
27465529	SLC9A4	solute carrier family 9 member A4	-0.601

17939358	GPR83	G protein-coupled receptor 83	-0.600
29789038	BMP6	bone morphogenetic protein 6	-0.600
56605940	RXFP3	relaxin/insulin like family peptide receptor 3	-0.599
281332166	GPR158	G protein-coupled receptor 158	-0.599
293347888	SRBD1	S1 RNA binding domain 1	-0.598
82654224	IDNK	IDNK, gluconokinase	-0.597
564387543	UGGT2	UDP-glucose glycoprotein glucosyltransferase 2	-0.596
672013014	N/A	N/A	-0.596
157817670	SLC2A10	solute carrier family 2 member 10	-0.595
18677743	FGF11	fibroblast growth factor 11	-0.594
13162324	MEPE	matrix extracellular phosphoglycoprotein	-0.593
6978737	CYP1B1	cytochrome P450 family 1 subfamily B member 1	-0.593
655886694	N/A	N/A	-0.592
354475081	N/A	N/A	-0.591
28461161	LDLR	low density lipoprotein receptor	-0.591
672018103	N/A	N/A	-0.590
108935976	DISC1	disrupted in schizophrenia 1	-0.590
537141843	N/A	N/A	-0.589
21245102	PLPP2	phospholipid phosphatase 2	-0.589
567316103	Ac1576	uncharacterized LOC102552783	-0.587
157822207	HOGA1	4-hydroxy-2-oxoglutarate aldolase 1	-0.587
197927123	LYRM7	LYR motif containing 7	-0.587
589932011	N/A	N/A	-0.586
201860292	CA13	carbonic anhydrase 13	-0.585
149063098	LAT2	linker for activation of T cells family member 2	-0.585
189230091	MIS18A	MIS18 kinetochore protein A	-0.585
300793858	PARP14	poly(ADP-ribose) polymerase family member 14	-0.585
56090245	TCP11	t-complex 11	-0.585
568950242	Pgap2	post-GPI attachment to proteins 2	-0.583
672086986	SLC38A5	solute carrier family 38 member 5	-0.583
83320101	AFG1L	AFG1 like ATPase	-0.583
564394999	CLGN	calmegin	-0.582
83649764	STYXL1	serine/threonine/tyrosine interacting like 1	-0.582
149057816	N/A	N/A	-0.582
148692627	N/A	N/A	-0.581
149051152	N/A	N/A	-0.581
392339456	CKAP2L	cytoskeleton associated protein 2 like	-0.579
470602254	N/A	N/A	-0.578

164519095	SLC9A2	solute carrier family 9 member A2	-0.578
187282394	USP43	ubiquitin specific peptidase 43	-0.577
564323143	Gprasp2	G protein-coupled receptor associated sorting protein 2	-0.576
157820485	SLC9B2	solute carrier family 9 member B2	-0.576
564300485	LOC102551095	uncharacterized LOC102551095	-0.576
564333152	N/A	N/A	-0.576
30027645	GHR	growth hormone receptor	-0.575
291463305	SHISA9	shisa family member 9	-0.574
16758560	WIF1	WNT inhibitory factor 1	-0.573
148690852	FCGRT	Fc fragment of IgG receptor and transporter	-0.573
672028919	N/A	N/A	-0.573
293346999	MBD4	methyl-CpG binding domain 4, DNA glycosylase	-0.573
78486556	C16orf58	chromosome 16 open reading frame 58	-0.572
564366772	MGC116197 (includes others)	similar to RIKEN cDNA 1700001E04	-0.569
55741859	XRCC4	X-ray repair cross complementing 4	-0.569
67846096	MFSD3	major facilitator superfamily domain containing 3	-0.568
157816941	PLXDC1	plexin domain containing 1	-0.567
19173754	TESK2	testis-specific kinase 2	-0.567
564390348	Klhl3	kelch-like family member 3	-0.566
62078799	QRSL1	glutaminyl-tRNA synthase (glutamine-hydrolyzing)-like 1	-0.566
672053541	N/A	N/A	-0.566
157817903	Dcaf12l1	DDB1 and CUL4 associated factor 12-like 1	-0.565
281182643	ALK	ALK receptor tyrosine kinase	-0.565
9845234	ANXA2	annexin A2	-0.565
149048141	PMF1/PMF1-BGLAP	polyamine modulated factor 1	-0.565
564303933	TET3	tet methylcytosine dioxygenase 3	-0.565
62078887	CLEC14A	C-type lectin domain containing 14A	-0.564
148691906	N/A	N/A	-0.564
7549765	HK2	hexokinase 2	-0.563
61889110	OSTF1	osteoclast stimulating factor 1	-0.563
120474989	KRT1	keratin 1	-0.563
672015205	N/A	N/A	-0.563
114145407	CNTNAP5	contactin associated protein like 5	-0.563
57012436	Krt10	keratin 10	-0.562

8393469	S1PR2	sphingosine-1-phosphate receptor 2	-0.561
78486570	ARHGAP4	Rho GTPase activating protein 4	-0.560
672051901	N/A	N/A	-0.559
157821487	ANKRD34B	ankyrin repeat domain 34B	-0.559
569009290	TENM1	teneurin transmembrane protein 1	-0.559
158508553	HAVCR2	hepatitis A virus cellular receptor 2	-0.558
57114344	UHRF1	ubiquitin like with PHD and ring finger domains 1	-0.558
187282036	ZBTB42	zinc finger and BTB domain containing 42	-0.558
197313645	SMTN	smoothelin	-0.558
564372698	N/A	N/A	-0.557
21326477	RIPK3	receptor interacting serine/threonine kinase 3	-0.557
472391630	N/A	N/A	-0.557
75832150	GALNT3	polypeptide N-acetylgalactosaminyltransferase 3	-0.557
564310904	N/A	N/A	-0.556
213385268	Gm10778	predicted gene 10778	-0.554
213513304	ATP10A	ATPase phospholipid transporting 10A (putative)	-0.554
300798350	LRRK1	leucine rich repeat kinase 1	-0.554
42476287	TGM2	transglutaminase 2	-0.553
16758788	PTPN6	protein tyrosine phosphatase, non-receptor type 6	-0.552
199562000	USP40	ubiquitin specific peptidase 40	-0.552
293349510	STAC	SH3 and cysteine rich domain	-0.550
392331598	MPV17L	MPV17 mitochondrial inner membrane protein like	-0.549
392331598	MPV17L	MPV17 mitochondrial inner membrane protein like	-0.549
564378482	MICALL2	MICAL like 2	-0.548
157820141	KLHDC1	kelch domain containing 1	-0.546
672023790	N/A	N/A	-0.545
300795183	SNTG1	syntrophin gamma 1	-0.543
171846640	FBLN1	fibulin 1	-0.543
188497675	CXorf57	chromosome X open reading frame 57	-0.542
815891318	ENTPD1	ectonucleoside triphosphate diphosphohydrolase 1	-0.541
75832132	ESYT1	extended synaptotagmin 1	-0.541
672080024	N/A	N/A	-0.540
672066171	GIN1	gypsy retrotransposon integrase 1	-0.539

6754808	NDP	NDP, norrin cystine knot growth factor	-0.538
9507045	RGS5	regulator of G protein signaling 5	-0.538
157823932	FAM212A	family with sequence similarity 212 member A	-0.538
564334233	CFAP43	cilia and flagella associated protein 43	-0.537
11560101	GCNT1	glucosaminyl (N-acetyl) transferase 1, core 2	-0.537
672015368	MAST4	microtubule associated serine/threonine kinase family member 4	-0.536
568972691	STAT5B	signal transducer and activator of transcription 5B	-0.535
16758390	CLIC5	chloride intracellular channel 5	-0.535
115392004	GPR17	G protein-coupled receptor 17	-0.535
564369844	NEWGENE_1308624	sialidase 4	-0.534
20302097	PIGL	phosphatidylinositol glycan anchor biosynthesis class L	-0.534
28461157	CRYL1	crystallin lambda 1	-0.534
148683700	TMEM98	transmembrane protein 98	-0.533
76096314	DSC2	desmocollin 2	-0.532
254675172	AK7	adenylate kinase 7	-0.531
569012000	KLF8	Kruppel like factor 8	-0.531
300794555	TMC7	transmembrane channel like 7	-0.531
984102375	N/A	N/A	-0.529
672051145	PARP11	poly(ADP-ribose) polymerase family member 11	-0.529
46310239	SIDT1	SID1 transmembrane family member 1	-0.528
564319108	ADGRA2	adhesion G protein-coupled receptor A2	-0.527
157822527	Prorsd1	prolyl-tRNA synthetase domain containing 1	-0.527
157818843	EXTL1	exostosin like glycosyltransferase 1	-0.527
149067143	N/A	N/A	-0.527
635047523	N/A	N/A	-0.526
56090301	NUDT5	nudix hydrolase 5	-0.526
40018594	POPDC2	popeye domain containing 2	-0.525
157820317	STXBP4	syntaxin binding protein 4	-0.524
148747464	SCD	stearoyl-CoA desaturase	-0.524
19424232	CSF2RB	colony stimulating factor 2 receptor beta common subunit	-0.524
148678784	PTHLH	parathyroid hormone like hormone	-0.523
157822879	EFS	embryonal Fyn-associated substrate	-0.523
564350006	PREX2	phosphatidylinositol-3,4,5-trisphosphate dependent Rac exchange factor 2	-0.523

13929182	VAMP8	vesicle associated membrane protein 8	-0.522
672071899	BHLHA15	basic helix-loop-helix family member a15	-0.522
124244050	PPIP5K1	diphosphoinositol pentakisphosphate kinase 1	-0.522
16758130	WNT4	Wnt family member 4	-0.520
56676356	SLC10A4	solute carrier family 10 member 4	-0.520
157822555	RIN3	Ras and Rab interactor 3	-0.520
672063281	PPM1M	protein phosphatase, Mg2+/Mn2+ dependent 1M	-0.519
568966731	HMG20B	high mobility group 20B	-0.518
392340179	RERG	RAS like estrogen regulated growth inhibitor	-0.518
6981180	MAOB	monoamine oxidase B	-0.518
148692356	ARHGEF1	Rho guanine nucleotide exchange factor 1	-0.517
11067395	Tcam1	testicular cell adhesion molecule 1	-0.516
293348472	ZFR2	zinc finger RNA binding protein 2	-0.516
149037033	PRDM5	PR/SET domain 5	-0.516
157823277	DYSF	dysferlin	-0.515
759101041	N/A	N/A	-0.515
61556921	UBXN10	UBX domain protein 10	-0.515
392338379	SLC26A8	solute carrier family 26 member 8	-0.514
237757336	OLIG1	oligodendrocyte transcription factor 1	-0.514
76159291	CAST	calpastatin	-0.514
149047863	LOC690190	hypothetical protein LOC690190	-0.514
72255523	DNALI1	dynein axonemal light intermediate chain 1	-0.513
25282441	DECR2	2,4-dienoyl-CoA reductase 2	-0.513
157820973	RAB32	RAB32, member RAS oncogene family	-0.512
157817684	TRIM14	tripartite motif containing 14	-0.512
149029225	N/A	N/A	-0.511
147900684	TLR7	toll like receptor 7	-0.511
62078773	CCDC81	coiled-coil domain containing 81	-0.511
154937382	MYL9	myosin light chain 9	-0.510
308044487	KIAA0319	KIAA0319	-0.509
293355224	Rps12-ps24	ribosomal protein S12, pseudogene 24	-0.509
47058976	TYROBP	TYRO protein tyrosine kinase binding protein	-0.508
293344916	COL6A1	collagen type VI alpha 1 chain	-0.508
148698795	GPX7	glutathione peroxidase 7	-0.507
312922352	TTF2	transcription termination factor 2	-0.506
17105368	KLF9	Kruppel like factor 9	-0.505
564399060	PIGA	phosphatidylinositol glycan anchor biosynthesis class A	-0.505
62079187	IQCG	IQ motif containing G	-0.504

564355112	EMILIN1	elastin microfibril interfacer 1	-0.503
148692950	CDHR1	cadherin related family member 1	-0.503
157816963	IRF4	interferon regulatory factor 4	-0.503
880876474	N/A	N/A	-0.501
654824082	Fbxl21	F-box and leucine-rich repeat protein 21	-0.500
37693510	Bst2	bone marrow stromal cell antigen 2	-0.500
9506709	GALR2	galanin receptor 2	-0.500
25742772	KCNA2	potassium voltage-gated channel subfamily A member 2	-0.500
209529675	TXLNB	taxilin beta	-0.498
58865396	FIGNL1	fidgetin like 1	-0.497
50233928	TMEM159	transmembrane protein 159	-0.497
470631944	N/A	N/A	-0.497
158138532	ATP7B	ATPase copper transporting beta	-0.497
281371499	COL5A2	collagen type V alpha 2 chain	-0.495
73487332	C1orf115	chromosome 1 open reading frame 115	-0.495
16758444	ST14	suppression of tumorigenicity 14	-0.495
157822847	PSMB11	proteasome subunit beta 11	-0.495
157817292	TPCN2	two pore segment channel 2	-0.495
564330609	SYT17	synaptotagmin 17	-0.494
84662732	DNASE1L1	deoxyribonuclease 1 like 1	-0.494
62078635	CCDC153	coiled-coil domain containing 153	-0.494
157822759	PARP2	poly(ADP-ribose) polymerase 2	-0.493
41386749	PCLAF	PCNA clamp associated factor	-0.492
399220341	SLC2A13	solute carrier family 2 member 13	-0.492
157819347	CDC6	cell division cycle 6	-0.492
145207953	PLAU	plasminogen activator, urokinase	-0.491
149045505	TTPA	alpha tocopherol transfer protein	-0.491
197385188	CIART	circadian associated repressor of transcription	-0.491
913518709	N/A	N/A	-0.491
545208112	N/A	N/A	-0.491
51491896	SPATA18	spermatogenesis associated 18	-0.490
564301698	LY75	lymphocyte antigen 75	-0.489
40018602	FGFRL1	fibroblast growth factor receptor like 1	-0.489
12738847	MERTK	MER proto-oncogene, tyrosine kinase	-0.488
58219539	ENG	endoglin	-0.488
672080825	N/A	N/A	-0.488
558611343	MCM3	minichromosome maintenance complex component 3	-0.488
157817033	TJAP1	tight junction associated protein 1	-0.487

6978867	GABRB1	gamma-aminobutyric acid type A receptor beta1 subunit	-0.487
27545428	MAPK15	mitogen-activated protein kinase 15	-0.487
672084625	LOC100909409 (includes others)	RGD1562660	-0.487
7106349	LYNX1	Ly6/neurotoxin 1	-0.487
112350	N/A	N/A	-0.486
293350806	ZRSR2	zinc finger CCCH-type, RNA binding motif and serine/arginine rich 2	-0.485
157821433	CSF3R	colony stimulating factor 3 receptor	-0.485
157824113	GPR84	G protein-coupled receptor 84	-0.485
77020250	PCSK9	proprotein convertase subtilisin/kexin type 9	-0.485
149058686	PIGR	polymeric immunoglobulin receptor	-0.485
149034989	RADIL	Rap associating with DIL domain	-0.485
281427229	COL6A2	collagen type VI alpha 2 chain	-0.485
157786894	PYCR1	pyrroline-5-carboxylate reductase 1	-0.485
13786160	SLC22A8	solute carrier family 22 member 8	-0.485
209954806	PIGN	phosphatidylinositol glycan anchor biosynthesis class N	-0.485
564399546	STARD8	StAR related lipid transfer domain containing 8	-0.484
157951643	ACTN2	actinin alpha 2	-0.484
625213993	N/A	N/A	-0.484
58865450	BAG3	BCL2 associated athanogene 3	-0.484
672066409	N/A	N/A	-0.483
164607119	SUMF2	sulfatase modifying factor 2	-0.483
392333084	CC2D2A	coiled-coil and C2 domain containing 2A	-0.483
395759219	AQP4	aquaporin 4	-0.482
157786612	B9D1	B9 domain containing 1	-0.482
109497496	MMAB	methylmalonic aciduria (cobalamin deficiency) cblB type	-0.482
13591971	HNMT	histamine N-methyltransferase	-0.481
16758322	SYNGR2	synaptogyrin 2	-0.481
568968462	REEP6	receptor accessory protein 6	-0.481
537241732	N/A	N/A	-0.480
761631363	EPGN	epithelial mitogen	-0.479
56090459	CNDP1	carnosine dipeptidase 1	-0.479
293340174	DNAH9	dynein axonemal heavy chain 9	-0.479
157073947	C1orf74	chromosome 1 open reading frame 74	-0.478
6978505	ANXA5	annexin A5	-0.478
564306247	PHACTR4	phosphatase and actin regulator 4	-0.477

189011606	NCEH1	neutral cholesterol ester hydrolase 1	-0.476
62078713	ZNF385D	zinc finger protein 385D	-0.476
57528252	QPRT	quinolinate phosphoribosyltransferase	-0.475
392332443	PRKDC	protein kinase, DNA-activated, catalytic polypeptide	-0.475
16923978	SLC26A2	solute carrier family 26 member 2	-0.474
916043983	Baiap3	BAI1-associated protein 3	-0.474
202070751	RFTN1	raftlin, lipid raft linker 1	-0.474
77917594	ZFYVE19	zinc finger FYVE-type containing 19	-0.473
148695091	BBS5	Bardet-Biedl syndrome 5	-0.473
225007623	TCFL5	transcription factor like 5	-0.472
58865984	TRAF3IP1	TRAF3 interacting protein 1	-0.471
307078146	UACA	uveal autoantigen with coiled-coil domains and ankyrin repeats	-0.470
149032914	MOXD1	monooxygenase DBH like 1	-0.469
312922379	TNN	tenascin N	-0.469
157816939	WASHC3	WASH complex subunit 3	-0.469
672034240	N/A	N/A	-0.469
114145710	SEC61G	Sec61 translocon gamma subunit	-0.469
157823399	COG4	component of oligomeric golgi complex 4	-0.468
589269168	WDR34	WD repeat domain 34	-0.467
19924069	SPON2	spondin 2	-0.467
219281893	ZNF583	zinc finger protein 583	-0.467
75905809	AKAP12	A-kinase anchoring protein 12	-0.466
41056215	XRCC5	X-ray repair cross complementing 5	-0.466
157822891	ADCK2	aarF domain containing kinase 2	-0.465
564380929	KCNT2	potassium sodium-activated channel subfamily T member 2	-0.465
71043706	MUS81	MUS81 structure-specific endonuclease subunit	-0.465
672044191	TBCK	TBC1 domain containing kinase	-0.465
672055145	N/A	N/A	-0.465
58865618	PAPOLB	poly(A) polymerase beta	-0.464
148356229	CCND1	cyclin D1	-0.464
149031202	Saysd1	SAYSVFN motif domain containing 1	-0.464
564375796	N/A	N/A	-0.463
589965307	N/A	N/A	-0.463
56090632	ATP5S	ATP synthase, H+ transporting, mitochondrial Fo complex subunit s (factor B)	-0.463
212549544	C15orf39	chromosome 15 open reading frame 39	-0.463
56605808	CENPN	centromere protein N	-0.462

149053720	CA4	carbonic anhydrase 4	-0.461
148709823	PCGF5	polycomb group ring finger 5	-0.460
148710197	Smim10l2a	small integral membrane protein 10 like 2A	-0.459
148669431	DNAJC27	DnaJ heat shock protein family (Hsp40) member C27	-0.459
672034901	DNAAF3	dynein axonemal assembly factor 3	-0.459
157823151	DLEU7	deleted in lymphocytic leukemia, 7	-0.459
68163403	SLC46A3	solute carrier family 46 member 3	-0.458
9506953	PCOLCE	procollagen C-endopeptidase enhancer	-0.458
672084224	CCDC113	coiled-coil domain containing 113	-0.458
219275548	DUSP19	dual specificity phosphatase 19	-0.458
194440693	Maml2	mastermind like transcriptional coactivator 2	-0.457
310703673	GRIN3A	glutamate ionotropic receptor NMDA type subunit 3A	-0.457
16758600	RGS14	regulator of G protein signaling 14	-0.457
148699893	COL6A1	collagen type VI alpha 1 chain	-0.457
56090411	POLE3	DNA polymerase epsilon 3, accessory subunit	-0.456
157821107	MYO1F	myosin IF	-0.456
121722562	CA9	carbonic anhydrase 9	-0.455
564346692	GIMAP8	GTPase, IMAP family member 8	-0.455
149067372	MTERF2	mitochondrial transcription termination factor 2	-0.455
157786618	RANGRF	RAN guanine nucleotide release factor	-0.455
195976802	DNLZ	DNL-type zinc finger	-0.455
67078530	CHAF1B	chromatin assembly factor 1 subunit B	-0.454
157817743	CDH5	cadherin 5	-0.454
149028405	EBP	emopamil binding protein (sterol isomerase)	-0.454
13562118	LRP2	LDL receptor related protein 2	-0.454
149048674	PEX5L	peroxisomal biogenesis factor 5 like	-0.453
9437326	SLC4A4	solute carrier family 4 member 4	-0.453
13027400	GUCY1A2	guanylate cyclase 1 soluble subunit alpha 2	-0.453
157819513	ABCA4	ATP binding cassette subfamily A member 4	-0.453
167555114	CCDC17	coiled-coil domain containing 17	-0.453
157820241	Marveld1	MARVEL domain containing 1	-0.452
62945352	C4orf19	chromosome 4 open reading frame 19	-0.452
392341425	PTPRB	protein tyrosine phosphatase, receptor type B	-0.452
768711606	THBS3	thrombospondin 3	-0.452
158138494	PTPRC	protein tyrosine phosphatase, receptor type C	-0.451
157817911	C21orf62	chromosome 21 open reading frame 62	-0.451
725595815	N/A	N/A	-0.451

33414515	PXK	PX domain containing serine/threonine kinase like	-0.450
58865380	STAT2	signal transducer and activator of transcription 2	-0.450
568953371	4932443I19Rik	RIKEN cDNA 4932443I19 gene	-0.450
40254754	OCLN	occludin	-0.450
293346302	FBLN7	fibulin 7	-0.448
635015168	N/A	N/A	-0.448
564349878	KIAA1551	KIAA1551	-0.447
8393807	MYH7	myosin heavy chain 7	-0.447
149015786	N/A	N/A	-0.446
149026322	PTGER3	prostaglandin E receptor 3	-0.445
201066407	EAPP	E2F associated phosphoprotein	-0.444
77993368	ACSF2	acyl-CoA synthetase family member 2	-0.444
51854227	GSN	gelsolin	-0.444
157073937	PARP9	poly(ADP-ribose) polymerase family member 9	-0.443
48428501	SYNPO	synaptopodin	-0.443
392331978	CDR2L	cerebellar degeneration related protein 2 like	-0.442
157786756	CDC45	cell division cycle 45	-0.442
27436863	HACL1	2-hydroxyacyl-CoA lyase 1	-0.441
16758186	SLCO1C1	solute carrier organic anion transporter family member 1C1	-0.441
6978629	CD38	CD38 molecule	-0.440
198386351	TBC1D31	TBC1 domain family member 31	-0.439
62078835	TTLL9	tubulin tyrosine ligase like 9	-0.439
149058661	RAB7B	RAB7B, member RAS oncogene family	-0.439
77993356	CDCA7L	cell division cycle associated 7 like	-0.439
12831205	EPAS1	endothelial PAS domain protein 1	-0.438
71361639	GLI4	GLI family zinc finger 4	-0.437
157823657	SMAD6	SMAD family member 6	-0.437
149022245	SCRN3	secernin 3	-0.437
293344128	ADAMTS17	ADAM metallopeptidase with thrombospondin type 1 motif 17	-0.437
68534547	NUDT18	nudix hydrolase 18	-0.437
149028840	N/A	N/A	-0.436
149017515	ALG12	ALG12, alpha-1,6-mannosyltransferase	-0.436
123782692	Cntnap5b	contactin associated protein-like 5B	-0.436
8393057	SERPINH1	serpin family H member 1	-0.436
148686921	SLC24A4	solute carrier family 24 member 4	-0.435
755499410	CCM2L	CCM2 like scaffolding protein	-0.435

68341937	ZNF365	zinc finger protein 365	-0.435
201066363	LOXL2	lysyl oxidase like 2	-0.435
564332022	N/A	N/A	-0.435
404312655	SDR42E1	short chain dehydrogenase/reductase family 42E, member 1	-0.435
564394961	N/A	N/A	-0.434
469663646	NDUFA13	NADH:ubiquinone oxidoreductase subunit A13	-0.433
172045714	MIIP	migration and invasion inhibitory protein	-0.433
61889119	TNFSF12	TNF superfamily member 12	-0.432
210031518	MOGAT2	monoacylglycerol O-acyltransferase 2	-0.432
56605988	FANK1	fibronectin type III and ankyrin repeat domains 1	-0.431
157822677	LGI3	leucine rich repeat LGI family member 3	-0.431
11560087	PYGL	glycogen phosphorylase L	-0.431
537191633	N/A	N/A	-0.431
148673911	Gm21596/Hmgb1	high mobility group box 1	-0.430
110347493	PCDHA9	protocadherin alpha 9	-0.429
157787135	DUSP10	dual specificity phosphatase 10	-0.428
187937018	ITPRIPL2	ITPRIP like 2	-0.428
564400410	AMOT	angiomotin	-0.428
157822319	EVC2	EvC ciliary complex subunit 2	-0.428
157786874	Fmnl1	formin-like 1	-0.427
403225005	Col6a4	collagen, type VI, alpha 4	-0.426
537170289	N/A	N/A	-0.426
13928796	PXMP2	peroxisomal membrane protein 2	-0.426
672089660	ZNF280B	zinc finger protein 280B	-0.426
149038013	SLC9A5	solute carrier family 9 member A5	-0.426
157821557	CD248	CD248 molecule	-0.426
157823913	ARHGAP10	Rho GTPase activating protein 10	-0.425
38454282	ETFBKMT	electron transfer flavoprotein beta subunit lysine methyltransferase	-0.424
157823259	TMEM229A	transmembrane protein 229A	-0.424
76443683	LOC100912042/Surf2	surfeit 2	-0.423
149061527	PGGHG	protein-glucosylgalactosylhydroxylysine glucosidase	-0.423
300796997	ARHGAP28	Rho GTPase activating protein 28	-0.423
672023090	N/A	N/A	-0.423
31745146	TBX3	T-box 3	-0.423
1346731	HAPLN1	hyaluronan and proteoglycan link protein 1	-0.423

300797330	PTPRU	protein tyrosine phosphatase, receptor type U	-0.422
210032365	HSP90B1	heat shock protein 90 beta family member 1	-0.421
56090564	GALM	galactose mutarotase	-0.420
672032219	REPS2	RALBP1 associated Eps domain containing 2	-0.420
62078447	HBA1/HBA2	hemoglobin subunit alpha 2	-0.420
293344558	PCNX3	pecanex homolog 3	-0.420
58743349	FAM89A	family with sequence similarity 89 member A	-0.419
157823313	KANK3	KN motif and ankyrin repeat domains 3	-0.419
564352140	PODN	podocan	-0.419
149037644	N/A	N/A	-0.418
157823279	CGNL1	cingulin like 1	-0.418
149057830	Hgsnat	heparan-alpha-glucosaminide N-acetyltransferase	-0.418
74218228	HNRNPC	heterogeneous nuclear ribonucleoprotein C (C1/C2)	-0.418
672077137	N/A	N/A	-0.417
564329859	COA4	cytochrome c oxidase assembly factor 4 homolog	-0.417
149035030	MAFK	MAF bZIP transcription factor K	-0.417
83642834	NAGK	N-acetylglucosamine kinase	-0.416
13994179	SLC24A2	solute carrier family 24 member 2	-0.416
564342737	DTWD1	DTW domain containing 1	-0.416
62078539	Pagr1	Paxip1-associated glutamate-rich protein 1	-0.415
537268521	N/A	N/A	-0.415
349585066	C10orf90	chromosome 10 open reading frame 90	-0.415
76563944	CLCF1	cardiotrophin like cytokine factor 1	-0.415
748983333	PATJ	PATJ, crumbs cell polarity complex component	-0.415
6981562	SLC9A3	solute carrier family 9 member A3	-0.415
149054795	RGD1309310	similar to mKIAA0195 protein	-0.415
564367529	ENPP4	ectonucleotide pyrophosphatase/phosphodiesterase 4	-0.414
62945312	CXCL16	C-X-C motif chemokine ligand 16	-0.414
568916013	N/A	N/A	-0.414
194473652	TTC38	tetratricopeptide repeat domain 38	-0.413
157819765	OGDHL	oxoglutarate dehydrogenase like	-0.413
50811823	NENF	neudesin neurotrophic factor	-0.413
68342019	LRRC17	leucine rich repeat containing 17	-0.413
672041704	NIPBL	NIPBL, cohesin loading factor	-0.412
672041704	NIPBL	NIPBL, cohesin loading factor	-0.412
187469604	EPS8L2	EPS8 like 2	-0.412

672085293	FANCA	Fanconi anemia complementation group A	-0.412
312596884	SLC31A2	solute carrier family 31 member 2	-0.411
73746573	TGFB1I1	transforming growth factor beta 1 induced transcript 1	-0.411
9506405	ARPC1B	actin related protein 2/3 complex subunit 1B	-0.410
71043794	CEP41	centrosomal protein 41	-0.410
157822913	LHFPL2	LHFPL tetraspan subfamily member 2	-0.410
13591949	GATM	glycine amidinotransferase	-0.409
23463307	RIOX2	ribosomal oxygenase 2	-0.409
672061813	ACSBG1	acyl-CoA synthetase bubblegum family member 1	-0.409
78126167	SLC1A2	solute carrier family 1 member 2	-0.408
672023055	TLN2	talin 2	-0.408
157786690	PRKCA	protein kinase C alpha	-0.408
148697062	TMEM255A	transmembrane protein 255A	-0.408
109480433	GNPTAB	N-acetylglucosamine-1-phosphate transferase alpha and beta subunits	-0.407
564351356	PAPPA	pappalysin 1	-0.407
157819227	PRPF31	pre-mRNA processing factor 31	-0.407
58865490	LCMT2	leucine carboxyl methyltransferase 2	-0.407
198278450	CPT1C	carnitine palmitoyltransferase 1C	-0.406
46402488	NOS3	nitric oxide synthase 3	-0.405
157821089	PEX10	peroxisomal biogenesis factor 10	-0.405
392353178	SEL1L3	SEL1L family member 3	-0.405
7949020	CDK2	cyclin dependent kinase 2	-0.405
880939564	N/A	N/A	-0.405
187281569	CFAP52	cilia and flagella associated protein 52	-0.405
157821021	ZC3H6	zinc finger CCCH-type containing 6	-0.405
13162347	FDXR	ferredoxin reductase	-0.405
358030320	DMTN	dematin actin binding protein	-0.404
672070295	BAHCC1	BAH domain and coiled-coil containing 1	-0.404
118763791	REXO5	RNA exonuclease 5	-0.404
6981664	TNFRSF1A	TNF receptor superfamily member 1A	-0.403
149049048	RECQL	RecQ like helicase	-0.403
127140886	EML6	echinoderm microtubule associated protein like 6	-0.403
195973006	EGFLAM	EGF like, fibronectin type III and laminin G domains	-0.403
157786694	CAVIN1	caveolae associated protein 1	-0.403
62079139	C11orf54	chromosome 11 open reading frame 54	-0.403

122065191	ABAT	4-aminobutyrate aminotransferase	-0.401
58865466	SLC37A1	solute carrier family 37 member 1	-0.401
61889088	CYP2J2	cytochrome P450 family 2 subfamily J member 2	-0.401
188536090	FAM241B	family with sequence similarity 241 member B	-0.400
149024348	RAP1GAP	RAP1 GTPase activating protein	-0.400
157819163	SYPL2	synaptophysin like 2	-0.400
148701235	TIGD3	tigger transposable element derived 3	-0.400
77695926	STAT1	signal transducer and activator of transcription 1	-0.399
9507041	RESP18	regulated endocrine specific protein 18	-0.399
28212252	TPSG1	tryptase gamma 1	-0.399
56605714	NDUFAF7	NADH:ubiquinone oxidoreductase complex assembly factor 7	-0.399
51571903	C6orf47	chromosome 6 open reading frame 47	-0.398
149041576	REXO2	RNA exonuclease 2	-0.398
253683447	ETV1	ETS variant 1	-0.398
62078719	HAUS4	HAUS augmin like complex subunit 4	-0.397
148666792	ARHGAP25	Rho GTPase activating protein 25	-0.396
312836782	MRPS27	mitochondrial ribosomal protein S27	-0.395
13591981	LSS	lanosterol synthase	-0.394
28972652	SLC12A5	solute carrier family 12 member 5	-0.394
755494737	N/A	N/A	-0.394
157819229	RPA3	replication protein A3	-0.393
78187977	TCF19	transcription factor 19	-0.393
564371892	N/A	N/A	-0.393
817259544	N/A	N/A	-0.392
77917572	LIPA	lipase A, lysosomal acid type	-0.392
11177892	KCNT1	potassium sodium-activated channel subfamily T member 1	-0.392
56605634	EMC9	ER membrane protein complex subunit 9	-0.392
149052857	KCNJ12	potassium voltage-gated channel subfamily J member 12	-0.392
57528326	MTFMT	mitochondrial methionyl-tRNA formyltransferase	-0.391
157820327	THSD1	thrombospondin type 1 domain containing 1	-0.391
110347559	PCDHA13	protocadherin alpha 13	-0.391
77628031	SP110	SP110 nuclear body protein	-0.391
564342244	NUTM1	NUT midline carcinoma family member 1	-0.390

8394209	Rpl29 (includes others)	ribosomal protein L29	-0.389
157824150	PTPN22	protein tyrosine phosphatase, non-receptor type 22	-0.389
187937124	TMEM126B	transmembrane protein 126B	-0.389
124286858	B230217C12Rik	RIKEN cDNA B230217C12 gene	-0.389
58865650	LIAS	lipoic acid synthetase	-0.388
564375702	N/A	N/A	-0.388
164663795	KCNB2	potassium voltage-gated channel subfamily B member 2	-0.388
51948488	SIRT5	sirtuin 5	-0.388
296040479	TXNRD3	thioredoxin reductase 3	-0.387
157823193	LOXL3	lysyl oxidase like 3	-0.387
149046383	Lman2l	lectin, mannose-binding 2-like	-0.387
16758268	TEKT1	tektin 1	-0.386
260271475	CCHCR1	coiled-coil alpha-helical rod protein 1	-0.386
672080705	TMCO3	transmembrane and coiled-coil domains 3	-0.386
120586985	APEX2	apurinic/apyrimidinic endodeoxyribonuclease 2	-0.385
166999225	GRM1	glutamate metabotropic receptor 1	-0.385
71043764	C20orf27	chromosome 20 open reading frame 27	-0.384
158081747	PDGFB	platelet derived growth factor subunit B	-0.384
569000267	MDC1	mediator of DNA damage checkpoint 1	-0.384
157786698	RDM1	RAD52 motif containing 1	-0.383
157820337	GGCT	gamma-glutamylcyclotransferase	-0.383
6981210	MME	membrane metalloendopeptidase	-0.382
149057193	N/A	N/A	-0.382
149034469	GNG7	G protein subunit gamma 7	-0.381
148690851	RCN3	reticulocalbin 3	-0.381
564305413	E130308A19Rik	RIKEN cDNA E130308A19 gene	-0.381
149036529	DGUOK	deoxyguanosine kinase	-0.381
19924041	Cyp2d22	cytochrome P450, family 2, subfamily d, polypeptide 22	-0.381
197387452	UVSSA	UV stimulated scaffold protein A	-0.380
114052238	FIG4	FIG4 phosphoinositide 5-phosphatase	-0.380
40786487	GPR108	G protein-coupled receptor 108	-0.379
67078420	PRIM2	DNA primase subunit 2	-0.379
71043890	SMPDL3B	sphingomyelin phosphodiesterase acid like 3B	-0.379
157822957	GPATCH11	G-patch domain containing 11	-0.378
672083001	N/A	N/A	-0.378
18426846	DCBLD2	discoidin, CUB and LCCL domain containing 2	-0.378

672063869	MGC116197 (includes others)	similar to RIKEN cDNA 1700001E04	-0.378
157823299	CSGALNACT1	chondroitin sulfate N-acetylgalactosaminyltransferase 1	-0.378
564370911	CHTF18	chromosome transmission fidelity factor 18	-0.378
672026317	N/A	N/A	-0.377
157817249	CDC14B	cell division cycle 14B	-0.377
209447117	NAA10	N(alpha)-acetyltransferase 10, NatA catalytic subunit	-0.376
568974348	KIF19	kinesin family member 19	-0.376
149058556	LGR6	leucine rich repeat containing G protein-coupled receptor 6	-0.376
672037039	TTC23	tetratricopeptide repeat domain 23	-0.375
205235	Slc6a7	solute carrier family 6 member 7	-0.375
157819009	PHF19	PHD finger protein 19	-0.375
537252133	N/A	N/A	-0.375
162287337	APOE	apolipoprotein E	-0.375
25282445	ENTPD2	ectonucleoside triphosphate diphosphohydrolase 2	-0.375
157822743	KIF20A	kinesin family member 20A	-0.374
219282679	ZNF43	zinc finger protein 43	-0.374
672083553	SLC14A1	solute carrier family 14 member 1 (Kidd blood group)	-0.373
189011677	GLB1L	galactosidase beta 1 like	-0.373
157823373	TRHDE	thyrotropin releasing hormone degrading enzyme	-0.373
149042939	WFDC2	WAP four-disulfide core domain 2	-0.373
16758778	EFNA5	ephrin A5	-0.372
929981595	NPHP1	nephrocystin 1	-0.372
157824002	ATG10	autophagy related 10	-0.372
158631254	MSH4	mutS homolog 4	-0.372
83320092	PRTG	protogenin	-0.372
154090999	NOXA1	NADPH oxidase activator 1	-0.372
257900470	B3GALT4	beta-1,3-galactosyltransferase 4	-0.372
564299653	FAM169A	family with sequence similarity 169 member A	-0.372
113061	CHRNA3	cholinergic receptor nicotinic alpha 3 subunit	-0.372
672055092	N/A	N/A	-0.372
672076077	N/A	N/A	-0.371
8393919	LOC100911216/Pcsk1	proprotein convertase subtilisin/kexin type 1	-0.371

958805663	N/A	N/A	-0.371
672087893	Dmrtc1b	DMRT-like family C1b	-0.371
392337475	SIX5	SIX homeobox 5	-0.371
987938912	N/A	N/A	-0.370
198386343	TRPS1	transcriptional repressor GATA binding 1	-0.370
672039093	N/A	N/A	-0.370
8393861	HPCAL4	hippocalcin like 4	-0.370
53850644	FAM151A	family with sequence similarity 151 member A	-0.369
568970276	SH3PXD2B	SH3 and PX domains 2B	-0.369
568970276	SH3PXD2B	SH3 and PX domains 2B	-0.369
157820213	PERP	PERP, TP53 apoptosis effector	-0.369
157819959	PCDHB2	protocadherin beta 2	-0.369
158303308	PCCA	propionyl-CoA carboxylase alpha subunit	-0.369
884874427	N/A	N/A	-0.368
157819753	RCN1	reticulocalbin 1	-0.368
9247217	MSX1	msh homeobox 1	-0.368
564343748	CDK5RAP1	CDK5 regulatory subunit associated protein 1	-0.368
148677309	CMC1	C-X9-C motif containing 1	-0.367
53734563	ACCS	1-aminocyclopropane-1-carboxylate synthase homolog (inactive)	-0.367
157824128	SOX17	SRY-box 17	-0.367
675781130	N/A	N/A	-0.367
672028474	CDH24	cadherin 24	-0.367
54019428	PCDHA5	protocadherin alpha 5	-0.366
157822461	C20orf194	chromosome 20 open reading frame 194	-0.366
26024221	PRSS12	protease, serine 12	-0.366
697012013	N/A	N/A	-0.366
564357619	ITGB8	integrin subunit beta 8	-0.366
189011681	C17orf49	chromosome 17 open reading frame 49	-0.366
158534064	RET	ret proto-oncogene	-0.365
564365550	GMPPB	GDP-mannose pyrophosphorylase B	-0.365
213512607	CLYBL	citrate lyase beta like	-0.365
820994385	N/A	N/A	-0.365
157818983	SIRT7	sirtuin 7	-0.364
149054665	ABCA9	ATP binding cassette subfamily A member 9	-0.364
157817598	INVS	inversin	-0.364
66730351	C11orf16	chromosome 11 open reading frame 16	-0.364
294979146	TCTN1	tectonic family member 1	-0.363
672057384	N/A	N/A	-0.363
6978761	DGKG	diacylglycerol kinase gamma	-0.362

219278723	ZNF23	zinc finger protein 23	-0.362
16758712	PDIA4	protein disulfide isomerase family A member 4	-0.362
281604225	PUS7	pseudouridylate synthase 7 (putative)	-0.362
398650618	MMP11	matrix metallopeptidase 11	-0.361
157820695	MRPL57	mitochondrial ribosomal protein L57	-0.360
61556907	GALT	galactose-1-phosphate uridylyltransferase	-0.360
55741549	MRPL13	mitochondrial ribosomal protein L13	-0.360
157820839	Dnajb3	DnaJ heat shock protein family (Hsp40) member B3	-0.360
29293811	SERPINF1	serpin family F member 1	-0.359
296470851	PABPC1L2A	poly(A) binding protein cytoplasmic 1 like 2A	-0.359
392337574	N/A	N/A	-0.358
40786491	CYP20A1	cytochrome P450 family 20 subfamily A member 1	-0.358
58865802	SPAG1	sperm associated antigen 1	-0.357
149020543	C19orf66	chromosome 19 open reading frame 66	-0.357
25453410	CACNA1B	calcium voltage-gated channel subunit alpha1 B	-0.357
149066868	MDM1	Mdm1 nuclear protein	-0.357
672072376	N/A	N/A	-0.357
672086023	HSF2BP	heat shock transcription factor 2 binding protein	-0.357
672051732	N/A	N/A	-0.356
215272398	HIP1	huntingtin interacting protein 1	-0.356
384368019	Snhg11	small nucleolar RNA host gene 11	-0.356
149065269	N/A	N/A	-0.355
157822187	WWOX	WW domain containing oxidoreductase	-0.355
310772247	VWA3A	von Willebrand factor A domain containing 3A	-0.355
78042613	NICN1	nicolin 1	-0.354
348041395	DLGAP2	DLG associated protein 2	-0.354
564361507	CRELD2	cysteine rich with EGF like domains 2	-0.354
568918806	TP53RK	TP53 regulating kinase	-0.354
672052705	FRRS1L	ferric chelate reductase 1 like	-0.354
11693172	CALR	calreticulin	-0.354
568945717	HSPB6	heat shock protein family B (small) member 6	-0.352
507532705	N/A	N/A	-0.352
594191048	C19orf54	chromosome 19 open reading frame 54	-0.352
564331077	HIRIP3	HIRA interacting protein 3	-0.352
31077108	GLDN	gliomedin	-0.351
61557118	PCGF6	polycomb group ring finger 6	-0.351
157823944	SUSD2	sushi domain containing 2	-0.351

16758024	SYT9	synaptotagmin 9	-0.351
149060100	AIFM1	apoptosis inducing factor mitochondria associated 1	-0.351
209862829	SEMA3E	semaphorin 3E	-0.350
149039154	COQ4	coenzyme Q4	-0.350
224420	N/A	N/A	-0.350
939319594	CPNE7	copine 7	-0.350
26006275	ABCB9	ATP binding cassette subfamily B member 9	-0.350
149023022	Oip5	Opa interacting protein 5	-0.350
56605656	DONSON	downstream neighbor of SON	-0.350
149053793	TSPOAP1	TSPO associated protein 1	-0.349
16758418	C5AR1	complement C5a receptor 1	-0.349
148747194	SLC16A7	solute carrier family 16 member 7	-0.349
451172073	CHRM3	cholinergic receptor muscarinic 3	-0.349
158749582	MST1R	macrophage stimulating 1 receptor	-0.349
148702120	ETV4	ETS variant 4	-0.348
149058577	Ppfia4	protein tyrosine phosphatase, receptor type, f polypeptide (PTPRF), interacting protein (liprin), alpha 4	-0.348
149048968	ITPR2	inositol 1,4,5-trisphosphate receptor type 2	-0.348
31088854	HPS6	HPS6, biogenesis of lysosomal organelles complex 2 subunit 3	-0.347
564329392	FLNA	filamin A	-0.347
984104888	N/A	N/A	-0.347
62078697	NPL	N-acetylneuraminate pyruvate lyase	-0.347
374253863	CPNE2	copine 2	-0.347
564312446	LMF1	lipase maturation factor 1	-0.347
537137169	N/A	N/A	-0.346
2735334	PDPN	podoplanin	-0.345
55741540	KATNAL1	katanin catalytic subunit A1 like 1	-0.345
568956384	ADAMTS18	ADAM metallopeptidase with thrombospondin type 1 motif 18	-0.345
564332376	RASGRP2	RAS guanyl releasing protein 2	-0.345
157819433	MAP6D1	MAP6 domain containing 1	-0.344
955478861	N/A	N/A	-0.344
672015093	VWA2	von Willebrand factor A domain containing 2	-0.344
56119120	SNF8	SNF8, ESCRT-II complex subunit	-0.343
62078847	TSEN2	tRNA splicing endonuclease subunit 2	-0.343
16758580	AURKB	aurora kinase B	-0.343
6981018	HCRTR1	hypocretin receptor 1	-0.343

157817498	GLDC	glycine decarboxylase	-0.343
50511312	POFUT1	protein O-fucosyltransferase 1	-0.343
14269568	LXN	latexin	-0.343
300795140	TAF1	TATA-box binding protein associated factor 1	-0.342
11024664	LTBP1	latent transforming growth factor beta binding protein 1	-0.342
81158091	PCDHGA9	protocadherin gamma subfamily A, 9	-0.342
77157795	MAL2	mal, T cell differentiation protein 2 (gene/pseudogene)	-0.342
58219522	SLC25A29	solute carrier family 25 member 29	-0.342
564374356	ADAM11	ADAM metallopeptidase domain 11	-0.341
815891312	CACNA1G	calcium voltage-gated channel subunit alpha1 G	-0.340
160333093	TPRG1L	tumor protein p63 regulated 1 like	-0.340
56605758	THAP1	THAP domain containing 1	-0.340
672052684	N/A	N/A	-0.340
62078469	RSPH10B/RSPH10B2	radial spoke head 10 homolog B	-0.340
149027325	DNAJC21	DnaJ heat shock protein family (Hsp40) member C21	-0.339
157820807	GCDH	glutaryl-CoA dehydrogenase	-0.339
11693162	INSIG1	insulin induced gene 1	-0.339
56605710	LTBR	lymphotoxin beta receptor	-0.338
60360648	KLHL2	kelch like family member 2	-0.338
162135927	DCLRE1C	DNA cross-link repair 1C	-0.338
300795679	CD84	CD84 molecule	-0.338
672084347	N/A	N/A	-0.338
149044388	N/A	N/A	-0.337
300797558	ROPN1L	rhophilin associated tail protein 1 like	-0.337
71361669	CIT	citron rho-interacting serine/threonine kinase	-0.336
625254629	N/A	N/A	-0.336
672081597	AGTR1	angiotensin II receptor type 1	-0.336
564339225	N/A	N/A	-0.335
672046176	N/A	N/A	-0.335
148680122	UNC5C	unc-5 netrin receptor C	-0.335
57527061	ZGPAT	zinc finger CCCH-type and G-patch domain containing	-0.335
148683194	INTS3	integrator complex subunit 3	-0.335
564317005	TBC1D1	TBC1 domain family member 1	-0.335
672073977	LOC103690089	pleckstrin homology domain-containing family A member 6-like	-0.334

672085480	TLDC1	TBC/LysM-associated domain containing 1	-0.334
78486544	SLC5A2	solute carrier family 5 member 2	-0.334
157819371	SYNGR3	synaptogyrin 3	-0.333
157822521	EPPIN-WFDC6	EPPIN-WFDC6 readthrough	-0.333
157819569	TEAD2	TEA domain transcription factor 2	-0.333
18426814	DHFR	dihydrofolate reductase	-0.333
13786174	TIMELESS	timeless circadian regulator	-0.332
57527498	KLC4	kinesin light chain 4	-0.331
537212823	N/A	N/A	-0.331
77539756	MED24	mediator complex subunit 24	-0.331
157823597	SLC39A5	solute carrier family 39 member 5	-0.331
524957514	N/A	N/A	-0.331
47059110	DXO	decapping exoribonuclease	-0.330
13592129	DOC2B	double C2 domain beta	-0.330
149046900	N/A	N/A	-0.330
569006768	N/A	N/A	-0.330
300794275	MFSD10	major facilitator superfamily domain containing 10	-0.330
672038615	GSG1L	GSG1 like	-0.330
300795285	Cyp4f17	cytochrome P450, family 4, subfamily f, polypeptide 17	-0.330
157824146	ITGA5	integrin subunit alpha 5	-0.329
564309649	CCDC159	coiled-coil domain containing 159	-0.329
672038342	XYLT1	xylosyltransferase 1	-0.329
13591934	CTBS	chitobiase	-0.329
84781644	TMEM176A	transmembrane protein 176A	-0.329
58865810	NAGA	alpha-N-acetylgalactosaminidase	-0.328
157822761	MICAL1	microtubule associated monooxygenase, calponin and LIM domain containing 1	-0.328
157787183	KCND1	potassium voltage-gated channel subfamily D member 1	-0.328
22024392	KIF1C	kinesin family member 1C	-0.328
77628027	PSMC3IP	PSMC3 interacting protein	-0.328
281604125	Fam50a/LOC100910130	family with sequence similarity 50, member A	-0.327
564393980	ME2	malic enzyme 2	-0.327
564386482	N/A	N/A	-0.327
149030652	TARS2	threonyl-tRNA synthetase 2, mitochondrial	-0.326
166851836	CPA2	carboxypeptidase A2	-0.326
61557206	ZBTB16	zinc finger and BTB domain containing 16	-0.326

21728394	KLHL17	kelch like family member 17	-0.326
564344373	ZMYND8	zinc finger MYND-type containing 8	-0.325
149066394	SAMD12	sterile alpha motif domain containing 12	-0.325
149025799	N/A	N/A	-0.325
149045696	Ccl27a	chemokine (C-C motif) ligand 27A	-0.325
109475601	GPATCH3	G-patch domain containing 3	-0.325
672016444	N/A	N/A	-0.325
672043651	FAM46C	family with sequence similarity 46 member C	-0.324
109481229	DBX2	developing brain homeobox 2	-0.324
18266696	PDE7B	phosphodiesterase 7B	-0.324
157819563	ETHE1	ETHE1, persulfide dioxygenase	-0.324
31377530	RASGRP1	RAS guanyl releasing protein 1	-0.324
62078809	TNFAIP8L2	TNF alpha induced protein 8 like 2	-0.324
72255543	MANBA	mannosidase beta	-0.323
32452540	RHOT2	ras homolog family member T2	-0.323
78214350	COQ9	coenzyme Q9	-0.323
51036680	SLC29A3	solute carrier family 29 member 3	-0.322
6981186	MAS1	MAS1 proto-oncogene, G protein-coupled receptor	-0.322
56119152	ABCB8	ATP binding cassette subfamily B member 8	-0.321
958702531	N/A	N/A	-0.321
45267819	CAV2	caveolin 2	-0.321
451172111	HINT3	histidine triad nucleotide binding protein 3	-0.321
564368910	FN1	fibronectin 1	-0.321
157821925	IFT88	intraflagellar transport 88	-0.320
58865398	LAP3	leucine aminopeptidase 3	-0.320
50510855	RIMKLB	ribosomal modification protein rimK like family member B	-0.320
635102546	N/A	N/A	-0.320
672066518	N/A	N/A	-0.320
672063876	MGC116197 (includes others)	similar to RIKEN cDNA 1700001E04	-0.320
672033554	LOC102557335	uncharacterized LOC102557335	-0.319
564304076	FGD5	FYVE, RhoGEF and PH domain containing 5	-0.319
18426850	LCP2	lymphocyte cytosolic protein 2	-0.318
672079756	N/A	N/A	-0.318
672026392	PNPLA6	patatin like phospholipase domain containing 6	-0.318
148705386	SLC30A3	solute carrier family 30 member 3	-0.318
672050244	APLF	aprataxin and PNKP like factor	-0.318
6678297	TEX261	testis expressed 261	-0.318

148702301	CYB561	cytochrome b561	-0.317
564343903	DSN1	DSN1 homolog, MIS12 kinetochore complex component	-0.317
672043401	POGZ	pogo transposable element derived with ZNF domain	-0.317
157823879	NUDT12	nudix hydrolase 12	-0.317
157819457	MAP3K14	mitogen-activated protein kinase kinase kinase 14	-0.317
19424260	CDC25B	cell division cycle 25B	-0.317
755536182	RHBDF1	rhomboid 5 homolog 1	-0.317
51948466	TMED3	transmembrane p24 trafficking protein 3	-0.316
40018538	ADI1	acireductone dioxygenase 1	-0.316
443940	CCNE1	cyclin E1	-0.316
139948516	MANEA	mannosidase endo-alpha	-0.316
564318054	R3hcc1	R3H domain and coiled-coil containing 1	-0.316
157821205	PAOX	polyamine oxidase	-0.316
70794782	RBMS2	RNA binding motif single stranded interacting protein 2	-0.316
300797242	SPG11	SPG11, spatacsin vesicle trafficking associated	-0.316
149052198	NPRL3	NPR3 like, GATOR1 complex subunit	-0.315
57528352	DMAC2	distal membrane arm assembly complex 2	-0.315
158631250	HAUS8	HAUS augmin like complex subunit 8	-0.315
672080674	Myo16	myosin XVI	-0.315
16758502	HCN3	hyperpolarization activated cyclic nucleotide gated potassium channel 3	-0.314
8393896	PACSIN1	protein kinase C and casein kinase substrate in neurons 1	-0.314
149021160	N/A	N/A	-0.313
281332095	RB1	RB transcriptional corepressor 1	-0.313
149041432	THY1	Thy-1 cell surface antigen	-0.313
61557127	NNT	nicotinamide nucleotide transhydrogenase	-0.313
148686123	CEND1	cell cycle exit and neuronal differentiation 1	-0.313
157786608	MRPL55	mitochondrial ribosomal protein L55	-0.313
148702078	CPSF3	cleavage and polyadenylation specific factor 3	-0.312
672084625	LOC100909409 (includes others)	RGD1562660	-0.312
157821393	LRRC20	leucine rich repeat containing 20	-0.312
12849161	NRARP	NOTCH regulated ankyrin repeat protein	-0.312
667290090	N/A	N/A	-0.312
210033118	COG1	component of oligomeric golgi complex 1	-0.311

68163523	TTC26	tetratricopeptide repeat domain 26	-0.311
109484871	HERC1	HECT and RLD domain containing E3 ubiquitin protein ligase family member 1	-0.310
564360941	N/A	N/A	-0.310
63101489	ACHE	acetylcholinesterase (Cartwright blood group)	-0.310
157823815	ILVBL	ilvB acetolactate synthase like	-0.310
78187981	TRPV2	transient receptor potential cation channel subfamily V member 2	-0.309
564336403	EXOSC8	exosome component 8	-0.309
62078551	GNB4	G protein subunit beta 4	-0.309
149063353	IFT81	intraflagellar transport 81	-0.309
392342224	N/A	N/A	-0.308
197313632	FRZB	frizzled related protein	-0.308
38541109	BSPRY	B-box and SPRY domain containing	-0.308
167860097	FN3KRP	fructosamine 3 kinase related protein	-0.308
672067893	N/A	N/A	-0.308
12621108	NR1I3	nuclear receptor subfamily 1 group I member 3	-0.308
16758084	CCS	copper chaperone for superoxide dismutase	-0.308
77628037	PEX7	peroxisomal biogenesis factor 7	-0.307
171847060	TTC8	tetratricopeptide repeat domain 8	-0.306
61556910	SNX10	sorting nexin 10	-0.306
83320086	RBM43	RNA binding motif protein 43	-0.306
966975500	MMP17	matrix metallopeptidase 17	-0.306
58865938	CRYZ	crystallin zeta	-0.306
29789369	PTPRG	protein tyrosine phosphatase, receptor type G	-0.306
564326692	ZC3H4	zinc finger CCCH-type containing 4	-0.306
545532952	EIF4E3	eukaryotic translation initiation factor 4E family member 3	-0.305
115292425	KIRREL3	kirre like nephrin family adhesion molecule 3	-0.305
564372688	RPA1	replication protein A1	-0.305
399124777	GLS2	glutaminase 2	-0.305
16758656	BAK1	BCL2 antagonist/killer 1	-0.305
755566690	HUWE1	HECT, UBA and WWE domain containing 1, E3 ubiquitin protein ligase	-0.305
655851627	N/A	N/A	-0.304
11119239	SYT13	synaptotagmin 13	-0.304
568961602	VPS13C	vacuolar protein sorting 13 homolog C	-0.304
31982028	RSU1	Ras suppressor protein 1	-0.304
564356909	N/A	N/A	-0.304
31982487	BMP7	bone morphogenetic protein 7	-0.304

672080024	N/A	N/A	-0.304
300796069	THADA	THADA, armadillo repeat containing	-0.303
56090361	EPDR1	ependymin related 1	-0.303
149023323	GFRA4	GDNF family receptor alpha 4	-0.303
157819589	BOLA3	bolA family member 3	-0.303
564352420	MKNK1	MAP kinase interacting serine/threonine kinase 1	-0.302
198041989	PARVB	parvin beta	-0.302
13929176	WFS1	wolframin ER transmembrane glycoprotein	-0.302
564376043	PIGP	phosphatidylinositol glycan anchor biosynthesis class P	-0.302
6978575	TSPO	translocator protein	-0.302
13540624	GRK5	G protein-coupled receptor kinase 5	-0.302
40352944	NXT2	nuclear transport factor 2 like export factor 2	-0.302
564341926	N/A	N/A	-0.301
149024259	RCAN3	RCAN family member 3	-0.301
672066518	N/A	N/A	-0.301
149024818	MIB2	mindbomb E3 ubiquitin protein ligase 2	-0.300
21245116	Nradd	neurotrophin receptor associated death domain	-0.299
198278496	C1R	complement C1r	-0.299
12621142	RASSF9	Ras association domain family member 9	-0.299
158635969	FLAD1	flavin adenine dinucleotide synthetase 1	-0.299
124107592	MYO1C	myosin IC	-0.298
149030301	PNOC	prepronociceptin	-0.298
564365342	N/A	N/A	-0.298
157820737	NUSAP1	nucleolar and spindle associated protein 1	-0.298
210031334	NGEF	neuronal guanine nucleotide exchange factor	-0.298
564397303	CCDC167	coiled-coil domain containing 167	-0.297
19173736	SCPEP1	serine carboxypeptidase 1	-0.297
672040378	N/A	N/A	-0.297
38259192	TOP2A	DNA topoisomerase II alpha	-0.297
160961483	Serinc4	serine incorporator 4	-0.297
162287198	HSD17B4	hydroxysteroid 17-beta dehydrogenase 4	-0.297
954249788	N/A	N/A	-0.296
149024084	COL16A1	collagen type XVI alpha 1 chain	-0.296
148705684	SLIT2	slit guidance ligand 2	-0.296
157822933	ZNF385A	zinc finger protein 385A	-0.296
149016018	N/A	N/A	-0.296
83025052	ANKS6	ankyrin repeat and sterile alpha motif domain containing 6	-0.296

51036684	G6PC3	glucose-6-phosphatase catalytic subunit 3	-0.296
149032924	ARG1	arginase 1	-0.296
109491454	UTP6	UTP6, small subunit processome component	-0.296
6978789	SPARCL1	SPARC like 1	-0.295
404247454	COL26A1	collagen type XXVI alpha 1 chain	-0.295
686661093	SLC24A3	solute carrier family 24 member 3	-0.295
8393992	PMP22	peripheral myelin protein 22	-0.295
731271938	N/A	N/A	-0.294
58865848	PLSCR3	phospholipid scramblase 3	-0.294
403048729	PAQR9	progestin and adipoQ receptor family member 9	-0.294
149039905	TSPAN17	tetraspanin 17	-0.294
6978888	GFRA1	GDNF family receptor alpha 1	-0.294
27465523	KCNA1	potassium voltage-gated channel subfamily A member 1	-0.294
6981474	GFRA2	GDNF family receptor alpha 2	-0.294
68341971	MINDY1	MINDY lysine 48 deubiquitinase 1	-0.294
53850628	NDUFS1	NADH:ubiquinone oxidoreductase core subunit S1	-0.294
984128285	N/A	N/A	-0.294
149058109	HSD17B7	hydroxysteroid 17-beta dehydrogenase 7	-0.294
762005996	AFAP1L2	actin filament associated protein 1 like 2	-0.293
672035779	Proser3	proline and serine rich 3	-0.293
167560911	SGF29	SAGA complex associated factor 29	-0.293
149023046	N/A	N/A	-0.293
164519052	ARSA	arylsulfatase A	-0.292
55741426	NFKBIB	NFKB inhibitor beta	-0.292
564358911	CHPT1	choline phosphotransferase 1	-0.292
157822577	MAN1C1	mannosidase alpha class 1C member 1	-0.292
215272415	LMO2	LIM domain only 2	-0.292
28972363	DOCK4	dedicator of cytokinesis 4	-0.292
83816933	AP4M1	adaptor related protein complex 4 mu 1 subunit	-0.291
157817979	Egfem1	EGF-like and EMI domain containing 1	-0.291
148701441	N/A	N/A	-0.291
672065746	SCLY	selenocysteine lyase	-0.291
27545388	ABCA5	ATP binding cassette subfamily A member 5	-0.291
564400249	LIMA1	LIM domain and actin binding 1	-0.290
820987601	N/A	N/A	-0.290
670979961	N/A	N/A	-0.290
74271892	RPAIN	RPA interacting protein	-0.290
402794599	PRR22	proline rich 22	-0.290

197313640	TMEM132E	transmembrane protein 132E	-0.290
74223968	5031425E22Rik	RIKEN cDNA 5031425E22 gene	-0.290
149057745	NEK3	NIMA related kinase 3	-0.290
30519995	SFXN5	sideroflexin 5	-0.289
149063995	GMPR2	guanosine monophosphate reductase 2	-0.289
162287200	CD82	CD82 molecule	-0.289
537140956	N/A	N/A	-0.289
310772205	MAP7	microtubule associated protein 7	-0.289
564302385	C20orf196	chromosome 20 open reading frame 196	-0.289
12018270	PNPO	pyridoxamine 5'-phosphate oxidase	-0.288
12844128	CCDC90B	coiled-coil domain containing 90B	-0.288
157821439	CPNE5	copine 5	-0.288
672083256	MYO5B	myosin VB	-0.287
27465571	BRINP3	BMP/retinoic acid inducible neural specific 3	-0.287
157820517	CARD6	caspase recruitment domain family member 6	-0.287
157786648	SMYD4	SET and MYND domain containing 4	-0.287
149022622	ACP2	acid phosphatase 2, lysosomal	-0.287
564387894	BTD	biotinidase	-0.287
157820833	HERC3	HECT and RLD domain containing E3 ubiquitin protein ligase 3	-0.287
402765953	0610009B22Rik	RIKEN cDNA 0610009B22 gene	-0.287
51948412	ETFB	electron transfer flavoprotein beta subunit	-0.286
6981208	NR3C2	nuclear receptor subfamily 3 group C member 2	-0.286
198386336	NAT9	N-acetyltransferase 9 (putative)	-0.286
406362834	MYLIP	myosin regulatory light chain interacting protein	-0.286
9507083	SEMA4F	ssemaphorin 4F	-0.286
8394443	TFPI	tissue factor pathway inhibitor	-0.286
16758138	POMT1	protein O-mannosyltransferase 1	-0.285
300669604	ADAM15	ADAM metallopeptidase domain 15	-0.285
157818065	GPR21	G protein-coupled receptor 21	-0.285
564329612	ME3	malic enzyme 3	-0.284
564319191	MCPH1	microcephalin 1	-0.284
6754024	GNG4	G protein subunit gamma 4	-0.284
672013431	N/A	N/A	-0.283
392338478	TTC37	tetratricopeptide repeat domain 37	-0.283
672074685	ILDR2	immunoglobulin like domain containing receptor 2	-0.283
189181708	RWDD2B	RWD domain containing 2B	-0.283
76559913	COG7	component of oligomeric golgi complex 7	-0.283
564396646	VARS2	valyl-tRNA synthetase 2, mitochondrial	-0.283

194272139	TCERG1L	transcription elongation regulator 1 like	-0.283
56605636	TMEM254	transmembrane protein 254	-0.283
149034870	RNF6	ring finger protein 6	-0.283
149053229	CAMTA2	calmodulin binding transcription activator 2	-0.282
21489989	KCNH8	potassium voltage-gated channel subfamily H member 8	-0.282
157823996	ELK3	ELK3, ETS transcription factor	-0.282
564367076	Mocs1	molybdenum cofactor synthesis 1	-0.281
564317068	CCDC149	coiled-coil domain containing 149	-0.281
672055289	PLEKHH2	pleckstrin homology, MyTH4 and FERM domain containing H2	-0.281
392338823	TIPARP	TCDD inducible poly(ADP-ribose) polymerase	-0.281
13786144	PREP	prolyl endopeptidase	-0.281
197313643	GLTP	glycolipid transfer protein	-0.280
261599034	IL1RAPL2	interleukin 1 receptor accessory protein like 2	-0.280
564361358	PPARA	peroxisome proliferator activated receptor alpha	-0.280
755520134	N/A	N/A	-0.280
16758716	CACNB2	calcium voltage-gated channel auxiliary subunit beta 2	-0.279
402747140	SPN	sialophorin	-0.279
50510949	N/A	N/A	-0.279
149025408	N/A	N/A	-0.279
77539442	EPHX1	epoxide hydrolase 1	-0.279
60097941	HP	haptoglobin	-0.279
457866801	PCSK5	proprotein convertase subtilisin/kexin type 5	-0.279
142349612	GLUL	glutamate-ammonia ligase	-0.279
197927244	TIE1	tyrosine kinase with immunoglobulin like and EGF like domains 1	-0.278
72255507	CD68	CD68 molecule	-0.278
13928886	MAP2K1	mitogen-activated protein kinase kinase 1	-0.278
21728400	Ggta1	glycoprotein galactosyltransferase alpha 1, 3	-0.278
157818005	HPS3	HPS3, biogenesis of lysosomal organelles complex 2 subunit 1	-0.277
148701441	N/A	N/A	-0.277
21687008	CACNG3	calcium voltage-gated channel auxiliary subunit gamma 3	-0.277
209447075	DLGAP5	DLG associated protein 5	-0.276
672073723	GLI2	GLI family zinc finger 2	-0.276
56090612	CDCA3	cell division cycle associated 3	-0.276

564364473	RNF111	ring finger protein 111	-0.276
29789104	NAPB	NSF attachment protein beta	-0.276
149016843	N/A	N/A	-0.275
330340430	WDR19	WD repeat domain 19	-0.275
56090313	MOCS2	molybdenum cofactor synthesis 2	-0.275
672082700	N/A	N/A	-0.275
672083159	N/A	N/A	-0.274
226874871	OMG	oligodendrocyte myelin glycoprotein	-0.274
18959266	KHDRBS2	KH RNA binding domain containing, signal transduction associated 2	-0.274
48843737	FANCD2	Fanconi anemia complementation group D2	-0.274
134948398	PDS5A	PDS5 cohesin associated factor A	-0.274
213972556	OXSM	3-oxoacyl-ACP synthase, mitochondrial	-0.273
157819133	GPR75	G protein-coupled receptor 75	-0.273
157822627	PLXDC2	plexin domain containing 2	-0.273
62078997	WDR1	WD repeat domain 1	-0.273
62079057	IL33	interleukin 33	-0.273
56605664	METTL23	methyltransferase like 23	-0.272
12018318	NPHS1	NPHS1, nephrin	-0.272
149039219	SARDH	sarcosine dehydrogenase	-0.272
586547666	N/A	N/A	-0.272
157820147	TNFRSF10A	TNF receptor superfamily member 10a	-0.272
532055548	N/A	N/A	-0.271
157820447	SDSL	serine dehydratase like	-0.271
29126232	SLCO3A1	solute carrier organic anion transporter family member 3A1	-0.271
197333840	CAMKMT	calmodulin-lysine N-methyltransferase	-0.270
157821645	C8orf59	chromosome 8 open reading frame 59	-0.270
149022319	AGPS	alkylglycerone phosphate synthase	-0.270
197387642	ZNF710	zinc finger protein 710	-0.270
392339871	PARP12	poly(ADP-ribose) polymerase family member 12	-0.270
38454238	Rab15	RAB15, member RAS oncogene family	-0.270
157819337	SLC35B4	solute carrier family 35 member B4	-0.270
158508684	BCAS1	breast carcinoma amplified sequence 1	-0.270
149068830	SLCO2B1	solute carrier organic anion transporter family member 2B1	-0.270
148701892	EBF1	early B cell factor 1	-0.270
66730535	Armcx1/LOC102554790	armadillo repeat containing, X-linked 1	-0.270

57164113	NSDHL	NAD(P) dependent steroid dehydrogenase-like	-0.269
524955710	N/A	N/A	-0.269
672076787	N/A	N/A	-0.269
576796148	MAP7D2	MAP7 domain containing 2	-0.269
157817953	RPGRIP1L	RPGRIP1 like	-0.269
300795283	SHROOM4	shroom family member 4	-0.269
13027442	ARHGEF11	Rho guanine nucleotide exchange factor 11	-0.269
74354506	ACBD5	acyl-CoA binding domain containing 5	-0.269
219804406	DOCK1	dedicator of cytokinesis 1	-0.269
25742783	PLK1	polo like kinase 1	-0.269
149016965	GRB10	growth factor receptor bound protein 10	-0.268
148690381	BRICD5	BRICHOS domain containing 5	-0.268
197313676	AIG1	androgen induced 1	-0.268
635147633	N/A	N/A	-0.268
392339847	CADPS2	calcium dependent secretion activator 2	-0.268
928136440	SRRT	serrate, RNA effector molecule	-0.268
149040074	FAM107A	family with sequence similarity 107 member A	-0.267
10242377	GRIK4	glutamate ionotropic receptor kainate type subunit 4	-0.267
157821289	SOX8	SRY-box 8	-0.267
38181552	SCG2	secretogranin II	-0.267
11177880	VAPB	VAMP associated protein B and C	-0.267
158631185	XPO5	exportin 5	-0.267
300797332	DCP1A	decapping mRNA 1A	-0.267
672033256	LOC100912904	disks large homolog 5-like	-0.267
149017535	HDAC10	histone deacetylase 10	-0.267
52138628	RAP1B	RAP1B, member of RAS oncogene family	-0.267
157786622	ALOXE3	arachidonate lipoxygenase 3	-0.266
157819753	RCN1	reticulocalbin 1	-0.266
859847521	N/A	N/A	-0.266
13786142	SLIT3	slit guidance ligand 3	-0.266
672051414	N/A	N/A	-0.266
564338026	BCL9	B cell CLL/lymphoma 9	-0.265
300795339	RYR2	ryanodine receptor 2	-0.265
29789271	RAB13	RAB13, member RAS oncogene family	-0.265
157820795	BBS1	Bardet-Biedl syndrome 1	-0.265
564382316	HSD11B1	hydroxysteroid 11-beta dehydrogenase 1	-0.265
208973280	TRIM65	tripartite motif containing 65	-0.265
387935412	ZCCHC4	zinc finger CCHC-type containing 4	-0.264
672037217	OTUD7A	OTU deubiquitinase 7A	-0.264

149041411	SC5D	sterol-C5-desaturase	-0.264
148372343	RAMP2	receptor activity modifying protein 2	-0.264
205294	ME1	malic enzyme 1	-0.264
219275534	VPS13A	vacuolar protein sorting 13 homolog A	-0.264
148668227	GPC6	glypican 6	-0.264
13540701	OGG1	8-oxoguanine DNA glycosylase	-0.264
672083937	TSHZ1	teashirt zinc finger homeobox 1	-0.264
157822535	LATS2	large tumor suppressor kinase 2	-0.264
148703340	SERTM1	serine rich and transmembrane domain containing 1	-0.263
158341630	MPG	N-methylpurine DNA glycosylase	-0.263
826286496	N/A	N/A	-0.263
157821975	ZCCHC24	zinc finger CCHC-type containing 24	-0.263
148696365	AP5S1	adaptor related protein complex 5 sigma 1 subunit	-0.262
157819183	FAM212B	family with sequence similarity 212 member B	-0.262
149020634	TAF1D	TATA-box binding protein associated factor, RNA polymerase I subunit D	-0.262
149054651	N/A	N/A	-0.262
403225019	SGO2	shugoshin 2	-0.262
747165376	BUB1B	BUB1 mitotic checkpoint serine/threonine kinase B	-0.262
281427203	TMEM260	transmembrane protein 260	-0.262
564335541	RGD1310081	similar to hypothetical protein FLJ13231	-0.262
157821525	GLYCTK	glycerate kinase	-0.261
564344520	LOC102555457	engulfment and cell motility protein 2-like	-0.261
524957674	N/A	N/A	-0.261
672048779	N/A	N/A	-0.261
227913	N/A	N/A	-0.261
157817017	MRPS16	mitochondrial ribosomal protein S16	-0.261
77627987	DARS2	aspartyl-tRNA synthetase 2, mitochondrial	-0.261
564389540	MGC116197 (includes others)	similar to RIKEN cDNA 1700001E04	-0.260
564372831	N/A	N/A	-0.260
13929166	CLIC4	chloride intracellular channel 4	-0.259
157821191	CHST11	carbohydrate sulfotransferase 11	-0.259
77993374	ARSB	arylsulfatase B	-0.259
157817284	MRPL42	mitochondrial ribosomal protein L42	-0.259
18266702	BNIP1	BCL2 interacting protein 1	-0.258
564382292	ANGEL2	angel homolog 2	-0.258

594667802	N/A	N/A	-0.258
149023046	N/A	N/A	-0.258
149060525	FSTL1	follistatin like 1	-0.258
114145534	Mtap	methylthioadenosine phosphorylase	-0.258
157787147	TEK	TEK receptor tyrosine kinase	-0.258
109476714	FOCAD	focadhesin	-0.258
157820403	RARS2	arginyl-tRNA synthetase 2, mitochondrial	-0.257
625270039	N/A	N/A	-0.257
17865325	GLRB	glycine receptor beta	-0.256
148700512	NRSN1	neurensin 1	-0.256
58865672	CAGE1	cancer antigen 1	-0.256
21245094	MAN2C1	mannosidase alpha class 2C member 1	-0.256
300795738	RASSF8	Ras association domain family member 8	-0.255
148671944	N/A	N/A	-0.255
255708437	PIK3CD	phosphatidylinositol-4,5-bisphosphate 3-kinase catalytic subunit delta	-0.255
140971205	GRIN2A	glutamate ionotropic receptor NMDA type subunit 2A	-0.255
667299645	N/A	N/A	-0.255
564344961	RTEL1	regulator of telomere elongation helicase 1	-0.254
9507167	SYNGR1	synaptogyrin 1	-0.254
51948504	KNSTRN	kinetochore localized astrin/SPAG5 binding protein	-0.254
149017194	KDM3B	lysine demethylase 3B	-0.254
564341663	SLC43A3	solute carrier family 43 member 3	-0.254
57164145	NT5DC2	5'-nucleotidase domain containing 2	-0.254
16758538	RASGRF2	Ras protein specific guanine nucleotide releasing factor 2	-0.254
470611409	N/A	N/A	-0.254
953867923	N/A	N/A	-0.254
50510975	RUFY2	RUN and FYVE domain containing 2	-0.253
564346652	ZNF775	zinc finger protein 775	-0.253
564320452	SAP130	Sin3A associated protein 130	-0.253
157818605	ABCG4	ATP binding cassette subfamily G member 4	-0.253
62078973	MIF4GD	MIF4G domain containing	-0.253
672041250	ARHGEF28	Rho guanine nucleotide exchange factor 28	-0.253
22902132	RBM10	RNA binding motif protein 10	-0.252
18034793	GABRG1	gamma-aminobutyric acid type A receptor gamma1 subunit	-0.252

148696839	RFXANK	regulatory factor X associated ankyrin containing protein	-0.252
31560385	RPL21	ribosomal protein L21	-0.252
56605728	TMEM218	transmembrane protein 218	-0.251
148677779	N/A	N/A	-0.251
431910532	N/A	N/A	-0.251
291190715	ITGA8	integrin subunit alpha 8	-0.251
880855451	N/A	N/A	-0.251
664708230	N/A	N/A	-0.251
157786892	POP7	POP7 homolog, ribonuclease P/MRP subunit	-0.250
157823169	LRRC61	leucine rich repeat containing 61	-0.250
158749613	UROD	uroporphyrinogen decarboxylase	-0.250
568959109	CEP164	centrosomal protein 164	-0.250
149046389	ARID5A	AT-rich interaction domain 5A	-0.249
537146226	N/A	N/A	-0.249
12018276	HPSE	heparanase	-0.249
28212254	IGF2BP1	insulin like growth factor 2 mRNA binding protein 1	-0.249
6978435	ACADVL	acyl-CoA dehydrogenase very long chain	-0.249
426357412	N/A	N/A	-0.249
8394502	UBC	ubiquitin C	-0.249
537217423	N/A	N/A	-0.249
149057830	Hgsnat	heparan-alpha-glucosaminide N-acetyltransferase	-0.248
564371801	TBC1D9B	TBC1 domain family member 9B	-0.248
158186732	GFAP	glial fibrillary acidic protein	-0.248
157819311	LRGUK	leucine rich repeats and guanylate kinase domain containing	-0.248
6978485	ALAS2	5'-aminolevulinate synthase 2	-0.248
392341280	NCAPG2	non-SMC condensin II complex subunit G2	-0.248
61097902	ABCC4	ATP binding cassette subfamily C member 4	-0.248
58219512	TMEM120A	transmembrane protein 120A	-0.248
187469267	GPRC5B	G protein-coupled receptor class C group 5 member B	-0.247
128485638	PLOD3	procollagen-lysine,2-oxoglutarate 5-dioxygenase 3	-0.247
157823901	TSPAN9	tetraspanin 9	-0.247
641706489	N/A	N/A	-0.247
157818445	C1orf50	chromosome 1 open reading frame 50	-0.247
564304730	N/A	N/A	-0.246

298493223	TMEM132B	transmembrane protein 132B	-0.246
59858990	UNC13A	unc-13 homolog A	-0.246
27754155	CLIP4	CAP-Gly domain containing linker protein family member 4	-0.246
157819077	TRIM37	tripartite motif containing 37	-0.246
58865500	STOM	stomatin	-0.246
61557172	GULP1	GULP, engulfment adaptor PTB domain containing 1	-0.245
15805026	ZFAND6	zinc finger AN1-type containing 6	-0.245
296439269	PHF10	PHD finger protein 10	-0.245
6978631	CD4	CD4 molecule	-0.245
568974832	YBX2	Y-box binding protein 2	-0.245
54312094	DAGLA	diacylglycerol lipase alpha	-0.244
564391295	DUSP22	dual specificity phosphatase 22	-0.244
672071009	N/A	N/A	-0.244
209364562	Fundc2	FUN14 domain containing 2	-0.244
70794768	HDAC1	histone deacetylase 1	-0.244
300794684	MSH3	mutS homolog 3	-0.244
164607158	PTPRR	protein tyrosine phosphatase, receptor type R	-0.244
861445795	N/A	N/A	-0.244
85861168	SLC39A13	solute carrier family 39 member 13	-0.244
67078466	CYP2U1	cytochrome P450 family 2 subfamily U member 1	-0.244
62543513	PTGR2	prostaglandin reductase 2	-0.244
56090433	GLT8D1	glycosyltransferase 8 domain containing 1	-0.243
564369492	HJURP	Holliday junction recognition protein	-0.243
13162287	DDT	D-dopachrome tautomerase	-0.243
290563168	DUSP3	dual specificity phosphatase 3	-0.243
157823659	Eefsec	eukaryotic elongation factor, selenocysteine-tRNA-specific	-0.243
13242271	SLC6A11	solute carrier family 6 member 11	-0.242
13095924	DRP2	dystrophin related protein 2	-0.242
12018300	AKAP6	A-kinase anchoring protein 6	-0.242
198442857	NEK4	NIMA related kinase 4	-0.242
66730317	LYSMD1	LysM domain containing 1	-0.242
764020083	CLUH	clustered mitochondria homolog	-0.242
77917570	ENDOG	endonuclease G	-0.242
300793894	URB1	URB1 ribosome biogenesis 1 homolog (S. cerevisiae)	-0.242
299829287	DISP2	dispatched RND transporter family member 2	-0.242

25453420	GSTP1	glutathione S-transferase pi 1	-0.241
197382169	CNTROB	centrobin, centriole duplication and spindle assembly protein	-0.241
564319079	N/A	N/A	-0.241
236467366	CGREF1	cell growth regulator with EF-hand domain 1	-0.241
52138603	CYP4F12	cytochrome P450 family 4 subfamily F member 12	-0.241
25742807	RASSF5	Ras association domain family member 5	-0.241
187937143	C2orf42	chromosome 2 open reading frame 42	-0.241
57527332	PSPH	phosphoserine phosphatase	-0.241
149018420	PTH1R	parathyroid hormone 1 receptor	-0.241
54035529	SS18	SS18, nBAF chromatin remodeling complex subunit	-0.241
6978513	APEH	acylaminoacyl-peptide hydrolase	-0.240
672079582	N/A	N/A	-0.240
157818959	KMT5C	lysine methyltransferase 5C	-0.240
97537309	SYNJ1	synaptojanin 1	-0.240
511925477	N/A	N/A	-0.240
12831215	KCNK10	potassium two pore domain channel subfamily K member 10	-0.240
58865958	RDH11	retinol dehydrogenase 11 (all-trans/9-cis/11-cis)	-0.240
194473630	RECQL4	RecQ like helicase 4	-0.240
11177910	HSPA2	heat shock protein family A (Hsp70) member 2	-0.240
57192	P3H4	prolyl 3-hydroxylase family member 4 (non-enzymatic)	-0.240
157818257	SFXN4	sideroflexin 4	-0.239
589922499	N/A	N/A	-0.239
309319799	EIF2AK4	eukaryotic translation initiation factor 2 alpha kinase 4	-0.239
57527084	HAT1	histone acetyltransferase 1	-0.238
672081577	N/A	N/A	-0.238
672054612	FBXO44	F-box protein 44	-0.238
157820535	GATC	glutamyl-tRNA amidotransferase subunit C	-0.238
564344879	N/A	N/A	-0.237
149033803	CDKL2	cyclin dependent kinase like 2	-0.237
119310200	KDM4D	lysine demethylase 4D	-0.237
157822599	GSAP	gamma-secretase activating protein	-0.237
51948438	PYROXD1	pyridine nucleotide-disulphide oxidoreductase domain 1	-0.237

158749602	TRAM1L1	translocation associated membrane protein 1 like 1	-0.237
291490673	NGFR	nerve growth factor receptor	-0.236
60359932	PPM1H	protein phosphatase, Mg2+/Mn2+ dependent 1H	-0.236
449784888	ALDH5A1	aldehyde dehydrogenase 5 family member A1	-0.236
564342542	MAP1A	microtubule associated protein 1A	-0.236
160333179	FBXO27	F-box protein 27	-0.236
672047003	CDAN1	codanin 1	-0.236
56605774	NFKB2	nuclear factor kappa B subunit 2	-0.235
157817480	RWDD2A	RWD domain containing 2A	-0.235
672014800	N/A	N/A	-0.235
157817015	LDLRAP1	low density lipoprotein receptor adaptor protein 1	-0.234
564314389	DZIP3	DAZ interacting zinc finger protein 3	-0.234
149016209	SLC4A3	solute carrier family 4 member 3	-0.234
672062815	N/A	N/A	-0.234
149066401	AARD	alanine and arginine rich domain containing protein	-0.234
40786477	SNAP47	synaptosome associated protein 47	-0.234
77404265	JAM2	junctional adhesion molecule 2	-0.233
157818279	MMACHC	methylmalonic aciduria (cobalamin deficiency) cblC type, with homocystinuria	-0.233
62543563	KYAT3	kynurenine aminotransferase 3	-0.233
62078935	ALS2CR12	amyotrophic lateral sclerosis 2 chromosome region 12	-0.233
48040475	GCNT2	glucosaminyl (N-acetyl) transferase 2, I-branching enzyme (I blood group)	-0.233
584277046	SLC1A3	solute carrier family 1 member 3	-0.232
213688380	GXYLT1	glucoside xylosyltransferase 1	-0.232
149053021	TMEM107	transmembrane protein 107	-0.232
306482651	DNAJB14	DnaJ heat shock protein family (Hsp40) member B14	-0.232
564358836	NT5DC3	5'-nucleotidase domain containing 3	-0.232
748983393	ZBTB4	zinc finger and BTB domain containing 4	-0.232
594661984	N/A	N/A	-0.232
293346766	TCAF1	TRPM8 channel associated factor 1	-0.232
9457244	RBBP9	RB binding protein 9, serine hydrolase	-0.231
13489067	NSF	N-ethylmaleimide sensitive factor, vesicle fusing ATPase	-0.231

158749644	MCM6	minichromosome maintenance complex component 6	-0.231
149029770	N/A	N/A	-0.231
157818341	PPP1R3D	protein phosphatase 1 regulatory subunit 3D	-0.231
158261984	PSMG2	proteasome assembly chaperone 2	-0.231
149028840	N/A	N/A	-0.230
30025028	AMIGO3	adhesion molecule with Ig like domain 3	-0.230
56605668	FLII	FLII, actin remodeling protein	-0.230
157817839	SEMA5A	semaphorin 5A	-0.230
564322686	N/A	N/A	-0.230
197927216	TBC1D5	TBC1 domain family member 5	-0.230
157820653	TMEM63C	transmembrane protein 63C	-0.229
81884455	PDE12	phosphodiesterase 12	-0.229
73661200	SPRN	shadow of prion protein	-0.229
13994225	HSD17B10	hydroxysteroid 17-beta dehydrogenase 10	-0.229
634833336	N/A	N/A	-0.229
404434384	GALNT11	polypeptide N-acetylgalactosaminyltransferase 11	-0.229
564397761	GCC2	GRIP and coiled-coil domain containing 2	-0.229
300797244	RHBDD2	rhomboid domain containing 2	-0.229
8393355	FDX1	ferredoxin 1	-0.229
58865718	HERC4	HECT and RLD domain containing E3 ubiquitin protein ligase 4	-0.228
12248187	P2RY12	purinergic receptor P2Y12	-0.228
392334157	SEMA6A	semaphorin 6A	-0.228
672084703	N/A	N/A	-0.228
149062310	BSCL2	BSCL2, seipin lipid droplet biogenesis associated	-0.228
62655853	TELO2	telomere maintenance 2	-0.228
62079099	ORC5	origin recognition complex subunit 5	-0.228
564389730	PLAT	plasminogen activator, tissue type	-0.228
281485606	STT3B	STT3B, catalytic subunit of the oligosaccharyltransferase complex	-0.228
54312088	ATP2B4	ATPase plasma membrane Ca2+ transporting 4	-0.228
157818709	TMEM205	transmembrane protein 205	-0.227
731267527	N/A	N/A	-0.227
403259801	N/A	N/A	-0.227
149033235	PYURF	PIGY upstream reading frame	-0.227
51948524	IGFBP4	insulin like growth factor binding protein 4	-0.226
672050038	NDNF	neuron derived neurotrophic factor	-0.226

56799390	ATP1B2	ATPase Na+/K+ transporting subunit beta 2	-0.226
564384526	TACC3	transforming acidic coiled-coil containing protein 3	-0.226
672072960	KNTC1	kinetochore associated 1	-0.226
160406706	SH3GL3	SH3 domain containing GRB2 like 3, endophilin A3	-0.226
205277356	TVP23B	trans-golgi network vesicle protein 23 homolog B	-0.226
62079015	FAM213A	family with sequence similarity 213 member A	-0.226
157819139	PITRM1	pitrilysin metallopeptidase 1	-0.226
157817710	FER	FER tyrosine kinase	-0.225
62078843	TMEM209	transmembrane protein 209	-0.225
293345175	DHX29	DExH-box helicase 29	-0.225
13592117	KLF10	Kruppel like factor 10	-0.225
399154129	POP5	POP5 homolog, ribonuclease P/MRP subunit	-0.225
62078989	CCDC181	coiled-coil domain containing 181	-0.225
672055862	N/A	N/A	-0.225
564383995	EVC	EvC ciliary complex subunit 1	-0.225
157819921	ZNF385B	zinc finger protein 385B	-0.224
61557398	ZDHHC12	zinc finger DHHC-type containing 12	-0.224
672048013	N/A	N/A	-0.224
25742576	NXF1	nuclear RNA export factor 1	-0.224
112983748	VSIR	V-set immunoregulatory receptor	-0.223
157818897	VANGL1	VANGL planar cell polarity protein 1	-0.223
13928806	P2RX4	purinergic receptor P2X 4	-0.223
157818805	DCLRE1A	DNA cross-link repair 1A	-0.223
672040941	ATRNL1	attractin like 1	-0.223
392348438	NIPAL3	NIPA like domain containing 3	-0.223
155369680	Ces2b	carboxyesterase 2B	-0.222
148697866	FAM3A	family with sequence similarity 3 member A	-0.222
209529673	PLEKHG2	pleckstrin homology and RhoGEF domain containing G2	-0.222
157786994	C1orf21	chromosome 1 open reading frame 21	-0.222
157818283	UQCC2	ubiquinol-cytochrome c reductase complex assembly factor 2	-0.222
157820311	OTUD6B	OTU domain containing 6B	-0.222
913512216	N/A	N/A	-0.222
672037313	N/A	N/A	-0.222
78369663	SLC38A9	solute carrier family 38 member 9	-0.222
533133321	N/A	N/A	-0.222

148689145	CPNE4	copine 4	-0.222
149061976	N/A	N/A	-0.222
74179798	PCSK2	proprotein convertase subtilisin/kexin type 2	-0.221
158711736	SMC2	structural maintenance of chromosomes 2	-0.221
62945338	EIF2D	eukaryotic translation initiation factor 2D	-0.221
56605770	RRP8	ribosomal RNA processing 8	-0.221
197927172	ZMAT4	zinc finger matrin-type 4	-0.221
61557009	SNAPC2	small nuclear RNA activating complex polypeptide 2	-0.221
564342402	PLA2G4B	phospholipase A2 group IVB	-0.221
149067833	ALDOA	aldolase, fructose-bisphosphate A	-0.221
148695758	CAPRIN1	cell cycle associated protein 1	-0.221
197246365	SP2	Sp2 transcription factor	-0.220
197927395	CCDC40	coiled-coil domain containing 40	-0.220
296010823	UBR1	ubiquitin protein ligase E3 component n-recognin 1	-0.220
157819361	TTYH1	tweety family member 1	-0.220
158631207	YIF1A	Yip1 interacting factor homolog A, membrane trafficking protein	-0.220
672042001	PLD1	phospholipase D1	-0.220
157823887	MLC1	megalencephalic leukoencephalopathy with subcortical cysts 1	-0.220
402766107	ALDH7A1	aldehyde dehydrogenase 7 family member A1	-0.220
148690402	SLC9A3R2	SLC9A3 regulator 2	-0.220
158186672	Nedd4	neural precursor cell expressed, developmentally down-regulated 4	-0.220
564378945	N/A	N/A	-0.219
57527353	TOR3A	torsin family 3 member A	-0.219
672017118	N/A	N/A	-0.219
564306838	ZNF512	zinc finger protein 512	-0.219
157822873	FBXO18	F-box protein, helicase, 18	-0.219
672061591	N/A	N/A	-0.219
402794954	MINK1	misshapen like kinase 1	-0.219
5174513	SMAD3	SMAD family member 3	-0.219
564307561	RPS6KL1	ribosomal protein S6 kinase like 1	-0.218
62079019	UFSP2	UFM1 specific peptidase 2	-0.218
9506469	CD47	CD47 molecule	-0.218
564387660	UBAC2	UBA domain containing 2	-0.217
564315812	NAV1	neuron navigator 1	-0.217

564333746	MMS19	MMS19 homolog, cytosolic iron-sulfur assembly component	-0.217
157817500	UBAP2	ubiquitin associated protein 2	-0.217
148666837	MGLL	monoglyceride lipase	-0.217
148697324	DEPTOR	DEP domain containing MTOR interacting protein	-0.216
913495139	N/A	N/A	-0.216
348605146	HDAC11	histone deacetylase 11	-0.216
148671603	LRP11	LDL receptor related protein 11	-0.216
564298436	WDR11	WD repeat domain 11	-0.216
155369305	PBXIP1	PBX homeobox interacting protein 1	-0.216
344255506	N/A	N/A	-0.216
157821397	SLC22A15	solute carrier family 22 member 15	-0.216
564333977	Cnnm1	cyclin and CBS domain divalent metal cation transport mediator 1	-0.216
9506425	BET1	Bet1 golgi vesicular membrane trafficking protein	-0.215
6978890	GGH	gamma-glutamyl hydrolase	-0.215
880921412	N/A	N/A	-0.215
564344397	TOMM34	translocase of outer mitochondrial membrane 34	-0.215
157821513	HEBP1	heme binding protein 1	-0.215
672088490	N/A	N/A	-0.215
38174623	FXYD7	FXYD domain containing ion transport regulator 7	-0.215
148687519	CALN1	calneuron 1	-0.214
300793998	SHISA6	shisa family member 6	-0.214
672055565	OTOF	otoferlin	-0.214
288541382	DIS3L2	DIS3 like 3'-5' exoribonuclease 2	-0.214
8393643	KCNAB1	potassium voltage-gated channel subfamily A member regulatory beta subunit 1	-0.214
564382871	N/A	N/A	-0.214
149028347	LIG1	DNA ligase 1	-0.214
672083376	N/A	N/A	-0.213
51948390	HSD17B11	hydroxysteroid 17-beta dehydrogenase 11	-0.213
157819089	CXorf40A/CXorf40B	chromosome X open reading frame 40A	-0.213
205755	TAGLN3	transgelin 3	-0.213
58865418	SUGP1	SURP and G-patch domain containing 1	-0.213
807677	N/A	N/A	-0.213

568986622	CNIH1	cornichon family AMPA receptor auxiliary protein 1	-0.213
564398053	MAN1A1	mannosidase alpha class 1A member 1	-0.213
157817061	SIAE	sialic acid acetylesterase	-0.213
827475641	CCDC151	coiled-coil domain containing 151	-0.213
57526927	LARS	leucyl-tRNA synthetase	-0.213
157952208	BAG1	BCL2 associated athanogene 1	-0.212
29789305	PTPRN	protein tyrosine phosphatase, receptor type N	-0.212
672066638	CLEC16A	C-type lectin domain containing 16A	-0.212
564397593	RAB36	RAB36, member RAS oncogene family	-0.212
149066531	VPS13B	vacuolar protein sorting 13 homolog B	-0.212
392338550	IPO11	importin 11	-0.212
664703981	N/A	N/A	-0.212
672051730	N/A	N/A	-0.212
149056788	VASP	vasodilator stimulated phosphoprotein	-0.211
564335892	PEX2	peroxisomal biogenesis factor 2	-0.211
187282043	TMEM179	transmembrane protein 179	-0.211
13928780	POR	cytochrome p450 oxidoreductase	-0.211
635049334	N/A	N/A	-0.211
415703079	NEBL	nebulette	-0.211
158635998	SLC40A1	solute carrier family 40 member 1	-0.211
25742763	HSPA5	heat shock protein family A (Hsp70) member 5	-0.211
157786612	B9D1	B9 domain containing 1	-0.211
158749540	NPEPPS	aminopeptidase puromycin sensitive	-0.210
18959250	PRKCD	protein kinase C delta	-0.210
281306814	RPS6KA2	ribosomal protein S6 kinase A2	-0.210
76096338	ZEB2	zinc finger E-box binding homeobox 2	-0.210
765067	PIM2	Pim-2 proto-oncogene, serine/threonine kinase	-0.209
564386387	EEF1AKMT1	EEF1A lysine methyltransferase 1	-0.209
281371490	LAMC1	laminin subunit gamma 1	-0.209
164414419	SP1	Sp1 transcription factor	-0.209
747811827	HID1	HID1 domain containing	-0.208
880942118	N/A	N/A	-0.208
149058126	ALDH9A1	aldehyde dehydrogenase 9 family member A1	-0.208
19705535	PACS1	phosphofurin acidic cluster sorting protein 1	-0.208
61557385	RNASEH2A	ribonuclease H2 subunit A	-0.207
157822271	DOLK	dolichol kinase	-0.207
672065543	TNS1	tensin 1	-0.207
37359962	PLPPR4	phospholipid phosphatase related 4	-0.207
398303848	RNH1	ribonuclease/angiogenin inhibitor 1	-0.207

225543229	TIAM1	T cell lymphoma invasion and metastasis 1	-0.207
148667192	LRTM2	leucine rich repeats and transmembrane domains 2	-0.207
1335860	PRKAG1	protein kinase AMP-activated non-catalytic subunit gamma 1	-0.207
564385704	FLNB	filamin B	-0.207
300798704	TLL2	tolloid like 2	-0.206
62078695	MLEC	malectin	-0.206
39104626	CAMK2A	calcium/calmodulin dependent protein kinase II alpha	-0.206
157823243	FAM172A	family with sequence similarity 172 member A	-0.206
155369301	ARHGEF37	Rho guanine nucleotide exchange factor 37	-0.206
564372562	PFAS	phosphoribosylformylglycinamidine synthase	-0.206
18266704	TRPC5	transient receptor potential cation channel subfamily C member 5	-0.206
300794237	LIMCH1	LIM and calponin homology domains 1	-0.205
402794666	NRG1	neuregulin 1	-0.205
149048608	N/A	N/A	-0.205
755540019	N/A	N/A	-0.205
9506523	CSPG5	chondroitin sulfate proteoglycan 5	-0.205
54019432	PCDHA7	protocadherin alpha 7	-0.205
31377525	ASL	argininosuccinate lyase	-0.205
206558322	JMJD8	jumonji domain containing 8	-0.205
403310680	GAMT	guanidinoacetate N-methyltransferase	-0.205
6978673	CNR1	cannabinoid receptor 1	-0.205
13540703	PDE1A	phosphodiesterase 1A	-0.205
40786489	ARHGEF25	Rho guanine nucleotide exchange factor 25	-0.204
564343174	PLCB4	phospholipase C beta 4	-0.204
6981504	ATXN1	ataxin 1	-0.204
188536071	DLC1	DLC1 Rho GTPase activating protein	-0.204
564361015	TRIOBP	TRIO and F-actin binding protein	-0.204
149018602	HYAL1	hyaluronoglucosaminidase 1	-0.204
157818191	SETD6	SET domain containing 6	-0.203
148747414	GDA	guanine deaminase	-0.203
564391231	SERPINB9	serpin family B member 9	-0.203
18426866	ACAA2	acetyl-CoA acyltransferase 2	-0.203
149020512	PDE4A	phosphodiesterase 4A	-0.203
568971594	GABRA1	gamma-aminobutyric acid type A receptor alpha1 subunit	-0.203
56605780	PMVK	phosphomevalonate kinase	-0.203

13027430	WDR7	WD repeat domain 7	-0.202
205277353	TRIM16	tripartite motif containing 16	-0.202
149044030	JAG2	jagged 2	-0.202
142385975	RNF25	ring finger protein 25	-0.202
149046617	MAGI2	membrane associated guanylate kinase, WW and PDZ domain containing 2	-0.202
451770389	HMGCLL1	3-hydroxymethyl-3-methylglutaryl-CoA lyase like 1	-0.202
148696104	CKMT1A/CKMT1B	creatine kinase, mitochondrial 1B	-0.202
310750417	FAM98C	family with sequence similarity 98 member C	-0.202
55741502	ACAT2	acetyl-CoA acetyltransferase 2	-0.202
672049447	N/A	N/A	-0.202
198278430	OSBPL9	oxysterol binding protein like 9	-0.202
293340174	DNAH9	dynein axonemal heavy chain 9	-0.202
594062377	N/A	N/A	-0.202
48675867	PLPP3	phospholipid phosphatase 3	-0.201
157786582	C16orf89	chromosome 16 open reading frame 89	-0.201
148693894	UBE2Q2	ubiquitin conjugating enzyme E2 Q2	-0.201
188536087	FAM103A1	family with sequence similarity 103 member A1	-0.201
68163417	FAHD1	fumarylacetoacetate hydrolase domain containing 1	-0.201
309243082	PTPRJ	protein tyrosine phosphatase, receptor type J	-0.201
38454284	PPM1E	protein phosphatase, Mg2+/Mn2+ dependent 1E	-0.201
148707634	SHISA4	shisa family member 4	-0.201
157786976	RGL1	ral guanine nucleotide dissociation stimulator like 1	-0.201
149053315	CAMKK1	calcium/calmodulin dependent protein kinase kinase 1	-0.200
148664829	NMRAL1	NmrA like redox sensor 1	-0.200
149033449	RGD1559747	similar to Zinc finger and SCAN domain containing protein 2 (Zinc finger protein 29)	-0.200
46485382	BHLHB9	basic helix-loop-helix family member b9	-0.200
564387864	CACNA1D	calcium voltage-gated channel subunit alpha1 D	-0.199
564354247	NPHP4	nephrocystin 4	-0.199
62078801	MEF2A	myocyte enhancer factor 2A	-0.199
77404395	SND1	staphylococcal nuclease and tudor domain containing 1	-0.199
564397109	N/A	N/A	-0.199

586975177	N/A	N/A	-0.199
148667088	ATP2B2	ATPase plasma membrane Ca2+ transporting 2	-0.199
148690392	N/A	N/A	-0.199
189163499	CYHR1	cysteine and histidine rich 1	-0.198
61556891	OSBPL2	oxysterol binding protein like 2	-0.198
16758726	SLC17A7	solute carrier family 17 member 7	-0.198
451172098	KCTD1	potassium channel tetramerization domain containing 1	-0.197
149024371	SH2D5	SH2 domain containing 5	-0.197
672089580	LOC103694865	TATA-binding protein-associated factor 2N-like	-0.197
26006243	KCND2	potassium voltage-gated channel subfamily D member 2	-0.197
880871034	N/A	N/A	-0.196
170671744	Mapk1ip1	mitogen-activated protein kinase 1 interacting protein 1	-0.196
9506913	Ndufs6	NADH:ubiquinone oxidoreductase subunit S6	-0.196
149062285	N/A	N/A	-0.195
62738001	N/A	N/A	-0.195
149016262	Col4a4	collagen type IV alpha 4 chain	-0.195
913505527	N/A	N/A	-0.195
11560055	KHDRBS3	KH RNA binding domain containing, signal transduction associated 3	-0.195
157820339	MESP2	mesoderm posterior bHLH transcription factor 2	-0.195
12018246	TSPAN2	tetraspanin 2	-0.195
564334053	SORCS1	sortilin related VPS10 domain containing receptor 1	-0.195
564347675	AAK1	AP2 associated kinase 1	-0.195
149025317	N/A	N/A	-0.195
58865716	PPP1R3C	protein phosphatase 1 regulatory subunit 3C	-0.194
32527705	N/A	N/A	-0.194
13591979	LIFR	LIF receptor alpha	-0.194
157818397	MFSD4A	major facilitator superfamily domain containing 4A	-0.194
840088206	INTS11	integrator complex subunit 11	-0.194
149018361	N/A	N/A	-0.194
32423788	Sept4	septin 4	-0.194
568941844	IQSEC3	IQ motif and Sec7 domain 3	-0.194
11072106	NUCB2	nucleobindin 2	-0.193

283046651	PTPRZ1	protein tyrosine phosphatase, receptor type Z1	-0.193
123780073	YOD1	YOD1 deubiquitinase	-0.193
157818293	MMAA	methylmalonic aciduria (cobalamin deficiency) cblA type	-0.193
564332776	LRRN4CL	LRRN4 C-terminal like	-0.193
564325125	PLEKHG1	pleckstrin homology and RhoGEF domain containing G1	-0.193
201025388	MPLKIP	M-phase specific PLK1 interacting protein	-0.193
62079143	YIPF2	Yip1 domain family member 2	-0.192
752420454	N/A	N/A	-0.192
63706033	Gm5174 (includes others)	serine/threonine kinase, pseudogene 1	-0.192
198278525	RIC8A	RIC8 guanine nucleotide exchange factor A	-0.192
62078931	PAQR8	progestin and adipoQ receptor family member 8	-0.192
157823930	PPP2R5A	protein phosphatase 2 regulatory subunit B'alpha	-0.192
431900674	N/A	N/A	-0.192
206725437	LRRC23	leucine rich repeat containing 23	-0.192
672078392	N/A	N/A	-0.192
13591963	GRM7	glutamate metabotropic receptor 7	-0.191
56090379	POMGNT1	protein O-linked mannose N-acetylglucosaminyltransferase 1 (beta 1,2-)	-0.191
17530977	ECHS1	enoyl-CoA hydratase, short chain 1	-0.191
157822043	PLGRKT	plasminogen receptor with a C-terminal lysine	-0.191
157820155	MRPL32	mitochondrial ribosomal protein L32	-0.191
157821335	GPR162	G protein-coupled receptor 162	-0.191
25282457	CCNB1	cyclin B1	-0.191
564345393	PRKAG2	protein kinase AMP-activated non-catalytic subunit gamma 2	-0.190
672055089	N/A	N/A	-0.190
564372825	SGSM2	small G protein signaling modulator 2	-0.190
18266684	MSMO1	methylsterol monooxygenase 1	-0.190
149027971	ATF6B	activating transcription factor 6 beta	-0.189
293349793	RFTN2	raftlin family member 2	-0.189
672069941	N/A	N/A	-0.189
148696094	TUBGCP4	tubulin gamma complex associated protein 4	-0.189
568950414	ATXN2L	ataxin 2 like	-0.189
201066342	MAN2B2	mannosidase alpha class 2B member 2	-0.189
42491372	ERMP1	endoplasmic reticulum metallopeptidase 1	-0.189
6981112	IVD	isovaleryl-CoA dehydrogenase	-0.189

655718534	N/A	N/A	-0.189
564342864	FAHD2B	fumarylacetoacetate hydrolase domain containing 2B	-0.189
6760457	TIMM22	translocase of inner mitochondrial membrane 22	-0.189
62079155	DALRD3	DALR anticodon binding domain containing 3	-0.189
157819187	AGL	amylo-alpha-1, 6-glucosidase, 4-alpha-glucanotransferase	-0.189
72255531	EFHD2	EF-hand domain family member D2	-0.189
564384429	N/A	N/A	-0.189
157822625	USP28	ubiquitin specific peptidase 28	-0.188
157818171	PLK4	polo like kinase 4	-0.188
564361228	TTLL1	tubulin tyrosine ligase like 1	-0.187
209413784	PRAMEF7/PRAMEF8	PRAME family member 7	-0.187
157822211	CTC1	CST telomere replication complex component 1	-0.187
149063212	TMEM132C	transmembrane protein 132C	-0.187
157823948	ANO6	anoctamin 6	-0.187
68163551	TBC1D22B	TBC1 domain family member 22B	-0.187
564334013	GBF1	golgi brefeldin A resistant guanine nucleotide exchange factor 1	-0.187
564399352	TAF9B	TATA-box binding protein associated factor 9b	-0.187
815891121	ZEB1	zinc finger E-box binding homeobox 1	-0.187
149040053	C3orf14	chromosome 3 open reading frame 14	-0.187
564382208	Gpatch2	G patch domain containing 2	-0.187
300794608	CEP120	centrosomal protein 120	-0.186
157821901	PNMA3	PNMA family member 3	-0.186
672088942	ATP2B3	ATPase plasma membrane Ca2+ transporting 3	-0.186
19705437	EPHA7	EPH receptor A7	-0.186
149046124	SPATS2L	spermatogenesis associated serine rich 2 like	-0.186
169234826	ELP4	elongator acetyltransferase complex subunit 4	-0.186
219803038	PDE2A	phosphodiesterase 2A	-0.186
312283667	WNK1	WNK lysine deficient protein kinase 1	-0.185
197209847	JAK1	Janus kinase 1	-0.185
59891444	FUT10	fucosyltransferase 10	-0.185
149022123	N/A	N/A	-0.185
52138739	HEXA	hexosaminidase subunit alpha	-0.185
674093471	N/A	N/A	-0.185
285026506	IDUA	iduronidase, alpha-L-	-0.185
16923964	CNTN1	contactin 1	-0.185

38051886	RABGGTB	Rab geranylgeranyltransferase beta subunit	-0.185
77628016	BAIAP2L1	BAI1 associated protein 2 like 1	-0.185
859783782	N/A	N/A	-0.185
149064529	N/A	N/A	-0.185
300797496	TDRD6	tudor domain containing 6	-0.184
564333197	TJP2	tight junction protein 2	-0.184
157787095	MIP	major intrinsic protein of lens fiber	-0.184
157822539	ANK1	ankyrin 1	-0.184
112984202	FZD8	frizzled class receptor 8	-0.183
17865335	DGAT1	diacylglycerol O-acyltransferase 1	-0.183
731277128	N/A	N/A	-0.183
157820421	SMIM17	small integral membrane protein 17	-0.183
157817420	NRIP3	nuclear receptor interacting protein 3	-0.183
564313504	TEX2	testis expressed 2	-0.183
76881830	Kcnip2	Kv channel-interacting protein 2	-0.183
300796855	PARD3B	par-3 family cell polarity regulator beta	-0.183
296010825	UBR2	ubiquitin protein ligase E3 component n-recognin 2	-0.182
56605704	SERINC3	serine incorporator 3	-0.182
56605784	MRRF	mitochondrial ribosome recycling factor	-0.182
62078977	PDIA5	protein disulfide isomerase family A member 5	-0.182
148699026	N/A	N/A	-0.182
84490431	DNM3	dynamin 3	-0.182
392343399	COL4A6	collagen type IV alpha 6 chain	-0.182
71037403	MYL12B	myosin light chain 12B	-0.181
149036441	SUCLG1	succinate-CoA ligase alpha subunit	-0.181
83415090	RAB2B	RAB2B, member RAS oncogene family	-0.181
32185285	BCL2L2	BCL2 like 2	-0.181
555987896	N/A	N/A	-0.181
157817620	PSD2	pleckstrin and Sec7 domain containing 2	-0.180
12621120	SFXN3	sideroflexin 3	-0.180
672088357	ZCCHC18	zinc finger CCHC-type containing 18	-0.180
19173794	LOC678813/Marf1	similar to limkain b1	-0.180
9506957	PCSK7	proprotein convertase subtilisin/kexin type 7	-0.180
187468990	DNAJB2	DnaJ heat shock protein family (Hsp40) member B2	-0.180
39930507	KCNK15	potassium two pore domain channel subfamily K member 15	-0.180
201025393	TTC7A	tetratricopeptide repeat domain 7A	-0.180
640833971	N/A	N/A	-0.180

13592087	SOAT1	sterol O-acyltransferase 1	-0.180
149051484	AKAP5	A-kinase anchoring protein 5	-0.180
946639423	N/A	N/A	-0.179
169642755	Pms2	PMS1 homolog 2, mismatch repair system component	-0.179
564361005	PLA2G6	phospholipase A2 group VI	-0.179
195972823	TMEM181	transmembrane protein 181	-0.179
255683359	FBXL17	F-box and leucine rich repeat protein 17	-0.179
564396135	KIAA0513	KIAA0513	-0.178
672042162	TRPC3	transient receptor potential cation channel subfamily C member 3	-0.178
13929208	Scd2	stearoyl-Coenzyme A desaturase 2	-0.178
66730413	FARSA	phenylalanyl-tRNA synthetase alpha subunit	-0.178
6981672	Tpm4	tropomyosin 4	-0.178
293349000	ARHGAP42	Rho GTPase activating protein 42	-0.178
16758644	TXN	thioredoxin	-0.178
13569846	PARVA	parvin alpha	-0.178
156139151	PDS5B	PDS5 cohesin associated factor B	-0.177
8393060	Cacnb1	calcium voltage-gated channel auxiliary subunit beta 1	-0.177
672029737	N/A	N/A	-0.177
564368926	N/A	N/A	-0.177
62955040	ELMOD3	ELMO domain containing 3	-0.177
672017219	ZNF106	zinc finger protein 106	-0.177
18677755	ADGRL3	adhesion G protein-coupled receptor L3	-0.177
564363914	CLK3	CDC like kinase 3	-0.177
201023331	MAPK11	mitogen-activated protein kinase 11	-0.176
564390898	KIF13A	kinesin family member 13A	-0.176
157818193	TTPAL	alpha tocopherol transfer protein like	-0.176
281306738	PCDH19	protocadherin 19	-0.176
537235061	N/A	N/A	-0.176
166063985	PKN1	protein kinase N1	-0.175
149049332	TULP3	tubby like protein 3	-0.175
392334416	TCF25	transcription factor 25	-0.175
157821497	NDUFS8	NADH:ubiquinone oxidoreductase core subunit S8	-0.175
8394091	PSME2	proteasome activator subunit 2	-0.175
78000203	Tpm1	tropomyosin 1, alpha	-0.174
667287162	N/A	N/A	-0.174
392352553	RBM19	RNA binding motif protein 19	-0.174

194240569	TRMT44	tRNA methyltransferase 44 homolog	-0.174
672062144	FAM219B	family with sequence similarity 219 member B	-0.174
61557085	SPTBN1	spectrin beta, non-erythrocytic 1	-0.174
392333710	COL4A2	collagen type IV alpha 2 chain	-0.173
397357	N/A	N/A	-0.173
837798914	N/A	N/A	-0.173
564395567	NFATC3	nuclear factor of activated T cells 3	-0.173
537236584	N/A	N/A	-0.173
157823485	ANGEL1	angel homolog 1	-0.173
166091519	GRHPR	glyoxylate and hydroxypyruvate reductase	-0.172
564387640	DOCK9	dedicator of cytokinesis 9	-0.172
77628157	ST18	ST18, C2H2C-type zinc finger	-0.172
672058640	N/A	N/A	-0.172
124248495	CHID1	chitinase domain containing 1	-0.171
444706842	N/A	N/A	-0.171
300797936	ACTR3B	ARP3 actin related protein 3 homolog B	-0.171
269954719	JAZF1	JAZF zinc finger 1	-0.171
148682229	PDZD11	PDZ domain containing 11	-0.171
157822465	MLH3	mutL homolog 3	-0.171
149019021	Sh3bgrl2	SH3 domain binding glutamate-rich protein like 2	-0.170
149028240	Fsd1	fibronectin type III and SPRY domain containing 1	-0.170
50356003	SCP2	sterol carrier protein 2	-0.170
625260426	N/A	N/A	-0.170
18034783	ABCC3	ATP binding cassette subfamily C member 3	-0.170
62079101	LAMP5	lysosomal associated membrane protein family member 5	-0.170
61557212	NARFL	nuclear prelamin A recognition factor like	-0.170
149030303	ELP3	elongator acetyltransferase complex subunit 3	-0.170
62078711	TMEM175	transmembrane protein 175	-0.170
157786960	PIK3C2B	phosphatidylinositol-4-phosphate 3-kinase catalytic subunit type 2 beta	-0.169
396941666	Dync1i2	dynein cytoplasmic 1 intermediate chain 2	-0.169
149063948	N/A	N/A	-0.169
281604190	INPP5B	inositol polyphosphate-5-phosphatase B	-0.169
440904939	N/A	N/A	-0.169
16758188	SLC7A8	solute carrier family 7 member 8	-0.169
209529691	TMCO3	transmembrane and coiled-coil domains 3	-0.169
348041331	SERTAD2	SERTA domain containing 2	-0.169

149042395	PRDX4	peroxiredoxin 4	-0.169
149048094	N/A	N/A	-0.168
925114268	GTF3C1	general transcription factor IIIC subunit 1	-0.168
148668175	EDNRB	endothelin receptor type B	-0.168
62339281	ADAM9	ADAM metallopeptidase domain 9	-0.168
58219062	CNDP2	carnosine dipeptidase 2	-0.168
817472062	CDK5RAP3	CDK5 regulatory subunit associated protein 3	-0.167
564335900	ZFHX4	zinc finger homeobox 4	-0.167
186910267	LYRM2	LYR motif containing 2	-0.167
145279186	GAS6	growth arrest specific 6	-0.167
169234846	MBD2	methyl-CpG binding domain protein 2	-0.167
149040047	SYNPR	synaptoporin	-0.167
157822019	ITGB1BP1	integrin subunit beta 1 binding protein 1	-0.166
9506591	FDFT1	farnesyl-diphosphate farnesyltransferase 1	-0.166
568975977	STRADA	STE20-related kinase adaptor alpha	-0.166
19705545	RAB3IL1	RAB3A interacting protein like 1	-0.166
20127390	RNF112	ring finger protein 112	-0.166
62945358	SCFD2	sec1 family domain containing 2	-0.165
155369271	PRKACA	protein kinase cAMP-activated catalytic subunit alpha	-0.165
157817201	NETO1	neuropilin and tolloid like 1	-0.165
6981076	IDE	insulin degrading enzyme	-0.165
149062946	EPHB4	EPH receptor B4	-0.165
157817971	FAM13B	family with sequence similarity 13 member B	-0.165
60360580	OGDH	oxoglutarate dehydrogenase	-0.165
568992887	PRPF40B	pre-mRNA processing factor 40 homolog B	-0.165
149031018	COX4I2	cytochrome c oxidase subunit 4I2	-0.165
149046296	CREG2	cellular repressor of E1A stimulated genes 2	-0.165
13929002	PFKM	phosphofructokinase, muscle	-0.165
13242243	AXIN2	axin 2	-0.164
148747253	ATP1B1	ATPase Na+/K+ transporting subunit beta 1	-0.164
149023207	N/A	N/A	-0.164
19173766	LONP1	lon peptidase 1, mitochondrial	-0.164
79750129	CAMK1D	calcium/calmodulin dependent protein kinase ID	-0.164
149057384	C15orf40	chromosome 15 open reading frame 40	-0.163
62079137	CD320	CD320 molecule	-0.163
197245729	CPSF1	cleavage and polyadenylation specific factor 1	-0.163
564343351	RIN2	Ras and Rab interactor 2	-0.163
672088848	PLXNA3	plexin A3	-0.163

12018278	ILKAP	ILK associated serine/threonine phosphatase	-0.163
67846010	ROGDI	rogdi homolog	-0.163
403224993	ABHD11	abhydrolase domain containing 11	-0.163
6981542	SLC16A1	solute carrier family 16 member 1	-0.163
197384515	UAP1L1	UDP-N-acetylglucosamine pyrophosphorylase 1 like 1	-0.163
672075219	N/A	N/A	-0.162
635141087	N/A	N/A	-0.162
148687256	N/A	N/A	-0.162
300798541	MID2	midline 2	-0.162
124486885	LRRC7	leucine rich repeat containing 7	-0.162
346989661	CPEB2	cytoplasmic polyadenylation element binding protein 2	-0.162
56090387	CPTP	ceramide-1-phosphate transfer protein	-0.161
807677	N/A	N/A	-0.161
982250550	N/A	N/A	-0.161
556758981	N/A	N/A	-0.161
392344250	SPTY2D1	SPT2 chromatin protein domain containing 1	-0.161
109488292	POLR2A	RNA polymerase II subunit A	-0.161
149052635	TNIP1	TNFAIP3 interacting protein 1	-0.161
157820049	LRFN5	leucine rich repeat and fibronectin type III domain containing 5	-0.161
40786447	CFDP1	craniofacial development protein 1	-0.160
672063062	N/A	N/A	-0.160
76096352	ALDH16A1	aldehyde dehydrogenase 16 family member A1	-0.160
224967128	TBRG1	transforming growth factor beta regulator 1	-0.159
16758808	EPB41L3	erythrocyte membrane protein band 4.1 like 3	-0.159
157819829	HACD3	3-hydroxyacyl-CoA dehydratase 3	-0.159
149066619	B4GALNT1	beta-1,4-N-acetyl-galactosaminyltransferase 1	-0.159
158186708	PDCD11	programmed cell death 11	-0.159
198278547	TMEM41A	transmembrane protein 41A	-0.159
154146247	TMEM38A	transmembrane protein 38A	-0.159
401664552	MRPS7	mitochondrial ribosomal protein S7	-0.159
52345385	PDIA6	protein disulfide isomerase family A member 6	-0.159
56605830	CLBA1	clathrin binding box of aftiphilin containing 1	-0.158
686713740	N/A	N/A	-0.158
198442897	AFG3L2	AFG3 like matrix AAA peptidase subunit 2	-0.158
61556993	HIBCH	3-hydroxyisobutyryl-CoA hydrolase	-0.158
387157884	INO80	INO80 complex subunit	-0.158
62078637	LCA5	LCA5, lebercilin	-0.158

564397835	ASCC1	activating signal cointegrator 1 complex subunit 1	-0.158
148677354	CNOT10	CCR4-NOT transcription complex subunit 10	-0.158
524962788	N/A	N/A	-0.158
18777747	MGEA5	meningioma expressed antigen 5 (hyaluronidase)	-0.158
74201328	ST6GALNAC4	ST6 N-acetylgalactosaminide alpha-2,6-sialyltransferase 4	-0.158
209529636	PPA2	pyrophosphatase (inorganic) 2	-0.158
6981168	LPL	lipoprotein lipase	-0.158
18034785	ABCB6	ATP binding cassette subfamily B member 6 (Langereis blood group)	-0.158
672033256	LOC100912904	disks large homolog 5-like	-0.157
537272666	N/A	N/A	-0.157
149634159	N/A	N/A	-0.157
56972416	IFI30	IFI30, lysosomal thiol reductase	-0.157
149016230	ACSL3	acyl-CoA synthetase long chain family member 3	-0.157
194474054	EFR3A	EFR3 homolog A	-0.157
62078609	Dync2li1	dynein cytoplasmic 2 light intermediate chain 1	-0.157
148677282	N/A	N/A	-0.157
672022833	Scaper	S-phase cyclin A-associated protein in the ER	-0.157
961763390	N/A	N/A	-0.156
48675845	ATIC	5-aminoimidazole-4-carboxamide ribonucleotide formyltransferase/IMP cyclohydrolase	-0.156
12838537	C19orf81	chromosome 19 open reading frame 81	-0.156
148668476	N/A	N/A	-0.156
564299821	PARP8	poly(ADP-ribose) polymerase family member 8	-0.155
13928730	SCARB1	scavenger receptor class B member 1	-0.155
19173802	PPP1R14C	protein phosphatase 1 regulatory inhibitor subunit 14C	-0.155
564376241	N/A	N/A	-0.155
18266726	PAICS	phosphoribosylaminoimidazole carboxylase and phosphoribosylaminoimidazolesuccinocarboxamide synthase	-0.155
109639149	Lonp2	lon peptidase 2, peroxisomal	-0.154
149026580	N/A	N/A	-0.154
6649914	GDF11	growth differentiation factor 11	-0.154
158534079	CHRNA5	cholinergic receptor nicotinic alpha 5 subunit	-0.154

564385664	FERMT2	fermitin family member 2	-0.154
564324344	LOC363306 (includes others)	hypothetical protein LOC363306	-0.154
6978765	DLX5	distal-less homeobox 5	-0.154
55741778	SMPD1	sphingomyelin phosphodiesterase 1	-0.153
157817678	BUB1	BUB1 mitotic checkpoint serine/threonine kinase	-0.153
158636004	FLOT1	flotillin 1	-0.153
195976798	SLBP	stem-loop binding protein	-0.153
532100790	N/A	N/A	-0.153
521031426	N/A	N/A	-0.153
8394496	TYRO3	TYRO3 protein tyrosine kinase	-0.152
149020656	MRE11	MRE11 homolog, double strand break repair nuclease	-0.152
149045578	N/A	N/A	-0.152
403225003	KCNQ5	potassium voltage-gated channel subfamily Q member 5	-0.152
664703871	N/A	N/A	-0.151
564388617	KXD1	KxDL motif containing 1	-0.150
564370968	MAPK8IP3	mitogen-activated protein kinase 8 interacting protein 3	-0.150
149024719	NOL9	nucleolar protein 9	-0.150
61556967	EEF1D	eukaryotic translation elongation factor 1 delta	-0.150
953875962	N/A	N/A	-0.150
149032539	HECW1	HECT, C2 and WW domain containing E3 ubiquitin protein ligase 1	-0.150
71043650	SRPK1	SRSF protein kinase 1	-0.150
118150676	CCNA2	cyclin A2	-0.149
672046065	N/A	N/A	-0.149
62078573	C20orf24	chromosome 20 open reading frame 24	-0.149
76880459	THOC6	THO complex 6	-0.149
537139221	N/A	N/A	-0.149
755498773	ITGA6	integrin subunit alpha 6	-0.149
41281619	PCDHA10	protocadherin alpha 10	-0.149
157819977	CERS4	ceramide synthase 4	-0.149
672041603	N/A	N/A	-0.149
113461996	COA5	cytochrome c oxidase assembly factor 5	-0.149
880936540	N/A	N/A	-0.149
50510837	KIAA1191	KIAA1191	-0.148
8394227	PTPRO	protein tyrosine phosphatase, receptor type O	-0.148

109505096	NID1	nidogen 1	-0.148
564388440	MYO9B	myosin IXB	-0.148
926693529	N/A	N/A	-0.148
157821569	ASH2L	ASH2 like histone lysine methyltransferase complex subunit	-0.148
84781723	TRAP1	TNF receptor associated protein 1	-0.148
544434017	N/A	N/A	-0.148
717324516	SCN8A	sodium voltage-gated channel alpha subunit 8	-0.147
672016955	MAP3K20	mitogen-activated protein kinase kinase kinase 20	-0.147
564393851	N/A	N/A	-0.147
564390319	GKAP1	G kinase anchoring protein 1	-0.147
300794996	NDST3	N-deacetylase and N-sulfotransferase 3	-0.147
48976085	GM2A	GM2 ganglioside activator	-0.147
755553984	THAP7	THAP domain containing 7	-0.147
564364792	SNAP91	synaptosome associated protein 91	-0.147
157818033	ATG4C	autophagy related 4C cysteine peptidase	-0.146
6980978	GPD2	glycerol-3-phosphate dehydrogenase 2	-0.146
157820591	AVEN	apoptosis and caspase activation inhibitor	-0.146
55742755	CTNNA1	catenin alpha 1	-0.146
40254752	PGK1	phosphoglycerate kinase 1	-0.146
6980956	GLUD1	glutamate dehydrogenase 1	-0.146
149064661	PLCH1	phospholipase C eta 1	-0.146
564311572	N/A	N/A	-0.145
564321893	ICOSLG/LOC102723996	inducible T cell costimulator ligand	-0.145
148703734	MID1IP1	MID1 interacting protein 1	-0.145
71043702	TM9SF4	transmembrane 9 superfamily member 4	-0.145
8392888	AKT2	AKT serine/threonine kinase 2	-0.145
157820565	HSCB	HscB mitochondrial iron-sulfur cluster cochaperone	-0.145
569006964	C9orf40	chromosome 9 open reading frame 40	-0.145
157820561	NAT10	N-acetyltransferase 10	-0.144
19173762	NAP1L3	nucleosome assembly protein 1 like 3	-0.144
564347453	ALMS1	ALMS1, centrosome and basal body associated protein	-0.144
984094253	N/A	N/A	-0.144
148672025	MAP3K12	mitogen-activated protein kinase kinase kinase 12	-0.144

281485631	NAA38	N(alpha)-acetyltransferase 38, NatC auxiliary subunit	-0.143
283837900	FOXH1	forkhead box H1	-0.143
148671058	N/A	N/A	-0.143
288806592	PRKCB	protein kinase C beta	-0.143
109472884	UBE3C	ubiquitin protein ligase E3C	-0.142
157823986	MAN1A2	mannosidase alpha class 1A member 2	-0.142
730229363	RALGAPA1	Ral GTPase activating protein catalytic alpha subunit 1	-0.142
71122474	PPA1	pyrophosphatase (inorganic) 1	-0.142
564334920	HMGCR	3-hydroxy-3-methylglutaryl-CoA reductase	-0.142
60223057	SUPV3L1	Suv3 like RNA helicase	-0.142
564383481	SRD5A3	steroid 5 alpha-reductase 3	-0.142
6756037	YWHAH	tyrosine 3-monooxygenase/tryptophan 5-monooxygenase activation protein eta	-0.142
157822025	NDOR1	NADPH dependent diflavin oxidoreductase 1	-0.142
148671875	TMEM50B	transmembrane protein 50B	-0.142
16905129	NECAB2	N-terminal EF-hand calcium binding protein 2	-0.141
56090241	THTPA	thiamine triphosphatase	-0.141
26328955	ETNK1	ethanolamine kinase 1	-0.141
300793996	TCHP	trichoplein keratin filament binding	-0.141
149065077	FAM3C	family with sequence similarity 3 member C	-0.141
984090530	N/A	N/A	-0.141
411147403	APLP2	amyloid beta precursor like protein 2	-0.141
148692881	OXNAD1	oxidoreductase NAD binding domain containing 1	-0.140
157822919	GANAB	glucosidase II alpha subunit	-0.140
66730507	CCDC134	coiled-coil domain containing 134	-0.140
281306811	ATP4A	ATPase H+/K+ transporting alpha subunit	-0.140
189027115	AIDA	axin interactor, dorsalization associated	-0.140
9507043	RGS12	regulator of G protein signaling 12	-0.139
16758736	NLGN1	neuroligin 1	-0.139
201066369	LGI2	leucine rich repeat LGI family member 2	-0.139
8393450	GLG1	golgi glycoprotein 1	-0.139
51948478	FARSB	phenylalanyl-tRNA synthetase beta subunit	-0.138
6978751	CYP51A1	cytochrome P450 family 51 subfamily A member 1	-0.138
444728185	N/A	N/A	-0.138
77627979	SRPRA	SRP receptor alpha subunit	-0.138
148665211	N/A	N/A	-0.137

564389848	ERICH1	glutamate rich 1	-0.137
403044506	SUPT5H	SPT5 homolog, DSIF elongation factor subunit	-0.137
68163557	CDCA8	cell division cycle associated 8	-0.136
281485565	RASGRF1	Ras protein specific guanine nucleotide releasing factor 1	-0.136
157820105	UVRAG	UV radiation resistance associated	-0.136
674044244	N/A	N/A	-0.136
25742686	ELOVL6	ELOVL fatty acid elongase 6	-0.136
14091777	Chn1	chimerin 1	-0.136
189083764	MARS	methionyl-tRNA synthetase	-0.136
564317187	N/A	N/A	-0.136
205640	CHRNA4	cholinergic receptor nicotinic alpha 4 subunit	-0.136
197384309	SLC39A1	solute carrier family 39 member 1	-0.136
62079259	CEP83	centrosomal protein 83	-0.135
612030228	N/A	N/A	-0.135
672044529	MIGA1	mitoguardin 1	-0.135
157823401	PIGH	phosphatidylinositol glycan anchor biosynthesis class H	-0.135
12018252	TKT	transketolase	-0.134
690969208	MCC	mutated in colorectal cancers	-0.134
564338482	SORT1	sortilin 1	-0.134
53850598	DDX59	DEAD-box helicase 59	-0.134
157823181	SKP2	S-phase kinase associated protein 2	-0.134
157817708	PPP6R2	protein phosphatase 6 regulatory subunit 2	-0.134
40288195	GABRG3	gamma-aminobutyric acid type A receptor gamma3 subunit	-0.134
537141312	N/A	N/A	-0.134
148693879	WDR61	WD repeat domain 61	-0.133
760996272	EHBP1	EH domain binding protein 1	-0.133
72384369	KIF1BP	KIF1 binding protein	-0.133
31542604	ERC1	ELKS/RAB6-interacting/CAST family member 1	-0.133
6981166	PLAGL1	PLAG1 like zinc finger 1	-0.133
300794317	SFI1	SFI1 centrin binding protein	-0.133
674075764	N/A	N/A	-0.133
672080550	TDRP	testis development related protein	-0.133
62078691	Lrwd1	leucine-rich repeats and WD repeat domain containing 1	-0.132
564345487	RINT1	RAD50 interactor 1	-0.132
157817604	PIK3R4	phosphoinositide-3-kinase regulatory subunit 4	-0.132

402534517	EPB41L1	erythrocyte membrane protein band 4.1 like 1	-0.132
149015561	N/A	N/A	-0.132
71896602	NEU1	neuraminidase 1	-0.132
20302117	FNBP1	formin binding protein 1	-0.132
725548711	N/A	N/A	-0.132
50510357	GPD1L	glycerol-3-phosphate dehydrogenase 1 like	-0.132
62078739	TCTA	T cell leukemia translocation altered	-0.131
35215304	CLMP	CXADR like membrane protein	-0.131
62078667	SLC46A1	solute carrier family 46 member 1	-0.131
56605798	RNF167	ring finger protein 167	-0.131
880928207	N/A	N/A	-0.130
148747541	HNRNPU	heterogeneous nuclear ribonucleoprotein U	-0.130
9507177	USO1	USO1 vesicle transport factor	-0.130
8394354	SQLE	squalene epoxidase	-0.129
144445950	XRCC6	X-ray repair cross complementing 6	-0.129
564351227	E130308A19Rik	RIKEN cDNA E130308A19 gene	-0.129
472381467	N/A	N/A	-0.129
568985444	CADPS	calcium dependent secretion activator	-0.129
158749632	DBT	dihydrolipoamide branched chain transacylase E2	-0.129
56090363	TEX264	testis expressed 264	-0.129
149019723	PPIL2	peptidylprolyl isomerase like 2	-0.129
66730427	CENPT	centromere protein T	-0.129
672048439	CABLES2	Cdk5 and Abl enzyme substrate 2	-0.128
189181730	PRKD3	protein kinase D3	-0.128
6981520	SDC2	syndecan 2	-0.128
158711729	HACE1	HECT domain and ankyrin repeat containing E3 ubiquitin protein ligase 1	-0.128
19924067	NME7	NME/NM23 family member 7	-0.128
398650648	SLC8A1	solute carrier family 8 member A1	-0.127
226874873	GUK1	guanylate kinase 1	-0.127
672071181	N/A	N/A	-0.127
21553105	AIF1L	allograft inflammatory factor 1 like	-0.127
37359832	SCRN1	secernin 1	-0.127
55742813	BDH1	3-hydroxybutyrate dehydrogenase 1	-0.127
163659911	GRIK1	glutamate ionotropic receptor kainate type subunit 1	-0.126
740086795	POLR2B	RNA polymerase II subunit B	-0.126
149035673	FAF1	Fas associated factor 1	-0.126
114052913	CADM2	cell adhesion molecule 2	-0.126

564382183	EPRS	glutamyl-prolyl-tRNA synthetase	-0.126
55926219	DDX39A	DExD-box helicase 39A	-0.126
77415383	HSPA8	heat shock protein family A (Hsp70) member 8	-0.125
564398462	Slc9a7	solute carrier family 9 member A7	-0.125
149053938	SLC35B1	solute carrier family 35 member B1	-0.125
357527369	PRMT8	protein arginine methyltransferase 8	-0.124
672059390	N/A	N/A	-0.124
399498531	NDRG2	NDRG family member 2	-0.123
564339312	FUBP1	far upstream element binding protein 1	-0.123
58865700	GRWD1	glutamate rich WD repeat containing 1	-0.123
157820919	POLE4	DNA polymerase epsilon 4, accessory subunit	-0.123
281604211	RAB3GAP2	RAB3 GTPase activating non-catalytic protein subunit 2	-0.123
149064207	COMMD10	COMM domain containing 10	-0.123
149040761	TAGLN2	transgelin 2	-0.122
672061192	ANLN	anillin actin binding protein	-0.122
51980666	CHAC2	ChaC cation transport regulator homolog 2	-0.122
564323194	Col4a5	collagen type IV alpha 5 chain	-0.122
28269685	HOPX	HOP homeobox	-0.122
157817043	ACOT13	acyl-CoA thioesterase 13	-0.122
564308814	N/A	N/A	-0.122
564301782	CERS6	ceramide synthase 6	-0.122
564326713	ZC3H4	zinc finger CCCH-type containing 4	-0.121
149024626	EXOSC10	exosome component 10	-0.121
166158339	REEP3	receptor accessory protein 3	-0.121
157822423	LRSAM1	leucine rich repeat and sterile alpha motif containing 1	-0.121
157820617	TUBGCP2	tubulin gamma complex associated protein 2	-0.121
253683488	NTRK2	neurotrophic receptor tyrosine kinase 2	-0.121
157817346	ATP9B	ATPase phospholipid transporting 9B (putative)	-0.121
158254369	CDK10	cyclin dependent kinase 10	-0.121
74229032	TPCN1	two pore segment channel 1	-0.121
401709975	GPR176	G protein-coupled receptor 176	-0.121
209870013	ITSN1	intersectin 1	-0.121
157822001	RACGAP1	Rac GTPase activating protein 1	-0.120
274325671	SCAMP3	secretory carrier membrane protein 3	-0.119
51948426	RTCA	RNA 3'-terminal phosphate cyclase	-0.119
568907121	N/A	N/A	-0.119
149043706	PRMT2	protein arginine methyltransferase 2	-0.119
197387125	CCSER1	coiled-coil serine rich protein 1	-0.118

672041232	PDE8B	phosphodiesterase 8B	-0.118
27229314	FIBP	FGF1 intracellular binding protein	-0.118
149047238	ADGRA3	adhesion G protein-coupled receptor A3	-0.118
585716513	N/A	N/A	-0.118
71896549	UTP14A	UTP14A, small subunit processome component	-0.118
537146709	N/A	N/A	-0.118
532074114	N/A	N/A	-0.118
28212228	RCAN2	regulator of calcineurin 2	-0.118
564385256	N/A	N/A	-0.117
157820497	DDX51	DEAD-box helicase 51	-0.117
476007242	EPS8	epidermal growth factor receptor pathway substrate 8	-0.117
398303839	SH3GL2	SH3 domain containing GRB2 like 2, endophilin A1	-0.117
953870558	N/A	N/A	-0.116
293339963	RAB11FIP3	RAB11 family interacting protein 3	-0.116
852792963	N/A	N/A	-0.116
201066352	ANKRD6	ankyrin repeat domain 6	-0.116
564347854	GRIP2	glutamate receptor interacting protein 2	-0.116
57012426	HLA-A	major histocompatibility complex, class I, A	-0.115
157823994	PNMA8A	PNMA family member 8A	-0.115
817280433	N/A	N/A	-0.115
8393390	GABRB3	gamma-aminobutyric acid type A receptor beta3 subunit	-0.115
52138635	ETFDH	electron transfer flavoprotein dehydrogenase	-0.115
56605990	LRPPRC	leucine rich pentatricopeptide repeat containing	-0.115
29789269	GRIA1	glutamate ionotropic receptor AMPA type subunit 1	-0.115
67078458	CRELD1	cysteine rich with EGF like domains 1	-0.115
149059529	LOC100910558	uncharacterized LOC100910558	-0.114
399124797	KIFC2	kinesin family member C2	-0.114
11560079	KIT	KIT proto-oncogene receptor tyrosine kinase	-0.114
6978621	CCNG1	cyclin G1	-0.114
672019106	ASPH	aspartate beta-hydroxylase	-0.114
56912225	CIAO1	cytosolic iron-sulfur assembly component 1	-0.114
157823175	ZC3H7A	zinc finger CCCH-type containing 7A	-0.114
78126149	SDF4	stromal cell derived factor 4	-0.113
149061951	CORO1B	coronin 1B	-0.113
404247435	YLPM1	YLP motif containing 1	-0.113
157823591	MTMR14	myotubularin related protein 14	-0.113

60360272	KLHL5	kelch like family member 5	-0.113
68163527	HMCES	5-hydroxymethylcytosine (hmC) binding, ES cell-specific	-0.113
931599326	N/A	N/A	-0.113
160333166	Pid1	phosphotyrosine interaction domain containing 1	-0.113
56090463	GORASP2	golgi reassembly stacking protein 2	-0.113
148698492	RIMKLA	ribosomal modification protein rimK like family member A	-0.112
149030275	PRSS55	protease, serine 55	-0.112
149031313	N/A	N/A	-0.112
431895094	N/A	N/A	-0.112
564353678	USP48	ubiquitin specific peptidase 48	-0.112
149065950	C22orf23	chromosome 22 open reading frame 23	-0.111
149064946	PON2	paraoxonase 2	-0.111
8393038	CAPN2	calpain 2	-0.111
469608396	DUSP18	dual specificity phosphatase 18	-0.111
672046063	Slc25a12	solute carrier family 25 member 12	-0.111
330340424	TMTC2	transmembrane and tetratricopeptide repeat containing 2	-0.111
148673922	HSPH1	heat shock protein family H (Hsp110) member 1	-0.110
300794591	FXN	frataxin	-0.110
149018872	N/A	N/A	-0.110
8394331	SOD2	superoxide dismutase 2	-0.110
564355419	Nbas	neuroblastoma amplified sequence	-0.110
349501010	FAM120B	family with sequence similarity 120B	-0.110
157786602	NHP2	NHP2 ribonucleoprotein	-0.110
157823249	FAM81A	family with sequence similarity 81 member A	-0.109
281352199	N/A	N/A	-0.109
524960503	N/A	N/A	-0.108
655863889	N/A	N/A	-0.108
57164133	NDUFC2	NADH:ubiquinone oxidoreductase subunit C2	-0.108
694981804	CLDN5	claudin 5	-0.108
37360274	N/A	N/A	-0.108
51948408	FUCA2	alpha-L-fucosidase 2	-0.107
89337260	FTO	FTO, alpha-ketoglutarate dependent dioxygenase	-0.107
148673176	FABP7	fatty acid binding protein 7	-0.107

81884516	Rhno1	RAD9-HUS1-RAD1 interacting nuclear orphan 1	-0.107
184160976	PRMT5	protein arginine methyltransferase 5	-0.107
61556863	BTBD9	BTB domain containing 9	-0.106
114326232	GPAT4	glycerol-3-phosphate acyltransferase 4	-0.106
564311487	LONRF2	LON peptidase N-terminal domain and ring finger 2	-0.106
274326692	UQCC3	ubiquinol-cytochrome c reductase complex assembly factor 3	-0.106
149040289	C10orf76	chromosome 10 open reading frame 76	-0.106
58865384	NDUFS2	NADH:ubiquinone oxidoreductase core subunit S2	-0.106
19424310	SLC31A1	solute carrier family 31 member 1	-0.106
672066454	Mkl2	MKL1/myocardin like 2	-0.106
564389912	N/A	N/A	-0.106
149031313	N/A	N/A	-0.105
512971254	N/A	N/A	-0.105
148691063	CCNY	cyclin Y	-0.105
149066342	N/A	N/A	-0.105
795140840	N/A	N/A	-0.105
157816887	LOC500028/Yae1d1	Yae1 domain containing 1	-0.105
880971033	N/A	N/A	-0.104
164663858	VAV2	vav guanine nucleotide exchange factor 2	-0.104
672065125	ADAM23	ADAM metallopeptidase domain 23	-0.104
298231200	INSR	insulin receptor	-0.104
157822757	MAP3K4	mitogen-activated protein kinase kinase kinase 4	-0.104
55249662	Masp1	mannan-binding lectin serine peptidase 1	-0.104
282158057	ASTN1	astrotactin 1	-0.103
341823648	RAPH1	Ras association (RalGDS/AF-6) and pleckstrin homology domains 1	-0.103
148707802	DARS	aspartyl-tRNA synthetase	-0.103
13928818	PTPRN2	protein tyrosine phosphatase, receptor type N2	-0.102
282154799	NFU1	NFU1 iron-sulfur cluster scaffold	-0.102
9665227	DLG4	discs large MAGUK scaffold protein 4	-0.101
158186625	FGFR4	fibroblast growth factor receptor 4	-0.101
164519053	FAM131B	family with sequence similarity 131 member B	-0.101
564368924	STK16	serine/threonine kinase 16	-0.101
67846036	DNPEP	aspartyl aminopeptidase	-0.101

55741823	TARS	threonyl-tRNA synthetase	-0.101
674095102	N/A	N/A	-0.101
16758210	NUCB1	nucleobindin 1	-0.101
157821419	C14orf37	chromosome 14 open reading frame 37	-0.101
281306763	NTN3	netrin 3	-0.100
10946604	SEC61A2	Sec61 translocon alpha 2 subunit	-0.100
58865936	SIKE1	suppressor of IKBKE 1	-0.100
157818589	TSPAN7	tetraspanin 7	-0.100
161760632	ACLY	ATP citrate lyase	-0.100
300794843	IQGAP3	IQ motif containing GTPase activating protein 3	-0.100
625187436	N/A	N/A	-0.099
392353562	ATP8A2	ATPase phospholipid transporting 8A2	-0.099
672068312	N/A	N/A	-0.099
157821685	ABL2	ABL proto-oncogene 2, non-receptor tyrosine kinase	-0.099
157819175	Gpr165	G protein-coupled receptor 165	-0.099
149036808	ARL6IP5	ADP ribosylation factor like GTPase 6 interacting protein 5	-0.099
61889092	AK1	adenylate kinase 1	-0.099
55741628	EIF2B5	eukaryotic translation initiation factor 2B subunit epsilon	-0.099
403377905	SRGAP2	SLIT-ROBO Rho GTPase activating protein 2	-0.098
224967068	PLCB1	phospholipase C beta 1	-0.098
149059350	Plpp1	phospholipid phosphatase 1	-0.098
25282395	GSTM3	glutathione S-transferase mu 3	-0.098
672069439	N/A	N/A	-0.098
77812688	ZNF384	zinc finger protein 384	-0.098
38649320	ENO1	enolase 1	-0.097
157822707	PCDH20	protocadherin 20	-0.097
744603535	N/A	N/A	-0.097
564355613	HBP1	HMG-box transcription factor 1	-0.097
157786742	HIC2	HIC ZBTB transcriptional repressor 2	-0.097
71043910	SMYD3	SET and MYND domain containing 3	-0.097
120586971	MAGEE1	MAGE family member E1	-0.097
58865778	DDOST	dolichyl-diphosphooligosaccharide--protein glycosyltransferase non-catalytic subunit	-0.096
40254595	DPYSL2	dihydropyrimidinase like 2	-0.096
281427192	CDH11	cadherin 11	-0.096

253970439	KCNA6	potassium voltage-gated channel subfamily A member 6	-0.096
41152510	PLPPR1	phospholipid phosphatase related 1	-0.096
672057488	CD63	CD63 molecule	-0.096
537169371	N/A	N/A	-0.095
734703982	SAFB2	scaffold attachment factor B2	-0.095
149060725	CEP19	centrosomal protein 19	-0.095
197927388	GART	phosphoribosylglycinamide formyltransferase, phosphoribosylglycinamide synthetase, phosphoribosylaminoimidazole synthetase	-0.094
748585198	DLGAP1	DLG associated protein 1	-0.094
9507157	STRN	striatin	-0.094
401461792	GOT1	glutamic-oxaloacetic transaminase 1	-0.094
50402124	GSR	glutathione-disulfide reductase	-0.093
564349386	N/A	N/A	-0.093
154800420	GNL3L	G protein nucleolar 3 like	-0.093
148703191	HSPA4L	heat shock protein family A (Hsp70) member 4 like	-0.093
46485440	GPI	glucose-6-phosphate isomerase	-0.093
157819101	MRPS23	mitochondrial ribosomal protein S23	-0.092
149024815	N/A	N/A	-0.092
148700731	ELMO1	engulfment and cell motility 1	-0.092
71682358	HIPK3	homeodomain interacting protein kinase 3	-0.092
40363268	WASHC2A/WASHC2C	WASH complex subunit 2A	-0.092
157820113	RANBP1	RAN binding protein 1	-0.092
148669961	SLC6A17	solute carrier family 6 member 17	-0.091
829972711	N/A	N/A	-0.091
300797435	RNF123	ring finger protein 123	-0.091
148690630	N/A	N/A	-0.090
224549858	Poldip2	DNA polymerase delta interacting protein 2	-0.090
148682688	CDK14	cyclin dependent kinase 14	-0.090
403420618	NUMA1	nuclear mitotic apparatus protein 1	-0.090
274326531	HSF1	heat shock transcription factor 1	-0.090
166201905	DIS3L	DIS3 like exosome 3'-5' exoribonuclease	-0.089
672068112	N/A	N/A	-0.089
672089019	N/A	N/A	-0.089
6978465	GRK2	G protein-coupled receptor kinase 2	-0.089
166235165	SYP	synaptophysin	-0.089
16258813	VHL	von Hippel-Lindau tumor suppressor	-0.089

8980843	GRIPAP1	GRIP1 associated protein 1	-0.089
149069421	2310039H08Rik	RIKEN cDNA 2310039H08 gene	-0.089
18093100	PLCD4	phospholipase C delta 4	-0.089
564346506	AGK	acylglycerol kinase	-0.088
6981370	PLCG1	phospholipase C gamma 1	-0.088
564350262	CHD7	chromodomain helicase DNA binding protein 7	-0.088
83649693	CHST12	carbohydrate sulfotransferase 12	-0.088
530362302	KPNA6	karyopherin subunit alpha 6	-0.087
58865950	LPCAT3	lysophosphatidylcholine acyltransferase 3	-0.087
308153222	ARHGEF39	Rho guanine nucleotide exchange factor 39	-0.086
157823007	EAF1	ELL associated factor 1	-0.086
12408298	DPP6	dipeptidyl peptidase like 6	-0.086
564370751	HCFC1R1	host cell factor C1 regulator 1	-0.086
157821255	RFX5	regulatory factor X5	-0.086
635071473	N/A	N/A	-0.085
926717140	N/A	N/A	-0.085
521032446	N/A	N/A	-0.085
89111939	EXOC1	exocyst complex component 1	-0.085
157822747	CLVS1	clavesin 1	-0.085
300798083	RAVER2	ribonucleoprotein, PTB binding 2	-0.084
926708692	N/A	N/A	-0.084
195927000	DLST	dihydrolipoamide S-succinyltransferase	-0.084
16758578	DPP3	dipeptidyl peptidase 3	-0.084
157821365	IFFO1	intermediate filament family orphan 1	-0.084
149022387	NCKAP1	NCK associated protein 1	-0.084
564314041	TMEM44	transmembrane protein 44	-0.084
568916417	N/A	N/A	-0.083
564375628	Foxk2	forkhead box K2	-0.082
46485379	RTCB	RNA 2',3'-cyclic phosphate and 5'-OH ligase	-0.082
55249691	CPE	carboxypeptidase E	-0.082
83404987	FTH1	ferritin heavy chain 1	-0.082
50054266	NLN	neurolysin	-0.082
586464862	N/A	N/A	-0.081
672054308	N/A	N/A	-0.081
12083661	ARL3	ADP ribosylation factor like GTPase 3	-0.081
220777	STX1A	syntaxin 1A	-0.080
54035294	ADH5	alcohol dehydrogenase 5 (class III), chi polypeptide	-0.080
926710268	N/A	N/A	-0.080
913498484	N/A	N/A	-0.080

672054406	N/A	N/A	-0.079
564380332	R3HDM1	R3H domain containing 1	-0.079
18034789	Phkg2	phosphorylase kinase catalytic subunit gamma 2	-0.079
148747375	CDS1	CDP-diacylglycerol synthase 1	-0.078
157818189	CIDEB	cell death-inducing DFFA-like effector b	-0.078
564354392	NADK	NAD kinase	-0.078
568906429	INPP4A	inositol polyphosphate-4-phosphatase type I A	-0.077
625205142	N/A	N/A	-0.077
149060735	SENP5	SUMO1/sentrin specific peptidase 5	-0.077
390979616	ITGA3	integrin subunit alpha 3	-0.077
25742586	VPS33B	VPS33B, late endosome and lysosome associated	-0.076
568963507	MAP4	microtubule associated protein 4	-0.076
564343911	RPN2	ribophorin II	-0.076
568986834	KCNMA1	potassium calcium-activated channel subfamily M alpha 1	-0.075
392346010	DNTTIP2	deoxynucleotidyltransferase terminal interacting protein 2	-0.075
57528205	C12orf43	chromosome 12 open reading frame 43	-0.075
297206894	E4F1	E4F transcription factor 1	-0.074
672061806	N/A	N/A	-0.073
13928690	IDH1	isocitrate dehydrogenase (NADP(+)) 1, cytosolic	-0.072
81870010	WIPF3	WAS/WASL interacting protein family member 3	-0.072
8394272	NUDC	nuclear distribution C, dynein complex regulator	-0.072
157817492	PAK6	p21 (RAC1) activated kinase 6	-0.072
189011661	PELI3	pellino E3 ubiquitin protein ligase family member 3	-0.071
34536836	EHD3	EH domain containing 3	-0.071
11559994	PIK3R3	phosphoinositide-3-kinase regulatory subunit 3	-0.070
564375060	SLC39A11	solute carrier family 39 member 11	-0.070
826287877	N/A	N/A	-0.070
564374948	MRC2	mannose receptor C type 2	-0.070
537137046	N/A	N/A	-0.069
261337192	DDX23	DEAD-box helicase 23	-0.069
62657153	EFTUD2	elongation factor Tu GTP binding domain containing 2	-0.068

672055279	PREPL	prolyl endopeptidase like	-0.068
188595689	STK24	serine/threonine kinase 24	-0.067
12083643	Exoc7	exocyst complex component 7	-0.067
67846018	CTNNBL1	catenin beta like 1	-0.066
157816897	UHRF1BP1L	UHRF1 binding protein 1 like	-0.066
392339359	LDLRAD3	low density lipoprotein receptor class A domain containing 3	-0.066
13786182	FADS2	fatty acid desaturase 2	-0.066
148747528	PTK2B	protein tyrosine kinase 2 beta	-0.066
52138624	SLC25A20	solute carrier family 25 member 20	-0.066
38454252	MRPS18A	mitochondrial ribosomal protein S18A	-0.066
157818729	SPATA33	spermatogenesis associated 33	-0.065
83649771	Polr1d	RNA polymerase I subunit D	-0.065
9507099	ST6GALNAC3	ST6 N-acetylgalactosaminide alpha-2,6-sialyltransferase 3	-0.065
564328458	Ldha/RGD1562690	lactate dehydrogenase A	-0.064
564395373	TMEM184C	transmembrane protein 184C	-0.064
255918181	NUS1	NUS1 dehydrodolichyl diphosphate synthase subunit	-0.062
215274190	PPIB	peptidylprolyl isomerase B	-0.062
60360518	KIF5A	kinesin family member 5A	-0.062
40786465	YIPF1	Yip1 domain family member 1	-0.061
149030689	N/A	N/A	-0.061
392352768	PAPPA2	pappalysin 2	-0.060
300794891	DDX20	DEAD-box helicase 20	-0.060
77020248	PFKFB2	6-phosphofructo-2-kinase/fructose-2,6-biphosphatase 2	-0.060
20302113	STIP1	stress induced phosphoprotein 1	-0.060
149017294	Pcdhb7	protocadherin beta 7	-0.060
58865442	BTBD1	BTB domain containing 1	-0.060
186910247	MRPS21	mitochondrial ribosomal protein S21	-0.059
149040371	XPNPEP1	X-prolyl aminopeptidase 1	-0.059
29789275	PDGFRB	platelet derived growth factor receptor beta	-0.059
161016776	ATP2A2	ATPase sarcoplasmic/endoplasmic reticulum Ca2+ transporting 2	-0.059
18543177	CS	citrate synthase	-0.059
213688386	GTF2E1	general transcription factor IIE subunit 1	-0.058
157821073	IPO9	importin 9	-0.058
408535187	PRDM11	PR/SET domain 11	-0.058
28212260	SSX2IP	SSX family member 2 interacting protein	-0.057

13385318	KDELR2	KDEL endoplasmic reticulum protein retention receptor 2	-0.057
149049470	TPI1	triosephosphate isomerase 1	-0.057
157817861	NDUFA2	NADH:ubiquinone oxidoreductase subunit A2	-0.057
198278575	BRCC3	BRCA1/BRCA2-containing complex subunit 3	-0.056
13027436	APAF1	apoptotic peptidase activating factor 1	-0.056
300794036	TMEM185B	transmembrane protein 185B	-0.056
157822951	SPIRE1	spire type actin nucleation factor 1	-0.055
947288638	N/A	N/A	-0.055
188536098	SLC48A1	solute carrier family 48 member 1	-0.055
13928838	TMOD2	tropomodulin 2	-0.054
672065682	AGAP1	ArfGAP with GTPase domain, ankyrin repeat and PH domain 1	-0.053
148696230	SNRNP200	small nuclear ribonucleoprotein U5 subunit 200	-0.053
109460217	NPM3	nucleophosmin/nucleoplasmin 3	-0.052
77627990	ATP6V0A1	ATPase H+ transporting V0 subunit a1	-0.052
149065158	AHCYL2	adenosylhomocysteinase like 2	-0.052
71795619	SLC19A2	solute carrier family 19 member 2	-0.052
83320121	RBM8A	RNA binding motif protein 8A	-0.052
67078460	PRKRA	protein activator of interferon induced protein kinase EIF2AK2	-0.051
162287208	FADS1	fatty acid desaturase 1	-0.050
9790083	RUVBL1	RuvB like AAA ATPase 1	-0.050
60678266	ENPP5	ectonucleotide pyrophosphatase/phosphodiesterase 5 (putative)	-0.049
16758168	FGF13	fibroblast growth factor 13	-0.049
880956999	N/A	N/A	-0.049
18426824	KHDRBS1	KH RNA binding domain containing, signal transduction associated 1	-0.047
402743461	DPY19L3	dpy-19 like C-mannosyltransferase 3	-0.046
77861906	PLPP6	phospholipid phosphatase 6	-0.045
953713397	AGO1	argonaute 1, RISC catalytic component	-0.045
157821319	ATP6V0B	ATPase H+ transporting V0 subunit b	-0.045
189027133	TTC30B	tetratricopeptide repeat domain 30B	-0.044
157823867	TLL1	tolloid like 1	-0.044
8394115	PTS	6-pyruvoyltetrahydropterin synthase	-0.044
947289938	N/A	N/A	-0.044
157820373	ELK1	ELK1, ETS transcription factor	-0.043

20809990	XPA	XPA, DNA damage recognition and repair factor	-0.043
672048779	N/A	N/A	-0.043
564359598	OS9	OS9, endoplasmic reticulum lectin	-0.043
28189917	Ubb	ubiquitin B	-0.043
149045755	CREB3	cAMP responsive element binding protein 3	-0.043
24638208	EXOC2	exocyst complex component 2	-0.041
20302061	ATP5PO	ATP synthase peripheral stalk subunit OSCP	-0.040
82830420	CTSB	cathepsin B	-0.040
148692627	N/A	N/A	-0.039
564391118	SLC22A23	solute carrier family 22 member 23	-0.039
281332197	Akr1c12/Akr1c13	aldo-keto reductase family 1, member C13	-0.037
18426858	SDHA	succinate dehydrogenase complex flavoprotein subunit A	-0.037
62079109	LANCL2	LanC like 2	-0.034
655862992	N/A	N/A	-0.033
537269232	N/A	N/A	-0.033
157818471	PPM1L	protein phosphatase, Mg2+/Mn2+ dependent 1L	-0.032
157819901	STAM	signal transducing adaptor molecule	-0.031
124430496	PRKAR1B	protein kinase cAMP-dependent type I regulatory subunit beta	-0.027
183986109	USP39	ubiquitin specific peptidase 39	-0.026
158534071	IL4R	interleukin 4 receptor	-0.025
57527209	UBA5	ubiquitin like modifier activating enzyme 5	-0.025
149047197	STIM2	stromal interaction molecule 2	-0.023
157819063	SRPK2	SRSF protein kinase 2	-0.022
74185161	PSMC3	proteasome 26S subunit, ATPase 3	-0.018
157819459	MRPS2	mitochondrial ribosomal protein S2	-0.011
564301354	PRRC2B	proline rich coiled-coil 2B	0.015
157820811	PCDHB14	protocadherin beta 14	0.015
672046138	N/A	N/A	0.022
157818483	GSPT2	G1 to S phase transition 2	0.024
392333100	FAM193A	family with sequence similarity 193 member A	0.027
564384241	NOP14	NOP14 nucleolar protein	0.029
58866026	XK	X-linked Kx blood group	0.033
157817541	GEMIN4	gem nuclear organelle associated protein 4	0.033
12004970	RNF11	ring finger protein 11	0.033
149022924	SCG5	secretogranin V	0.034
672046314	AMBRA1	autophagy and beclin 1 regulator 1	0.034

672067583	N/A	N/A	0.034
194474082	RRP7A	ribosomal RNA processing 7 homolog A	0.034
392343022	ZNF157	zinc finger protein 157	0.034
664752274	N/A	N/A	0.035
521020961	N/A	N/A	0.037
4507133	SNRPG	small nuclear ribonucleoprotein polypeptide G	0.037
148686551	PPWD1	peptidylprolyl isomerase domain and WD repeat containing 1	0.038
157821407	FBXO28	F-box protein 28	0.039
402794761	ARHGAP35	Rho GTPase activating protein 35	0.041
58037463	KLHDC10	kelch domain containing 10	0.041
199561474	VPS39	VPS39, HOPS complex subunit	0.041
62945264	IST1	IST1, ESCRT-III associated factor	0.042
157823135	Dpp8	dipeptidylpeptidase 8	0.044
672066031	DGKD	diacylglycerol kinase delta	0.044
961766277	N/A	N/A	0.044
162135934	TPST1	tyrosylprotein sulfotransferase 1	0.044
157819577	SAP30BP	SAP30 binding protein	0.045
22129759	ZWINT	ZW10 interacting kinetochore protein	0.045
77627906	KLHDC2	kelch domain containing 2	0.045
45478098	CMTR1	cap methyltransferase 1	0.046
183985961	MAN1B1	mannosidase alpha class 1B member 1	0.046
9506875	SMAD4	SMAD family member 4	0.047
564379423	N/A	N/A	0.047
29789096	PRKAR2A	protein kinase cAMP-dependent type II regulatory subunit alpha	0.047
148698058	MTFR1L	mitochondrial fission regulator 1 like	0.048
300798184	GRAMD1B	GRAM domain containing 1B	0.049
826351127	N/A	N/A	0.049
588480474	FRG1	FSHD region gene 1	0.051
13591894	AKR1A1	aldo-keto reductase family 1 member A1	0.051
187469177	WDR41	WD repeat domain 41	0.051
300797122	FRMD4A	FERM domain containing 4A	0.051
564352872	N/A	N/A	0.051
149049696	MKRN2	makorin ring finger protein 2	0.052
81295337	SLC25A40	solute carrier family 25 member 40	0.052
149052177	MRPL28	mitochondrial ribosomal protein L28	0.052
29825827	VPS26B	VPS26, retromer complex component B	0.052
25742663	MYT1L	myelin transcription factor 1 like	0.052
157820825	IFT57	intraflagellar transport 57	0.053

20544149	CSNK1D	casein kinase 1 delta	0.053
76443681	USP11	ubiquitin specific peptidase 11	0.053
300798222	SIAH3	siah E3 ubiquitin protein ligase family member 3	0.054
50511039	GNB1L	G protein subunit beta 1 like	0.054
148690831	PRMT1	protein arginine methyltransferase 1	0.054
157817871	MEGF9	multiple EGF like domains 9	0.055
33468857	HINT1	histidine triad nucleotide binding protein 1	0.055
564398411	N/A	N/A	0.055
157819325	SRP68	signal recognition particle 68	0.056
672056705	N/A	N/A	0.056
55250051	TXNRD1	thioredoxin reductase 1	0.056
148706586	TMEM178A	transmembrane protein 178A	0.057
58865796	PTDSS1	phosphatidylserine synthase 1	0.057
18644718	RGS3	regulator of G protein signaling 3	0.057
672073969	CLASP1	cytoplasmic linker associated protein 1	0.057
564375166	N/A	N/A	0.058
148697565	FAM83H	family with sequence similarity 83 member H	0.059
729042261	KBTBD6	kelch repeat and BTB domain containing 6	0.059
57527131	ACTR10	actin related protein 10 homolog	0.060
880909835	N/A	N/A	0.060
62078979	AMZ2	archaelysin family metallopeptidase 2	0.061
564387371	N/A	N/A	0.062
8393519	H2AFY	H2A histone family member Y	0.062
189011600	GPN1	GPN-loop GTPase 1	0.063
157816927	GMEB1	glucocorticoid modulatory element binding protein 1	0.063
62078949	GPC4	glypican 4	0.063
743739270	N/A	N/A	0.064
157819485	PLPPR5	phospholipid phosphatase related 5	0.064
293349181	HINFP	histone H4 transcription factor	0.065
149057651	TFDP1	transcription factor Dp-1	0.065
826286778	N/A	N/A	0.065
149039803	UBQLN1	ubiquilin 1	0.065
667245009	N/A	N/A	0.065
157822779	DNAJC11	DnaJ heat shock protein family (Hsp40) member C11	0.066
25453374	PEX14	peroxisomal biogenesis factor 14	0.067
157821879	ATF6	activating transcription factor 6	0.067
58865660	SPRY2	sprouty RTK signaling antagonist 2	0.067

157786974	Wdr83os	WD repeat domain 83 opposite strand	0.067
61557293	DNAJB6	DnaJ heat shock protein family (Hsp40) member B6	0.067
57528331	MAD2L1BP	MAD2L1 binding protein	0.068
37360568	RANGAP1	Ran GTPase activating protein 1	0.068
111494225	SELENOF	selenoprotein F	0.068
62089200	ZDHHC9	zinc finger DHHC-type containing 9	0.068
70778952	RAD23B	RAD23 homolog B, nucleotide excision repair protein	0.069
300797313	DUSP23	dual specificity phosphatase 23	0.069
68163473	THEM4	thioesterase superfamily member 4	0.069
25742568	DPYSL3	dihydropyrimidinase like 3	0.069
157821757	MRPL20	mitochondrial ribosomal protein L20	0.070
764020110	HNRNPUL2	heterogeneous nuclear ribonucleoprotein U like 2	0.070
672044697	EHMT1	euchromatic histone lysine methyltransferase 1	0.070
149042824	UBE2V1	ubiquitin conjugating enzyme E2 V1	0.070
564356550	PCNX1	pecanex homolog 1	0.070
564347292	ST3GAL5	ST3 beta-galactoside alpha-2,3-sialyltransferase 5	0.071
405113057	TBCD	tubulin folding cofactor D	0.071
392338110	TBC1D12	TBC1 domain family member 12	0.071
149031779	PRR3	proline rich 3	0.072
8393746	MAP2K5	mitogen-activated protein kinase kinase 5	0.072
403420594	CSRNP3	cysteine and serine rich nuclear protein 3	0.072
947197810	N/A	N/A	0.072
149731291	N/A	N/A	0.072
157786896	FIS1	fission, mitochondrial 1	0.073
148668459	N/A	N/A	0.073
564313416	RUNDC1	RUN domain containing 1	0.073
537241567	N/A	N/A	0.073
564334146	SLC25A28	solute carrier family 25 member 28	0.074
564353714	FBXO42	F-box protein 42	0.074
47718012	ATP6AP1	ATPase H+ transporting accessory protein 1	0.074
12861068	CFL1	cofilin 1	0.074
532046645	N/A	N/A	0.075
89363040	PCDHGA11	protocadherin gamma subfamily A, 11	0.075
564326269	RPL28	ribosomal protein L28	0.075
157073957	PNRC2	proline rich nuclear receptor coactivator 2	0.075
11120706	PLRG1	pleiotropic regulator 1	0.075

157822653	CD2BP2	CD2 cytoplasmic tail binding protein 2	0.075
564371002	CPEB4	cytoplasmic polyadenylation element binding protein 4	0.076
564366360	N/A	N/A	0.076
112984152	PES1	pescadillo ribosomal biogenesis factor 1	0.076
62078453	INPP5K	inositol polyphosphate-5-phosphatase K	0.077
40018592	HP1BP3	heterochromatin protein 1 binding protein 3	0.077
564345064	N/A	N/A	0.078
109692276	UBE2E3	ubiquitin conjugating enzyme E2 E3	0.078
755492511	MAP4K4	mitogen-activated protein kinase kinase kinase kinase 4	0.078
12083657	BAD	BCL2 associated agonist of cell death	0.078
672031258	N/A	N/A	0.078
157817783	SNX18	sorting nexin 18	0.079
68341979	PLEKHO1	pleckstrin homology domain containing O1	0.080
9507059	RNF5	ring finger protein 5	0.080
119351041	SYNRG	synergin gamma	0.080
51854215	RNF34	ring finger protein 34	0.080
148672705	TMEM184B	transmembrane protein 184B	0.080
149032182	TMEM106C	transmembrane protein 106C	0.081
37360414	NPLOC4	NPL4 homolog, ubiquitin recognition factor	0.081
13162349	ASIC1	acid sensing ion channel subunit 1	0.082
351710149	N/A	N/A	0.082
169790975	MRPS9	mitochondrial ribosomal protein S9	0.082
83649695	SMIM14	small integral membrane protein 14	0.082
60360532	OSBPL6	oxysterol binding protein like 6	0.083
157820107	PSMD11	proteasome 26S subunit, non-ATPase 11	0.083
672074758	NCSTN	nicastrin	0.083
157817696	PIN1	peptidylprolyl cis/trans isomerase, NIMA-interacting 1	0.083
672047204	N/A	N/A	0.083
148747227	SV2A	synaptic vesicle glycoprotein 2A	0.084
672081581	RANBP9	RAN binding protein 9	0.084
58865982	PIGC	phosphatidylinositol glycan anchor biosynthesis class C	0.084
214010118	TMEM59	transmembrane protein 59	0.085
564380740	Ppp1r12b	protein phosphatase 1, regulatory (inhibitor) subunit 12B	0.085
528762703	N/A	N/A	0.085

157818421	TVP23A	trans-golgi network vesicle protein 23 homolog A	0.085
6978449	ADD2	adducin 2	0.085
149033227	N/A	N/A	0.085
157817127	SOCS5	suppressor of cytokine signaling 5	0.086
148709653	N/A	N/A	0.086
157818873	ZNHIT6	zinc finger HIT-type containing 6	0.086
672080385	N/A	N/A	0.086
148693919	HMG20A	high mobility group 20A	0.086
48675371	C1QBP	complement C1q binding protein	0.086
564305145	NOL6	nucleolar protein 6	0.086
148667067	N/A	N/A	0.087
149019062	TMEM30A	transmembrane protein 30A	0.087
823394616	N/A	N/A	0.087
820973213	N/A	N/A	0.087
564386141	ACIN1	apoptotic chromatin condensation inducer 1	0.088
157819911	CSGALNACT2	chondroitin sulfate N-acetylgalactosaminyltransferase 2	0.088
149048726	ZNF267	zinc finger protein 267	0.088
401461805	ACAT1	acetyl-CoA acetyltransferase 1	0.089
987959513	N/A	N/A	0.089
40786445	WRB	tryptophan rich basic protein	0.089
537231819	N/A	N/A	0.089
189163515	USP47	ubiquitin specific peptidase 47	0.089
564325173	N/A	N/A	0.089
672040889	N/A	N/A	0.089
672075032	MARK1	microtubule affinity regulating kinase 1	0.089
758818546	NFE2L3	nuclear factor, erythroid 2 like 3	0.090
112807209	EXT1	exostosin glycosyltransferase 1	0.090
290560659	ZNF609	zinc finger protein 609	0.090
672066520	N/A	N/A	0.090
755756742	N/A	N/A	0.090
62078823	RMDN3	regulator of microtubule dynamics 3	0.090
967489450	MEX3C	mex-3 RNA binding family member C	0.091
564387037	COG3	component of oligomeric golgi complex 3	0.091
26346731	UBE2Z	ubiquitin conjugating enzyme E2 Z	0.091
564363529	NCAM1	neural cell adhesion molecule 1	0.091
281604140	THUMPD3	THUMP domain containing 3	0.091
149038024	RIPOR1	RHO family interacting cell polarization regulator 1	0.091

149016466	NIPBL	NIPBL, cohesin loading factor	0.092
564311321	N/A	N/A	0.092
22024394	FABP5	fatty acid binding protein 5	0.092
564356319	DAAM1	dishevelled associated activator of morphogenesis 1	0.093
62543509	DPH2	DPH2 homolog	0.093
564356795	ALKBH1	alkB homolog 1, histone H2A dioxygenase	0.094
640797812	N/A	N/A	0.094
795342519	N/A	N/A	0.094
564332984	OSBP	oxysterol binding protein	0.094
13385090	COX6B1	cytochrome c oxidase subunit 6B1	0.095
197313691	XPO6	exportin 6	0.095
60360602	KLHL20	kelch like family member 20	0.095
161760661	KCNK9	potassium two pore domain channel subfamily K member 9	0.095
14389301	SMPD2	sphingomyelin phosphodiesterase 2	0.096
672071786	N/A	N/A	0.096
528758424	N/A	N/A	0.096
672041947	ZNF704	zinc finger protein 704	0.096
189491673	FXR2	FMR1 autosomal homolog 2	0.097
209447030	DDX27	DEAD-box helicase 27	0.097
982285416	N/A	N/A	0.097
157073961	Nudcd3	NudC domain containing 3	0.097
564398783	USP27X	ubiquitin specific peptidase 27, X-linked	0.098
62751974	SNAP29	synaptosome associated protein 29	0.098
119618921	RAN	RAN, member RAS oncogene family	0.098
672084054	N/A	N/A	0.098
157817586	USP8	ubiquitin specific peptidase 8	0.098
970596961	MAPK10	mitogen-activated protein kinase 10	0.099
291084664	TRAPPC10	trafficking protein particle complex 10	0.099
157818285	C12orf49	chromosome 12 open reading frame 49	0.099
528767051	N/A	N/A	0.100
12621140	PIK3C3	phosphatidylinositol 3-kinase catalytic subunit type 3	0.100
300797976	ANKRD50	ankyrin repeat domain 50	0.100
13994121	FEZ1	fasciculation and elongation protein zeta 1	0.100
241666404	EPHA4	EPH receptor A4	0.100
725607246	N/A	N/A	0.100
564352364	N/A	N/A	0.100
157822179	COMMD8	COMM domain containing 8	0.100

564361208	SCUBE1	signal peptide, CUB domain and EGF like domain containing 1	0.100
114145722	RTRAF	RNA transcription, translation and transport factor	0.100
655605091	N/A	N/A	0.100
564338596	N/A	N/A	0.101
514475096	N/A	N/A	0.101
347921120	SLC23A2	solute carrier family 23 member 2	0.101
71043624	AZI2	5-azacytidine induced 2	0.101
166064004	GTF3A	general transcription factor IIIA	0.101
164565398	RBFOX1	RNA binding fox-1 homolog 1	0.101
672063576	N/A	N/A	0.102
57164027	PMS1	PMS1 homolog 1, mismatch repair system component	0.102
157819591	PARP6	poly(ADP-ribose) polymerase family member 6	0.102
22096326	PRKAB1	protein kinase AMP-activated non-catalytic subunit beta 1	0.102
431895484	N/A	N/A	0.103
27545414	LGR4	leucine rich repeat containing G protein-coupled receptor 4	0.103
585165276	N/A	N/A	0.103
28972173	KIF3B	kinesin family member 3B	0.103
795356643	N/A	N/A	0.103
672047825	N/A	N/A	0.103
564368059	KANSL3	KAT8 regulatory NSL complex subunit 3	0.103
157821483	BORCS5	BLOC-1 related complex subunit 5	0.103
148697875	GDI1	GDP dissociation inhibitor 1	0.103
58865572	FBXO9	F-box protein 9	0.104
803119291	N/A	N/A	0.104
58865712	RRP1	ribosomal RNA processing 1	0.104
70794762	HARS	histidyl-tRNA synthetase	0.104
564352682	PABPC4	poly(A) binding protein cytoplasmic 4	0.104
966932741	TMED4	transmembrane p24 trafficking protein 4	0.105
46391106	ARL10	ADP ribosylation factor like GTPase 10	0.105
564367956	CNNM3	cyclin and CBS domain divalent metal cation transport mediator 3	0.105
80751173	PCDHGA1	protocadherin gamma subfamily A, 1	0.106
149047559	MTMR3	myotubularin related protein 3	0.106
157821137	TMEM128	transmembrane protein 128	0.106
42476292	TALDO1	transaldolase 1	0.106

564358970	CDK17	cyclin dependent kinase 17	0.107
149040413	GPAM	glycerol-3-phosphate acyltransferase, mitochondrial	0.107
803119291	N/A	N/A	0.107
672054841	RCC2	regulator of chromosome condensation 2	0.107
564382285	RPS6KC1	ribosomal protein S6 kinase C1	0.107
564377243	OPA1	OPA1, mitochondrial dynamin like GTPase	0.107
444730664	N/A	N/A	0.107
431917236	N/A	N/A	0.107
431917236	N/A	N/A	0.107
86477155	PER1	period circadian regulator 1	0.108
62543537	TBC1D10A	TBC1 domain family member 10A	0.108
831324638	N/A	N/A	0.108
831234449	N/A	N/A	0.108
148673403	GRSF1	G-rich RNA sequence binding factor 1	0.108
61889115	PSAT1	phosphoserine aminotransferase 1	0.108
149063993	NEDD8	neural precursor cell expressed, developmentally down-regulated 8	0.109
19526763	CRCP	CGRP receptor component	0.109
157822563	AREL1	apoptosis resistant E3 ubiquitin protein ligase 1	0.109
564320563	RBM27	RNA binding motif protein 27	0.109
927205201	N/A	N/A	0.110
402534556	ZHX2	zinc fingers and homeoboxes 2	0.110
41054868	FPGT	fucose-1-phosphate guanylyltransferase	0.110
158631164	IGSF3	immunoglobulin superfamily member 3	0.110
157818159	AAR2	AAR2 splicing factor homolog	0.110
149046938	N/A	N/A	0.110
672050419	SLC6A6	solute carrier family 6 member 6	0.110
955507084	N/A	N/A	0.110
38454206	PSMD6	proteasome 26S subunit, non-ATPase 6	0.110
672027713	BMP2K	BMP2 inducible kinase	0.111
157786720	HIVEP1	human immunodeficiency virus type I enhancer binding protein 1	0.111
686661085	MTMR4	myotubularin related protein 4	0.111
564336099	PHC3	polyhomeotic homolog 3	0.112
672048159	RAE1	ribonucleic acid export 1	0.112
927219744	N/A	N/A	0.112
13928860	IGBP1	immunoglobulin binding protein 1	0.112
30794434	SRRM4	serine/arginine repetitive matrix 4	0.112

564388185	ERCC6	ERCC excision repair 6, chromatin remodeling factor	0.112
90108450	TAB2	TGF-beta activated kinase 1/MAP3K7 binding protein 2	0.113
594064744	N/A	N/A	0.113
158341666	SEL1L	SEL1L ERAD E3 ligase adaptor subunit	0.113
157817811	C5orf22	chromosome 5 open reading frame 22	0.113
880893486	N/A	N/A	0.114
56090369	TMX2	thioredoxin related transmembrane protein 2	0.114
170295834	NDUFA10	NADH:ubiquinone oxidoreductase subunit A10	0.114
611991798	N/A	N/A	0.114
78214352	CSRP2	cysteine and glycine rich protein 2	0.114
274325505	Pwp2	PWP2 periodic tryptophan protein homolog (yeast)	0.115
67078504	YTHDF1	YTH N6-methyladenosine RNA binding protein 1	0.115
564309116	RTL6	retrotransposon Gag like 6	0.115
564360651	LRRC14	leucine rich repeat containing 14	0.115
51948396	TUSC3	tumor suppressor candidate 3	0.116
725571770	N/A	N/A	0.116
955478861	N/A	N/A	0.116
53828922	PCYT1A	phosphate cytidylyltransferase 1, choline, alpha	0.116
564317984	N/A	N/A	0.116
672067122	N/A	N/A	0.116
674097005	N/A	N/A	0.116
25742739	ACSL1	acyl-CoA synthetase long chain family member 1	0.117
564353232	PUM1	pumilio RNA binding family member 1	0.117
293347618	RLF	rearranged L-myc fusion	0.117
755502295	SAMD10	sterile alpha motif domain containing 10	0.117
189011602	NLE1	notchless homolog 1	0.118
274321371	CRLF3	cytokine receptor like factor 3	0.118
397524574	N/A	N/A	0.118
81158095	PCDHGA3	protocadherin gamma subfamily A, 3	0.118
47847438	EXOC3	exocyst complex component 3	0.118
564372486	N/A	N/A	0.118
68163425	TMEM199	transmembrane protein 199	0.118
564358968	N/A	N/A	0.118
403224979	PPP4R3A	protein phosphatase 4 regulatory subunit 3A	0.118
11067423	SMAD5	SMAD family member 5	0.118

18959272	KCNQ2	potassium voltage-gated channel subfamily Q member 2	0.119
672040889	N/A	N/A	0.119
40018576	TOR1AIP2	torsin 1A interacting protein 2	0.119
300793721	TLK2	tousled like kinase 2	0.119
401709965	ANXA3	annexin A3	0.119
404501464	IFNAR1	interferon alpha and beta receptor subunit 1	0.119
507693337	N/A	N/A	0.120
157816973	PPP1R8	protein phosphatase 1 regulatory subunit 8	0.120
532015294	N/A	N/A	0.120
157818061	2510002D24Rik	RIKEN cDNA 2510002D24 gene	0.120
61556860	MRPL46	mitochondrial ribosomal protein L46	0.120
149050200	GPR180	G protein-coupled receptor 180	0.120
58865512	STRAP	serine/threonine kinase receptor associated protein	0.120
13242293	HMGCL	3-hydroxymethyl-3-methylglutaryl-CoA lyase	0.121
66730484	TAOK3	TAO kinase 3	0.121
293344794	FAM160B1	family with sequence similarity 160 member B1	0.121
109510888	FAM155B	family with sequence similarity 155 member B	0.121
402743472	SLC3A2	solute carrier family 3 member 2	0.121
562828743	N/A	N/A	0.121
148680747	ANKFY1	ankyrin repeat and FYVE domain containing 1	0.122
672045179	MAPKAP1	mitogen-activated protein kinase associated protein 1	0.122
157822659	RIOK3	RIO kinase 3	0.122
564319816	N/A	N/A	0.122
58865648	SAMD8	sterile alpha motif domain containing 8	0.122
555997205	N/A	N/A	0.122
641719431	N/A	N/A	0.122
114326177	SHMT1	serine hydroxymethyltransferase 1	0.123
148704614	N/A	N/A	0.123
672017866	DIDO1	death inducer-obliterator 1	0.123
942047300	N/A	N/A	0.123
672060579	SENP1	SUMO1/sentrin specific peptidase 1	0.123
392347634	CHD4	chromodomain helicase DNA binding protein 4	0.124
18041977	Serbp1	Serpine1 mRNA binding protein 1	0.124
672073083	N/A	N/A	0.124
393716310	WAC	WW domain containing adaptor with coiled-coil	0.124
672044181	HS2ST1	heparan sulfate 2-O-sulfotransferase 1	0.124

109479851	NRDE2	NRDE-2, necessary for RNA interference, domain containing	0.124
162287304	CDC42EP3	CDC42 effector protein 3	0.124
60359872	G3BP1	G3BP stress granule assembly factor 1	0.125
755530088	N/A	N/A	0.125
25742623	UGCG	UDP-glucose ceramide glucosyltransferase	0.125
148705576	CRMP1	collapsin response mediator protein 1	0.125
164664442	PIAS1	protein inhibitor of activated STAT 1	0.125
655660415	N/A	N/A	0.125
564381925	ACKR1	atypical chemokine receptor 1 (Duffy blood group)	0.125
157818629	HEYL	hes related family bHLH transcription factor with YRPW motif-like	0.126
564328894	N/A	N/A	0.126
157817011	LRRC28	leucine rich repeat containing 28	0.126
155369279	RSPRY1	ring finger and SPRY domain containing 1	0.126
19705555	IPMK	inositol polyphosphate multikinase	0.126
149016735	N/A	N/A	0.127
11968114	MRPL23	mitochondrial ribosomal protein L23	0.127
209977101	TRPM4	transient receptor potential cation channel subfamily M member 4	0.127
58866022	MGAT4A	mannosyl (alpha-1,3-)-glycoprotein beta-1,4-N-acetylglucosaminyltransferase, isozyme A	0.127
454601639	NCOA6	nuclear receptor coactivator 6	0.127
16758194	RGS2	regulator of G protein signaling 2	0.127
672068548	SUPT6H	SPT6 homolog, histone chaperone	0.127
768711622	MPEG1	macrophage expressed 1	0.128
564361244	TCF20	transcription factor 20	0.128
51036652	SLC33A1	solute carrier family 33 member 1	0.128
564331450	EEF1AKMT2	EEF1A lysine methyltransferase 2	0.129
148677380	MSANTD4	Myb/SANT DNA binding domain containing 4 with coiled-coils	0.129
162138924	YKT6	YKT6 v-SNARE homolog	0.129
270483894	CAMSAP1	calmodulin regulated spectrin associated protein 1	0.129
568957557	N/A	N/A	0.129
148747275	MCM7	minichromosome maintenance complex component 7	0.130
564361462	BRD1	bromodomain containing 1	0.130
672049965	N/A	N/A	0.130

23271707	EIF3B	eukaryotic translation initiation factor 3 subunit B	0.130
76008363	BICD2	BICD cargo adaptor 2	0.130
672042705	FNIP2	folliculin interacting protein 2	0.130
62656582	KIAA0100	KIAA0100	0.130
564363852	SNUPN	snurportin 1	0.131
300797157	TBC1D8	TBC1 domain family member 8	0.131
325974480	NDUFA7	NADH:ubiquinone oxidoreductase subunit A7	0.131
672063411	SETD2	SET domain containing 2	0.131
157821015	KDM5B	lysine demethylase 5B	0.131
60359978	KIF3C	kinesin family member 3C	0.131
148693260	TIMM29	translocase of inner mitochondrial membrane 29	0.131
17978459	Atp5k	ATP synthase, H+ transporting, mitochondrial F1F0 complex, subunit E	0.131
157824037	USP4	ubiquitin specific peptidase 4	0.131
68299787	TAF9	TATA-box binding protein associated factor 9	0.131
564396315	TAF5L	TATA-box binding protein associated factor 5 like	0.132
157821561	ATRIP	ATR interacting protein	0.132
589941671	N/A	N/A	0.132
77404259	RPAP1	RNA polymerase II associated protein 1	0.132
625263830	N/A	N/A	0.132
564390552	N/A	N/A	0.133
564333160	TMEM2	transmembrane protein 2	0.133
440890867	N/A	N/A	0.133
83582792	FAM117B	family with sequence similarity 117 member B	0.133
158749598	LRIG2	leucine rich repeats and immunoglobulin like domains 2	0.133
58865976	KLHDC3	kelch domain containing 3	0.133
11177894	TSC1	TSC complex subunit 1	0.134
149036172	FAM32A	family with sequence similarity 32 member A	0.134
150421568	N/A	N/A	0.134
50510937	MBD5	methyl-CpG binding domain protein 5	0.134
564397283	FKBP5	FK506 binding protein 5	0.134
157823607	ALDH18A1	aldehyde dehydrogenase 18 family member A1	0.134
8394405	SLC7A5	solute carrier family 7 member 5	0.134
148682677	MTERF1	mitochondrial transcription termination factor 1	0.135

149018452	SMARCC1	SWI/SNF related, matrix associated, actin dependent regulator of chromatin subfamily c member 1	0.135
13242322	ATF4	activating transcription factor 4	0.135
162287067	VAV1	vav guanine nucleotide exchange factor 1	0.135
29293823	SMUG1	single-strand-selective monofunctional uracil-DNA glycosylase 1	0.135
564398269	SCML4	Scm polycomb group protein like 4	0.135
451770387	PRRT2	proline rich transmembrane protein 2	0.136
61557130	B3GALNT1	beta-1,3-N-acetylgalactosaminyltransferase 1 (globoside blood group)	0.136
14010879	PSMD1	proteasome 26S subunit, non-ATPase 1	0.136
148747116	BIRC2	baculoviral IAP repeat containing 2	0.136
61556945	MOAP1	modulator of apoptosis 1	0.136
157786872	USP42	ubiquitin specific peptidase 42	0.136
157821401	UQCC1	ubiquinol-cytochrome c reductase complex assembly factor 1	0.136
18266706	ELP1	elongator complex protein 1	0.137
564398139	FYN	FYN proto-oncogene, Src family tyrosine kinase	0.137
537206173	N/A	N/A	0.137
157816981	DNAJC30	DnaJ heat shock protein family (Hsp40) member C30	0.137
67078426	SPIN1	spindlin 1	0.137
71043634	PDCL3	phosducin like 3	0.137
672060460	N/A	N/A	0.137
148491097	DYNC1H1	dynein cytoplasmic 1 heavy chain 1	0.137
674070361	N/A	N/A	0.137
157786686	KLHL11	kelch like family member 11	0.138
795270501	N/A	N/A	0.138
470611927	N/A	N/A	0.138
16758782	LMNB1	lamin B1	0.138
564393026	BRD8	bromodomain containing 8	0.138
77404168	WDR77	WD repeat domain 77	0.138
58865952	UBAP1	ubiquitin associated protein 1	0.139
158081739	B4GALT1	beta-1,4-galactosyltransferase 1	0.140
157818305	GPR101	G protein-coupled receptor 101	0.140
672069253	SOCS7	suppressor of cytokine signaling 7	0.141

157820897	MTHFD2	methylenetetrahydrofolate dehydrogenase (NADP+ dependent) 2, methenyltetrahydrofolate cyclohydrolase	0.141
672082436	N/A	N/A	0.141
795288364	N/A	N/A	0.141
157821921	QSOX2	quiescin sulfhydryl oxidase 2	0.141
58865606	FTSJ3	FtsJ RNA methyltransferase homolog 3	0.141
155369738	VPS33A	VPS33A, CORVET/HOPS core subunit	0.141
20301952	SLC2A1	solute carrier family 2 member 1	0.142
149025632	RBM15	RNA binding motif protein 15	0.142
19924073	TTL	tubulin tyrosine ligase	0.142
451172120	DUSP7	dual specificity phosphatase 7	0.142
60360118	FAM168B	family with sequence similarity 168 member B	0.142
56090235	UBAC1	UBA domain containing 1	0.142
61555249	NUDT11	nudix hydrolase 11	0.142
157819431	BRD3	bromodomain containing 3	0.142
40385881	ACVR1B	activin A receptor type 1B	0.143
213972547	KAT6A	lysine acetyltransferase 6A	0.143
71043834	RBMX	RNA binding motif protein, X-linked	0.143
655872066	N/A	N/A	0.143
74139306	TMED9	transmembrane p24 trafficking protein 9	0.143
672056381	PCNX4	pecanex homolog 4	0.143
157073939	LOC728392	uncharacterized LOC728392	0.143
987429374	N/A	N/A	0.144
564370907	ZNF598	zinc finger protein 598	0.144
9624979	ENSA	endosulfine alpha	0.144
46485444	NOP53	NOP53 ribosome biogenesis factor	0.144
55715816	GLYR1	glyoxylate reductase 1 homolog	0.144
6981458	RAF1	Raf-1 proto-oncogene, serine/threonine kinase	0.144
625279732	N/A	N/A	0.144
12408292	PGR	progesterone receptor	0.144
676272934	N/A	N/A	0.144
149018374	N/A	N/A	0.145
674066860	N/A	N/A	0.145
291042494	MED13L	mediator complex subunit 13 like	0.145
672052423	N/A	N/A	0.145
742149236	N/A	N/A	0.145
149049279	N/A	N/A	0.145
157821933	EXOG	exo/endonuclease G	0.145
672024670	FAM196B	family with sequence similarity 196 member B	0.145

68534951	CD40	CD40 molecule	0.145
30017419	NREP	neuronal regeneration related protein	0.145
744598167	N/A	N/A	0.145
507680885	N/A	N/A	0.146
392333209	DLG5	discs large MAGUK scaffold protein 5	0.146
399124804	SLC4A8	solute carrier family 4 member 8	0.146
157818521	COG8	component of oligomeric golgi complex 8	0.146
157820585	SART3	squamous cell carcinoma antigen recognized by T cells 3	0.146
157786906	MRPS17	mitochondrial ribosomal protein S17	0.146
84781731	ZDHHC3	zinc finger DHHC-type containing 3	0.146
672088463	N/A	N/A	0.146
13928926	MYBBP1A	MYB binding protein 1a	0.146
966923099	SMG7	SMG7, nonsense mediated mRNA decay factor	0.146
426382957	N/A	N/A	0.147
914896367	N/A	N/A	0.147
149064388	Hmgxb3	HMG-box containing 3	0.147
300798201	PYGO1	pygopus family PHD finger 1	0.147
148747154	POLB	DNA polymerase beta	0.147
148679302	CBFB	core-binding factor beta subunit	0.148
157818165	SMURF2	SMAD specific E3 ubiquitin protein ligase 2	0.148
62079033	PRMT7	protein arginine methyltransferase 7	0.148
403225011	SAMHD1	SAM and HD domain containing deoxynucleoside triphosphate triphosphohydrolase 1	0.148
41386755	FGFR1OP2	FGFR1 oncogene partner 2	0.148
293341411	Rc3h1	ring finger and CCCH-type domains 1	0.148
61556879	PKNOX1	PBX/knotted 1 homeobox 1	0.148
155369656	AQR	aquarius intron-binding spliceosomal factor	0.149
564319708	N/A	N/A	0.149
149022577	FNBP4	formin binding protein 4	0.149
148665617	NAA50	N(alpha)-acetyltransferase 50, NatE catalytic subunit	0.149
281427188	ZC3H13	zinc finger CCCH-type containing 13	0.149
114145618	MIS12	MIS12, kinetochore complex component	0.149
149722114	N/A	N/A	0.149
564321656	TCF25	transcription factor 25	0.149
68342005	HEXIM1	hexamethylene bisacetamide inducible 1	0.150
404351673	PPP1R21	protein phosphatase 1 regulatory subunit 21	0.150
157819885	SETD5	SET domain containing 5	0.150

157821581	PSMD13	proteasome 26S subunit, non-ATPase 13	0.150
40786455	BPGM	bisphosphoglycerate mutase	0.150
564311031	CLPP	caseinolytic mitochondrial matrix peptidase proteolytic subunit	0.150
392351939	BRWD1	bromodomain and WD repeat domain containing 1	0.150
114051946	YTHDF2	YTH N6-methyladenosine RNA binding protein 2	0.150
157819423	SPSB3	splA/ryanodine receptor domain and SOCS box containing 3	0.150
755537448	N/A	N/A	0.151
198278505	RPL7	ribosomal protein L7	0.151
82654218	AIMP2	aminoacyl tRNA synthetase complex interacting multifunctional protein 2	0.151
208973276	TMEM185A	transmembrane protein 185A	0.151
76363516	FEZ2	fasciculation and elongation protein zeta 2	0.152
16758772	CGRRF1	cell growth regulator with ring finger domain 1	0.152
157820079	C14orf28	chromosome 14 open reading frame 28	0.152
795554188	N/A	N/A	0.152
157823453	RBSN	rabenosyn, RAB effector	0.152
55741780	SPG21	SPG21, maspardin	0.152
918594634	N/A	N/A	0.153
918577634	N/A	N/A	0.153
829979268	N/A	N/A	0.153
568918679	TTC17	tetratricopeptide repeat domain 17	0.153
157820865	DDX28	DEAD-box helicase 28	0.153
617592859	N/A	N/A	0.153
51036650	MCL1	MCL1, BCL2 family apoptosis regulator	0.153
564394905	ZSWIM4	zinc finger SWIM-type containing 4	0.153
261337195	WDR91	WD repeat domain 91	0.153
157817718	DCAF10	DDB1 and CUL4 associated factor 10	0.153
149024327	C1QB	complement C1q B chain	0.154
13928696	JAK2	Janus kinase 2	0.154
300253233	LEMD3	LEM domain containing 3	0.154
148679797	DEF8	differentially expressed in FDCP 8 homolog	0.155
2088637	EED	embryonic ectoderm development	0.155
66730376	Arxes1/Arxes2	adipocyte-related X-chromosome expressed sequence 2	0.155
209863130	SEMA3F	semaphorin 3F	0.155
50511177	SLITRK1	SLIT and NTRK like family member 1	0.155

564361543	PPHLN1	periphilin 1	0.155
148702702	N/A	N/A	0.156
402692225	RNF168	ring finger protein 168	0.156
70608151	FAM118B	family with sequence similarity 118 member B	0.156
50510427	IP6K1	inositol hexakisphosphate kinase 1	0.156
30842813	SLC38A2	solute carrier family 38 member 2	0.156
171543899	PLXNA4	plexin A4	0.156
157820917	CDC7	cell division cycle 7	0.156
157822569	Tubgcp3	tubulin, gamma complex associated protein 3	0.156
224593264	BORCS8	BLOC-1 related complex subunit 8	0.156
821005859	N/A	N/A	0.156
564393951	MBD1	methyl-CpG binding domain protein 1	0.157
197927166	AGPAT5	1-acylglycerol-3-phosphate O-acyltransferase 5	0.157
41054820	CHRDL1	chordin like 1	0.157
157824174	HIGD2A	HIG1 hypoxia inducible domain family member 2A	0.157
829969914	N/A	N/A	0.157
564390490	N/A	N/A	0.157
201027430	WDR43	WD repeat domain 43	0.158
300798312	POLR3B	RNA polymerase III subunit B	0.158
77917548	DUS3L	dihydrouridine synthase 3 like	0.158
9910378	CDC42SE2	CDC42 small effector 2	0.158
564381466	N/A	N/A	0.158
74200325	UBE2G2	ubiquitin conjugating enzyme E2 G2	0.158
300793740	TANC2	tetratricopeptide repeat, ankyrin repeat and coiled-coil containing 2	0.158
672051957	N/A	N/A	0.158
197333849	ATP23	ATP23 metallopeptidase and ATP synthase assembly factor homolog	0.158
28972858	VCPIP1	valosin containing protein interacting protein 1	0.158
403310660	LATS1	large tumor suppressor kinase 1	0.158
149038734	EIF4EBP2	eukaryotic translation initiation factor 4E binding protein 2	0.158
6978805	EMD	emerin	0.159
164663846	PHPT1	phosphohistidine phosphatase 1	0.159
672043520	PI4KB	phosphatidylinositol 4-kinase beta	0.159
41053837	GPX3	glutathione peroxidase 3	0.159
564355148	AGBL5	ATP/GTP binding protein like 5	0.159
157823988	BLOC1S5	biogenesis of lysosomal organelles complex 1 subunit 5	0.159

157817763	NEK9	NIMA related kinase 9	0.159
564315719	N/A	N/A	0.159
564380021	TTC28	tetratricopeptide repeat domain 28	0.160
76559935	TUT1	terminal uridylyl transferase 1, U6 snRNA-specific	0.160
19705483	CLSTN2	calsyntenin 2	0.160
157819421	CEP97	centrosomal protein 97	0.160
157818167	PDPR	pyruvate dehydrogenase phosphatase regulatory subunit	0.160
404312665	DKK3	dickkopf WNT signaling pathway inhibitor 3	0.160
6981442	PTPN1	protein tyrosine phosphatase, non-receptor type 1	0.160
300796412	ATMIN	ATM interactor	0.160
532003341	N/A	N/A	0.161
19924085	FAT3	FAT atypical cadherin 3	0.161
58219518	RND2	Rho family GTPase 2	0.161
926688833	N/A	N/A	0.161
170016030	DDX31	DEAD-box helicase 31	0.161
748983435	VEZF1	vascular endothelial zinc finger 1	0.162
149067653	N/A	N/A	0.162
213688411	LPCAT1	lysophosphatidylcholine acyltransferase 1	0.162
149755772	N/A	N/A	0.162
124487247	PRICKLE2	prickle planar cell polarity protein 2	0.162
114145788	NAA25	N(alpha)-acetyltransferase 25, NatB auxiliary subunit	0.162
672035849	N/A	N/A	0.163
672042487	NBEA	neurobeachin	0.163
564386027	CHD8	chromodomain helicase DNA binding protein 8	0.163
221040576	MPPED1	metallophosphoesterase domain containing 1	0.163
11559951	NRBF2	nuclear receptor binding factor 2	0.163
56090283	RCHY1	ring finger and CHY zinc finger domain containing 1	0.163
281485586	MAP3K7	mitogen-activated protein kinase kinase kinase 7	0.163
149033480	Zfp956	zinc finger protein 956	0.163
157817632	ZNF202	zinc finger protein 202	0.164
672072928	CUX2	cut like homeobox 2	0.164
672067460	RGD1560464	similar to hypothetical protein FLJ38426	0.164
60359854	POLDIP3	DNA polymerase delta interacting protein 3	0.164
70794797	USP3	ubiquitin specific peptidase 3	0.164

300798499	AFF3	AF4/FMR2 family member 3	0.164
157822501	MCM3AP	minichromosome maintenance complex component 3 associated protein	0.164
296491570	N/A	N/A	0.164
197927315	CCDC94	coiled-coil domain containing 94	0.164
112984440	TNFRSF19	TNF receptor superfamily member 19	0.164
149042848	LOC100911177	uncharacterized LOC100911177	0.165
564340867	MMADHC	methylmalonic aciduria and homocystinuria, cblD type	0.165
76559929	NOC2L	NOC2 like nucleolar associated transcriptional repressor	0.165
164663909	SDE2	SDE2 telomere maintenance homolog	0.165
672042133	SPATA5	spermatogenesis associated 5	0.165
198278423	IQCJ-SCHIP1	IQCJ-SCHIP1 readthrough	0.166
300797915	Rbm33	RNA binding motif protein 33	0.166
403225023	BRAP	BRCA1 associated protein	0.166
115647917	Zfp874a/Zfp874b	zinc finger protein 874b	0.166
148674168	DYNLRB1	dynein light chain roadblock-type 1	0.166
408772026	Afg3l1	AFG3-like AAA ATPase 1	0.166
8393104	CHKB	choline kinase beta	0.166
158854035	RNF146	ring finger protein 146	0.166
635065321	N/A	N/A	0.166
149030824	N/A	N/A	0.167
157822433	EID1	EP300 interacting inhibitor of differentiation 1	0.167
672031565	N/A	N/A	0.167
148670274	KIAA0368	KIAA0368	0.167
46485387	NAPRT	nicotinate phosphoribosyltransferase	0.167
197385832	RD3L	retinal degeneration 3-like	0.167
392333169	CCDC88A	coiled-coil domain containing 88A	0.168
672028565	Setdb2	SET domain, bifurcated 2	0.168
56605826	LAMTOR3	late endosomal/lysosomal adaptor, MAPK and MTOR activator 3	0.168
281371454	ZNF613	zinc finger protein 613	0.168
55926133	RFC2	replication factor C subunit 2	0.168
532090801	N/A	N/A	0.169
795537196	N/A	N/A	0.169
40018600	CNPPD1	cyclin Pas1/PHO80 domain containing 1	0.169
422398900	CREBZF	CREB/ATF bZIP transcription factor	0.169
62087532	SRSF6	serine and arginine rich splicing factor 6	0.169
37360236	SMG5	SMG5, nonsense mediated mRNA decay factor	0.169

6981572	SP4	Sp4 transcription factor	0.169
640823357	N/A	N/A	0.169
57528294	NEPRO	nucleolus and neural progenitor protein	0.169
149061271	N/A	N/A	0.170
47059500	NSG1	neuronal vesicle trafficking associated 1	0.170
50511063	EEPD1	endonuclease/exonuclease/phosphatase family domain containing 1	0.170
293359997	SGPP1	sphingosine-1-phosphate phosphatase 1	0.170
564392297	SLC39A12	solute carrier family 39 member 12	0.170
13929130	SLC12A2	solute carrier family 12 member 2	0.170
157818643	KCTD3	potassium channel tetramerization domain containing 3	0.170
149067744	Znf48	zinc finger protein 48	0.170
148707518	RNF2	ring finger protein 2	0.171
189027131	TEDC2	tubulin epsilon and delta complex 2	0.171
16758536	AATF	apoptosis antagonizing transcription factor	0.171
157823165	DNAJB1	DnaJ heat shock protein family (Hsp40) member B1	0.171
149044496	PLAA	phospholipase A2 activating protein	0.171
674077614	N/A	N/A	0.171
157823942	COMMD2	COMM domain containing 2	0.172
40807349	DSTYK	dual serine/threonine and tyrosine protein kinase	0.172
21489987	PCYOX1	prenylcysteine oxidase 1	0.172
149044495	CAAP1	caspase activity and apoptosis inhibitor 1	0.172
332245592	N/A	N/A	0.172
33356154	UBE2H	ubiquitin conjugating enzyme E2 H	0.172
157822067	BAP1	BRCA1 associated protein 1	0.172
564375676	N/A	N/A	0.173
451172068	ST6GAL2	ST6 beta-galactoside alpha-2,6-sialyltransferase 2	0.173
56605790	HCFC2	host cell factor C2	0.173
300794761	Fat4	FAT atypical cadherin 4	0.173
47155561	DNAJC7	DnaJ heat shock protein family (Hsp40) member C7	0.173
157786908	ZBED5	zinc finger BED-type containing 5	0.173
568972665	TSPOAP1	TSPO associated protein 1	0.173
148806879	DNTTIP1	deoxynucleotidyltransferase terminal interacting protein 1	0.174
300798476	THAP12	THAP domain containing 12	0.174

672040432	Mrpl43	mitochondrial ribosomal protein L43	0.174
157823565	COQ10A	coenzyme Q10A	0.174
392355126	HAUS2	HAUS augmin like complex subunit 2	0.175
831218355	N/A	N/A	0.175
60360266	PPP2R2A	protein phosphatase 2 regulatory subunit Balpha	0.175
148672575	N/A	N/A	0.175
545804423	N/A	N/A	0.175
672035601	CEBPG	CCAAT/enhancer binding protein gamma	0.175
564382144	LIN9	lin-9 DREAM MuvB core complex component	0.176
564303143	KMT2C	lysine methyltransferase 2C	0.176
344253328	N/A	N/A	0.176
197304795	SLC7A6OS	solute carrier family 7 member 6 opposite strand	0.176
189491869	KCMF1	potassium channel modulatory factor 1	0.176
119569672	BUB3	BUB3, mitotic checkpoint protein	0.176
209915609	PRICKLE1	prickle planar cell polarity protein 1	0.177
157821479	METTL25	methyltransferase like 25	0.177
56090552	GTF2F1	general transcription factor IIF subunit 1	0.177
37360004	KDM1A	lysine demethylase 1A	0.177
164565387	TBC1D14	TBC1 domain family member 14	0.177
21955259	OLFM3	olfactomedin 3	0.177
564298823	EML3	echinoderm microtubule associated protein like 3	0.177
537151309	N/A	N/A	0.178
157818889	NCBP3	nuclear cap binding subunit 3	0.178
149067325	TDG	thymine DNA glycosylase	0.178
148677171	N/A	N/A	0.178
149048628	MYNN	myoneurin	0.179
149054120	ORMDL3	ORMDL sphingolipid biosynthesis regulator 3	0.179
149044006	TEDC1	tubulin epsilon and delta complex 1	0.179
28972780	TLE3	transducin like enhancer of split 3	0.180
564323153	ARMCX5	armadillo repeat containing, X-linked 5	0.180
77695933	NELL2	neural EGFL like 2	0.180
392333209	DLG5	discs large MAGUK scaffold protein 5	0.180
164565360	CTTNBP2NL	CTTNBP2 N-terminal like	0.180
61556927	EIF3G	eukaryotic translation initiation factor 3 subunit G	0.180
56090317	MFAP3	microfibril associated protein 3	0.180
38454226	TPD52L2	tumor protein D52 like 2	0.180

62079005	SLAIN1	SLAIN motif family member 1	0.180
6981296	NUP50	nucleoporin 50	0.181
9506755	GRIK2	glutamate ionotropic receptor kainate type subunit 2	0.181
405113028	TAF3	TATA-box binding protein associated factor 3	0.181
6981518	SDC1	syndecan 1	0.182
672084956	N/A	N/A	0.182
256818812	RNF165	ring finger protein 165	0.182
157822303	GPR107	G protein-coupled receptor 107	0.182
157817773	ZNF641	zinc finger protein 641	0.183
157823413	THOC3	THO complex 3	0.183
28076889	YIPF4	Yip1 domain family member 4	0.183
300797562	BCOR	BCL6 corepressor	0.183
401709959	Ppp1cc	protein phosphatase 1, catalytic subunit, gamma isoform	0.183
392342217	RANBP3	RAN binding protein 3	0.184
21914829	PDCD4	programmed cell death 4	0.184
697350955	N/A	N/A	0.184
9507007	PTGFRN	prostaglandin F2 receptor inhibitor	0.184
564353949	N/A	N/A	0.184
162287391	RPL6	ribosomal protein L6	0.185
11139303	JTB	jumping translocation breakpoint	0.185
568972475	NCOR1	nuclear receptor corepressor 1	0.185
762006019	FAM8A1	family with sequence similarity 8 member A1	0.185
148747146	PPP2R2D	protein phosphatase 2 regulatory subunit Bdelta	0.185
58865626	UBXN4	UBX domain protein 4	0.186
119628283	MACO1	macoilin 1	0.186
157818115	PATL1	PAT1 homolog 1, processing body mRNA decay factor	0.187
568983220	PAPD7	poly(A) RNA polymerase D7, non-canonical	0.187
60360108	BRD2	bromodomain containing 2	0.187
958720315	N/A	N/A	0.187
270483843	ARHGAP11A	Rho GTPase activating protein 11A	0.187
564384443	EIF4ENIF1	eukaryotic translation initiation factor 4E nuclear import factor 1	0.187
795203979	N/A	N/A	0.187
157817558	JAKMIP2	janus kinase and microtubule interacting protein 2	0.187
148693758	N/A	N/A	0.188
167555101	STRADB	STE20-related kinase adaptor beta	0.188

395824794	N/A	N/A	0.188
58652154	TRIM26	tripartite motif containing 26	0.188
148670058	PRMT6	protein arginine methyltransferase 6	0.188
66730335	SUMO3	small ubiquitin-like modifier 3	0.188
8393959	PIM1	Pim-1 proto-oncogene, serine/threonine kinase	0.188
56971807	PARL	presenilin associated rhomboid like	0.189
564333605	BTAF1	B-TFIID TATA-box binding protein associated factor 1	0.189
157823683	HDDC2	HD domain containing 2	0.189
149064951	DYNC1I1	dynein cytoplasmic 1 intermediate chain 1	0.189
281604121	C11orf58	chromosome 11 open reading frame 58	0.189
537273922	N/A	N/A	0.189
48040531	RNF114	ring finger protein 114	0.189
403310688	USP24	ubiquitin specific peptidase 24	0.189
50511227	ZBTB34	zinc finger and BTB domain containing 34	0.190
724804431	N/A	N/A	0.190
157819605	EPC2	enhancer of polycomb homolog 2	0.190
149047323	ZNF518B	zinc finger protein 518B	0.190
300796253	ALDH1L2	aldehyde dehydrogenase 1 family member L2	0.190
157823447	MFHAS1	malignant fibrous histiocytoma amplified sequence 1	0.191
149015884	ZNF407	zinc finger protein 407	0.191
19705519	AAGAB	alpha and gamma adaptin binding protein	0.191
148703547	CNOT7	CCR4-NOT transcription complex subunit 7	0.191
672085227	USP10	ubiquitin specific peptidase 10	0.191
148683335	SLC25A44	solute carrier family 25 member 44	0.191
157822221	NSUN2	NOP2/Sun RNA methyltransferase family member 2	0.191
67078422	TMX1	thioredoxin related transmembrane protein 1	0.191
586975096	N/A	N/A	0.192
72004267	AKIRIN1	akirin 1	0.192
29789319	CBLB	Cbl proto-oncogene B	0.192
672067124	N/A	N/A	0.192
300795677	IL12RB2	interleukin 12 receptor subunit beta 2	0.192
149063727	P2RX2	purinergic receptor P2X 2	0.192
197313795	MTX1	metaxin 1	0.192
564393080	WDR33	WD repeat domain 33	0.193
62079229	PDSS2	decaprenyl diphosphate synthase subunit 2	0.193
6978635	CD59	CD59 molecule (CD59 blood group)	0.193
149047360	N/A	N/A	0.193

403310664	KMT2E	lysine methyltransferase 2E	0.194
392332910	TP53BP2	tumor protein p53 binding protein 2	0.194
140971918	Hnrnpab	heterogeneous nuclear ribonucleoprotein A/B	0.194
157823197	NDUFB7	NADH:ubiquinone oxidoreductase subunit B7	0.194
348041394	PSMD5	proteasome 26S subunit, non-ATPase 5	0.194
913513476	N/A	N/A	0.195
564350633	SLC35A1	solute carrier family 35 member A1	0.195
281427139	TADA2B	transcriptional adaptor 2B	0.195
672078533	N/A	N/A	0.195
755470479	N/A	N/A	0.195
62078579	NUB1	negative regulator of ubiquitin like proteins 1	0.195
157818041	YEATS2	YEATS domain containing 2	0.195
157822191	MTMR2	myotubularin related protein 2	0.195
296489017	BEND5	BEN domain containing 5	0.196
149067040	LIN7A	lin-7 homolog A, crumbs cell polarity complex component	0.196
157823639	PPP1R13B	protein phosphatase 1 regulatory subunit 13B	0.196
9507235	UGT8	UDP glycosyltransferase 8	0.196
17105340	GMPR	guanosine monophosphate reductase	0.197
148674377	OSER1	oxidative stress responsive serine rich 1	0.197
987939967	N/A	N/A	0.197
157821953	NXPE3	neurexophilin and PC-esterase domain family member 3	0.197
157820969	SBNO2	strawberry notch homolog 2	0.197
157820401	ABHD2	abhydrolase domain containing 2	0.197
327358533	N/A	N/A	0.197
60360230	EZH2	enhancer of zeste 2 polycomb repressive complex 2 subunit	0.197
157820491	SESN1	sestrin 1	0.197
148674304	RPRD1B	regulation of nuclear pre-mRNA domain containing 1B	0.198
157821589	THAP6	THAP domain containing 6	0.198
171846592	GPBP1	GC-rich promoter binding protein 1	0.198
537238017	N/A	N/A	0.198
795445199	N/A	N/A	0.199
947230375	N/A	N/A	0.199
347800639	GFER	growth factor, augmenter of liver regeneration	0.199
148704607	TIMM9	translocase of inner mitochondrial membrane 9	0.199
57164107	NIPSNAP3A	nipsnap homolog 3A	0.199
157817801	ADCK1	aarF domain containing kinase 1	0.199

448824835	Mff	mitochondrial fission factor	0.199
68163459	JOSD1	Josephin domain containing 1	0.199
568965596	GTF3C6	general transcription factor IIIC subunit 6	0.199
15375324	ABCD2	ATP binding cassette subfamily D member 2	0.199
725566074	N/A	N/A	0.199
213511844	ALG2	ALG2, alpha-1,3/1,6-mannosyltransferase	0.200
672048604	PHTF2	putative homeodomain transcription factor 2	0.200
672075467	MTF2	metal response element binding transcription factor 2	0.200
28174943	RPL24	ribosomal protein L24	0.200
26350839	AP3S2	adaptor related protein complex 3 sigma 2 subunit	0.200
81295375	SLC35B2	solute carrier family 35 member B2	0.200
58865352	NUBP2	nucleotide binding protein 2	0.200
829785807	N/A	N/A	0.201
157821415	GZF1	GDNF inducible zinc finger protein 1	0.201
672086679	N/A	N/A	0.201
635090844	N/A	N/A	0.201
148667150	ZNF22	zinc finger protein 22	0.202
157821579	BICD1	BICD cargo adaptor 1	0.202
77797839	UBXN1	UBX domain protein 1	0.202
472375531	N/A	N/A	0.202
84781688	GAS8	growth arrest specific 8	0.202
149069422	RPL7L1	ribosomal protein L7 like 1	0.202
190360731	TMEM167B	transmembrane protein 167B	0.203
208973268	URB2	URB2 ribosome biogenesis 2 homolog (S. cerevisiae)	0.203
14277700	RPS12	ribosomal protein S12	0.203
16758392	MGAT2	mannosyl (alpha-1,6-)-glycoprotein beta-1,2-N-acetylglucosaminyltransferase	0.203
281604227	CEP104	centrosomal protein 104	0.203
149057558	ABHD13	abhydrolase domain containing 13	0.203
56090371	FAM210A	family with sequence similarity 210 member A	0.203
148674145	CHMP4B	charged multivesicular body protein 4B	0.203
219277692	NDUFB2	NADH:ubiquinone oxidoreductase subunit B2	0.203
4506681	RPS11	ribosomal protein S11	0.203
672031398	ANKRD11	ankyrin repeat domain 11	0.203
157818001	GFOD2	glucose-fructose oxidoreductase domain containing 2	0.203
755548745	N/A	N/A	0.204

157823465	CHSY1	chondroitin sulfate synthase 1	0.204
432091578	N/A	N/A	0.204
157817288	C18orf25	chromosome 18 open reading frame 25	0.204
61557021	BFAR	bifunctional apoptosis regulator	0.204
823419445	N/A	N/A	0.204
564384353	SH3BP2	SH3 domain binding protein 2	0.204
145558904	EML1	echinoderm microtubule associated protein like 1	0.205
148702333	DDX42	DEAD-box helicase 42	0.205
672062795	N/A	N/A	0.205
187469679	LDB1	LIM domain binding 1	0.205
926702009	N/A	N/A	0.205
672046840	UBOX5	U-box domain containing 5	0.206
564383487	SLAIN2	SLAIN motif family member 2	0.206
564370654	ZNF263	zinc finger protein 263	0.206
293341722	N/A	N/A	0.206
149025942	Gar1	GAR1 ribonucleoprotein	0.206
300794743	TSC22D2	TSC22 domain family member 2	0.206
568977804	ATAD2B	ATPase family, AAA domain containing 2B	0.206
258614012	PSMB8	proteasome subunit beta 8	0.206
157817674	ATP5MF-PTCD1	ATP5MF-PTCD1 readthrough	0.207
672024181	OBSL1	obscurin like 1	0.207
564305043	RBM12B	RNA binding motif protein 12B	0.207
564377460	N/A	N/A	0.207
40018540	DDX24	DEAD-box helicase 24	0.208
513021588	N/A	N/A	0.208
157822231	ZCCHC11	zinc finger CCHC-type containing 11	0.209
157822387	KLHL28	kelch like family member 28	0.209
27229022	2610002M06Rik	RIKEN cDNA 2610002M06 gene	0.209
971393650	MOB1B	MOB kinase activator 1B	0.209
672044177	N/A	N/A	0.210
157786600	RNF145	ring finger protein 145	0.210
149042879	N/A	N/A	0.210
926686523	N/A	N/A	0.210
61557082	TERF2IP	TERF2 interacting protein	0.211
61556748	TSPYL1	TSPY like 1	0.211
674076364	N/A	N/A	0.211
574584811	TUBB4A	tubulin beta 4A class IVa	0.211
210147441	ATXN7L3B	ataxin 7 like 3B	0.211

149030718	PIP5K1A	phosphatidylinositol-4-phosphate 5-kinase type 1 alpha	0.211
37359818	KCTD5	potassium channel tetramerization domain containing 5	0.212
310616720	DHX37	DEAH-box helicase 37	0.212
157823667	UTP15	UTP15, small subunit processome component	0.212
109470195	TNKS1BP1	tankyrase 1 binding protein 1	0.213
57526818	TMEM204	transmembrane protein 204	0.213
207318	TMSB10/TMSB4X	thymosin beta 4, X-linked	0.213
392337823	RSF1	remodeling and spacing factor 1	0.214
545215595	N/A	N/A	0.214
564320608	SEMA6A	semaphorin 6A	0.214
13928842	ZNF148	zinc finger protein 148	0.214
58865776	TRIM32	tripartite motif containing 32	0.214
58865624	NUF2	NUF2, NDC80 kinetochore complex component	0.214
392340953	ITSN2	intersectin 2	0.214
157822663	RAB22A	RAB22A, member RAS oncogene family	0.214
672034794	N/A	N/A	0.215
67078512	SNX15	sorting nexin 15	0.215
84781638	KLHL25	kelch like family member 25	0.215
511905488	N/A	N/A	0.215
157819363	ZNF282	zinc finger protein 282	0.215
859770113	N/A	N/A	0.216
639869	CHKA	choline kinase alpha	0.216
157818439	NHLRC2	NHL repeat containing 2	0.216
189163477	SCAF4	SR-related CTD associated factor 4	0.216
124249254	ZNF639	zinc finger protein 639	0.217
396080328	ADCYAP1R1	ADCYAP receptor type I	0.217
66911118	NFX1	nuclear transcription factor, X-box binding 1	0.217
24025618	DAB1	DAB1, reelin adaptor protein	0.217
564334521	CACUL1	CDK2 associated cullin domain 1	0.218
537216032	N/A	N/A	0.218
998662027	N/A	N/A	0.218
209954626	TAF2	TATA-box binding protein associated factor 2	0.218
564370219	LPIN2	lipin 2	0.218
213688373	GADD45GIP1	GADD45G interacting protein 1	0.218
56090383	TMEM43	transmembrane protein 43	0.218
300796732	ZNF445	zinc finger protein 445	0.219
8393855	NUP54	nucleoporin 54	0.219

148696021	N/A	N/A	0.219
765099233	LMNB2	lamin B2	0.219
157817121	TCTE1	t-complex-associated-testis-expressed 1	0.219
281604112	BAZ1A	bromodomain adjacent to zinc finger domain 1A	0.219
149045074	NUP153	nucleoporin 153	0.219
13027450	SENP2	SUMO1/sentrin/SMT3 specific peptidase 2	0.220
293343541	ICE1	interactor of little elongation complex ELL subunit 1	0.220
24638440	RIMS4	regulating synaptic membrane exocytosis 4	0.220
751361196	N/A	N/A	0.220
512957927	N/A	N/A	0.221
171916115	LRRC55	leucine rich repeat containing 55	0.221
157820985	NCK1	NCK adaptor protein 1	0.221
2804296	CDH8	cadherin 8	0.221
6678315	TSC22D1	TSC22 domain family member 1	0.221
42627869	VKORC1L1	vitamin K epoxide reductase complex subunit 1 like 1	0.221
149030883	GSS	glutathione synthetase	0.222
71051128	ANKRD10	ankyrin repeat domain 10	0.222
564355517	CMPK2	cytidine/uridine monophosphate kinase 2	0.222
288541353	CMTM4	CKLF like MARVEL transmembrane domain containing 4	0.222
74145693	N/A	N/A	0.222
724892384	N/A	N/A	0.222
148669751	SMNDC1	survival motor neuron domain containing 1	0.223
564340181	SETX	senataxin	0.223
293340917	C3orf70	chromosome 3 open reading frame 70	0.223
568992461	DIP2B	disco interacting protein 2 homolog B	0.223
568992461	DIP2B	disco interacting protein 2 homolog B	0.223
21902533	ZNF394	zinc finger protein 394	0.223
149064227	DMXL1	Dmx like 1	0.223
125988381	JMJD6	arginine demethylase and lysine hydroxylase	0.223
77917554	SLC39A9	solute carrier family 39 member 9	0.223
197252056	MED1	mediator complex subunit 1	0.223
117940043	MED22	mediator complex subunit 22	0.225
672026667	CUX1	cut like homeobox 1	0.225
51871603	ST7	suppression of tumorigenicity 7	0.225
405113035	E2F4	E2F transcription factor 4	0.225
677444634	N/A	N/A	0.225

189181698	ZNF131	zinc finger protein 131	0.225
149033481	ZNF212	zinc finger protein 212	0.225
958729596	N/A	N/A	0.226
149065426	CASP2	caspase 2	0.226
539937	ARL14	ADP ribosylation factor like GTPase 14	0.227
148696931	ARRDC2	arrestin domain containing 2	0.227
564303058	DIDO1	death inducer-obliterator 1	0.227
157818775	AFF1	AF4/FMR2 family member 1	0.228
157823503	PLPBP	pyridoxal phosphate binding protein	0.228
300793975	ZNF286A	zinc finger protein 286A	0.228
6978787	DYRK1A	dual specificity tyrosine phosphorylation regulated kinase 1A	0.228
70794793	MAP2K7	mitogen-activated protein kinase kinase 7	0.228
19173786	SYF2	SYF2 pre-mRNA splicing factor	0.229
149043402	N/A	N/A	0.230
270483881	CBFA2T2	CBFA2/RUNX1 translocation partner 2	0.231
223555981	UBE2QL1	ubiquitin conjugating enzyme E2 Q family like 1	0.231
6978483	ALAD	aminolevulinate dehydratase	0.231
58865962	RNF41	ring finger protein 41	0.231
625182285	N/A	N/A	0.231
84781676	MTRR	5-methyltetrahydrofolate-homocysteine methyltransferase reductase	0.231
48976087	TFB1M	transcription factor B1, mitochondrial	0.231
672047736	N/A	N/A	0.232
672039306	RCOR2	REST corepressor 2	0.232
148670791	ZFYVE1	zinc finger FYVE-type containing 1	0.232
76096324	ORC6	origin recognition complex subunit 6	0.233
672073089	CCDC62	coiled-coil domain containing 62	0.233
731286412	N/A	N/A	0.233
798974764	SRRD	SRR1 domain containing	0.233
300796674	TRANK1	tetratricopeptide repeat and ankyrin repeat containing 1	0.233
11560052	DUSP12	dual specificity phosphatase 12	0.234
67975423	N/A	N/A	0.234
187937036	ZC3HC1	zinc finger C3HC-type containing 1	0.234
149032040	SLC11A2	solute carrier family 11 member 2	0.234
802996718	N/A	N/A	0.234
404351667	BOD1	biorientation of chromosomes in cell division 1	0.234
62664711	FAM69C	family with sequence similarity 69 member C	0.235

77736608	XYLT2	xylosyltransferase 2	0.235
149037637	N/A	N/A	0.235
56676358	IPPK	inositol-pentakisphosphate 2-kinase	0.235
70794766	MRPS25	mitochondrial ribosomal protein S25	0.235
524957969	N/A	N/A	0.235
148692466	N/A	N/A	0.236
564340133	GTF3C4	general transcription factor IIIC subunit 4	0.236
565303947	N/A	N/A	0.236
847038810	N/A	N/A	0.236
149067780	MVP	major vault protein	0.236
149062169	MEN1	menin 1	0.236
359718966	TOM1L1	target of myb1 like 1 membrane trafficking protein	0.236
12847552	H3F3A/H3F3B	H3 histone family member 3A	0.236
37360264	TRMT6	tRNA methyltransferase 6	0.236
67078478	NAF1	nuclear assembly factor 1 ribonucleoprotein	0.237
121583782	ZNF426	zinc finger protein 426	0.237
40018556	NOB1	NIN1/PSMD8 binding protein 1 homolog	0.237
564359486	TBC1D30	TBC1 domain family member 30	0.237
162951835	CYTH1	cytohesin 1	0.237
19173746	STK17B	serine/threonine kinase 17b	0.237
76096328	COMMD9	COMM domain containing 9	0.238
114145762	WDR83	WD repeat domain 83	0.238
13929082	PDXK	pyridoxal kinase	0.239
13928816	EIF2AK3	eukaryotic translation initiation factor 2 alpha kinase 3	0.239
158636018	LRRC6	leucine rich repeat containing 6	0.240
74195118	PPAT	phosphoribosyl pyrophosphate amidotransferase	0.240
672023059	TLN2	talin 2	0.240
672023059	TLN2	talin 2	0.240
157819927	SNRPB2	small nuclear ribonucleoprotein polypeptide B2	0.241
821013078	N/A	N/A	0.241
149023044	TMEM87A	transmembrane protein 87A	0.241
300794867	RSBN1	round spermatid basic protein 1	0.241
388596656	SLC4A1AP	solute carrier family 4 member 1 adaptor protein	0.242
62078913	OAF	out at first homolog	0.242
537151504	N/A	N/A	0.242
149039916	N/A	N/A	0.242

148704679	N/A	N/A	0.242
6981302	ODC1	ornithine decarboxylase 1	0.242
148706231	N/A	N/A	0.243
56090303	NUFIP1	NUFIP1, FMR1 interacting protein 1	0.243
157821267	RFC5	replication factor C subunit 5	0.243
19424174	DNPH1	2'-deoxynucleoside 5'-phosphate N-hydrolase 1	0.243
157819279	TNPO3	transportin 3	0.243
58865780	ZBTB17	zinc finger and BTB domain containing 17	0.243
148678808	N/A	N/A	0.244
281371443	CASTOR2	cytosolic arginine sensor for mTORC1 subunit 2	0.245
57527612	SLC17A5	solute carrier family 17 member 5	0.245
71043628	OGFRL1	opioid growth factor receptor like 1	0.245
830036530	N/A	N/A	0.245
149049946	MED4	mediator complex subunit 4	0.245
355557615	N/A	N/A	0.246
31415868	MAFB	MAF bZIP transcription factor B	0.246
157819373	FDX1L	ferredoxin 1 like	0.247
154426327	KANSL2	KAT8 regulatory NSL complex subunit 2	0.247
392333339	FAM124A	family with sequence similarity 124 member A	0.247
4885579	CNOT9	CCR4-NOT transcription complex subunit 9	0.248
157820771	ZNRF2	zinc and ring finger 2	0.248
300797934	Ranbp2	RAN binding protein 2	0.249
158631258	KDSR	3-ketodihydrosphingosine reductase	0.249
189491614	SLC25A46	solute carrier family 25 member 46	0.249
157821413	USP30	ubiquitin specific peptidase 30	0.250
672055562	LBH	limb bud and heart development	0.250
58865438	TRIP13	thyroid hormone receptor interactor 13	0.250
157819833	ZNF287	zinc finger protein 287	0.250
954521187	N/A	N/A	0.250
8923415	MARCH5	membrane associated ring-CH-type finger 5	0.250
564354018	DFFA	DNA fragmentation factor subunit alpha	0.251
157823223	ZNF330	zinc finger protein 330	0.251
392340053	FRMD4B	FERM domain containing 4B	0.251
667261609	N/A	N/A	0.251
209529662	LOC100911166/Rpusd2	RNA pseudouridylate synthase domain containing 2	0.252
291042683	DCAF5	DDB1 and CUL4 associated factor 5	0.252
256818763	PLEKHH1	pleckstrin homology, MyTH4 and FERM domain containing H1	0.252

157823719	TRAIP	TRAF interacting protein	0.252
166157540	TMEM222	transmembrane protein 222	0.253
672036088	KMT2B	lysine methyltransferase 2B	0.253
158187529	LMX1A	LIM homeobox transcription factor 1 alpha	0.253
672052951	Zfp618	zinc finger protein 618	0.254
157819449	ZBTB11	zinc finger and BTB domain containing 11	0.254
157817961	PHF3	PHD finger protein 3	0.254
209954792	PDCD2	programmed cell death 2	0.254
149060523	GPR156	G protein-coupled receptor 156	0.254
40254779	EFNB1	ephrin B1	0.255
149031125	APMAP	adipocyte plasma membrane associated protein	0.256
157821435	RANBP17	RAN binding protein 17	0.256
149063273	MPHOSPH9	M-phase phosphoprotein 9	0.256
148666908	ADAMTS9	ADAM metallopeptidase with thrombospondin type 1 motif 9	0.256
149058240	N/A	N/A	0.256
57164019	B4GALT3	beta-1,4-galactosyltransferase 3	0.256
827475660	EPC1	enhancer of polycomb homolog 1	0.256
548456234	N/A	N/A	0.256
71361655	MRPL12	mitochondrial ribosomal protein L12	0.257
77627757	IQUB	IQ motif and ubiquitin domain containing	0.257
157821283	C19orf47	chromosome 19 open reading frame 47	0.257
9506805	IL18	interleukin 18	0.257
564328896	CHD2	chromodomain helicase DNA binding protein 2	0.258
119388826	TFPT	TCF3 fusion partner	0.258
564308639	N/A	N/A	0.258
564367958	SEMA4C	semaphorin 4C	0.258
57528225	UTP4	UTP4, small subunit processome component	0.258
13928966	HSF2	heat shock transcription factor 2	0.259
672028116	TAPT1	transmembrane anterior posterior transformation 1	0.259
148709965	CUTC	cutC copper transporter	0.259
564390712	NOL8	nucleolar protein 8	0.259
84781678	NET1	neuroepithelial cell transforming 1	0.260
2252816	AXIN1	axin 1	0.260
584052040	N/A	N/A	0.260
585155919	N/A	N/A	0.260
149066961	TBC1D15	TBC1 domain family member 15	0.260
74180575	PPM1D	protein phosphatase, Mg2+/Mn2+ dependent 1D	0.260

795170747	N/A	N/A	0.261
395627633	PCP4	Purkinje cell protein 4	0.261
784639986	NIT1	nitrilase 1	0.262
157817588	TBK1	TANK binding kinase 1	0.262
281427178	CEP76	centrosomal protein 76	0.262
425384	CAMK4	calcium/calmodulin dependent protein kinase IV	0.262
6978615	CCKAR	cholecystokinin A receptor	0.263
148691080	MKX	mohawk homeobox	0.263
67078454	SLC25A51	solute carrier family 25 member 51	0.263
149043921	N/A	N/A	0.263
958722695	N/A	N/A	0.263
197382256	PHF12	PHD finger protein 12	0.263
380877082	NAXE	NAD(P)HX epimerase	0.263
148687213	COX19	COX19, cytochrome c oxidase assembly factor	0.264
675706491	N/A	N/A	0.264
40254721	AMIGO2	adhesion molecule with Ig like domain 2	0.264
564353880	DDI2	DNA damage inducible 1 homolog 2	0.264
724928464	N/A	N/A	0.265
537141832	N/A	N/A	0.266
392350322	DNAJC13	DnaJ heat shock protein family (Hsp40) member C13	0.266
8393652	KCNJ2	potassium voltage-gated channel subfamily J member 2	0.267
157820043	ZKSCAN5	zinc finger with KRAB and SCAN domains 5	0.267
197381522	ZNF879	zinc finger protein 879	0.267
157819581	SESN2	sestrin 2	0.267
31543579	RELN	reelin	0.268
244792650	TNIK	TRAF2 and NCK interacting kinase	0.268
62543511	RAB30	RAB30, member RAS oncogene family	0.269
56090325	TMEM206	transmembrane protein 206	0.269
6693638	MORC3	MORC family CW-type zinc finger 3	0.269
148696370	PANK2	pantothenate kinase 2	0.270
404312698	CASC4	cancer susceptibility 4	0.270
149056387	N/A	N/A	0.270
37360132	RHOBTB3	Rho related BTB domain containing 3	0.270
157819717	NIPA2	non imprinted in Prader-Willi/Angelman syndrome 2	0.270
564309292	N/A	N/A	0.271
149022827	PAX6	paired box 6	0.271

157823369	TERF2	telomeric repeat binding factor 2	0.272
564368081	REV1	REV1, DNA directed polymerase	0.272
564316286	CNST	consortin, connexin sorting protein	0.272
56090463	GORASP2	golgi reassembly stacking protein 2	0.273
635108195	N/A	N/A	0.273
568941588	IQSEC1	IQ motif and Sec7 domain 1	0.273
117606399	RGS16	regulator of G protein signaling 16	0.273
672030146	N/A	N/A	0.274
961745338	N/A	N/A	0.274
537211362	N/A	N/A	0.274
568923507	VAV3	vav guanine nucleotide exchange factor 3	0.274
205830446	C11orf98	chromosome 11 open reading frame 98	0.275
157819811	C21orf91	chromosome 21 open reading frame 91	0.275
281371427	NRIP1	nuclear receptor interacting protein 1	0.275
157787107	CCNG2	cyclin G2	0.276
157823125	MRPS30	mitochondrial ribosomal protein S30	0.276
564394925	PAPD5	poly(A) RNA polymerase D5, non-canonical	0.276
564365504	CCDC51	coiled-coil domain containing 51	0.277
157822475	NGDN	neuroguidin	0.277
18376839	DDIT4	DNA damage inducible transcript 4	0.277
109464919	ARHGEF26	Rho guanine nucleotide exchange factor 26	0.278
194474032	RNF19A	ring finger protein 19A, RBR E3 ubiquitin protein ligase	0.278
37360160	ATP11B	ATPase phospholipid transporting 11B (putative)	0.278
37360160	ATP11B	ATPase phospholipid transporting 11B (putative)	0.278
402794103	ATG101	autophagy related 101	0.279
148670774	N/A	N/A	0.280
59937915	ARIH2	ariadne RBR E3 ubiquitin protein ligase 2	0.280
197386048	PTRHD1	peptidyl-tRNA hydrolase domain containing 1	0.280
392349899	ACAD8	acyl-CoA dehydrogenase family member 8	0.281
149053140	PHF23	PHD finger protein 23	0.281
166091450	RPS6KB2	ribosomal protein S6 kinase B2	0.281
537134105	N/A	N/A	0.281
68341997	GPR160	G protein-coupled receptor 160	0.281
315259095	UBN1	ubinuclein 1	0.281
672023887	N/A	N/A	0.283
32451765	FBXO10	F-box protein 10	0.283
157821325	TWNK	twinkle mtDNA helicase	0.283

157821915	MSANTD3	Myb/SANT DNA binding domain containing 3	0.283
300797224	GRAMD1C	GRAM domain containing 1C	0.284
148679437	HAS3	hyaluronan synthase 3	0.284
564307173	HEATR5A	HEAT repeat containing 5A	0.284
672029178	CCSER2	coiled-coil serine rich protein 2	0.285
672029178	CCSER2	coiled-coil serine rich protein 2	0.285
29612542	H2AFZ	H2A histone family member Z	0.285
149052738	RGD1561277	RGD1561277	0.285
281599335	BMS1	BMS1, ribosome biogenesis factor	0.286
157819221	NAA30	N(alpha)-acetyltransferase 30, NatC catalytic subunit	0.286
68342017	TC2N	tandem C2 domains, nuclear	0.286
585866350	PEMT	phosphatidylethanolamine N-methyltransferase	0.286
148701441	N/A	N/A	0.287
62078733	MAK16	MAK16 homolog	0.287
672020915	VCPKMT	valosin containing protein lysine methyltransferase	0.288
157817660	EBF3	early B cell factor 3	0.288
148687364	Cux1	cut-like homeobox 1	0.288
143359181	PQLC1	PQ loop repeat containing 1	0.288
392334341	USP38	ubiquitin specific peptidase 38	0.289
56090644	ABHD6	abhydrolase domain containing 6	0.289
403310686	SOX4	SRY-box 4	0.289
51948506	AK8	adenylate kinase 8	0.290
672053062	FKBP15	FK506 binding protein 15	0.290
347921954	Pirb	paired Ig-like receptor B	0.290
213512553	CCDC43	coiled-coil domain containing 43	0.290
206734	RPL5	ribosomal protein L5	0.290
635121365	N/A	N/A	0.290
672054320	N/A	N/A	0.291
149043544	ABCG1	ATP binding cassette subfamily G member 1	0.291
157822011	TGS1	trimethylguanosine synthase 1	0.291
62088698	SPSB1	splA/ryanodine receptor domain and SOCS box containing 1	0.291
281427149	FAM69A	family with sequence similarity 69 member A	0.292
586597897	DBP	D-box binding PAR bZIP transcription factor	0.292
76559919	N4BP3	NEDD4 binding protein 3	0.292
344249173	N/A	N/A	0.292
149066670	RXYLT1	ribitol xylosyltransferase 1	0.292
204744	IGFBP3	insulin like growth factor binding protein 3	0.292

166795897	PIMREG	PICALM interacting mitotic regulator	0.293
564363988	ISLR2	immunoglobulin superfamily containing leucine rich repeat 2	0.293
149064973	ASNS	asparagine synthetase (glutamine-hydrolyzing)	0.293
13592025	PGGT1B	protein geranylgeranyltransferase type I subunit beta	0.293
149024496	SPEN	spen family transcriptional repressor	0.293
149066830	Styxl2	serine/threonine/tyrosine interacting protein-like2	0.294
672017116	N/A	N/A	0.294
148677466	N/A	N/A	0.294
201066401	RCOR3	REST corepressor 3	0.294
149031601	Hist1h1c	histone cluster 1 H1 family member c	0.294
34328151	TBR1	T-box, brain 1	0.295
157821125	COA7	cytochrome c oxidase assembly factor 7 (putative)	0.295
149026477	N/A	N/A	0.295
359324332	N/A	N/A	0.295
56789732	VSTM5	V-set and transmembrane domain containing 5	0.295
537148165	N/A	N/A	0.296
145553978	SFMBT1	Scm like with four mbt domains 1	0.297
157818273	CDC42EP4	CDC42 effector protein 4	0.297
8394196	NTM	neurotrimin	0.297
68163385	GPATCH4	G-patch domain containing 4	0.297
157817213	C16orf91	chromosome 16 open reading frame 91	0.297
157821997	MED28	mediator complex subunit 28	0.297
918611848	N/A	N/A	0.299
74220037	FAM107B	family with sequence similarity 107 member B	0.299
58866018	BIN2	bridging integrator 2	0.299
157822519	CBLN4	cerebellin 4 precursor	0.299
564327642	CEP89	centrosomal protein 89	0.300
61097926	RYK	receptor-like tyrosine kinase	0.300
13929168	FAT1	FAT atypical cadherin 1	0.300
62078947	MOSPD1	motile sperm domain containing 1	0.301
148683687	RHBDL3	rhomboid like 3	0.301
157822367	PUS3	pseudouridylate synthase 3	0.301
60223061	TERF1	telomeric repeat binding factor 1	0.301
982920935	DLX6	distal-less homeobox 6	0.301
51948532	TBC1D20	TBC1 domain family member 20	0.302
157819365	TBC1D25	TBC1 domain family member 25	0.302

7339838	SUV39H1	suppressor of variegation 3-9 homolog 1	0.302
528768520	N/A	N/A	0.302
537238000	N/A	N/A	0.302
300798394	NPHP3	nephrocystin 3	0.303
13928740	RGN	regucalcin	0.303
73621376	PDZK1	PDZ domain containing 1	0.303
61557316	ST3GAL1	ST3 beta-galactoside alpha-2,3-sialyltransferase 1	0.303
293349725	AMER3	APC membrane recruitment protein 3	0.303
56605644	TFB2M	transcription factor B2, mitochondrial	0.303
187937148	CXorf38	chromosome X open reading frame 38	0.303
13324708	ZBTB10	zinc finger and BTB domain containing 10	0.303
672084304	N/A	N/A	0.303
300795716	CCDC77	coiled-coil domain containing 77	0.303
285002227	SH3BP1	SH3 domain binding protein 1	0.304
625246380	N/A	N/A	0.304
28212232	GNL3	G protein nucleolar 3	0.305
29789082	COIL	coilin	0.305
148678764	MED21	mediator complex subunit 21	0.305
148702008	N/A	N/A	0.305
300796434	FOXRED2	FAD dependent oxidoreductase domain containing 2	0.305
13592081	SCTR	secretin receptor	0.306
56605628	SFT2D1	SFT2 domain containing 1	0.306
157818733	ZBTB2	zinc finger and BTB domain containing 2	0.306
157819315	OSBPL11	oxysterol binding protein like 11	0.306
672042113	N/A	N/A	0.307
157822893	IMP3	IMP3, U3 small nucleolar ribonucleoprotein	0.307
672017641	N/A	N/A	0.308
62078701	DIEXF	digestive organ expansion factor homolog (zebrafish)	0.308
6680532	KCNJ3	potassium voltage-gated channel subfamily J member 3	0.308
300797262	BRPF1	bromodomain and PHD finger containing 1	0.308
6978497	AMBP	alpha-1-microglobulin/bikunin precursor	0.308
300798436	NME6	NME/NM23 nucleoside diphosphate kinase 6	0.308
51491900	TOR1A	torsin family 1 member A	0.308
537250304	N/A	N/A	0.308
672059756	N/A	N/A	0.309
56605776	TAF11	TATA-box binding protein associated factor 11	0.309

349501022	2410002F23Rik	RIKEN cDNA 2410002F23 gene	0.309
72255513	AGA	aspartylglucosaminidase	0.309
50510821	AMIGO1	adhesion molecule with Ig like domain 1	0.310
755532277	Lrrfip2	leucine rich repeat (in FLII) interacting protein 2	0.311
300798014	ICE2	interactor of little elongation complex ELL subunit 2	0.311
672043577	Rprd2	regulation of nuclear pre-mRNA domain containing 2	0.312
109480728	TMEM74	transmembrane protein 74	0.313
109480728	TMEM74	transmembrane protein 74	0.313
157819887	LACTB	lactamase beta	0.314
149048116	KHDC4	KH domain containing 4, pre-mRNA splicing factor	0.314
293351303	METTL22	methyltransferase like 22	0.315
148707009	HMGCS2	3-hydroxy-3-methylglutaryl-CoA synthase 2	0.315
62078923	DZIP1L	DAZ interacting zinc finger protein 1 like	0.315
157816943	MCM8	minichromosome maintenance 8 homologous recombination repair factor	0.316
33086606	SRPRB	SRP receptor beta subunit	0.318
148676796	N/A	N/A	0.319
408821459	STYX	serine/threonine/tyrosine interacting protein	0.319
672017463	N/A	N/A	0.320
884934330	N/A	N/A	0.321
300793780	ZNF251	zinc finger protein 251	0.321
149036607	N/A	N/A	0.321
724851156	N/A	N/A	0.321
34882672	ETAA1	ETAA1, ATR kinase activator	0.322
149051028	RNF144A	ring finger protein 144A	0.322
17865345	CDH23	cadherin related 23	0.322
68534262	C1orf43	chromosome 1 open reading frame 43	0.322
41386747	ZC3H18	zinc finger CCCH-type containing 18	0.322
672030183	HIST1H2AH	histone cluster 1 H2A family member h	0.323
913496630	N/A	N/A	0.324
880805457	GPR161	G protein-coupled receptor 161	0.324
148681991	MIOS	meiosis regulator for oocyte development	0.325
564397086	BRPF3	bromodomain and PHD finger containing 3	0.326
672026763	ATXN2	ataxin 2	0.326
157820341	GPR63	G protein-coupled receptor 63	0.326
76362828	TEF	TEF, PAR bZIP transcription factor	0.327

50510655	PCF11	PCF11 cleavage and polyadenylation factor subunit	0.327
47155567	ARHGAP20	Rho GTPase activating protein 20	0.328
148710252	SLITRK4	SLIT and NTRK like family member 4	0.328
157820119	LRRTM1	leucine rich repeat transmembrane neuronal 1	0.328
68163537	NXPE4	neurexophilin and PC-esterase domain family member 4	0.329
564318054	R3hcc1	R3H domain and coiled-coil containing 1	0.329
404501489	DDX55	DEAD-box helicase 55	0.329
157823980	CCNT1	cyclin T1	0.330
109479851	NRDE2	NRDE-2, necessary for RNA interference, domain containing	0.330
157817260	ORAOV1	oral cancer overexpressed 1	0.330
564298767	N/A	N/A	0.331
62244083	PDRG1	p53 and DNA damage regulated 1	0.331
672063138	IP6K2	inositol hexakisphosphate kinase 2	0.332
582015198	CRY2	cryptochrome circadian regulator 2	0.332
946774489	N/A	N/A	0.332
149032986	MFSD4B	major facilitator superfamily domain containing 4B	0.332
564309026	SUN2	Sad1 and UNC84 domain containing 2	0.332
564333920	PPRC1	peroxisome proliferator-activated receptor gamma, coactivator-related 1	0.333
149064983	N/A	N/A	0.333
157824124	NUAK1	NUAK family kinase 1	0.335
349732232	NFATC1	nuclear factor of activated T cells 1	0.335
564303955	EMX1	empty spiracles homeobox 1	0.335
156627555	NT5C3B	5'-nucleotidase, cytosolic IIIB	0.335
6680007	GJC1	gap junction protein gamma 1	0.336
157820863	RPIA	ribose 5-phosphate isomerase A	0.336
158186659	CBS/CBSL	cystathionine-beta-synthase	0.339
564307081	ATXN7L1	ataxin 7 like 1	0.339
3212116	PFDN2	prefoldin subunit 2	0.340
564350836	MELK	maternal embryonic leucine zipper kinase	0.341
157824026	TCEANC	transcription elongation factor A N-terminal and central domain containing	0.341
672055128	SAMD11	sterile alpha motif domain containing 11	0.342
300794780	GPSM2	G protein signaling modulator 2	0.342
157819851	ZNF565	zinc finger protein 565	0.342
157818881	Zfp954	zinc finger protein 954	0.342

30017415	ITPKC	inositol-trisphosphate 3-kinase C	0.343
61557263	GDAP2	ganglioside induced differentiation associated protein 2	0.344
77917610	GPBP1L1	GC-rich promoter binding protein 1 like 1	0.344
199561799	LRP12	LDL receptor related protein 12	0.344
392337836	RNF169	ring finger protein 169	0.345
73990974	LZTS3	leucine zipper tumor suppressor family member 3	0.345
160333172	COG2	component of oligomeric golgi complex 2	0.346
157787079	NVL	nuclear VCP-like	0.347
291167790	Fam13a	family with sequence similarity 13, member A	0.347
164518930	SDK1	sidekick cell adhesion molecule 1	0.347
157786710	CEP112	centrosomal protein 112	0.347
564365330	CDC25A	cell division cycle 25A	0.348
625263218	N/A	N/A	0.348
672037086	N/A	N/A	0.348
157824010	MRPS31	mitochondrial ribosomal protein S31	0.348
66730433	RBM48	RNA binding motif protein 48	0.349
157786956	IL27RA	interleukin 27 receptor subunit alpha	0.350
672053077	N/A	N/A	0.351
672067227	ZNF354A	zinc finger protein 354A	0.351
564368492	CARF	calcium responsive transcription factor	0.351
149067193	Cfap54	cilia and flagella associated protein 54	0.351
141803183	ZKSCAN3	zinc finger with KRAB and SCAN domains 3	0.351
109460021	KIAA2026	KIAA2026	0.352
157821859	RNF219	ring finger protein 219	0.352
927108944	N/A	N/A	0.353
58865826	ZNF35	zinc finger protein 35	0.353
157822711	RBM28	RNA binding motif protein 28	0.353
56119156	Zfp58	zinc finger protein 58	0.353
440909886	N/A	N/A	0.354
157818251	FBXO4	F-box protein 4	0.354
76096340	ANKRD16	ankyrin repeat domain 16	0.354
281604129	HELQ	helicase, POLQ like	0.354
392306987	N/A	N/A	0.355
532034304	N/A	N/A	0.355
40018598	ANGPTL4	angiopoietin like 4	0.356
672065395	CCNYL1	cyclin Y like 1	0.356
672018101	N/A	N/A	0.356
148673748	FAM110B	family with sequence similarity 110 member B	0.357

672053428	LRP8	LDL receptor related protein 8	0.358
201066365	PAPSS2	3'-phosphoadenosine 5'-phosphosulfate synthase 2	0.359
348041294	Spata24	spermatogenesis associated 24	0.359
564393142	WDR36	WD repeat domain 36	0.359
157820017	FBXO33	F-box protein 33	0.359
57528321	RIOK2	RIO kinase 2	0.360
392342123	ALS2CL	ALS2 C-terminal like	0.360
74183022	Zfp773	zinc finger protein 773	0.360
564344754	PMEPA1	prostate transmembrane protein, androgen induced 1	0.360
37360398	ISLR2	immunoglobulin superfamily containing leucine rich repeat 2	0.360
88853859	UBE3D	ubiquitin protein ligase E3D	0.361
281332148	RIOK1	RIO kinase 1	0.361
157822721	NUPL2	nucleoporin like 2	0.361
157822027	CSRNP2	cysteine and serine rich nuclear protein 2	0.361
755542850	RGS6	regulator of G protein signaling 6	0.361
58865996	TRIM13	tripartite motif containing 13	0.361
149056682	N/A	N/A	0.361
300794219	OPN3	opsin 3	0.362
62543527	TGIF1	TGFB induced factor homeobox 1	0.362
6981048	HSD17B1	hydroxysteroid 17-beta dehydrogenase 1	0.363
319996608	Spin2c	spindlin family, member 2C	0.363
77627740	ING3	inhibitor of growth family member 3	0.363
62642955	NIM1K	NIM1 serine/threonine protein kinase	0.364
148675659	CSDE1	cold shock domain containing E1	0.364
672069572	KANSL1	KAT8 regulatory NSL complex subunit 1	0.364
300797828	KAT14	lysine acetyltransferase 14	0.365
406362836	HS6ST3	heparan sulfate 6-O-sulfotransferase 3	0.365
148710078	TAF5	TATA-box binding protein associated factor 5	0.366
564395350	N/A	N/A	0.366
157817592	HEXIM2	hexamethylene bisacetamide inducible 2	0.366
586989396	N/A	N/A	0.367
672055229	N/A	N/A	0.368
564311478	KIAA1211L	KIAA1211 like	0.369
209529638	RSBN1L	round spermatid basic protein 1 like	0.369
238859603	ISLR2	immunoglobulin superfamily containing leucine rich repeat 2	0.369
926682956	N/A	N/A	0.369

149067053	LRRIQ1	leucine rich repeats and IQ motif containing 1	0.370
74143776	INIP	INTS3 and NABP interacting protein	0.371
350540004	CENPW	centromere protein W	0.371
148704240	ZMYM2	zinc finger MYM-type containing 2	0.371
157821875	PTCD2	pentatricopeptide repeat domain 2	0.372
157820949	SAMD5	sterile alpha motif domain containing 5	0.372
884872268	N/A	N/A	0.372
672036551	ZDHHC13	zinc finger DHHC-type containing 13	0.373
672042720	N/A	N/A	0.373
157817720	SLC16A14	solute carrier family 16 member 14	0.374
149048326	N/A	N/A	0.374
148671273	N/A	N/A	0.375
410960574	N/A	N/A	0.376
62078501	TTI2	TELO2 interacting protein 2	0.376
16758666	TIMP1	TIMP metallopeptidase inhibitor 1	0.376
157821747	MDM2	MDM2 proto-oncogene	0.377
158508524	DDIT3	DNA damage inducible transcript 3	0.377
213972545	MXD1	MAX dimerization protein 1	0.378
564326636	Zfp94	zinc finger protein 94	0.378
40538878	NEXN	nexilin F-actin binding protein	0.379
157822681	EFNB2	ephrin B2	0.379
164565364	ITPKB	inositol-trisphosphate 3-kinase B	0.380
564338579	SASS6	SAS-6 centriolar assembly protein	0.381
564323985	LOC108348337	uncharacterized LOC108348337	0.382
568997192	PRDM15	PR/SET domain 15	0.383
17105346	PLSCR1	phospholipid scramblase 1	0.383
14388593	SPATA2	spermatogenesis associated 2	0.385
564382837	LIN54	lin-54 DREAM MuvB core complex component	0.387
56605820	PAPD4	poly(A) RNA polymerase D4, non-canonical	0.387
970744322	N/A	N/A	0.388
149037585	N/A	N/A	0.389
564352668	MYCL	MYCL proto-oncogene, bHLH transcription factor	0.392
51592090	CCR5	C-C motif chemokine receptor 5 (gene/pseudogene)	0.393
171846573	FBXL4	F-box and leucine rich repeat protein 4	0.393
59709429	ZSCAN21	zinc finger and SCAN domain containing 21	0.395
672019438	FKBP15	FK506 binding protein 15	0.395
157823389	TRMT9B	tRNA methyltransferase 9B (putative)	0.395

404501518	ZNF569	zinc finger protein 569	0.396
564321498	ZFP1	ZFP1 zinc finger protein	0.396
148681067	VASH2	vasohibin 2	0.397
8392855	ADCYAP1	adenylate cyclase activating polypeptide 1	0.397
40789237	PCDHA4	protocadherin alpha 4	0.398
149025439	DICER1	dicer 1, ribonuclease III	0.398
198278467	DCUN1D2	defective in cullin neddylation 1 domain containing 2	0.398
157821403	RASSF7	Ras association domain family member 7	0.399
148664478	N/A	N/A	0.400
16758238	SPA17	sperm autoantigenic protein 17	0.402
67078450	OSGEPL1	O-sialoglycoprotein endopeptidase like 1	0.402
11560016	HTR1B	5-hydroxytryptamine receptor 1B	0.402
564318492	FAM208A	family with sequence similarity 208 member A	0.403
157817446	LINGO2	leucine rich repeat and Ig domain containing 2	0.403
23097354	FADD	Fas associated via death domain	0.403
148695049	FIGN	fidgetin, microtubule severing factor	0.403
51948522	PLA2G15	phospholipase A2 group XV	0.404
282158061	Ttc41	tetratricopeptide repeat domain 41	0.404
62945262	PIK3IP1	phosphoinositide-3-kinase interacting protein 1	0.404
61889068	MXI1	MAX interactor 1, dimerization protein	0.405
157819301	ZNF777	zinc finger protein 777	0.406
148747270	PTGS2	prostaglandin-endoperoxide synthase 2	0.408
293345066	PPIL6	peptidylprolyl isomerase like 6	0.408
226371633	CABLES1	Cdk5 and Abl enzyme substrate 1	0.409
157817845	KMT5B	lysine methyltransferase 5B	0.413
16758574	CNTN5	contactin 5	0.414
66730347	PTPRCAP	protein tyrosine phosphatase, receptor type C associated protein	0.415
13928944	P2RY4	pyrimidinergic receptor P2Y4	0.415
564351113	N/A	N/A	0.415
67846052	DCUN1D3	defective in cullin neddylation 1 domain containing 3	0.416
672036241	RGD1584023/Zfp939	similar to zinc finger protein 11B	0.419
148688049	N/A	N/A	0.419
157821351	EXO1	exonuclease 1	0.420
14861862	CRYGD	crystallin gamma D	0.421
293348214	CCDC88C	coiled-coil domain containing 88C	0.421
955485868	N/A	N/A	0.422

68163435	MBLAC1	metallo-beta-lactamase domain containing 1	0.423
564318578	ANKRD28	ankyrin repeat domain 28	0.424
38454286	TMEM110-MUSTN1	TMEM110-MUSTN1 readthrough	0.425
188595675	RFX7	regulatory factor X7	0.426
444741673	CYHR1	cysteine and histidine rich 1	0.426
672054464	Srrm1	serine/arginine repetitive matrix 1	0.427
350591922	N/A	N/A	0.427
255982592	RBM20	RNA binding motif protein 20	0.427
568997192	PRDM15	PR/SET domain 15	0.429
56090421	PXYLP1	2-phosphoxylose phosphatase 1	0.429
564362348	Fam76b	family with sequence similarity 76, member B	0.429
564316243	CEP170	centrosomal protein 170	0.430
157819193	DOK5	docking protein 5	0.430
56090305	NFATC2IP	nuclear factor of activated T cells 2 interacting protein	0.430
537166446	N/A	N/A	0.430
625275780	N/A	N/A	0.430
672087260	N/A	N/A	0.431
25742816	TIMM17A	translocase of inner mitochondrial membrane 17A	0.431
56090289	PELO	pelota mRNA surveillance and ribosome rescue factor	0.432
564303928	TET3	tet methylcytosine dioxygenase 3	0.432
564322442	Kdm6a	lysine demethylase 6A	0.432
157819993	CCDC112	coiled-coil domain containing 112	0.433
129772	PENK	proenkephalin	0.433
293348129	DACT1	dishevelled binding antagonist of beta catenin 1	0.433
564395313	OTUD4	OTU deubiquitinase 4	0.434
392345518	SENP5	SUMO1/sentrin specific peptidase 5	0.434
625269293	N/A	N/A	0.435
56090592	EMP2	epithelial membrane protein 2	0.436
672031975	LOC299312	similar to G protein-binding protein CRFG	0.436
926720695	N/A	N/A	0.437
157823891	ING2	inhibitor of growth family member 2	0.439
74196108	NECTIN4	nectin cell adhesion molecule 4	0.442
62078765	Zfp819	zinc finger protein 819	0.442
564352534	Szt2	SZT2, KICSTOR complex subunit	0.443
58865998	PCDHGB7	protocadherin gamma subfamily B, 7	0.443
564311452	TMEM131	transmembrane protein 131	0.443

821394612	N/A	N/A	0.444
893846521	MARCH11	membrane associated ring-CH-type finger 11	0.445
564335481	FYB1	FYN binding protein 1	0.446
884914541	N/A	N/A	0.450
3676248	Prim1	DNA primase subunit 1	0.450
157817047	NEDD1	neural precursor cell expressed, developmentally down-regulated 1	0.451
880954369	N/A	N/A	0.451
293341533	LOC108348225	feline leukemia virus subgroup C receptor-related protein 1	0.451
51980294	COQ3	coenzyme Q3, methyltransferase	0.452
157820727	RPL27A	ribosomal protein L27a	0.452
672045396	N/A	N/A	0.452
524983306	N/A	N/A	0.453
149066425	A930017M01Rik	Smg-5 homolog, nonsense mediated mRNA decay factor pseudogene	0.454
76881802	KCNG3	potassium voltage-gated channel modifier subfamily G member 3	0.454
60360636	GAREM1	GRB2 associated regulator of MAPK1 subtype 1	0.454
755537242	CLK4	CDC like kinase 4	0.456
53850630	LOC100362724/MGC95208	similar to 4930453N24Rik protein	0.456
149041559	BUD13	BUD13 homolog	0.456
157818699	TSEN54	tRNA splicing endonuclease subunit 54	0.458
845633640	TSSC4	tumor suppressing subtransferable candidate 4	0.458
67078462	SOX18	SRY-box 18	0.460
126722629	HSPBAP1	HSPB1 associated protein 1	0.460
8392993	BMP3	bone morphogenetic protein 3	0.461
157819737	SARS2	seryl-tRNA synthetase 2, mitochondrial	0.463
655889411	N/A	N/A	0.463
564317714	Ktn1	kinectin 1	0.463
157822327	ATG14	autophagy related 14	0.464
564382848	Hnrnpdl	heterogeneous nuclear ribonucleoprotein D-like	0.464
21955138	RAB38	RAB38, member RAS oncogene family	0.465
884945546	N/A	N/A	0.465
8394142	RAB27A	RAB27A, member RAS oncogene family	0.466
255708448	KATNA1	katanin catalytic subunit A1	0.466
157818819	TMEM144	transmembrane protein 144	0.466
157817797	PDCD2L	programmed cell death 2 like	0.468

564347830	ZXDC	ZXD family zinc finger C	0.468
7949105	PBX3	PBX homeobox 3	0.468
148706598	PKDCC	protein kinase domain containing, cytoplasmic	0.469
114145706	LOC499407	LRRGT00097	0.469
51948492	NUDT19	nudix hydrolase 19	0.470
66730445	LZTFL1	leucine zipper transcription factor like 1	0.472
68163485	WDYHV1	WDYHV motif containing 1	0.472
672061753	N/A	N/A	0.474
117940029	ZC3H12A	zinc finger CCCH-type containing 12A	0.474
198278471	ZBED4	zinc finger BED-type containing 4	0.476
213385320	LRTOMT	leucine rich transmembrane and O-methyltransferase domain containing	0.476
70608121	Dmrtc1a	DMRT-like family C1a	0.479
70912374	CCNQ	cyclin Q	0.479
672047066	CEP152	centrosomal protein 152	0.480
676284727	N/A	N/A	0.481
157821565	MTHFD2L	methylenetetrahydrofolate dehydrogenase (NADP+ dependent) 2 like	0.482
17530969	SLC8A3	solute carrier family 8 member A3	0.483
672055181	VIT	vitrin	0.484
803269187	N/A	N/A	0.485
149024753	DFFB	DNA fragmentation factor subunit beta	0.487
564299234	Lcor	ligand dependent nuclear receptor corepressor	0.488
149053039	KCNAB3	potassium voltage-gated channel subfamily A regulatory beta subunit 3	0.488
817337812	N/A	N/A	0.488
564361059	CYTH4	cytohesin 4	0.489
281306771	ADAMTS4	ADAM metallopeptidase with thrombospondin type 1 motif 4	0.489
148689230	MAPKAPK3	mitogen-activated protein kinase-activated protein kinase 3	0.490
56090445	PHOSPHO2	phosphatase, orphan 2	0.490
564347830	ZXDC	ZXD family zinc finger C	0.490
568970985	MBTD1	mbt domain containing 1	0.491
62078749	C7orf25	chromosome 7 open reading frame 25	0.493
672061705	KMT2A	lysine methyltransferase 2A	0.498
11560065	GPR85	G protein-coupled receptor 85	0.502
76096320	USHBP1	USH1 protein network component harmonin binding protein 1	0.504
212549645	KIF18A	kinesin family member 18A	0.505

157817521	Oacyl	O-acyltransferase like	0.505
672084062	TEPP	testis, prostate and placenta expressed	0.510
149017375	ARAP3	ArfGAP with RhoGAP domain, ankyrin repeat and PH domain 3	0.513
300798653	ALPK3	alpha kinase 3	0.515
240255436	T2	brachyury 2	0.515
672057962	N/A	N/A	0.517
157821277	HAUS3	HAUS augmin like complex subunit 3	0.518
19424300	GCHFR	GTP cyclohydrolase I feedback regulator	0.519
54312090	RBM47	RNA binding motif protein 47	0.522
195539325	MTBP	MDM2 binding protein	0.523
537271325	N/A	N/A	0.524
89145411	SULT2B1	sulfotransferase family 2B member 1	0.524
149066158	ZNF623	zinc finger protein 623	0.528
62078463	LPP	LIM domain containing preferred translocation partner in lipoma	0.528
955534901	N/A	N/A	0.530
404434380	ZNF133	zinc finger protein 133	0.530
77627983	MLX	MLX, MAX dimerization protein	0.531
112984176	EPSTI1	epithelial stromal interaction 1	0.532
4507133	SNRPG	small nuclear ribonucleoprotein polypeptide G	0.533
21955136	MXD3	MAX dimerization protein 3	0.535
564320728	Fbxo38	F-box protein 38	0.537
148673911	Gm21596/Hmgb1	high mobility group box 1	0.538
205830438	MYH1	myosin heavy chain 1	0.541
672056683	N/A	N/A	0.541
157821317	Ifitm1	interferon induced transmembrane protein 1	0.545
306482607	DENND2C	DENN domain containing 2C	0.547
672026767	N/A	N/A	0.549
148687591	TMEM132D	transmembrane protein 132D	0.550
213688370	EXOSC7	exosome component 7	0.551
564315667	CLASP1	cytoplasmic linker associated protein 1	0.555
564320454	SAP130	Sin3A associated protein 130	0.559
568916876	GREM1	gremlin 1, DAN family BMP antagonist	0.559
219879771	PGAP3	post-GPI attachment to proteins 3	0.560
157820217	Gsta4	glutathione S-transferase, alpha 4	0.560
474451689	RASEF	RAS and EF-hand domain containing	0.560
13928942	PER2	period circadian regulator 2	0.562
625253043	N/A	N/A	0.562
62640766	GDPGP1	GDP-D-glucose phosphorylase 1	0.562

149025256	NGB	neuroglobin	0.563
149056609	DEDD2	death effector domain containing 2	0.565
62078827	CTDSPL2	CTD small phosphatase like 2	0.570
52851389	OSMR	oncostatin M receptor	0.570
62078983	DNAJC28	DnaJ heat shock protein family (Hsp40) member C28	0.570
149023178	CEP152	centrosomal protein 152	0.572
148692940	WAPL	WAPL cohesin release factor	0.573
672029702	ZCCHC6	zinc finger CCHC-type containing 6	0.576
157822673	HS3ST5	heparan sulfate-glucosamine 3-sulfotransferase 5	0.577
564372912	GPS2	G protein pathway suppressor 2	0.577
74186677	SIN3B	SIN3 transcription regulator family member B	0.578
71051382	Fhl4	four and a half LIM domains 4	0.581
148664537	Gm10269	ribosomal protein L35 pseudogene	0.582
149016587	N/A	N/A	0.583
555290059	MED7	mediator complex subunit 7	0.584
17105344	KLHL41	kelch like family member 41	0.585
16758872	CDH17	cadherin 17	0.585
148705473	FAM53A	family with sequence similarity 53 member A	0.585
12621078	PTPRQ	protein tyrosine phosphatase, receptor type Q	0.585
511094004	RUNX2	runt related transcription factor 2	0.585
16924020	XPNPEP2	X-prolyl aminopeptidase 2	0.585
564315183	CUX1	cut like homeobox 1	0.585
148701441	N/A	N/A	0.585
386869333	TFF3	trefoil factor 3	0.589
564295968	ADGRG6	adhesion G protein-coupled receptor G6	0.590
564386624	AMER2	APC membrane recruitment protein 2	0.591
564304579	ATF7IP	activating transcription factor 7 interacting protein	0.591
564303143	KMT2C	lysine methyltransferase 2C	0.593
157786962	NANOS3	nanos C2HC-type zinc finger 3	0.596
148705043	RRM2	ribonucleotide reductase regulatory subunit M2	0.596
62078705	STBD1	starch binding domain 1	0.601
148683584	VEPH1	ventricular zone expressed PH domain containing 1	0.603
564337450	S100A9	S100 calcium binding protein A9	0.603
537191098	N/A	N/A	0.603
344248288	N/A	N/A	0.603
24415396	GPR3	G protein-coupled receptor 3	0.604

114145465	LOC689840	LRRGT00142	0.604
21703842	RTCB	RNA 2',3'-cyclic phosphate and 5'-OH ligase	0.609
672065765	KCNE4	potassium voltage-gated channel subfamily E regulatory subunit 4	0.609
149038509	N/A	N/A	0.611
13994119	KHK	ketohexokinase	0.612
8393564	HRH1	histamine receptor H1	0.615
22122541	LRRC3B	leucine rich repeat containing 3B	0.615
203096609	GABPB1	GA binding protein transcription factor beta subunit 1	0.616
148702599	UNK	unkempt family zinc finger	0.619
672053077	N/A	N/A	0.620
157822359	PELI2	pellino E3 ubiquitin protein ligase family member 2	0.620
197386987	HDX	highly divergent homeobox	0.620
148702471	N/A	N/A	0.620
149035460	N/A	N/A	0.622
157820433	CPEB1	cytoplasmic polyadenylation element binding protein 1	0.624
187957728	FANCM	Fanconi anemia complementation group M	0.625
38016150	QRFPR	pyroglutamylated RFamide peptide receptor	0.625
148689488	SYN3	synapsin III	0.627
672068300	TOP3A	DNA topoisomerase III alpha	0.629
1083798	Bmpr1b	bone morphogenetic protein receptor type 1B	0.630
198386330	CCDC89	coiled-coil domain containing 89	0.630
149048372	N/A	N/A	0.632
149048372	N/A	N/A	0.632
149048372	N/A	N/A	0.632
197386066	ZNF784	zinc finger protein 784	0.633
564389552	LOC100910854	zinc finger MYND domain-containing protein 19-like	0.635
564303706	OSBPL3	oxysterol binding protein like 3	0.637
564339225	N/A	N/A	0.637
186659510	MYH6	myosin heavy chain 6	0.637
157823803	DOK3	docking protein 3	0.639
39104628	SORBS1	sorbin and SH3 domain containing 1	0.639
148693644	N/A	N/A	0.642
672043573	LOC100363520	mCG16729-like	0.643
564318727	Zfp961	zinc finger protein 961	0.643
564400410	AMOT	angiomotin	0.644

568907669	NYAP2	neuronal tyrosine-phosphorylated phosphoinositide-3-kinase adaptor 2	0.645
16758796	INPP4B	inositol polyphosphate-4-phosphatase type II B	0.646
672066276	N/A	N/A	0.648
281604200	COL9A1	collagen type IX alpha 1 chain	0.648
149025186	RPS6KL1	ribosomal protein S6 kinase like 1	0.661
149031102	N/A	N/A	0.662
564327667	TSHZ3	teashirt zinc finger homeobox 3	0.664
149021160	N/A	N/A	0.665
256220048	PCDHGC5	protocadherin gamma subfamily C, 5	0.666
157823385	SLITRK6	SLIT and NTRK like family member 6	0.667
913498340	N/A	N/A	0.669
704532863	N/A	N/A	0.671
112984092	RPRM	reprimo, TP53 dependent G2 arrest mediator homolog	0.675
51890226	IL17RE	interleukin 17 receptor E	0.678
71896574	Sult1c2 (includes others)	sulfotransferase family 1C member 2	0.678
676270739	N/A	N/A	0.689
149052692	N/A	N/A	0.689
149016574	ZNF324	zinc finger protein 324	0.689
157822083	PBX4	PBX homeobox 4	0.690
672013556	N/A	N/A	0.690
672015275	N/A	N/A	0.691
66730382	TRNT1	tRNA nucleotidyl transferase 1	0.692
148693601	N/A	N/A	0.692
6978894	GIPR	gastric inhibitory polypeptide receptor	0.699
112984482	SBSN	suprabasin	0.700
197387536	TEX26	testis expressed 26	0.706
61557100	PLEKHF1	pleckstrin homology and FYVE domain containing 1	0.707
348041347	CENPL	centromere protein L	0.708
392354293	Hmgb3	high mobility group box 3	0.709
675743174	N/A	N/A	0.710
149058952	MEF2C	myocyte enhancer factor 2C	0.716
149062619	CBWD1	COBW domain containing 1	0.717
80861398	CRY1	cryptochrome circadian regulator 1	0.720
672049461	N/A	N/A	0.721
148701660	FSTL4	follistatin like 4	0.724
148704285	CBLN3	cerebellin 3 precursor	0.724

281485597	PRDM9	PR/SET domain 9	0.726
148698485	N/A	N/A	0.731
672028080	PRR14L	proline rich 14 like	0.733
564389875	ARHGEF10	Rho guanine nucleotide exchange factor 10	0.734
38454200	CHDH	choline dehydrogenase	0.734
672014266	TMEM219	transmembrane protein 219	0.735
20302047	AMPD1	adenosine monophosphate deaminase 1	0.737
77539456	C4BPA	complement component 4 binding protein alpha	0.737
84781680	CNKSR1	connector enhancer of kinase suppressor of Ras 1	0.737
672059431	SLC39A4	solute carrier family 39 member 4	0.737
6681177	TWIST2	twist family bHLH transcription factor 2	0.737
6978663	CLCN1	chloride voltage-gated channel 1	0.742
157818947	FAAP24	Fanconi anemia core complex associated protein 24	0.744
731286412	N/A	N/A	0.746
564297338	ZNF816	zinc finger protein 816	0.748
293339965	RAB11FIP3	RAB11 family interacting protein 3	0.750
403420582	TMEM71	transmembrane protein 71	0.767
431895836	N/A	N/A	0.768
672070295	BAHCC1	BAH domain and coiled-coil containing 1	0.769
157817525	LGR5	leucine rich repeat containing G protein-coupled receptor 5	0.772
431916930	N/A	N/A	0.777
564298396	ZNF764	zinc finger protein 764	0.777
564358680	N/A	N/A	0.777
114145559	ARSI	arylsulfatase family member I	0.778
453178	AMHR2	anti-Mullerian hormone receptor type 2	0.778
452085169	BCO1	beta-carotene oxygenase 1	0.778
62078489	CLHC1	clathrin heavy chain linker domain containing 1	0.778
58865600	LAMP3	lysosomal associated membrane protein 3	0.778
282397098	PRR30	proline rich 30	0.778
149042882	ZNF334	zinc finger protein 334	0.779
58865510	GIMAP6	GTPase, IMAP family member 6	0.783
755566692	HUWE1	HECT, UBA and WWE domain containing 1, E3 ubiquitin protein ligase	0.787
752423229	N/A	N/A	0.789
564316247	CEP170	centrosomal protein 170	0.793
672031995	Kdm6a	lysine demethylase 6A	0.794
564297423	FAM71E1	family with sequence similarity 71 member E1	0.803

564377118	WDR53	WD repeat domain 53	0.807
6978801	CELA1	chymotrypsin like elastase family member 1	0.807
56606104	Aox4	aldehyde oxidase 4	0.807
6978747	Cyp2d26	cytochrome P450, family 2, subfamily d, polypeptide 26	0.807
149024681	N/A	N/A	0.813
568921554	Ank2	ankyrin 2, brain	0.813
300795362	PTCHD1	patched domain containing 1	0.820
293348634	LRIG3	leucine rich repeats and immunoglobulin like domains 3	0.825
568975399	FAM114A2	family with sequence similarity 114 member A2	0.827
157818453	CXorf21	chromosome X open reading frame 21	0.830
294979130	FOXP3	forkhead box P3	0.830
149025130	Fam161b	family with sequence similarity 161, member B	0.832
672086719	FAM184A	family with sequence similarity 184 member A	0.836
26024223	ABCG5	ATP binding cassette subfamily G member 5	0.841
16758770	F2RL1	F2R like trypsin receptor 1	0.842
149025439	DICER1	dicer 1, ribonuclease III	0.846
158517955	PKHD1L1	PKHD1 like 1	0.848
755528524	N/A	N/A	0.849
189011634	ARMC7	armadillo repeat containing 7	0.850
58865582	Irgm1	immunity-related GTPase family M member 1	0.856
293347270	OSGIN2	oxidative stress induced growth inhibitor family member 2	0.861
16758000	CXCR5	C-X-C motif chemokine receptor 5	0.862
564378170	PAN3	PAN3 poly(A) specific ribonuclease subunit	0.863
568959785	PRDM10	PR/SET domain 10	0.865
6978657	CHRNB1	cholinergic receptor nicotinic beta 1 subunit	0.865
471359561	N/A	N/A	0.871
121583673	CACTIN	cactin, spliceosome C complex subunit	0.872
157822171	C1orf210	chromosome 1 open reading frame 210	0.874
672058654	CAPS2	calcyphosine 2	0.874
157821967	SLC45A2	solute carrier family 45 member 2	0.874
537222639	N/A	N/A	0.880
78097110	N4BP2L1	NEDD4 binding protein 2 like 1	0.885
672025117	MBTD1	mbt domain containing 1	0.886
589930143	N/A	N/A	0.893
564302768	KIAA1755	KIAA1755	0.906
672088752	MCF2	MCF.2 cell line derived transforming sequence	0.909
157820193	Tbx2	T-box 2	0.909

672015368	MAST4	microtubule associated serine/threonine kinase family member 4	0.909
672051730	N/A	N/A	0.909
71795623	ELMO3	engulfment and cell motility 3	0.910
392339806	CFAP69	cilia and flagella associated protein 69	0.915
16758266	NME3	NME/NM23 nucleoside diphosphate kinase 3	0.921
568929584	TRIM62	tripartite motif containing 62	0.922
829796090	N/A	N/A	0.922
562838033	N/A	N/A	0.926
672051147	N/A	N/A	0.928
564378170	PAN3	PAN3 poly(A) specific ribonuclease subunit	0.932
50845391	LY6G5B	lymphocyte antigen 6 family member G5B	0.936
564318679	CCSER2	coiled-coil serine rich protein 2	0.939
186972129	TMPRSS9	transmembrane protease, serine 9	0.941
149033319	PRR15	proline rich 15	0.943
28972035	NUP214	nucleoporin 214	0.944
293353154	TBC1D1	TBC1 domain family member 1	0.949
58865986	RNASE12	ribonuclease A family member 12 (inactive)	0.954
157823859	METTL27	methyltransferase like 27	0.958
57012446	Krt42	keratin 42	0.959
23463263	NPW	neuropeptide W	0.962
564395190	LOC100909409 (includes others)	RGD1562660	0.962
9506775	HES2	hes family bHLH transcription factor 2	0.963
564312671	N/A	N/A	0.965
157821687	NEURL2	neuralized E3 ubiquitin protein ligase 2	0.966
209571573	ZNF707	zinc finger protein 707	0.966
68062078	N/A	N/A	0.968
148693657	DDX6	DEAD-box helicase 6	0.969
672043253	DENND4B	DENN domain containing 4B	0.969
755515866	BRAF	B-Raf proto-oncogene, serine/threonine kinase	0.990
149030324	CHRNA2	cholinergic receptor nicotinic alpha 2 subunit	0.994
1438906	NPY5R	neuropeptide Y receptor Y5	1.000
53734355	P2RY14	purinergic receptor P2Y14	1.000
149035338	APBB2	amyloid beta precursor protein binding family B member 2	1.000
149065466	ARHGEF5	Rho guanine nucleotide exchange factor 5	1.000
148235584	CLEC4A	C-type lectin domain family 4 member A	1.000
58865742	CYTIP	cytohesin 1 interacting protein	1.000
82654234	LILRA6	leukocyte immunoglobulin like receptor A6	1.000

157787179	POU2F3	POU class 2 homeobox 3	1.000
157786830	RILP	Rab interacting lysosomal protein	1.000
564395350	N/A	N/A	1.000
157817241	ISCA2	iron-sulfur cluster assembly 2	1.006
961454277	Dmd	dystrophin	1.007
149042010	Dapk2	death-associated protein kinase 2	1.010
672087472	REPS2	RALBP1 associated Eps domain containing 2	1.012
672026785	N/A	N/A	1.013
149059246	N/A	N/A	1.014
149041229	N/A	N/A	1.018
672087657	N/A	N/A	1.027
672083970	N/A	N/A	1.033
404501522	NXNL1	nucleoredoxin like 1	1.037
109512267	N/A	N/A	1.037
148674299	Gm14176	ubiquitin-conjugating enzyme E2I pseudogene	1.041
61097928	SNAI1	snail family transcriptional repressor 1	1.053
16758598	CYP27B1	cytochrome P450 family 27 subfamily B member 1	1.054
86129546	ZDHHC22	zinc finger DHHC-type containing 22	1.056
149067796	TMEM219	transmembrane protein 219	1.064
149018242	N/A	N/A	1.065
19424314	KCNE2	potassium voltage-gated channel subfamily E regulatory subunit 2	1.066
672080026	N/A	N/A	1.070
564296988	ZNF235	zinc finger protein 235	1.072
148665412	N/A	N/A	1.074
157818609	NT5C1A	5'-nucleotidase, cytosolic IA	1.075
16758572	DLK1	delta like non-canonical Notch ligand 1	1.086
157819397	CD22	CD22 molecule	1.100
281306708	VSX2	visual system homeobox 2	1.108
537260438	N/A	N/A	1.109
7242211	TRH	thyrotropin releasing hormone	1.113
8393654	KCNJ5	potassium voltage-gated channel subfamily J member 5	1.115
672082610	N/A	N/A	1.117
149066891	Lyc2	lysozyme C type 2	1.126
188536090	FAM241B	family with sequence similarity 241 member B	1.127
392339412	PLA2G4E	phospholipase A2 group IVE	1.128
12083683	SRD5A2	steroid 5 alpha-reductase 2	1.128
672054835	N/A	N/A	1.131

157786632	ZMYND15	zinc finger MYND-type containing 15	1.152
37693512	TLR9	toll like receptor 9	1.160
564372692	N/A	N/A	1.162
300796953	SYCE2	synaptonemal complex central element protein 2	1.163
149042270	LAS1L	LAS1 like, ribosome biogenesis factor	1.169
62078917	PAQR5	progestin and adipoQ receptor family member 5	1.181
6978525	FASLG	Fas ligand	1.186
564306247	PHACTR4	phosphatase and actin regulator 4	1.189
6978493	ALOX5	arachidonate 5-lipoxygenase	1.193
16758320	TNFSF4	TNF superfamily member 4	1.193
149047683	N/A	N/A	1.195
40786461	NAPEPLD	N-acyl phosphatidylethanolamine phospholipase D	1.199
6981080	IGFBP1	insulin like growth factor binding protein 1	1.209
564306696	SRBD1	S1 RNA binding domain 1	1.216
287323377	SLA2	Src like adaptor 2	1.216
669303362	N/A	N/A	1.221
11596857	KCNE3	potassium voltage-gated channel subfamily E regulatory subunit 3	1.222
157787081	WNT1	Wnt family member 1	1.222
564385630	ACOX2	acyl-CoA oxidase 2	1.222
149045964	PTH2R	parathyroid hormone 2 receptor	1.222
224967052	TLR5	toll like receptor 5	1.222
300793935	GSX1	GS homeobox 1	1.225
157822667	TAL2	TAL bHLH transcription factor 2	1.226
564309671	KIAA0895	KIAA0895	1.230
672056787	PRIMA1	proline rich membrane anchor 1	1.234
7106248	ANKRD1	ankyrin repeat domain 1	1.252
157786716	USH1G	USH1 protein network component sans	1.256
157818969	SEC14L4	SEC14 like lipid binding 4	1.263
157818187	Tfap2d	transcription factor AP-2 delta	1.263
672023787	N/A	N/A	1.264
157822485	HIST1H2BN	histone cluster 1 H2B family member n	1.265
476007854	BFSP2	beaded filament structural protein 2	1.267
672068318	PITPNM3	PITPNM family member 3	1.283
537158431	N/A	N/A	1.287
8393938	PADI3	peptidyl arginine deiminase 3	1.303
564317923	SACS	sacsin molecular chaperone	1.308
21070938	OTOS	otospiralin	1.317

564351511	RGD1565987	similar to F-box and leucine-rich repeat protein 18	1.319
13928802	WISP2	WNT1 inducible signaling pathway protein 2	1.322
149035901	N/A	N/A	1.322
149020413	Zfp599	zinc finger protein 599	1.322
568914642	N/A	N/A	1.333
392338392	PCNT	pericentrin	1.342
913513476	N/A	N/A	1.342
568972622	BPTF	bromodomain PHD finger transcription factor	1.343
56605846	DPEP3	dipeptidase 3	1.348
2231145	N/A	N/A	1.354
961766127	N/A	N/A	1.355
564300485	LOC102551095	uncharacterized LOC102551095	1.355
140969796	Cyp2c23	cytochrome P450, family 2, subfamily c, polypeptide 23	1.363
577019502	OVOL3	ovo like zinc finger 3	1.363
829923130	N/A	N/A	1.364
31745152	GPR151	G protein-coupled receptor 151	1.379
672084787	DPEP2	dipeptidase 2	1.392
121949750	PTPN20	protein tyrosine phosphatase, non-receptor type 20	1.392
126517485	TBC1D10C	TBC1 domain family member 10C	1.392
564301284	Ttf1	transcription termination factor, RNA polymerase I	1.402
569012000	KLF8	Kruppel like factor 8	1.406
672084625	LOC100909409 (includes others)	RGD1562660	1.407
564396366	PCNX2	pecanex homolog 2	1.408
6981312	OTC	ornithine carbamoyltransferase	1.415
19173800	Actn3	actinin alpha 3	1.415
57012346	HLA-DQA1	major histocompatibility complex, class II, DQ alpha 1	1.415
106879208	MYH4	myosin heavy chain 4	1.415
198442901	PKDREJ	polycystin family receptor for egg jelly	1.415
62078839	LRRC26	leucine rich repeat containing 26	1.421
11024678	Dbil5	diazepam binding inhibitor-like 5	1.423
270133003	H2-M5	histocompatibility 2, M region locus 5	1.429
13277927	RPLP0	ribosomal protein lateral stalk subunit P0	1.431
62079153	PLET1	placenta expressed transcript 1	1.433
564380493	SLC45A3	solute carrier family 45 member 3	1.441

157824012	TRIM45	tripartite motif containing 45	1.444
564329918	EMSY	EMSY, BRCA2 interacting transcriptional repressor	1.449
56606094	Aox2	aldehyde oxidase 2	1.459
350534944	STAB2	stabilin 2	1.459
803286756	N/A	N/A	1.475
84000579	FTL	ferritin light chain	1.478
149052674	NMUR2	neuromedin U receptor 2	1.485
157817264	ANKRD23	ankyrin repeat domain 23	1.505
625182464	N/A	N/A	1.507
155369646	AGBL4	ATP/GTP binding protein like 4	1.510
24899633	SLC12A8	solute carrier family 12 member 8	1.519
124487463	GPR161	G protein-coupled receptor 161	1.523
293347270	OSGIN2	oxidative stress induced growth inhibitor family member 2	1.541
74004170	HIST1H2AM	histone cluster 1 H2A family member m	1.549
54312124	HCST	hematopoietic cell signal transducer	1.554
149031942	N/A	N/A	1.558
564312944	KIAA0753	KIAA0753	1.568
672088045	N/A	N/A	1.568
568914628	GARNL3	GTPase activating Rap/RanGAP domain like 3	1.578
6981536	SI	sucrase-isomaltase	1.585
13928980	AQP3	aquaporin 3 (Gill blood group)	1.585
58865680	CES5A	carboxylesterase 5A	1.585
291490665	Gcg	glucagon	1.585
94400879	HRH2	histamine receptor H2	1.585
282154817	PLA2R1	phospholipase A2 receptor 1	1.585
149058209	SELE	selectin E	1.585
158533972	SPTA1	spectrin alpha, erythrocytic 1	1.585
11560026	STC2	stanniocalcin 2	1.585
56785424	Vom2r31	vomeronasal 2 receptor, 31	1.585
24308466	ITGB3	integrin subunit beta 3	1.596
575403049	ERBIN	erbb2 interacting protein	1.596
157818463	Gm38422	predicted gene, 38422	1.597
564321167	CHD9	chromodomain helicase DNA binding protein 9	1.600
564296772	N/A	N/A	1.601
672013187	DMWD	DM1 locus, WD repeat containing	1.605
594061361	N/A	N/A	1.609
23463269	NPB	neuropeptide B	1.609
564317999	N/A	N/A	1.633

58866012	TRIM55	tripartite motif containing 55	1.644
672019789	BTBD19	BTB domain containing 19	1.676
300795081	B4GALNT3	beta-1,4-N-acetyl-galactosaminyltransferase 3	1.678
157817368	SPDEF	SAM pointed domain containing ETS transcription factor	1.681
19424330	SLC25A21	solute carrier family 25 member 21	1.683
204595	Hist1h1e	histone cluster 1, H1e	1.688
408407614	DNA2	DNA replication helicase/nuclease 2	1.697
11464989	CD86	CD86 molecule	1.700
41054896	FUT7	fucosyltransferase 7	1.700
157817658	VIL1	villin 1	1.700
149020581	ZNF560	zinc finger protein 560	1.703
568990288	NIPBL	NIPBL, cohesin loading factor	1.705
672035395	DMWD	DM1 locus, WD repeat containing	1.706
564313512	N/A	N/A	1.707
157816947	GUCA1B	guanylate cyclase activator 1B	1.708
254553399	FBXO24	F-box protein 24	1.716
157819799	IQCH	IQ motif containing H	1.716
157786614	MFSD6L	major facilitator superfamily domain containing 6 like	1.716
576080555	GAPDH	glyceraldehyde-3-phosphate dehydrogenase	1.720
594100882	N/A	N/A	1.726
300795020	DEF6	DEF6, guanine nucleotide exchange factor	1.726
672088674	N/A	N/A	1.726
157820841	GP1BA	glycoprotein Ib platelet alpha subunit	1.726
569001477	MTCL1	microtubule crosslinking factor 1	1.726
149046722	IBSP	integrin binding sialoprotein	1.737
149032888	LOC100910237	uncharacterized LOC100910237	1.751
300794357	AOAH	acyloxyacyl hydrolase	1.755
157820135	CHRDL2	chordin like 2	1.762
13492975	NR5A2	nuclear receptor subfamily 5 group A member 2	1.778
157816967	Gm4925	predicted gene 4925	1.781
46237647	PSORS1C2	psoriasis susceptibility 1 candidate 2	1.807
140970928	DBH	dopamine beta-hydroxylase	1.807
402534543	GABRR1	gamma-aminobutyric acid type A receptor rho1 subunit	1.807
157821823	Ngp	neutrophilic granule protein	1.807
564318930	WDR17	WD repeat domain 17	1.807
157818127	CA7	carbonic anhydrase 7	1.824
148230802	Akr1c13	aldo-keto reductase family 1, member C13	1.826

207446700	Sec1	secretory blood group 1	1.826
157787012	SLAMF9	SLAM family member 9	1.841
392342449	PRSS56	protease, serine 56	1.848
8393090	CEBPE	CCAAT/enhancer binding protein epsilon	1.858
149042883	LOC100365365	rCG32328-like	1.861
16758254	CNGA1	cyclic nucleotide gated channel alpha 1	1.874
189181736	LAD1	ladinin 1	1.874
109488483	KIAA0753	KIAA0753	1.905
157821723	Gm30289	predicted gene, 30289	1.907
564297942	LOC103690302	AF4/FMR2 family member 2	1.909
149024390	PLA2G2D	phospholipase A2 group IID	1.914
955517176	N/A	N/A	1.918
162287188	LIF	LIF, interleukin 6 family cytokine	1.933
157820301	TMEM202	transmembrane protein 202	1.934
672019792	N/A	N/A	1.935
13027398	CD200R1	CD200 receptor 1	1.943
826279832	N/A	N/A	1.952
197381585	Urah	urate (5-hydroxyiso-) hydrolase	1.976
755495595	PRRC2C	proline rich coiled-coil 2C	1.981
149055781	N/A	N/A	1.994
148685413	ATP2A1	ATPase sarcoplasmic/endoplasmic reticulum Ca2+ transporting 1	2.000
157823427	KLHL31	kelch like family member 31	2.000
51243038	LY6G6D	lymphocyte antigen 6 family member G6D	2.000
160961485	MYLK3	myosin light chain kinase 3	2.000
564374250	NAGS	N-acetylglutamate synthase	2.000
568979594	SYT16	synaptotagmin 16	2.059
672070295	BAHCC1	BAH domain and coiled-coil containing 1	2.083
568995287	MKL2	MKL1/myocardin like 2	2.087
300794644	FREM3	FRAS1 related extracellular matrix 3	2.087
564329926	EMSY	EMSY, BRCA2 interacting transcriptional repressor	2.094
392354688	LVRN	laeverin	2.121
672067367	BC049762	cDNA sequence BC049762	2.126
148675846	FAM114A2	family with sequence similarity 114 member A2	2.128
157821741	LY86	lymphocyte antigen 86	2.128
564312627	ZFP62	ZFP62 zinc finger protein	2.139
28972866	CSMD3	CUB and Sushi multiple domains 3	2.140
568974167	SLC26A11	solute carrier family 26 member 11	2.149
564297852	CRTC3	CREB regulated transcription coactivator 3	2.151

564296586	Zfp943	zinc finger prtoein 943	2.170
109480102	SMARCC2	SWI/SNF related, matrix associated, actin dependent regulator of chromatin subfamily c member 2	2.178
149061720	Tnnt3	troponin T3, fast skeletal type	2.182
564329920	EMSY	EMSY, BRCA2 interacting transcriptional repressor	2.185
11024668	AIPL1	aryl hydrocarbon receptor interacting protein like 1	2.188
158508517	SDS	serine dehydratase	2.202
392343703	LOC100911027	protein MAL2-like	2.222
392342157	PHIP	pleckstrin homology domain interacting protein	2.226
568964954	EPB41L2	erythrocyte membrane protein band 4.1 like 2	2.228
392340509	PTPRD	protein tyrosine phosphatase, receptor type D	2.231
293352381	PAN3	PAN3 poly(A) specific ribonuclease subunit	2.239
56912237	KRT28	keratin 28	2.241
564323057	ARMCX4	armadillo repeat containing, X-linked 4	2.242
478536438	N/A	N/A	2.259
149042879	N/A	N/A	2.278
149053951	N/A	N/A	2.298
197385133	RGD1561157	RGD1561157	2.303
568941582	IQSEC1	IQ motif and Sec7 domain 1	2.308
56270329	ATP1A4	ATPase Na+/K+ transporting subunit alpha 4	2.322
300795696	CACNA2D4	calcium voltage-gated channel auxiliary subunit alpha2delta 4	2.322
71896592	IGFALS	insulin like growth factor binding protein acid labile subunit	2.322
8393941	PADI4	peptidyl arginine deiminase 4	2.322
564324736	L3MBTL3	L3MBTL3, histone methyl-lysine binding protein	2.353
164448680	HBB	hemoglobin subunit beta	2.371
392342139	TTC21A	tetratricopeptide repeat domain 21A	2.389
157818163	POF1B	POF1B, actin binding protein	2.392
672053814	N/A	N/A	2.392
149034139	C10orf128	chromosome 10 open reading frame 128	2.406
56119154	CPA6	carboxypeptidase A6	2.415
987404821	N/A	N/A	2.436
157819659	RRH	retinal pigment epithelium-derived rhodopsin homolog	2.447
672071273	GRAMD1C	GRAM domain containing 1C	2.450

293356488	RIC1	RIC1 homolog, RAB6A GEF complex partner 1	2.479
157820647	Scgb1c1	secretoglobin, family 1C, member 1	2.544
672013014	N/A	N/A	2.561
387182	HIST1H2AD	histone cluster 1 H2A family member d	2.562
13994175	FUT2	fucosyltransferase 2	2.585
157820271	LOXL4	lysyl oxidase like 4	2.585
13540693	MYOC	myocilin	2.585
11120690	NR1H4	nuclear receptor subfamily 1 group H member 4	2.585
57222314	OAS3	2'-5'-oligoadenylate synthetase 3	2.585
564329376	SRPK3	SRSF protein kinase 3	2.585
157819281	TRIM29	tripartite motif containing 29	2.585
8394529	VDR	vitamin D receptor	2.585
58866038	XKRX	XK related, X-linked	2.585
672038008	N/A	N/A	2.585
148696062	LRRC57	leucine rich repeat containing 57	2.603
672020363	N/A	N/A	2.620
148702471	N/A	N/A	2.698
157817726	ARL5C	ADP ribosylation factor like GTPase 5C	2.737
392334596	RSPH3	radial spoke head 3 homolog	2.755
9507065	SCN11A	sodium voltage-gated channel alpha subunit 11	2.755
521036477	N/A	N/A	2.766
672027860	APBB2	amyloid beta precursor protein binding family B member 2	2.804
25742760	AMH	anti-Mullerian hormone	2.807
157787002	Dpt	dermatopontin	2.807
1890097	IHH	indian hedgehog	2.907
197384923	C1orf87	chromosome 1 open reading frame 87	3.000
300796937	ESPNL	espin like	3.000
13591993	MMP9	matrix metallopeptidase 9	3.000
472235305	RPE65	RPE65, retinoid isomerohydrolase	3.000
564347547	LOC103690120	probable N-acetyltransferase CML1	3.030
283135196	THSD4	thrombospondin type 1 domain containing 4	3.044
149029159	N/A	N/A	3.080
564329920	EMSY	EMSY, BRCA2 interacting transcriptional repressor	3.112
672029704	ZCCHC6	zinc finger CCHC-type containing 6	3.117
149020413	Zfp599	zinc finger protein 599	3.158
260099641	MSH5	mutS homolog 5	3.170
8393891	P2RY2	purinergic receptor P2Y2	3.170

281332212	SH2D4B	SH2 domain containing 4B	3.170
564392795	MOCOS	molybdenum cofactor sulfurase	3.248
112984288	STEAP4	STEAP4 metalloreductase	3.248
672078236	Gucy1b2	guanylate cyclase 1, soluble, beta 2	3.322
61556961	THEG	theg spermatid protein	3.322
50370130	PALLD	palladin, cytoskeletal associated protein	3.361
28174920	RPL17	ribosomal protein L17	3.389
293340128	MIEF2	mitochondrial elongation factor 2	3.426
569009290	TENM1	teneurin transmembrane protein 1	3.450
16758550	BCL2L10	BCL2 like 10	3.459
25282405	BPIFA1	BPI fold containing family A member 1	3.459
208022681	Rhox2-ps	reproductive homeobox 2, pseudogene	3.459
299473749	C1orf226	chromosome 1 open reading frame 226	3.496
672088045	N/A	N/A	3.600
148670929	BATF	basic leucine zipper ATF-like transcription factor	3.700
148747510	BAAT	bile acid-CoA:amino acid N-acyltransferase	3.807
21245088	Ly6a (includes others)	lymphocyte antigen 6 complex, locus A	3.807
158187515	OAZ3	ornithine decarboxylase antizyme 3	3.807
62078779	ORAI3	ORAI calcium release-activated calcium modulator 3	3.807
850284744	N/A	N/A	3.858
672052120	RBM12B	RNA binding motif protein 12B	3.907
402747041	FAM217A	family with sequence similarity 217 member A	3.954
166157542	PABPN1L	poly(A) binding protein nuclear 1 like, cytoplasmic	3.954
755783452	N/A	N/A	4.087
392351087	HAGHL	hydroxyacylglutathione hydrolase like	4.120
23463315	Cyp2d1/Cyp2d5	cytochrome P450, family 2, subfamily d, polypeptide 1	4.170
564309734	IGSF9B	immunoglobulin superfamily member 9B	4.173
8393641	AADAT	aminoadipate aminotransferase	4.248
47577861	OR7D2	olfactory receptor family 7 subfamily D member 2	4.248
564352686	N/A	N/A	4.248
165970757	H2-T24	histocompatibility 2, T region locus 24	4.285
11024666	NTRK1	neurotrophic receptor tyrosine kinase 1	4.285
300795122	CLEC1B	C-type lectin domain family 1 member B	4.322

27545443	CEACAM4	carcinoembryonic antigen related cell adhesion molecule 4	4.392
298231202	PRG2	proteoglycan 2, pro eosinophil major basic protein	4.426
555986737	N/A	N/A	4.459
149038931	CNTRL	centriolin	4.492
341940965	MOS	MOS proto-oncogene, serine/threonine kinase	4.492
13928958	CRYBB3	crystallin beta B3	4.672
564308814	N/A	N/A	4.700
149034317	NUCKS1	nuclear casein kinase and cyclin dependent kinase substrate 1	4.833
392333013	CEP135	centrosomal protein 135	4.858
9506733	GJB5	gap junction protein beta 5	4.907
293349510	STAC	SH3 and cysteine rich domain	4.954
392338425	FAM184A	family with sequence similarity 184 member A	5.000
672020628	ATXN7L1	ataxin 7 like 1	5.157
59709455	EPOR	erythropoietin receptor	5.170
8394516	PLAUR	plasminogen activator, urokinase receptor	5.229
16758218	Hamp	hepcidin antimicrobial peptide	5.267
392342412	N/A	N/A	5.285
20301998	PROK2	prokineticin 2	5.358
70778849	Abcg3	ATP binding cassette subfamily G member 3	5.392
157818205	NOC3L	NOC3 like DNA replication regulator	5.833
351710149	N/A	N/A	5.966
148705984	Spink2	serine peptidase inhibitor, Kazal type 2	6.000
149054614	N/A	N/A	6.066
672017085	N/A	N/A	6.098
672052120	RBM12B	RNA binding motif protein 12B	6.366
149038394	N/A	N/A	6.539
564316929	FRYL	FRY like transcription coactivator	6.948
663243313	N/A	N/A	7.098
672019127	N/A	N/A	7.214
608785644	NPAS3	neuronal PAS domain protein 3	7.238
149026222	N/A	N/A	7.353
300797609	ELOVL7	ELOVL fatty acid elongase 7	7.451
293347435	PTPRD	protein tyrosine phosphatase, receptor type D	7.710
149057336	ZSCAN2	zinc finger and SCAN domain containing 2	7.758
62650795	DACT1	dishevelled binding antagonist of beta catenin 1	7.762
109472884	UBE3C	ubiquitin protein ligase E3C	8.197
927135720	N/A	N/A	8.311

672029702	ZCCHC6	zinc finger CCHC-type containing 6	8.441
**DEGs in male**			
**ID**	**Symbol**	**Entrez Gene Name**	**Expr Log Ratio**
399154114	KPNA2	karyopherin subunit alpha 2	-10.164
564314532	LRCH3	leucine rich repeats and calponin homology domain containing 3	-9.794
148675846	FAM114A2	family with sequence similarity 114 member A2	-9.570
672085486	EGLN1	egl-9 family hypoxia inducible factor 1	-9.276
440913556	N/A	N/A	-9.006
564378315	Zfp853	zinc finger protein 853	-8.895
672027854	APBB2	amyloid beta precursor protein binding family B member 2	-8.830
293349986	SMCHD1	structural maintenance of chromosomes flexible hinge domain containing 1	-8.728
8393418	GAPDH	glyceraldehyde-3-phosphate dehydrogenase	-8.658
672057084	N/A	N/A	-8.611
392351087	HAGHL	hydroxyacylglutathione hydrolase like	-8.539
50510463	PRUNE2	prune homolog 2	-8.443
392337738	LYSMD4	LysM domain containing 4	-8.426
189011657	Uqcrb	ubiquinol-cytochrome c reductase binding protein	-8.409
672035064	N/A	N/A	-8.388
672088045	N/A	N/A	-8.384
564324736	L3MBTL3	L3MBTL3, histone methyl-lysine binding protein	-8.349
157820217	Gsta4	glutathione S-transferase, alpha 4	-8.317
564380050	2410141K09Rik	RIKEN cDNA 2410141K09 gene	-8.234
672035060	CIC	capicua transcriptional repressor	-8.114
197304784	IQSEC1	IQ motif and Sec7 domain 1	-8.109
672032217	REPS2	RALBP1 associated Eps domain containing 2	-8.071
672088045	N/A	N/A	-8.055
154090947	NPAS3	neuronal PAS domain protein 3	-8.028
672040275	CPEB3	cytoplasmic polyadenylation element binding protein 3	-8.022
66730276	C7orf49	chromosome 7 open reading frame 49	-7.989
672059354	N/A	N/A	-7.983
564378170	PAN3	PAN3 poly(A) specific ribonuclease subunit	-7.966

109484871	HERC1	HECT and RLD domain containing E3 ubiquitin protein ligase family member 1	-7.895
564377502	EIF4G1	eukaryotic translation initiation factor 4 gamma 1	-7.870
392331668	HAGHL	hydroxyacylglutathione hydrolase like	-7.864
755566690	HUWE1	HECT, UBA and WWE domain containing 1, E3 ubiquitin protein ligase	-7.864
149051960	FLYWCH2	FLYWCH family member 2	-7.781
6690510	CHD9	chromodomain helicase DNA binding protein 9	-7.762
564311678	PLEKHM3	pleckstrin homology domain containing M3	-7.748
293347435	PTPRD	protein tyrosine phosphatase, receptor type D	-7.707
564310551	N/A	N/A	-7.679
34872960	SMG8	SMG8, nonsense mediated mRNA decay factor	-7.600
149057336	ZSCAN2	zinc finger and SCAN domain containing 2	-7.600
149029159	N/A	N/A	-7.585
293341811	PRR14L	proline rich 14 like	-7.570
564313676	FBF1	Fas binding factor 1	-7.508
148704240	ZMYM2	zinc finger MYM-type containing 2	-7.459
149047880	SCAI	suppressor of cancer cell invasion	-7.426
66911867	PHF20L1	PHD finger protein 20 like 1	-7.418
149020413	Zfp599	zinc finger protein 599	-7.375
564296586	Zfp943	zinc finger prtoein 943	-7.375
226698394	UNC80	unc-80 homolog, NALCN channel complex subunit	-7.349
564316241	CEP170	centrosomal protein 170	-7.322
293347435	PTPRD	protein tyrosine phosphatase, receptor type D	-7.304
293344558	PCNX3	pecanex homolog 3	-7.285
148675846	FAM114A2	family with sequence similarity 114 member A2	-7.276
564322493	RPGR	retinitis pigmentosa GTPase regulator	-7.248
568979794	NPAS3	neuronal PAS domain protein 3	-7.219
564315185	CUX1	cut like homeobox 1	-7.209
564375502	Mxra7	matrix-remodelling associated 7	-7.209
672039093	N/A	N/A	-7.209
672026702	N/A	N/A	-7.180
672089723	LOC103690073	protein FAM76A	-7.160
564312627	ZFP62	ZFP62 zinc finger protein	-7.160
564305043	RBM12B	RNA binding motif protein 12B	-7.150
672089122	N/A	N/A	-7.150
564302768	KIAA1755	KIAA1755	-7.077
564320728	Fbxo38	F-box protein 38	-7.066

564312515	SH3PXD2B	SH3 and PX domains 2B	-7.066
625198911	N/A	N/A	-7.066
913507039	N/A	N/A	-7.055
672020363	N/A	N/A	-7.033
148694630	N/A	N/A	-6.919
149035005	IQCE	IQ motif containing E	-6.907
755783452	N/A	N/A	-6.907
392339806	CFAP69	cilia and flagella associated protein 69	-6.895
672019578	MYSM1	Myb like, SWIRM and MPN domains 1	-6.895
564303135	KMT2C	lysine methyltransferase 2C	-6.870
147907212	ASAP2	ArfGAP with SH3 domain, ankyrin repeat and PH domain 2	-6.867
672029115	N/A	N/A	-6.845
672082610	N/A	N/A	-6.781
564314663	VPS8	VPS8, CORVET complex subunit	-6.755
564307783	TECPR2	tectonin beta-propeller repeat containing 2	-6.741
62644863	NUP188	nucleoporin 188	-6.700
672031484	PCNX2	pecanex homolog 2	-6.687
109460021	KIAA2026	KIAA2026	-6.658
564296517	N/A	N/A	-6.615
568992323	CSMD3	CUB and Sushi multiple domains 3	-6.508
672087657	N/A	N/A	-6.492
564314389	DZIP3	DAZ interacting zinc finger protein 3	-6.443
672024381	N/A	N/A	-6.443
564320724	Fbxo38	F-box protein 38	-6.426
392338862	ATP8B2	ATPase phospholipid transporting 8B2	-6.358
149031942	N/A	N/A	-6.304
672060766	N/A	N/A	-6.304
672017191	N/A	N/A	-6.267
564318843	PSD3	pleckstrin and Sec7 domain containing 3	-6.248
109470195	TNKS1BP1	tankyrase 1 binding protein 1	-6.229
564302561	KIZ	kizuna centrosomal protein	-6.209
109490297	ABCA3	ATP binding cassette subfamily A member 3	-6.190
672084304	N/A	N/A	-6.170
672089090	LOC103694537	mediator of RNA polymerase II transcription subunit 14-like	-6.109
564310188	IGDCC4	immunoglobulin superfamily DCC subclass member 4	-6.087
672087664	N/A	N/A	-6.087
148684403	N/A	N/A	-6.087

564297387	Zfp658	zinc finger protein 658	-6.022
149042879	N/A	N/A	-6.022
672041794	RGD1310081	similar to hypothetical protein FLJ13231	-6.000
568970987	MBTD1	mbt domain containing 1	-5.977
672088045	N/A	N/A	-5.931
537216146	N/A	N/A	-5.931
149065087	CADPS2	calcium dependent secretion activator 2	-5.883
13242279	GJA3	gap junction protein alpha 3	-5.883
164698411	CTDSP2	CTD small phosphatase 2	-5.858
564307079	ATXN7L1	ataxin 7 like 1	-5.833
672079010	N/A	N/A	-5.833
672068318	PITPNM3	PITPNM family member 3	-5.700
569009290	TENM1	teneurin transmembrane protein 1	-5.700
564307173	HEATR5A	HEAT repeat containing 5A	-5.672
109458652	VSIG10L	V-set and immunoglobulin domain containing 10 like	-5.672
672063876	MGC116197 (includes others)	similar to RIKEN cDNA 1700001E04	-5.644
672031167	C19orf57	chromosome 19 open reading frame 57	-5.615
564329920	EMSY	EMSY, BRCA2 interacting transcriptional repressor	-5.585
568941572	IQSEC1	IQ motif and Sec7 domain 1	-5.585
392340959	ITSN2	intersectin 2	-5.585
294568	LOC108348108	heat shock 70 kDa protein 1A	-5.585
568979792	NPAS3	neuronal PAS domain protein 3	-5.585
672069572	KANSL1	KAT8 regulatory NSL complex subunit 1	-5.555
31077126	ROBO4	roundabout guidance receptor 4	-5.524
281306763	NTN3	netrin 3	-5.426
293346251	TMEM62	transmembrane protein 62	-5.322
392334509	N/A	N/A	-5.285
148668227	GPC6	glypican 6	-5.248
564323075	ZMAT1	zinc finger matrin-type 1	-5.129
672053077	N/A	N/A	-5.033
672066092	UNC80	unc-80 homolog, NALCN channel complex subunit	-4.954
672029704	ZCCHC6	zinc finger CCHC-type containing 6	-4.937
149052470	ZNF454	zinc finger protein 454	-4.807
564329926	EMSY	EMSY, BRCA2 interacting transcriptional repressor	-4.655
74184716	Kat6b	K(lysine) acetyltransferase 6B	-4.644

564342627	TP53BP1	tumor protein p53 binding protein 1	-4.594
114053317	Aph1c	aph1 homolog C, gamma secretase subunit	-4.459
568977658	ASAP2	ArfGAP with SH3 domain, ankyrin repeat and PH domain 2	-4.392
392334411	ANKRD11	ankyrin repeat domain 11	-4.358
564342632	TP53BP1	tumor protein p53 binding protein 1	-4.322
564305557	PTPRD	protein tyrosine phosphatase, receptor type D	-4.261
564305562	PTPRD	protein tyrosine phosphatase, receptor type D	-4.170
149020413	Zfp599	zinc finger protein 599	-4.000
392340509	PTPRD	protein tyrosine phosphatase, receptor type D	-3.792
28972714	KLHL13	kelch like family member 13	-3.463
564317997	N/A	N/A	-3.459
672026861	N/A	N/A	-3.459
564357330	N/A	N/A	-3.358
564321260	N/A	N/A	-3.298
672022282	KIF21A	kinesin family member 21A	-3.257
109492822	PAXBP1	PAX3 and PAX7 binding protein 1	-3.208
913486723	N/A	N/A	-3.178
109498003	ODR4	odr-4 GPCR localization factor homolog	-3.170
54019438	PCDHAC1	protocadherin alpha subfamily C, 1	-3.170
655644820	N/A	N/A	-3.170
564397808	HNRNPH3	heterogeneous nuclear ribonucleoprotein H3	-3.035
564298047	GDPD5	glycerophosphodiester phosphodiesterase domain containing 5	-3.017
20806113	BAMBI	BMP and activin membrane bound inhibitor	-2.929
564323078	N/A	N/A	-2.916
392331954	KANSL1	KAT8 regulatory NSL complex subunit 1	-2.915
747019224	SRCAP	Snf2 related CREBBP activator protein	-2.874
947324855	N/A	N/A	-2.791
124486586	AUTS2	AUTS2, activator of transcription and developmental regulator	-2.742
157819701	Ctla2a	cytotoxic T lymphocyte-associated protein 2 alpha	-2.682
157818607	C4orf46	chromosome 4 open reading frame 46	-2.585
148682823	N/A	N/A	-2.547
61556945	MOAP1	modulator of apoptosis 1	-2.508
564303143	KMT2C	lysine methyltransferase 2C	-2.497
149037646	N/A	N/A	-2.478
672022270	KIF21A	kinesin family member 21A	-2.422
672022227	RGS22	regulator of G protein signaling 22	-2.411

564339225	N/A	N/A	-2.410
14277700	RPS12	ribosomal protein S12	-2.381
564305934	BTBD19	BTB domain containing 19	-2.363
817273427	N/A	N/A	-2.336
293351303	METTL22	methyltransferase like 22	-2.334
67078412	LRRC63	leucine rich repeat containing 63	-2.322
755566692	HUWE1	HECT, UBA and WWE domain containing 1, E3 ubiquitin protein ligase	-2.222
564321167	CHD9	chromodomain helicase DNA binding protein 9	-2.203
392331829	ATAD5	ATPase family, AAA domain containing 5	-2.188
149408137	DHX58	DExH-box helicase 58	-2.138
564311717	KANSL1L	KAT8 regulatory NSL complex subunit 1 like	-2.095
158711755	C17orf97	chromosome 17 open reading frame 97	-2.092
672074711	N/A	N/A	-2.071
56090600	DNAI2	dynein axonemal intermediate chain 2	-2.066
293340825	USF3	upstream transcription factor family member 3	-2.065
148702471	N/A	N/A	-2.048
209870097	NLRP6	NLR family pyrin domain containing 6	-1.977
157820267	MEI4	meiotic double-stranded break formation protein 4	-1.975
884945546	N/A	N/A	-1.956
124487463	GPR161	G protein-coupled receptor 161	-1.954
918577634	N/A	N/A	-1.944
672079397	CCDC66	coiled-coil domain containing 66	-1.932
149038394	N/A	N/A	-1.902
568959295	Msantd2	Myb/SANT-like DNA-binding domain containing 2	-1.891
568973498	TNRC6C	trinucleotide repeat containing 6C	-1.886
293356488	RIC1	RIC1 homolog, RAB6A GEF complex partner 1	-1.826
68063179	N/A	N/A	-1.817
293349343	MYO6	myosin VI	-1.813
672038314	N/A	N/A	-1.800
57526854	IFI35	interferon induced protein 35	-1.798
296486245	N/A	N/A	-1.783
148679862	SLC35F3	solute carrier family 35 member F3	-1.767
817328905	N/A	N/A	-1.766
564331258	ZNF688	zinc finger protein 688	-1.762
672029702	ZCCHC6	zinc finger CCHC-type containing 6	-1.759
672073127	WDR66	WD repeat domain 66	-1.737

62079275	Ptges3l1	prostaglandin E synthase 3-like 1	-1.729
672025117	MBTD1	mbt domain containing 1	-1.724
752993027	HSPB1	heat shock protein family B (small) member 1	-1.704
672052399	N/A	N/A	-1.703
119226204	CFAP206	cilia and flagella associated protein 206	-1.697
564379810	TMEM119	transmembrane protein 119	-1.693
8394221	Rps3a1	ribosomal protein S3A1	-1.691
564315358	N/A	N/A	-1.686
594061361	N/A	N/A	-1.675
8393742	MAG	myelin associated glycoprotein	-1.661
942065067	N/A	N/A	-1.658
74271851	CLDN23	claudin 23	-1.637
149025029	SUSD6	sushi domain containing 6	-1.637
392339263	PKP4	plakophilin 4	-1.635
124378035	TNRC6C	trinucleotide repeat containing 6C	-1.634
57526957	ACY3	aminoacylase 3	-1.624
672040664	CFAP43	cilia and flagella associated protein 43	-1.622
625210478	N/A	N/A	-1.619
149025439	DICER1	dicer 1, ribonuclease III	-1.603
293347270	OSGIN2	oxidative stress induced growth inhibitor family member 2	-1.603
38454288	P4HA3	prolyl 4-hydroxylase subunit alpha 3	-1.585
149052692	N/A	N/A	-1.576
564322532	FTSJ1	FtsJ RNA methyltransferase homolog 1	-1.564
559098430	ARMC3	armadillo repeat containing 3	-1.556
564312627	ZFP62	ZFP62 zinc finger protein	-1.556
281332082	THBS2	thrombospondin 2	-1.536
149064672	ARHGEF26	Rho guanine nucleotide exchange factor 26	-1.525
676272056	N/A	N/A	-1.497
672028646	N/A	N/A	-1.490
564341299	N/A	N/A	-1.458
9910536	RNASE4	ribonuclease A family member 4	-1.456
20301982	UNC13D	unc-13 homolog D	-1.449
31542125	ALOX15	arachidonate 15-lipoxygenase	-1.437
149056134	Zfp17	zinc finger protein 585B	-1.433
564310412	DOPEY1	dopey family member 1	-1.429
392339263	PKP4	plakophilin 4	-1.429
187957728	FANCM	Fanconi anemia complementation group M	-1.425
672086880	N/A	N/A	-1.424

755566692	HUWE1	HECT, UBA and WWE domain containing 1, E3 ubiquitin protein ligase	-1.410
672028287	Kat6b	K(lysine) acetyltransferase 6B	-1.409
564297736	N/A	N/A	-1.401
672053062	FKBP15	FK506 binding protein 15	-1.398
672063750	N/A	N/A	-1.394
480306394	Mcpt4	mast cell protease 4	-1.391
564323057	ARMCX4	armadillo repeat containing, X-linked 4	-1.388
564315207	TMEM270	transmembrane protein 270	-1.386
674082951	N/A	N/A	-1.383
568927637	ADAMTSL1	ADAMTS like 1	-1.379
227116255	P2RX6	purinergic receptor P2X 6	-1.379
149043399	TAPBP	TAP binding protein	-1.370
564393142	WDR36	WD repeat domain 36	-1.364
149056609	DEDD2	death effector domain containing 2	-1.351
470605072	N/A	N/A	-1.350
564300505	SH3D19	SH3 domain containing 19	-1.349
564388219	ARHGAP22	Rho GTPase activating protein 22	-1.344
40254742	NCF1	neutrophil cytosolic factor 1	-1.338
392334596	RSPH3	radial spoke head 3 homolog	-1.337
6981642	TCP1	t-complex 1	-1.328
672061705	KMT2A	lysine methyltransferase 2A	-1.326
672023936	N/A	N/A	-1.325
293358436	FOXP2	forkhead box P2	-1.322
149043385	N/A	N/A	-1.322
392340768	DISP3	dispatched RND transporter family member 3	-1.320
77404174	HLA-A	major histocompatibility complex, class I, A	-1.314
12621098	EPHX2	epoxide hydrolase 2	-1.311
62656582	KIAA0100	KIAA0100	-1.300
564400341	N/A	N/A	-1.293
672071353	N/A	N/A	-1.284
564329920	EMSY	EMSY, BRCA2 interacting transcriptional repressor	-1.275
149050972	N/A	N/A	-1.272
672063675	N/A	N/A	-1.266
157787062	GRID2IP	Grid2 interacting protein	-1.260
564311918	C2orf72	chromosome 2 open reading frame 72	-1.253
672088045	N/A	N/A	-1.253
149020413	Zfp599	zinc finger protein 599	-1.250
149044121	N/A	N/A	-1.243

197333844	LLPH	LLP homolog, long-term synaptic facilitation	-1.241
564312009	COL6A3	collagen type VI alpha 3 chain	-1.239
18266694	PDE11A	phosphodiesterase 11A	-1.239
564311681	PIKFYVE	phosphoinositide kinase, FYVE-type zinc finger containing	-1.237
281306821	HEY2	hes related family bHLH transcription factor with YRPW motif 2	-1.232
293346251	TMEM62	transmembrane protein 62	-1.225
672046728	N/A	N/A	-1.224
61889085	GADD45A	growth arrest and DNA damage inducible alpha	-1.219
568964944	EPB41L2	erythrocyte membrane protein band 4.1 like 2	-1.215
293344867	N/A	N/A	-1.209
410515422	NTN1	netrin 1	-1.204
109501553	METTL17	methyltransferase like 17	-1.202
149041058	RCOR3	REST corepressor 3	-1.198
149018406	ALS2CL	ALS2 C-terminal like	-1.194
293349725	AMER3	APC membrane recruitment protein 3	-1.190
166235131	MTCL1	microtubule crosslinking factor 1	-1.187
564314685	EIF4G1	eukaryotic translation initiation factor 4 gamma 1	-1.186
66730305	ERICH2	glutamate rich 2	-1.181
293341054	IQCE	IQ motif containing E	-1.180
672016875	LOC103690320	FERM and PDZ domain-containing protein 3	-1.179
149048115	KHDC4	KH domain containing 4, pre-mRNA splicing factor	-1.168
564345430	LOC100910079	actin-related protein 3B-like	-1.150
157822457	SYNC	syncoilin, intermediate filament protein	-1.146
157821431	GAL3ST1	galactose-3-O-sulfotransferase 1	-1.141
157818989	LRRC71	leucine rich repeat containing 71	-1.141
672042306	N/A	N/A	-1.134
32996721	NDUFA10	NADH:ubiquinone oxidoreductase subunit A10	-1.133
672076690	N/A	N/A	-1.133
392334475	RGD1560020_predicted	similar to Myb proto-oncogene protein (C-myb) (predicted)	-1.127
672012705	SYNE1	spectrin repeat containing nuclear envelope protein 1	-1.120
291463305	SHISA9	shisa family member 9	-1.118
564373460	SLFN13	schlafen family member 13	-1.109
672047353	RALGAPA2	Ral GTPase activating protein catalytic alpha subunit 2	-1.107

164448680	HBB	hemoglobin subunit beta	-1.101
564305252	FBXO10	F-box protein 10	-1.097
71361637	STRA6	stimulated by retinoic acid 6	-1.096
564310624	CDHR4	cadherin related family member 4	-1.089
66730382	TRNT1	tRNA nucleotidyl transferase 1	-1.089
2231145	N/A	N/A	-1.075
392332346	RUBCN	RUN and cysteine rich domain containing beclin 1 interacting protein	-1.067
8393123	CHRM5	cholinergic receptor muscarinic 5	-1.066
157823801	SLC50A1	solute carrier family 50 member 1	-1.064
672076581	N/A	N/A	-1.062
589937133	N/A	N/A	-1.060
564355419	Nbas	neuroblastoma amplified sequence	-1.053
293344867	N/A	N/A	-1.050
674048263	N/A	N/A	-1.037
392339456	CKAP2L	cytoskeleton associated protein 2 like	-1.026
157820739	GPR68	G protein-coupled receptor 68	-1.024
300797715	NDST4	N-deacetylase and N-sulfotransferase 4	-1.016
564316927	FRYL	FRY like transcription coactivator	-1.010
657940868	PALLD	palladin, cytoskeletal associated protein	-1.009
564297387	Zfp658	zinc finger protein 658	-1.000
149047375	CCDC96	coiled-coil domain containing 96	-0.993
564301284	Ttf1	transcription termination factor, RNA polymerase I	-0.993
470602254	N/A	N/A	-0.990
392331598	MPV17L	MPV17 mitochondrial inner membrane protein like	-0.987
392331598	MPV17L	MPV17 mitochondrial inner membrane protein like	-0.987
149050030	MTRF1	mitochondrial translation release factor 1	-0.977
672089449	N/A	N/A	-0.973
818015	HBB	hemoglobin subunit beta	-0.970
194440693	Maml2	mastermind like transcriptional coactivator 2	-0.967
672028919	N/A	N/A	-0.964
685536628	N/A	N/A	-0.961
672018099	N/A	N/A	-0.953
38322763	WNT2	Wnt family member 2	-0.943
569009290	TENM1	teneurin transmembrane protein 1	-0.942
672088045	N/A	N/A	-0.942
17105368	KLF9	Kruppel like factor 9	-0.940

149052383	TRIM7	tripartite motif containing 7	-0.939
293349179	CBL	Cbl proto-oncogene	-0.938
209870105	GPR37L1	G protein-coupled receptor 37 like 1	-0.938
56676356	SLC10A4	solute carrier family 10 member 4	-0.938
564296997	ZNF45	zinc finger protein 45	-0.931
672028986	N/A	N/A	-0.931
149066285	PHF20L1	PHD finger protein 20 like 1	-0.930
61097937	VEGFB	vascular endothelial growth factor B	-0.926
281332166	GPR158	G protein-coupled receptor 158	-0.922
564313508	BPTF	bromodomain PHD finger transcription factor	-0.921
564348231	RPUSD3	RNA pseudouridylate synthase domain containing 3	-0.918
293340128	MIEF2	mitochondrial elongation factor 2	-0.914
148687007	CEP128	centrosomal protein 128	-0.912
564301352	PRRC2B	proline rich coiled-coil 2B	-0.910
293346666	RBMXL1	RNA binding motif protein, X-linked like 1	-0.907
197333844	LLPH	LLP homolog, long-term synaptic facilitation	-0.904
187282382	RGD1563441	similar to RIKEN cDNA A030009H04	-0.904
564342470	MGA	MGA, MAX dimerization protein	-0.903
56119147	ARRDC3	arrestin domain containing 3	-0.898
564318923	WDR17	WD repeat domain 17	-0.898
149054281	CNTNAP1	contactin associated protein 1	-0.888
307548437	NYAP2	neuronal tyrosine-phosphorylated phosphoinositide-3-kinase adaptor 2	-0.887
149046828	N/A	N/A	-0.885
149051391	N/A	N/A	-0.883
209862917	FCGR2A	Fc fragment of IgG receptor IIa	-0.882
148702528	OTOP3	otopetrin 3	-0.875
564352536	Szt2	SZT2, KICSTOR complex subunit	-0.868
61889110	OSTF1	osteoclast stimulating factor 1	-0.865
564329916	EMSY	EMSY, BRCA2 interacting transcriptional repressor	-0.864
564324344	LOC363306 (includes others)	hypothetical protein LOC363306	-0.864
72255523	DNALI1	dynein axonemal light intermediate chain 1	-0.862
884945546	N/A	N/A	-0.862
672052120	RBM12B	RNA binding motif protein 12B	-0.861
83320101	AFG1L	AFG1 like ATPase	-0.857
30027645	GHR	growth hormone receptor	-0.857
564342055	Apip	APAF1 interacting protein	-0.853

564396366	PCNX2	pecanex homolog 2	-0.852
148747412	CBWD1	COBW domain containing 1	-0.850
148704596	L3HYPDH	trans-L-3-hydroxyproline dehydratase	-0.846
157817364	Fbxl22	F-box and leucine-rich repeat protein 22	-0.842
564334233	CFAP43	cilia and flagella associated protein 43	-0.841
19924079	DNASE2	deoxyribonuclease 2, lysosomal	-0.835
672069802	C1QTNF1	C1q and TNF related 1	-0.833
672016642	GAPVD1	GTPase activating protein and VPS9 domains 1	-0.832
309243084	FUT4	fucosyltransferase 4	-0.831
564311685	PIKFYVE	phosphoinositide kinase, FYVE-type zinc finger containing	-0.826
149018731	TMEM108	transmembrane protein 108	-0.826
149031998	ACVRL1	activin A receptor like type 1	-0.823
293341722	N/A	N/A	-0.823
672087474	REPS2	RALBP1 associated Eps domain containing 2	-0.818
672087474	REPS2	RALBP1 associated Eps domain containing 2	-0.818
149042171	RTL5	retrotransposon Gag like 5	-0.818
564336411	POSTN	periostin	-0.811
531999911	N/A	N/A	-0.806
725571770	N/A	N/A	-0.803
73487332	C1orf115	chromosome 1 open reading frame 115	-0.799
46485412	ABCA7	ATP binding cassette subfamily A member 7	-0.798
564309742	IGSF9B	immunoglobulin superfamily member 9B	-0.796
564317722	N/A	N/A	-0.792
564313508	BPTF	bromodomain PHD finger transcription factor	-0.791
672078695	N/A	N/A	-0.786
354475081	N/A	N/A	-0.786
564313510	BPTF	bromodomain PHD finger transcription factor	-0.785
157820751	FAM241A	family with sequence similarity 241 member A	-0.771
987938912	N/A	N/A	-0.768
157823151	DLEU7	deleted in lymphocytic leukemia, 7	-0.761
568961599	VPS13C	vacuolar protein sorting 13 homolog C	-0.758
157823283	Coch	cochlin	-0.757
672012431	SASH1	SAM and SH3 domain containing 1	-0.756
672053541	N/A	N/A	-0.756
157820141	KLHDC1	kelch domain containing 1	-0.755
880855761	N/A	N/A	-0.755
293351987	UBE2O	ubiquitin conjugating enzyme E2 O	-0.752
13540697	NOV	nephroblastoma overexpressed	-0.751
392340959	ITSN2	intersectin 2	-0.750

157819949	ITGA4	integrin subunit alpha 4	-0.748
672026534	N/A	N/A	-0.746
293342292	FAM208A	family with sequence similarity 208 member A	-0.742
392351290	DNAH9	dynein axonemal heavy chain 9	-0.741
564378166	PAN3	PAN3 poly(A) specific ribonuclease subunit	-0.740
675706185	N/A	N/A	-0.739
157824208	NTNG1	netrin G1	-0.737
672022659	N/A	N/A	-0.737
281182643	ALK	ALK receptor tyrosine kinase	-0.734
13592079	S100A10	S100 calcium binding protein A10	-0.733
672066075	COL6A3	collagen type VI alpha 3 chain	-0.732
149057816	N/A	N/A	-0.730
513022818	N/A	N/A	-0.724
672023724	PRICKLE4	prickle planar cell polarity protein 4	-0.721
157786678	Cisd3	CDGSH iron sulfur domain 3	-0.719
212549544	C15orf39	chromosome 15 open reading frame 39	-0.716
564366772	MGC116197 (includes others)	similar to RIKEN cDNA 1700001E04	-0.715
26024221	PRSS12	protease, serine 12	-0.708
564340633	OLFML2A	olfactomedin like 2A	-0.707
148664561	DTNA	dystrobrevin alpha	-0.706
672044124	N/A	N/A	-0.706
9506475	CDK1	cyclin dependent kinase 1	-0.702
564309732	IGSF9B	immunoglobulin superfamily member 9B	-0.702
803119291	N/A	N/A	-0.700
149053909	COL1A1	collagen type I alpha 1 chain	-0.697
672058561	N/A	N/A	-0.697
537241732	N/A	N/A	-0.694
149034092	Grid1	glutamate ionotropic receptor delta type subunit 1	-0.693
392341692	FMNL3	formin like 3	-0.689
672017085	N/A	N/A	-0.684
157822891	ADCK2	aarF domain containing kinase 2	-0.683
149062990	CUX1	cut like homeobox 1	-0.682
197927137	WDR63	WD repeat domain 63	-0.679
154937382	MYL9	myosin light chain 9	-0.678
68534736	ERAP1	endoplasmic reticulum aminopeptidase 1	-0.676
157786914	OGFOD2	2-oxoglutarate and iron dependent oxygenase domain containing 2	-0.675
672014740	MAMDC2	MAM domain containing 2	-0.671

725595815	N/A	N/A	-0.671
155369293	AEBP1	AE binding protein 1	-0.668
672057459	DGKA	diacylglycerol kinase alpha	-0.664
19924029	ADRA2C	adrenoceptor alpha 2C	-0.663
148697042	N/A	N/A	-0.658
564323143	Gprasp2	G protein-coupled receptor associated sorting protein 2	-0.655
12083595	GRM4	glutamate metabotropic receptor 4	-0.653
564300485	LOC102551095	uncharacterized LOC102551095	-0.652
154090947	NPAS3	neuronal PAS domain protein 3	-0.652
537234259	N/A	N/A	-0.651
188536090	FAM241B	family with sequence similarity 241 member B	-0.644
149039154	COQ4	coenzyme Q4	-0.642
74200445	PNPLA6	patatin like phospholipase domain containing 6	-0.642
157818491	DUS2	dihydrouridine synthase 2	-0.630
219275548	DUSP19	dual specificity phosphatase 19	-0.630
672070295	BAHCC1	BAH domain and coiled-coil containing 1	-0.629
149053793	TSPOAP1	TSPO associated protein 1	-0.627
672027054	N/A	N/A	-0.627
67846096	MFSD3	major facilitator superfamily domain containing 3	-0.626
198041781	GLTPD2	glycolipid transfer protein domain containing 2	-0.625
157819765	OGDHL	oxoglutarate dehydrogenase like	-0.625
157820695	MRPL57	mitochondrial ribosomal protein L57	-0.622
564317714	Ktn1	kinectin 1	-0.621
568950242	Pgap2	post-GPI attachment to proteins 2	-0.618
300798165	ZBTB40	zinc finger and BTB domain containing 40	-0.618
16758130	WNT4	Wnt family member 4	-0.615
402478640	HTRA3	HtrA serine peptidase 3	-0.614
312836782	MRPS27	mitochondrial ribosomal protein S27	-0.614
57527919	GPD1	glycerol-3-phosphate dehydrogenase 1	-0.612
568983685	TMEM161B	transmembrane protein 161B	-0.612
11560087	PYGL	glycogen phosphorylase L	-0.610
148747464	SCD	stearoyl-CoA desaturase	-0.610
564315812	NAV1	neuron navigator 1	-0.607
564379463	N/A	N/A	-0.606
157823299	CSGALNACT1	chondroitin sulfate N-acetylgalactosaminyltransferase 1	-0.605
672029180	CCSER2	coiled-coil serine rich protein 2	-0.603
61556857	TM7SF2	transmembrane 7 superfamily member 2	-0.602

672015368	MAST4	microtubule associated serine/threonine kinase family member 4	-0.600
237757336	OLIG1	oligodendrocyte transcription factor 1	-0.599
564299864	N/A	N/A	-0.599
672043209	RUSC1	RUN and SH3 domain containing 1	-0.597
157817033	TJAP1	tight junction associated protein 1	-0.597
47059110	DXO	decapping exoribonuclease	-0.596
672066409	N/A	N/A	-0.596
62079069	RSRC1	arginine and serine rich coiled-coil 1	-0.595
157819033	RNASET2	ribonuclease T2	-0.594
672026129	LRCH3	leucine rich repeats and calponin homology domain containing 3	-0.593
672057384	N/A	N/A	-0.593
402745263	COL11A1	collagen type XI alpha 1 chain	-0.592
157951643	ACTN2	actinin alpha 2	-0.590
110347493	PCDHA9	protocadherin alpha 9	-0.587
157822759	PARP2	poly(ADP-ribose) polymerase 2	-0.585
149034959	ZNF12	zinc finger protein 12	-0.585
14269568	LXN	latexin	-0.584
157822577	MAN1C1	mannosidase alpha class 1C member 1	-0.584
564314671	VPS8	VPS8, CORVET complex subunit	-0.584
565655	ZIC1	Zic family member 1	-0.583
392343448	KLHL13	kelch like family member 13	-0.582
1478205	PPP1R14B	protein phosphatase 1 regulatory inhibitor subunit 14B	-0.582
586551369	N/A	N/A	-0.580
672035060	CIC	capicua transcriptional repressor	-0.579
197386131	Acad10	acyl-CoA dehydrogenase family, member 10	-0.578
564307839	JAG2	jagged 2	-0.573
157823545	WDR54	WD repeat domain 54	-0.573
16758622	IFT172	intraflagellar transport 172	-0.568
672035395	DMWD	DM1 locus, WD repeat containing	-0.566
984107618	N/A	N/A	-0.565
157821925	IFT88	intraflagellar transport 88	-0.563
109470195	TNKS1BP1	tankyrase 1 binding protein 1	-0.562
431916930	N/A	N/A	-0.561
149046941	BEND3	BEN domain containing 3	-0.560
672070787	NSUN3	NOP2/Sun RNA methyltransferase family member 3	-0.560
148701441	N/A	N/A	-0.560

672015904	TMEM131L	transmembrane 131 like	-0.559
564395350	N/A	N/A	-0.559
564394999	CLGN	calmegin	-0.558
675649807	N/A	N/A	-0.555
564310904	N/A	N/A	-0.553
672048447	CABLES2	Cdk5 and Abl enzyme substrate 2	-0.552
564345556	CROT	carnitine O-octanoyltransferase	-0.551
197927123	LYRM7	LYR motif containing 7	-0.549
558611343	MCM3	minichromosome maintenance complex component 3	-0.549
564308822	SLC45A4	solute carrier family 45 member 4	-0.546
50657355	TOP1MT	DNA topoisomerase I mitochondrial	-0.544
568972691	STAT5B	signal transducer and activator of transcription 5B	-0.543
109497496	MMAB	methylmalonic aciduria (cobalamin deficiency) cblB type	-0.542
148674017	NRSN2	neurensin 2	-0.542
48428501	SYNPO	synaptopodin	-0.542
13162329	PIGM	phosphatidylinositol glycan anchor biosynthesis class M	-0.541
149061352	ADAM12	ADAM metallopeptidase domain 12	-0.529
395759219	AQP4	aquaporin 4	-0.525
6978737	CYP1B1	cytochrome P450 family 1 subfamily B member 1	-0.524
149048774	Spry1	sprouty RTK signaling antagonist 1	-0.522
564338026	BCL9	B cell CLL/lymphoma 9	-0.517
149016584	ZNF606	zinc finger protein 606	-0.517
195976802	DNLZ	DNL-type zinc finger	-0.516
149035030	MAFK	MAF bZIP transcription factor K	-0.515
119574218	COL23A1	collagen type XXIII alpha 1 chain	-0.512
9506405	ARPC1B	actin related protein 2/3 complex subunit 1B	-0.511
293347888	SRBD1	S1 RNA binding domain 1	-0.510
148356229	CCND1	cyclin D1	-0.506
564310188	IGDCC4	immunoglobulin superfamily DCC subclass member 4	-0.504
281371499	COL5A2	collagen type V alpha 2 chain	-0.503
564313842	SLC38A10	solute carrier family 38 member 10	-0.503
564302924	TSHZ2	teashirt zinc finger homeobox 2	-0.503
672022667	ARHGAP32	Rho GTPase activating protein 32	-0.502
25742686	ELOVL6	ELOVL fatty acid elongase 6	-0.496

157818909	Zim1	zinc finger, imprinted 1	-0.495
672038342	XYLT1	xylosyltransferase 1	-0.494
392332017	ASPSCR1	ASPSCR1, UBX domain containing tether for SLC2A4	-0.493
62543507	CIB2	calcium and integrin binding family member 2	-0.493
62647202	KRBA1	KRAB-A domain containing 1	-0.492
984126115	N/A	N/A	-0.491
12849161	NRARP	NOTCH regulated ankyrin repeat protein	-0.489
31560385	RPL21	ribosomal protein L21	-0.482
70608094	SLC10A5	solute carrier family 10 member 5	-0.481
564367862	Dst	dystonin	-0.480
260271475	CCHCR1	coiled-coil alpha-helical rod protein 1	-0.478
293340128	MIEF2	mitochondrial elongation factor 2	-0.477
564315753	N/A	N/A	-0.476
19424260	CDC25B	cell division cycle 25B	-0.470
149048674	PEX5L	peroxisomal biogenesis factor 5 like	-0.468
148674444	DBNDD2	dysbindin domain containing 2	-0.466
115392004	GPR17	G protein-coupled receptor 17	-0.466
202070751	RFTN1	raftlin, lipid raft linker 1	-0.465
6754024	GNG4	G protein subunit gamma 4	-0.463
564320452	SAP130	Sin3A associated protein 130	-0.462
694978694	N/A	N/A	-0.462
564324736	L3MBTL3	L3MBTL3, histone methyl-lysine binding protein	-0.461
215490074	SAP18	Sin3A associated protein 18	-0.461
157823259	TMEM229A	transmembrane protein 229A	-0.458
51948480	MTIF2	mitochondrial translational initiation factor 2	-0.457
564302105	ZNF770	zinc finger protein 770	-0.457
392331978	CDR2L	cerebellar degeneration related protein 2 like	-0.456
310703673	GRIN3A	glutamate ionotropic receptor NMDA type subunit 3A	-0.453
41056215	XRCC5	X-ray repair cross complementing 5	-0.452
392353178	SEL1L3	SEL1L family member 3	-0.451
672080674	Myo16	myosin XVI	-0.450
674054416	N/A	N/A	-0.449
82654188	KLF11	Kruppel like factor 11	-0.448
564311658	N/A	N/A	-0.447
13592057	RPL18	ribosomal protein L18	-0.446
57527498	KLC4	kinesin light chain 4	-0.445
78126167	SLC1A2	solute carrier family 1 member 2	-0.445

157820211	ABHD3	abhydrolase domain containing 3	-0.444
392349170	ZFP36L2	ZFP36 ring finger protein like 2	-0.444
672018552	N/A	N/A	-0.442
148692356	ARHGEF1	Rho guanine nucleotide exchange factor 1	-0.441
564345274	FASTK	Fas activated serine/threonine kinase	-0.440
160406706	SH3GL3	SH3 domain containing GRB2 like 3, endophilin A3	-0.440
568926543	KIAA0368	KIAA0368	-0.437
586908220	ARHGAP44	Rho GTPase activating protein 44	-0.434
51854227	GSN	gelsolin	-0.431
635015168	N/A	N/A	-0.431
564386622	AMER2	APC membrane recruitment protein 2	-0.430
672080026	N/A	N/A	-0.430
524964924	N/A	N/A	-0.427
296470851	PABPC1L2A	poly(A) binding protein cytoplasmic 1 like 2A	-0.426
149054848	FOXJ1	forkhead box J1	-0.425
336285191	ZFPM1	zinc finger protein, FOG family member 1	-0.424
157821511	SDR39U1	short chain dehydrogenase/reductase family 39U member 1	-0.423
148706671	RHOQ	ras homolog family member Q	-0.422
564374356	ADAM11	ADAM metallopeptidase domain 11	-0.421
62078551	GNB4	G protein subunit beta 4	-0.421
564357619	ITGB8	integrin subunit beta 8	-0.421
18087805	RPS2	ribosomal protein S2	-0.421
672082323	N/A	N/A	-0.421
199562000	USP40	ubiquitin specific peptidase 40	-0.420
725568549	N/A	N/A	-0.419
564304730	N/A	N/A	-0.418
293348823	SUN2	Sad1 and UNC84 domain containing 2	-0.416
157823399	COG4	component of oligomeric golgi complex 4	-0.415
281604125	Fam50a/LOC100910130	family with sequence similarity 50, member A	-0.412
929981595	NPHP1	nephrocystin 1	-0.412
167900441	FOXK1	forkhead box K1	-0.411
293342200	DGKH	diacylglycerol kinase eta	-0.410
189011647	RNF139	ring finger protein 139	-0.410
148747268	Sik1	salt inducible kinase 1	-0.408
157818005	HPS3	HPS3, biogenesis of lysosomal organelles complex 2 subunit 1	-0.405
157818133	KLHL29	kelch like family member 29	-0.404

807677	N/A	N/A	-0.404
109480433	GNPTAB	N-acetylglucosamine-1-phosphate transferase alpha and beta subunits	-0.403
38454238	Rab15	RAB15, member RAS oncogene family	-0.402
13592117	KLF10	Kruppel like factor 10	-0.400
149028840	N/A	N/A	-0.400
451172073	CHRM3	cholinergic receptor muscarinic 3	-0.399
58865380	STAT2	signal transducer and activator of transcription 2	-0.399
958720315	N/A	N/A	-0.399
672061813	ACSBG1	acyl-CoA synthetase bubblegum family member 1	-0.397
11693172	CALR	calreticulin	-0.396
114052238	FIG4	FIG4 phosphoinositide 5-phosphatase	-0.396
148703340	SERTM1	serine rich and transmembrane domain containing 1	-0.395
198386343	TRPS1	transcriptional repressor GATA binding 1	-0.395
672084347	N/A	N/A	-0.395
56605636	TMEM254	transmembrane protein 254	-0.394
672089580	LOC103694865	TATA-binding protein-associated factor 2N-like	-0.389
564375702	N/A	N/A	-0.389
149034469	GNG7	G protein subunit gamma 7	-0.388
285026497	ICAM5	intercellular adhesion molecule 5	-0.386
524932722	N/A	N/A	-0.386
731271938	N/A	N/A	-0.386
16758502	HCN3	hyperpolarization activated cyclic nucleotide gated potassium channel 3	-0.384
149064803	NHLRC3	NHL repeat containing 3	-0.384
399220341	SLC2A13	solute carrier family 2 member 13	-0.384
205294	ME1	malic enzyme 1	-0.382
149066394	SAMD12	sterile alpha motif domain containing 12	-0.381
672033256	LOC100912904	disks large homolog 5-like	-0.380
171846640	FBLN1	fibulin 1	-0.379
157786892	POP7	POP7 homolog, ribonuclease P/MRP subunit	-0.378
157819227	PRPF31	pre-mRNA processing factor 31	-0.377
55742862	PLIN2	perilipin 2	-0.375
655851627	N/A	N/A	-0.374
672032219	REPS2	RALBP1 associated Eps domain containing 2	-0.373
78486556	C16orf58	chromosome 16 open reading frame 58	-0.372

119624653	TMEM63B	transmembrane protein 63B	-0.372
58865418	SUGP1	SURP and G-patch domain containing 1	-0.371
187282043	TMEM179	transmembrane protein 179	-0.371
217416396	NEURL1B	neuralized E3 ubiquitin protein ligase 1B	-0.370
672060362	ELFN2	extracellular leucine rich repeat and fibronectin type III domain containing 2	-0.369
56605780	PMVK	phosphomevalonate kinase	-0.368
625226717	N/A	N/A	-0.368
880957181	N/A	N/A	-0.368
149066868	MDM1	Mdm1 nuclear protein	-0.367
13591949	GATM	glycine amidinotransferase	-0.365
768018507	MAP1LC3A	microtubule associated protein 1 light chain 3 alpha	-0.365
449784888	ALDH5A1	aldehyde dehydrogenase 5 family member A1	-0.363
298493223	TMEM132B	transmembrane protein 132B	-0.363
589965307	N/A	N/A	-0.362
157821975	ZCCHC24	zinc finger CCHC-type containing 24	-0.361
674082488	N/A	N/A	-0.361
157817545	PTX3	pentraxin 3	-0.360
92110015	RMND1	required for meiotic nuclear division 1 homolog	-0.360
61556910	SNX10	sorting nexin 10	-0.359
564396113	ZCCHC14	zinc finger CCHC-type containing 14	-0.359
33414515	PXK	PX domain containing serine/threonine kinase like	-0.358
635039352	N/A	N/A	-0.356
77404405	TRIL	TLR4 interactor with leucine rich repeats	-0.355
672074685	ILDR2	immunoglobulin like domain containing receptor 2	-0.354
38322759	MET	MET proto-oncogene, receptor tyrosine kinase	-0.354
568968076	PLXNC1	plexin C1	-0.354
209915558	Prrt1	proline-rich transmembrane protein 1	-0.354
162287337	APOE	apolipoprotein E	-0.353
62079143	YIPF2	Yip1 domain family member 2	-0.352
755566690	HUWE1	HECT, UBA and WWE domain containing 1, E3 ubiquitin protein ligase	-0.351
11693162	INSIG1	insulin induced gene 1	-0.351
189491867	Phykpl	5-phosphohydroxy-L-lysine phospho-lyase	-0.351
9437326	SLC4A4	solute carrier family 4 member 4	-0.351
197927216	TBC1D5	TBC1 domain family member 5	-0.351
157818161	AAAS	aladin WD repeat nucleoporin	-0.350

308044487	KIAA0319	KIAA0319	-0.350
210032365	HSP90B1	heat shock protein 90 beta family member 1	-0.349
157822325	MPDU1	mannose-P-dolichol utilization defect 1	-0.349
672044191	TBCK	TBC1 domain containing kinase	-0.349
122065191	ABAT	4-aminobutyrate aminotransferase	-0.348
157821205	PAOX	polyamine oxidase	-0.348
451172098	KCTD1	potassium channel tetramerization domain containing 1	-0.347
672052705	FRRS1L	ferric chelate reductase 1 like	-0.346
817259544	N/A	N/A	-0.346
392342224	N/A	N/A	-0.346
148669431	DNAJC27	DnaJ heat shock protein family (Hsp40) member C27	-0.344
126012523	FBXW9	F-box and WD repeat domain containing 9	-0.344
148700512	NRSN1	neurensin 1	-0.343
564332376	RASGRP2	RAS guanyl releasing protein 2	-0.343
148702917	N/A	N/A	-0.343
149020546	COL5A3	collagen type V alpha 3 chain	-0.341
564389540	MGC116197 (includes others)	similar to RIKEN cDNA 1700001E04	-0.341
77046155	PHYHIP	phytanoyl-CoA 2-hydroxylase interacting protein	-0.341
11119239	SYT13	synaptotagmin 13	-0.341
62388885	YIF1B	Yip1 interacting factor homolog B, membrane trafficking protein	-0.341
562831120	N/A	N/A	-0.341
625292335	N/A	N/A	-0.340
290677867	BCAS3	BCAS3, microtubule associated cell migration factor	-0.339
149063131	GALNT17	polypeptide N-acetylgalactosaminyltransferase 17	-0.339
77157795	MAL2	mal, T cell differentiation protein 2 (gene/pseudogene)	-0.338
38051964	TXNDC9	thioredoxin domain containing 9	-0.338
157821439	CPNE5	copine 5	-0.337
157820489	NEURL1	neuralized E3 ubiquitin protein ligase 1	-0.337
149058577	Ppfia4	protein tyrosine phosphatase, receptor type, f polypeptide (PTPRF), interacting protein (liprin), alpha 4	-0.334
472380424	N/A	N/A	-0.334

1334149	N/A	N/A	-0.331
537237916	N/A	N/A	-0.331
672014573	N/A	N/A	-0.330
6981208	NR3C2	nuclear receptor subfamily 3 group C member 2	-0.328
148681013	SYT14	synaptotagmin 14	-0.328
6981010	Hba1/Hba2	hemoglobin, alpha 1	-0.327
564320789	SEH1L	SEH1 like nucleoporin	-0.327
537193698	N/A	N/A	-0.327
157822543	DSCAML1	DS cell adhesion molecule like 1	-0.326
13540703	PDE1A	phosphodiesterase 1A	-0.326
13786144	PREP	prolyl endopeptidase	-0.325
300797330	PTPRU	protein tyrosine phosphatase, receptor type U	-0.323
112350	N/A	N/A	-0.323
672026875	N/A	N/A	-0.323
257096063	IGLON5	IgLON family member 5	-0.321
149041432	THY1	Thy-1 cell surface antigen	-0.321
148702078	CPSF3	cleavage and polyadenylation specific factor 3	-0.320
149041576	REXO2	RNA exonuclease 2	-0.319
392334157	SEMA6A	semaphorin 6A	-0.318
158186726	FUCA1	alpha-L-fucosidase 1	-0.317
33286888	GJA1	gap junction protein alpha 1	-0.317
16758712	PDIA4	protein disulfide isomerase family A member 4	-0.317
57114344	UHRF1	ubiquitin like with PHD and ring finger domains 1	-0.317
74315992	INHBB	inhibin beta B subunit	-0.315
564310645	PLXNB1	plexin B1	-0.315
672060041	N/A	N/A	-0.315
928154944	HNRNPC	heterogeneous nuclear ribonucleoprotein C (C1/C2)	-0.314
672076306	NWD2	NACHT and WD repeat domain containing 2	-0.314
346989661	CPEB2	cytoplasmic polyadenylation element binding protein 2	-0.312
288541382	DIS3L2	DIS3 like 3'-5' exoribonuclease 2	-0.312
149063353	IFT81	intraflagellar transport 81	-0.311
564358836	NT5DC3	5'-nucleotidase domain containing 3	-0.311
209364556	BACH2	BTB domain and CNC homolog 2	-0.310
13994179	SLC24A2	solute carrier family 24 member 2	-0.310
149041833	N/A	N/A	-0.310
25453420	GSTP1	glutathione S-transferase pi 1	-0.309
564389730	PLAT	plasminogen activator, tissue type	-0.309

38259192	TOP2A	DNA topoisomerase II alpha	-0.309
672062144	FAM219B	family with sequence similarity 219 member B	-0.308
6981168	LPL	lipoprotein lipase	-0.308
109480098	SMARCC2	SWI/SNF related, matrix associated, actin dependent regulator of chromatin subfamily c member 2	-0.307
672043249	DENND4B	DENN domain containing 4B	-0.306
269954719	JAZF1	JAZF zinc finger 1	-0.305
752420454	N/A	N/A	-0.305
123405	POU3F1	POU class 3 homeobox 1	-0.304
426357412	N/A	N/A	-0.304
564399600	PHKA1	phosphorylase kinase regulatory subunit alpha 1	-0.303
564360941	N/A	N/A	-0.302
62078897	NCLN	nicalin	-0.301
51948488	SIRT5	sirtuin 5	-0.301
672024549	SLX4	SLX4 structure-specific endonuclease subunit	-0.301
927191125	N/A	N/A	-0.299
20301974	VMP1	vacuole membrane protein 1	-0.298
27229055	HYPK	huntingtin interacting protein K	-0.297
564333746	MMS19	MMS19 homolog, cytosolic iron-sulfur assembly component	-0.297
564307561	RPS6KL1	ribosomal protein S6 kinase like 1	-0.297
686661093	SLC24A3	solute carrier family 24 member 3	-0.296
13592129	DOC2B	double C2 domain beta	-0.295
74201328	ST6GALNAC4	ST6 N-acetylgalactosaminide alpha-2,6-sialyltransferase 4	-0.295
157819209	ZNF575	zinc finger protein 575	-0.295
198442897	AFG3L2	AFG3 like matrix AAA peptidase subunit 2	-0.294
747811827	HID1	HID1 domain containing	-0.294
404434384	GALNT11	polypeptide N-acetylgalactosaminyltransferase 11	-0.293
564357258	CCDC85C	coiled-coil domain containing 85C	-0.292
672085486	EGLN1	egl-9 family hypoxia inducible factor 1	-0.291
157786874	Fmnl1	formin-like 1	-0.291
13027442	ARHGEF11	Rho guanine nucleotide exchange factor 11	-0.288
161086978	CAPZA1	capping actin protein of muscle Z-line alpha subunit 1	-0.288
61556795	IGFBP7	insulin like growth factor binding protein 7	-0.288
60359932	PPM1H	protein phosphatase, Mg2+/Mn2+ dependent 1H	-0.288

75905809	AKAP12	A-kinase anchoring protein 12	-0.286
157819725	CEP131	centrosomal protein 131	-0.286
9506523	CSPG5	chondroitin sulfate proteoglycan 5	-0.286
564318875	PSD3	pleckstrin and Sec7 domain containing 3	-0.286
309243082	PTPRJ	protein tyrosine phosphatase, receptor type J	-0.286
954249788	N/A	N/A	-0.286
28972652	SLC12A5	solute carrier family 12 member 5	-0.285
50356003	SCP2	sterol carrier protein 2	-0.284
564396646	VARS2	valyl-tRNA synthetase 2, mitochondrial	-0.284
42491372	ERMP1	endoplasmic reticulum metallopeptidase 1	-0.283
157820833	HERC3	HECT and RLD domain containing E3 ubiquitin protein ligase 3	-0.283
149024348	RAP1GAP	RAP1 GTPase activating protein	-0.283
61556829	RIT2	Ras like without CAAX 2	-0.283
16758208	SRM	spermidine synthase	-0.283
158749584	SUCLA2	succinate-CoA ligase ADP-forming beta subunit	-0.283
672058250	CKAP4	cytoskeleton associated protein 4	-0.282
73661200	SPRN	shadow of prion protein	-0.282
293343483	NHSL1	NHS like 1	-0.281
511925477	N/A	N/A	-0.281
537212823	N/A	N/A	-0.281
672057964	N/A	N/A	-0.280
149022622	ACP2	acid phosphatase 2, lysosomal	-0.278
157820195	C1QTNF4	C1q and TNF related 4	-0.278
564360297	N/A	N/A	-0.277
71725385	DIRAS2	DIRAS family GTPase 2	-0.276
189027115	AIDA	axin interactor, dorsalization associated	-0.275
205755	TAGLN3	transgelin 3	-0.275
564394868	CC2D1A	coiled-coil and C2 domain containing 1A	-0.273
13591981	LSS	lanosterol synthase	-0.273
128485638	PLOD3	procollagen-lysine,2-oxoglutarate 5-dioxygenase 3	-0.273
589922499	N/A	N/A	-0.273
149049455	Ptms	parathymosin	-0.272
300797936	ACTR3B	ARP3 actin related protein 3 homolog B	-0.271
145279186	GAS6	growth arrest specific 6	-0.271
157820049	LRFN5	leucine rich repeat and fibronectin type III domain containing 5	-0.271
9055178	MPC1	mitochondrial pyruvate carrier 1	-0.271

16758726	SLC17A7	solute carrier family 17 member 7	-0.271
50878267	HCN2	hyperpolarization activated cyclic nucleotide gated potassium and sodium channel 2	-0.270
149034474	TIMM13	translocase of inner mitochondrial membrane 13	-0.270
672055089	N/A	N/A	-0.270
50510855	RIMKLB	ribosomal modification protein rimK like family member B	-0.269
160333093	TPRG1L	tumor protein p63 regulated 1 like	-0.269
157816923	MRPL4	mitochondrial ribosomal protein L4	-0.268
77404265	JAM2	junctional adhesion molecule 2	-0.267
148671603	LRP11	LDL receptor related protein 11	-0.267
358030320	DMTN	dematin actin binding protein	-0.266
19705437	EPHA7	EPH receptor A7	-0.266
71122474	PPA1	pyrophosphatase (inorganic) 1	-0.266
8394496	TYRO3	TYRO3 protein tyrosine kinase	-0.266
157822933	ZNF385A	zinc finger protein 385A	-0.266
79750129	CAMK1D	calcium/calmodulin dependent protein kinase ID	-0.265
155369295	KIAA1841	KIAA1841	-0.265
13489067	NSF	N-ethylmaleimide sensitive factor, vesicle fusing ATPase	-0.265
148678721	SOX5	SRY-box 5	-0.265
861445795	N/A	N/A	-0.265
564382292	ANGEL2	angel homolog 2	-0.263
115292425	KIRREL3	kirre like nephrin family adhesion molecule 3	-0.263
13242271	SLC6A11	solute carrier family 6 member 11	-0.263
16758186	SLCO1C1	solute carrier organic anion transporter family member 1C1	-0.263
795289844	N/A	N/A	-0.263
157819077	TRIM37	tripartite motif containing 37	-0.262
148666046	FAM84A	family with sequence similarity 84 member A	-0.261
148674172	TP53INP2	tumor protein p53 inducible nuclear protein 2	-0.261
564344879	N/A	N/A	-0.261
62078931	PAQR8	progestin and adipoQ receptor family member 8	-0.260
157786976	RGL1	ral guanine nucleotide dissociation stimulator like 1	-0.259
74179798	PCSK2	proprotein convertase subtilisin/kexin type 2	-0.258
747019224	SRCAP	Snf2 related CREBBP activator protein	-0.258
564371892	N/A	N/A	-0.258

564378247	CCZ1/CCZ1B	CCZ1 homolog B, vacuolar protein trafficking and biogenesis associated	-0.257
564351380	CDK5RAP2	CDK5 regulatory subunit associated protein 2	-0.257
9507083	SEMA4F	ssemaphorin 4F	-0.257
157822443	AGPAT3	1-acylglycerol-3-phosphate O-acyltransferase 3	-0.256
56388748	Anp32a	acidic (leucine-rich) nuclear phosphoprotein 32 family, member A	-0.256
157952208	BAG1	BCL2 associated athanogene 1	-0.256
404312657	NCAN	neurocan	-0.256
940716821	N/A	N/A	-0.255
40254752	PGK1	phosphoglycerate kinase 1	-0.254
674094346	N/A	N/A	-0.254
3043568	IQSEC2	IQ motif and Sec7 domain 2	-0.253
77539756	MED24	mediator complex subunit 24	-0.253
672026416	PRR36	proline rich 36	-0.252
157824000	C8orf46	chromosome 8 open reading frame 46	-0.251
157817710	FER	FER tyrosine kinase	-0.251
564337070	N/A	N/A	-0.251
148693489	Nrgn	neurogranin	-0.250
564377215	N/A	N/A	-0.250
16758080	COL1A2	collagen type I alpha 2 chain	-0.249
8393823	Nefm	neurofilament, medium polypeptide	-0.249
300795140	TAF1	TATA-box binding protein associated factor 1	-0.248
672013187	DMWD	DM1 locus, WD repeat containing	-0.247
158749644	MCM6	minichromosome maintenance complex component 6	-0.247
22902132	RBM10	RNA binding motif protein 10	-0.247
22219454	SLC16A2	solute carrier family 16 member 2	-0.247
564343565	N/A	N/A	-0.247
149050766	CAD	carbamoyl-phosphate synthetase 2, aspartate transcarbamylase, and dihydroorotase	-0.246
13386106	NUDT21	nudix hydrolase 21	-0.246
913512819	N/A	N/A	-0.246
21687010	CACNG4	calcium voltage-gated channel auxiliary subunit gamma 4	-0.245
795351870	N/A	N/A	-0.245
215272398	HIP1	huntingtin interacting protein 1	-0.244
149054795	RGD1309310	similar to mKIAA0195 protein	-0.244
931568103	N/A	N/A	-0.244
15805026	ZFAND6	zinc finger AN1-type containing 6	-0.243

15011857	SELENOP	selenoprotein P	-0.241
672017118	N/A	N/A	-0.241
564368926	N/A	N/A	-0.241
965962418	N/A	N/A	-0.240
564329074	UNC45A	unc-45 myosin chaperone A	-0.238
6978613	CCK	cholecystokinin	-0.237
157818983	SIRT7	sirtuin 7	-0.236
821419425	N/A	N/A	-0.236
162287198	HSD17B4	hydroxysteroid 17-beta dehydrogenase 4	-0.235
148683194	INTS3	integrator complex subunit 3	-0.235
300797239	MARK4	microtubule affinity regulating kinase 4	-0.234
77627981	SHANK1	SH3 and multiple ankyrin repeat domains 1	-0.233
16758840	CRYM	crystallin mu	-0.230
157819371	SYNGR3	synaptogyrin 3	-0.229
54234046	CST3	cystatin C	-0.228
149037913	N/A	N/A	-0.227
672079582	N/A	N/A	-0.226
148689145	CPNE4	copine 4	-0.225
283046651	PTPRZ1	protein tyrosine phosphatase, receptor type Z1	-0.225
880942118	N/A	N/A	-0.225
142349612	GLUL	glutamate-ammonia ligase	-0.224
18777747	MGEA5	meningioma expressed antigen 5 (hyaluronidase)	-0.224
148710162	SLC8A2	solute carrier family 8 member A2	-0.224
157819753	RCN1	reticulocalbin 1	-0.223
157819361	TTYH1	tweety family member 1	-0.223
149017194	KDM3B	lysine demethylase 3B	-0.221
300794237	LIMCH1	LIM and calponin homology domains 1	-0.221
19705535	PACS1	phosphofurin acidic cluster sorting protein 1	-0.220
300793998	SHISA6	shisa family member 6	-0.220
149066619	B4GALNT1	beta-1,4-N-acetyl-galactosaminyltransferase 1	-0.219
255683359	FBXL17	F-box and leucine rich repeat protein 17	-0.219
157822175	NDUFB10	NADH:ubiquinone oxidoreductase subunit B10	-0.219
392334416	TCF25	transcription factor 25	-0.219
54312094	DAGLA	diacylglycerol lipase alpha	-0.218
6978888	GFRA1	GDNF family receptor alpha 1	-0.218
11560055	KHDRBS3	KH RNA binding domain containing, signal transduction associated 3	-0.217
672046063	Slc25a12	solute carrier family 25 member 12	-0.217
20376818	WBP2	WW domain binding protein 2	-0.217

16758160	BNIP3	BCL2 interacting protein 3	-0.215
14091777	Chn1	chimerin 1	-0.214
148687519	CALN1	calneuron 1	-0.212
68341995	NDUFS4	NADH:ubiquinone oxidoreductase subunit S4	-0.212
157817839	SEMA5A	semaphorin 5A	-0.212
564342542	MAP1A	microtubule associated protein 1A	-0.211
564344373	ZMYND8	zinc finger MYND-type containing 8	-0.211
57526927	LARS	leucyl-tRNA synthetase	-0.210
564385256	N/A	N/A	-0.210
568980664	ATG2B	autophagy related 2B	-0.209
148747253	ATP1B1	ATPase Na+/K+ transporting subunit beta 1	-0.209
149015822	SLC9A6	solute carrier family 9 member A6	-0.209
149023410	SNAP25	synaptosome associated protein 25	-0.209
149016843	N/A	N/A	-0.209
149022319	AGPS	alkylglycerone phosphate synthase	-0.208
149067833	ALDOA	aldolase, fructose-bisphosphate A	-0.208
160333459	Cox6c	cytochrome c oxidase subunit VIc	-0.208
672057971	MEX3D	mex-3 RNA binding family member D	-0.208
972956729	N/A	N/A	-0.207
39104626	CAMK2A	calcium/calmodulin dependent protein kinase II alpha	-0.206
195972823	TMEM181	transmembrane protein 181	-0.206
564322686	N/A	N/A	-0.206
672036086	ARHGAP33	Rho GTPase activating protein 33	-0.205
594629505	N/A	N/A	-0.205
50511338	FUT8	fucosyltransferase 8	-0.204
157821895	GDAP1	ganglioside induced differentiation associated protein 1	-0.204
71896543	SHANK3	SH3 and multiple ankyrin repeat domains 3	-0.204
300796933	WNK2	WNK lysine deficient protein kinase 2	-0.204
157819955	DTD1	D-tyrosyl-tRNA deacylase 1	-0.203
8393490	GRM5	glutamate metabotropic receptor 5	-0.203
568999199	MEA1	male-enhanced antigen 1	-0.203
157786690	PRKCA	protein kinase C alpha	-0.203
686735522	N/A	N/A	-0.203
817280433	N/A	N/A	-0.203
823430370	N/A	N/A	-0.203
148706945	TXNIP	thioredoxin interacting protein	-0.202
564377500	N/A	N/A	-0.202
554543965	N/A	N/A	-0.202

564370831	CASKIN1	CASK interacting protein 1	-0.201
386781556	YBX3	Y-box binding protein 3	-0.201
149043701	N/A	N/A	-0.201
564388576	CILP2	cartilage intermediate layer protein 2	-0.200
149041736	RCN2	reticulocalbin 2	-0.200
6978545	ATP1A2	ATPase Na+/K+ transporting subunit alpha 2	-0.199
148707802	DARS	aspartyl-tRNA synthetase	-0.199
198278463	MAST3	microtubule associated serine/threonine kinase 3	-0.199
59858990	UNC13A	unc-13 homolog A	-0.199
55741502	ACAT2	acetyl-CoA acetyltransferase 2	-0.198
50511245	MEX3B	mex-3 RNA binding family member B	-0.198
157817420	NRIP3	nuclear receptor interacting protein 3	-0.198
109472884	UBE3C	ubiquitin protein ligase E3C	-0.198
13592131	DGKZ	diacylglycerol kinase zeta	-0.197
672036872	PRR12	proline rich 12	-0.197
13929208	Scd2	stearoyl-Coenzyme A desaturase 2	-0.197
664703871	N/A	N/A	-0.197
227913	N/A	N/A	-0.197
672040941	ATRNL1	attractin like 1	-0.196
157818219	MMP15	matrix metallopeptidase 15	-0.195
29789369	PTPRG	protein tyrosine phosphatase, receptor type G	-0.195
60360078	SHTN1	shootin 1	-0.195
584277046	SLC1A3	solute carrier family 1 member 3	-0.195
84000579	FTL	ferritin light chain	-0.194
56605990	LRPPRC	leucine rich pentatricopeptide repeat containing	-0.194
62078997	WDR1	WD repeat domain 1	-0.194
926693529	N/A	N/A	-0.194
31542604	ERC1	ELKS/RAB6-interacting/CAST family member 1	-0.193
41529837	JUP	junction plakoglobin	-0.193
38454208	KCTD13	potassium channel tetramerization domain containing 13	-0.193
58865508	SMARCD3	SWI/SNF related, matrix associated, actin dependent regulator of chromatin, subfamily d, member 3	-0.193
564381813	N/A	N/A	-0.192
8394227	PTPRO	protein tyrosine phosphatase, receptor type O	-0.191
56605704	SERINC3	serine incorporator 3	-0.191
820973857	N/A	N/A	-0.191

149032539	HECW1	HECT, C2 and WW domain containing E3 ubiquitin protein ligase 1	-0.190
148695758	CAPRIN1	cell cycle associated protein 1	-0.188
6978751	CYP51A1	cytochrome P450 family 51 subfamily A member 1	-0.188
55249662	Masp1	mannan-binding lectin serine peptidase 1	-0.188
57114294	SLC39A7	solute carrier family 39 member 7	-0.187
149053224	PFN1	profilin 1	-0.186
157823887	MLC1	megalencephalic leukoencephalopathy with subcortical cysts 1	-0.185
293346766	TCAF1	TRPM8 channel associated factor 1	-0.185
795272288	N/A	N/A	-0.185
259155312	AGAP2	ArfGAP with GTPase domain, ankyrin repeat and PH domain 2	-0.184
182888557	FAM57B	family with sequence similarity 57 member B	-0.184
568941844	IQSEC3	IQ motif and Sec7 domain 3	-0.184
8394328	SOD1	superoxide dismutase 1	-0.184
83267872	ADNP	activity dependent neuroprotector homeobox	-0.183
568974914	SRCIN1	SRC kinase signaling inhibitor 1	-0.183
402794619	ADAM10	ADAM metallopeptidase domain 10	-0.182
471434853	BCL11B	B cell CLL/lymphoma 11B	-0.182
564324344	LOC363306 (includes others)	hypothetical protein LOC363306	-0.182
148692349	ATP1A3	ATPase Na+/K+ transporting subunit alpha 3	-0.181
257796229	SYNGAP1	synaptic Ras GTPase activating protein 1	-0.181
164519053	FAM131B	family with sequence similarity 131 member B	-0.179
149036745	FBLN2	fibulin 2	-0.178
148698650	NASP	nuclear autoantigenic sperm protein	-0.177
148696327	PTPRA	protein tyrosine phosphatase, receptor type A	-0.177
564364792	SNAP91	synaptosome associated protein 91	-0.176
6756037	YWHAH	tyrosine 3-monooxygenase/tryptophan 5-monooxygenase activation protein eta	-0.176
6681095	CYCS	cytochrome c, somatic	-0.174
564396135	KIAA0513	KIAA0513	-0.174
830015746	N/A	N/A	-0.174
564396731	N/A	N/A	-0.174
625196919	N/A	N/A	-0.173
149028426	PCSK1N	proprotein convertase subtilisin/kexin type 1 inhibitor	-0.171
672058640	N/A	N/A	-0.171

9665227	DLG4	discs large MAGUK scaffold protein 4	-0.170
183396771	HSPD1	heat shock protein family D (Hsp60) member 1	-0.170
744617549	N/A	N/A	-0.170
32527699	LOC301444	pseudogene for diazepam binding inhibitor 1	-0.168
109475418	FOXO6	forkhead box O6	-0.166
564379471	RPH3A	rabphilin 3A	-0.165
70778983	SFPQ	splicing factor proline and glutamine rich	-0.165
77404395	SND1	staphylococcal nuclease and tudor domain containing 1	-0.165
148700340	N/A	N/A	-0.165
6739575	ARL6IP1	ADP ribosylation factor like GTPase 6 interacting protein 1	-0.164
625214802	N/A	N/A	-0.164
157786960	PIK3C2B	phosphatidylinositol-4-phosphate 3-kinase catalytic subunit type 2 beta	-0.163
8850229	MAP6	microtubule associated protein 6	-0.161
164519074	PDE4D	phosphodiesterase 4D	-0.160
158186636	PDGFRA	platelet derived growth factor receptor alpha	-0.160
8394502	UBC	ubiquitin C	-0.160
564347713	N/A	N/A	-0.160
47271544	FKBP1A	FK506 binding protein 1A	-0.159
6980956	GLUD1	glutamate dehydrogenase 1	-0.159
197209847	JAK1	Janus kinase 1	-0.159
149019802	SEPT5	septin 5	-0.159
148673176	FABP7	fatty acid binding protein 7	-0.158
823419536	N/A	N/A	-0.158
17985949	HBB	hemoglobin subunit beta	-0.157
156139151	PDS5B	PDS5 cohesin associated factor B	-0.157
37359962	PLPPR4	phospholipid phosphatase related 4	-0.157
674071606	N/A	N/A	-0.157
58865384	NDUFS2	NADH:ubiquinone oxidoreductase core subunit S2	-0.156
16923964	CNTN1	contactin 1	-0.155
83404987	FTH1	ferritin heavy chain 1	-0.155
744616901	N/A	N/A	-0.155
564329392	FLNA	filamin A	-0.154
634833336	N/A	N/A	-0.154
641706489	N/A	N/A	-0.154
201066380	FSCN1	fascin actin-bundling protein 1	-0.153
13027448	ZNF354C	zinc finger protein 354C	-0.153

97537309	SYNJ1	synaptojanin 1	-0.152
396941666	Dync1i2	dynein cytoplasmic 1 intermediate chain 2	-0.151
58865906	PLD3	phospholipase D family member 3	-0.151
148679695	COX4I1	cytochrome c oxidase subunit 4I1	-0.150
60360580	OGDH	oxoglutarate dehydrogenase	-0.150
56799390	ATP1B2	ATPase Na+/K+ transporting subunit beta 2	-0.149
149050122	KCTD12	potassium channel tetramerization domain containing 12	-0.149
56388799	CKB	creatine kinase B	-0.148
157823333	FBXO41	F-box protein 41	-0.148
537137169	N/A	N/A	-0.148
71043650	SRPK1	SRSF protein kinase 1	-0.146
564301979	CKAP5	cytoskeleton associated protein 5	-0.145
187469796	MBD3	methyl-CpG binding domain protein 3	-0.145
564394830	NACC1	nucleus accumbens associated 1	-0.145
537235061	N/A	N/A	-0.145
924184038	INA	internexin neuronal intermediate filament protein alpha	-0.144
823419836	N/A	N/A	-0.144
625184908	N/A	N/A	-0.144
299829287	DISP2	dispatched RND transporter family member 2	-0.143
157818467	HSPA12A	heat shock protein family A (Hsp70) member 12A	-0.143
403044506	SUPT5H	SPT5 homolog, DSIF elongation factor subunit	-0.143
815891312	CACNA1G	calcium voltage-gated channel subunit alpha1 G	-0.142
564368910	FN1	fibronectin 1	-0.142
55741681	ITM2B	integral membrane protein 2B	-0.142
148669961	SLC6A17	solute carrier family 6 member 17	-0.142
58865922	ZFAND3	zinc finger AN1-type containing 3	-0.142
537236584	N/A	N/A	-0.142
40254595	DPYSL2	dihydropyrimidinase like 2	-0.141
953851560	N/A	N/A	-0.141
564352388	N/A	N/A	-0.141
112421036	POU3F3	POU class 3 homeobox 3	-0.139
74224296	RNF10	ring finger protein 10	-0.139
148689290	CAMKV	CaM kinase like vesicle associated	-0.137
56605688	USP14	ubiquitin specific peptidase 14	-0.137
16923990	EPN1	epsin 1	-0.134
13929002	PFKM	phosphofructokinase, muscle	-0.134
149036434	N/A	N/A	-0.134

148673282	CCNI	cyclin I	-0.133
38649320	ENO1	enolase 1	-0.133
270288782	REEP5	receptor accessory protein 5	-0.132
926708692	N/A	N/A	-0.132
507617067	N/A	N/A	-0.132
672044124	N/A	N/A	-0.132
157819095	PNMA8B	PNMA family member 8B	-0.131
564379895	SEZ6L	seizure related 6 homolog like	-0.130
157824115	AGAP3	ArfGAP with GTPase domain, ankyrin repeat and PH domain 3	-0.129
407728599	CTNND2	catenin delta 2	-0.129
39930503	ATP5F1C	ATP synthase F1 subunit gamma	-0.128
25453410	CACNA1B	calcium voltage-gated channel subunit alpha1 B	-0.128
25282419	CANX	calnexin	-0.127
208022666	CELF5	CUGBP Elav-like family member 5	-0.126
564396924	PRRC2A	proline rich coiled-coil 2A	-0.126
569007927	PSD	pleckstrin and Sec7 domain containing	-0.126
254028210	CMIP	c-Maf inducing protein	-0.125
25742763	HSPA5	heat shock protein family A (Hsp70) member 5	-0.125
564304046	PLXNA1	plexin A1	-0.125
253683488	NTRK2	neurotrophic receptor tyrosine kinase 2	-0.124
50510949	N/A	N/A	-0.124
51172604	JPH4	junctophilin 4	-0.123
524962788	N/A	N/A	-0.123
157820001	AGTPBP1	ATP/GTP binding protein 1	-0.122
564398462	Slc9a7	solute carrier family 9 member A7	-0.122
953875962	N/A	N/A	-0.122
60360518	KIF5A	kinesin family member 5A	-0.119
60678292	Hba1/Hba2	hemoglobin, alpha 1	-0.117
68534276	NSMCE3	NSE3 homolog, SMC5-SMC6 complex component	-0.117
281306763	NTN3	netrin 3	-0.117
12667448	Syt7	synaptotagmin 7	-0.115
634876791	N/A	N/A	-0.113
585689575	N/A	N/A	-0.113
148747541	HNRNPU	heterogeneous nuclear ribonucleoprotein U	-0.109
68341973	WASF1	WAS protein family member 1	-0.109
472359423	N/A	N/A	-0.109
166235165	SYP	synaptophysin	-0.108
148678962	N/A	N/A	-0.108

672059390	N/A	N/A	-0.108
119614889	DYNLL2	dynein light chain LC8-type 2	-0.107
158186672	Nedd4	neural precursor cell expressed, developmentally down-regulated 4	-0.107
32189355	SLC25A4	solute carrier family 25 member 4	-0.107
61557085	SPTBN1	spectrin beta, non-erythrocytic 1	-0.107
354496829	N/A	N/A	-0.107
564378945	N/A	N/A	-0.106
149043744	CABIN1	calcineurin binding protein 1	-0.104
55249691	CPE	carboxypeptidase E	-0.103
564370968	MAPK8IP3	mitogen-activated protein kinase 8 interacting protein 3	-0.103
62078555	MLLT11	MLLT11, transcription factor 7 cofactor	-0.103
672035558	NOVA2	NOVA alternative splicing regulator 2	-0.103
149058216	KIFAP3	kinesin associated protein 3	-0.102
71143096	PCDHGC3	protocadherin gamma subfamily C, 3	-0.102
156231065	PHACTR1	phosphatase and actin regulator 1	-0.099
148699288	N/A	N/A	-0.099
149028085	BAG6	BCL2 associated athanogene 6	-0.097
254281247	EVL	Enah/Vasp-like	-0.097
77404238	GABBR1	gamma-aminobutyric acid type B receptor subunit 1	-0.097
568963507	MAP4	microtubule associated protein 4	-0.097
402534517	EPB41L1	erythrocyte membrane protein band 4.1 like 1	-0.096
149016331	NCL	nucleolin	-0.096
281340051	N/A	N/A	-0.096
77415383	HSPA8	heat shock protein family A (Hsp70) member 8	-0.090
674052018	N/A	N/A	-0.090
672050570	N/A	N/A	-0.090
744542371	N/A	N/A	-0.089
6981574	SPARC	secreted protein acidic and cysteine rich	-0.088
149025239	VASH1	vasohibin 1	-0.088
635067979	N/A	N/A	-0.085
884928897	N/A	N/A	-0.084
148747194	SLC16A7	solute carrier family 16 member 7	-0.080
149029718	ATP5F1B	ATP synthase F1 subunit beta	-0.076
148679159	GNAO1	G protein subunit alpha o1	-0.072
149065907	NPTXR	neuronal pentraxin receptor	-0.071
676282501	N/A	N/A	-0.071
672068312	N/A	N/A	-0.071

755472674	N/A	N/A	-0.064
731456449	N/A	N/A	-0.057
568935291	EPHA5	EPH receptor A5	-0.055
29789269	GRIA1	glutamate ionotropic receptor AMPA type subunit 1	-0.052
676279510	N/A	N/A	-0.052
11693176	RPLP0	ribosomal protein lateral stalk subunit P0	-0.051
586556217	N/A	N/A	-0.050
564378965	N/A	N/A	-0.047
149042205	N/A	N/A	-0.046
28189917	Ubb	ubiquitin B	-0.043
568931380	CHD5	chromodomain helicase DNA binding protein 5	-0.036
585192925	N/A	N/A	-0.036
6978673	CNR1	cannabinoid receptor 1	-0.023
386869319	LRRC75B	leucine rich repeat containing 75B	-0.020
157820753	MDGA1	MAM domain containing glycosylphosphatidylinositol anchor 1	-0.002
157823691	SPOCK2	SPARC/osteonectin, cwcv and kazal like domains proteoglycan 2	0.001
16758706	NRXN2	neurexin 2	0.005
564327948	JOSD2	Josephin domain containing 2	0.037
672065610	ZNF142	zinc finger protein 142	0.047
148705576	CRMP1	collapsin response mediator protein 1	0.048
31543764	SPTAN1	spectrin alpha, non-erythrocytic 1	0.048
532003341	N/A	N/A	0.050
20376820	MFN1	mitofusin 1	0.052
9507011	PTPRS	protein tyrosine phosphatase, receptor type S	0.052
803119291	N/A	N/A	0.054
8393296	EEF2	eukaryotic translation elongation factor 2	0.057
672013322	LOC103689966	MARCKS-related protein-like	0.059
998662027	N/A	N/A	0.062
30017419	NREP	neuronal regeneration related protein	0.063
803119291	N/A	N/A	0.065
148674292	NNAT	neuronatin	0.069
564339412	LRRC40	leucine rich repeat containing 40	0.073
655660415	N/A	N/A	0.074
12847552	H3F3A/H3F3B	H3 histone family member 3A	0.075
564354448	AGRN	agrin	0.077
564358123	MIDN	midnolin	0.080
672070615	TTC3	tetratricopeptide repeat domain 3	0.081

625258416	N/A	N/A	0.081
488547912	N/A	N/A	0.083
296491570	N/A	N/A	0.084
880915307	N/A	N/A	0.084
672047825	N/A	N/A	0.084
829910413	N/A	N/A	0.085
158749620	MAP1B	microtubule associated protein 1B	0.089
30017437	GPM6A	glycoprotein M6A	0.092
207318	TMSB10/TMSB4X	thymosin beta 4, X-linked	0.092
537229315	N/A	N/A	0.092
820973213	N/A	N/A	0.092
149030791	GPRASP1	G protein-coupled receptor associated sorting protein 1	0.093
460838694	Srrm2	serine/arginine repetitive matrix 2	0.096
208973284	MAP9	microtubule associated protein 9	0.098
148691289	TUBB	tubulin beta class I	0.103
564386141	ACIN1	apoptotic chromatin condensation inducer 1	0.104
149059533	NFIB	nuclear factor I B	0.105
197252056	MED1	mediator complex subunit 1	0.106
564356550	PCNX1	pecanex homolog 1	0.107
640823357	N/A	N/A	0.107
403310686	SOX4	SRY-box 4	0.109
198278505	RPL7	ribosomal protein L7	0.111
155369307	MTFR1	mitochondrial fission regulator 1	0.112
148698430	N/A	N/A	0.112
744598167	N/A	N/A	0.112
564301352	PRRC2B	proline rich coiled-coil 2B	0.113
198278535	TNC	tenascin C	0.113
667481282	N/A	N/A	0.113
158749559	BSN	bassoon presynaptic cytomatrix protein	0.114
545838484	N/A	N/A	0.118
672041858	PKIA	cAMP-dependent protein kinase inhibitor alpha	0.119
83267872	ADNP	activity dependent neuroprotector homeobox	0.123
50510427	IP6K1	inositol hexakisphosphate kinase 1	0.123
27465617	ABI2	abl interactor 2	0.124
568939281	FRY	FRY microtubule binding protein	0.124
189163518	MBOAT2	membrane bound O-acyltransferase domain containing 2	0.125
585165276	N/A	N/A	0.127
564390508	NSD1	nuclear receptor binding SET domain protein 1	0.128

300795060	SRGAP3	SLIT-ROBO Rho GTPase activating protein 3	0.128
179074	SRSF1	serine and arginine rich splicing factor 1	0.129
201066348	PEAR1	platelet endothelial aggregation receptor 1	0.131
18041977	Serbp1	Serpine1 mRNA binding protein 1	0.132
241666404	EPHA4	EPH receptor A4	0.133
8394158	FASN	fatty acid synthase	0.133
283837871	LRP1	LDL receptor related protein 1	0.133
312836802	SPAG9	sperm associated antigen 9	0.133
242397499	DCHS1	dachsous cadherin-related 1	0.134
158631164	IGSF3	immunoglobulin superfamily member 3	0.134
33636726	SERINC1	serine incorporator 1	0.134
332245592	N/A	N/A	0.134
821013078	N/A	N/A	0.134
115312278	MECP2	methyl-CpG binding protein 2	0.135
998662027	N/A	N/A	0.135
672038527	PLEKHA1	pleckstrin homology domain containing A1	0.136
564353622	UBR4	ubiquitin protein ligase E3 component n-recognin 4	0.136
672055431	SPAST	spastin	0.138
924859455	NEXMIF	neurite extension and migration factor	0.139
171543899	PLXNA4	plexin A4	0.139
300390195	Akap9	A kinase (PRKA) anchor protein (yotiao) 9	0.141
213511844	ALG2	ALG2, alpha-1,3/1,6-mannosyltransferase	0.141
281371335	WDR6	WD repeat domain 6	0.141
60359872	G3BP1	G3BP stress granule assembly factor 1	0.142
672041947	ZNF704	zinc finger protein 704	0.143
157819885	SETD5	SET domain containing 5	0.144
149066497	Ubr5	ubiquitin protein ligase E3 component n-recognin 5	0.144
564363346	BCL9L	B cell CLL/lymphoma 9 like	0.145
149040413	GPAM	glycerol-3-phosphate acyltransferase, mitochondrial	0.145
198442895	STOX2	storkhead box 2	0.146
58865862	XKR4	XK related 4	0.146
149027694	N/A	N/A	0.148
672055757	N/A	N/A	0.149
404312698	CASC4	cancer susceptibility 4	0.150
56605726	EIF4B	eukaryotic translation initiation factor 4B	0.150
686661085	MTMR4	myotubularin related protein 4	0.152
26023947	NRP1	neuropilin 1	0.152

564359927	RIMS2	regulating synaptic membrane exocytosis 2	0.152
564358968	N/A	N/A	0.152
157817412	ARID1A	AT-rich interaction domain 1A	0.153
728864372	WDFY3	WD repeat and FYVE domain containing 3	0.153
625183267	N/A	N/A	0.155
274327131	GCN1	GCN1, eIF2 alpha kinase activator homolog	0.156
586975096	N/A	N/A	0.157
119582941	ZFAND5	zinc finger AN1-type containing 5	0.158
167555091	CTTNBP2	cortactin binding protein 2	0.159
672053016	LOC691387	similar to HBxAg transactivated protein 2	0.159
197246909	PHC2	polyhomeotic homolog 2	0.159
59709464	PIK3R2	phosphoinositide-3-kinase regulatory subunit 2	0.159
300793740	TANC2	tetratricopeptide repeat, ankyrin repeat and coiled-coil containing 2	0.160
26006223	FBXO21	F-box protein 21	0.161
829969914	N/A	N/A	0.162
4506681	RPS11	ribosomal protein S11	0.163
27545420	TAOK1	TAO kinase 1	0.163
10720132	NEO1	neogenin 1	0.164
672038248	SMG1	SMG1, nonsense mediated mRNA decay associated PI3K related kinase	0.165
672017116	N/A	N/A	0.166
19745186	CREB1	cAMP responsive element binding protein 1	0.167
24418849	KCNB1	potassium voltage-gated channel subfamily B member 1	0.167
145312253	REV3L	REV3 like, DNA directed polymerase zeta catalytic subunit	0.168
564325173	N/A	N/A	0.168
157822563	AREL1	apoptosis resistant E3 ubiquitin protein ligase 1	0.169
564327171	ACTN4	actinin alpha 4	0.170
16758310	LRP3	LDL receptor related protein 3	0.170
157821103	PITPNM2	phosphatidylinositol transfer protein membrane associated 2	0.170
672056431	ZC3H14	zinc finger CCCH-type containing 14	0.170
564396111	ZCCHC14	zinc finger CCHC-type containing 14	0.170
672044124	N/A	N/A	0.170
349501022	2410002F23Rik	RIKEN cDNA 2410002F23 gene	0.171
293346096	FAM171B	family with sequence similarity 171 member B	0.171
568938931	TRRAP	transformation/transcription domain associated protein	0.171

60359854	POLDIP3	DNA polymerase delta interacting protein 3	0.173
148689279	RBM5	RNA binding motif protein 5	0.173
13591904	ADAR	adenosine deaminase, RNA specific	0.174
149054120	ORMDL3	ORMDL sphingolipid biosynthesis regulator 3	0.174
20301990	Podxl	podocalyxin-like	0.175
564353232	PUM1	pumilio RNA binding family member 1	0.175
697993427	TPR	translocated promoter region, nuclear basket protein	0.175
224451084	GPSM1	G protein signaling modulator 1	0.176
408387590	TRIP12	thyroid hormone receptor interactor 12	0.176
392347634	CHD4	chromodomain helicase DNA binding protein 4	0.177
162287391	RPL6	ribosomal protein L6	0.177
672081765	N/A	N/A	0.177
6978787	DYRK1A	dual specificity tyrosine phosphorylation regulated kinase 1A	0.179
23263334	LZTS1	leucine zipper tumor suppressor 1	0.179
795554188	N/A	N/A	0.179
148698133	AHDC1	AT-hook DNA binding motif containing 1	0.180
674066860	N/A	N/A	0.180
189217530	ANAPC2	anaphase promoting complex subunit 2	0.181
157819257	Ubxn7	UBX domain protein 7	0.181
157821015	KDM5B	lysine demethylase 5B	0.182
148706565	N/A	N/A	0.182
6981632	CNTN2	contactin 2	0.183
197384571	UBA2	ubiquitin like modifier activating enzyme 2	0.184
187469679	LDB1	LIM domain binding 1	0.185
149026331	SRSF11	serine and arginine rich splicing factor 11	0.185
148696021	N/A	N/A	0.185
755499467	PPP1R16B	protein phosphatase 1 regulatory subunit 16B	0.186
42627759	SMC3	structural maintenance of chromosomes 3	0.186
60360108	BRD2	bromodomain containing 2	0.188
164663913	HELZ	helicase with zinc finger	0.188
564365520	USP19	ubiquitin specific peptidase 19	0.188
674086663	N/A	N/A	0.189
6981458	RAF1	Raf-1 proto-oncogene, serine/threonine kinase	0.190
203097404	ZNF266	zinc finger protein 266	0.190
672034032	N/A	N/A	0.190
404351649	CDK13	cyclin dependent kinase 13	0.191
109506395	FAM53C	family with sequence similarity 53 member C	0.191
564399456	TSPYL2	TSPY like 2	0.191

672016550	RAPGEF1	Rap guanine nucleotide exchange factor 1	0.192
149049603	ADIPOR2	adiponectin receptor 2	0.194
300797788	DHX15	DEAH-box helicase 15	0.195
21326463	SIPA1L1	signal induced proliferation associated 1 like 1	0.195
157820255	MED13	mediator complex subunit 13	0.196
71043930	Ppip5k2	diphosphoinositol pentakisphosphate kinase 2	0.196
6678315	TSC22D1	TSC22 domain family member 1	0.196
28972113	AVL9	AVL9 cell migration associated	0.197
564389291	PCM1	pericentriolar material 1	0.198
758818575	Peg3	paternally expressed 3	0.198
61556748	TSPYL1	TSPY like 1	0.198
564388185	ERCC6	ERCC excision repair 6, chromatin remodeling factor	0.200
71067095	GON4L	gon-4 like	0.200
213417659	KITLG	KIT ligand	0.200
119616373	MEF2C	myocyte enhancer factor 2C	0.200
564400602	STAG2	stromal antigen 2	0.200
148702008	N/A	N/A	0.200
199561113	ZMYM3	zinc finger MYM-type containing 3	0.201
157819431	BRD3	bromodomain containing 3	0.202
291042494	MED13L	mediator complex subunit 13 like	0.202
521020666	N/A	N/A	0.202
672042964	ASH1L	ASH1 like histone lysine methyltransferase	0.203
157820711	Foxn3	forkhead box N3	0.203
157819149	CUL4B	cullin 4B	0.204
564321656	TCF25	transcription factor 25	0.204
29789299	XPO1	exportin 1	0.204
13928696	JAK2	Janus kinase 2	0.206
425384	CAMK4	calcium/calmodulin dependent protein kinase IV	0.207
157824032	Ptprt	protein tyrosine phosphatase, receptor type, T	0.208
157821923	SLCO5A1	solute carrier organic anion transporter family member 5A1	0.209
157822303	GPR107	G protein-coupled receptor 107	0.212
208022685	IARS	isoleucyl-tRNA synthetase	0.212
189491614	SLC25A46	solute carrier family 25 member 46	0.212
9910320	TENM2	teneurin transmembrane protein 2	0.212
30061483	HAP1	huntingtin associated protein 1	0.213
6981264	NF1	neurofibromin 1	0.213
19705483	CLSTN2	calsyntenin 2	0.214

169259769	ZNF292	zinc finger protein 292	0.214
171846592	GPBP1	GC-rich promoter binding protein 1	0.215
62088168	ELAVL2	ELAV like RNA binding protein 2	0.216
189163477	SCAF4	SR-related CTD associated factor 4	0.216
564367958	SEMA4C	semaphorin 4C	0.216
831218355	N/A	N/A	0.216
157819275	SMURF1	SMAD specific E3 ubiquitin protein ligase 1	0.217
564347477	ZNF638	zinc finger protein 638	0.217
149069422	RPL7L1	ribosomal protein L7 like 1	0.218
564386027	CHD8	chromodomain helicase DNA binding protein 8	0.219
149031601	Hist1h1c	histone cluster 1 H1 family member c	0.219
2804296	CDH8	cadherin 8	0.221
564298823	EML3	echinoderm microtubule associated protein like 3	0.221
148680747	ANKFY1	ankyrin repeat and FYVE domain containing 1	0.222
928135679	GAN	gigaxonin	0.222
114145788	NAA25	N(alpha)-acetyltransferase 25, NatB auxiliary subunit	0.222
30024612	SOX11	SRY-box 11	0.222
564361244	TCF20	transcription factor 20	0.222
149054141	CASC3	cancer susceptibility 3	0.223
157818273	CDC42EP4	CDC42 effector protein 4	0.223
148702333	DDX42	DEAD-box helicase 42	0.223
392333209	DLG5	discs large MAGUK scaffold protein 5	0.223
724804431	N/A	N/A	0.223
672076339	RBPJ	recombination signal binding protein for immunoglobulin kappa J region	0.224
281371443	CASTOR2	cytosolic arginine sensor for mTORC1 subunit 2	0.225
148491097	DYNC1H1	dynein cytoplasmic 1 heavy chain 1	0.225
13928850	PHGDH	phosphoglycerate dehydrogenase	0.225
157819757	RNF182	ring finger protein 182	0.225
62087776	SYNCRIP	synaptotagmin binding cytoplasmic RNA interacting protein	0.225
823419445	N/A	N/A	0.225
529367218	Abca8a	ATP-binding cassette, sub-family A (ABC1), member 8a	0.226
13540699	NRP2	neuropilin 2	0.226
6981636	TCF12	transcription factor 12	0.226
112984440	TNFRSF19	TNF receptor superfamily member 19	0.226

76096354	ARFGAP2	ADP ribosylation factor GTPase activating protein 2	0.227
564344160	CHD6	chromodomain helicase DNA binding protein 6	0.227
157819605	EPC2	enhancer of polycomb homolog 2	0.227
403310688	USP24	ubiquitin specific peptidase 24	0.227
392348740	LAMB1	laminin subunit beta 1	0.228
403420604	PCDH11X	protocadherin 11 X-linked	0.228
672072009	Zfp68	zinc finger protein 68	0.228
731286412	N/A	N/A	0.229
440909886	N/A	N/A	0.229
17864836	CACNA1C	calcium voltage-gated channel subunit alpha1 C	0.230
157817961	PHF3	PHD finger protein 3	0.230
11177894	TSC1	TSC complex subunit 1	0.230
672087260	N/A	N/A	0.230
145553966	CACNA1E	calcium voltage-gated channel subunit alpha1 E	0.231
392351663	GPATCH8	G-patch domain containing 8	0.231
157818545	ZNF316	zinc finger protein 316	0.231
149031313	N/A	N/A	0.231
157817873	ANKRD12	ankyrin repeat domain 12	0.232
61557491	TMEM263	transmembrane protein 263	0.232
913500911	N/A	N/A	0.232
672084956	N/A	N/A	0.232
157821429	BAZ2A	bromodomain adjacent to zinc finger domain 2A	0.233
564353949	N/A	N/A	0.233
564381925	ACKR1	atypical chemokine receptor 1 (Duffy blood group)	0.234
19923674	PAM	peptidylglycine alpha-amidating monooxygenase	0.234
564383487	SLAIN2	SLAIN motif family member 2	0.234
641717602	N/A	N/A	0.234
68163459	JOSD1	Josephin domain containing 1	0.235
149016025	N/A	N/A	0.235
213972547	KAT6A	lysine acetyltransferase 6A	0.236
293341411	Rc3h1	ring finger and CCCH-type domains 1	0.237
2266994	OGT	O-linked N-acetylglucosamine (GlcNAc) transferase	0.238
58865776	TRIM32	tripartite motif containing 32	0.238
14091754	GRIP1	glutamate receptor interacting protein 1	0.239
297681940	N/A	N/A	0.239

564334521	CACUL1	CDK2 associated cullin domain 1	0.240
37360004	KDM1A	lysine demethylase 1A	0.240
62656582	KIAA0100	KIAA0100	0.240
537233715	N/A	N/A	0.240
456367248	ARFGEF1	ADP ribosylation factor guanine nucleotide exchange factor 1	0.241
672071650	N/A	N/A	0.241
403310691	RAI1	retinoic acid induced 1	0.242
62087532	SRSF6	serine and arginine rich splicing factor 6	0.242
148697254	TBL1X	transducin beta like 1 X-linked	0.243
300797458	UBE3A	ubiquitin protein ligase E3A	0.243
187282338	Zfp955a/Zfp955b	zinc finger protein 955B	0.243
859770113	N/A	N/A	0.244
966923099	SMG7	SMG7, nonsense mediated mRNA decay factor	0.245
62078579	NUB1	negative regulator of ubiquitin like proteins 1	0.247
149067682	HSD3B7	hydroxy-delta-5-steroid dehydrogenase, 3 beta- and steroid delta-isomerase 7	0.248
403310660	LATS1	large tumor suppressor kinase 1	0.248
672022994	NEO1	neogenin 1	0.248
149034059	CDH4	cadherin 4	0.249
37360236	SMG5	SMG5, nonsense mediated mRNA decay factor	0.249
157823639	PPP1R13B	protein phosphatase 1 regulatory subunit 13B	0.250
167234435	EML4	echinoderm microtubule associated protein like 4	0.251
157820119	LRRTM1	leucine rich repeat transmembrane neuronal 1	0.251
80751171	PCDHGA10	protocadherin gamma subfamily A, 10	0.251
674077166	N/A	N/A	0.251
61097926	RYK	receptor-like tyrosine kinase	0.252
564329093	TM6SF1	transmembrane 6 superfamily member 1	0.252
149058465	ZBTB41	zinc finger and BTB domain containing 41	0.252
12831217	GPR27	G protein-coupled receptor 27	0.253
293343541	ICE1	interactor of little elongation complex ELL subunit 1	0.253
148671090	LSM14A	LSM14A, mRNA processing body assembly factor	0.253
537151504	N/A	N/A	0.253
396080328	ADCYAP1R1	ADCYAP receptor type I	0.255
155369656	AQR	aquarius intron-binding spliceosomal factor	0.255
290560930	CREBBP	CREB binding protein	0.255
564383236	YTHDC1	YTH domain containing 1	0.255

672035849	N/A	N/A	0.255
157822191	MTMR2	myotubularin related protein 2	0.256
67078454	SLC25A51	solute carrier family 25 member 51	0.256
71043896	FEM1A	fem-1 homolog A	0.257
57527570	LRRC41	leucine rich repeat containing 41	0.257
157818967	KDM6B	lysine demethylase 6B	0.258
672026416	PRR36	proline rich 36	0.258
166157456	PPP4R2	protein phosphatase 4 regulatory subunit 2	0.259
431911661	N/A	N/A	0.259
564316243	CEP170	centrosomal protein 170	0.261
564333160	TMEM2	transmembrane protein 2	0.261
545215595	N/A	N/A	0.262
672040416	N/A	N/A	0.262
403310664	KMT2E	lysine methyltransferase 2E	0.263
149065851	XPNPEP3	X-prolyl aminopeptidase 3	0.263
926691741	N/A	N/A	0.263
537216032	N/A	N/A	0.263
71361653	STRN3	striatin 3	0.264
157820313	GIGYF1	GRB10 interacting GYF protein 1	0.265
564315812	NAV1	neuron navigator 1	0.265
293340917	C3orf70	chromosome 3 open reading frame 70	0.266
51948482	DEK	DEK proto-oncogene	0.266
12018256	ECH1	enoyl-CoA hydratase 1	0.266
625182285	N/A	N/A	0.266
154426327	KANSL2	KAT8 regulatory NSL complex subunit 2	0.267
48040531	RNF114	ring finger protein 114	0.267
537273922	N/A	N/A	0.267
300796412	ATMIN	ATM interactor	0.268
392332910	TP53BP2	tumor protein p53 binding protein 2	0.268
84781684	ZNF207	zinc finger protein 207	0.268
464391331	KDM5A	lysine demethylase 5A	0.269
164565360	CTTNBP2NL	CTTNBP2 N-terminal like	0.271
66911118	NFX1	nuclear transcription factor, X-box binding 1	0.271
6678349	TIAL1	TIA1 cytotoxic granule associated RNA binding protein like 1	0.271
672055562	LBH	limb bud and heart development	0.272
564350633	SLC35A1	solute carrier family 35 member A1	0.272
568977804	ATAD2B	ATPase family, AAA domain containing 2B	0.273
348605217	SENP6	SUMO1/sentrin specific peptidase 6	0.273
4885579	CNOT9	CCR4-NOT transcription complex subunit 9	0.274

9506755	GRIK2	glutamate ionotropic receptor kainate type subunit 2	0.274
157820491	SESN1	sestrin 1	0.274
392337823	RSF1	remodeling and spacing factor 1	0.275
564368081	REV1	REV1, DNA directed polymerase	0.276
748983435	VEZF1	vascular endothelial zinc finger 1	0.276
300796674	TRANK1	tetratricopeptide repeat and ankyrin repeat containing 1	0.277
300797562	BCOR	BCL6 corepressor	0.278
62078991	Rsrc2	arginine/serine-rich coiled-coil 2	0.278
149032040	SLC11A2	solute carrier family 11 member 2	0.278
27465559	BRINP2	BMP/retinoic acid inducible neural specific 2	0.279
157820199	ZNF358	zinc finger protein 358	0.279
40254779	EFNB1	ephrin B1	0.280
568930638	HSPG2	heparan sulfate proteoglycan 2	0.280
281371328	PTPN4	protein tyrosine phosphatase, non-receptor type 4	0.280
50511177	SLITRK1	SLIT and NTRK like family member 1	0.281
300793780	ZNF251	zinc finger protein 251	0.281
300797978	FLNC	filamin C	0.282
309951113	USPL1	ubiquitin specific peptidase like 1	0.282
672022657	N/A	N/A	0.282
11067415	ERBB4	erb-b2 receptor tyrosine kinase 4	0.283
28972858	VCPIP1	valosin containing protein interacting protein 1	0.283
34877176	GPRIN2	G protein regulated inducer of neurite outgrowth 2	0.284
955485868	N/A	N/A	0.285
157819499	ANKRD17	ankyrin repeat domain 17	0.286
564353880	DDI2	DNA damage inducible 1 homolog 2	0.286
148673748	FAM110B	family with sequence similarity 110 member B	0.286
300794761	Fat4	FAT atypical cadherin 4	0.286
52138617	YME1L1	YME1 like 1 ATPase	0.286
40018556	NOB1	NIN1/PSMD8 binding protein 1 homolog	0.287
672053718	N/A	N/A	0.287
19924085	FAT3	FAT atypical cadherin 3	0.288
56090389	INTS14	integrator complex subunit 14	0.288
672045624	MARCH7	membrane associated ring-CH-type finger 7	0.288
149048628	MYNN	myoneurin	0.288
13928966	HSF2	heat shock transcription factor 2	0.289
164565364	ITPKB	inositol-trisphosphate 3-kinase B	0.289

157821413	USP30	ubiquitin specific peptidase 30	0.289
13928842	ZNF148	zinc finger protein 148	0.289
827475660	EPC1	enhancer of polycomb homolog 1	0.290
300794743	TSC22D2	TSC22 domain family member 2	0.290
564333920	PPRC1	peroxisome proliferator-activated receptor gamma, coactivator-related 1	0.292
564364877	TBC1D2B	TBC1 domain family member 2B	0.292
672051957	N/A	N/A	0.292
68534262	C1orf43	chromosome 1 open reading frame 43	0.293
29789319	CBLB	Cbl proto-oncogene B	0.293
162951835	CYTH1	cytohesin 1	0.293
157819315	OSBPL11	oxysterol binding protein like 11	0.293
149060466	ZBTB20	zinc finger and BTB domain containing 20	0.293
584052040	N/A	N/A	0.293
149065426	CASP2	caspase 2	0.294
568992461	DIP2B	disco interacting protein 2 homolog B	0.294
568992461	DIP2B	disco interacting protein 2 homolog B	0.294
672073809	N/A	N/A	0.296
635017744	N/A	N/A	0.296
672046069	SP3	Sp3 transcription factor	0.297
569005738	KDM2A	lysine demethylase 2A	0.298
149045074	NUP153	nucleoporin 153	0.298
58865780	ZBTB17	zinc finger and BTB domain containing 17	0.300
148699086	ZDHHC21	zinc finger DHHC-type containing 21	0.300
149067383	PRDM4	PR/SET domain 4	0.301
300797915	Rbm33	RNA binding motif protein 33	0.301
564344738	N/A	N/A	0.301
156627555	NT5C3B	5'-nucleotidase, cytosolic IIIB	0.302
672052418	N/A	N/A	0.302
827475647	OSBPL8	oxysterol binding protein like 8	0.303
564382848	Hnrnpdl	heterogeneous nuclear ribonucleoprotein D-like	0.304
672043577	Rprd2	regulation of nuclear pre-mRNA domain containing 2	0.304
149053570	WSB1	WD repeat and SOCS box containing 1	0.304
124249254	ZNF639	zinc finger protein 639	0.304
109490297	ABCA3	ATP binding cassette subfamily A member 3	0.305
13929168	FAT1	FAT atypical cadherin 1	0.305
672082027	N/A	N/A	0.305
564364873	ADAMTS7	ADAM metallopeptidase with thrombospondin type 1 motif 7	0.306

6978755	DCC	DCC netrin 1 receptor	0.306
755548135	SPATA13	spermatogenesis associated 13	0.306
149024675	N/A	N/A	0.307
157818041	YEATS2	YEATS domain containing 2	0.309
404351667	BOD1	biorientation of chromosomes in cell division 1	0.310
392340053	FRMD4B	FERM domain containing 4B	0.310
755566493	LRCH2	leucine rich repeats and calponin homology domain containing 2	0.310
171916115	LRRC55	leucine rich repeat containing 55	0.310
264681499	Ddx3	DEAD (Asp-Glu-Ala-Asp) box polypeptide 3	0.311
270483881	CBFA2T2	CBFA2/RUNX1 translocation partner 2	0.312
392333169	CCDC88A	coiled-coil domain containing 88A	0.313
157822027	CSRNP2	cysteine and serine rich nuclear protein 2	0.313
157819449	ZBTB11	zinc finger and BTB domain containing 11	0.313
564353506	ZNF436	zinc finger protein 436	0.313
859858910	N/A	N/A	0.313
1334149	N/A	N/A	0.314
13786132	Acot1	acyl-CoA thioesterase 1	0.316
50510655	PCF11	PCF11 cleavage and polyadenylation factor subunit	0.316
672016634	GAPVD1	GTPase activating protein and VPS9 domains 1	0.317
14091779	Chn2	chimerin 2	0.318
61557316	ST3GAL1	ST3 beta-galactoside alpha-2,3-sialyltransferase 1	0.318
157819279	TNPO3	transportin 3	0.318
149023886	N/A	N/A	0.318
157818691	YTHDF3	YTH N6-methyladenosine RNA binding protein 3	0.319
293341722	N/A	N/A	0.319
149067028	Ppp1r12a	protein phosphatase 1, regulatory subunit 12A	0.320
149031125	APMAP	adipocyte plasma membrane associated protein	0.321
148681067	VASH2	vasohibin 2	0.321
344249173	N/A	N/A	0.321
564335225	MIER3	MIER family member 3	0.323
30842813	SLC38A2	solute carrier family 38 member 2	0.323
564298767	N/A	N/A	0.323
157821303	PPP4R3B	protein phosphatase 4 regulatory subunit 3B	0.324
157823419	PRAG1	PEAK1 related, kinase-activating pseudokinase 1	0.324
672035060	CIC	capicua transcriptional repressor	0.325

564371471	CLINT1	clathrin interactor 1	0.325
672078533	N/A	N/A	0.325
958729596	N/A	N/A	0.325
672039306	RCOR2	REST corepressor 2	0.326
672072366	N/A	N/A	0.326
564370219	LPIN2	lipin 2	0.327
149067244	SCYL2	SCY1 like pseudokinase 2	0.329
50866	SMARCAD1	SWI/SNF-related, matrix-associated actin-dependent regulator of chromatin, subfamily a, containing DEAD/H box 1	0.329
40018600	CNPPD1	cyclin Pas1/PHO80 domain containing 1	0.330
149021415	SNX25	sorting nexin 25	0.330
344239802	N/A	N/A	0.330
149050659	BIRC6	baculoviral IAP repeat containing 6	0.331
512983752	N/A	N/A	0.332
564330111	NUP98	nucleoporin 98	0.333
672052120	RBM12B	RNA binding motif protein 12B	0.333
157817720	SLC16A14	solute carrier family 16 member 14	0.333
157821187	SLITRK2	SLIT and NTRK like family member 2	0.333
71051128	ANKRD10	ankyrin repeat domain 10	0.334
293348129	DACT1	dishevelled binding antagonist of beta catenin 1	0.334
902763351	LARP4B	La ribonucleoprotein domain family member 4B	0.335
3676248	Prim1	DNA primase subunit 1	0.335
564303143	KMT2C	lysine methyltransferase 2C	0.336
564377460	N/A	N/A	0.336
29612542	H2AFZ	H2A histone family member Z	0.337
511905488	N/A	N/A	0.337
148703547	CNOT7	CCR4-NOT transcription complex subunit 7	0.339
157819969	CTU1	cytosolic thiouridylase subunit 1	0.339
51871603	ST7	suppression of tumorigenicity 7	0.339
564303575	DENND2A	DENN domain containing 2A	0.340
157818529	SLITRK3	SLIT and NTRK like family member 3	0.340
149024496	SPEN	spen family transcriptional repressor	0.341
564334870	Ap3b1	adaptor-related protein complex 3, beta 1 subunit	0.342
198041681	LTN1	listerin E3 ubiquitin protein ligase 1	0.342
62244083	PDRG1	p53 and DNA damage regulated 1	0.342
60360568	GRIA3	glutamate ionotropic receptor AMPA type subunit 3	0.343

564310551	N/A	N/A	0.343
198041672	TNRC6B	trinucleotide repeat containing 6B	0.345
32451765	FBXO10	F-box protein 10	0.346
512957927	N/A	N/A	0.346
76362828	TEF	TEF, PAR bZIP transcription factor	0.349
148687368	SH2B2	SH2B adaptor protein 2	0.351
300796732	ZNF445	zinc finger protein 445	0.351
47155567	ARHGAP20	Rho GTPase activating protein 20	0.352
164518906	COX15	COX15, cytochrome c oxidase assembly homolog	0.352
564313637	CDR2L	cerebellar degeneration related protein 2 like	0.353
880888432	N/A	N/A	0.353
164698411	CTDSP2	CTD small phosphatase 2	0.354
28076889	YIPF4	Yip1 domain family member 4	0.355
148694162	CSNK1G1	casein kinase 1 gamma 1	0.356
564303058	DIDO1	death inducer-obliterator 1	0.356
157818913	OTUD7B	OTU deubiquitinase 7B	0.356
149027091	GABRA3	gamma-aminobutyric acid type A receptor alpha3 subunit	0.357
315259095	UBN1	ubinuclein 1	0.357
672052399	N/A	N/A	0.360
672084304	N/A	N/A	0.362
672083055	AMMECR1L	AMMECR1 like	0.363
392348187	LRP8	LDL receptor related protein 8	0.363
109467304	TRIM33	tripartite motif containing 33	0.363
564310645	PLXNB1	plexin B1	0.364
998455137	RGS10	regulator of G protein signaling 10	0.364
157823223	ZNF330	zinc finger protein 330	0.365
157820963	INTS5	integrator complex subunit 5	0.366
672031398	ANKRD11	ankyrin repeat domain 11	0.367
37360278	CNOT6	CCR4-NOT transcription complex subunit 6	0.368
57528225	UTP4	UTP4, small subunit processome component	0.369
674076364	N/A	N/A	0.369
564328896	CHD2	chromodomain helicase DNA binding protein 2	0.370
157817286	EZH1	enhancer of zeste 1 polycomb repressive complex 2 subunit	0.370
157823615	KBTBD11	kelch repeat and BTB domain containing 11	0.371
564299019	KIAA2026	KIAA2026	0.371
157818387	DHX36	DEAH-box helicase 36	0.372
672061705	KMT2A	lysine methyltransferase 2A	0.372

564342632	TP53BP1	tumor protein p53 binding protein 1	0.372
564361788	COL2A1	collagen type II alpha 1 chain	0.373
157820557	EDRF1	erythroid differentiation regulatory factor 1	0.374
157817845	KMT5B	lysine methyltransferase 5B	0.374
564351113	N/A	N/A	0.374
392334198	SEH1L	SEH1 like nucleoporin	0.375
157819301	ZNF777	zinc finger protein 777	0.375
199561799	LRP12	LDL receptor related protein 12	0.377
672037929	TNRC6A	trinucleotide repeat containing 6A	0.377
148690303	ZNF205	zinc finger protein 205	0.377
564363988	ISLR2	immunoglobulin superfamily containing leucine rich repeat 2	0.378
37360474	MIER1	MIER1 transcriptional regulator	0.379
149030718	PIP5K1A	phosphatidylinositol-4-phosphate 5-kinase type 1 alpha	0.379
300794867	RSBN1	round spermatid basic protein 1	0.382
56090383	TMEM43	transmembrane protein 43	0.382
121583782	ZNF426	zinc finger protein 426	0.382
109464982	TMEM131L	transmembrane 131 like	0.383
564367862	Dst	dystonin	0.386
148675659	CSDE1	cold shock domain containing E1	0.388
149047445	FGFR3	fibroblast growth factor receptor 3	0.388
149062169	MEN1	menin 1	0.390
149063273	MPHOSPH9	M-phase phosphoprotein 9	0.390
149015884	ZNF407	zinc finger protein 407	0.390
672069572	KANSL1	KAT8 regulatory NSL complex subunit 1	0.391
564333605	BTAF1	B-TFIID TATA-box binding protein associated factor 1	0.392
582015198	CRY2	cryptochrome circadian regulator 2	0.392
672036088	KMT2B	lysine methyltransferase 2B	0.392
157819851	ZNF565	zinc finger protein 565	0.392
961745338	N/A	N/A	0.392
13928816	EIF2AK3	eukaryotic translation initiation factor 2 alpha kinase 3	0.394
672082323	N/A	N/A	0.394
149054498	CDC27	cell division cycle 27	0.395
61557304	SNAPC3	small nuclear RNA activating complex polypeptide 3	0.397
109470195	TNKS1BP1	tankyrase 1 binding protein 1	0.397
564320608	SEMA6A	semaphorin 6A	0.398

40018598	ANGPTL4	angiopoietin like 4	0.399
577019520	CLOCK	clock circadian regulator	0.399
149064227	DMXL1	Dmx like 1	0.399
244792650	TNIK	TRAF2 and NCK interacting kinase	0.399
127139124	ZNF597	zinc finger protein 597	0.399
51948532	TBC1D20	TBC1 domain family member 20	0.400
158138517	FGFR2	fibroblast growth factor receptor 2	0.401
755532277	Lrrfip2	leucine rich repeat (in FLII) interacting protein 2	0.403
157820043	ZKSCAN5	zinc finger with KRAB and SCAN domains 5	0.403
148687487	N/A	N/A	0.403
14388593	SPATA2	spermatogenesis associated 2	0.404
157819125	PHF13	PHD finger protein 13	0.405
564377500	N/A	N/A	0.405
672057084	N/A	N/A	0.405
383087760	PKD1	polycystin 1, transient receptor potential channel interacting	0.406
149024245	RSRP1	arginine and serine rich protein 1	0.406
672014912	SORBS1	sorbin and SH3 domain containing 1	0.406
672033285	N/A	N/A	0.406
61557263	GDAP2	ganglioside induced differentiation associated protein 2	0.407
157787155	NDST2	N-deacetylase and N-sulfotransferase 2	0.407
149016466	NIPBL	NIPBL, cohesin loading factor	0.407
33086606	SRPRB	SRP receptor beta subunit	0.407
960953448	N/A	N/A	0.408
392339261	PKP4	plakophilin 4	0.410
281599331	ZKSCAN8	zinc finger with KRAB and SCAN domains 8	0.410
224451093	ZNF317	zinc finger protein 317	0.410
148683901	MSI2	musashi RNA binding protein 2	0.413
564317925	SACS	sacsin molecular chaperone	0.415
57527375	THUMPD1	THUMP domain containing 1	0.415
300797262	BRPF1	bromodomain and PHD finger containing 1	0.416
762005986	N4BP1	NEDD4 binding protein 1	0.416
157822073	PAPOLG	poly(A) polymerase gamma	0.417
724851156	N/A	N/A	0.418
40789237	PCDHA4	protocadherin alpha 4	0.419
67846052	DCUN1D3	defective in cullin neddylation 1 domain containing 3	0.420
293346811	MINDY4	MINDY lysine 48 deubiquitinase 4	0.422

564316962	N/A	N/A	0.424
564382837	LIN54	lin-54 DREAM MuvB core complex component	0.425
281599335	BMS1	BMS1, ribosome biogenesis factor	0.426
204744	IGFBP3	insulin like growth factor binding protein 3	0.426
694864049	CEP295	centrosomal protein 295	0.427
392353586	INTS6	integrator complex subunit 6	0.427
281353901	N/A	N/A	0.427
676284727	N/A	N/A	0.427
62543511	RAB30	RAB30, member RAS oncogene family	0.428
157819811	C21orf91	chromosome 21 open reading frame 91	0.432
672063138	IP6K2	inositol hexakisphosphate kinase 2	0.432
201025402	PCDH18	protocadherin 18	0.432
564355945	HECTD1	HECT domain E3 ubiquitin protein ligase 1	0.433
149023254	POLR1B	RNA polymerase I subunit B	0.433
26335263	GDF10	growth differentiation factor 10	0.435
17530969	SLC8A3	solute carrier family 8 member A3	0.436
189181698	ZNF131	zinc finger protein 131	0.437
52345439	NXPH4	neurexophilin 4	0.438
913491397	N/A	N/A	0.438
56090325	TMEM206	transmembrane protein 206	0.442
564311452	TMEM131	transmembrane protein 131	0.443
568940286	BRAF	B-Raf proto-oncogene, serine/threonine kinase	0.444
76559921	CRTC2	CREB regulated transcription coactivator 2	0.444
157819941	PEX26	peroxisomal biogenesis factor 26	0.444
672057962	N/A	N/A	0.445
564344754	PMEPA1	prostate transmembrane protein, androgen induced 1	0.446
971825925	DDHD2	DDHD domain containing 2	0.447
148686948	RPS6KA5	ribosomal protein S6 kinase A5	0.447
564352690	TRIT1	tRNA isopentenyltransferase 1	0.447
635141277	N/A	N/A	0.447
667261609	N/A	N/A	0.447
74183022	Zfp773	zinc finger protein 773	0.448
148693035	CEP57	centrosomal protein 57	0.449
564395313	OTUD4	OTU deubiquitinase 4	0.449
194474032	RNF19A	ring finger protein 19A, RBR E3 ubiquitin protein ligase	0.449
148705863	GABRA4	gamma-aminobutyric acid type A receptor alpha4 subunit	0.450

672043253	DENND4B	DENN domain containing 4B	0.451
880805457	GPR161	G protein-coupled receptor 161	0.451
109484871	HERC1	HECT and RLD domain containing E3 ubiquitin protein ligase family member 1	0.451
37360132	RHOBTB3	Rho related BTB domain containing 3	0.452
148683687	RHBDL3	rhomboid like 3	0.453
568973498	TNRC6C	trinucleotide repeat containing 6C	0.454
74145569	BNIP2	BCL2 interacting protein 2	0.456
59709429	ZSCAN21	zinc finger and SCAN domain containing 21	0.456
672051965	NSMAF	neutral sphingomyelinase activation associated factor	0.459
112984092	RPRM	reprimo, TP53 dependent G2 arrest mediator homolog	0.460
31543579	RELN	reelin	0.461
672035062	N/A	N/A	0.461
19173786	SYF2	SYF2 pre-mRNA splicing factor	0.463
157822011	TGS1	trimethylguanosine synthase 1	0.463
564354018	DFFA	DNA fragmentation factor subunit alpha	0.465
39930812	AKR7A2	aldo-keto reductase family 7 member A2	0.467
564316247	CEP170	centrosomal protein 170	0.467
149066961	TBC1D15	TBC1 domain family member 15	0.467
109464919	ARHGEF26	Rho guanine nucleotide exchange factor 26	0.468
532045909	N/A	N/A	0.468
300797934	Ranbp2	RAN binding protein 2	0.469
149048326	N/A	N/A	0.469
537134105	N/A	N/A	0.469
564332832	INCENP	inner centromere protein	0.470
392355241	UHRF1BP1	UHRF1 binding protein 1	0.470
157818267	CNEP1R1	CTD nuclear envelope phosphatase 1 regulatory subunit 1	0.471
149025439	DICER1	dicer 1, ribonuclease III	0.472
392334341	USP38	ubiquitin specific peptidase 38	0.472
403224961	TRPM7	transient receptor potential cation channel subfamily M member 7	0.473
58865998	PCDHGB7	protocadherin gamma subfamily B, 7	0.476
6981430	PTGDS	prostaglandin D2 synthase	0.476
568997192	PRDM15	PR/SET domain 15	0.477
213512587	RNF31	ring finger protein 31	0.477
50510855	RIMKLB	ribosomal modification protein rimK like family member B	0.478

672065395	CCNYL1	cyclin Y like 1	0.479
157821129	UHRF2	ubiquitin like with PHD and ring finger domains 2	0.479
672014312	ZNF646	zinc finger protein 646	0.479
219273429	A430033K04Rik	RIKEN cDNA A430033K04 gene	0.481
564318679	CCSER2	coiled-coil serine rich protein 2	0.482
149059823	N/A	N/A	0.482
66730382	TRNT1	tRNA nucleotidyl transferase 1	0.483
931568101	N/A	N/A	0.483
568972622	BPTF	bromodomain PHD finger transcription factor	0.484
537271325	N/A	N/A	0.485
672042931	GATAD2B	GATA zinc finger domain containing 2B	0.486
109460021	KIAA2026	KIAA2026	0.489
564297823	KIF7	kinesin family member 7	0.489
803269187	N/A	N/A	0.489
56090445	PHOSPHO2	phosphatase, orphan 2	0.490
537148165	N/A	N/A	0.490
149064065	ZMYM5	zinc finger MYM-type containing 5	0.495
672017463	N/A	N/A	0.496
564390712	NOL8	nucleolar protein 8	0.498
157822711	RBM28	RNA binding motif protein 28	0.498
672023887	N/A	N/A	0.498
564297338	ZNF816	zinc finger protein 816	0.500
62651891	WASHC4	WASH complex subunit 4	0.502
149016574	ZNF324	zinc finger protein 324	0.503
149041559	BUD13	BUD13 homolog	0.506
149067702	ZNF629	zinc finger protein 629	0.506
160333172	COG2	component of oligomeric golgi complex 2	0.507
300797651	FOXO1	forkhead box O1	0.508
148681991	MIOS	meiosis regulator for oocyte development	0.508
71043716	FBXO46	F-box protein 46	0.509
149274619	ZFHX2	zinc finger homeobox 2	0.510
61889068	MXI1	MAX interactor 1, dimerization protein	0.512
672020901	N/A	N/A	0.512
564393142	WDR36	WD repeat domain 36	0.513
149025439	DICER1	dicer 1, ribonuclease III	0.514
281371441	LOC100910540/Mepce	methylphosphate capping enzyme	0.515
198278557	NRF1	nuclear respiratory factor 1	0.515
66730445	LZTFL1	leucine zipper transcription factor like 1	0.518

114145794	ANKLE2	ankyrin repeat and LEM domain containing 2	0.519
39104628	SORBS1	sorbin and SH3 domain containing 1	0.521
672088045	N/A	N/A	0.521
564301979	CKAP5	cytoskeleton associated protein 5	0.526
926717435	N/A	N/A	0.529
564352121	LRP8	LDL receptor related protein 8	0.530
635121365	N/A	N/A	0.530
564310412	DOPEY1	dopey family member 1	0.535
815891112	AHR	aryl hydrocarbon receptor	0.536
672027688	N/A	N/A	0.537
149020413	Zfp599	zinc finger protein 599	0.538
564323252	KLHL13	kelch like family member 13	0.539
257196174	PDZRN4	PDZ domain containing ring finger 4	0.539
157821859	RNF219	ring finger protein 219	0.542
148710252	SLITRK4	SLIT and NTRK like family member 4	0.542
564309026	SUN2	Sad1 and UNC84 domain containing 2	0.543
148704240	ZMYM2	zinc finger MYM-type containing 2	0.543
755516639	N/A	N/A	0.543
157819299	FAM167A	family with sequence similarity 167 member A	0.544
157824010	MRPS31	mitochondrial ribosomal protein S31	0.544
198278471	ZBED4	zinc finger BED-type containing 4	0.547
402692201	EML5	echinoderm microtubule associated protein like 5	0.548
149066158	ZNF623	zinc finger protein 623	0.549
672022661	N/A	N/A	0.553
16258817	ATP7A	ATPase copper transporting alpha	0.554
672087657	N/A	N/A	0.554
149063010	DTX2	deltex E3 ubiquitin ligase 2	0.556
148682691	CLDN12	claudin 12	0.558
564329414	IKBKG	inhibitor of nuclear factor kappa B kinase subunit gamma	0.558
672087275	KDM5C	lysine demethylase 5C	0.558
157820309	ZNF592	zinc finger protein 592	0.558
635017744	N/A	N/A	0.558
293349725	AMER3	APC membrane recruitment protein 3	0.560
215490074	SAP18	Sin3A associated protein 18	0.561
62078501	TTI2	TELO2 interacting protein 2	0.561
209364558	CEP290	centrosomal protein 290	0.563
149044768	VPS54	VPS54, GARP complex subunit	0.563

149048116	KHDC4	KH domain containing 4, pre-mRNA splicing factor	0.565
145553978	SFMBT1	Scm like with four mbt domains 1	0.566
564325866	ZNF274	zinc finger protein 274	0.568
51948522	PLA2G15	phospholipase A2 group XV	0.570
77917610	GPBP1L1	GC-rich promoter binding protein 1 like 1	0.571
213688370	EXOSC7	exosome component 7	0.575
564341299	N/A	N/A	0.576
256000825	FAM227A	family with sequence similarity 227 member A	0.577
564363988	ISLR2	immunoglobulin superfamily containing leucine rich repeat 2	0.577
344250492	N/A	N/A	0.578
884934330	N/A	N/A	0.579
564367862	Dst	dystonin	0.580
564311697	PIKFYVE	phosphoinositide kinase, FYVE-type zinc finger containing	0.589
672066092	UNC80	unc-80 homolog, NALCN channel complex subunit	0.589
6981680	TSHR	thyroid stimulating hormone receptor	0.591
392337836	RNF169	ring finger protein 169	0.596
241666396	CLK1	CDC like kinase 1	0.597
13928942	PER2	period circadian regulator 2	0.600
672063745	SEC22C	SEC22 homolog C, vesicle trafficking protein	0.600
56605820	PAPD4	poly(A) RNA polymerase D4, non-canonical	0.601
564299234	Lcor	ligand dependent nuclear receptor corepressor	0.607
731286412	N/A	N/A	0.608
62078947	MOSPD1	motile sperm domain containing 1	0.609
392354293	Hmgb3	high mobility group box 3	0.611
404351643	HMCN1	hemicentin 1	0.618
392352051	N/A	N/A	0.619
74186677	SIN3B	SIN3 transcription regulator family member B	0.622
157817797	PDCD2L	programmed cell death 2 like	0.623
672039849	STX3	syntaxin 3	0.632
55741772	SDAD1	SDA1 domain containing 1	0.634
564305530	PTPRD	protein tyrosine phosphatase, receptor type D	0.635
293339965	RAB11FIP3	RAB11 family interacting protein 3	0.640
845633640	TSSC4	tumor suppressing subtransferable candidate 4	0.640
16758666	TIMP1	TIMP metallopeptidase inhibitor 1	0.642
564395350	N/A	N/A	0.644
21703842	RTCB	RNA 2',3'-cyclic phosphate and 5'-OH ligase	0.646

28972363	DOCK4	dedicator of cytokinesis 4	0.647
157820477	ZFYVE26	zinc finger FYVE-type containing 26	0.647
149041904	GLCE	glucuronic acid epimerase	0.648
149052692	N/A	N/A	0.648
157819993	CCDC112	coiled-coil domain containing 112	0.649
34872960	SMG8	SMG8, nonsense mediated mRNA decay factor	0.650
149042018	N/A	N/A	0.650
157817885	MED17	mediator complex subunit 17	0.652
188595675	RFX7	regulatory factor X7	0.654
564301893	CWC22	CWC22 spliceosome associated protein homolog	0.655
157822681	EFNB2	ephrin B2	0.658
149066285	PHF20L1	PHD finger protein 20 like 1	0.659
537191098	N/A	N/A	0.660
348041347	CENPL	centromere protein L	0.663
171846573	FBXL4	F-box and leucine rich repeat protein 4	0.663
564367481	TMEM63B	transmembrane protein 63B	0.663
149047880	SCAI	suppressor of cancer cell invasion	0.664
209529687	TMTC3	transmembrane and tetratricopeptide repeat containing 3	0.664
564309890	CBL	Cbl proto-oncogene	0.665
149041414	TBCEL	tubulin folding cofactor E like	0.666
564320724	Fbxo38	F-box protein 38	0.667
149019004	IBTK	inhibitor of Bruton tyrosine kinase	0.667
293349986	SMCHD1	structural maintenance of chromosomes flexible hinge domain containing 1	0.667
672065534	IKZF2	IKAROS family zinc finger 2	0.669
56090421	PXYLP1	2-phosphoxylose phosphatase 1	0.669
829923130	N/A	N/A	0.669
293341533	LOC108348225	feline leukemia virus subgroup C receptor-related protein 1	0.670
431918221	N/A	N/A	0.671
672078177	NYNRIN	NYN domain and retroviral integrase containing	0.673
149042171	RTL5	retrotransposon Gag like 5	0.674
63100438	ZBTB8OS	zinc finger and BTB domain containing 8 opposite strand	0.674
672026767	N/A	N/A	0.676
157786898	ALKBH4	alkB homolog 4, lysine demethylase	0.680
66730382	TRNT1	tRNA nucleotidyl transferase 1	0.684

80861398	CRY1	cryptochrome circadian regulator 1	0.687
157818399	RAI2	retinoic acid induced 2	0.687
672029123	PBRM1	polybromo 1	0.688
197927116	ZNF566	zinc finger protein 566	0.688
564347830	ZXDC	ZXD family zinc finger C	0.688
564320621	DMXL1	Dmx like 1	0.689
124487354	TAF4	TATA-box binding protein associated factor 4	0.689
109479106	WDCP	WD repeat and coiled coil containing	0.689
149031942	N/A	N/A	0.699
31324552	NADSYN1	NAD synthetase 1	0.700
884945546	N/A	N/A	0.700
149025031	SUSD6	sushi domain containing 6	0.704
625182464	N/A	N/A	0.708
568970985	MBTD1	mbt domain containing 1	0.709
884934330	N/A	N/A	0.709
456367253	CEP162	centrosomal protein 162	0.710
157819265	CWC25	CWC25 spliceosome associated protein homolog	0.710
293349917	C2orf72	chromosome 2 open reading frame 72	0.711
157822327	ATG14	autophagy related 14	0.714
157820809	KCTD21	potassium channel tetramerization domain containing 21	0.714
564301426	CNTRL	centriolin	0.718
564312324	ZNF174	zinc finger protein 174	0.719
564352534	Szt2	SZT2, KICSTOR complex subunit	0.723
672015224	N/A	N/A	0.725
564315959	CEP350	centrosomal protein 350	0.730
149025439	DICER1	dicer 1, ribonuclease III	0.743
392341307	Ikzf4	IKAROS family zinc finger 4	0.744
149035563	MUTYH	mutY DNA glycosylase	0.745
564315183	CUX1	cut like homeobox 1	0.748
672084062	TEPP	testis, prostate and placenta expressed	0.748
564395631	Slc25a36l1	solute carrier family 25 (pyrimidine nucleotide carrier), member 36-like 1	0.751
149038509	N/A	N/A	0.754
157822713	NPAT	nuclear protein, coactivator of histone transcription	0.757
67078426	SPIN1	spindlin 1	0.760
564318578	ANKRD28	ankyrin repeat domain 28	0.762
62078827	CTDSPL2	CTD small phosphatase like 2	0.762

564389552	LOC100910854	zinc finger MYND domain-containing protein 19-like	0.764
672055261	N/A	N/A	0.767
149034958	ZNF12	zinc finger protein 12	0.769
117414145	ABCD4	ATP binding cassette subfamily D member 4	0.770
157821277	HAUS3	HAUS augmin like complex subunit 3	0.772
149047034	N/A	N/A	0.772
564320606	SEMA6A	semaphorin 6A	0.776
431916930	N/A	N/A	0.777
83320078	CCDC130	coiled-coil domain containing 130	0.778
672069572	KANSL1	KAT8 regulatory NSL complex subunit 1	0.781
970744322	N/A	N/A	0.781
37360610	FMNL3	formin like 3	0.787
77627983	MLX	MLX, MAX dimerization protein	0.788
399154114	KPNA2	karyopherin subunit alpha 2	0.795
281332148	RIOK1	RIO kinase 1	0.796
672027054	N/A	N/A	0.812
564320728	Fbxo38	F-box protein 38	0.819
19424182	CABP1	calcium binding protein 1	0.821
149018273	XYLB	xylulokinase	0.825
293651589	CPEB2	cytoplasmic polyadenylation element binding protein 2	0.826
74184716	Kat6b	K(lysine) acetyltransferase 6B	0.828
67078462	SOX18	SRY-box 18	0.831
755537242	CLK4	CDC like kinase 4	0.833
149032239	N/A	N/A	0.833
672017191	N/A	N/A	0.834
672014954	DNMBP	dynamin binding protein	0.835
16758212	FUT9	fucosyltransferase 9	0.842
157818841	POGZ	pogo transposable element derived with ZNF domain	0.842
157819737	SARS2	seryl-tRNA synthetase 2, mitochondrial	0.842
672029117	N/A	N/A	0.842
564339225	N/A	N/A	0.844
817337812	N/A	N/A	0.847
109495817	GLT1D1	glycosyltransferase 1 domain containing 1	0.862
16758572	DLK1	delta like non-canonical Notch ligand 1	0.873
300795477	PDZD3	PDZ domain containing 3	0.873
407339769	TTC30A	tetratricopeptide repeat domain 30A	0.880
564400410	AMOT	angiomotin	0.892

293342292	FAM208A	family with sequence similarity 208 member A	0.906
157821209	ZBTB37	zinc finger and BTB domain containing 37	0.906
149035460	N/A	N/A	0.908
564302924	TSHZ2	teashirt zinc finger homeobox 2	0.917
197386987	HDX	highly divergent homeobox	0.922
989935538	N/A	N/A	0.922
68163515	FIBIN	fin bud initiation factor homolog (zebrafish)	0.924
564320454	SAP130	Sin3A associated protein 130	0.927
564317999	N/A	N/A	0.928
71361639	GLI4	GLI family zinc finger 4	0.929
568929584	TRIM62	tripartite motif containing 62	0.929
293347435	PTPRD	protein tyrosine phosphatase, receptor type D	0.930
672035395	DMWD	DM1 locus, WD repeat containing	0.940
564317923	SACS	sacsin molecular chaperone	0.945
704532863	N/A	N/A	0.950
672076513	N/A	N/A	0.950
672062667	N/A	N/A	0.952
392332010	SLC38A10	solute carrier family 38 member 10	0.955
109488483	KIAA0753	KIAA0753	0.960
568916876	GREM1	gremlin 1, DAN family BMP antagonist	0.961
392339806	CFAP69	cilia and flagella associated protein 69	0.964
672025117	MBTD1	mbt domain containing 1	0.965
148687007	CEP128	centrosomal protein 128	0.975
157818625	SLC2A12	solute carrier family 2 member 12	0.979
392340108	TMCC1	transmembrane and coiled-coil domain family 1	0.980
625226711	N/A	N/A	0.980
148747200	KCNJ8	potassium voltage-gated channel subfamily J member 8	0.981
149021160	N/A	N/A	0.981
564304579	ATF7IP	activating transcription factor 7 interacting protein	0.984
19424314	KCNE2	potassium voltage-gated channel subfamily E regulatory subunit 2	0.986
189011634	ARMC7	armadillo repeat containing 7	0.987
293353154	TBC1D1	TBC1 domain family member 1	0.989
829912534	N/A	N/A	0.990
564303143	KMT2C	lysine methyltransferase 2C	0.997
672061705	KMT2A	lysine methyltransferase 2A	1.003
149031942	N/A	N/A	1.003
564298396	ZNF764	zinc finger protein 764	1.006

669303362	N/A	N/A	1.009
148693601	N/A	N/A	1.009
672031484	PCNX2	pecanex homolog 2	1.017
672040273	CPEB3	cytoplasmic polyadenylation element binding protein 3	1.024
564386624	AMER2	APC membrane recruitment protein 2	1.043
568979800	NPAS3	neuronal PAS domain protein 3	1.043
672013187	DMWD	DM1 locus, WD repeat containing	1.051
672044124	N/A	N/A	1.054
564313512	N/A	N/A	1.059
564296988	ZNF235	zinc finger protein 235	1.066
564313514	BPTF	bromodomain PHD finger transcription factor	1.074
672088045	N/A	N/A	1.074
672060610	KMT2D	lysine methyltransferase 2D	1.075
594100882	N/A	N/A	1.075
564317714	Ktn1	kinectin 1	1.078
564300485	LOC102551095	uncharacterized LOC102551095	1.079
426370908	N/A	N/A	1.080
293353154	TBC1D1	TBC1 domain family member 1	1.082
564314535	RUBCN	RUN and cysteine rich domain containing beclin 1 interacting protein	1.084
672058561	N/A	N/A	1.085
672025458	FBF1	Fas binding factor 1	1.090
672028080	PRR14L	proline rich 14 like	1.099
672074697	ILDR2	immunoglobulin like domain containing receptor 2	1.101
564342470	MGA	MGA, MAX dimerization protein	1.104
747019224	SRCAP	Snf2 related CREBBP activator protein	1.107
157822717	ARHGEF19	Rho guanine nucleotide exchange factor 19	1.110
569012000	KLF8	Kruppel like factor 8	1.118
913507039	N/A	N/A	1.120
149058952	MEF2C	myocyte enhancer factor 2C	1.126
149042270	LAS1L	LAS1 like, ribosome biogenesis factor	1.128
392337738	LYSMD4	LysM domain containing 4	1.128
672026785	N/A	N/A	1.128
755566692	HUWE1	HECT, UBA and WWE domain containing 1, E3 ubiquitin protein ligase	1.130
293352381	PAN3	PAN3 poly(A) specific ribonuclease subunit	1.141
157817955	ACER2	alkaline ceramidase 2	1.144
28570188	CLIC6	chloride intracellular channel 6	1.158

564323057	ARMCX4	armadillo repeat containing, X-linked 4	1.159
564341932	HARBI1	harbinger transposase derived 1	1.160
564320724	Fbxo38	F-box protein 38	1.165
149020413	Zfp599	zinc finger protein 599	1.175
255982585	DIO3	iodothyronine deiodinase 3	1.176
724831496	N/A	N/A	1.176
404501478	SLX4IP	SLX4 interacting protein	1.178
13786168	KL	klotho	1.180
672082610	N/A	N/A	1.184
564297736	N/A	N/A	1.184
564321849	KCTD20	potassium channel tetramerization domain containing 20	1.188
148702599	UNK	unkempt family zinc finger	1.188
564303928	TET3	tet methylcytosine dioxygenase 3	1.190
140969817	BPTF	bromodomain PHD finger transcription factor	1.191
672087657	N/A	N/A	1.198
186659510	MYH6	myosin heavy chain 6	1.202
564313512	N/A	N/A	1.206
78097110	N4BP2L1	NEDD4 binding protein 2 like 1	1.208
187957728	FANCM	Fanconi anemia complementation group M	1.210
564378170	PAN3	PAN3 poly(A) specific ribonuclease subunit	1.219
149018731	TMEM108	transmembrane protein 108	1.226
149042882	ZNF334	zinc finger protein 334	1.226
109475482	COL8A2	collagen type VIII alpha 2 chain	1.230
724831496	N/A	N/A	1.240
672083937	TSHZ1	teashirt zinc finger homeobox 1	1.250
149056503	Zfp60	zinc finger protein 60	1.254
829964589	N/A	N/A	1.258
157822425	MFRP	membrane frizzled-related protein	1.264
6978527	AQP1	aquaporin 1 (Colton blood group)	1.265
672032215	REPS2	RALBP1 associated Eps domain containing 2	1.276
293362695	Akap17b	A kinase (PRKA) anchor protein 17B	1.280
672014266	TMEM219	transmembrane protein 219	1.281
149028625	BRDT	bromodomain testis associated	1.286
564296586	Zfp943	zinc finger prtoein 943	1.291
293348214	CCDC88C	coiled-coil domain containing 88C	1.293
121583673	CACTIN	cactin, spliceosome C complex subunit	1.298
755566690	HUWE1	HECT, UBA and WWE domain containing 1, E3 ubiquitin protein ligase	1.299
149035005	IQCE	IQ motif containing E	1.303

568921554	Ank2	ankyrin 2, brain	1.311
61556986	TF	transferrin	1.311
564320109	FAM208B	family with sequence similarity 208 member B	1.318
149016805	PSD3	pleckstrin and Sec7 domain containing 3	1.318
672078055	IRF9	interferon regulatory factor 9	1.330
672040275	CPEB3	cytoplasmic polyadenylation element binding protein 3	1.342
149020413	Zfp599	zinc finger protein 599	1.352
568975399	FAM114A2	family with sequence similarity 114 member A2	1.356
672072794	N/A	N/A	1.363
672089090	LOC103694537	mediator of RNA polymerase II transcription subunit 14-like	1.366
672054310	N/A	N/A	1.366
293352381	PAN3	PAN3 poly(A) specific ribonuclease subunit	1.369
157822929	EPHA2	EPH receptor A2	1.379
293358899	ANKRD26	ankyrin repeat domain 26	1.385
564395676	CDH3	cadherin 3	1.394
149051391	N/A	N/A	1.403
817314209	N/A	N/A	1.407
672087275	KDM5C	lysine demethylase 5C	1.411
672087472	REPS2	RALBP1 associated Eps domain containing 2	1.413
672079407	CCDC66	coiled-coil domain containing 66	1.427
333033763	TTR	transthyretin	1.427
149024626	EXOSC10	exosome component 10	1.428
293347270	OSGIN2	oxidative stress induced growth inhibitor family member 2	1.433
672046728	N/A	N/A	1.446
392340179	RERG	RAS like estrogen regulated growth inhibitor	1.455
392338392	PCNT	pericentrin	1.465
197304784	IQSEC1	IQ motif and Sec7 domain 1	1.468
564300507	SH3D19	SH3 domain containing 19	1.469
568972665	TSPOAP1	TSPO associated protein 1	1.480
149041229	N/A	N/A	1.483
585192925	N/A	N/A	1.489
157821221	TMEM72	transmembrane protein 72	1.500
672088752	MCF2	MCF.2 cell line derived transforming sequence	1.501
392332921	N/A	N/A	1.501
569001477	MTCL1	microtubule crosslinking factor 1	1.517
47059175	GTF2H4	general transcription factor IIH subunit 4	1.520
913498340	N/A	N/A	1.529

564304274	ZNF248	zinc finger protein 248	1.561
961766127	N/A	N/A	1.565
109492822	PAXBP1	PAX3 and PAX7 binding protein 1	1.570
564378315	Zfp853	zinc finger protein 853	1.578
672088045	N/A	N/A	1.585
149059246	N/A	N/A	1.588
564310365	MYO6	myosin VI	1.594
564388164	WAPL	WAPL cohesin release factor	1.599
694937047	ARPC5L	actin related protein 2/3 complex subunit 5 like	1.612
28972866	CSMD3	CUB and Sushi multiple domains 3	1.635
157817406	SKA1	spindle and kinetochore associated complex subunit 1	1.642
672053077	N/A	N/A	1.655
586908220	ARHGAP44	Rho GTPase activating protein 44	1.659
568990288	NIPBL	NIPBL, cohesin loading factor	1.663
149042883	LOC100365365	rCG32328-like	1.665
672080026	N/A	N/A	1.672
149047683	N/A	N/A	1.673
755566690	HUWE1	HECT, UBA and WWE domain containing 1, E3 ubiquitin protein ligase	1.675
564329926	EMSY	EMSY, BRCA2 interacting transcriptional repressor	1.703
68063179	N/A	N/A	1.705
672047351	RALGAPA2	Ral GTPase activating protein catalytic alpha subunit 2	1.714
149045719	PIGO	phosphatidylinositol glycan anchor biosynthesis class O	1.726
672047003	CDAN1	codanin 1	1.728
293340825	USF3	upstream transcription factor family member 3	1.732
564306696	SRBD1	S1 RNA binding domain 1	1.739
564311678	PLEKHM3	pleckstrin homology domain containing M3	1.801
564313028	ATAD5	ATPase family, AAA domain containing 5	1.813
568941582	IQSEC1	IQ motif and Sec7 domain 1	1.836
149056134	Zfp17	zinc finger protein 585B	1.837
392342123	ALS2CL	ALS2 C-terminal like	1.856
61097928	SNAI1	snail family transcriptional repressor 1	1.858
672063748	N/A	N/A	1.858
564395696	TANGO6	transport and golgi organization 6 homolog	1.879
564324736	L3MBTL3	L3MBTL3, histone methyl-lysine binding protein	1.904

564378170	PAN3	PAN3 poly(A) specific ribonuclease subunit	1.907
149016012	KANSL1L	KAT8 regulatory NSL complex subunit 1 like	1.914
149056134	Zfp17	zinc finger protein 585B	1.916
672015368	MAST4	microtubule associated serine/threonine kinase family member 4	1.956
149035338	APBB2	amyloid beta precursor protein binding family B member 2	1.962
672066800	N/A	N/A	1.987
672062667	N/A	N/A	2.000
40786461	NAPEPLD	N-acyl phosphatidylethanolamine phospholipase D	2.014
149019127	N/A	N/A	2.016
946681954	N/A	N/A	2.040
672025361	BPTF	bromodomain PHD finger transcription factor	2.077
124378035	TNRC6C	trinucleotide repeat containing 6C	2.132
157824012	TRIM45	tripartite motif containing 45	2.144
672013014	N/A	N/A	2.147
564313510	BPTF	bromodomain PHD finger transcription factor	2.170
568907669	NYAP2	neuronal tyrosine-phosphorylated phosphoinositide-3-kinase adaptor 2	2.190
148675846	FAM114A2	family with sequence similarity 114 member A2	2.238
149048910	N/A	N/A	2.239
564314522	LRCH3	leucine rich repeats and calponin homology domain containing 3	2.265
672023724	PRICKLE4	prickle planar cell polarity protein 4	2.271
564320493	KDM3B	lysine demethylase 3B	2.284
564306247	PHACTR4	phosphatase and actin regulator 4	2.325
672063750	N/A	N/A	2.347
293351303	METTL22	methyltransferase like 22	2.365
672022282	KIF21A	kinesin family member 21A	2.374
564312627	ZFP62	ZFP62 zinc finger protein	2.394
564309671	KIAA0895	KIAA0895	2.415
755548575	N/A	N/A	2.415
50510463	PRUNE2	prune homolog 2	2.470
149060669	LRCH3	leucine rich repeats and calponin homology domain containing 3	2.478
564303135	KMT2C	lysine methyltransferase 2C	2.505
78000177	SORBS1	sorbin and SH3 domain containing 1	2.557
672063748	N/A	N/A	2.602
158303341	MPP4	membrane palmitoylated protein 4	2.655

672084625	LOC100909409 (includes others)	RGD1562660	2.679
625292335	N/A	N/A	2.696
568979790	NPAS3	neuronal PAS domain protein 3	2.794
564305043	RBM12B	RNA binding motif protein 12B	2.803
148676240	DPH7	diphthamide biosynthesis 7	2.807
569009290	TENM1	teneurin transmembrane protein 1	2.844
149066285	PHF20L1	PHD finger protein 20 like 1	2.858
392340509	PTPRD	protein tyrosine phosphatase, receptor type D	2.880
293349593	Pot1b	protection of telomeres 1B	2.890
564329920	EMSY	EMSY, BRCA2 interacting transcriptional repressor	2.899
564342627	TP53BP1	tumor protein p53 binding protein 1	2.920
157820641	CLDN2	claudin 2	2.963
84000579	FTL	ferritin light chain	2.972
50370130	PALLD	palladin, cytoskeletal associated protein	3.018
755495595	PRRC2C	proline rich coiled-coil 2C	3.040
672063332	N/A	N/A	3.126
564302768	KIAA1755	KIAA1755	3.150
672048712	KMT2C	lysine methyltransferase 2C	3.209
9507065	SCN11A	sodium voltage-gated channel alpha subunit 11	3.248
672022268	KIF21A	kinesin family member 21A	3.359
672054835	N/A	N/A	3.372
564312515	SH3PXD2B	SH3 and PX domains 2B	3.490
672060073	CCNT1	cyclin T1	3.492
564376498	N/A	N/A	3.511
392342412	N/A	N/A	3.636
564309734	IGSF9B	immunoglobulin superfamily member 9B	3.710
149046828	N/A	N/A	3.758
803286756	N/A	N/A	3.794
392351087	HAGHL	hydroxyacylglutathione hydrolase like	3.871
307548437	NYAP2	neuronal tyrosine-phosphorylated phosphoinositide-3-kinase adaptor 2	4.459
564297850	CRTC3	CREB regulated transcription coactivator 3	4.524
164448680	HBB	hemoglobin subunit beta	4.532
564307783	TECPR2	tectonin beta-propeller repeat containing 2	4.728
568906591	UNC80	unc-80 homolog, NALCN channel complex subunit	4.755
293347435	PTPRD	protein tyrosine phosphatase, receptor type D	4.807
672023724	PRICKLE4	prickle planar cell polarity protein 4	4.858

149059823	N/A	N/A	5.000
293340128	MIEF2	mitochondrial elongation factor 2	5.030
62644808	ADAMTSL2	ADAMTS like 2	5.044
293358899	ANKRD26	ankyrin repeat domain 26	5.087
564307792	TECPR2	tectonin beta-propeller repeat containing 2	5.129
672020628	ATXN7L1	ataxin 7 like 1	5.170
13027424	TRPV4	transient receptor potential cation channel subfamily V member 4	5.170
564312627	ZFP62	ZFP62 zinc finger protein	5.170
564297371	LOC102556967	zinc finger protein 484-like	5.248
568939804	N/A	N/A	5.300
564303143	KMT2C	lysine methyltransferase 2C	5.322
564308814	N/A	N/A	5.322
672023090	N/A	N/A	5.322
24962814	KCNJ14	potassium voltage-gated channel subfamily J member 14	5.375
564329918	EMSY	EMSY, BRCA2 interacting transcriptional repressor	5.476
149052383	TRIM7	tripartite motif containing 7	5.524
564367862	Dst	dystonin	5.565
8394422	TBXA2R	thromboxane A2 receptor	5.672
392339806	CFAP69	cilia and flagella associated protein 69	5.728
149050030	MTRF1	mitochondrial translation release factor 1	5.755
148696094	TUBGCP4	tubulin gamma complex associated protein 4	5.755
149042274	ZC3H12B	zinc finger CCCH-type containing 12B	5.755
149064065	ZMYM5	zinc finger MYM-type containing 5	5.781
564296586	Zfp943	zinc finger prtoein 943	5.807
672017085	N/A	N/A	5.858
672063678	N/A	N/A	5.907
18677749	DHRS9	dehydrogenase/reductase 9	6.022
672026875	N/A	N/A	6.044
564316929	FRYL	FRY like transcription coactivator	6.087
296473176	N/A	N/A	6.087
564341215	COBLL1	cordon-bleu WH2 repeat protein like 1	6.109
293347270	OSGIN2	oxidative stress induced growth inhibitor family member 2	6.129
564317927	SACS	sacsin molecular chaperone	6.129
149050865	ITSN2	intersectin 2	6.150
625198911	N/A	N/A	6.248
149031942	N/A	N/A	6.248

148702471	N/A	N/A	6.248
564315188	CUX1	cut like homeobox 1	6.267
392340768	DISP3	dispatched RND transporter family member 3	6.267
293344867	N/A	N/A	6.322
672020363	N/A	N/A	6.340
408407614	DNA2	DNA replication helicase/nuclease 2	6.358
672052120	RBM12B	RNA binding motif protein 12B	6.358
672052394	N/A	N/A	6.358
672019789	BTBD19	BTB domain containing 19	6.375
6754958	OTP	orthopedia homeobox	6.409
970703429	N/A	N/A	6.570
564312007	COL6A3	collagen type VI alpha 3 chain	6.585
564312944	KIAA0753	KIAA0753	6.585
293347435	PTPRD	protein tyrosine phosphatase, receptor type D	6.585
672051732	N/A	N/A	6.658
149055517	Hmgn5/Hmgn5b	high mobility group nucleosome binding domain 5	6.672
122114537	VPS13C	vacuolar protein sorting 13 homolog C	6.687
672013014	N/A	N/A	6.687
564318175	DGKH	diacylglycerol kinase eta	6.714
8393418	GAPDH	glyceraldehyde-3-phosphate dehydrogenase	6.728
166235131	MTCL1	microtubule crosslinking factor 1	6.741
672049060	N/A	N/A	6.741
77404174	HLA-A	major histocompatibility complex, class I, A	6.755
672088045	N/A	N/A	6.755
672071273	GRAMD1C	GRAM domain containing 1C	6.768
564351511	RGD1565987	similar to F-box and leucine-rich repeat protein 18	6.768
149047826	RIF1	replication timing regulatory factor 1	6.768
149031942	N/A	N/A	6.768
672019792	N/A	N/A	6.781
568995287	MKL2	MKL1/myocardin like 2	6.807
564313845	SLC38A10	solute carrier family 38 member 10	6.807
392334596	RSPH3	radial spoke head 3 homolog	6.820
564375742	SLC26A11	solute carrier family 26 member 11	6.845
594061361	N/A	N/A	6.845
149042879	N/A	N/A	6.931
672088045	N/A	N/A	6.931
149020581	ZNF560	zinc finger protein 560	6.989
149038394	N/A	N/A	7.033

157822579	UCKL1	uridine-cytidine kinase 1 like 1	7.044
564396366	PCNX2	pecanex homolog 2	7.140
149026222	N/A	N/A	7.150
564305557	PTPRD	protein tyrosine phosphatase, receptor type D	7.180
148666118	N/A	N/A	7.190
672029704	ZCCHC6	zinc finger CCHC-type containing 6	7.200
157818983	SIRT7	sirtuin 7	7.248
148670274	KIAA0368	KIAA0368	7.313
197333844	LLPH	LLP homolog, long-term synaptic facilitation	7.358
293347435	PTPRD	protein tyrosine phosphatase, receptor type D	7.362
149016584	ZNF606	zinc finger protein 606	7.492
564301284	Ttf1	transcription termination factor, RNA polymerase I	7.524
755566690	HUWE1	HECT, UBA and WWE domain containing 1, E3 ubiquitin protein ligase	7.555
564322493	RPGR	retinitis pigmentosa GTPase regulator	7.562
149055413	ZMAT1	zinc finger matrin-type 1	7.570
672027860	APBB2	amyloid beta precursor protein binding family B member 2	7.577
672086880	N/A	N/A	7.665
663243313	N/A	N/A	7.687
564302105	ZNF770	zinc finger protein 770	7.714
564329920	EMSY	EMSY, BRCA2 interacting transcriptional repressor	7.728
109492822	PAXBP1	PAX3 and PAX7 binding protein 1	7.735
149029159	N/A	N/A	7.768
672016875	LOC103690320	FERM and PDZ domain-containing protein 3	7.801
672066298	N/A	N/A	7.807
149064803	NHLRC3	NHL repeat containing 3	7.852
62650795	DACT1	dishevelled binding antagonist of beta catenin 1	7.895
149057336	ZSCAN2	zinc finger and SCAN domain containing 2	7.925
672089126	Kdm5d	lysine demethylase 5D	7.943
564323057	ARMCX4	armadillo repeat containing, X-linked 4	7.977
29500537	ADAMTS20	ADAM metallopeptidase with thrombospondin type 1 motif 20	8.022
672069572	KANSL1	KAT8 regulatory NSL complex subunit 1	8.087
300797609	ELOVL7	ELOVL fatty acid elongase 7	8.155
392331668	HAGHL	hydroxyacylglutathione hydrolase like	8.160
564297387	Zfp658	zinc finger protein 658	8.160
564314663	VPS8	VPS8, CORVET complex subunit	8.170

392337738	LYSMD4	LysM domain containing 4	8.195
293356488	RIC1	RIC1 homolog, RAB6A GEF complex partner 1	8.209
109472884	UBE3C	ubiquitin protein ligase E3C	8.238
564339233	LOC100909794	afadin- and alpha-actinin-binding protein-like	8.257
755566692	HUWE1	HECT, UBA and WWE domain containing 1, E3 ubiquitin protein ligase	8.326
392340768	DISP3	dispatched RND transporter family member 3	8.358
564321167	CHD9	chromodomain helicase DNA binding protein 9	8.430
149020413	Zfp599	zinc finger protein 599	8.484
147907212	ASAP2	ArfGAP with SH3 domain, ankyrin repeat and PH domain 2	8.551
672088045	N/A	N/A	8.581
6681095	CYCS	cytochrome c, somatic	8.676
672074697	ILDR2	immunoglobulin like domain containing receptor 2	8.752
564377502	EIF4G1	eukaryotic translation initiation factor 4 gamma 1	8.807
672029702	ZCCHC6	zinc finger CCHC-type containing 6	8.830
564311685	PIKFYVE	phosphoinositide kinase, FYVE-type zinc finger containing	8.867
672074711	N/A	N/A	9.093
672023724	PRICKLE4	prickle planar cell polarity protein 4	9.222
672085486	EGLN1	egl-9 family hypoxia inducible factor 1	9.267
109480102	SMARCC2	SWI/SNF related, matrix associated, actin dependent regulator of chromatin subfamily c member 2	10.050
38454242	EIF3H	eukaryotic translation initiation factor 3 subunit H	10.430
**DEGs in female**			
**ID**	**Symbol**	**Entrez Gene Name**	**Expr Log Ratio**
672089449	N/A	N/A	-10.534
564315358	N/A	N/A	-9.721
148675846	FAM114A2	family with sequence similarity 114 member A2	-9.326
564316241	CEP170	centrosomal protein 170	-9.267
148223355	ASAP2	ArfGAP with SH3 domain, ankyrin repeat and PH domain 2	-9.238

672027854	APBB2	amyloid beta precursor protein binding family B member 2	-8.607
884934330	N/A	N/A	-8.607
564317925	SACS	sacsin molecular chaperone	-8.592
58865558	TUBA1C	tubulin alpha 1c	-8.592
672088045	N/A	N/A	-8.555
672053062	FKBP15	FK506 binding protein 15	-8.547
568979800	NPAS3	neuronal PAS domain protein 3	-8.531
79750234	ZFP36L2	ZFP36 ring finger protein like 2	-8.484
154090947	NPAS3	neuronal PAS domain protein 3	-8.476
149050865	ITSN2	intersectin 2	-8.430
672088045	N/A	N/A	-8.317
564388164	WAPL	WAPL cohesin release factor	-8.267
564314997	IQCE	IQ motif containing E	-8.229
672085107	WDR59	WD repeat domain 59	-8.200
672075678	N/A	N/A	-8.129
564311697	PIKFYVE	phosphoinositide kinase, FYVE-type zinc finger containing	-8.087
987424694	N/A	N/A	-8.077
392337738	LYSMD4	LysM domain containing 4	-8.061
293347435	PTPRD	protein tyrosine phosphatase, receptor type D	-8.061
109488483	KIAA0753	KIAA0753	-8.017
149060669	LRCH3	leucine rich repeats and calponin homology domain containing 3	-7.972
672087657	N/A	N/A	-7.948
392331954	KANSL1	KAT8 regulatory NSL complex subunit 1	-7.937
149020413	Zfp599	zinc finger protein 599	-7.931
913507039	N/A	N/A	-7.907
884945546	N/A	N/A	-7.845
564380050	2410141K09Rik	RIKEN cDNA 2410141K09 gene	-7.827
564298047	GDPD5	glycerophosphodiester phosphodiesterase domain containing 5	-7.807
564306382	FHAD1	forkhead associated phosphopeptide binding domain 1	-7.801
568914626	GARNL3	GTPase activating Rap/RanGAP domain like 3	-7.794
148669850	GFRA1	GDNF family receptor alpha 1	-7.781
392334596	RSPH3	radial spoke 3 homolog	-7.768
672023280	N/A	N/A	-7.735
31745164	HAX1	HCLS1 associated protein X-1	-7.714
672057084	N/A	N/A	-7.651

148676240	DPH7	diphthamide biosynthesis 7	-7.622
148670273	KIAA0368	KIAA0368	-7.615
148670274	KIAA0368	KIAA0368	-7.577
68063179	N/A	N/A	-7.577
755566690	HUWE1	HECT, UBA and WWE domain containing 1, E3 ubiquitin protein ligase	-7.570
154090947	NPAS3	neuronal PAS domain protein 3	-7.562
672039093	N/A	N/A	-7.547
149029159	N/A	N/A	-7.516
921126126	N/A	N/A	-7.516
913486723	N/A	N/A	-7.508
564309742	IGSF9B	immunoglobulin superfamily member 9B	-7.500
148682823	N/A	N/A	-7.492
564306888	N/A	N/A	-7.476
28972363	DOCK4	dedicator of cytokinesis 4	-7.468
109480728	TMEM74	transmembrane protein 74	-7.468
392331668	HAGHL	hydroxyacylglutathione hydrolase-like	-7.435
564352536	Szt2	seizure threshold 2 homolog (mouse)	-7.435
564316929	FRYL	FRY like transcription coactivator	-7.426
149051960	FLYWCH2	FLYWCH family member 2	-7.418
568979796	NPAS3	neuronal PAS domain protein 3	-7.349
564307173	HEATR5A	HEAT repeat containing 5A	-7.340
672013187	DMWD	dystrophia myotonica, WD repeat containing	-7.313
564307783	TECPR2	tectonin beta-propeller repeat containing 2	-7.276
148675846	FAM114A2	family with sequence similarity 114 member A2	-7.267
724831496	N/A	N/A	-7.267
755566692	HUWE1	HECT, UBA and WWE domain containing 1, E3 ubiquitin protein ligase	-7.238
672084304	N/A	N/A	-7.238
564375502	Mxra7	matrix-remodelling associated 7	-7.209
564310188	IGDCC4	immunoglobulin superfamily DCC subclass member 4	-7.170
564320724	Fbxo38	F-box protein 38	-7.150
755531756	N/A	N/A	-7.109
149055517	Hmgn5/Hmgn5b	high mobility group nucleosome binding domain 5B	-7.087
947311993	N/A	N/A	-7.077
694937047	ARPC5L	actin related protein 2/3 complex subunit 5 like	-7.066
564313510	BPTF	bromodomain PHD finger transcription factor	-7.044
672088045	N/A	N/A	-7.033

672031167	C19orf57	chromosome 19 open reading frame 57	-7.011
672052394	N/A	N/A	-7.000
148694630	N/A	N/A	-6.977
149041058	RCOR3	REST corepressor 3	-6.966
149054804	N/A	N/A	-6.954
564314389	DZIP3	DAZ interacting zinc finger protein 3	-6.919
392339806	CFAP69	cilia and flagella associated protein 69	-6.907
293344558	PCNX3	pecanex homolog 3 (Drosophila)	-6.895
672042306	N/A	N/A	-6.891
672023474	NKTR	natural killer cell triggering receptor	-6.858
122114537	VPS13C	vacuolar protein sorting 13 homolog C	-6.845
672050496	IQSEC1	IQ motif and Sec7 domain 1	-6.833
564301284	Ttf1	transcription termination factor, RNA polymerase I	-6.807
625198911	N/A	N/A	-6.781
392339806	CFAP69	cilia and flagella associated protein 69	-6.741
62198215	C4BPB	complement component 4 binding protein beta	-6.728
34877176	GPRIN2	G protein regulated inducer of neurite outgrowth 2	-6.728
564367862	Dst	dystonin	-6.714
149035005	IQCE	IQ motif containing E	-6.693
568961599	VPS13C	vacuolar protein sorting 13 homolog C	-6.672
564313180	HLF	HLF, PAR bZIP transcription factor	-6.658
672040275	CPEB3	cytoplasmic polyadenylation element binding protein 3	-6.644
672074697	ILDR2	immunoglobulin like domain containing receptor 2	-6.615
294610780	SYNE2	spectrin repeat containing nuclear envelope protein 2	-6.615
62078965	SLC47A1	solute carrier family 47 member 1	-6.585
884945546	N/A	N/A	-6.585
154090947	NPAS3	neuronal PAS domain protein 3	-6.570
564305252	FBXO10	F-box protein 10	-6.508
564302768	KIAA1755	KIAA1755	-6.508
672023724	PRICKLE4	prickle planar cell polarity protein 4	-6.476
149053275	N/A	N/A	-6.476
672035395	DMWD	dystrophia myotonica, WD repeat containing	-6.443
564381298	KIAA1614	KIAA1614	-6.443
672063678	N/A	N/A	-6.443
564317925	SACS	sacsin molecular chaperone	-6.392

149057336	ZSCAN2	zinc finger and SCAN domain containing 2	-6.285
672074711	N/A	N/A	-6.285
293344988	CCDC138	coiled-coil domain containing 138	-6.267
148679862	SLC35F3	solute carrier family 35 member F3	-6.267
149052470	ZNF454	zinc finger protein 454	-6.248
672082610	N/A	N/A	-6.170
140969817	BPTF	bromodomain PHD finger transcription factor	-6.098
569009290	TENM1	teneurin transmembrane protein 1	-6.044
672084304	N/A	N/A	-5.977
672026875	N/A	N/A	-5.931
672065933	DOCK10	dedicator of cytokinesis 10	-5.907
47058988	TOMM70	translocase of outer mitochondrial membrane 70	-5.888
672026416	PRR36	proline rich 36	-5.883
672017191	N/A	N/A	-5.858
564313516	BPTF	bromodomain PHD finger transcription factor	-5.833
672024381	N/A	N/A	-5.781
564347830	ZXDC	ZXD family zinc finger C	-5.700
37360610	FMNL3	formin like 3	-5.693
157818655	MPZL2	myelin protein zero like 2	-5.672
392332008	SLC38A10	solute carrier family 38 member 10	-5.672
672029704	ZCCHC6	zinc finger CCHC-type containing 6	-5.640
149031942	N/A	N/A	-5.555
755548575	N/A	N/A	-5.524
207522	TNNT2	troponin T2, cardiac type	-5.403
21426773	ASPG	asparaginase	-5.392
149067796	TMEM219	transmembrane protein 219	-5.392
755519636	ZNF536	zinc finger protein 536	-5.358
564393142	WDR36	WD repeat domain 36	-5.248
149024772	N/A	N/A	-5.209
149031942	N/A	N/A	-5.129
564303143	KMT2C	lysine methyltransferase 2C	-5.044
157822843	WNT6	Wnt family member 6	-5.030
293341811	PRR14L	proline rich 14 like	-4.907
149042274	ZC3H12B	zinc finger CCCH-type containing 12B	-4.907
140969817	BPTF	bromodomain PHD finger transcription factor	-4.858
672031484	PCNX2	pecanex homolog 2 (Drosophila)	-4.858
148684403	N/A	N/A	-4.858
568941572	IQSEC1	IQ motif and Sec7 domain 1	-4.807
55741514	TSEN34	tRNA splicing endonuclease subunit 34	-4.780

568977658	ASAP2	ArfGAP with SH3 domain, ankyrin repeat and PH domain 2	-4.720
564367862	Dst	dystonin	-4.700
149067879	NUPR1	nuclear protein 1, transcriptional regulator	-4.591
109460021	KIAA2026	KIAA2026	-4.585
625217108	N/A	N/A	-4.433
564313508	BPTF	bromodomain PHD finger transcription factor	-4.392
392348187	LRP8	LDL receptor related protein 8	-4.392
16758400	KCNJ13	potassium voltage-gated channel subfamily J member 13	-4.256
156119589	FOXC2	forkhead box C2	-4.209
564313782	Tha1	threonine aldolase 1	-4.157
564313676	FBF1	Fas binding factor 1	-4.150
672035060	CIC	capicua transcriptional repressor	-3.861
149018115	N/A	N/A	-3.845
564307245	MIA2	melanoma inhibitory activity 2	-3.815
149064065	ZMYM5	zinc finger MYM-type containing 5	-3.661
672041794	RGD1310081	similar to hypothetical protein FLJ13231	-3.585
148703035	CLDN11	claudin 11	-3.579
6981430	PTGDS	prostaglandin D2 synthase	-3.532
564315188	CUX1	cut like homeobox 1	-3.459
62079023	ADTRP	androgen dependent TFPI regulating protein	-3.435
21326469	SLC15A3	solute carrier family 15 member 3	-3.322
672022290	KIF21A	kinesin family member 21A	-3.303
189083739	ANKS4B	ankyrin repeat and sterile alpha motif domain containing 4B	-3.298
672072352	Evi5l	ecotropic viral integration site 5 like	-3.280
157817652	BNC2	basonuclin 2	-3.273
58331126	GJB6	gap junction protein beta 6	-3.262
663434101	CUX1	cut like homeobox 1	-3.222
293351303	METTL22	methyltransferase like 22	-3.205
832626572	CHI3L1	chitinase 3 like 1	-3.193
149066285	PHF20L1	PHD finger protein 20-like 1	-3.179
71896590	AOC3	amine oxidase, copper containing 3	-3.170
74202463	EYA2	EYA transcriptional coactivator and phosphatase 2	-3.152
149016131	N/A	N/A	-3.136
255982585	DIO3	iodothyronine deiodinase 3	-3.037
564311681	PIKFYVE	phosphoinositide kinase, FYVE-type zinc finger containing	-3.016

194474002	MEI1	meiotic double-stranded break formation protein 1	-2.970
672087499	N/A	N/A	-2.928
274321177	MCM4	minichromosome maintenance complex component 4	-2.844
148702636	SPHK1	sphingosine kinase 1	-2.829
568964944	EPB41L2	erythrocyte membrane protein band 4.1 like 2	-2.823
564324736	L3MBTL3	l(3)mbt-like 3 (Drosophila)	-2.759
827012496	NLRC4	NLR family CARD domain containing 4	-2.652
157820611	INSC	inscuteable homolog (Drosophila)	-2.609
672075760	RASSF6	Ras association domain family member 6	-2.605
274315796	SNTB2	syntrophin beta 2	-2.602
162138928	SLC13A3	solute carrier family 13 member 3	-2.596
57527344	OASL	2'-5'-oligoadenylate synthetase like	-2.585
149054972	SLC26A11	solute carrier family 26 member 11	-2.567
672053075	N/A	N/A	-2.555
564314671	VPS8	VPS8, CORVET complex subunit	-2.531
672028646	N/A	N/A	-2.521
157819447	HYKK	hydroxylysine kinase	-2.515
755551565	CSMD3	CUB and Sushi multiple domains 3	-2.503
195540030	Tnxa-ps1	tenascin XA, pseudogene 1	-2.500
672062667	N/A	N/A	-2.459
8393733	LOX	lysyl oxidase	-2.445
148689488	SYN3	synapsin III	-2.438
209571549	RNF43	ring finger protein 43	-2.433
564312515	SH3PXD2B	SH3 and PX domains 2B	-2.418
60460909	SLC13A4	solute carrier family 13 member 4	-2.405
564342627	TP53BP1	tumor protein p53 binding protein 1	-2.392
672088296	N/A	N/A	-2.390
564390739	N/A	N/A	-2.382
112984202	FZD8	frizzled class receptor 8	-2.375
672033554	LOC102557335	uncharacterized LOC102557335	-2.368
672029117	N/A	N/A	-2.367
6981628	TACR1	tachykinin receptor 1	-2.361
19424240	PCSK4	proprotein convertase subtilisin/kexin type 4	-2.337
672079259	N/A	N/A	-2.312
148702471	N/A	N/A	-2.229
109495817	GLT1D1	glycosyltransferase 1 domain containing 1	-2.228
58865522	TEKT2	tektin 2	-2.228
25742828	SCN7A	sodium voltage-gated channel alpha subunit 7	-2.202

148664561	DTNA	dystrobrevin alpha	-2.190
672022282	KIF21A	kinesin family member 21A	-2.181
16758354	TINAGL1	tubulointerstitial nephritis antigen like 1	-2.178
194239635	Tpsab1	tryptase alpha/beta 1	-2.176
13929066	CPZ	carboxypeptidase Z	-2.170
25453404	Crabp2/LOC100911902	cellular retinoic acid binding protein 2	-2.168
625206860	N/A	N/A	-2.159
157816965	DKK2	dickkopf WNT signaling pathway inhibitor 2	-2.144
672022268	KIF21A	kinesin family member 21A	-2.143
672029435	N/A	N/A	-2.140
226823227	UBE2T	ubiquitin conjugating enzyme E2 T	-2.139
158303341	MPP4	membrane palmitoylated protein 4	-2.138
6981068	ICAM1	intercellular adhesion molecule 1	-2.133
9506725	GJA5	gap junction protein alpha 5	-2.127
564336264	BBS12	Bardet-Biedl syndrome 12	-2.126
18677739	CDKN2B	cyclin dependent kinase inhibitor 2B	-2.121
672019901	LOC100361036	rCG31267-like	-2.121
54312106	ZNF705A	zinc finger protein 705A	-2.107
148675846	FAM114A2	family with sequence similarity 114 member A2	-2.092
672025368	BPTF	bromodomain PHD finger transcription factor	-2.074
48675870	PPP1R3B	protein phosphatase 1 regulatory subunit 3B	-2.066
149044855	N/A	N/A	-2.062
672047351	RALGAPA2	Ral GTPase activating protein catalytic alpha subunit 2	-2.023
149042879	N/A	N/A	-2.018
58293772	AMY2A	amylase, alpha 2A (pancreatic)	-2.012
58865654	EFEMP1	EGF containing fibulin like extracellular matrix protein 1	-2.006
8393186	CPS1	carbamoyl-phosphate synthase 1	-2.000
577861042	PTGS1	prostaglandin-endoperoxide synthase 1	-2.000
293351303	METTL22	methyltransferase like 22	-1.995
970699122	N/A	N/A	-1.989
300795477	PDZD3	PDZ domain containing 3	-1.983
480306394	Mcpt4	mast cell protease 4	-1.978
672013187	DMWD	dystrophia myotonica, WD repeat containing	-1.977
58865768	XKR8	XK related 8	-1.972
568933200	WDR86	WD repeat domain 86	-1.957
158138505	SLC5A5	solute carrier family 5 member 5	-1.954
149060001	N/A	N/A	-1.951

157786780	MELTF	melanotransferrin	-1.942
672029702	ZCCHC6	zinc finger CCHC-type containing 6	-1.917
149017274	N/A	N/A	-1.914
392332921	N/A	N/A	-1.911
672022657	N/A	N/A	-1.891
564297387	Zfp658	zinc finger protein 658	-1.890
158186711	F13A1	coagulation factor XIII A chain	-1.886
672032217	REPS2	RALBP1 associated Eps domain containing 2	-1.883
149053435	N/A	N/A	-1.883
149042883	LOC100365365	rCG32328-like	-1.882
564342320	FSIP1	fibrous sheath interacting protein 1	-1.881
226958688	RBP4	retinol binding protein 4	-1.867
149061735	IGF2	insulin like growth factor 2	-1.858
149050087	N/A	N/A	-1.848
293341722	N/A	N/A	-1.840
672082610	N/A	N/A	-1.830
18959230	SLC6A20	solute carrier family 6 member 20	-1.823
672017085	N/A	N/A	-1.820
300798520	PRRG1	proline rich and Gla domain 1	-1.812
672062795	N/A	N/A	-1.811
564312009	COL6A3	collagen type VI alpha 3 chain	-1.801
55741827	TERT	telomerase reverse transcriptase	-1.801
169234643	SLC16A11	solute carrier family 16 member 11	-1.797
31542125	ALOX15	arachidonate 15-lipoxygenase	-1.796
76443687	SLC4A1	solute carrier family 4 member 1 (Diego blood group)	-1.794
149024371	SH2D5	SH2 domain containing 5	-1.778
564395676	CDH3	cadherin 3	-1.766
672085823	N/A	N/A	-1.762
564317122	CC2D2A	coiled-coil and C2 domain containing 2A	-1.755
564356749	N/A	N/A	-1.753
564321163	CHD9	chromodomain helicase DNA binding protein 9	-1.750
297374767	TPSAB1/TPSB2	tryptase alpha/beta 1	-1.750
295391913	LOC100366054	Da1-10-like	-1.742
532056233	N/A	N/A	-1.739
157823213	CAPSL	calcyphosine like	-1.732
537264393	N/A	N/A	-1.726
148704234	GJB2	gap junction protein beta 2	-1.724
564380050	2410141K09Rik	RIKEN cDNA 2410141K09 gene	-1.712
157823809	CD163	CD163 molecule	-1.710

564315753	N/A	N/A	-1.689
61557187	Apol3	apolipoprotein L, 3	-1.688
157819493	Igbp1b	immunoglobulin (CD79A) binding protein 1b	-1.681
397787567	BDKRB2	bradykinin receptor B2	-1.678
672043249	DENND4B	DENN domain containing 4B	-1.673
6981176	MAK	male germ cell associated kinase	-1.670
672022651	N/A	N/A	-1.667
672028781	NAA16	N(alpha)-acetyltransferase 16, NatA auxiliary subunit	-1.666
672074150	N/A	N/A	-1.665
149055413	ZMAT1	zinc finger matrin-type 1	-1.660
149053793	TSPOAP1	TSPO associated protein 1	-1.659
68163517	CCDC146	coiled-coil domain containing 146	-1.652
564297823	KIF7	kinesin family member 7	-1.650
155369293	AEBP1	AE binding protein 1	-1.644
218156285	CFB	complement factor B	-1.641
149052383	TRIM7	tripartite motif containing 7	-1.639
672034794	N/A	N/A	-1.636
156347833	N/A	N/A	-1.635
67846074	EHD2	EH domain containing 2	-1.629
672045595	RIF1	replication timing regulatory factor 1	-1.618
6980976	GPC3	glypican 3	-1.609
564337843	LOC100363520	mCG16729-like	-1.609
672028287	Kat6b	K(lysine) acetyltransferase 6B	-1.608
42476116	FBLN5	fibulin 5	-1.598
62644808	ADAMTSL2	ADAMTS like 2	-1.593
672038314	N/A	N/A	-1.591
148675846	FAM114A2	family with sequence similarity 114 member A2	-1.585
564313512	N/A	N/A	-1.585
149064803	NHLRC3	NHL repeat containing 3	-1.580
568972622	BPTF	bromodomain PHD finger transcription factor	-1.576
568941576	IQSEC1	IQ motif and Sec7 domain 1	-1.575
9506461	CAPN1	calpain 1	-1.574
655644820	N/A	N/A	-1.572
6981280	NPR1	natriuretic peptide receptor 1	-1.562
187281975	DENND1C	DENN domain containing 1C	-1.559
148686343	MRGPRF	MAS related GPR family member F	-1.547
564312671	N/A	N/A	-1.546
672083480	TNFAIP8	TNF alpha induced protein 8	-1.545
9910378	CDC42SE2	CDC42 small effector 2	-1.544

672085160	N/A	N/A	-1.537
564320724	Fbxo38	F-box protein 38	-1.532
56961645	Fcna	ficolin A	-1.532
51491891	CASP6	caspase 6	-1.524
451770406	Nlrp1a	NLR family, pyrin domain containing 1A	-1.524
225735575	ALDH1A2	aldehyde dehydrogenase 1 family member A2	-1.521
1698696	CMA1	chymase 1	-1.521
157823171	NME4	NME/NM23 nucleoside diphosphate kinase 4	-1.520
281371494	LAMC2	laminin subunit gamma 2	-1.518
148680846	HIC1	HIC ZBTB transcriptional repressor 1	-1.507
672066486	SHISA9	shisa family member 9	-1.507
2231145	N/A	N/A	-1.499
157821719	CPM	carboxypeptidase M	-1.498
57516	ATAD3B	ATPase family, AAA domain containing 3B	-1.497
109480098	SMARCC2	SWI/SNF related, matrix associated, actin dependent regulator of chromatin subfamily c member 2	-1.490
157823079	RBKS	ribokinase	-1.484
672070303	N/A	N/A	-1.481
568924481	COL25A1	collagen type XXV alpha 1 chain	-1.470
672071704	N/A	N/A	-1.470
564298020	KCTD14	potassium channel tetramerization domain containing 14	-1.461
672053969	Ccdc30	coiled-coil domain containing 30	-1.459
157820793	RTBDN	retbindin	-1.452
672063745	SEC22C	SEC22 homolog C, vesicle trafficking protein	-1.446
768033504	KDM6A	lysine demethylase 6A	-1.442
672054310	N/A	N/A	-1.442
564366187	LOC100361039 (includes others)	similar to nidogen 2	-1.441
564300462	DCHS2	dachsous cadherin-related 2	-1.430
148694035	SENP8	SUMO/sentrin peptidase family member, NEDD8 specific	-1.427
672018107	N/A	N/A	-1.426
213512704	CPA3	carboxypeptidase A3	-1.424
672088045	N/A	N/A	-1.421
399154114	KPNA2	karyopherin subunit alpha 2	-1.399
672023090	N/A	N/A	-1.391
672020326	MTA3	metastasis associated 1 family member 3	-1.390
149053909	COL1A1	collagen type I alpha 1 chain	-1.388

197384727	Smco4	single-pass membrane protein with coiled-coil domains 4	-1.384
201861483	LOC102548396 (includes others)	zinc finger protein 951	-1.382
672029180	CCSER2	coiled-coil serine rich protein 2	-1.381
149016574	ZNF324	zinc finger protein 324	-1.381
564297942	LOC103690302	AF4/FMR2 family member 2	-1.379
149018731	TMEM108	transmembrane protein 108	-1.378
392339874	SLC37A3	solute carrier family 37 member 3	-1.374
564310551	N/A	N/A	-1.371
293346251	TMEM62	transmembrane protein 62	-1.368
13929084	THBD	thrombomodulin	-1.366
672064087	N/A	N/A	-1.365
54020664	DCN	decorin	-1.358
149051391	N/A	N/A	-1.356
564350417	SLC26A7	solute carrier family 26 member 7	-1.355
40018618	CBX7	chromobox 7	-1.354
672044124	N/A	N/A	-1.350
320089574	FAM161A	family with sequence similarity 161 member A	-1.342
564320621	DMXL1	Dmx like 1	-1.340
16758284	SLC5A7	solute carrier family 5 member 7	-1.340
149045275	EXOC2	exocyst complex component 2	-1.335
564325156	SYNE1	spectrin repeat containing nuclear envelope protein 1	-1.323
149054264	N/A	N/A	-1.322
16758080	COL1A2	collagen type I alpha 2 chain	-1.318
149069514	N/A	N/A	-1.318
672064422	N/A	N/A	-1.318
568941586	IQSEC1	IQ motif and Sec7 domain 1	-1.317
392338492	TMEM161B	transmembrane protein 161B	-1.313
25742776	MC4R	melanocortin 4 receptor	-1.309
189163513	SH2D1A	SH2 domain containing 1A	-1.308
113205500	STAT6	signal transducer and activator of transcription 6	-1.304
51260641	Ugt1a7c	UDP glucuronosyltransferase 1 family, polypeptide A7C	-1.302
564299864	N/A	N/A	-1.302
149020413	Zfp599	zinc finger protein 599	-1.300
148664646	GYPC	glycophorin C (Gerbich blood group)	-1.298
300794610	PGM5	phosphoglucomutase 5	-1.298

145046230	SPP1	secreted phosphoprotein 1	-1.293
149046828	N/A	N/A	-1.292
672028781	NAA16	N(alpha)-acetyltransferase 16, NatA auxiliary subunit	-1.290
564311658	N/A	N/A	-1.290
564395350	N/A	N/A	-1.284
672066092	UNC80	unc-80 homolog, NALCN activator	-1.273
13591940	DPYD	dihydropyrimidine dehydrogenase	-1.272
149050030	MTRF1	mitochondrial translation release factor 1	-1.269
194097493	EVA1B	eva-1 homolog B	-1.263
672022615	N/A	N/A	-1.263
564317722	N/A	N/A	-1.259
149056256	FXYD5	FXYD domain containing ion transport regulator 5	-1.254
401709944	MPP7	membrane palmitoylated protein 7	-1.252
119226202	CDC42EP1	CDC42 effector protein 1	-1.251
764020103	ROBO4	roundabout guidance receptor 4	-1.251
13786164	CDH1	cadherin 1	-1.250
66730337	NMRK1	nicotinamide riboside kinase 1	-1.250
149025029	SUSD6	sushi domain containing 6	-1.250
30794230	TNFRSF1B	TNF receptor superfamily member 1B	-1.248
157821829	POLI	DNA polymerase iota	-1.244
71043760	RRM2	ribonucleotide reductase regulatory subunit M2	-1.243
564396113	ZCCHC14	zinc finger CCHC-type containing 14	-1.241
149021126	MRC1	mannose receptor C-type 1	-1.240
62656582	KIAA0100	KIAA0100	-1.237
149044212	N/A	N/A	-1.237
392334002	CCDC3	coiled-coil domain containing 3	-1.236
672061705	KMT2A	lysine methyltransferase 2A	-1.236
268607712	SNED1	sushi, nidogen and EGF like domains 1	-1.235
564297338	ZNF816	zinc finger protein 816	-1.235
589934569	N/A	N/A	-1.234
537174241	N/A	N/A	-1.230
148701441	N/A	N/A	-1.227
537234259	N/A	N/A	-1.226
672044124	N/A	N/A	-1.223
19424348	SLC6A13	solute carrier family 6 member 13	-1.220
392334475	RGD1560020_predicted	similar to Myb proto-oncogene protein (C-myb) (predicted)	-1.213
13540656	EMP3	epithelial membrane protein 3	-1.209

28461151	SLC1A5	solute carrier family 1 member 5	-1.203
157823801	SLC50A1	solute carrier family 50 member 1	-1.200
66730475	Tpm2	tropomyosin 2, beta	-1.196
564307792	TECPR2	tectonin beta-propeller repeat containing 2	-1.194
6981108	ITGB4	integrin subunit beta 4	-1.190
564320606	SEMA6A	semaphorin 6A	-1.189
300798598	MYOF	myoferlin	-1.183
82617598	SLC5A3	solute carrier family 5 member 3	-1.182
157820637	LYVE1	lymphatic vessel endothelial hyaluronan receptor 1	-1.180
293358899	ANKRD26	ankyrin repeat domain 26	-1.174
564314685	EIF4G1	eukaryotic translation initiation factor 4 gamma 1	-1.168
197313645	SMTN	smoothelin	-1.167
564326123	CDC42EP5	CDC42 effector protein 5	-1.166
6978807	EMP1	epithelial membrane protein 1	-1.164
6978773	DPP4	dipeptidyl peptidase 4	-1.161
672022611	KIAA0895	KIAA0895	-1.161
29789140	GRM6	glutamate metabotropic receptor 6	-1.152
309319796	COL18A1	collagen type XVIII alpha 1 chain	-1.151
392352101	LRCH3	leucine rich repeats and calponin homology domain containing 3	-1.150
569012000	KLF8	Kruppel like factor 8	-1.148
18777755	Slco1a4	solute carrier organic anion transporter family, member 1a4	-1.148
157821541	ACSS3	acyl-CoA synthetase short-chain family member 3	-1.145
157819945	ARPIN/C15orf38-AP3S2	C15orf38-AP3S2 readthrough	-1.140
1763306	UNC13C	unc-13 homolog C	-1.140
157818625	SLC2A12	solute carrier family 2 member 12	-1.139
149039662	LAMA2	laminin subunit alpha 2	-1.137
392332010	SLC38A10	solute carrier family 38 member 10	-1.136
148691168	N/A	N/A	-1.136
564340633	OLFML2A	olfactomedin like 2A	-1.132
71043880	COLEC12	collectin subfamily member 12	-1.130
157823857	SSPN	sarcospan	-1.129
564315959	CEP350	centrosomal protein 350	-1.126
672046728	N/A	N/A	-1.124
68163370	CARNMT1	carnosine N-methyltransferase 1	-1.123

426352061	N/A	N/A	-1.121
672088045	N/A	N/A	-1.120
149054281	CNTNAP1	contactin associated protein 1	-1.115
157818393	BICC1	BicC family RNA binding protein 1	-1.113
157820417	LIME1	Lck interacting transmembrane adaptor 1	-1.111
13929194	Sult1a1	sulfotransferase family 1A, phenol-preferring, member 1	-1.111
672025117	MBTD1	mbt domain containing 1	-1.110
149068918	RSF1	remodeling and spacing factor 1	-1.101
8393886	SLC22A6	solute carrier family 22 member 6	-1.101
293362695	Akap17b	A kinase (PRKA) anchor protein 17B	-1.099
564389875	ARHGEF10	Rho guanine nucleotide exchange factor 10	-1.098
149022338	FKBP7	FK506 binding protein 7	-1.098
672015205	N/A	N/A	-1.098
149055898	BCAT2	branched chain amino acid transaminase 2	-1.092
282158051	MYH11	myosin heavy chain 11	-1.084
564303933	TET3	tet methylcytosine dioxygenase 3	-1.084
295391840	LOC100361645	LRRGT00075-like	-1.080
149059246	N/A	N/A	-1.078
672062394	N/A	N/A	-1.077
197386131	Acad10	acyl-CoA dehydrogenase family, member 10	-1.076
157821459	OLFML3	olfactomedin like 3	-1.072
564303928	TET3	tet methylcytosine dioxygenase 3	-1.072
149016012	KANSL1L	KAT8 regulatory NSL complex subunit 1 like	-1.070
166157468	CLMN	calmin	-1.068
13540697	NOV	nephroblastoma overexpressed	-1.066
157819343	FNDC7	fibronectin type III domain containing 7	-1.064
189011703	KDELR3	KDEL endoplasmic reticulum protein retention receptor 3	-1.064
149057336	ZSCAN2	zinc finger and SCAN domain containing 2	-1.063
672052120	RBM12B	RNA binding motif protein 12B	-1.062
672081701	N/A	N/A	-1.061
672023109	COL12A1	collagen type XII alpha 1 chain	-1.060
672020846	N/A	N/A	-1.060
300796557	PIH1D2	PIH1 domain containing 2	-1.059
197386139	SSC5D	scavenger receptor cysteine rich family member with 5 domains	-1.058
674082951	N/A	N/A	-1.054
57528269	ABHD14A	abhydrolase domain containing 14A	-1.051

63706033	Gm5174 (includes others)	serine/threonine kinase, pseudogene 1	-1.051
8392864	ADORA2B	adenosine A2b receptor	-1.050
568972854	N/A	N/A	-1.050
197927252	SHQ1	SHQ1, H/ACA ribonucleoprotein assembly factor	-1.049
281332117	TGFBI	transforming growth factor beta induced	-1.048
564345556	CROT	carnitine O-octanoyltransferase	-1.045
311771704	C15orf65	chromosome 15 open reading frame 65	-1.044
149016584	ZNF606	zinc finger protein 606	-1.044
672056365	KIAA0586	KIAA0586	-1.041
672019578	MYSM1	Myb like, SWIRM and MPN domains 1	-1.040
672063750	N/A	N/A	-1.039
564296997	ZNF45	zinc finger protein 45	-1.038
672027054	N/A	N/A	-1.037
401461786	CP	ceruloplasmin	-1.035
564309732	IGSF9B	immunoglobulin superfamily member 9B	-1.035
158508544	DDR2	discoidin domain receptor tyrosine kinase 2	-1.033
148665664	PHLDB2	pleckstrin homology like domain family B member 2	-1.030
564308822	SLC45A4	solute carrier family 45 member 4	-1.029
157819321	GSDMA	gasdermin A	-1.025
392351290	DNAH9	dynein axonemal heavy chain 9	-1.024
569009290	TENM1	teneurin transmembrane protein 1	-1.024
7549765	HK2	hexokinase 2	-1.021
149059823	N/A	N/A	-1.021
113206040	LRRC34	leucine rich repeat containing 34	-1.020
56090485	M6PR	mannose-6-phosphate receptor, cation dependent	-1.019
564322493	RPGR	retinitis pigmentosa GTPase regulator	-1.018
29789038	BMP6	bone morphogenetic protein 6	-1.017
564339943	PHYHD1	phytanoyl-CoA dioxygenase domain containing 1	-1.017
564300507	SH3D19	SH3 domain containing 19	-1.017
157824136	Zic3	Zic family member 3	-1.017
62078617	PPP1R36	protein phosphatase 1 regulatory subunit 36	-1.016
157817334	Tmem198b	transmembrane protein 198b	-1.016
149048116	KIAA0907	KIAA0907	-1.013
113951677	LPAR6	lysophosphatidic acid receptor 6	-1.012
880855761	N/A	N/A	-1.010

672055261	N/A	N/A	-1.010
109471717	SAMD9	sterile alpha motif domain containing 9	-1.008
655832893	N/A	N/A	-1.005
26380608	KCNE5	potassium voltage-gated channel subfamily E regulatory subunit 5	-0.998
28461161	LDLR	low density lipoprotein receptor	-0.995
62339302	Pcdhb8	protocadherin beta 8	-0.992
9845234	ANXA2	annexin A2	-0.989
149038394	N/A	N/A	-0.989
46358064	AURKA	aurora kinase A	-0.987
149044576	N/A	N/A	-0.984
568977750	DOCK4	dedicator of cytokinesis 4	-0.983
564393142	WDR36	WD repeat domain 36	-0.983
916043983	Baiap3	BAI1-associated protein 3	-0.982
58865948	CREB3L2	cAMP responsive element binding protein 3 like 2	-0.980
164448680	HBB	hemoglobin subunit beta	-0.978
768711606	THBS3	thrombospondin 3	-0.977
55742709	ELN	elastin	-0.976
672024549	SLX4	SLX4 structure-specific endonuclease subunit	-0.975
78486570	ARHGAP4	Rho GTPase activating protein 4	-0.973
74180977	MYH9	myosin heavy chain 9	-0.972
18959222	SCN9A	sodium voltage-gated channel alpha subunit 9	-0.968
281427229	COL6A2	collagen type VI alpha 2 chain	-0.965
672059136	N/A	N/A	-0.964
149053793	TSPOAP1	TSPO associated protein 1	-0.963
300798165	ZBTB40	zinc finger and BTB domain containing 40	-0.963
685536628	N/A	N/A	-0.963
311771720	DGKQ	diacylglycerol kinase theta	-0.962
157822349	SLC52A2	solute carrier family 52 member 2	-0.960
672063748	N/A	N/A	-0.959
33286888	GJA1	gap junction protein alpha 1	-0.955
187469467	SMPD5	sphingomyelin phosphodiesterase 5	-0.955
300795183	SNTG1	syntrophin gamma 1	-0.955
795568671	N/A	N/A	-0.953
48675865	PDP2	pyruvate dehyrogenase phosphatase catalytic subunit 2	-0.952
564342055	Apip	APAF1 interacting protein	-0.951
149056134	Dzf17	zinc finger protein 17	-0.951
114145565	GALNS	galactosamine (N-acetyl)-6-sulfatase	-0.951

672082323	N/A	N/A	-0.950
300794555	TMC7	transmembrane channel like 7	-0.949
312836829	ADAMTS12	ADAM metallopeptidase with thrombospondin type 1 motif 12	-0.947
55742713	ECM1	extracellular matrix protein 1	-0.946
19424190	CX3CR1	C-X3-C motif chemokine receptor 1	-0.945
6981306	TNFRSF11B	TNF receptor superfamily member 11b	-0.944
62078689	CYYR1	cysteine and tyrosine rich 1	-0.943
157817670	SLC2A10	solute carrier family 2 member 10	-0.943
672062667	N/A	N/A	-0.942
60360628	ATP8A1	ATPase phospholipid transporting 8A1	-0.941
149047034	N/A	N/A	-0.941
672012920	N/A	N/A	-0.941
19424254	ANGPTL2	angiopoietin like 2	-0.937
564297371	LOC102556967	zinc finger protein 484-like	-0.937
672064415	LOC103693202	uncharacterized LOC103693202	-0.936
564380038	FBRSL1	fibrosin like 1	-0.935
672051145	PARP11	poly(ADP-ribose) polymerase family member 11	-0.935
9506953	PCOLCE	procollagen C-endopeptidase enhancer	-0.935
42476287	TGM2	transglutaminase 2	-0.935
672080024	N/A	N/A	-0.935
67078530	CHAF1B	chromatin assembly factor 1 subunit B	-0.934
157821893	SDF2L1	stromal cell derived factor 2 like 1	-0.934
157820317	STXBP4	syntaxin binding protein 4	-0.932
157822365	LAMC3	laminin subunit gamma 3	-0.930
564367862	Dst	dystonin	-0.928
672063869	MGC116197 (includes others)	similar to RIKEN cDNA 1700001E04	-0.928
68163507	LOC100912029 (includes others)	similar to DNA segment, Chr 19, Brigham & Womens Genetics 1357 expressed	-0.927
931568101	N/A	N/A	-0.926
8392983	BGN	biglycan	-0.924
13928758	CTSK	cathepsin K	-0.923
13591971	HNMT	histamine N-methyltransferase	-0.922
564310195	ANKDD1A	ankyrin repeat and death domain containing 1A	-0.919
31324556	RDH10	retinol dehydrogenase 10	-0.919
672051730	N/A	N/A	-0.919
685536628	N/A	N/A	-0.918

564369844	NEWGENE_1308624	sialidase 4	-0.916
164519095	SLC9A2	solute carrier family 9 member A2	-0.914
13929156	MYBPH	myosin binding protein H	-0.913
344250492	N/A	N/A	-0.913
564318923	WDR17	WD repeat domain 17	-0.909
449279816	N/A	N/A	-0.906
13592057	RPL18	ribosomal protein L18	-0.905
256773236	DNAH1	dynein axonemal heavy chain 1	-0.904
149061916	NUDT8	nudix hydrolase 8	-0.904
149031942	N/A	N/A	-0.904
157786850	TUBD1	tubulin delta 1	-0.903
564311681	PIKFYVE	phosphoinositide kinase, FYVE-type zinc finger containing	-0.901
672087260	N/A	N/A	-0.899
198442871	KANK1	KN motif and ankyrin repeat domains 1	-0.897
392355027	TANGO6	transport and golgi organization 6 homolog	-0.894
149034450	N/A	N/A	-0.892
38454234	COL27A1	collagen type XXVII alpha 1 chain	-0.891
672060362	ELFN2	extracellular leucine rich repeat and fibronectin type III domain containing 2	-0.890
57114344	UHRF1	ubiquitin like with PHD and ring finger domains 1	-0.886
880876474	N/A	N/A	-0.885
586908220	ARHGAP44	Rho GTPase activating protein 44	-0.884
305682588	PDZD7	PDZ domain containing 7	-0.882
402744047	SLC25A18	solute carrier family 25 member 18	-0.881
8393469	S1PR2	sphingosine-1-phosphate receptor 2	-0.880
672031392	N/A	N/A	-0.879
392340959	ITSN2	intersectin 2	-0.878
62078663	Mmgt2	membrane magnesium transporter 2	-0.878
149068766	PLEKHB1	pleckstrin homology domain containing B1	-0.877
149031998	ACVRL1	activin A receptor like type 1	-0.876
13591914	ANPEP	alanyl aminopeptidase, membrane	-0.875
13162359	CYBA	cytochrome b-245 alpha chain	-0.874
672087474	REPS2	RALBP1 associated Eps domain containing 2	-0.873
672087474	REPS2	RALBP1 associated Eps domain containing 2	-0.873
564364327	CA12	carbonic anhydrase 12	-0.869
157823627	MFSD12	major facilitator superfamily domain containing 12	-0.868

6754808	NDP	NDP, norrin cystine knot growth factor	-0.867
672087260	N/A	N/A	-0.867
478732983	MAP3K5	mitogen-activated protein kinase kinase kinase 5	-0.865
13786192	APLNR	apelin receptor	-0.864
672086986	SLC38A5	solute carrier family 38 member 5	-0.864
564350006	PREX2	phosphatidylinositol-3,4,5-trisphosphate dependent Rac exchange factor 2	-0.862
149065838	N/A	N/A	-0.862
148747412	CBWD1	COBW domain containing 1	-0.861
76159291	CAST	calpastatin	-0.860
149034989	RADIL	Rap associating with DIL domain	-0.859
672012705	SYNE1	spectrin repeat containing nuclear envelope protein 1	-0.859
293353154	TBC1D1	TBC1 domain family member 1	-0.859
672069572	KANSL1	KAT8 regulatory NSL complex subunit 1	-0.858
157820241	Marveld1	MARVEL domain containing 1	-0.858
564312944	KIAA0753	KIAA0753	-0.857
408407614	DNA2	DNA replication helicase/nuclease 2	-0.853
672064676	N/A	N/A	-0.853
568940286	BRAF	B-Raf proto-oncogene, serine/threonine kinase	-0.852
6978703	CPT1B	carnitine palmitoyltransferase 1B	-0.851
406719604	SLC9A9	solute carrier family 9 member A9	-0.851
392338379	SLC26A8	solute carrier family 26 member 8	-0.848
75832132	ESYT1	extended synaptotagmin 1	-0.844
564380929	KCNT2	potassium sodium-activated channel subfamily T member 2	-0.844
755566692	HUWE1	HECT, UBA and WWE domain containing 1, E3 ubiquitin protein ligase	-0.837
564378315	Zfp853	zinc finger protein 853	-0.837
6754928	OSR1	odd-skipped related transciption factor 1	-0.835
20302097	PIGL	phosphatidylinositol glycan anchor biosynthesis class L	-0.835
149034207	ITIH3	inter-alpha-trypsin inhibitor heavy chain 3	-0.834
11177892	KCNT1	potassium sodium-activated channel subfamily T member 1	-0.833
25742772	KCNA2	potassium voltage-gated channel subfamily A member 2	-0.832
564387543	UGGT2	UDP-glucose glycoprotein glucosyltransferase 2	-0.831
157820681	DLL4	delta like canonical Notch ligand 4	-0.826

157818909	Zim1	zinc finger, imprinted 1	-0.825
157821487	ANKRD34B	ankyrin repeat domain 34B	-0.824
149053566	N/A	N/A	-0.824
157822353	Hells/LOC100911660	helicase, lymphoid specific	-0.823
564320335	TMEM241	transmembrane protein 241	-0.823
815891318	ENTPD1	ectonucleoside triphosphate diphosphohydrolase 1	-0.822
183986528	ZNF692	zinc finger protein 692	-0.822
148695091	BBS5	Bardet-Biedl syndrome 5	-0.821
109509239	RRP1B	ribosomal RNA processing 1B	-0.820
672073969	CLASP1	cytoplasmic linker associated protein 1	-0.817
187282311	ISLR	immunoglobulin superfamily containing leucine rich repeat	-0.817
77861917	CFH	complement factor H	-0.816
226698394	UNC80	unc-80 homolog, NALCN activator	-0.814
149020543	C19orf66	chromosome 19 open reading frame 66	-0.813
1346731	HAPLN1	hyaluronan and proteoglycan link protein 1	-0.813
672062052	ITGA11	integrin subunit alpha 11	-0.813
124244050	PPIP5K1	diphosphoinositol pentakisphosphate kinase 1	-0.813
27436863	HACL1	2-hydroxyacyl-CoA lyase 1	-0.812
157818989	LRRC71	leucine rich repeat containing 71	-0.811
564394961	N/A	N/A	-0.811
6978545	ATP1A2	ATPase Na+/K+ transporting subunit alpha 2	-0.810
292781228	DECR1	2,4-dienoyl-CoA reductase 1	-0.810
149038682	Srgn	serglycin	-0.810
672080026	N/A	N/A	-0.810
209870105	GPR37L1	G protein-coupled receptor 37 like 1	-0.807
672070295	BAHCC1	BAH domain and coiled-coil containing 1	-0.800
148679862	SLC35F3	solute carrier family 35 member F3	-0.800
157818137	RECK	reversion inducing cysteine rich protein with kazal motifs	-0.798
672023790	N/A	N/A	-0.798
78486556	C16orf58	chromosome 16 open reading frame 58	-0.797
16758800	TRIP10	thyroid hormone receptor interactor 10	-0.797
149051152	N/A	N/A	-0.797
589932011	N/A	N/A	-0.796
62078751	GRAMD2B	GRAM domain containing 2B	-0.795
672013014	N/A	N/A	-0.795
166197716	PER3	period circadian clock 3	-0.794

51571903	C6orf47	chromosome 6 open reading frame 47	-0.793
564319108	ADGRA2	adhesion G protein-coupled receptor A2	-0.791
157821089	PEX10	peroxisomal biogenesis factor 10	-0.791
198041781	GLTPD2	glycolipid transfer protein domain containing 2	-0.790
564333959	STN1	STN1, CST complex subunit	-0.790
755566682	HUWE1	HECT, UBA and WWE domain containing 1, E3 ubiquitin protein ligase	-0.788
564307839	JAG2	jagged 2	-0.788
57528326	MTFMT	mitochondrial methionyl-tRNA formyltransferase	-0.787
564299019	KIAA2026	KIAA2026	-0.786
293341811	PRR14L	proline rich 14 like	-0.786
6978587	CALCRL	calcitonin receptor like receptor	-0.785
157822879	EFS	embryonal Fyn-associated substrate	-0.785
300794353	FANCL	Fanconi anemia complementation group L	-0.784
62078681	OGFOD3	2-oxoglutarate and iron dependent oxygenase domain containing 3	-0.784
164518939	SLC7A11	solute carrier family 7 member 11	-0.783
167830444	DCDC2	doublecortin domain containing 2	-0.781
67514566	POLA2	DNA polymerase alpha 2, accessory subunit	-0.780
307078146	UACA	uveal autoantigen with coiled-coil domains and ankyrin repeats	-0.780
164565435	SYNJ2	synaptojanin 2	-0.778
312922352	TTF2	transcription termination factor 2	-0.774
564296650	ZSCAN18	zinc finger and SCAN domain containing 18	-0.774
201023375	ZFAT	zinc finger and AT-hook domain containing	-0.771
55741551	CHCHD10	coiled-coil-helix-coiled-coil-helix domain containing 10	-0.770
188595660	SNX33	sorting nexin 33	-0.769
157823511	TBX18	T-box 18	-0.769
672034684	N/A	N/A	-0.769
149068170	N/A	N/A	-0.768
402745263	COL11A1	collagen type XI alpha 1 chain	-0.766
31324552	NADSYN1	NAD synthetase 1	-0.763
8393218	CTSC	cathepsin C	-0.761
19173756	ERG	ERG, ETS transcription factor	-0.761
256000825	FAM227A	family with sequence similarity 227 member A	-0.761
672041704	NIPBL	NIPBL, cohesin loading factor	-0.760
672041704	NIPBL	NIPBL, cohesin loading factor	-0.760

157821969	ARAP2	ArfGAP with RhoGAP domain, ankyrin repeat and PH domain 2	-0.757
25742733	P2RY1	purinergic receptor P2Y1	-0.757
201861690	TPK1	thiamin pyrophosphokinase 1	-0.756
564333748	N/A	N/A	-0.756
731457978	N/A	N/A	-0.755
300794574	AHNAK	AHNAK nucleoprotein	-0.754
108935976	DISC1	disrupted in schizophrenia 1	-0.754
589937133	N/A	N/A	-0.754
62945368	ITGBL1	integrin subunit beta like 1	-0.752
149044145	N/A	N/A	-0.752
157822217	ZFYVE28	zinc finger FYVE-type containing 28	-0.751
470631944	N/A	N/A	-0.751
149053435	N/A	N/A	-0.751
293344558	PCNX3	pecanex homolog 3 (Drosophila)	-0.748
672079407	CCDC66	coiled-coil domain containing 66	-0.747
149029225	N/A	N/A	-0.747
157818843	EXTL1	exostosin like glycosyltransferase 1	-0.746
18777774	KCNH7	potassium voltage-gated channel subfamily H member 7	-0.746
22129763	LOC257642	rRNA promoter binding protein	-0.743
148692627	N/A	N/A	-0.743
149061352	ADAM12	ADAM metallopeptidase domain 12	-0.742
7949020	CDK2	cyclin dependent kinase 2	-0.742
635092008	N/A	N/A	-0.742
564317714	Ktn1	kinectin 1	-0.740
564349878	KIAA1551	KIAA1551	-0.738
77993356	CDCA7L	cell division cycle associated 7 like	-0.737
18249941	CHRM1	cholinergic receptor muscarinic 1	-0.735
325530254	FAM109A	family with sequence similarity 109 member A	-0.734
197333870	NID2	nidogen 2	-0.733
564324344	LOC363306 (includes others)	hypothetical protein LOC363306	-0.731
293343546	C5orf49	chromosome 5 open reading frame 49	-0.730
13591983	LUM	lumican	-0.730
157787103	RASA2	RAS p21 protein activator 2	-0.730
564400410	AMOT	angiomotin	-0.729
564310412	DOPEY1	dopey family member 1	-0.729
672034690	N/A	N/A	-0.729
50657355	TOP1MT	topoisomerase (DNA) I, mitochondrial	-0.728

589269168	WDR34	WD repeat domain 34	-0.728
73746573	TGFB1I1	transforming growth factor beta 1 induced transcript 1	-0.724
157821021	ZC3H6	zinc finger CCCH-type containing 6	-0.724
672046728	N/A	N/A	-0.723
40254754	OCLN	occludin	-0.720
564327948	JOSD2	Josephin domain containing 2	-0.719
149028840	N/A	N/A	-0.719
672063785	N/A	N/A	-0.719
564388153	N/A	N/A	-0.719
564329859	COA4	cytochrome c oxidase assembly factor 4 homolog	-0.718
293344916	COL6A1	collagen type VI alpha 1 chain	-0.718
672022615	N/A	N/A	-0.716
564352121	LRP8	LDL receptor related protein 8	-0.715
8393057	SERPINH1	serpin family H member 1	-0.714
392355060	VAT1L	vesicle amine transport 1 like	-0.714
149054246	KCNH4	potassium voltage-gated channel subfamily H member 4	-0.712
209954806	PIGN	phosphatidylinositol glycan anchor biosynthesis class N	-0.712
564301698	LY75	lymphocyte antigen 75	-0.711
68163509	THNSL1	threonine synthase like 1	-0.711
171846640	FBLN1	fibulin 1	-0.709
157786618	RANGRF	RAN guanine nucleotide release factor	-0.707
589917689	N/A	N/A	-0.705
46310239	SIDT1	SID1 transmembrane family member 1	-0.704
672026767	N/A	N/A	-0.703
13786160	SLC22A8	solute carrier family 22 member 8	-0.700
148699893	COL6A1	collagen type VI alpha 1 chain	-0.699
112350	N/A	N/A	-0.699
576067885	AGBL3	ATP/GTP binding protein like 3	-0.698
62078635	CCDC153	coiled-coil domain containing 153	-0.698
157817903	Dcaf12l1	DDB1 and CUL4 associated factor 12-like 1	-0.698
564395631	Slc25a36l1	solute carrier family 25 (pyrimidine nucleotide carrier), member 36-like 1	-0.698
77917594	ZFYVE19	zinc finger FYVE-type containing 19	-0.698
392332443	PRKDC	protein kinase, DNA-activated, catalytic polypeptide	-0.697
53850640	MAVS	mitochondrial antiviral signaling protein	-0.696

46402488	NOS3	nitric oxide synthase 3	-0.696
149043511	N/A	N/A	-0.696
568917022	DZANK1	double zinc ribbon and ankyrin repeat domains 1	-0.695
74184716	Kat6b	K(lysine) acetyltransferase 6B	-0.694
157823373	TRHDE	thyrotropin releasing hormone degrading enzyme	-0.694
201066348	PEAR1	platelet endothelial aggregation receptor 1	-0.693
149048177	RRNAD1	ribosomal RNA adenine dimethylase domain containing 1	-0.693
674048263	N/A	N/A	-0.693
199562000	USP40	ubiquitin specific peptidase 40	-0.692
189303595	RNPC3	RNA binding region (RNP1, RRM) containing 3	-0.690
407228394	ZNF397	zinc finger protein 397	-0.690
564306228	N/A	N/A	-0.690
307548437	NYAP2	neuronal tyrosine-phosphorylated phosphoinositide-3-kinase adaptor 2	-0.689
149039207	COL5A1	collagen type V alpha 1 chain	-0.687
76257398	Cdkn1c	cyclin-dependent kinase inhibitor 1C (P57)	-0.686
293342292	FAM208A	family with sequence similarity 208 member A	-0.686
672085293	FANCA	Fanconi anemia complementation group A	-0.686
157822819	PNP	purine nucleoside phosphorylase	-0.686
672055092	N/A	N/A	-0.686
16758622	IFT172	intraflagellar transport 172	-0.684
238859561	DCTD	dCMP deaminase	-0.683
149038013	SLC9A5	solute carrier family 9 member A5	-0.681
657940868	PALLD	palladin, cytoskeletal associated protein	-0.680
12831205	EPAS1	endothelial PAS domain protein 1	-0.679
308044487	KIAA0319	KIAA0319	-0.679
293347888	SRBD1	S1 RNA binding domain 1	-0.679
564305934	BTBD19	BTB domain containing 19	-0.678
6978505	ANXA5	annexin A5	-0.677
157821335	GPR162	G protein-coupled receptor 162	-0.677
568975606	SLC16A13	solute carrier family 16 member 13	-0.677
19705485	SULF1	sulfatase 1	-0.677
564314522	LRCH3	leucine rich repeats and calponin homology domain containing 3	-0.676
294979146	TCTN1	tectonic family member 1	-0.676
148698795	GPX7	glutathione peroxidase 7	-0.675

83642834	NAGK	N-acetylglucosamine kinase	-0.675
672079010	N/A	N/A	-0.675
148699227	MYADM	myeloid associated differentiation marker	-0.672
149053435	N/A	N/A	-0.670
71043696	DUSP11	dual specificity phosphatase 11	-0.668
9507107	SLC12A4	solute carrier family 12 member 4	-0.668
300388140	MYLK	myosin light chain kinase	-0.667
815891112	AHR	aryl hydrocarbon receptor	-0.666
172045714	MIIP	migration and invasion inhibitory protein	-0.666
392340768	DISP3	dispatched RND transporter family member 3	-0.664
148690852	FCGRT	Fc fragment of IgG receptor and transporter	-0.664
157817600	SMARCAL1	SWI/SNF related, matrix associated, actin dependent regulator of chromatin, subfamily a like 1	-0.664
672057459	DGKA	diacylglycerol kinase alpha	-0.663
398650618	MMP11	matrix metallopeptidase 11	-0.663
149034958	ZNF12	zinc finger protein 12	-0.663
564305252	FBXO10	F-box protein 10	-0.662
697012013	N/A	N/A	-0.662
403310668	FOXC1	forkhead box C1	-0.661
569000267	MDC1	mediator of DNA damage checkpoint 1	-0.661
149034401	TLE2	transducin like enhancer of split 2	-0.661
149053793	TSPOAP1	TSPO associated protein 1	-0.661
672027054	N/A	N/A	-0.661
149054665	ABCA9	ATP binding cassette subfamily A member 9	-0.660
164607119	SUMF2	sulfatase modifying factor 2	-0.659
23463307	RIOX2	ribosomal oxygenase 2	-0.658
672023055	TLN2	talin 2	-0.658
564296997	ZNF45	zinc finger protein 45	-0.658
404247433	SVIL	supervillin	-0.654
392340509	PTPRD	protein tyrosine phosphatase, receptor type D	-0.652
392346344	N/A	N/A	-0.652
57164109	KRCC1	lysine rich coiled-coil 1	-0.650
281599331	ZKSCAN8	zinc finger with KRAB and SCAN domains 8	-0.650
242397450	PCSK6	proprotein convertase subtilisin/kexin type 6	-0.649
6978737	CYP1B1	cytochrome P450 family 1 subfamily B member 1	-0.648
392334509	N/A	N/A	-0.648
149731778	N/A	N/A	-0.648
6681095	CYCS	cytochrome c, somatic	-0.647

157819753	RCN1	reticulocalbin 1	-0.647
148680122	UNC5C	unc-5 netrin receptor C	-0.647
58219539	ENG	endoglin	-0.646
25282463	CDC20	cell division cycle 20	-0.645
68341969	CDKL1	cyclin dependent kinase like 1	-0.645
157823279	CGNL1	cingulin like 1	-0.645
564357839	PAN2	PAN2 poly(A) specific ribonuclease subunit	-0.644
81158091	PCDHGA9	protocadherin gamma subfamily A, 9	-0.639
16923978	SLC26A2	solute carrier family 26 member 2	-0.639
564330609	SYT17	synaptotagmin 17	-0.639
672066075	COL6A3	collagen type VI alpha 3 chain	-0.638
392341425	PTPRB	protein tyrosine phosphatase, receptor type B	-0.637
57528264	MCCC1	methylcrotonoyl-CoA carboxylase 1	-0.636
568954992	PALLD	palladin, cytoskeletal associated protein	-0.636
655886694	N/A	N/A	-0.635
75905809	AKAP12	A-kinase anchoring protein 12	-0.634
31077144	CAND2	cullin associated and neddylation dissociated 2 (putative)	-0.634
8394446	TGFBR3	transforming growth factor beta receptor 3	-0.634
672023055	TLN2	talin 2	-0.634
13929062	GGCX	gamma-glutamyl carboxylase	-0.632
74218228	HNRNPC	heterogeneous nuclear ribonucleoprotein C (C1/C2)	-0.631
400153797	PLOD1	procollagen-lysine,2-oxoglutarate 5-dioxygenase 1	-0.631
568930638	HSPG2	heparan sulfate proteoglycan 2	-0.630
21728394	KLHL17	kelch like family member 17	-0.630
7106349	LYNX1	Ly6/neurotoxin 1	-0.629
18959236	PECR	peroxisomal trans-2-enoyl-CoA reductase	-0.629
880939564	N/A	N/A	-0.629
149057830	Hgsnat	heparan-alpha-glucosaminide N-acetyltransferase	-0.628
148667878	IGFBP5	insulin like growth factor binding protein 5	-0.628
672073977	LOC103690089	pleckstrin homology domain-containing family A member 6-like	-0.628
61557127	NNT	nicotinamide nucleotide transhydrogenase	-0.627
564311685	PIKFYVE	phosphoinositide kinase, FYVE-type zinc finger containing	-0.627
149064065	ZMYM5	zinc finger MYM-type containing 5	-0.627
158081747	PDGFB	platelet derived growth factor subunit B	-0.626

149036738	Slc41a3	solute carrier family 41, member 3	-0.626
672047353	RALGAPA2	Ral GTPase activating protein catalytic alpha subunit 2	-0.625
157819207	BCL6	B-cell CLL/lymphoma 6	-0.623
197927123	LYRM7	LYR motif containing 7	-0.622
16758928	RGCC	regulator of cell cycle	-0.622
58865848	PLSCR3	phospholipid scramblase 3	-0.619
564323252	KLHL13	kelch like family member 13	-0.618
148698920	N/A	N/A	-0.618
77695926	STAT1	signal transducer and activator of transcription 1	-0.617
157786694	CAVIN1	caveolae associated protein 1	-0.616
157787030	DISP1	dispatched RND transporter family member 1	-0.616
11024664	LTBP1	latent transforming growth factor beta binding protein 1	-0.616
392340179	RERG	RAS like estrogen regulated growth inhibitor	-0.616
61097937	VEGFB	vascular endothelial growth factor B	-0.616
564369812	D2HGDH	D-2-hydroxyglutarate dehydrogenase	-0.615
68534736	ERAP1	endoplasmic reticulum aminopeptidase 1	-0.615
157786690	PRKCA	protein kinase C alpha	-0.614
6981128	KDR	kinase insert domain receptor	-0.613
564391229	SERPINB6	serpin family B member 6	-0.613
537179361	N/A	N/A	-0.613
672047351	RALGAPA2	Ral GTPase activating protein catalytic alpha subunit 2	-0.611
312147379	LAMA1	laminin subunit alpha 1	-0.610
56119147	ARRDC3	arrestin domain containing 3	-0.609
115392004	GPR17	G protein-coupled receptor 17	-0.609
672063869	MGC116197 (includes others)	similar to RIKEN cDNA 1700001E04	-0.609
149054795	RGD1309310	similar to mKIAA0195 protein	-0.609
157816939	WASHC3	WASH complex subunit 3	-0.609
194474072	RRM2B	ribonucleotide reductase regulatory TP53 inducible subunit M2B	-0.607
568956384	ADAMTS18	ADAM metallopeptidase with thrombospondin type 1 motif 18	-0.604
253683447	ETV1	ETS variant 1	-0.604
83025052	ANKS6	ankyrin repeat and sterile alpha motif domain containing 6	-0.603
402478640	HTRA3	HtrA serine peptidase 3	-0.603

27720599	PLEKHO2	pleckstrin homology domain containing O2	-0.603
58865984	TRAF3IP1	TRAF3 interacting protein 1	-0.603
672069567	N/A	N/A	-0.603
25453410	CACNA1B	calcium voltage-gated channel subunit alpha1 B	-0.602
672014912	SORBS1	sorbin and SH3 domain containing 1	-0.602
293348472	ZFR2	zinc finger RNA binding protein 2	-0.602
293348472	ZFR2	zinc finger RNA binding protein 2	-0.602
564301426	CNTRL	centriolin	-0.601
198278450	CPT1C	carnitine palmitoyltransferase 1C	-0.601
157824134	DENND6B	DENN domain containing 6B	-0.601
672053977	KCNQ4	potassium voltage-gated channel subfamily Q member 4	-0.601
815891336	TCN2	transcobalamin 2	-0.601
672054770	LOC500584	similar to casein kinase 1, gamma 3 isoform 2	-0.600
672034032	N/A	N/A	-0.600
189163483	PCK2	phosphoenolpyruvate carboxykinase 2, mitochondrial	-0.599
392339261	PKP4	plakophilin 4	-0.599
50510463	PRUNE2	prune homolog 2	-0.599
594075460	N/A	N/A	-0.599
293356488	RIC1	RIC1 homolog, RAB6A GEF complex partner 1	-0.598
294610780	SYNE2	spectrin repeat containing nuclear envelope protein 2	-0.598
18959240	ADARB2	adenosine deaminase, RNA specific B2 (inactive)	-0.597
56090457	ARMCX6	armadillo repeat containing, X-linked 6	-0.597
672041794	RGD1310081	similar to hypothetical protein FLJ13231	-0.597
148747194	SLC16A7	solute carrier family 16 member 7	-0.597
537229522	N/A	N/A	-0.597
426352465	N/A	N/A	-0.597
157821557	CD248	CD248 molecule	-0.595
12018250	TEP1	telomerase associated protein 1	-0.594
121583772	BBX	BBX, HMG-box containing	-0.593
399220341	SLC2A13	solute carrier family 2 member 13	-0.593
16758186	SLCO1C1	solute carrier organic anion transporter family member 1C1	-0.593
148697042	N/A	N/A	-0.593
149025910	NEUROG2	neurogenin 2	-0.592
300797349	EBF4	early B-cell factor 4	-0.590

149046383	Lman2l	lectin, mannose-binding 2-like	-0.589
13562118	LRP2	LDL receptor related protein 2	-0.589
564355112	EMILIN1	elastin microfibril interfacer 1	-0.588
13928736	AMPD3	adenosine monophosphate deaminase 3	-0.587
148692356	ARHGEF1	Rho guanine nucleotide exchange factor 1	-0.587
564297850	CRTC3	CREB regulated transcription coactivator 3	-0.587
564367529	ENPP4	ectonucleotide pyrophosphatase/phosphodiesterase 4 (putative)	-0.587
157786874	Fmnl1	formin-like 1	-0.587
966948818	N/A	N/A	-0.587
672044191	TBCK	TBC1 domain containing kinase	-0.585
109490297	ABCA3	ATP binding cassette subfamily A member 3	-0.584
3157995	DAB2	DAB2, clathrin adaptor protein	-0.584
11067409	NEGR1	neuronal growth regulator 1	-0.584
18426846	DCBLD2	discoidin, CUB and LCCL domain containing 2	-0.583
157817498	GLDC	glycine decarboxylase	-0.583
348041395	DLGAP2	DLG associated protein 2	-0.582
149028753	N/A	N/A	-0.582
157824146	ITGA5	integrin subunit alpha 5	-0.581
7542357	QKI	QKI, KH domain containing RNA binding	-0.581
157822461	C20orf194	chromosome 20 open reading frame 194	-0.579
404247454	COL26A1	collagen type XXVI alpha 1 chain	-0.579
132566529	DIO2	iodothyronine deiodinase 2	-0.579
672073723	GLI2	GLI family zinc finger 2	-0.578
6981142	LAMB2	laminin subunit beta 2	-0.578
124487354	TAF4	TATA-box binding protein associated factor 4	-0.578
76443683	LOC100912042/Surf2	surfeit 2	-0.577
50510855	RIMKLB	ribosomal modification protein rimK like family member B	-0.576
157818491	DUS2	dihydrouridine synthase 2	-0.575
78042613	NICN1	nicolin 1	-0.575
124286858	B230217C12Rik	RIKEN cDNA B230217C12 gene	-0.573
148669431	DNAJC27	DnaJ heat shock protein family (Hsp40) member C27	-0.573
300797038	HHIP	hedgehog interacting protein	-0.573
157821393	LRRC20	leucine rich repeat containing 20	-0.573
625292335	N/A	N/A	-0.572

672064584	DAAM2	dishevelled associated activator of morphogenesis 2	-0.571
77993368	ACSF2	acyl-CoA synthetase family member 2	-0.570
564302153	BAHD1	bromo adjacent homology domain containing 1	-0.569
68341971	MINDY1	MINDY lysine 48 deubiquitinase 1	-0.569
564361876	RAPGEF3	Rap guanine nucleotide exchange factor 3	-0.569
384368019	Snhg11	small nucleolar RNA host gene 11	-0.569
939319594	CPNE7	copine 7	-0.568
532008265	N/A	N/A	-0.568
61557118	PCGF6	polycomb group ring finger 6	-0.567
589965307	N/A	N/A	-0.567
529367218	Abca8a	ATP-binding cassette, sub-family A (ABC1), member 8a	-0.566
68163523	TTC26	tetratricopeptide repeat domain 26	-0.566
50054384	CSF1	colony stimulating factor 1	-0.565
149059979	KLHL13	kelch like family member 13	-0.565
537137169	N/A	N/A	-0.565
149046941	BEND3	BEN domain containing 3	-0.564
564382262	Ptpn14	protein tyrosine phosphatase, non-receptor type 14	-0.564
672017461	Rrbp1	ribosome binding protein 1	-0.564
635141277	N/A	N/A	-0.563
127140886	EML6	echinoderm microtubule associated protein like 6	-0.562
71043706	MUS81	MUS81 structure-specific endonuclease subunit	-0.562
157819569	TEAD2	TEA domain transcription factor 2	-0.562
815891312	CACNA1G	calcium voltage-gated channel subunit alpha1 G	-0.561
564342470	MGA	MGA, MAX dimerization protein	-0.560
16758138	POMT1	protein O-mannosyltransferase 1	-0.560
157823283	Coch	cochlin	-0.559
564352420	MKNK1	MAP kinase interacting serine/threonine kinase 1	-0.558
564310904	N/A	N/A	-0.558
568916013	N/A	N/A	-0.557
358030320	DMTN	dematin actin binding protein	-0.556
672014089	N/A	N/A	-0.556
6981008	HAS2	hyaluronan synthase 2	-0.555
149038513	SYNE1	spectrin repeat containing nuclear envelope protein 1	-0.555
219282643	Zfp61	zinc finger protein 61	-0.555

374253863	CPNE2	copine 2	-0.554
672014740	MAMDC2	MAM domain containing 2	-0.554
9507045	RGS5	regulator of G protein signaling 5	-0.554
954470796	N/A	N/A	-0.554
55741859	XRCC4	X-ray repair cross complementing 4	-0.552
594191048	C19orf54	chromosome 19 open reading frame 54	-0.551
293349337	COL12A1	collagen type XII alpha 1 chain	-0.551
16758716	CACNB2	calcium voltage-gated channel auxiliary subunit beta 2	-0.550
818015	HBB	hemoglobin subunit beta	-0.550
149049048	RECQL	RecQ like helicase	-0.550
167900441	FOXK1	forkhead box K1	-0.549
149065851	XPNPEP3	X-prolyl aminopeptidase 3	-0.549
149060466	ZBTB20	zinc finger and BTB domain containing 20	-0.549
71043616	CDCA7	cell division cycle associated 7	-0.548
148690851	RCN3	reticulocalbin 3	-0.548
568950242	Pgap2	post-GPI attachment to proteins 2	-0.547
10242377	GRIK4	glutamate ionotropic receptor kainate type subunit 4	-0.546
157822743	KIF20A	kinesin family member 20A	-0.545
9437326	SLC4A4	solute carrier family 4 member 4	-0.544
157786876	ELFN1	extracellular leucine rich repeat and fibronectin type III domain containing 1	-0.543
148686921	SLC24A4	solute carrier family 24 member 4	-0.543
755783452	N/A	N/A	-0.543
344250708	N/A	N/A	-0.543
672058561	N/A	N/A	-0.542
45478182	N/A	N/A	-0.542
564329392	FLNA	filamin A	-0.541
109488672	SLFN5	schlafen family member 5	-0.541
18104933	FMOD	fibromodulin	-0.540
564360472	OPLAH	5-oxoprolinase (ATP-hydrolysing)	-0.540
45478072	N/A	N/A	-0.540
564318054	R3hcc1	R3H domain and coiled-coil containing 1	-0.539
62078469	RSPH10B/RSPH10B2	radial spoke head 10 homolog B	-0.539
219278723	ZNF23	zinc finger protein 23	-0.539
817307535	N/A	N/A	-0.539
625254629	N/A	N/A	-0.538
281604225	PUS7	pseudouridylate synthase 7 (putative)	-0.537

6978867	GABRB1	gamma-aminobutyric acid type A receptor beta1 subunit	-0.536
149041357	RGD1311744	similar to RIKEN cDNA 5830475I06	-0.536
33414515	PXK	PX domain containing serine/threonine kinase like	-0.535
33086478	N/A	N/A	-0.535
300797651	FOXO1	forkhead box O1	-0.534
16758486	PHYH	phytanoyl-CoA 2-hydroxylase	-0.534
39930495	PTCH1	patched 1	-0.534
157819687	TUBGCP5	tubulin gamma complex associated protein 5	-0.534
29293811	SERPINF1	serpin family F member 1	-0.533
392331829	ATAD5	ATPase family, AAA domain containing 5	-0.531
672028474	CDH24	cadherin 24	-0.531
110347559	PCDHA13	protocadherin alpha 13	-0.531
6981664	TNFRSF1A	TNF receptor superfamily member 1A	-0.530
210032529	IQGAP1	IQ motif containing GTPase activating protein 1	-0.529
13591981	LSS	lanosterol synthase	-0.529
149028178	RANBP3	RAN binding protein 3	-0.529
157817704	TRIM36	tripartite motif containing 36	-0.529
109475601	GPATCH3	G-patch domain containing 3	-0.528
157824208	NTNG1	netrin G1	-0.528
300797330	PTPRU	protein tyrosine phosphatase, receptor type U	-0.528
281604221	POLA1	DNA polymerase alpha 1, catalytic subunit	-0.527
149024348	RAP1GAP	RAP1 GTPase activating protein	-0.527
109484871	HERC1	HECT and RLD domain containing E3 ubiquitin protein ligase family member 1	-0.526
568972691	STAT5B	signal transducer and activator of transcription 5B	-0.526
564302924	TSHZ2	teashirt zinc finger homeobox 2	-0.526
312922377	POLE	DNA polymerase epsilon, catalytic subunit	-0.525
755520134	N/A	N/A	-0.525
13994179	SLC24A2	solute carrier family 24 member 2	-0.524
392341019	TOGARAM1	TOG array regulator of axonemal microtubules 1	-0.524
50511215	TTC14	tetratricopeptide repeat domain 14	-0.524
383087738	TYK2	tyrosine kinase 2	-0.524
564323057	ARMCX4	armadillo repeat containing, X-linked 4	-0.523
589954679	N/A	N/A	-0.523
46485403	CHST7	carbohydrate sulfotransferase 7	-0.522

157823399	COG4	component of oligomeric golgi complex 4	-0.522
124107592	MYO1C	myosin IC	-0.522
78187981	TRPV2	transient receptor potential cation channel subfamily V member 2	-0.522
62079077	ALG14	ALG14, UDP-N-acetylglucosaminyltransferase subunit	-0.521
405778354	NKD1	naked cuticle homolog 1	-0.521
672057084	N/A	N/A	-0.521
157820275	OCEL1	occludin/ELL domain containing 1	-0.520
392338478	TTC37	tetratricopeptide repeat domain 37	-0.520
219804406	DOCK1	dedicator of cytokinesis 1	-0.519
28972652	SLC12A5	solute carrier family 12 member 5	-0.519
2773162	ABCC8	ATP binding cassette subfamily C member 8	-0.515
197313711	PALD1	phosphatase domain containing, paladin 1	-0.515
149016805	PSD3	pleckstrin and Sec7 domain containing 3	-0.515
109460492	Arfgef3	ARFGEF family member 3	-0.514
984104888	N/A	N/A	-0.514
672043357	ARNT	aryl hydrocarbon receptor nuclear translocator	-0.513
148702301	CYB561	cytochrome b561	-0.513
18959266	KHDRBS2	KH RNA binding domain containing, signal transduction associated 2	-0.513
564371892	N/A	N/A	-0.513
148697713	CACNG2	calcium voltage-gated channel auxiliary subunit gamma 2	-0.512
71361669	CIT	citron rho-interacting serine/threonine kinase	-0.512
58865750	ZC3H8	zinc finger CCCH-type containing 8	-0.512
149057193	N/A	N/A	-0.512
71043764	C20orf27	chromosome 20 open reading frame 27	-0.510
157822913	LHFPL2	lipoma HMGIC fusion partner-like 2	-0.510
157818983	SIRT7	sirtuin 7	-0.509
13929166	CLIC4	chloride intracellular channel 4	-0.508
148698628	HECTD3	HECT domain E3 ubiquitin protein ligase 3	-0.508
157817743	CDH5	cadherin 5	-0.507
149049654	CXCL12	C-X-C motif chemokine ligand 12	-0.507
149032830	CCDC127	coiled-coil domain containing 127	-0.505
55741540	KATNAL1	katanin catalytic subunit A1 like 1	-0.505
564317005	TBC1D1	TBC1 domain family member 1	-0.505
149061998	N/A	N/A	-0.505
564300485	LOC102551095	uncharacterized LOC102551095	-0.504
149067993	RBBP6	RB binding protein 6, ubiquitin ligase	-0.503

149022245	SCRN3	secernin 3	-0.503
564365342	N/A	N/A	-0.503
149025408	N/A	N/A	-0.503
392343941	ZC3H4	zinc finger CCCH-type containing 4	-0.502
58865380	STAT2	signal transducer and activator of transcription 2	-0.501
675781130	N/A	N/A	-0.501
672035060	CIC	capicua transcriptional repressor	-0.499
564304076	FGD5	FYVE, RhoGEF and PH domain containing 5	-0.499
281332137	KIF11	kinesin family member 11	-0.499
293352381	PAN3	PAN3 poly(A) specific ribonuclease subunit	-0.499
564339225	N/A	N/A	-0.499
672026392	PNPLA6	patatin like phospholipase domain containing 6	-0.497
149029577	N/A	N/A	-0.497
564367481	TMEM63B	transmembrane protein 63B	-0.496
78214350	COQ9	coenzyme Q9	-0.495
392350322	DNAJC13	DnaJ heat shock protein family (Hsp40) member C13	-0.495
672016550	RAPGEF1	Rap guanine nucleotide exchange factor 1	-0.495
403420604	PCDH11X	protocadherin 11 X-linked	-0.494
913508404	N/A	N/A	-0.494
13027400	GUCY1A2	guanylate cyclase 1 soluble subunit alpha 2	-0.493
149052198	NPRL3	NPR3 like, GATOR1 complex subunit	-0.493
148689279	RBM5	RNA binding motif protein 5	-0.493
149025849	ARHGAP29	Rho GTPase activating protein 29	-0.492
210033118	COG1	component of oligomeric golgi complex 1	-0.492
13591989	MLH1	mutL homolog 1	-0.492
157817205	USP45	ubiquitin specific peptidase 45	-0.492
219275534	VPS13A	vacuolar protein sorting 13 homolog A	-0.492
210032083	ATG4D	autophagy related 4D cysteine peptidase	-0.491
6978761	DGKG	diacylglycerol kinase gamma	-0.491
392338392	PCNT	pericentrin	-0.491
6978789	SPARCL1	SPARC like 1	-0.491
820994385	N/A	N/A	-0.491
157819911	CSGALNACT2	chondroitin sulfate N-acetylgalactosaminyltransferase 2	-0.490
13592007	MVK	mevalonate kinase	-0.490
392338478	TTC37	tetratricopeptide repeat domain 37	-0.490
281371499	COL5A2	collagen type V alpha 2 chain	-0.489
210032365	HSP90B1	heat shock protein 90 beta family member 1	-0.489

310772205	MAP7	microtubule associated protein 7	-0.488
67078430	METTL3	methyltransferase like 3	-0.488
564372831	N/A	N/A	-0.488
694870853	AP5Z1	adaptor related protein complex 5 zeta 1 subunit	-0.487
564323143	Gprasp2	G protein-coupled receptor associated sorting protein 2	-0.487
149016965	GRB10	growth factor receptor bound protein 10	-0.487
148699520	SLC39A3	solute carrier family 39 member 3	-0.487
300669604	ADAM15	ADAM metallopeptidase domain 15	-0.486
148679892	WLS	wntless Wnt ligand secretion mediator	-0.486
748983393	ZBTB4	zinc finger and BTB domain containing 4	-0.486
149046900	N/A	N/A	-0.485
310688881	NEK1	NIMA related kinase 1	-0.484
27545388	ABCA5	ATP binding cassette subfamily A member 5	-0.483
202070751	RFTN1	raftlin, lipid raft linker 1	-0.483
86129590	TES	testin LIM domain protein	-0.483
672015261	N/A	N/A	-0.483
149040074	FAM107A	family with sequence similarity 107 member A	-0.482
74181920	FADS3	fatty acid desaturase 3	-0.481
672036991	N/A	N/A	-0.481
149041576	REXO2	RNA exonuclease 2	-0.480
564335541	RGD1310081	similar to hypothetical protein FLJ13231	-0.480
56119120	SNF8	SNF8, ESCRT-II complex subunit	-0.480
197927244	TIE1	tyrosine kinase with immunoglobulin like and EGF like domains 1	-0.480
24980968	ABRACL	ABRA C-terminal like	-0.479
564323057	ARMCX4	armadillo repeat containing, X-linked 4	-0.479
188536057	LRP5	LDL receptor related protein 5	-0.478
41056215	XRCC5	X-ray repair cross complementing 5	-0.478
215272398	HIP1	huntingtin interacting protein 1	-0.477
13786142	SLIT3	slit guidance ligand 3	-0.477
568970276	SH3PXD2B	SH3 and PX domains 2B	-0.476
568970276	SH3PXD2B	SH3 and PX domains 2B	-0.476
51948488	SIRT5	sirtuin 5	-0.476
149020634	TAF1D	TATA-box binding protein associated factor, RNA polymerase I subunit D	-0.475
67972654	TIMP3	TIMP metallopeptidase inhibitor 3	-0.475
158508684	BCAS1	breast carcinoma amplified sequence 1	-0.474
295148052	DCAF17	DDB1 and CUL4 associated factor 17	-0.474

56605714	NDUFAF7	NADH:ubiquinone oxidoreductase complex assembly factor 7	-0.474
149054589	PECAM1	platelet and endothelial cell adhesion molecule 1	-0.474
157787147	TEK	TEK receptor tyrosine kinase	-0.474
157819565	WBP1	WW domain binding protein 1	-0.474
564368910	FN1	fibronectin 1	-0.473
149032573	KIAA1462	KIAA1462	-0.473
189011606	NCEH1	neutral cholesterol ester hydrolase 1	-0.473
672032219	REPS2	RALBP1 associated Eps domain containing 2	-0.473
8394310	SLC4A2	solute carrier family 4 member 2	-0.472
157822957	GPATCH11	G-patch domain containing 11	-0.471
157822627	PLXDC2	plexin domain containing 2	-0.470
149039367	ABCA2	ATP binding cassette subfamily A member 2	-0.469
77917572	LIPA	lipase A, lysosomal acid type	-0.468
149056394	Sipa1l3	signal-induced proliferation-associated 1 like 3	-0.468
564329612	ME3	malic enzyme 3	-0.467
487524631	OBSL1	obscurin like 1	-0.467
672066518	N/A	N/A	-0.467
149027639	Apeg3	antisense paternally expressed gene 3	-0.466
149024084	COL16A1	collagen type XVI alpha 1 chain	-0.465
348605214	TMEM67	transmembrane protein 67	-0.465
635015168	N/A	N/A	-0.465
71051779	MRS2	MRS2, magnesium transporter	-0.464
155369305	PBXIP1	PBX homeobox interacting protein 1	-0.464
158534064	RET	ret proto-oncogene	-0.464
296040479	TXNRD3	thioredoxin reductase 3	-0.464
310703673	GRIN3A	glutamate ionotropic receptor NMDA type subunit 3A	-0.463
8923942	NOP10	NOP10 ribonucleoprotein	-0.463
195976786	CERS2	ceramide synthase 2	-0.462
149058109	HSD17B7	hydroxysteroid 17-beta dehydrogenase 7	-0.462
564344961	RTEL1	regulator of telomere elongation helicase 1	-0.462
149031942	N/A	N/A	-0.462
77539442	EPHX1	epoxide hydrolase 1	-0.461
560186584	VEGFA	vascular endothelial growth factor A	-0.461
966975500	MMP17	matrix metallopeptidase 17	-0.460
47058990	ABCB7	ATP binding cassette subfamily B member 7	-0.459
157818437	CASKIN2	CASK interacting protein 2	-0.459
392339730	COL9A3	collagen type IX alpha 3 chain	-0.459

157819457	MAP3K14	mitogen-activated protein kinase kinase kinase 14	-0.459
880911340	N/A	N/A	-0.458
51854227	GSN	gelsolin	-0.457
672015368	MAST4	microtubule associated serine/threonine kinase family member 4	-0.457
61557206	ZBTB16	zinc finger and BTB domain containing 16	-0.457
57527061	ZGPAT	zinc finger CCCH-type and G-patch domain containing	-0.457
209862829	SEMA3E	semaphorin 3E	-0.456
149274619	ZFHX2	zinc finger homeobox 2	-0.456
122065191	ABAT	4-aminobutyrate aminotransferase	-0.455
157820039	FAM214A	family with sequence similarity 214 member A	-0.455
880886217	N/A	N/A	-0.455
564361507	CRELD2	cysteine rich with EGF like domains 2	-0.454
564327030	DYRK1B	dual specificity tyrosine phosphorylation regulated kinase 1B	-0.454
15011857	SELENOP	selenoprotein P	-0.454
507532705	N/A	N/A	-0.454
40786487	GPR108	G protein-coupled receptor 108	-0.453
672019578	MYSM1	Myb like, SWIRM and MPN domains 1	-0.453
140971205	GRIN2A	glutamate ionotropic receptor NMDA type subunit 2A	-0.452
213512359	NACC2	NACC family member 2	-0.452
53850628	NDUFS1	NADH:ubiquinone oxidoreductase core subunit S1	-0.452
392342157	PHIP	pleckstrin homology domain interacting protein	-0.452
731253346	N/A	N/A	-0.452
13591949	GATM	glycine amidinotransferase	-0.451
635039352	N/A	N/A	-0.451
58865810	NAGA	alpha-N-acetylgalactosaminidase	-0.450
205235	Slc6a7	solute carrier family 6 member 7	-0.450
672015224	N/A	N/A	-0.450
157819371	SYNGR3	synaptogyrin 3	-0.448
817259544	N/A	N/A	-0.448
157822725	GNPDA2	glucosamine-6-phosphate deaminase 2	-0.447
226874871	OMG	oligodendrocyte myelin glycoprotein	-0.446
70794768	HDAC1	histone deacetylase 1	-0.445
395759219	AQP4	aquaporin 4	-0.444

28461153	PPM1F	protein phosphatase, Mg2+/Mn2+ dependent 1F	-0.444
449083357	VWF	von Willebrand factor	-0.444
670979961	N/A	N/A	-0.444
281306842	Med12l	mediator complex subunit 12-like	-0.443
13592005	MVD	mevalonate diphosphate decarboxylase	-0.443
148747464	SCD	stearoyl-CoA desaturase	-0.443
16758746	CNTN4	contactin 4	-0.442
201066395	MBOAT7	membrane bound O-acyltransferase domain containing 7	-0.442
564344383	TOX2	TOX high mobility group box family member 2	-0.441
58865956	TSSC1	tumor suppressing subtransferable candidate 1	-0.441
149065004	N/A	N/A	-0.441
148671944	N/A	N/A	-0.441
149024818	MIB2	mindbomb E3 ubiquitin protein ligase 2	-0.440
564344373	ZMYND8	zinc finger MYND-type containing 8	-0.440
672052684	N/A	N/A	-0.440
157819227	PRPF31	pre-mRNA processing factor 31	-0.439
61556936	CCNL2	cyclin L2	-0.438
157819183	FAM212B	family with sequence similarity 212 member B	-0.437
22024392	KIF1C	kinesin family member 1C	-0.437
402794954	MINK1	misshapen like kinase 1	-0.437
281332095	RB1	RB transcriptional corepressor 1	-0.437
928136440	SRRT	serrate, RNA effector molecule	-0.437
171847060	TTC8	tetratricopeptide repeat domain 8	-0.437
602695898	N/A	N/A	-0.437
392342046	ATR	ATR serine/threonine kinase	-0.436
456367250	ASCC3	activating signal cointegrator 1 complex subunit 3	-0.435
149048674	PEX5L	peroxisomal biogenesis factor 5 like	-0.435
148681112	TGFB2	transforming growth factor beta 2	-0.435
537217423	N/A	N/A	-0.435
402766107	ALDH7A1	aldehyde dehydrogenase 7 family member A1	-0.434
21245094	MAN2C1	mannosidase alpha class 2C member 1	-0.434
58865436	FAR1	fatty acyl-CoA reductase 1	-0.433
148683194	INTS3	integrator complex subunit 3	-0.433
564296010	NHSL1	NHS like 1	-0.433
50510321	ANGPT1	angiopoietin 1	-0.432
157821397	SLC22A15	solute carrier family 22 member 15	-0.432

149030652	TARS2	threonyl-tRNA synthetase 2, mitochondrial (putative)	-0.432
564316234	CEP170	centrosomal protein 170	-0.431
114145766	WDR75	WD repeat domain 75	-0.431
149063684	N/A	N/A	-0.431
672055862	N/A	N/A	-0.431
470611409	N/A	N/A	-0.430
392331978	CDR2L	cerebellar degeneration related protein 2 like	-0.429
8393861	HPCAL4	hippocalcin like 4	-0.429
564363988	ISLR2	immunoglobulin superfamily containing leucine rich repeat 2	-0.429
149058726	N/A	N/A	-0.429
149017535	HDAC10	histone deacetylase 10	-0.428
157786958	RFX1	regulatory factor X1	-0.428
149023083	TUBGCP4	tubulin gamma complex associated protein 4	-0.428
564317997	N/A	N/A	-0.428
309319799	EIF2AK4	eukaryotic translation initiation factor 2 alpha kinase 4	-0.427
149060525	FSTL1	follistatin like 1	-0.427
157822761	MICAL1	microtubule associated monooxygenase, calponin and LIM domain containing 1	-0.427
403310684	MON2	MON2 homolog, regulator of endosome-to-Golgi trafficking	-0.427
2735334	PDPN	podoplanin	-0.427
305855087	UNC5D	unc-5 netrin receptor D	-0.427
148686123	CEND1	cell cycle exit and neuronal differentiation 1	-0.426
61557218	COQ8A	coenzyme Q8A	-0.426
18034793	GABRG1	gamma-aminobutyric acid type A receptor gamma1 subunit	-0.426
558611343	MCM3	minichromosome maintenance complex component 3	-0.426
157817953	RPGRIP1L	RPGRIP1 like	-0.426
300795140	TAF1	TATA-box binding protein associated factor 1	-0.426
160333093	TPRG1L	tumor protein p63 regulated 1 like	-0.426
306482651	DNAJB14	DnaJ heat shock protein family (Hsp40) member B14	-0.425
157818329	PROSER1	proline and serine rich 1	-0.425
564340177	SETX	senataxin	-0.425
672020901	N/A	N/A	-0.425

6978595	CAMK2D	calcium/calmodulin dependent protein kinase II delta	-0.424
77539756	MED24	mediator complex subunit 24	-0.424
672055565	OTOF	otoferlin	-0.424
57528352	ATP5SL	ATP5S like	-0.423
47155563	KIF13B	kinesin family member 13B	-0.423
8393992	PMP22	peripheral myelin protein 22	-0.423
672073522	N/A	N/A	-0.423
148356229	CCND1	cyclin D1	-0.422
672062476	DENND4A	DENN domain containing 4A	-0.422
564375919	USP25	ubiquitin specific peptidase 25	-0.421
295148092	ABLIM2	actin binding LIM protein family member 2	-0.420
672061813	ACSBG1	acyl-CoA synthetase bubblegum family member 1	-0.420
11067415	ERBB4	erb-b2 receptor tyrosine kinase 4	-0.420
46485389	KIRREL	kin of IRRE like (Drosophila)	-0.420
50510949	N/A	N/A	-0.420
29789369	PTPRG	protein tyrosine phosphatase, receptor type G	-0.419
564388675	SLC25A42	solute carrier family 25 member 42	-0.419
672012705	SYNE1	spectrin repeat containing nuclear envelope protein 1	-0.419
25742617	EGFR	epidermal growth factor receptor	-0.418
58865398	LAP3	leucine aminopeptidase 3	-0.418
672051965	NSMAF	neutral sphingomyelinase activation associated factor	-0.418
157822759	PARP2	poly(ADP-ribose) polymerase 2	-0.418
672043401	POGZ	pogo transposable element derived with ZNF domain	-0.418
6978847	FMO1	flavin containing monooxygenase 1	-0.417
148682440	TMEM14A	transmembrane protein 14A	-0.417
157823934	MZF1	myeloid zinc finger 1	-0.416
672029123	PBRM1	polybromo 1	-0.416
16758538	RASGRF2	Ras protein specific guanine nucleotide releasing factor 2	-0.416
62078847	TSEN2	tRNA splicing endonuclease subunit 2	-0.416
568974832	YBX2	Y-box binding protein 2	-0.416
10048483	PCLO	piccolo presynaptic cytomatrix protein	-0.415
377823717	PLEKHA5	pleckstrin homology domain containing A5	-0.415
564305557	PTPRD	protein tyrosine phosphatase, receptor type D	-0.415
827835089	Flt1	FMS-related tyrosine kinase 1	-0.414

755566690	HUWE1	HECT, UBA and WWE domain containing 1, E3 ubiquitin protein ligase	-0.414
564375424	MFSD11	major facilitator superfamily domain containing 11	-0.414
134948398	PDS5A	PDS5 cohesin associated factor A	-0.414
149045696	Ccl27a	chemokine (C-C motif) ligand 27A	-0.413
405113061	MKI67	marker of proliferation Ki-67	-0.413
149042536	MPV17	MPV17, mitochondrial inner membrane protein	-0.413
831326005	N/A	N/A	-0.413
109480433	GNPTAB	N-acetylglucosamine-1-phosphate transferase alpha and beta subunits	-0.412
8393290	S1PR1	sphingosine-1-phosphate receptor 1	-0.412
431910532	N/A	N/A	-0.412
54312088	ATP2B4	ATPase plasma membrane Ca2+ transporting 4	-0.411
157820325	CSE1L	chromosome segregation 1 like	-0.411
166999225	GRM1	glutamate metabotropic receptor 1	-0.411
51948524	IGFBP4	insulin like growth factor binding protein 4	-0.411
157823905	RDH13	retinol dehydrogenase 13	-0.411
148705684	SLIT2	slit guidance ligand 2	-0.411
564363049	STT3A	STT3A, catalytic subunit of the oligosaccharyltransferase complex	-0.411
402478646	WDR44	WD repeat domain 44	-0.411
672013431	N/A	N/A	-0.411
564400249	LIMA1	LIM domain and actin binding 1	-0.410
300797242	SPG11	SPG11, spatacsin vesicle trafficking associated	-0.409
594668377	N/A	N/A	-0.409
157817823	ATG2A	autophagy related 2A	-0.408
672038615	GSG1L	GSG1 like	-0.408
157824160	VSTM2B	V-set and transmembrane domain containing 2B	-0.408
25282445	ENTPD2	ectonucleoside triphosphate diphosphohydrolase 2	-0.407
158635969	FLAD1	flavin adenine dinucleotide synthetase 1	-0.407
62079065	Supt20	suppressor of Ty 20	-0.407
880915307	N/A	N/A	-0.407
149066531	VPS13B	vacuolar protein sorting 13 homolog B	-0.406
13095924	DRP2	dystrophin related protein 2	-0.405
16758712	PDIA4	protein disulfide isomerase family A member 4	-0.405
201860270	NRBP2	nuclear receptor binding protein 2	-0.404

9506901	MPDZ	multiple PDZ domain crumbs cell polarity complex component	-0.403
296439269	PHF10	PHD finger protein 10	-0.403
672083937	TSHZ1	teashirt zinc finger homeobox 1	-0.403
162287337	APOE	apolipoprotein E	-0.402
157822133	VCL	vinculin	-0.401
157824091	ATF7	activating transcription factor 7	-0.400
198041989	PARVB	parvin beta	-0.400
12055542	SLC25A27	solute carrier family 25 member 27	-0.400
13540699	NRP2	neuropilin 2	-0.399
8393896	PACSIN1	protein kinase C and casein kinase substrate in neurons 1	-0.399
472380424	N/A	N/A	-0.399
162287200	CD82	CD82 molecule	-0.398
149063995	GMPR2	guanosine monophosphate reductase 2	-0.398
17298688	HTR2C	5-hydroxytryptamine receptor 2C	-0.398
281371412	LTBP4	latent transforming growth factor beta binding protein 4	-0.398
237757336	OLIG1	oligodendrocyte transcription factor 1	-0.398
62655853	TELO2	telomere maintenance 2	-0.398
300796151	WDR60	WD repeat domain 60	-0.398
672057084	N/A	N/A	-0.398
13162287	DDT	D-dopachrome tautomerase	-0.397
291219919	STK36	serine/threonine kinase 36	-0.397
218505767	Zfp958	zinc finger protein 958	-0.397
77404411	SFT2D2	SFT2 domain containing 2	-0.396
330340430	WDR19	WD repeat domain 19	-0.396
86439949	ENPP2	ectonucleotide pyrophosphatase/phosphodiesterase 2	-0.395
157817777	MAP3K3	mitogen-activated protein kinase kinase kinase 3	-0.395
568961602	VPS13C	vacuolar protein sorting 13 homolog C	-0.395
884874427	N/A	N/A	-0.395
74223968	5031425E22Rik	RIKEN cDNA 5031425E22 gene	-0.394
121583776	LMF2	lipase maturation factor 2	-0.394
168823431	Nrxn3	neurexin III	-0.394
568999446	PTPRM	protein tyrosine phosphatase, receptor type M	-0.394
672063748	N/A	N/A	-0.394
564343781	N/A	N/A	-0.394

149060100	AIFM1	apoptosis inducing factor mitochondria associated 1	-0.393
157823259	TMEM229A	transmembrane protein 229A	-0.393
296010823	UBR1	ubiquitin protein ligase E3 component n-recognin 1	-0.393
167860097	FN3KRP	fructosamine 3 kinase related protein	-0.392
224420	N/A	N/A	-0.392
564315812	NAV1	neuron navigator 1	-0.391
755494737	N/A	N/A	-0.390
12711694	DYNC2H1	dynein cytoplasmic 2 heavy chain 1	-0.389
300793894	URB1	URB1 ribosome biogenesis 1 homolog (S. cerevisiae)	-0.389
564375702	N/A	N/A	-0.388
32452540	RHOT2	ras homolog family member T2	-0.387
149039557	UTRN	utrophin	-0.387
672064087	N/A	N/A	-0.387
23463287	DCPS	decapping enzyme, scavenger	-0.386
672087275	KDM5C	lysine demethylase 5C	-0.386
672049251	ZNF800	zinc finger protein 800	-0.386
672083376	N/A	N/A	-0.386
564393980	ME2	malic enzyme 2	-0.385
149060362	ALCAM	activated leukocyte cell adhesion molecule	-0.384
201066342	MAN2B2	mannosidase alpha class 2B member 2	-0.384
12621108	NR1I3	nuclear receptor subfamily 1 group I member 3	-0.384
148690402	SLC9A3R2	SLC9A3 regulator 2	-0.384
672024549	SLX4	SLX4 structure-specific endonuclease subunit	-0.384
60360648	KLHL2	kelch like family member 2	-0.383
564325866	ZNF274	zinc finger protein 274	-0.383
145553966	CACNA1E	calcium voltage-gated channel subunit alpha1 E	-0.382
16758114	CD93	CD93 molecule	-0.382
13928886	MAP2K1	mitogen-activated protein kinase kinase 1	-0.382
564354247	NPHP4	nephrocystin 4	-0.382
76563954	REXO4	REX4 homolog, 3'-5' exonuclease	-0.382
62079019	UFSP2	UFM1 specific peptidase 2	-0.382
344252018	N/A	N/A	-0.382
157820795	BBS1	Bardet-Biedl syndrome 1	-0.381
42538976	PGAP1	post-GPI attachment to proteins 1	-0.381
564336549	EIF2A	eukaryotic translation initiation factor 2A	-0.380
149044030	JAG2	jagged 2	-0.380
564372688	RPA1	replication protein A1	-0.380

62388885	YIF1B	Yip1 interacting factor homolog B, membrane trafficking protein	-0.380
537212823	N/A	N/A	-0.380
823419836	N/A	N/A	-0.380
157818713	ATM	ATM serine/threonine kinase	-0.379
149065235	Cald1	caldesmon 1	-0.379
157821651	GUF1	GUF1 homolog, GTPase	-0.379
564343174	PLCB4	phospholipase C beta 4	-0.379
109491454	UTP6	UTP6, small subunit processome component	-0.379
6678637	ZBTB14	zinc finger and BTB domain containing 14	-0.379
12018300	AKAP6	A-kinase anchoring protein 6	-0.378
153218522	KDELC2	KDEL motif containing 2	-0.377
157822619	PNMA2	paraneoplastic Ma antigen 2	-0.377
158631185	XPO5	exportin 5	-0.377
564334870	Ap3b1	adaptor-related protein complex 3, beta 1 subunit	-0.376
564367862	Dst	dystonin	-0.376
158186732	GFAP	glial fibrillary acidic protein	-0.376
149034469	GNG7	G protein subunit gamma 7	-0.376
149064540	N/A	N/A	-0.376
564372902	2810408A11Rik	RIKEN cDNA 2810408A11 gene	-0.375
149053229	CAMTA2	calmodulin binding transcription activator 2	-0.375
564316927	FRYL	FRY like transcription coactivator	-0.375
564344520	LOC102555457	engulfment and cell motility protein 2-like	-0.375
76096338	ZEB2	zinc finger E-box binding homeobox 2	-0.375
149056689	ZNF180	zinc finger protein 180	-0.375
157822881	CFP	complement factor properdin	-0.374
7542357	QKI	QKI, KH domain containing RNA binding	-0.374
149055779	SLC17A6	solute carrier family 17 member 6	-0.374
281371490	LAMC1	laminin subunit gamma 1	-0.373
300797477	CUL9	cullin 9	-0.372
48675855	LGALS8	galectin 8	-0.371
149039905	TSPAN17	tetraspanin 17	-0.371
672046176	N/A	N/A	-0.371
6978673	CNR1	cannabinoid receptor 1	-0.370
57527084	HAT1	histone acetyltransferase 1	-0.370
40786505	MAN2B1	mannosidase alpha class 2B member 1	-0.370
564330111	NUP98	nucleoporin 98	-0.370
564377500	N/A	N/A	-0.369
564341299	N/A	N/A	-0.369

13592129	DOC2B	double C2 domain beta	-0.368
348605146	HDAC11	histone deacetylase 11	-0.368
255708437	PIK3CD	phosphatidylinositol-4,5-bisphosphate 3-kinase catalytic subunit delta	-0.368
157819667	SLC41A1	solute carrier family 41 member 1	-0.368
109466809	GATB	glutamyl-tRNA amidotransferase subunit B	-0.367
148668185	RBM26	RNA binding motif protein 26	-0.367
149041411	SC5D	sterol-C5-desaturase	-0.367
13592150	SHARPIN	SHANK associated RH domain interactor	-0.367
6981700	VCAM1	vascular cell adhesion molecule 1	-0.367
149020512	PDE4A	phosphodiesterase 4A	-0.366
78126167	SLC1A2	solute carrier family 1 member 2	-0.366
157822669	SLC36A4	solute carrier family 36 member 4	-0.366
157822279	AK5	adenylate kinase 5	-0.365
149056708	NECTIN2	nectin cell adhesion molecule 2	-0.365
300795339	RYR2	ryanodine receptor 2	-0.365
11024674	SLC9A3R1	SLC9A3 regulator 1	-0.365
403224961	TRPM7	transient receptor potential cation channel subfamily M member 7	-0.365
157822363	PCDH17	protocadherin 17	-0.364
149058577	Ppfia4	protein tyrosine phosphatase, receptor type, f polypeptide (PTPRF), interacting protein (liprin), alpha 4	-0.364
533133321	N/A	N/A	-0.364
82617648	CSMD1	CUB and Sushi multiple domains 1	-0.363
114052238	FIG4	FIG4 phosphoinositide 5-phosphatase	-0.363
109476714	FOCAD	focadhesin	-0.363
149059343	IL6ST	interleukin 6 signal transducer	-0.363
402478644	CARMIL3	capping protein regulator and myosin 1 linker 3	-0.362
210031334	NGEF	neuronal guanine nucleotide exchange factor	-0.362
568977996	PXDN	peroxidasin	-0.362
564382871	N/A	N/A	-0.362
392339847	CADPS2	calcium dependent secretion activator 2	-0.361
672052705	FRRS1L	ferric chelate reductase 1 like	-0.361
564367417	SRF	serum response factor	-0.361
528769450	N/A	N/A	-0.361
157817286	EZH1	enhancer of zeste 1 polycomb repressive complex 2 subunit	-0.360
54035529	SS18	SS18, nBAF chromatin remodeling complex subunit	-0.360

564370831	CASKIN1	CASK interacting protein 1	-0.359
40538868	BABAM2	BRISC and BRCA1 A complex member 2	-0.358
41529837	JUP	junction plakoglobin	-0.358
187937016	WIPI1	WD repeat domain, phosphoinositide interacting 1	-0.358
672076515	N/A	N/A	-0.358
148667088	ATP2B2	ATPase plasma membrane Ca2+ transporting 2	-0.357
158138517	FGFR2	fibroblast growth factor receptor 2	-0.357
56605668	FLII	FLII, actin remodeling protein	-0.357
62647202	KRBA1	KRAB-A domain containing 1	-0.357
149028347	LIG1	DNA ligase 1	-0.357
29789104	NAPB	NSF attachment protein beta	-0.357
219803038	PDE2A	phosphodiesterase 2A	-0.356
197313640	TMEM132E	transmembrane protein 132E	-0.356
672029178	CCSER2	coiled-coil serine rich protein 2	-0.354
9506957	PCSK7	proprotein convertase subtilisin/kexin type 7	-0.354
392353178	SEL1L3	SEL1L family member 3	-0.354
62078997	WDR1	WD repeat domain 1	-0.354
672063876	MGC116197 (includes others)	similar to RIKEN cDNA 1700001E04	-0.353
157819941	PEX26	peroxisomal biogenesis factor 26	-0.353
70608092	SLC9A8	solute carrier family 9 member A8	-0.353
300796069	THADA	THADA, armadillo repeat containing	-0.353
672057964	N/A	N/A	-0.353
162287198	HSD17B4	hydroxysteroid 17-beta dehydrogenase 4	-0.352
18266684	MSMO1	methylsterol monooxygenase 1	-0.352
197387642	ZNF710	zinc finger protein 710	-0.352
148693587	N/A	N/A	-0.352
564317068	CCDC149	coiled-coil domain containing 149	-0.351
149058381	RNASEL	ribonuclease L	-0.351
299829287	DISP2	dispatched RND transporter family member 2	-0.350
115292425	KIRREL3	kin of IRRE like 3 (Drosophila)	-0.350
672041794	RGD1310081	similar to hypothetical protein FLJ13231	-0.350
755536182	RHBDF1	rhomboid 5 homolog 1	-0.350
198278545	IAH1	isoamyl acetate-hydrolyzing esterase 1 homolog	-0.349
564342402	PLA2G4B	phospholipase A2 group IVB	-0.349
149066868	MDM1	Mdm1 nuclear protein	-0.348
38181552	SCG2	secretogranin II	-0.348
564400341	N/A	N/A	-0.348
564332376	RASGRP2	RAS guanyl releasing protein 2	-0.347

30519995	SFXN5	sideroflexin 5	-0.347
6678297	TEX261	testis expressed 261	-0.347
296010825	UBR2	ubiquitin protein ligase E3 component n-recognin 2	-0.347
187469267	GPRC5B	G protein-coupled receptor class C group 5 member B	-0.346
206558322	JMJD8	jumonji domain containing 8	-0.346
913512819	N/A	N/A	-0.346
148675460	ARFRP1	ADP ribosylation factor related protein 1	-0.345
157822769	KLF12	Kruppel like factor 12	-0.345
77157795	MAL2	mal, T-cell differentiation protein 2 (gene/pseudogene)	-0.345
54019432	PCDHA7	protocadherin alpha 7	-0.345
672024793	N/A	N/A	-0.345
635102546	N/A	N/A	-0.345
564385704	FLNB	filamin B	-0.344
183979966	HSPG2	heparan sulfate proteoglycan 2	-0.344
564372825	SGSM2	small G protein signaling modulator 2	-0.344
148707634	SHISA4	shisa family member 4	-0.344
564384429	N/A	N/A	-0.344
157822247	CHORDC1	cysteine and histidine rich domain containing 1	-0.343
690969206	MBD6	methyl-CpG binding domain protein 6	-0.343
29789305	PTPRN	protein tyrosine phosphatase, receptor type N	-0.343
685536524	N/A	N/A	-0.343
213688380	GXYLT1	glucoside xylosyltransferase 1	-0.342
57164111	IFT122	intraflagellar transport 122	-0.342
30842796	MAST1	microtubule associated serine/threonine kinase 1	-0.342
58865476	PIGK	phosphatidylinositol glycan anchor biosynthesis class K	-0.342
164607158	PTPRR	protein tyrosine phosphatase, receptor type R	-0.342
564397593	RAB36	RAB36, member RAS oncogene family	-0.341
281306814	RPS6KA2	ribosomal protein S6 kinase A2	-0.341
6981574	SPARC	secreted protein acidic and cysteine rich	-0.341
8394502	UBC	ubiquitin C	-0.341
149016843	N/A	N/A	-0.340
564372562	PFAS	phosphoribosylformylglycinamidine synthase	-0.339
564314535	RUBCN	RUN and cysteine rich domain containing beclin 1 interacting protein	-0.338
564330477	TEAD1	TEA domain transcription factor 1	-0.337

672065543	TNS1	tensin 1	-0.337
564301979	CKAP5	cytoskeleton associated protein 5	-0.336
328683463	LRP4	LDL receptor related protein 4	-0.336
149027291	PDZD2	PDZ domain containing 2	-0.336
564304046	PLXNA1	plexin A1	-0.336
672052120	RBM12B	RNA binding motif protein 12B	-0.336
672051414	N/A	N/A	-0.336
32527705	N/A	N/A	-0.336
148669742	ADD3	adducin 3	-0.335
755540019	N/A	N/A	-0.335
564349673	C2CD5	C2 calcium dependent domain containing 5	-0.334
840088206	INTS11	integrator complex subunit 11	-0.333
158749540	NPEPPS	aminopeptidase puromycin sensitive	-0.333
58865454	SCYL1	SCY1 like pseudokinase 1	-0.333
157823901	TSPAN9	tetraspanin 9	-0.333
564378945	N/A	N/A	-0.333
149063401	ALDH2	aldehyde dehydrogenase 2 family (mitochondrial)	-0.332
300794608	CEP120	centrosomal protein 120	-0.332
764020083	CLUH	clustered mitochondria homolog	-0.332
755524429	Evi5l	ecotropic viral integration site 5 like	-0.332
57164145	NT5DC2	5'-nucleotidase domain containing 2	-0.332
564346102	N/A	N/A	-0.332
149022319	AGPS	alkylglycerone phosphate synthase	-0.331
157822141	KBTBD3	kelch repeat and BTB domain containing 3	-0.331
158138535	NUP107	nucleoporin 107	-0.331
641706489	N/A	N/A	-0.331
142349612	GLUL	glutamate-ammonia ligase	-0.330
109484871	HERC1	HECT and RLD domain containing E3 ubiquitin protein ligase family member 1	-0.330
57164113	NSDHL	NAD(P) dependent steroid dehydrogenase-like	-0.330
227913	N/A	N/A	-0.330
564376923	GOLGB1	golgin B1	-0.329
564358911	CHPT1	choline phosphotransferase 1	-0.328
16758280	LYST	lysosomal trafficking regulator	-0.328
157822933	ZNF385A	zinc finger protein 385A	-0.328
743735648	N/A	N/A	-0.328
11693162	INSIG1	insulin induced gene 1	-0.327
564364873	ADAMTS7	ADAM metallopeptidase with thrombospondin type 1 motif 7	-0.326

157822539	ANK1	ankyrin 1	-0.326
157817476	HECW2	HECT, C2 and WW domain containing E3 ubiquitin protein ligase 2	-0.325
392348740	LAMB1	laminin subunit beta 1	-0.325
158186672	Nedd4	neural precursor cell expressed, developmentally down-regulated 4	-0.325
149062647	Rfx3	regulatory factor X3	-0.325
157819337	SLC35B4	solute carrier family 35 member B4	-0.325
157819187	AGL	amylo-alpha-1, 6-glucosidase, 4-alpha-glucanotransferase	-0.324
564394868	CC2D1A	coiled-coil and C2 domain containing 1A	-0.324
290563168	DUSP3	dual specificity phosphatase 3	-0.324
157822337	FGD3	FYVE, RhoGEF and PH domain containing 3	-0.324
8393490	GRM5	glutamate metabotropic receptor 5	-0.324
281604190	INPP5B	inositol polyphosphate-5-phosphatase B	-0.324
198041681	LTN1	listerin E3 ubiquitin protein ligase 1	-0.324
27229135	MARCH8	membrane associated ring-CH-type finger 8	-0.324
20376820	MFN1	mitofusin 1	-0.324
158186636	PDGFRA	platelet derived growth factor receptor alpha	-0.324
403259801	N/A	N/A	-0.324
194473622	ADSL	adenylosuccinate lyase	-0.323
564325169	AGPAT4	1-acylglycerol-3-phosphate O-acyltransferase 4	-0.323
157817478	PIK3C2A	phosphatidylinositol-4-phosphate 3-kinase catalytic subunit type 2 alpha	-0.323
731197958	N/A	N/A	-0.323
12831227	AACS	acetoacetyl-CoA synthetase	-0.322
300795884	DOCK11	dedicator of cytokinesis 11	-0.322
672068740	NEWGENE_1308105	kinase suppressor of ras 1	-0.322
564371801	TBC1D9B	TBC1 domain family member 9B	-0.322
6678349	TIAL1	TIA1 cytotoxic granule associated RNA binding protein like 1	-0.322
62078971	UBLCP1	ubiquitin like domain containing CTD phosphatase 1	-0.322
149050766	CAD	carbamoyl-phosphate synthetase 2, aspartate transcarbamylase, and dihydroorotase	-0.321
58865808	CBX6	chromobox 6	-0.321
9507083	SEMA4F	ssemaphorin 4F	-0.321
97537309	SYNJ1	synaptojanin 1	-0.321
58865906	PLD3	phospholipase D family member 3	-0.320

13027430	WDR7	WD repeat domain 7	-0.320
672084703	N/A	N/A	-0.320
157822535	LATS2	large tumor suppressor kinase 2	-0.319
16758890	MYO1B	myosin IB	-0.319
81230489	PCDHGA8	protocadherin gamma subfamily A, 8	-0.319
564379349	Zcchc8	zinc finger CCHC-type containing 8	-0.319
564360941	N/A	N/A	-0.319
148682503	IMP4	IMP4 homolog, U3 small nucleolar ribonucleoprotein	-0.318
826336802	N/A	N/A	-0.318
223590233	DDN	dendrin	-0.317
74354506	ACBD5	acyl-CoA binding domain containing 5	-0.316
50511316	AHI1	Abelson helper integration site 1	-0.316
564357619	ITGB8	integrin subunit beta 8	-0.315
62078695	MLEC	malectin	-0.314
67078434	SLC25A39	solute carrier family 25 member 39	-0.314
532042299	N/A	N/A	-0.314
84662766	ELAVL4	ELAV like RNA binding protein 4	-0.313
56799390	ATP1B2	ATPase Na+/K+ transporting subunit beta 2	-0.312
11693172	CALR	calreticulin	-0.312
148704942	NAMPT	nicotinamide phosphoribosyltransferase	-0.312
149020944	PFKP	phosphofructokinase, platelet	-0.312
157787028	SUSD4	sushi domain containing 4	-0.312
157817500	UBAP2	ubiquitin associated protein 2	-0.312
148372343	RAMP2	receptor activity modifying protein 2	-0.311
672030652	N/A	N/A	-0.311
672067893	N/A	N/A	-0.311
925114268	GTF3C1	general transcription factor IIIC subunit 1	-0.310
149016209	SLC4A3	solute carrier family 4 member 3	-0.310
634833336	N/A	N/A	-0.310
777419	N/A	N/A	-0.310
674093471	N/A	N/A	-0.310
672041570	N/A	N/A	-0.310
807677	N/A	N/A	-0.309
672014573	N/A	N/A	-0.309
157823930	PPP2R5A	protein phosphatase 2 regulatory subunit B'alpha	-0.308
157819753	RCN1	reticulocalbin 1	-0.308
157820971	TMEM150C	transmembrane protein 150C	-0.308
157818787	BBS4	Bardet-Biedl syndrome 4	-0.307

537271325	N/A	N/A	-0.307
404501459	ARHGEF40	Rho guanine nucleotide exchange factor 40	-0.306
27229304	ELAC2	elaC ribonuclease Z 2	-0.306
149046617	MAGI2	membrane associated guanylate kinase, WW and PDZ domain containing 2	-0.306
564338426	N/A	N/A	-0.306
954249788	N/A	N/A	-0.306
664708230	N/A	N/A	-0.306
84490431	DNM3	dynamin 3	-0.305
300794741	FNDC3B	fibronectin type III domain containing 3B	-0.305
26378096	OSTC	oligosaccharyltransferase complex non-catalytic subunit	-0.305
149041432	THY1	Thy-1 cell surface antigen	-0.305
564325846	Zfp40	zinc finger protein 40	-0.305
451172073	CHRM3	cholinergic receptor muscarinic 3	-0.304
148702078	CPSF3	cleavage and polyadenylation specific factor 3	-0.304
28972363	DOCK4	dedicator of cytokinesis 4	-0.304
149049163	GRIN2B	glutamate ionotropic receptor NMDA type subunit 2B	-0.304
38454284	PPM1E	protein phosphatase, Mg2+/Mn2+ dependent 1E	-0.304
568979427	RCOR1	REST corepressor 1	-0.304
62543499	VGLL4	vestigial like family member 4	-0.304
149048968	ITPR2	inositol 1,4,5-trisphosphate receptor type 2	-0.303
564361015	TRIOBP	TRIO and F-actin binding protein	-0.303
157821429	BAZ2A	bromodomain adjacent to zinc finger domain 2A	-0.302
197246191	PDXDC1	pyridoxal dependent decarboxylase domain containing 1	-0.302
765099237	XRN1	5'-3' exoribonuclease 1	-0.302
50510855	RIMKLB	ribosomal modification protein rimK like family member B	-0.301
51948538	ZMYND10	zinc finger MYND-type containing 10	-0.301
33299962	CAMK1G	calcium/calmodulin dependent protein kinase IG	-0.300
149029475	EPG5	ectopic P-granules autophagy protein 5 homolog	-0.300
564367517	ADGRF5	adhesion G protein-coupled receptor F5	-0.299
564387864	CACNA1D	calcium voltage-gated channel subunit alpha1 D	-0.299
148696689	MFAP3L	microfibrillar associated protein 3 like	-0.299

59858990	UNC13A	unc-13 homolog A	-0.299
672088357	ZCCHC18	zinc finger CCHC-type containing 18	-0.299
293349986	SMCHD1	structural maintenance of chromosomes flexible hinge domain containing 1	-0.298
672017219	ZNF106	zinc finger protein 106	-0.298
149022622	ACP2	acid phosphatase 2, lysosomal	-0.297
815891121	ZEB1	zinc finger E-box binding homeobox 1	-0.297
189339241	MAN2A1	mannosidase alpha class 2A member 1	-0.296
149024245	RSRP1	arginine and serine rich protein 1	-0.296
157820653	TMEM63C	transmembrane protein 63C	-0.296
568938931	TRRAP	transformation/transcription domain associated protein	-0.296
672019307	N/A	N/A	-0.296
9507167	SYNGR1	synaptogyrin 1	-0.295
68163551	TBC1D22B	TBC1 domain family member 22B	-0.295
807677	N/A	N/A	-0.295
198278430	OSBPL9	oxysterol binding protein like 9	-0.294
37360344	PDCD6IP	programmed cell death 6 interacting protein	-0.294
13928780	POR	cytochrome p450 oxidoreductase	-0.294
686661093	SLC24A3	solute carrier family 24 member 3	-0.294
157817560	ZCCHC7	zinc finger CCHC-type containing 7	-0.294
568986622	CNIH1	cornichon family AMPA receptor auxiliary protein 1	-0.293
380876953	MYO10	myosin X	-0.293
62078459	CEP95	centrosomal protein 95	-0.292
157822873	FBXO18	F-box protein, helicase, 18	-0.292
25742763	HSPA5	heat shock protein family A (Hsp70) member 5	-0.292
564328017	RASIP1	Ras interacting protein 1	-0.292
157820833	HERC3	HECT and RLD domain containing E3 ubiquitin protein ligase 3	-0.291
731267527	N/A	N/A	-0.291
655879926	N/A	N/A	-0.291
157817971	FAM13B	family with sequence similarity 13 member B	-0.290
149017194	KDM3B	lysine demethylase 3B	-0.290
270288740	MAOA	monoamine oxidase A	-0.290
61556891	OSBPL2	oxysterol binding protein like 2	-0.290
296470851	PABPC1L2A	poly(A) binding protein cytoplasmic 1 like 2A	-0.290
157821191	CHST11	carbohydrate sulfotransferase 11	-0.289
285026506	IDUA	iduronidase, alpha-L-	-0.289
77993374	ARSB	arylsulfatase B	-0.288

57528238	PEPD	peptidase D	-0.288
149058978	EDIL3	EGF like repeats and discoidin domains 3	-0.287
71795664	MAGI1	membrane associated guanylate kinase, WW and PDZ domain containing 1	-0.287
148283739	SELENOO	selenoprotein O	-0.287
157821127	TROVE2	TROVE domain family member 2	-0.287
672061591	N/A	N/A	-0.287
564347675	AAK1	AP2 associated kinase 1	-0.286
672035097	LENG8	leukocyte receptor cluster member 8	-0.286
564364473	RNF111	ring finger protein 111	-0.286
38259192	TOP2A	topoisomerase (DNA) II alpha	-0.286
564314389	DZIP3	DAZ interacting zinc finger protein 3	-0.285
392333710	COL4A2	collagen type IV alpha 2 chain	-0.284
672083212	N/A	N/A	-0.284
344255506	N/A	N/A	-0.284
62339281	ADAM9	ADAM metallopeptidase domain 9	-0.283
124486885	LRRC7	leucine rich repeat containing 7	-0.283
157821205	PAOX	polyamine oxidase	-0.283
56605656	DONSON	downstream neighbor of SON	-0.282
564396646	VARS2	valyl-tRNA synthetase 2, mitochondrial	-0.282
672042073	N/A	N/A	-0.282
674043799	N/A	N/A	-0.282
568950414	ATXN2L	ataxin 2 like	-0.281
148689929	CYFIP1	cytoplasmic FMR1 interacting protein 1	-0.281
576796148	MAP7D2	MAP7 domain containing 2	-0.281
56090433	GLT8D1	glycosyltransferase 8 domain containing 1	-0.280
672085253	SPG7	SPG7, paraplegin matrix AAA peptidase subunit	-0.280
149056749	Clasrp	CLK4-associating serine/arginine rich protein	-0.279
157823031	IPO4	importin 4	-0.279
1335860	PRKAG1	protein kinase AMP-activated non-catalytic subunit gamma 1	-0.279
334724478	SRGAP1	SLIT-ROBO Rho GTPase activating protein 1	-0.279
281604092	VCAN	versican	-0.279
149016025	N/A	N/A	-0.279
19705443	MYO9A	myosin IXA	-0.278
149066394	SAMD12	sterile alpha motif domain containing 12	-0.278
672022833	Scaper	S-phase cyclin A-associated protein in the ER	-0.278
859862188	N/A	N/A	-0.278
564337214	FAM189B	family with sequence similarity 189 member B	-0.277

114145640	LOC499219	hypothetical protein LOC499219	-0.277
62078801	MEF2A	myocyte enhancer factor 2A	-0.277
672088848	PLXNA3	plexin A3	-0.277
672052597	ABCA1	ATP binding cassette subfamily A member 1	-0.276
157818605	ABCG4	ATP binding cassette subfamily G member 4	-0.276
54312094	DAGLA	diacylglycerol lipase alpha	-0.276
754169724	SERINC5	serine incorporator 5	-0.276
293346766	TCAF1	TRPM8 channel associated factor 1	-0.276
564312952	ZZEF1	zinc finger ZZ-type and EF-hand domain containing 1	-0.276
19173794	LOC678813/Marf1	similar to limkain b1	-0.275
672022994	NEO1	neogenin 1	-0.275
62079039	PHKB	phosphorylase kinase regulatory subunit beta	-0.275
635147633	N/A	N/A	-0.275
53759110	CR1L	complement C3b/C4b receptor 1 like	-0.274
67846040	POMK	protein-O-mannose kinase	-0.274
564361228	TTLL1	tubulin tyrosine ligase like 1	-0.274
114052795	AMZ1	archaelysin family metallopeptidase 1	-0.273
672066638	CLEC16A	C-type lectin domain containing 16A	-0.273
145312274	EPHA6	EPH receptor A6	-0.273
25006379	GUCY1A3	guanylate cyclase 1 soluble subunit alpha	-0.273
469469055	KCNQ3	potassium voltage-gated channel subfamily Q member 3	-0.273
564305530	PTPRD	protein tyrosine phosphatase, receptor type D	-0.273
537146226	N/A	N/A	-0.273
564298436	WDR11	WD repeat domain 11	-0.272
11993954	METAP2	methionyl aminopeptidase 2	-0.271
20127390	RNF112	ring finger protein 112	-0.271
70778983	SFPQ	splicing factor proline and glutamine rich	-0.271
148695071	STK39	serine/threonine kinase 39	-0.271
12018268	ADCY5	adenylate cyclase 5	-0.270
157821679	ANAPC4	anaphase promoting complex subunit 4	-0.270
62650795	DACT1	dishevelled binding antagonist of beta catenin 1	-0.270
148680991	PLXNA2	plexin A2	-0.270
149019021	Sh3bgrl2	SH3 domain binding glutamate-rich protein like 2	-0.270
281485606	STT3B	STT3B, catalytic subunit of the oligosaccharyltransferase complex	-0.270
564313504	TEX2	testis expressed 2	-0.270
672084347	N/A	N/A	-0.270

672048013	N/A	N/A	-0.270
17865325	GLRB	glycine receptor beta	-0.269
672038527	PLEKHA1	pleckstrin homology domain containing A1	-0.269
584277046	SLC1A3	solute carrier family 1 member 3	-0.269
537233715	N/A	N/A	-0.269
564390898	KIF13A	kinesin family member 13A	-0.268
392342224	N/A	N/A	-0.268
9506469	CD47	CD47 molecule	-0.267
564314685	EIF4G1	eukaryotic translation initiation factor 4 gamma 1	-0.267
6981076	IDE	insulin degrading enzyme	-0.267
157822211	CTC1	CST telomere replication complex component 1	-0.266
157824032	Ptprt	protein tyrosine phosphatase, receptor type, T	-0.266
913500389	N/A	N/A	-0.266
564342542	MAP1A	microtubule associated protein 1A	-0.265
157816949	NOMO1 (includes others)	NODAL modulator 1	-0.264
984094253	N/A	N/A	-0.264
300797892	C2CD3	C2 calcium dependent domain containing 3	-0.262
157818225	CNTNAP4	contactin associated protein like 4	-0.262
149053315	CAMKK1	calcium/calmodulin dependent protein kinase kinase 1	-0.261
672050038	NDNF	neuron derived neurotrophic factor	-0.261
564370872	N/A	N/A	-0.261
119226197	CXXC1	CXXC finger protein 1	-0.260
564385664	FERMT2	fermitin family member 2	-0.260
149028240	Fsd1	fibronectin type III and SPRY domain containing 1	-0.260
564334920	HMGCR	3-hydroxy-3-methylglutaryl-CoA reductase	-0.260
291084699	TDRKH	tudor and KH domain containing	-0.260
913512216	N/A	N/A	-0.260
672061209	N/A	N/A	-0.260
564397229	ANKS1A	ankyrin repeat and sterile alpha motif domain containing 1A	-0.259
564299655	FAM169A	family with sequence similarity 169 member A	-0.259
148828533	NALCN	sodium leak channel, non-selective	-0.259
312283667	WNK1	WNK lysine deficient protein kinase 1	-0.259
149048608	N/A	N/A	-0.259
46048420	CLU	clusterin	-0.258
148677354	CNOT10	CCR4-NOT transcription complex subunit 10	-0.258

22902132	RBM10	RNA binding motif protein 10	-0.258
207113155	SELENOI	selenoprotein I	-0.258
76096306	VAT1	vesicle amine transport 1	-0.258
157823887	MLC1	megalencephalic leukoencephalopathy with subcortical cysts 1	-0.257
37359962	PLPPR4	phospholipid phosphatase related 4	-0.257
61556910	SNX10	sorting nexin 10	-0.257
672079582	N/A	N/A	-0.257
672016955	MAP3K20	mitogen-activated protein kinase kinase kinase 20	-0.256
67846078	TMEM55A	transmembrane protein 55A	-0.256
568928882	ZFYVE9	zinc finger FYVE-type containing 9	-0.256
675747737	N/A	N/A	-0.256
21322238	CABP7	calcium binding protein 7	-0.255
157817995	DOT1L	DOT1 like histone lysine methyltransferase	-0.255
149041904	GLCE	glucuronic acid epimerase	-0.255
564382292	ANGEL2	angel homolog 2	-0.254
392337767	AP3B2	adaptor related protein complex 3 beta 2 subunit	-0.254
157819725	CEP131	centrosomal protein 131	-0.254
149030303	ELP3	elongator acetyltransferase complex subunit 3	-0.254
564388440	MYO9B	myosin IXB	-0.254
564374356	ADAM11	ADAM metallopeptidase domain 11	-0.253
300793879	BAZ1B	bromodomain adjacent to zinc finger domain 1B	-0.253
47087105	RGL2	ral guanine nucleotide dissociation stimulator like 2	-0.253
724945729	N/A	N/A	-0.253
148695758	CAPRIN1	cell cycle associated protein 1	-0.252
415703079	NEBL	nebulette	-0.252
115432015	RASA3	RAS p21 protein activator 3	-0.252
564359927	RIMS2	regulating synaptic membrane exocytosis 2	-0.252
67846010	ROGDI	rogdi homolog	-0.252
6981672	Tpm4	tropomyosin 4	-0.252
564322686	N/A	N/A	-0.252
148677779	N/A	N/A	-0.252
48675845	ATIC	5-aminoimidazole-4-carboxamide ribonucleotide formyltransferase/IMP cyclohydrolase	-0.251
564392757	NOL4	nucleolar protein 4	-0.251

124249077	POLR2E	RNA polymerase II subunit E	-0.251
8394354	SQLE	squalene epoxidase	-0.251
672037929	TNRC6A	trinucleotide repeat containing 6A	-0.251
158341644	USP54	ubiquitin specific peptidase 54	-0.251
564352388	N/A	N/A	-0.251
411147403	APLP2	amyloid beta precursor like protein 2	-0.250
157817456	C2CD4C	C2 calcium dependent domain containing 4C	-0.250
148697866	FAM3A	family with sequence similarity 3 member A	-0.250
148708948	PRPF4B	pre-mRNA processing factor 4B	-0.250
13027442	ARHGEF11	Rho guanine nucleotide exchange factor 11	-0.249
148703921	FNDC3A	fibronectin type III domain containing 3A	-0.249
564323143	Gprasp2	G protein-coupled receptor associated sorting protein 2	-0.249
157817620	PSD2	pleckstrin and Sec7 domain containing 2	-0.249
149027971	ATF6B	activating transcription factor 6 beta	-0.248
564334013	GBF1	golgi brefeldin A resistant guanine nucleotide exchange factor 1	-0.247
157817839	SEMA5A	semaphorin 5A	-0.247
15805026	ZFAND6	zinc finger AN1-type containing 6	-0.247
148683809	N/A	N/A	-0.247
594629505	N/A	N/A	-0.247
672040941	ATRNL1	attractin like 1	-0.246
9506591	FDFT1	farnesyl-diphosphate farnesyltransferase 1	-0.246
564345393	PRKAG2	protein kinase AMP-activated non-catalytic subunit gamma 2	-0.246
148701638	SEPT8	septin 8	-0.246
564387640	DOCK9	dedicator of cytokinesis 9	-0.245
6981208	NR3C2	nuclear receptor subfamily 3 group C member 2	-0.245
224471866	UGGT1	UDP-glucose glycoprotein glucosyltransferase 1	-0.245
444728185	N/A	N/A	-0.245
148672873	PTPRK	protein tyrosine phosphatase, receptor type K	-0.244
564327171	ACTN4	actinin alpha 4	-0.243
149016256	IRS1	insulin receptor substrate 1	-0.243
281604229	LAS1L	LAS1 like, ribosome biogenesis factor	-0.243
33413429	NTRK3	neurotrophic receptor tyrosine kinase 3	-0.243
40786489	ARHGEF25	Rho guanine nucleotide exchange factor 25	-0.242
170016079	ATP13A1	ATPase 13A1	-0.242
564309890	CBL	Cbl proto-oncogene	-0.242
56090393	DCAKD	dephospho-CoA kinase domain containing	-0.242
72255531	EFHD2	EF-hand domain family member D2	-0.242

30061483	HAP1	huntingtin associated protein 1	-0.242
226874873	GUK1	guanylate kinase 1	-0.241
158303324	ITGB1	integrin subunit beta 1	-0.241
672019361	TMEM245	transmembrane protein 245	-0.241
564311572	N/A	N/A	-0.241
196115100	CSTF2	cleavage stimulation factor subunit 2	-0.240
62078863	ZCCHC12	zinc finger CCHC-type containing 12	-0.240
55742755	CTNNA1	catenin alpha 1	-0.239
300793877	DOPEY2	dopey family member 2	-0.239
81295339	TECPR1	tectonin beta-propeller repeat containing 1	-0.239
149048094	N/A	N/A	-0.239
686713740	N/A	N/A	-0.239
149634159	N/A	N/A	-0.239
294845709	OCRL	OCRL, inositol polyphosphate-5-phosphatase	-0.237
149066130	PLEC	plectin	-0.237
61557085	SPTBN1	spectrin beta, non-erythrocytic 1	-0.237
157821453	TBC1D10B	TBC1 domain family member 10B	-0.237
157822357	SLC25A23	solute carrier family 25 member 23	-0.236
564347453	ALMS1	ALMS1, centrosome and basal body associated protein	-0.235
564299821	PARP8	poly(ADP-ribose) polymerase family member 8	-0.235
158186708	PDCD11	programmed cell death 11	-0.235
13786144	PREP	prolyl endopeptidase	-0.235
148682490	RAB23	RAB23, member RAS oncogene family	-0.235
77404395	SND1	staphylococcal nuclease and tudor domain containing 1	-0.235
149067833	ALDOA	aldolase, fructose-bisphosphate A	-0.234
201023331	MAPK11	mitogen-activated protein kinase 11	-0.234
109467956	PPP1R26	protein phosphatase 1 regulatory subunit 26	-0.234
197245729	CPSF1	cleavage and polyadenylation specific factor 1	-0.233
564382183	EPRS	glutamyl-prolyl-tRNA synthetase	-0.233
48675867	PLPP3	phospholipid phosphatase 3	-0.233
159163110	N/A	N/A	-0.233
149024626	EXOSC10	exosome component 10	-0.232
149041012	SMYD2	SET and MYND domain containing 2	-0.232
564400604	THOC2	THO complex 2	-0.232
157819829	HACD3	3-hydroxyacyl-CoA dehydratase 3	-0.230
26006243	KCND2	potassium voltage-gated channel subfamily D member 2	-0.230
189083764	MARS	methionyl-tRNA synthetase	-0.230

281604211	RAB3GAP2	RAB3 GTPase activating non-catalytic protein subunit 2	-0.230
148710162	SLC8A2	solute carrier family 8 member A2	-0.230
158711736	SMC2	structural maintenance of chromosomes 2	-0.230
157819077	TRIM37	tripartite motif containing 37	-0.230
537146709	N/A	N/A	-0.230
56090241	THTPA	thiamine triphosphatase	-0.229
564397109	N/A	N/A	-0.229
564344160	CHD6	chromodomain helicase DNA binding protein 6	-0.228
109464982	KIAA0922	KIAA0922	-0.228
157819469	SHPRH	SNF2 histone linker PHD RING helicase	-0.228
913505527	N/A	N/A	-0.228
148693489	Nrgn	neurogranin	-0.227
25742576	NXF1	nuclear RNA export factor 1	-0.227
18543353	SLC5A6	solute carrier family 5 member 6	-0.227
267133	TIMP2	TIMP metallopeptidase inhibitor 2	-0.227
564400166	ACSL4	acyl-CoA synthetase long-chain family member 4	-0.226
149052868	MFAP4	microfibrillar associated protein 4	-0.226
56090291	PIGQ	phosphatidylinositol glycan anchor biosynthesis class Q	-0.226
717324516	SCN8A	sodium voltage-gated channel alpha subunit 8	-0.226
57351	Tgoln1	trans-golgi network protein	-0.226
672081765	N/A	N/A	-0.226
564328949	N/A	N/A	-0.226
189163481	ISYNA1	inositol-3-phosphate synthase 1	-0.225
148681399	SZRD1	SUZ RNA binding domain containing 1	-0.225
18677755	ADGRL3	adhesion G protein-coupled receptor L3	-0.224
149061951	CORO1B	coronin 1B	-0.224
564382544	ZNF644	zinc finger protein 644	-0.224
148747421	IGFBP2	insulin like growth factor binding protein 2	-0.223
564329414	IKBKG	inhibitor of nuclear factor kappa B kinase subunit gamma	-0.223
283837871	LRP1	LDL receptor related protein 1	-0.223
198278525	RIC8A	RIC8 guanine nucleotide exchange factor A	-0.221
281306738	PCDH19	protocadherin 19	-0.220
861445795	N/A	N/A	-0.220
564393851	N/A	N/A	-0.220
148689145	CPNE4	copine 4	-0.219
149045522	PNISR	PNN interacting serine and arginine rich protein	-0.219

13242271	SLC6A11	solute carrier family 6 member 11	-0.218
74151445	Zranb2	zinc finger, RAN-binding domain containing 2	-0.218
149031313	N/A	N/A	-0.218
625251453	N/A	N/A	-0.218
672088942	ATP2B3	ATPase plasma membrane Ca2+ transporting 3	-0.217
148687519	CALN1	calneuron 1	-0.217
12621120	SFXN3	sideroflexin 3	-0.216
78000203	Tpm1	tropomyosin 1, alpha	-0.216
157819361	TTYH1	tweety family member 1	-0.216
744541945	N/A	N/A	-0.216
731271938	N/A	N/A	-0.216
11559947	CASK	calcium/calmodulin dependent serine protein kinase	-0.215
16923964	CNTN1	contactin 1	-0.215
57526927	LARS	leucyl-tRNA synthetase	-0.215
767172165	NOTCH2	notch 2	-0.215
9507177	USO1	USO1 vesicle transport factor	-0.215
672039485	N/A	N/A	-0.215
157821335	GPR162	G protein-coupled receptor 162	-0.214
40786511	MICU1	mitochondrial calcium uptake 1	-0.214
564315812	NAV1	neuron navigator 1	-0.214
149034828	N/A	N/A	-0.214
672034978	N/A	N/A	-0.214
47846864	CADM1	cell adhesion molecule 1	-0.213
13786140	CELSR3	cadherin EGF LAG seven-pass G-type receptor 3	-0.213
197209847	JAK1	Janus kinase 1	-0.212
1334149	N/A	N/A	-0.212
755533955	ANK3	ankyrin 3	-0.211
21070934	CALCOCO1	calcium binding and coiled-coil domain 1	-0.211
149031313	N/A	N/A	-0.211
35215304	CLMP	CXADR like membrane protein	-0.210
155369271	PRKACA	protein kinase cAMP-activated catalytic subunit alpha	-0.210
564344879	N/A	N/A	-0.210
56090379	POMGNT1	protein O-linked mannose N-acetylglucosaminyltransferase 1 (beta 1,2-)	-0.209
564385371	SEC24C	SEC24 homolog C, COPII coat complex component	-0.209
149050844	DNMT3A	DNA methyltransferase 3 alpha	-0.208

47059112	EHMT2	euchromatic histone lysine methyltransferase 2	-0.208
564385965	SALL2	spalt like transcription factor 2	-0.208
300793998	SHISA6	shisa family member 6	-0.207
77628157	ST18	ST18, C2H2C-type zinc finger	-0.207
39104626	CAMK2A	calcium/calmodulin dependent protein kinase II alpha	-0.206
472824971	MEGF8	multiple EGF like domains 8	-0.206
109488292	POLR2A	RNA polymerase II subunit A	-0.206
730229363	RALGAPA1	Ral GTPase activating protein catalytic alpha subunit 1	-0.206
55741502	ACAT2	acetyl-CoA acetyltransferase 2	-0.205
57164101	EPS15	epidermal growth factor receptor pathway substrate 15	-0.205
157821895	GDAP1	ganglioside induced differentiation associated protein 1	-0.205
568941844	IQSEC3	IQ motif and Sec7 domain 3	-0.205
148681975	NXPH1	neurexophilin 1	-0.205
6981166	PLAGL1	PLAG1 like zinc finger 1	-0.205
564301794	UBR3	ubiquitin protein ligase E3 component n-recognin 3 (putative)	-0.205
537236584	N/A	N/A	-0.205
209413778	GABRA2	gamma-aminobutyric acid type A receptor alpha2 subunit	-0.204
18266726	PAICS	phosphoribosylaminoimidazole carboxylase and phosphoribosylaminoimidazolesuccinocarboxamide synthase	-0.204
672026416	PRR36	proline rich 36	-0.204
62078785	Slc25a22	solute carrier family 25 member 22	-0.204
537235061	N/A	N/A	-0.204
6978723	CTSV	cathepsin V	-0.203
564370968	MAPK8IP3	mitogen-activated protein kinase 8 interacting protein 3	-0.203
13929124	PPIG	peptidylprolyl isomerase G	-0.203
672035153	PPP1R12C	protein phosphatase 1 regulatory subunit 12C	-0.203
149016230	ACSL3	acyl-CoA synthetase long-chain family member 3	-0.201
747019224	SRCAP	Snf2 related CREBBP activator protein	-0.201
460838694	Srrm2	serine/arginine repetitive matrix 2	-0.201
11560002	AMPH	amphiphysin	-0.200
6978699	CPD	carboxypeptidase D	-0.200

157817710	FER	FER tyrosine kinase	-0.200
158303294	GLS	glutaminase	-0.200
585652696	N/A	N/A	-0.200
672017118	N/A	N/A	-0.200
472381467	N/A	N/A	-0.199
537141312	N/A	N/A	-0.199
85861200	NARF	nuclear prelamin A recognition factor	-0.198
10946604	SEC61A2	Sec61 translocon alpha 2 subunit	-0.198
57770372	ATL1	atlastin GTPase 1	-0.197
113461996	COA5	cytochrome c oxidase assembly factor 5	-0.197
189163506	DNAJC10	DnaJ heat shock protein family (Hsp40) member C10	-0.197
564392854	KIAA1328	KIAA1328	-0.197
13489067	NSF	N-ethylmaleimide sensitive factor, vesicle fusing ATPase	-0.197
26328955	ETNK1	ethanolamine kinase 1	-0.196
157822919	GANAB	glucosidase II alpha subunit	-0.196
8393450	GLG1	golgi glycoprotein 1	-0.196
156139151	PDS5B	PDS5 cohesin associated factor B	-0.196
13929002	PFKM	phosphofructokinase, muscle	-0.196
564311487	LONRF2	LON peptidase N-terminal domain and ring finger 2	-0.195
672068926	N/A	N/A	-0.195
161760632	ACLY	ATP citrate lyase	-0.194
19705535	PACS1	phosphofurin acidic cluster sorting protein 1	-0.194
672042689	RAPGEF2	Rap guanine nucleotide exchange factor 2	-0.194
564355754	SNX13	sorting nexin 13	-0.194
524962788	N/A	N/A	-0.194
655898351	N/A	N/A	-0.194
564328626	HERC2	HECT and RLD domain containing E3 ubiquitin protein ligase 2	-0.193
9506947	PAK3	p21 (RAC1) activated kinase 3	-0.193
184160976	PRMT5	protein arginine methyltransferase 5	-0.193
672041603	N/A	N/A	-0.193
564379306	N/A	N/A	-0.193
26006191	ARHGEF2	Rho/Rac guanine nucleotide exchange factor 2	-0.192
760996272	EHBP1	EH domain binding protein 1	-0.192
564310645	PLXNB1	plexin B1	-0.192
29126232	SLCO3A1	solute carrier organic anion transporter family member 3A1	-0.192

880942118	N/A	N/A	-0.192
568981376	DIP2C	disco interacting protein 2 homolog C	-0.191
399124797	KIFC2	kinesin family member C2	-0.191
399567836	IDI1	isopentenyl-diphosphate delta isomerase 1	-0.190
300794237	LIMCH1	LIM and calponin homology domains 1	-0.190
148709219	UNC5A	unc-5 netrin receptor A	-0.190
149030541	N/A	N/A	-0.190
60360272	KLHL5	kelch like family member 5	-0.189
564389540	MGC116197 (includes others)	similar to RIKEN cDNA 1700001E04	-0.189
157817201	NETO1	neuropilin and tolloid like 1	-0.189
16758210	NUCB1	nucleobindin 1	-0.189
148698430	N/A	N/A	-0.189
300795842	CACHD1	cache domain containing 1	-0.188
71896543	SHANK3	SH3 and multiple ankyrin repeat domains 3	-0.188
19424342	ZHX1	zinc fingers and homeoboxes 1	-0.188
806704487	Palm2	paralemmin 2	-0.187
288806592	PRKCB	protein kinase C beta	-0.187
396941666	Dync1i2	dynein cytoplasmic 1 intermediate chain 2	-0.186
564346409	HIPK2	homeodomain interacting protein kinase 2	-0.186
404312657	NCAN	neurocan	-0.186
201066352	ANKRD6	ankyrin repeat domain 6	-0.185
115311606	MAPK3	mitogen-activated protein kinase 3	-0.185
52345385	PDIA6	protein disulfide isomerase family A member 6	-0.185
166063985	PKN1	protein kinase N1	-0.184
564337070	N/A	N/A	-0.184
149033803	CDKL2	cyclin dependent kinase like 2	-0.183
37360128	CNTNAP2	contactin associated protein-like 2	-0.183
564396135	KIAA0513	KIAA0513	-0.183
157816911	SNRNP48	small nuclear ribonucleoprotein U11/U12 subunit 48	-0.183
60360568	GRIA3	glutamate ionotropic receptor AMPA type subunit 3	-0.182
161760675	DNAJC14	DnaJ heat shock protein family (Hsp40) member C14	-0.181
51948398	SARAF	store-operated calcium entry associated regulatory factor	-0.181
241666396	CLK1	CDC like kinase 1	-0.180
124487463	GPR161	G protein-coupled receptor 161	-0.180
60360580	OGDH	oxoglutarate dehydrogenase	-0.180

157818589	TSPAN7	tetraspanin 7	-0.180
13928946	SMC1A	structural maintenance of chromosomes 1A	-0.179
6978543	ATP1A1	ATPase Na+/K+ transporting subunit alpha 1	-0.178
149069395	CUL7	cullin 7	-0.178
157823569	EXOC6B	exocyst complex component 6B	-0.178
29789269	GRIA1	glutamate ionotropic receptor AMPA type subunit 1	-0.178
198386334	KDM4B	lysine demethylase 4B	-0.178
953875962	N/A	N/A	-0.178
564377814	N/A	N/A	-0.178
282158057	ASTN1	astrotactin 1	-0.177
148695341	CTNND1	catenin delta 1	-0.177
73661200	SPRN	shadow of prion protein	-0.177
157820315	IPO7	importin 7	-0.176
11560079	KIT	KIT proto-oncogene receptor tyrosine kinase	-0.176
78042585	MAGED2	MAGE family member D2	-0.176
149067028	Ppp1r12a	protein phosphatase 1, regulatory subunit 12A	-0.176
674099552	N/A	N/A	-0.176
8393038	CAPN2	calpain 2	-0.175
242117994	KIAA0408	KIAA0408	-0.175
672080674	Myo16	myosin XVI	-0.175
76253845	PELP1	proline, glutamate and leucine rich protein 1	-0.175
564338482	SORT1	sortilin 1	-0.175
755550800	ADGRB1	adhesion G protein-coupled receptor B1	-0.174
11560055	KHDRBS3	KH RNA binding domain containing, signal transduction associated 3	-0.174
56605704	SERINC3	serine incorporator 3	-0.174
67078522	SLC39A6	solute carrier family 39 member 6	-0.174
9966775	NOTCH3	notch 3	-0.173
672026392	PNPLA6	patatin like phospholipase domain containing 6	-0.173
8392888	AKT2	AKT serine/threonine kinase 2	-0.172
149059533	NFIB	nuclear factor I B	-0.172
208973274	SRSF12	serine and arginine rich splicing factor 12	-0.172
27545420	TAOK1	TAO kinase 1	-0.172
672058640	N/A	N/A	-0.172
46485382	BHLHB9	basic helix-loop-helix family member b9	-0.171
14091754	GRIP1	glutamate receptor interacting protein 1	-0.170
50510837	KIAA1191	KIAA1191	-0.170
404501516	RBM14	RNA binding motif protein 14	-0.170
37360274	N/A	N/A	-0.170

402534517	EPB41L1	erythrocyte membrane protein band 4.1 like 1	-0.169
148673922	HSPH1	heat shock protein family H (Hsp110) member 1	-0.169
281485565	RASGRF1	Ras protein specific guanine nucleotide releasing factor 1	-0.169
989935538	N/A	N/A	-0.169
17864836	CACNA1C	calcium voltage-gated channel subunit alpha1 C	-0.168
157817420	NRIP3	nuclear receptor interacting protein 3	-0.168
403224975	SBF1	SET binding factor 1	-0.168
564400602	STAG2	stromal antigen 2	-0.168
564389912	N/A	N/A	-0.168
149052114	CACNA1H	calcium voltage-gated channel subunit alpha1 H	-0.167
13592131	DGKZ	diacylglycerol kinase zeta	-0.167
6978465	GRK2	G protein-coupled receptor kinase 2	-0.167
472359423	N/A	N/A	-0.167
695917192	Nacad	NAC alpha domain containing	-0.166
109505096	NID1	nidogen 1	-0.166
157820677	B4GALNT4	beta-1,4-N-acetyl-galactosaminyltransferase 4	-0.165
6978795	EEF2K	eukaryotic elongation factor 2 kinase	-0.165
283046651	PTPRZ1	protein tyrosine phosphatase, receptor type Z1	-0.165
14249130	LASP1	LIM and SH3 protein 1	-0.164
27806017	NCALD	neurocalcin delta	-0.164
157817708	PPP6R2	protein phosphatase 6 regulatory subunit 2	-0.164
19745186	CREB1	cAMP responsive element binding protein 1	-0.163
740086795	POLR2B	RNA polymerase II subunit B	-0.163
62654101	U2SURP	U2 snRNP associated SURP domain containing	-0.163
149032502	VPS41	VPS41, HOPS complex subunit	-0.163
392353562	ATP8A2	ATPase phospholipid transporting 8A2	-0.161
77415383	HSPA8	heat shock protein family A (Hsp70) member 8	-0.160
148671603	LRP11	LDL receptor related protein 11	-0.160
13929208	Scd2	stearoyl-Coenzyme A desaturase 2	-0.160
676282501	N/A	N/A	-0.160
62945282	C16orf62	chromosome 16 open reading frame 62	-0.159
6981200	MFGE8	milk fat globule-EGF factor 8 protein	-0.158
114145542	ARHGAP5	Rho GTPase activating protein 5	-0.157
564343665	HM13	histocompatibility minor 13	-0.157
564396924	PRRC2A	proline rich coiled-coil 2A	-0.157
19705545	RAB3IL1	RAB3A interacting protein like 1	-0.157
281306746	TFRC	transferrin receptor	-0.157
9507159	SYN1	synapsin I	-0.156

16758936	ABCC5	ATP binding cassette subfamily C member 5	-0.155
148672025	MAP3K12	mitogen-activated protein kinase kinase kinase 12	-0.155
6981352	PFKL	phosphofructokinase, liver type	-0.155
672071784	PI4KA	phosphatidylinositol 4-kinase alpha	-0.155
310688893	TNKS	tankyrase	-0.155
672044935	N/A	N/A	-0.155
6978755	DCC	DCC netrin 1 receptor	-0.154
568985444	CADPS	calcium dependent secretion activator	-0.153
568916417	N/A	N/A	-0.153
564347477	ZNF638	zinc finger protein 638	-0.152
214010196	DNMT1	DNA methyltransferase 1	-0.151
148747541	HNRNPU	heterogeneous nuclear ribonucleoprotein U	-0.150
734703982	SAFB2	scaffold attachment factor B2	-0.150
148669929	DRAM2	DNA damage regulated autophagy modulator 2	-0.149
56605814	PITPNM1	phosphatidylinositol transfer protein membrane associated 1	-0.149
208973288	PREX1	phosphatidylinositol-3,4,5-trisphosphate dependent Rac exchange factor 1	-0.149
149044802	USP34	ubiquitin specific peptidase 34	-0.149
157819455	VPS13D	vacuolar protein sorting 13 homolog D	-0.149
73532768	COPG1	coatomer protein complex subunit gamma 1	-0.148
209870013	ITSN1	intersectin 1	-0.148
300797536	MICAL3	microtubule associated monooxygenase, calponin and LIM domain containing 3	-0.148
148682872	MTOR	mechanistic target of rapamycin	-0.148
37359942	MTSS1	MTSS1, I-BAR domain containing	-0.147
148703340	SERTM1	serine rich and transmembrane domain containing 1	-0.146
564353622	UBR4	ubiquitin protein ligase E3 component n-recognin 4	-0.146
149043744	CABIN1	calcineurin binding protein 1	-0.144
148700348	ZMYND11	zinc finger MYND-type containing 11	-0.144
403225003	KCNQ5	potassium voltage-gated channel subfamily Q member 5	-0.142
157822435	PGM2L1	phosphoglucomutase 2 like 1	-0.142
564352958	C1orf216	chromosome 1 open reading frame 216	-0.141
40254785	LAMP2	lysosomal associated membrane protein 2	-0.141
45478142	LOC108353803	uncharacterized LOC108353803	-0.141
568910783	CDC42BPA	CDC42 binding protein kinase alpha	-0.140

145312253	REV3L	REV3 like, DNA directed polymerase zeta catalytic subunit	-0.139
568974914	SRCIN1	SRC kinase signaling inhibitor 1	-0.139
672059390	N/A	N/A	-0.139
672089019	N/A	N/A	-0.139
13540675	LRRN3	leucine rich repeat neuronal 3	-0.138
26023947	NRP1	neuropilin 1	-0.138
398650648	SLC8A1	solute carrier family 8 member A1	-0.138
938319566	LDHB	lactate dehydrogenase B	-0.137
52138628	RAP1B	RAP1B, member of RAS oncogene family	-0.137
537236381	N/A	N/A	-0.135
521032446	N/A	N/A	-0.135
259155312	AGAP2	ArfGAP with GTPase domain, ankyrin repeat and PH domain 2	-0.134
149017161	APC	APC, WNT signaling pathway regulator	-0.134
149018602	HYAL1	hyaluronoglucosaminidase 1	-0.134
300798434	TENM4	teneurin transmembrane protein 4	-0.134
6980956	GLUD1	glutamate dehydrogenase 1	-0.133
197927419	RNF14	ring finger protein 14	-0.133
672073809	N/A	N/A	-0.133
16758224	PPP1R9A	protein phosphatase 1 regulatory subunit 9A	-0.132
625184908	N/A	N/A	-0.132
399498531	NDRG2	NDRG family member 2	-0.131
744603535	N/A	N/A	-0.131
829910413	N/A	N/A	-0.130
35215315	Ccdc50	coiled-coil domain containing 50	-0.129
672053016	LOC691387	similar to HBxAg transactivated protein 2	-0.129
224960	N/A	N/A	-0.129
564364956	TFDP2	transcription factor Dp-2	-0.128
672068312	N/A	N/A	-0.128
224967068	PLCB1	phospholipase C beta 1	-0.127
564398462	Slc9a7	solute carrier family 9 member A7	-0.127
42821116	NCAM2	neural cell adhesion molecule 2	-0.126
940760818	N/A	N/A	-0.126
149050659	BIRC6	baculoviral IAP repeat containing 6	-0.125
672083507	LOC361346	similar to chromosome 18 open reading frame 54	-0.125
564369793	N/A	N/A	-0.125
47058982	SPTB	spectrin beta, erythrocytic	-0.124
675670628	N/A	N/A	-0.124

148747253	ATP1B1	ATPase Na+/K+ transporting subunit beta 1	-0.122
758818575	Peg3	paternally expressed 3	-0.122
12621130	SLIT1	slit guidance ligand 1	-0.120
157824053	PPFIA2	PTPRF interacting protein alpha 2	-0.119
564318589	N/A	N/A	-0.119
300794532	ARHGAP21	Rho GTPase activating protein 21	-0.118
253683488	NTRK2	neurotrophic receptor tyrosine kinase 2	-0.118
149023410	SNAP25	synaptosome associated protein 25	-0.118
209571464	USP9X	ubiquitin specific peptidase 9, X-linked	-0.118
672044124	N/A	N/A	-0.118
672049471	N/A	N/A	-0.117
13929102	APBA2	amyloid beta precursor protein binding family A member 2	-0.116
226958320	DENND5A	DENN domain containing 5A	-0.116
16758726	SLC17A7	solute carrier family 17 member 7	-0.116
564364792	SNAP91	synaptosome associated protein 91	-0.116
158711717	SNX27	sorting nexin family member 27	-0.116
148677026	EPM2AIP1	EPM2A interacting protein 1	-0.115
149053570	WSB1	WD repeat and SOCS box containing 1	-0.115
148666837	MGLL	monoglyceride lipase	-0.114
209915579	TMX4	thioredoxin related transmembrane protein 4	-0.113
198278523	AP3D1	adaptor related protein complex 3 delta 1 subunit	-0.112
149043706	PRMT2	protein arginine methyltransferase 2	-0.112
674077166	N/A	N/A	-0.112
672073607	N/A	N/A	-0.112
6756037	YWHAH	tyrosine 3-monooxygenase/tryptophan 5-monooxygenase activation protein eta	-0.111
672088045	N/A	N/A	-0.111
815890893	CD81	CD81 molecule	-0.110
149032539	HECW1	HECT, C2 and WW domain containing E3 ubiquitin protein ligase 1	-0.110
164519074	PDE4D	phosphodiesterase 4D	-0.110
640831572	N/A	N/A	-0.110
13786196	ATRN	attractin	-0.109
25282419	CANX	calnexin	-0.107
157822407	MAST2	microtubule associated serine/threonine kinase 2	-0.107
148695277	SESTD1	SEC14 and spectrin domain containing 1	-0.107
77627981	SHANK1	SH3 and multiple ankyrin repeat domains 1	-0.107

17985955	Dclk1	doublecortin-like kinase 1	-0.105
672054406	N/A	N/A	-0.103
672063358	CLASP2	cytoplasmic linker associated protein 2	-0.101
149020473	SMARCA4	SWI/SNF related, matrix associated, actin dependent regulator of chromatin, subfamily a, member 4	-0.101
29789307	KIF1B	kinesin family member 1B	-0.098
564355241	NCOA1	nuclear receptor coactivator 1	-0.088
149055603	N/A	N/A	-0.087
148694359	MYO5A	myosin VA	-0.086
564379895	SEZ6L	seizure related 6 homolog like	-0.086
20301986	Gpm6b	glycoprotein m6b	-0.085
298231227	PSAP	prosaposin	-0.083
38372401	NRCAM	neuronal cell adhesion molecule	-0.080
148706565	N/A	N/A	-0.079
148704375	PABPN1	poly(A) binding protein nuclear 1	-0.076
880889979	N/A	N/A	-0.076
744542371	N/A	N/A	-0.074
149022387	NCKAP1	NCK associated protein 1	-0.073
124286791	CELF2	CUGBP Elav-like family member 2	-0.069
148679159	GNAO1	G protein subunit alpha o1	-0.069
148692349	ATP1A3	ATPase Na+/K+ transporting subunit alpha 3	-0.064
55249691	CPE	carboxypeptidase E	-0.060
148673176	FABP7	fatty acid binding protein 7	-0.057
149047906	STRBP	spermatid perinuclear RNA binding protein	-0.055
40254595	DPYSL2	dihydropyrimidinase like 2	-0.051
676279510	N/A	N/A	-0.049
825725	SNRNP70	small nuclear ribonucleoprotein U1 subunit 70	-0.046
545838484	N/A	N/A	-0.045
568955606	INTS10	integrator complex subunit 10	-0.044
28189917	Ubb	ubiquitin B	-0.042
564351830	N/A	N/A	-0.040
158749620	MAP1B	microtubule associated protein 1B	-0.033
744617549	N/A	N/A	-0.028
564392534	Gpr137b	G protein-coupled receptor 137B	-0.024
57634518	SEPT11	septin 11	-0.013
2266994	OGT	O-linked N-acetylglucosamine (GlcNAc) transferase	-0.008
12667448	Syt7	synaptotagmin 7	0.005

157819423	SPSB3	splA/ryanodine receptor domain and SOCS box containing 3	0.009
62087776	SYNCRIP	synaptotagmin binding cytoplasmic RNA interacting protein	0.017
16758950	CSNK1A1	casein kinase 1 alpha 1	0.025
255291787	DBN1	drebrin 1	0.027
148683063	GNB1	G protein subunit beta 1	0.027
564352872	N/A	N/A	0.031
57526804	SUB1	SUB1 homolog, transcriptional regulator	0.036
157820391	TULP4	tubby like protein 4	0.038
50510503	SH3GLB1	SH3 domain containing GRB2 like, endophilin B1	0.041
13242233	STMN3	stathmin 3	0.048
564391098	SSR1	signal sequence receptor subunit 1	0.049
16758674	CSNK2A1	casein kinase 2 alpha 1	0.050
672070615	TTC3	tetratricopeptide repeat domain 3	0.050
14165437	HNRNPK	heterogeneous nuclear ribonucleoprotein K	0.052
586556217	N/A	N/A	0.056
12711692	DPYSL5	dihydropyrimidinase like 5	0.057
7305363	PAFAH1B1	platelet activating factor acetylhydrolase 1b regulatory subunit 1	0.060
22129759	ZWINT	ZW10 interacting kinetochore protein	0.061
998662027	N/A	N/A	0.061
28467005	HSP90AA1	heat shock protein 90 alpha family class A member 1	0.063
585192925	N/A	N/A	0.063
585645604	N/A	N/A	0.063
87299586	Fnbp1l	formin binding protein 1-like	0.064
407728599	CTNND2	catenin delta 2	0.066
8394158	FASN	fatty acid synthase	0.068
347300280	CLSTN1	calsyntenin 1	0.070
157820133	TTYH3	tweety family member 3	0.073
564398411	N/A	N/A	0.076
158303318	MAPT	microtubule associated protein tau	0.077
826351127	N/A	N/A	0.081
560954566	N/A	N/A	0.081
913517850	N/A	N/A	0.082
13786162	CDH2	cadherin 2	0.083
8393296	EEF2	eukaryotic translation elongation factor 2	0.084
17978461	BRINP1	BMP/retinoic acid inducible neural specific 1	0.087

820983550	N/A	N/A	0.087
51948418	EEF1G	eukaryotic translation elongation factor 1 gamma	0.088
149049455	Ptms	parathymosin	0.088
4503529	EIF4A1	eukaryotic translation initiation factor 4A1	0.089
18543359	MARK3	microtubule affinity regulating kinase 3	0.089
6978449	ADD2	adducin 2	0.093
157821213	KIF21B	kinesin family member 21B	0.095
189095264	UBTF	upstream binding transcription factor, RNA polymerase I	0.095
18677765	PALM	paralemmin	0.096
37359970	SDC3	syndecan 3	0.098
114145618	MIS12	MIS12, kinetochore complex component	0.099
281371335	WDR6	WD repeat domain 6	0.100
25742568	DPYSL3	dihydropyrimidinase like 3	0.101
12004976	NDFIP1	Nedd4 family interacting protein 1	0.101
12850126	STMN2	stathmin 2	0.101
528762703	N/A	N/A	0.101
672055757	N/A	N/A	0.101
149028085	BAG6	BCL2 associated athanogene 6	0.102
61889073	MATR3	matrin 3	0.102
511905488	N/A	N/A	0.103
676280076	N/A	N/A	0.103
74210167	DDX5	DEAD-box helicase 5	0.105
15126683	DNAJC5	DnaJ heat shock protein family (Hsp40) member C5	0.105
51172604	JPH4	junctophilin 4	0.105
62078555	MLLT11	myeloid/lymphoid or mixed-lineage leukemia; translocated to, 11	0.106
564301352	PRRC2B	proline rich coiled-coil 2B	0.106
704584573	N/A	N/A	0.106
148672704	CSNK1E	casein kinase 1 epsilon	0.109
242397499	DCHS1	dachsous cadherin-related 1	0.109
261878588	WDR13	WD repeat domain 13	0.110
537149923	N/A	N/A	0.110
93352570	ATCAY	ATCAY, caytaxin	0.111
58865558	TUBA1C	tubulin alpha 1c	0.111
157817085	ADGRB2	adhesion G protein-coupled receptor B2	0.113
148702066	N/A	N/A	0.113
12861068	CFL1	cofilin 1	0.114

564363529	NCAM1	neural cell adhesion molecule 1	0.116
507546779	N/A	N/A	0.116
148227578	ITFG1	integrin alpha FG-GAP repeat containing 1	0.117
149063507	PEBP1	phosphatidylethanolamine binding protein 1	0.117
120538101	RTN3	reticulon 3	0.117
13929006	RAB2A	RAB2A, member RAS oncogene family	0.118
926720695	N/A	N/A	0.118
74212223	RPL8	ribosomal protein L8	0.119
672068062	ARF1	ADP ribosylation factor 1	0.120
755524534	ARHGEF7	Rho guanine nucleotide exchange factor 7	0.121
344252784	N/A	N/A	0.121
755492511	MAP4K4	mitogen-activated protein kinase kinase kinase kinase 4	0.122
66730447	NUDT3	nudix hydrolase 3	0.123
672047825	N/A	N/A	0.123
554543965	N/A	N/A	0.123
74145569	BNIP2	BCL2 interacting protein 2	0.124
347361005	USP22	ubiquitin specific peptidase 22	0.124
564301979	CKAP5	cytoskeleton associated protein 5	0.125
675970145	N/A	N/A	0.127
987937084	N/A	N/A	0.127
725607246	N/A	N/A	0.128
157817558	JAKMIP2	janus kinase and microtubule interacting protein 2	0.129
244792650	TNIK	TRAF2 and NCK interacting kinase	0.129
149065002	PHF14	PHD finger protein 14	0.130
70778952	RAD23B	RAD23 homolog B, nucleotide excision repair protein	0.130
564346502	N/A	N/A	0.130
27465617	ABI2	abl interactor 2	0.131
149046410	ARHGEF4	Rho guanine nucleotide exchange factor 4	0.131
58865642	PAQR3	progestin and adipoQ receptor family member 3	0.131
672082099	N/A	N/A	0.131
171846760	HDAC2	histone deacetylase 2	0.132
12408334	NOLC1	nucleolar and coiled-body phosphoprotein 1	0.132
564326930	MAP3K10	mitogen-activated protein kinase kinase kinase 10	0.133
987926417	N/A	N/A	0.133
157824115	AGAP3	ArfGAP with GTPase domain, ankyrin repeat and PH domain 3	0.134

76253725	CCT6A	chaperonin containing TCP1 subunit 6A	0.134
201066380	FSCN1	fascin actin-bundling protein 1	0.134
149042824	UBE2V1	ubiquitin conjugating enzyme E2 V1	0.134
672057722	N/A	N/A	0.134
537242782	N/A	N/A	0.134
83582792	FAM117B	family with sequence similarity 117 member B	0.135
29336093	Tpm3	tropomyosin 3	0.135
148666815	CNBP	CCHC-type zinc finger nucleic acid binding protein	0.136
148699288	N/A	N/A	0.136
281340051	N/A	N/A	0.137
625259717	N/A	N/A	0.137
351710149	N/A	N/A	0.137
926686110	N/A	N/A	0.138
157818315	CUL3	cullin 3	0.139
672045791	Tlk1	tousled-like kinase 1	0.139
803119291	N/A	N/A	0.140
755756742	N/A	N/A	0.140
528767051	N/A	N/A	0.140
564305145	NOL6	nucleolar protein 6	0.142
564309116	RTL6	retrotransposon Gag like 6	0.142
444730664	N/A	N/A	0.142
521031535	N/A	N/A	0.142
164663868	RGMA	repulsive guidance molecule family member a	0.143
119351041	SYNRG	synergin gamma	0.143
859916344	N/A	N/A	0.143
431895484	N/A	N/A	0.143
686735522	N/A	N/A	0.143
564364568	ARPP19	cAMP regulated phosphoprotein 19	0.144
148697875	GDI1	GDP dissociation inhibitor 1	0.144
564328896	CHD2	chromodomain helicase DNA binding protein 2	0.145
672035558	NOVA2	NOVA alternative splicing regulator 2	0.145
672068548	SUPT6H	SPT6 homolog, histone chaperone	0.145
955507084	N/A	N/A	0.145
514460070	N/A	N/A	0.145
18959272	KCNQ2	potassium voltage-gated channel subfamily Q member 2	0.146
158081759	PRKAR2B	protein kinase cAMP-dependent type II regulatory subunit beta	0.146
880945834	N/A	N/A	0.146

625274124	N/A	N/A	0.146
564331017	Fbxl19	F-box and leucine-rich repeat protein 19	0.147
157820255	MED13	mediator complex subunit 13	0.147
157817340	Ube2l3	ubiquitin-conjugating enzyme E2L 3	0.148
84781731	ZDHHC3	zinc finger DHHC-type containing 3	0.148
664746285	N/A	N/A	0.148
158341666	SEL1L	SEL1L ERAD E3 ligase adaptor subunit	0.149
970727843	N/A	N/A	0.149
589929864	N/A	N/A	0.149
28174920	RPL17	ribosomal protein L17	0.151
240120166	STRN4	striatin 4	0.151
351698877	N/A	N/A	0.151
672075032	MARK1	microtubule affinity regulating kinase 1	0.152
829969914	N/A	N/A	0.152
664777237	N/A	N/A	0.152
23956194	ARL8A	ADP ribosylation factor like GTPase 8A	0.153
672050419	SLC6A6	solute carrier family 6 member 6	0.153
68163407	SAMD14	sterile alpha motif domain containing 14	0.154
564373361	SEZ6	seizure related 6 homolog	0.154
55741787	STX7	syntaxin 7	0.154
393716310	WAC	WW domain containing adaptor with coiled-coil	0.154
148688660	FEZF2	FEZ family zinc finger 2	0.155
157819885	SETD5	SET domain containing 5	0.155
403310688	USP24	ubiquitin specific peptidase 24	0.155
821024661	N/A	N/A	0.155
672046732	N/A	N/A	0.155
51890219	CCT5	chaperonin containing TCP1 subunit 5	0.156
254281247	EVL	Enah/Vasp-like	0.156
300793740	TANC2	tetratricopeptide repeat, ankyrin repeat and coiled-coil containing 2	0.156
803119291	N/A	N/A	0.157
149047683	N/A	N/A	0.157
18266706	IKBKAP	inhibitor of kappa light polypeptide gene enhancer in B-cells, kinase complex-associated protein	0.158
33563266	NDUFA4	NDUFA4, mitochondrial complex associated	0.158
8394209	Rpl29 (includes others)	ribosomal protein L29	0.158
884928897	N/A	N/A	0.158

157819223	KIAA1324L	KIAA1324 like	0.159
28972173	KIF3B	kinesin family member 3B	0.159
149033397	DFNA5	DFNA5, deafness associated tumor suppressor	0.160
148680533	EIF5A	eukaryotic translation initiation factor 5A	0.160
431921423	N/A	N/A	0.160
625206686	N/A	N/A	0.160
139947663	NCDN	neurochondrin	0.161
947231308	N/A	N/A	0.161
672082436	N/A	N/A	0.161
987959513	N/A	N/A	0.162
565318441	N/A	N/A	0.162
14010879	PSMD1	proteasome 26S subunit, non-ATPase 1	0.163
926689000	N/A	N/A	0.163
291049776	IMPAD1	inositol monophosphatase domain containing 1	0.164
12861758	LY6H	lymphocyte antigen 6 family member H	0.165
149016331	NCL	nucleolin	0.165
13928824	YWHAE	tyrosine 3-monooxygenase/tryptophan 5-monooxygenase activation protein epsilon	0.165
281427141	ZMIZ2	zinc finger MIZ-type containing 2	0.165
402794806	GARS	glycyl-tRNA synthetase	0.166
27465567	HSBP1	heat shock factor binding protein 1	0.166
60359978	KIF3C	kinesin family member 3C	0.166
209447111	PDZD4	PDZ domain containing 4	0.166
148696233	TMEM127	transmembrane protein 127	0.166
564311321	N/A	N/A	0.166
568921780	EIF4E	eukaryotic translation initiation factor 4E	0.167
667481282	N/A	N/A	0.167
293651541	LRRN2	leucine rich repeat neuronal 2	0.168
148666954	RYBP	RING1 and YY1 binding protein	0.168
537127177	N/A	N/A	0.168
112983826	KIAA0895L	KIAA0895 like	0.170
149056919	SELENOW	selenoprotein W	0.170
655889416	N/A	N/A	0.170
795561827	N/A	N/A	0.170
9910214	EIF5	eukaryotic translation initiation factor 5	0.171
197245939	HNRNPL	heterogeneous nuclear ribonucleoprotein L	0.171
8394021	PPP2CB	protein phosphatase 2 catalytic subunit beta	0.171
8394432	PRDX2	peroxiredoxin 2	0.171
568972243	PRKAR1A	protein kinase cAMP-dependent type I regulatory subunit alpha	0.171

33636726	SERINC1	serine incorporator 1	0.171
672067122	N/A	N/A	0.171
149063095	EIF4H	eukaryotic translation initiation factor 4H	0.172
30851559	MAPRE1	microtubule associated protein RP/EB family member 1	0.172
293675	SRSF2	serine and arginine rich splicing factor 2	0.172
545215595	N/A	N/A	0.172
26329593	C11orf87	chromosome 11 open reading frame 87	0.173
171543899	PLXNA4	plexin A4	0.173
148692593	PTGES3	prostaglandin E synthase 3	0.173
672050411	RAB11FIP5	RAB11 family interacting protein 5	0.173
42476292	TALDO1	transaldolase 1	0.173
157819279	TNPO3	transportin 3	0.173
926685845	N/A	N/A	0.173
635012684	N/A	N/A	0.173
23271707	EIF3B	eukaryotic translation initiation factor 3 subunit B	0.174
31560202	TMEM246	transmembrane protein 246	0.174
13994121	FEZ1	fasciculation and elongation protein zeta 1	0.175
209870077	TRIM28	tripartite motif containing 28	0.175
149046938	N/A	N/A	0.175
284005558	N/A	N/A	0.175
562821651	N/A	N/A	0.175
76160821	DNAJC18	DnaJ heat shock protein family (Hsp40) member C18	0.176
148667025	LHFPL4	lipoma HMGIC fusion partner-like 4	0.176
564358123	MIDN	midnolin	0.176
51948430	NPDC1	neural proliferation, differentiation and control 1	0.176
744598167	N/A	N/A	0.176
22024394	FABP5	fatty acid binding protein 5	0.177
564383487	SLAIN2	SLAIN motif family member 2	0.177
655660415	N/A	N/A	0.178
565318824	N/A	N/A	0.178
564352364	N/A	N/A	0.178
396080328	ADCYAP1R1	ADCYAP receptor type I	0.179
755562682	C11orf95	chromosome 11 open reading frame 95	0.179
13928922	STX6	syntaxin 6	0.179
293340917	C3orf70	chromosome 3 open reading frame 70	0.180
148704795	CFL2	cofilin 2	0.180

26346731	UBE2Z	ubiquitin conjugating enzyme E2 Z	0.180
68341973	WASF1	WAS protein family member 1	0.180
611991798	N/A	N/A	0.180
594092878	N/A	N/A	0.180
158711738	MICU2	mitochondrial calcium uptake 2	0.181
564377243	OPA1	OPA1, mitochondrial dynamin like GTPase	0.181
965930210	N/A	N/A	0.181
16758036	RPL21	ribosomal protein L21	0.182
67078426	SPIN1	spindlin 1	0.182
40786451	EIF2S2	eukaryotic translation initiation factor 2 subunit beta	0.183
149047559	MTMR3	myotubularin related protein 3	0.183
507693337	N/A	N/A	0.183
823419445	N/A	N/A	0.183
431899854	N/A	N/A	0.183
171846592	GPBP1	GC-rich promoter binding protein 1	0.184
149054120	ORMDL3	ORMDL sphingolipid biosynthesis regulator 3	0.184
672054841	RCC2	regulator of chromosome condensation 2	0.184
767996256	FBXL20	F-box and leucine rich repeat protein 20	0.185
86477155	PER1	period circadian clock 1	0.185
829944637	N/A	N/A	0.185
60360108	BRD2	bromodomain containing 2	0.186
795270501	N/A	N/A	0.186
640823833	N/A	N/A	0.186
589927407	N/A	N/A	0.186
198278505	RPL7	ribosomal protein L7	0.187
149069422	RPL7L1	ribosomal protein L7 like 1	0.187
924442952	SPTBN2	spectrin beta, non-erythrocytic 2	0.187
293343541	ICE1	interactor of little elongation complex ELL subunit 1	0.188
16758782	LMNB1	lamin B1	0.188
820987686	N/A	N/A	0.188
148702333	DDX42	DEAD-box helicase 42	0.189
148666026	FAM49A	family with sequence similarity 49 member A	0.189
13591985	MIF	macrophage migration inhibitory factor (glycosylation-inhibiting factor)	0.189
6753130	NEUROD6	neuronal differentiation 6	0.189
148708155	N/A	N/A	0.189
537141832	N/A	N/A	0.189
70794762	HARS	histidyl-tRNA synthetase	0.190

50510427	IP6K1	inositol hexakisphosphate kinase 1	0.190
149018452	SMARCC1	SWI/SNF related, matrix associated, actin dependent regulator of chromatin subfamily c member 1	0.190
859770113	N/A	N/A	0.190
8393415	GAP43	growth associated protein 43	0.191
404501464	IFNAR1	interferon alpha and beta receptor subunit 1	0.191
157823419	PRAG1	PEAK1 related kinase activating pseudokinase 1	0.191
56550075	PSMA7	proteasome subunit alpha 7	0.191
157821581	PSMD13	proteasome 26S subunit, non-ATPase 13	0.191
58865776	TRIM32	tripartite motif containing 32	0.191
148705826	UCHL1	ubiquitin C-terminal hydrolase L1	0.191
823419836	N/A	N/A	0.191
940716821	N/A	N/A	0.191
564376494	N/A	N/A	0.191
76008363	BICD2	BICD cargo adaptor 2	0.192
162287391	RPL6	ribosomal protein L6	0.192
674086663	N/A	N/A	0.192
585638696	N/A	N/A	0.192
270483894	CAMSAP1	calmodulin regulated spectrin associated protein 1	0.193
157818643	KCTD3	potassium channel tetramerization domain containing 3	0.193
16758298	PSMB7	proteasome subunit beta 7	0.193
564319816	N/A	N/A	0.193
208973284	MAP9	microtubule associated protein 9	0.194
564361208	SCUBE1	signal peptide, CUB domain and EGF like domain containing 1	0.194
795537196	N/A	N/A	0.194
564367862	Dst	dystonin	0.195
157817789	DUSP8	dual specificity phosphatase 8	0.195
564359050	NUDT4	nudix hydrolase 4	0.195
402743472	SLC3A2	solute carrier family 3 member 2	0.195
18699726	VPS4A	vacuolar protein sorting 4 homolog A	0.195
755785705	N/A	N/A	0.195
440890867	N/A	N/A	0.195
725571770	N/A	N/A	0.195
148706791	CELF3	CUGBP Elav-like family member 3	0.196
672042487	NBEA	neurobeachin	0.196

148682476	PTP4A1	protein tyrosine phosphatase type IVA, member 1	0.196
224451084	GPSM1	G protein signaling modulator 1	0.197
157819591	PARP6	poly(ADP-ribose) polymerase family member 6	0.197
295789408	N/A	N/A	0.197
77736544	COX6A1	cytochrome c oxidase subunit 6A1	0.198
10720132	NEO1	neogenin 1	0.198
564398139	FYN	FYN proto-oncogene, Src family tyrosine kinase	0.199
564305239	TMEM8B	transmembrane protein 8B	0.199
625183267	N/A	N/A	0.199
444706073	N/A	N/A	0.199
148675893	LGALSL	galectin like	0.200
119612238	ZNF706	zinc finger protein 706	0.200
817328905	N/A	N/A	0.200
677283307	AZIN1	antizyme inhibitor 1	0.201
187960160	BRSK1	BR serine/threonine kinase 1	0.201
109504669	CDHR2	cadherin related family member 2	0.201
54400738	CHPF	chondroitin polymerizing factor	0.201
672045157	PRPF40A	pre-mRNA processing factor 40 homolog A	0.201
672082389	N/A	N/A	0.201
403263938	N/A	N/A	0.201
26006223	FBXO21	F-box protein 21	0.202
84000579	FTL	ferritin light chain	0.202
694879429	ADGRL2	adhesion G protein-coupled receptor L2	0.203
157823607	ALDH18A1	aldehyde dehydrogenase 18 family member A1	0.203
158186685	RAB12	RAB12, member RAS oncogene family	0.203
795271913	N/A	N/A	0.204
16758310	LRP3	LDL receptor related protein 3	0.205
148689223	MANF	mesencephalic astrocyte derived neurotrophic factor	0.205
197927410	SETBP1	SET binding protein 1	0.205
13385526	ZMYND19	zinc finger MYND-type containing 19	0.205
671001540	N/A	N/A	0.205
564381466	N/A	N/A	0.205
2852640	ERI3	ERI1 exoribonuclease family member 3	0.206
672044181	HS2ST1	heparan sulfate 2-O-sulfotransferase 1	0.206
281485586	MAP3K7	mitogen-activated protein kinase kinase kinase 7	0.206
11120704	NOP58	NOP58 ribonucleoprotein	0.206

148664717	PAIP2	poly(A) binding protein interacting protein 2	0.206
625258416	N/A	N/A	0.206
743729098	N/A	N/A	0.206
56549649	NFIX	nuclear factor I X	0.207
655875254	N/A	N/A	0.207
966647643	N/A	N/A	0.207
148705576	CRMP1	collapsin response mediator protein 1	0.208
213688408	LINGO1	leucine rich repeat and Ig domain containing 1	0.208
157823005	RAB11FIP4	RAB11 family interacting protein 4	0.208
56605632	RNMT	RNA guanine-7 methyltransferase	0.208
12852725	SPPL3	signal peptide peptidase like 3	0.208
431917236	N/A	N/A	0.208
431917236	N/A	N/A	0.208
672015267	N/A	N/A	0.208
157823775	PHC1	polyhomeotic homolog 1	0.209
76096332	SREBF2	sterol regulatory element binding transcription factor 2	0.209
564386624	AMER2	APC membrane recruitment protein 2	0.210
158508473	FAM69B	family with sequence similarity 69 member B	0.210
47059187	GNL1	G protein nucleolar 1 (putative)	0.210
253683473	METTL9	methyltransferase like 9	0.210
148707518	RNF2	ring finger protein 2	0.210
68533845	UBE2E1	ubiquitin conjugating enzyme E2 E1	0.210
33356154	UBE2H	ubiquitin conjugating enzyme E2 H	0.210
157820283	NYAP1	neuronal tyrosine phosphorylated phosphoinositide-3-kinase adaptor 1	0.211
29788994	TMEM132A	transmembrane protein 132A	0.211
332245592	N/A	N/A	0.211
672055149	N/A	N/A	0.211
926725899	N/A	N/A	0.211
491668487	N/A	N/A	0.212
149048659	N/A	N/A	0.212
58865604	DIRC2	disrupted in renal carcinoma 2	0.213
47155561	DNAJC7	DnaJ heat shock protein family (Hsp40) member C7	0.213
762006019	FAM8A1	family with sequence similarity 8 member A1	0.214
13507268	MLLT3	MLLT3, super elongation complex subunit	0.214
926691741	N/A	N/A	0.214
55715816	GLYR1	glyoxylate reductase 1 homolog	0.215
58865976	KLHDC3	kelch domain containing 3	0.215

188595675	RFX7	regulatory factor X7	0.215
672085227	USP10	ubiquitin specific peptidase 10	0.215
19344056	MCRIP1	MAPK regulated corepressor interacting protein 1	0.216
189163477	SCAF4	SR-related CTD associated factor 4	0.216
743742979	N/A	N/A	0.216
672053690	N/A	N/A	0.216
564393951	MBD1	methyl-CpG binding domain protein 1	0.217
148707748	NUCKS1	nuclear casein kinase and cyclin dependent kinase substrate 1	0.217
58865512	STRAP	serine/threonine kinase receptor associated protein	0.217
157819739	PODXL2	podocalyxin like 2	0.218
955478861	N/A	N/A	0.218
148670589	RAB5C	RAB5C, member RAS oncogene family	0.219
119628283	TMEM57	transmembrane protein 57	0.219
157822501	MCM3AP	minichromosome maintenance complex component 3 associated protein	0.220
30017419	NREP	neuronal regeneration related protein	0.220
8394405	SLC7A5	solute carrier family 7 member 5	0.220
25742623	UGCG	UDP-glucose ceramide glucosyltransferase	0.220
2804296	CDH8	cadherin 8	0.221
59709464	PIK3R2	phosphoinositide-3-kinase regulatory subunit 2	0.221
655872066	N/A	N/A	0.221
300798499	AFF3	AF4/FMR2 family member 3	0.222
54400718	GHITM	growth hormone inducible transmembrane protein	0.222
67078422	TMX1	thioredoxin related transmembrane protein 1	0.222
589941671	N/A	N/A	0.222
157786926	ARPC3	actin related protein 2/3 complex subunit 3	0.223
545490388	ZMAT2	zinc finger matrin-type 2	0.223
61556823	RBM17	RNA binding motif protein 17	0.224
148692189	SAMD4B	sterile alpha motif domain containing 4B	0.224
61556748	TSPYL1	TSPY like 1	0.224
586975096	N/A	N/A	0.224
13540661	BMPR1A	bone morphogenetic protein receptor type 1A	0.225
148674168	DYNLRB1	dynein light chain roadblock-type 1	0.225
827475660	EPC1	enhancer of polycomb homolog 1	0.225
109150410	SH3BP5	SH3 domain binding protein 5	0.225
655698052	N/A	N/A	0.225

197927166	AGPAT5	1-acylglycerol-3-phosphate O-acyltransferase 5	0.226
404312665	DKK3	dickkopf WNT signaling pathway inhibitor 3	0.226
56090568	FBXO30	F-box protein 30	0.226
21703344	SAR1A	secretion associated Ras related GTPase 1A	0.226
5870130	SNRPB	small nuclear ribonucleoprotein polypeptides B and B1	0.226
564315719	N/A	N/A	0.226
83267872	ADNP	activity dependent neuroprotector homeobox	0.227
187469679	LDB1	LIM domain binding 1	0.227
21489987	PCYOX1	prenylcysteine oxidase 1	0.227
640823357	N/A	N/A	0.227
488534205	N/A	N/A	0.227
672036872	PRR12	proline rich 12	0.228
8394221	Rps3a1	ribosomal protein S3A1	0.228
403225023	BRAP	BRCA1 associated protein	0.229
157822985	CEBPZ	CCAAT/enhancer binding protein zeta	0.229
57164107	NIPSNAP3A	nipsnap homolog 3A	0.229
755537448	N/A	N/A	0.229
537145497	N/A	N/A	0.229
189491871	B3GAT3	beta-1,3-glucuronyltransferase 3	0.230
154426327	KANSL2	KAT8 regulatory NSL complex subunit 2	0.230
42627759	SMC3	structural maintenance of chromosomes 3	0.230
300796772	TMEM151B	transmembrane protein 151B	0.230
58865626	UBXN4	UBX domain protein 4	0.230
13928966	HSF2	heat shock transcription factor 2	0.231
213688411	LPCAT1	lysophosphatidylcholine acyltransferase 1	0.231
537151504	N/A	N/A	0.232
958756481	N/A	N/A	0.232
277349626	AATK	apoptosis associated tyrosine kinase	0.233
403224991	MAP3K9	mitogen-activated protein kinase kinase kinase 9	0.233
13929130	SLC12A2	solute carrier family 12 member 2	0.233
11559976	EXOC5	exocyst complex component 5	0.234
157817418	NCK2	NCK adaptor protein 2	0.234
521020666	N/A	N/A	0.234
564386622	AMER2	APC membrane recruitment protein 2	0.235
569000962	CRIM1	cysteine rich transmembrane BMP regulator 1	0.236
68341995	NDUFS4	NADH:ubiquinone oxidoreductase subunit S4	0.236
62079005	SLAIN1	SLAIN motif family member 1	0.236
731286412	N/A	N/A	0.236

56540888	ATG12	autophagy related 12	0.237
392333349	ELP3	elongator acetyltransferase complex subunit 3	0.237
149044496	PLAA	phospholipase A2 activating protein	0.237
71043834	RBMX	RNA binding motif protein, X-linked	0.237
149024496	SPEN	spen family transcriptional repressor	0.237
114051946	YTHDF2	YTH N6-methyladenosine RNA binding protein 2	0.237
27229298	ELAVL3	ELAV like RNA binding protein 3	0.238
672041858	PKIA	cAMP-dependent protein kinase inhibitor alpha	0.238
28972780	TLE3	transducin like enhancer of split 3	0.238
942047300	N/A	N/A	0.238
60360118	FAM168B	family with sequence similarity 168 member B	0.239
61557199	HES6	hes family bHLH transcription factor 6	0.239
77415381	PDAP1	PDGFA associated protein 1	0.239
401709959	Ppp1cc	protein phosphatase 1, catalytic subunit, gamma isoform	0.239
157787066	SLC29A4	solute carrier family 29 member 4	0.239
674077614	N/A	N/A	0.239
158138509	DNAJC2	DnaJ heat shock protein family (Hsp40) member C2	0.240
51036650	MCL1	MCL1, BCL2 family apoptosis regulator	0.240
157817763	NEK9	NIMA related kinase 9	0.240
4506681	RPS11	ribosomal protein S11	0.240
66730335	SUMO3	small ubiquitin-like modifier 3	0.240
29612542	H2AFZ	H2A histone family member Z	0.241
157821927	XPR1	xenotropic and polytropic retrovirus receptor 1	0.241
85541051	PDXP	pyridoxal phosphatase	0.242
198442823	PRCC	papillary renal cell carcinoma (translocation-associated)	0.242
641719431	N/A	N/A	0.242
213417659	KITLG	KIT ligand	0.243
564380021	TTC28	tetratricopeptide repeat domain 28	0.243
926714902	N/A	N/A	0.243
443906726	DLGAP3	DLG associated protein 3	0.245
157823165	DNAJB1	DnaJ heat shock protein family (Hsp40) member B1	0.245
39850096	RPS16	ribosomal protein S16	0.245
6678315	TSC22D1	TSC22 domain family member 1	0.245
187937028	NDUFB9	NADH:ubiquinone oxidoreductase subunit B9	0.246
672069253	SOCS7	suppressor of cytokine signaling 7	0.246

296491570	N/A	N/A	0.246
765099233	LMNB2	lamin B2	0.247
281371452	ORAI2	ORAI calcium release-activated calcium modulator 2	0.247
157820401	ABHD2	abhydrolase domain containing 2	0.248
148664543	RNF138	ring finger protein 138	0.249
823430370	N/A	N/A	0.249
18266682	CDK5	cyclin dependent kinase 5	0.250
13929008	CLNS1A	chloride nucleotide-sensitive channel 1A	0.250
564345074	N/A	N/A	0.250
53850606	IK	IK cytokine, down-regulator of HLA II	0.251
148696021	N/A	N/A	0.251
23263334	LZTS1	leucine zipper tumor suppressor 1	0.252
9507007	PTGFRN	prostaglandin F2 receptor inhibitor	0.252
676260928	N/A	N/A	0.252
28972574	CDK19	cyclin dependent kinase 19	0.253
157820509	KCTD15	potassium channel tetramerization domain containing 15	0.253
148707931	RNF152	ring finger protein 152	0.253
145558904	EML1	echinoderm microtubule associated protein like 1	0.254
74216801	RNF4	ring finger protein 4	0.254
344245515	N/A	N/A	0.254
676272934	N/A	N/A	0.254
293343898	EID2	EP300 interacting inhibitor of differentiation 2	0.255
404351673	PPP1R21	protein phosphatase 1 regulatory subunit 21	0.255
821005859	N/A	N/A	0.255
594115840	N/A	N/A	0.255
293346096	FAM171B	family with sequence similarity 171 member B	0.256
74147193	HNRNPLL	heterogeneous nuclear ribonucleoprotein L like	0.256
884945546	N/A	N/A	0.256
9910378	CDC42SE2	CDC42 small effector 2	0.257
564384443	EIF4ENIF1	eukaryotic translation initiation factor 4E nuclear import factor 1	0.257
730529	RPL13	ribosomal protein L13	0.257
148709301	SPOCK1	SPARC/osteonectin, cwcv and kazal like domains proteoglycan 1	0.258
213511844	ALG2	ALG2, alpha-1,3/1,6-mannosyltransferase	0.259
149038734	EIF4EBP2	eukaryotic translation initiation factor 4E binding protein 2	0.260

62543521	FAM89B	family with sequence similarity 89 member B	0.260
55741780	SPG21	SPG21, maspardin	0.260
344249173	N/A	N/A	0.260
13242322	ATF4	activating transcription factor 4	0.261
157822067	BAP1	BRCA1 associated protein 1	0.261
142375024	CCDC92	coiled-coil domain containing 92	0.261
149049279	N/A	N/A	0.261
564399115	N/A	N/A	0.261
802996718	N/A	N/A	0.261
539937	ARL14	ADP ribosylation factor like GTPase 14	0.262
388596656	SLC4A1AP	solute carrier family 4 member 1 adaptor protein	0.262
71361619	BRIX1	BRX1, biogenesis of ribosomes	0.263
281371443	CASTOR2	cytosolic arginine sensor for mTORC1 subunit 2	0.263
12004970	RNF11	ring finger protein 11	0.263
149018882	N/A	N/A	0.263
148694630	N/A	N/A	0.263
926702009	N/A	N/A	0.263
149755772	N/A	N/A	0.263
114052915	CADM4	cell adhesion molecule 4	0.264
564390601	GPRIN1	G protein regulated inducer of neurite outgrowth 1	0.264
201066397	MPP6	membrane palmitoylated protein 6	0.264
124487247	PRICKLE2	prickle planar cell polarity protein 2	0.264
148672373	N/A	N/A	0.264
408772026	Afg3l1	AFG3-like AAA ATPase 1	0.265
157822675	FLRT2	fibronectin leucine rich transmembrane protein 2	0.265
189163475	LRFN1	leucine rich repeat and fibronectin type III domain containing 1	0.265
57527375	THUMPD1	THUMP domain containing 1	0.265
50511227	ZBTB34	zinc finger and BTB domain containing 34	0.265
281332151	ROBO2	roundabout guidance receptor 2	0.266
397787574	ST8SIA1	ST8 alpha-N-acetyl-neuraminide alpha-2,8-sialyltransferase 1	0.266
672067124	N/A	N/A	0.266
149018342	DYNC1LI1	dynein cytoplasmic 1 light intermediate chain 1	0.267
167555101	STRADB	STE20-related kinase adaptor beta	0.267
6981296	NUP50	nucleoporin 50	0.268

564368081	REV1	REV1, DNA directed polymerase	0.268
625263830	N/A	N/A	0.268
582015198	CRY2	cryptochrome circadian clock 2	0.269
61556927	EIF3G	eukaryotic translation initiation factor 3 subunit G	0.269
157824087	LRFN4	leucine rich repeat and fibronectin type III domain containing 4	0.269
149028217	SEMA6B	semaphorin 6B	0.269
148691289	TUBB	tubulin beta class I	0.269
568948348	AKT1S1	AKT1 substrate 1	0.270
67078454	SLC25A51	solute carrier family 25 member 51	0.270
148701223	N/A	N/A	0.270
157073939	LOC728392	uncharacterized LOC728392	0.271
309319787	OGFR	opioid growth factor receptor	0.271
209863068	SEMA7A	semaphorin 7A (John Milton Hagen blood group)	0.271
157820903	BCL7C	BCL tumor suppressor 7C	0.272
24586721	EEF1B2	eukaryotic translation elongation factor 1 beta 2	0.272
635137880	N/A	N/A	0.272
6978787	DYRK1A	dual specificity tyrosine phosphorylation regulated kinase 1A	0.273
197386405	FAM217B	family with sequence similarity 217 member B	0.273
21326451	HMGA1	high mobility group AT-hook 1	0.273
422398900	CREBZF	CREB/ATF bZIP transcription factor	0.274
60360266	PPP2R2A	protein phosphatase 2 regulatory subunit Balpha	0.274
164565387	TBC1D14	TBC1 domain family member 14	0.274
148705008	N/A	N/A	0.274
537193756	N/A	N/A	0.274
26337455	HDGFL3	HDGF like 3	0.276
199560289	REXO1	RNA exonuclease 1 homolog	0.276
157817961	PHF3	PHD finger protein 3	0.277
148695032	PSMD14	proteasome 26S subunit, non-ATPase 14	0.277
149047323	ZNF518B	zinc finger protein 518B	0.277
817271762	N/A	N/A	0.277
759100021	N/A	N/A	0.277
148687213	COX19	COX19, cytochrome c oxidase assembly factor	0.278
672071367	N/A	N/A	0.278
148710059	TRIM8	tripartite motif containing 8	0.279
829979052	N/A	N/A	0.279

71680975	Akap17a	A-kinase anchoring protein 17A	0.280
392333339	FAM124A	family with sequence similarity 124 member A	0.280
157819153	IRF2BP1	interferon regulatory factor 2 binding protein 1	0.280
154759279	MARCH9	membrane associated ring-CH-type finger 9	0.280
157819369	LRFN3	leucine rich repeat and fibronectin type III domain containing 3	0.281
817476911	PSMB4	proteasome subunit beta 4	0.281
149047261	QDPR	quinoid dihydropteridine reductase	0.281
16758158	UFD1	ubiquitin recognition factor in ER associated degradation 1	0.281
532003341	N/A	N/A	0.281
672062795	N/A	N/A	0.281
35068	NME1	NME/NM23 nucleoside diphosphate kinase 1	0.282
28174943	RPL24	ribosomal protein L24	0.282
189491614	SLC25A46	solute carrier family 25 member 46	0.282
655832893	N/A	N/A	0.282
109505188	FAM208B	family with sequence similarity 208 member B	0.283
6981010	Hba1/Hba2	hemoglobin, alpha 1	0.283
70794797	USP3	ubiquitin specific peptidase 3	0.283
564329567	Vbp1	VHL binding protein 1	0.283
14010889	RPS6KB1	ribosomal protein S6 kinase B1	0.284
672040089	N/A	N/A	0.284
188595725	C11orf57	chromosome 11 open reading frame 57	0.285
6753384	CDK5R1	cyclin dependent kinase 5 regulatory subunit 1	0.285
40018540	DDX24	DEAD-box helicase 24	0.285
149064951	DYNC1I1	dynein cytoplasmic 1 intermediate chain 1	0.285
46485444	NOP53	NOP53 ribosome biogenesis factor	0.285
61889068	MXI1	MAX interactor 1, dimerization protein	0.286
564311072	TNFAIP8L1	TNF alpha induced protein 8 like 1	0.286
488510613	N/A	N/A	0.286
149036010	COPE	coatomer protein complex subunit epsilon	0.287
157821079	ITPA	inosine triphosphatase	0.287
50510855	RIMKLB	ribosomal modification protein rimK like family member B	0.287
77797839	UBXN1	UBX domain protein 1	0.287
149037637	N/A	N/A	0.287
847038810	N/A	N/A	0.287
60499029	SET	SET nuclear proto-oncogene	0.288
25453384	SLC30A4	solute carrier family 30 member 4	0.288
537222089	N/A	N/A	0.288

119569672	BUB3	BUB3, mitotic checkpoint protein	0.289
47059500	NSG1	neuron specific gene family member 1	0.290
61557316	ST3GAL1	ST3 beta-galactoside alpha-2,3-sialyltransferase 1	0.290
672026875	N/A	N/A	0.290
918611848	N/A	N/A	0.291
931568103	N/A	N/A	0.292
584088128	N/A	N/A	0.292
60360548	DTX1	deltex E3 ubiquitin ligase 1	0.293
148747146	PPP2R2D	protein phosphatase 2 regulatory subunit Bdelta	0.293
293346766	TCAF1	TRPM8 channel associated factor 1	0.293
926688833	N/A	N/A	0.294
958720315	N/A	N/A	0.294
625204821	N/A	N/A	0.294
157823551	MAP1S	microtubule associated protein 1S	0.295
77695933	NELL2	neural EGFL like 2	0.295
114145515	Pkn3	protein kinase N3	0.295
310616720	DHX37	DEAH-box helicase 37	0.296
281427149	FAM69A	family with sequence similarity 69 member A	0.296
13929168	FAT1	FAT atypical cadherin 1	0.296
2920825	RPS2	ribosomal protein S2	0.296
148670791	ZFYVE1	zinc finger FYVE-type containing 1	0.296
157824174	HIGD2A	HIG1 hypoxia inducible domain family member 2A	0.297
157822151	ERH	enhancer of rudimentary homolog (Drosophila)	0.298
672026416	PRR36	proline rich 36	0.298
149067653	N/A	N/A	0.298
564340181	SETX	senataxin	0.299
157278004	SOBP	sine oculis binding protein homolog	0.299
23097350	TOMM20	translocase of outer mitochondrial membrane 20	0.299
672064354	FOXP4	forkhead box P4	0.300
448824835	Mff	mitochondrial fission factor	0.300
197382256	PHF12	PHD finger protein 12	0.300
16758316	PIP4K2B	phosphatidylinositol-5-phosphate 4-kinase type 2 beta	0.300
21914829	PDCD4	programmed cell death 4	0.302
148697614	MAF1	MAF1 homolog, negative regulator of RNA polymerase III	0.303
564367958	SEMA4C	semaphorin 4C	0.304

672082323	N/A	N/A	0.304
157822433	EID1	EP300 interacting inhibitor of differentiation 1	0.305
76362828	TEF	TEF, PAR bZIP transcription factor	0.305
47155567	ARHGAP20	Rho GTPase activating protein 20	0.307
148679797	DEF8	differentially expressed in FDCP 8 homolog	0.307
293347435	PTPRD	protein tyrosine phosphatase, receptor type D	0.307
987939967	N/A	N/A	0.307
672013322	LOC103689966	MARCKS-related protein-like	0.308
395627633	PCP4	Purkinje cell protein 4	0.308
395824794	N/A	N/A	0.308
564394038	CTDP1	CTD phosphatase subunit 1	0.309
156139120	NTS	neurotensin	0.309
148682320	PCMTD1	protein-L-isoaspartate (D-aspartate) O-methyltransferase domain containing 1	0.309
593735187	N/A	N/A	0.309
149057945	DUSP4	dual specificity phosphatase 4	0.310
34877176	GPRIN2	G protein regulated inducer of neurite outgrowth 2	0.310
564340133	GTF3C4	general transcription factor IIIC subunit 4	0.310
149067325	TDG	thymine DNA glycosylase	0.310
26350839	AP3S2	adaptor related protein complex 3 sigma 2 subunit	0.311
6978613	CCK	cholecystokinin	0.311
187282055	ZNF579	zinc finger protein 579	0.311
149037114	GNG10	G protein subunit gamma 10	0.312
55926145	NME2	NME/NM23 nucleoside diphosphate kinase 2	0.312
998662027	N/A	N/A	0.312
511848456	N/A	N/A	0.312
425384	CAMK4	calcium/calmodulin dependent protein kinase IV	0.313
199561799	LRP12	LDL receptor related protein 12	0.313
157823125	MRPS30	mitochondrial ribosomal protein S30	0.313
62078927	CDV3	CDV3 homolog	0.314
940782404	N/A	N/A	0.314
327358533	N/A	N/A	0.315
127138710	HNRNPD	heterogeneous nuclear ribonucleoprotein D	0.316
24233544	SBK1	SH3 domain binding kinase 1	0.316
187937036	ZC3HC1	zinc finger C3HC-type containing 1	0.316
300796515	DPY19L1	dpy-19 like 1	0.317
28212232	GNL3	G protein nucleolar 3	0.317

402692348	LRRC4B	leucine rich repeat containing 4B	0.317
56090552	GTF2F1	general transcription factor IIF subunit 1	0.318
27229055	HYPK	huntingtin interacting protein K	0.318
110624761	YIPF5	Yip1 domain family member 5	0.318
221040576	MPPED1	metallophosphoesterase domain containing 1	0.319
157819315	OSBPL11	oxysterol binding protein like 11	0.319
672043577	Rprd2	regulation of nuclear pre-mRNA domain containing 2	0.319
148700264	SH3RF3	SH3 domain containing ring finger 3	0.319
207318	TMSB10/TMSB4X	thymosin beta 4, X-linked	0.319
47058994	ATP5L	ATP synthase, H+ transporting, mitochondrial Fo complex subunit G	0.320
62244083	PDRG1	p53 and DNA damage regulated 1	0.321
564384353	SH3BP2	SH3 domain binding protein 2	0.321
60359940	SYT4	synaptotagmin 4	0.321
300798476	THAP12	THAP domain containing 12	0.321
157823667	UTP15	UTP15, small subunit processome component	0.321
755472674	N/A	N/A	0.321
157786600	RNF145	ring finger protein 145	0.322
61557082	TERF2IP	TERF2 interacting protein	0.323
189491869	KCMF1	potassium channel modulatory factor 1	0.325
149066670	TMEM5	transmembrane protein 5	0.325
148704849	N/A	N/A	0.325
431916930	N/A	N/A	0.325
564363346	BCL9L	B-cell CLL/lymphoma 9 like	0.326
564394925	PAPD5	poly(A) RNA polymerase D5, non-canonical	0.326
564377500	N/A	N/A	0.326
72004267	AKIRIN1	akirin 1	0.327
15079262	SPNS2	sphingolipid transporter 2	0.327
795445199	N/A	N/A	0.327
148697042	N/A	N/A	0.327
3513451	ALG10	ALG10, alpha-1,2-glucosyltransferase	0.328
564332092	CARS	cysteinyl-tRNA synthetase	0.328
672028116	TAPT1	transmembrane anterior posterior transformation 1	0.329
66730525	LRRC73	leucine rich repeat containing 73	0.330
149064983	N/A	N/A	0.330
639869	CHKA	choline kinase alpha	0.331
157787068	GRM3	glutamate metabotropic receptor 3	0.331

74195796	Ptma (includes others)	prothymosin alpha	0.331
564394180	SALL3	spalt like transcription factor 3	0.332
281604121	C11orf58	chromosome 11 open reading frame 58	0.333
140971918	Hnrnpab	heterogeneous nuclear ribonucleoprotein A/B	0.334
61556795	IGFBP7	insulin like growth factor binding protein 7	0.334
537238017	N/A	N/A	0.334
564315672	CLASP1	cytoplasmic linker associated protein 1	0.336
59709429	ZSCAN21	zinc finger and SCAN domain containing 21	0.337
672026667	CUX1	cut like homeobox 1	0.338
50510655	PCF11	PCF11 cleavage and polyadenylation factor subunit	0.338
568970985	MBTD1	mbt domain containing 1	0.339
210147441	ATXN7L3B	ataxin 7 like 3B	0.340
672069572	KANSL1	KAT8 regulatory NSL complex subunit 1	0.340
564298767	N/A	N/A	0.340
197252056	MED1	mediator complex subunit 1	0.341
157822663	RAB22A	RAB22A, member RAS oncogene family	0.341
157822643	RPF2	ribosome production factor 2 homolog	0.341
149063191	STX2	syntaxin 2	0.341
725566074	N/A	N/A	0.341
148675659	CSDE1	cold shock domain containing E1	0.342
81907791	SVOP	SV2 related protein	0.342
157819919	TRAF4	TNF receptor associated factor 4	0.342
2252816	AXIN1	axin 1	0.343
58865962	RNF41	ring finger protein 41	0.343
51491900	TOR1A	torsin family 1 member A	0.343
189491666	DDA1	DET1 and DDB1 associated 1	0.344
20806137	DDX46	DEAD-box helicase 46	0.344
821013078	N/A	N/A	0.344
568983220	PAPD7	poly(A) RNA polymerase D7, non-canonical	0.345
672028764	ENOX1	ecto-NOX disulfide-thiol exchanger 1	0.346
157818841	POGZ	pogo transposable element derived with ZNF domain	0.346
162417967	IER5	immediate early response 5	0.347
14277700	RPS12	ribosomal protein S12	0.348
13786132	Acot1	acyl-CoA thioesterase 1	0.349
68534262	C1orf43	chromosome 1 open reading frame 43	0.349
564325373	PRR18	proline rich 18	0.349
9506421	BACE1	beta-secretase 1	0.350

70794766	MRPS25	mitochondrial ribosomal protein S25	0.350
157819257	Ubxn7	UBX domain protein 7	0.350
119616373	MEF2C	myocyte enhancer factor 2C	0.351
169234953	SELENOH	selenoprotein H	0.351
24025618	DAB1	DAB1, reelin adaptor protein	0.352
149056919	SELENOW	selenoprotein W	0.352
291042683	DCAF5	DDB1 and CUL4 associated factor 5	0.353
57527466	DCUN1D5	defective in cullin neddylation 1 domain containing 5	0.353
62078455	PTRH2	peptidyl-tRNA hydrolase 2	0.353
574584811	TUBB4A	tubulin beta 4A class IVa	0.353
392343448	KLHL13	kelch like family member 13	0.354
148707696	Snrpe	small nuclear ribonucleoprotein E	0.354
149044118	WDR20	WD repeat domain 20	0.355
672085484	Rhou	ras homolog family member U	0.356
357588462	BCCIP	BRCA2 and CDKN1A interacting protein	0.358
149047360	N/A	N/A	0.358
160333089	MAZ	MYC associated zinc finger protein	0.359
8393823	Nefm	neurofilament, medium polypeptide	0.359
677444634	N/A	N/A	0.359
119603586	THAP11	THAP domain containing 11	0.360
85701722	RPRML	reprimo like	0.361
157823413	THOC3	THO complex 3	0.361
157787107	CCNG2	cyclin G2	0.362
109791	CLPS	colipase	0.363
157822835	IGFBPL1	insulin like growth factor binding protein like 1	0.363
300793780	ZNF251	zinc finger protein 251	0.363
537229315	N/A	N/A	0.364
148673911	Gm21596/Hmgb1	high mobility group box 1	0.366
157817121	TCTE1	t-complex-associated-testis-expressed 1	0.367
149031601	Hist1h1c	histone cluster 1 H1 family member c	0.368
156627555	NT5C3B	5'-nucleotidase, cytosolic IIIB	0.368
149064973	ASNS	asparagine synthetase (glutamine-hydrolyzing)	0.369
56606102	BAG5	BCL2 associated athanogene 5	0.369
157824132	TTC9B	tetratricopeptide repeat domain 9B	0.369
109490297	ABCA3	ATP binding cassette subfamily A member 3	0.370
564308639	N/A	N/A	0.370
405113035	E2F4	E2F transcription factor 4	0.371
157819221	NAA30	N(alpha)-acetyltransferase 30, NatC catalytic subunit	0.371

440907053	N/A	N/A	0.372
17985949	HBB	hemoglobin subunit beta	0.373
281427139	TADA2B	transcriptional adaptor 2B	0.373
672014573	N/A	N/A	0.373
149044005	N/A	N/A	0.373
564333920	PPRC1	peroxisome proliferator-activated receptor gamma, coactivator-related 1	0.374
158517832	SEC61G	Sec61 translocon gamma subunit	0.374
77917554	SLC39A9	solute carrier family 39 member 9	0.374
564343121	SMOX	spermine oxidase	0.374
564323057	ARMCX4	armadillo repeat containing, X-linked 4	0.375
37359818	KCTD5	potassium channel tetramerization domain containing 5	0.376
537237916	N/A	N/A	0.376
157823465	CHSY1	chondroitin sulfate synthase 1	0.377
71361655	MRPL12	mitochondrial ribosomal protein L12	0.377
40789237	PCDHA4	protocadherin alpha 4	0.377
55741514	TSEN34	tRNA splicing endonuclease subunit 34	0.377
672028599	SACS	sacsin molecular chaperone	0.378
564300780	TRIM33	tripartite motif containing 33	0.378
548456234	N/A	N/A	0.378
594042682	N/A	N/A	0.380
392306987	N/A	N/A	0.380
62945326	FAM220A	family with sequence similarity 220 member A	0.382
12847552	H3F3A/H3F3B	H3 histone family member 3A	0.383
312596922	MRPL52	mitochondrial ribosomal protein L52	0.383
57164019	B4GALT3	beta-1,4-galactosyltransferase 3	0.384
157818273	CDC42EP4	CDC42 effector protein 4	0.385
157821097	MRPL3	mitochondrial ribosomal protein L3	0.385
524964924	N/A	N/A	0.385
112180619	YPEL3	yippee like 3	0.386
564300505	SH3D19	SH3 domain containing 19	0.387
197246116	PINK1	PTEN induced putative kinase 1	0.388
149045006	N/A	N/A	0.388
340805866	ADRA2A	adrenoceptor alpha 2A	0.389
142976617	SEPHS2	selenophosphate synthetase 2	0.389
672036551	ZDHHC13	zinc finger DHHC-type containing 13	0.389
404312698	CASC4	cancer susceptibility 4	0.390
62078827	CTDSPL2	CTD small phosphatase like 2	0.390
60678292	Hba1/Hba2	hemoglobin, alpha 1	0.390

149023303	MRPS26	mitochondrial ribosomal protein S26	0.390
584277018	NR1D2	nuclear receptor subfamily 1 group D member 2	0.390
355557615	N/A	N/A	0.390
203278	CLTA	clathrin light chain A	0.391
157822431	FJX1	four jointed box 1	0.391
564299234	Lcor	ligand dependent nuclear receptor corepressor	0.392
634895400	N/A	N/A	0.392
48976087	TFB1M	transcription factor B1, mitochondrial	0.393
149043402	N/A	N/A	0.393
157819969	CTU1	cytosolic thiouridylase subunit 1	0.396
564317718	Ktn1	kinectin 1	0.396
6981302	ODC1	ornithine decarboxylase 1	0.396
58865996	TRIM13	tripartite motif containing 13	0.397
625246380	N/A	N/A	0.400
880805457	GPR161	G protein-coupled receptor 161	0.402
58865998	PCDHGB7	protocadherin gamma subfamily B, 7	0.403
157820771	ZNRF2	zinc and ring finger 2	0.403
148702008	N/A	N/A	0.403
59937915	ARIH2	ariadne RBR E3 ubiquitin protein ligase 2	0.404
141803183	ZKSCAN3	zinc finger with KRAB and SCAN domains 3	0.404
946774489	N/A	N/A	0.404
281371427	NRIP1	nuclear receptor interacting protein 1	0.405
71043628	OGFRL1	opioid growth factor receptor like 1	0.405
74220037	FAM107B	family with sequence similarity 107 member B	0.406
392351637	FAM171A2	family with sequence similarity 171 member A2	0.406
157821997	MED28	mediator complex subunit 28	0.406
201066403	TGIF2	TGFB induced factor homeobox 2	0.406
112984540	FCF1	FCF1 rRNA-processing protein	0.407
795155982	N/A	N/A	0.407
148676796	N/A	N/A	0.407
564363988	ISLR2	immunoglobulin superfamily containing leucine rich repeat 2	0.408
82617653	PCDHGA12	protocadherin gamma subfamily A, 12	0.409
820987686	N/A	N/A	0.409
564357258	CCDC85C	coiled-coil domain containing 85C	0.410
198278471	ZBED4	zinc finger BED-type containing 4	0.410
724928464	N/A	N/A	0.410
17933496	TMEM158	transmembrane protein 158 (gene/pseudogene)	0.411
672050972	N/A	N/A	0.411
672084101	N/A	N/A	0.412

157822027	CSRNP2	cysteine and serine rich nuclear protein 2	0.413
149058345	N/A	N/A	0.414
19424250	CST6	cystatin E/M	0.415
148672336	N/A	N/A	0.415
149047844	LYPD6	LY6/PLAUR domain containing 6	0.416
8923415	MARCH5	membrane associated ring-CH-type finger 5	0.416
157817720	SLC16A14	solute carrier family 16 member 14	0.416
300797828	KAT14	lysine acetyltransferase 14	0.417
585689575	N/A	N/A	0.417
564378247	CCZ1/CCZ1B	CCZ1 homolog B, vacuolar protein trafficking and biogenesis associated	0.418
704532863	N/A	N/A	0.418
56090485	M6PR	mannose-6-phosphate receptor, cation dependent	0.419
564395313	OTUD4	OTU deubiquitinase 4	0.419
109480728	TMEM74	transmembrane protein 74	0.419
109480728	TMEM74	transmembrane protein 74	0.419
161621245	ABT1	activator of basal transcription 1	0.420
157820119	LRRTM1	leucine rich repeat transmembrane neuronal 1	0.420
537238000	N/A	N/A	0.421
157821267	RFC5	replication factor C subunit 5	0.422
537211362	N/A	N/A	0.422
564357463	KIF26A	kinesin family member 26A	0.423
50511183	TMEM200A	transmembrane protein 200A	0.423
359324332	N/A	N/A	0.423
148700102	SIRT1	sirtuin 1	0.424
189011649	NUDT16	nudix hydrolase 16	0.425
33086594	SAT1	spermidine/spermine N1-acetyltransferase 1	0.425
226693321	FKBP11	FK506 binding protein 11	0.426
672059756	N/A	N/A	0.426
157819927	SNRPB2	small nuclear ribonucleoprotein polypeptide B2	0.427
8394196	NTM	neurotrimin	0.428
148673748	FAM110B	family with sequence similarity 110 member B	0.429
157819717	NIPA2	non imprinted in Prader-Willi/Angelman syndrome 2	0.429
76096328	COMMD9	COMM domain containing 9	0.430
635102874	N/A	N/A	0.430
61557236	RAD23A	RAD23 homolog A, nucleotide excision repair protein	0.431
148709746	N/A	N/A	0.432

77917610	GPBP1L1	GC-rich promoter binding protein 1 like 1	0.434
18426862	PSMD9	proteasome 26S subunit, non-ATPase 9	0.435
157822367	PUS3	pseudouridylate synthase 3	0.435
109467956	PPP1R26	protein phosphatase 1 regulatory subunit 26	0.436
564379881	PGAM5	PGAM family member 5, mitochondrial serine/threonine protein phosphatase	0.437
223555981	UBE2QL1	ubiquitin conjugating enzyme E2 Q family like 1	0.437
21450249	C9orf78	chromosome 9 open reading frame 78	0.438
6680007	GJC1	gap junction protein gamma 1	0.438
157823823	CCNJ	cyclin J	0.439
109475418	FOXO6	forkhead box O6	0.441
149058952	MEF2C	myocyte enhancer factor 2C	0.441
672028080	PRR14L	proline rich 14 like	0.441
157823369	TERF2	telomeric repeat binding factor 2	0.442
148687364	Cux1	cut-like homeobox 1	0.443
58865880	TBRG4	transforming growth factor beta regulator 4	0.443
564311452	TMEM131	transmembrane protein 131	0.444
635108195	N/A	N/A	0.444
672073189	N/A	N/A	0.444
672017116	N/A	N/A	0.445
564345076	N/A	N/A	0.445
157819301	ZNF777	zinc finger protein 777	0.446
26351105	MDK	midkine (neurite growth-promoting factor 2)	0.448
157821747	MDM2	MDM2 proto-oncogene	0.448
149016584	ZNF606	zinc finger protein 606	0.448
672026129	LRCH3	leucine rich repeats and calponin homology domain containing 3	0.449
157817845	KMT5B	lysine methyltransferase 5B	0.450
40789241	PCDHA3	protocadherin alpha 3	0.450
564384243	Gm1673	predicted gene 1673	0.451
392350322	DNAJC13	DnaJ heat shock protein family (Hsp40) member C13	0.452
143359181	PQLC1	PQ loop repeat containing 1	0.452
402794103	ATG101	autophagy related 101	0.453
11559958	SYT6	synaptotagmin 6	0.453
564338846	INTS12	integrator complex subunit 12	0.454
31543340	NR4A2	nuclear receptor subfamily 4 group A member 2	0.456
672053062	FKBP15	FK506 binding protein 15	0.458
349501022	2410002F23Rik	RIKEN cDNA 2410002F23 gene	0.460

817273101	N/A	N/A	0.460
62647770	H1FX	H1 histone family member X	0.461
672044124	N/A	N/A	0.462
158186787	TK1	thymidine kinase 1	0.463
149031942	N/A	N/A	0.465
568941586	IQSEC1	IQ motif and Sec7 domain 1	0.466
564316286	CNST	consortin, connexin sorting protein	0.467
3212116	PFDN2	prefoldin subunit 2	0.467
112421036	POU3F3	POU class 3 homeobox 3	0.467
564351113	N/A	N/A	0.467
51095128	LINC00998	long intergenic non-protein coding RNA 998	0.468
675706491	N/A	N/A	0.468
564365330	CDC25A	cell division cycle 25A	0.469
148681067	VASH2	vasohibin 2	0.469
189011636	ATRAID	all-trans retinoic acid induced differentiation factor	0.471
84000579	FTL	ferritin light chain	0.472
564320789	SEH1L	SEH1 like nucleoporin	0.472
149052692	N/A	N/A	0.472
164565364	ITPKB	inositol-trisphosphate 3-kinase B	0.473
300795203	HS6ST2	heparan sulfate 6-O-sulfotransferase 2	0.474
148679437	HAS3	hyaluronan synthase 3	0.475
157822691	NTSR1	neurotensin receptor 1	0.475
30024612	SOX11	SRY-box 11	0.476
440909886	N/A	N/A	0.476
30017415	ITPKC	inositol-trisphosphate 3-kinase C	0.480
803269187	N/A	N/A	0.482
884926991	N/A	N/A	0.482
564362348	Fam76b	family with sequence similarity 76, member B	0.484
564335688	TRIO	trio Rho guanine nucleotide exchange factor	0.485
18875392	RFXAP	regulatory factor X associated protein	0.486
528768520	N/A	N/A	0.486
148673911	Gm21596/Hmgb1	high mobility group box 1	0.487
157822475	NGDN	neuroguidin	0.488
109478211	TOGARAM1	TOG array regulator of axonemal microtubules 1	0.489
725568549	N/A	N/A	0.490
56090445	PHOSPHO2	phosphatase, orphan 2	0.491
254028164	NGRN	neugrin, neurite outgrowth associated	0.494
157824124	NUAK1	NUAK family kinase 1	0.494

151554393	SIAH1	siah E3 ubiquitin protein ligase 1	0.497
431911718	N/A	N/A	0.497
300794118	MN1	MN1 proto-oncogene, transcriptional regulator	0.498
403310686	SOX4	SRY-box 4	0.500
965920370	N/A	N/A	0.503
795170747	N/A	N/A	0.503
73990974	LZTS3	leucine zipper tumor suppressor family member 3	0.504
56605644	TFB2M	transcription factor B2, mitochondrial	0.509
149066158	ZNF623	zinc finger protein 623	0.509
880913460	N/A	N/A	0.509
13928942	PER2	period circadian clock 2	0.513
392354145	HNRNPA0	heterogeneous nuclear ribonucleoprotein A0	0.515
157786766	RFC4	replication factor C subunit 4	0.515
544442375	N/A	N/A	0.516
403043582	SOCS6	suppressor of cytokine signaling 6	0.517
34328151	TBR1	T-box, brain 1	0.518
29789082	COIL	coilin	0.519
51948492	NUDT19	nudix hydrolase 19	0.519
62078923	DZIP1L	DAZ interacting zinc finger protein 1 like	0.521
672080026	N/A	N/A	0.522
62078729	CDKN2AIP	CDKN2A interacting protein	0.523
17530969	SLC8A3	solute carrier family 8 member A3	0.523
157819731	PRR7	proline rich 7, synaptic	0.526
564315812	NAV1	neuron navigator 1	0.527
672047353	RALGAPA2	Ral GTPase activating protein catalytic alpha subunit 2	0.527
392331954	KANSL1	KAT8 regulatory NSL complex subunit 1	0.529
149064803	NHLRC3	NHL repeat containing 3	0.530
74143776	INIP	INTS3 and NABP interacting protein	0.531
25453400	CDH6	cadherin 6	0.532
238859603	ISLR2	immunoglobulin superfamily containing leucine rich repeat 2	0.532
672080115	TENM3	teneurin transmembrane protein 3	0.533
213385295	Ppp2r3d	protein phosphatase 2 (formerly 2A), regulatory subunit B", delta	0.534
564314663	VPS8	VPS8, CORVET complex subunit	0.535
238859603	ISLR2	immunoglobulin superfamily containing leucine rich repeat 2	0.537
404501518	ZNF569	zinc finger protein 569	0.537

537166446	N/A	N/A	0.539
157820017	FBXO33	F-box protein 33	0.542
197381846	PP2D1	protein phosphatase 2C like domain containing 1	0.542
676284727	N/A	N/A	0.543
655889411	N/A	N/A	0.543
56090289	PELO	pelota homolog (Drosophila)	0.545
564389552	LOC100910854	zinc finger MYND domain-containing protein 19-like	0.547
3800869	PIM3	Pim-3 proto-oncogene, serine/threonine kinase	0.548
537191098	N/A	N/A	0.548
157818339	ZBTB33	zinc finger and BTB domain containing 33	0.549
564333892	WNT8B	Wnt family member 8B	0.550
982264581	N/A	N/A	0.552
672085486	EGLN1	egl-9 family hypoxia inducible factor 1	0.556
149029231	LOC100365289	rCG57257-like	0.556
148706598	PKDCC	protein kinase domain containing, cytoplasmic	0.558
149064065	ZMYM5	zinc finger MYM-type containing 5	0.558
67078426	SPIN1	spindlin 1	0.560
293348129	DACT1	dishevelled binding antagonist of beta catenin 1	0.561
158186659	CBS/CBSL	cystathionine-beta-synthase	0.562
564307173	HEATR5A	HEAT repeat containing 5A	0.565
564318054	R3hcc1	R3H domain and coiled-coil containing 1	0.565
51980294	COQ3	coenzyme Q3, methyltransferase	0.566
955485868	N/A	N/A	0.566
148670774	N/A	N/A	0.566
201066401	RCOR3	REST corepressor 3	0.567
28972099	RUBCN	RUN and cysteine rich domain containing beclin 1 interacting protein	0.567
149026477	N/A	N/A	0.571
83642816	APOPT1	apoptogenic 1, mitochondrial	0.573
293351303	METTL22	methyltransferase like 22	0.573
21703842	RTCB	RNA 2',3'-cyclic phosphate and 5'-OH ligase	0.574
30017421	SLA	Src like adaptor	0.575
564303955	EMX1	empty spiracles homeobox 1	0.577
803119291	N/A	N/A	0.582
148704240	ZMYM2	zinc finger MYM-type containing 2	0.584
564317927	SACS	sacsin molecular chaperone	0.585
672072796	N/A	N/A	0.585
18376839	DDIT4	DNA damage inducible transcript 4	0.588

3676248	Prim1	primase (DNA) subunit 1	0.589
564316243	CEP170	centrosomal protein 170	0.590
149046410	ARHGEF4	Rho guanine nucleotide exchange factor 4	0.591
281604129	HELQ	helicase, POLQ-like	0.591
672057962	N/A	N/A	0.591
564310188	IGDCC4	immunoglobulin superfamily DCC subclass member 4	0.593
672053428	LRP8	LDL receptor related protein 8	0.593
884914541	N/A	N/A	0.593
7949105	PBX3	PBX homeobox 3	0.594
564382848	Hnrnpdl	heterogeneous nuclear ribonucleoprotein D-like	0.595
226371633	CABLES1	Cdk5 and Abl enzyme substrate 1	0.596
2341056	SOX12	SRY-box 12	0.597
157822893	IMP3	IMP3, U3 small nucleolar ribonucleoprotein	0.598
392349170	ZFP36L2	ZFP36 ring finger protein like 2	0.599
123405	POU3F1	POU class 3 homeobox 1	0.604
392345518	SENP5	SUMO1/sentrin specific peptidase 5	0.608
157818733	ZBTB2	zinc finger and BTB domain containing 2	0.609
406362836	HS6ST3	heparan sulfate 6-O-sulfotransferase 3	0.610
399154114	KPNA2	karyopherin subunit alpha 2	0.612
109467956	PPP1R26	protein phosphatase 1 regulatory subunit 26	0.613
672046728	N/A	N/A	0.613
444741673	CYHR1	cysteine and histidine rich 1	0.614
564378170	PAN3	PAN3 poly(A) specific ribonuclease subunit	0.614
672026767	N/A	N/A	0.615
395504625	N/A	N/A	0.615
149059246	N/A	N/A	0.616
219879771	PGAP3	post-GPI attachment to proteins 3	0.619
524940250	N/A	N/A	0.619
37360398	ISLR2	immunoglobulin superfamily containing leucine rich repeat 2	0.623
564397086	BRPF3	bromodomain and PHD finger containing 3	0.629
672078059	N/A	N/A	0.630
157819193	DOK5	docking protein 5	0.631
157823891	ING2	inhibitor of growth family member 2	0.633
694978694	N/A	N/A	0.633
16758574	CNTN5	contactin 5	0.636
564396111	ZCCHC14	zinc finger CCHC-type containing 14	0.636
149018731	TMEM108	transmembrane protein 108	0.643
564393060	N/A	N/A	0.644

20302045	JUND	JunD proto-oncogene, AP-1 transcription factor subunit	0.646
157820217	Gsta4	glutathione S-transferase, alpha 4	0.647
149035005	IQCE	IQ motif containing E	0.654
77627740	ING3	inhibitor of growth family member 3	0.655
11560065	GPR85	G protein-coupled receptor 85	0.656
399154114	KPNA2	karyopherin subunit alpha 2	0.656
672018099	N/A	N/A	0.657
124486586	AUTS2	AUTS2, activator of transcription and developmental regulator	0.660
524983306	N/A	N/A	0.660
293348214	CCDC88C	coiled-coil domain containing 88C	0.665
148704682	N/A	N/A	0.675
674054416	N/A	N/A	0.683
300796169	SP8	Sp8 transcription factor	0.684
564313508	BPTF	bromodomain PHD finger transcription factor	0.685
567316240	CEBPA	CCAAT/enhancer binding protein alpha	0.686
672022427	ZNF740	zinc finger protein 740	0.687
625253043	N/A	N/A	0.690
884945546	N/A	N/A	0.697
66730382	TRNT1	tRNA nucleotidyl transferase 1	0.698
568920164	TAF4	TATA-box binding protein associated factor 4	0.703
189011634	ARMC7	armadillo repeat containing 7	0.705
149024753	DFFB	DNA fragmentation factor subunit beta	0.705
302595867	Trim11	tripartite motif-containing 11	0.707
564352668	MYCL	MYCL proto-oncogene, bHLH transcription factor	0.713
149042171	RTL5	retrotransposon Gag like 5	0.713
149036607	N/A	N/A	0.713
157822359	PELI2	pellino E3 ubiquitin protein ligase family member 2	0.717
564377502	EIF4G1	eukaryotic translation initiation factor 4 gamma 1	0.722
39104628	SORBS1	sorbin and SH3 domain containing 1	0.725
555290059	MED7	mediator complex subunit 7	0.728
149052692	N/A	N/A	0.728
197386066	ZNF784	zinc finger protein 784	0.729
149045812	EXOSC3	exosome component 3	0.730
672029117	N/A	N/A	0.732

149034092	Grid1	glutamate ionotropic receptor delta type subunit 1	0.740
672019522	N/A	N/A	0.740
300797222	ANKRD13C	ankyrin repeat domain 13C	0.741
672088045	N/A	N/A	0.745
820980553	N/A	N/A	0.747
564347830	ZXDC	ZXD family zinc finger C	0.748
697023347	N/A	N/A	0.749
672060362	ELFN2	extracellular leucine rich repeat and fibronectin type III domain containing 2	0.751
348041347	CENPL	centromere protein L	0.752
148692940	WAPL	WAPL cohesin release factor	0.752
918577634	N/A	N/A	0.752
149056503	Zfp60	zinc finger protein 60	0.757
80861398	CRY1	cryptochrome circadian clock 1	0.761
148702781	N/A	N/A	0.764
564313637	CDR2L	cerebellar degeneration related protein 2 like	0.769
755566692	HUWE1	HECT, UBA and WWE domain containing 1, E3 ubiquitin protein ligase	0.776
149044808	PUS10	pseudouridylate synthase 10	0.777
149034959	ZNF12	zinc finger protein 12	0.777
431916930	N/A	N/A	0.777
672031975	LOC299312	similar to G protein-binding protein CRFG	0.779
564303928	TET3	tet methylcytosine dioxygenase 3	0.780
149048609	N/A	N/A	0.780
12831217	GPR27	G protein-coupled receptor 27	0.781
564329918	EMSY	EMSY, BRCA2 interacting transcriptional repressor	0.785
149047683	N/A	N/A	0.790
149044121	N/A	N/A	0.791
672025117	MBTD1	mbt domain containing 1	0.796
586908220	ARHGAP44	Rho GTPase activating protein 44	0.797
672019438	FKBP15	FK506 binding protein 15	0.798
958720315	N/A	N/A	0.799
672022661	N/A	N/A	0.805
672086880	N/A	N/A	0.811
564372912	GPS2	G protein pathway suppressor 2	0.816
392337738	LYSMD4	LysM domain containing 4	0.819
568907669	NYAP2	neuronal tyrosine-phosphorylated phosphoinositide-3-kinase adaptor 2	0.820

392354293	Hmgb3	high mobility group box 3	0.821
564321169	CHD9	chromodomain helicase DNA binding protein 9	0.822
20376822	PROKR1	prokineticin receptor 1	0.825
568926543	KIAA0368	KIAA0368	0.830
149016587	N/A	N/A	0.832
148682476	PTP4A1	protein tyrosine phosphatase type IVA, member 1	0.836
564306696	SRBD1	S1 RNA binding domain 1	0.837
731286412	N/A	N/A	0.840
564322442	Kdm6a	lysine demethylase 6A	0.842
109460021	KIAA2026	KIAA2026	0.846
392346263	TTLL11	tubulin tyrosine ligase like 11	0.849
564315667	CLASP1	cytoplasmic linker associated protein 1	0.851
149018731	TMEM108	transmembrane protein 108	0.852
564352950	N/A	N/A	0.857
149025186	RPS6KL1	ribosomal protein S6 kinase like 1	0.858
293342200	DGKH	diacylglycerol kinase eta	0.860
392339263	PKP4	plakophilin 4	0.861
672026785	N/A	N/A	0.862
672026702	N/A	N/A	0.865
392339806	CFAP69	cilia and flagella associated protein 69	0.866
112984092	RPRM	reprimo, TP53 dependent G2 arrest mediator homolog	0.867
197386527	LYPD6B	LY6/PLAUR domain containing 6B	0.876
537271325	N/A	N/A	0.880
293339965	RAB11FIP3	RAB11 family interacting protein 3	0.890
675649807	N/A	N/A	0.890
685536628	N/A	N/A	0.893
149058726	N/A	N/A	0.895
672087260	N/A	N/A	0.896
564312944	KIAA0753	KIAA0753	0.903
568992323	CSMD3	CUB and Sushi multiple domains 3	0.912
568929584	TRIM62	tripartite motif containing 62	0.916
149024681	N/A	N/A	0.920
568979800	NPAS3	neuronal PAS domain protein 3	0.926
293353154	TBC1D1	TBC1 domain family member 1	0.928
564298336	RBBP6	RB binding protein 6, ubiquitin ligase	0.934
68063179	N/A	N/A	0.946
741943159	OTX1	orthodenticle homeobox 1	0.947
109472884	UBE3C	ubiquitin protein ligase E3C	0.950

410515422	NTN1	netrin 1	0.956
6679423	POU3F2	POU class 3 homeobox 2	0.956
157818769	NSL1	NSL1, MIS12 kinetochore complex component	0.958
149025130	Fam161b	family with sequence similarity 161, member B	0.969
564310195	ANKDD1A	ankyrin repeat and death domain containing 1A	0.979
564297387	Zfp658	zinc finger protein 658	0.982
564329920	EMSY	EMSY, BRCA2 interacting transcriptional repressor	0.991
564316247	CEP170	centrosomal protein 170	1.000
26329649	IER5L	immediate early response 5 like	1.005
149025439	DICER1	dicer 1, ribonuclease III	1.008
148705473	FAM53A	family with sequence similarity 53 member A	1.008
148705043	RRM2	ribonucleotide reductase regulatory subunit M2	1.012
672086719	FAM184A	family with sequence similarity 184 member A	1.013
293346251	TMEM62	transmembrane protein 62	1.013
564309098	EFCAB6	EF-hand calcium binding domain 6	1.014
148701660	FSTL4	follistatin like 4	1.015
564307783	TECPR2	tectonin beta-propeller repeat containing 2	1.019
564341299	N/A	N/A	1.020
564307241	MIA2	melanoma inhibitory activity 2	1.021
6978894	GIPR	gastric inhibitory polypeptide receptor	1.026
672029702	ZCCHC6	zinc finger CCHC-type containing 6	1.027
672082610	N/A	N/A	1.030
564298041	GDPD5	glycerophosphodiester phosphodiesterase domain containing 5	1.031
672012920	N/A	N/A	1.034
752423229	N/A	N/A	1.034
109480102	SMARCC2	SWI/SNF related, matrix associated, actin dependent regulator of chromatin subfamily c member 2	1.042
672017191	N/A	N/A	1.048
157821155	RNF151	ring finger protein 151	1.052
149020413	Zfp599	zinc finger protein 599	1.054
564377118	WDR53	WD repeat domain 53	1.058
149053436	FAM57A	family with sequence similarity 57 member A	1.075
568983685	TMEM161B	transmembrane protein 161B	1.075
149045719	PIGO	phosphatidylinositol glycan anchor biosynthesis class O	1.076
564296988	ZNF235	zinc finger protein 235	1.078
564327667	TSHZ3	teashirt zinc finger homeobox 3	1.080

675706185	N/A	N/A	1.082
61556894	Akr1b10	aldo-keto reductase family 1, member B10 (aldose reductase)	1.085
444741673	CYHR1	cysteine and histidine rich 1	1.111
149016574	ZNF324	zinc finger protein 324	1.113
149050030	MTRF1	mitochondrial translation release factor 1	1.114
672024381	N/A	N/A	1.134
80751167	PCDHGA5	protocadherin gamma subfamily A, 5	1.135
149051391	N/A	N/A	1.135
293356488	RIC1	RIC1 homolog, RAB6A GEF complex partner 1	1.136
961766127	N/A	N/A	1.147
564323075	ZMAT1	zinc finger matrin-type 1	1.163
672023090	N/A	N/A	1.166
19424314	KCNE2	potassium voltage-gated channel subfamily E regulatory subunit 2	1.167
75832143	FLVCR2	feline leukemia virus subgroup C cellular receptor family member 2	1.170
149048115	KIAA0907	KIAA0907	1.173
672015275	N/A	N/A	1.183
564341299	N/A	N/A	1.185
589930143	N/A	N/A	1.188
57526957	ACY3	aminoacylase 3	1.189
62644808	ADAMTSL2	ADAMTS like 2	1.195
672063675	N/A	N/A	1.208
625210478	N/A	N/A	1.219
664771727	N/A	N/A	1.224
148689488	SYN3	synapsin III	1.225
392338392	PCNT	pericentrin	1.238
124378035	TNRC6C	trinucleotide repeat containing 6C	1.239
672038660	ZNF48	zinc finger protein 48	1.244
14277700	RPS12	ribosomal protein S12	1.245
564297338	ZNF816	zinc finger protein 816	1.248
564312671	N/A	N/A	1.268
293342693	SLC35B3	solute carrier family 35 member B3	1.271
256220048	PCDHGC5	protocadherin gamma subfamily C, 5	1.272
564323057	ARMCX4	armadillo repeat containing, X-linked 4	1.275
755566692	HUWE1	HECT, UBA and WWE domain containing 1, E3 ubiquitin protein ligase	1.288
672070295	BAHCC1	BAH domain and coiled-coil containing 1	1.298

564302599	INSM1	INSM transcriptional repressor 1	1.310
672031995	Kdm6a	lysine demethylase 6A	1.326
112984482	SBSN	suprabasin	1.334
293358436	FOXP2	forkhead box P2	1.342
148705008	N/A	N/A	1.350
564299767	IPO11	importin 11	1.362
157818947	FAAP24	Fanconi anemia core complex associated protein 24	1.365
564318679	CCSER2	coiled-coil serine rich protein 2	1.370
669303362	N/A	N/A	1.388
392340509	PTPRD	protein tyrosine phosphatase, receptor type D	1.396
149020413	Zfp599	zinc finger protein 599	1.399
109474690	KLHL32	kelch like family member 32	1.401
564307839	JAG2	jagged 2	1.408
564315753	N/A	N/A	1.409
148675156	KLHL25	kelch like family member 25	1.414
564320493	KDM3B	lysine demethylase 3B	1.466
672043253	DENND4B	DENN domain containing 4B	1.477
564302924	TSHZ2	teashirt zinc finger homeobox 2	1.481
564311918	C2orf72	chromosome 2 open reading frame 72	1.485
11560067	GPR173	G protein-coupled receptor 173	1.505
392339412	PLA2G4E	phospholipase A2 group IVE	1.508
564313510	BPTF	bromodomain PHD finger transcription factor	1.529
149067796	TMEM219	transmembrane protein 219	1.539
564300485	LOC102551095	uncharacterized LOC102551095	1.546
8393418	GAPDH	glyceraldehyde-3-phosphate dehydrogenase	1.548
564311695	PIKFYVE	phosphoinositide kinase, FYVE-type zinc finger containing	1.564
564297736	N/A	N/A	1.565
293358899	ANKRD26	ankyrin repeat domain 26	1.569
672072352	Evi5l	ecotropic viral integration site 5 like	1.576
16758572	DLK1	delta like non-canonical Notch ligand 1	1.578
635017744	N/A	N/A	1.579
564313842	SLC38A10	solute carrier family 38 member 10	1.585
672012705	SYNE1	spectrin repeat containing nuclear envelope protein 1	1.617
672059136	N/A	N/A	1.619
672051147	N/A	N/A	1.649
109484871	HERC1	HECT and RLD domain containing E3 ubiquitin protein ligase family member 1	1.659

209571573	ZNF707	zinc finger protein 707	1.680
672088752	MCF2	MCF.2 cell line derived transforming sequence	1.684
564317923	SACS	sacsin molecular chaperone	1.684
966979996	N/A	N/A	1.684
672087657	N/A	N/A	1.702
164448680	HBB	hemoglobin subunit beta	1.704
829992801	N/A	N/A	1.704
918591579	N/A	N/A	1.707
817273427	N/A	N/A	1.709
568959785	PRDM10	PR/SET domain 10	1.720
564312627	ZFP62	ZFP62 zinc finger protein	1.722
564310553	TOPBP1	topoisomerase (DNA) II binding protein 1	1.732
568990288	NIPBL	NIPBL, cohesin loading factor	1.747
672013014	N/A	N/A	1.770
149041357	RGD1311744	similar to RIKEN cDNA 5830475I06	1.773
293362695	Akap17b	A kinase (PRKA) anchor protein 17B	1.784
568973498	TNRC6C	trinucleotide repeat containing 6C	1.791
149020413	Zfp599	zinc finger protein 599	1.829
149029159	N/A	N/A	1.841
725571770	N/A	N/A	1.848
149042879	N/A	N/A	1.869
755515866	BRAF	B-Raf proto-oncogene, serine/threonine kinase	1.876
564326713	ZC3H4	zinc finger CCCH-type containing 4	1.881
672057084	N/A	N/A	1.906
829923130	N/A	N/A	1.915
569001477	MTCL1	microtubule crosslinking factor 1	1.931
672089580	LOC103694865	TATA-binding protein-associated factor 2N-like	1.932
148681013	SYT14	synaptotagmin 14	1.942
569012000	KLF8	Kruppel like factor 8	1.954
564395350	N/A	N/A	1.964
2231145	N/A	N/A	1.978
564310412	DOPEY1	dopey family member 1	1.987
672023431	ZCWPW2	zinc finger CW-type and PWWP domain containing 2	1.991
672016550	RAPGEF1	Rap guanine nucleotide exchange factor 1	1.996
148708817	CA5B	carbonic anhydrase 5B	2.000
16758266	NME3	NME/NM23 nucleoside diphosphate kinase 3	2.007
672027860	APBB2	amyloid beta precursor protein binding family B member 2	2.023

564313512	N/A	N/A	2.085
672035395	DMWD	dystrophia myotonica, WD repeat containing	2.101
149030324	CHRNA2	cholinergic receptor nicotinic alpha 2 subunit	2.112
528758424	N/A	N/A	2.152
157822691	NTSR1	neurotensin receptor 1	2.178
149042883	LOC100365365	rCG32328-like	2.183
752385306	N/A	N/A	2.204
392334596	RSPH3	radial spoke 3 homolog	2.222
148696062	LRRC57	leucine rich repeat containing 57	2.228
672020363	N/A	N/A	2.244
148702471	N/A	N/A	2.248
564339225	N/A	N/A	2.250
1478205	PPP1R14B	protein phosphatase 1 regulatory inhibitor subunit 14B	2.266
672029704	ZCCHC6	zinc finger CCHC-type containing 6	2.273
568972622	BPTF	bromodomain PHD finger transcription factor	2.292
564324736	L3MBTL3	l(3)mbt-like 3 (Drosophila)	2.296
564310904	N/A	N/A	2.299
24308466	ITGB3	integrin subunit beta 3	2.303
913513476	N/A	N/A	2.322
149028845	CCNB2	cyclin B2	2.368
149043399	TAPBP	TAP binding protein	2.406
293348472	ZFR2	zinc finger RNA binding protein 2	2.409
672088045	N/A	N/A	2.423
672069572	KANSL1	KAT8 regulatory NSL complex subunit 1	2.457
672056365	KIAA0586	KIAA0586	2.459
672020328	MTA3	metastasis associated 1 family member 3	2.481
594100882	N/A	N/A	2.509
672013187	DMWD	dystrophia myotonica, WD repeat containing	2.555
148664561	DTNA	dystrobrevin alpha	2.684
672061705	KMT2A	lysine methyltransferase 2A	2.760
564313514	BPTF	bromodomain PHD finger transcription factor	2.807
293352633	FBRSL1	fibrosin like 1	2.880
564331258	ZNF688	zinc finger protein 688	2.910
672025361	BPTF	bromodomain PHD finger transcription factor	3.000
28972866	CSMD3	CUB and Sushi multiple domains 3	3.000
672026534	N/A	N/A	3.018
672044124	N/A	N/A	3.049
672088045	N/A	N/A	3.080
478536438	N/A	N/A	3.096

109488483	KIAA0753	KIAA0753	3.216
672053979	KCNQ4	potassium voltage-gated channel subfamily Q member 4	3.240
564313508	BPTF	bromodomain PHD finger transcription factor	3.248
148690434	MRPS34	mitochondrial ribosomal protein S34	3.263
564299019	KIAA2026	KIAA2026	3.322
124378035	TNRC6C	trinucleotide repeat containing 6C	3.341
470605072	N/A	N/A	3.341
568964954	EPB41L2	erythrocyte membrane protein band 4.1 like 2	3.402
333033763	TTR	transthyretin	3.420
4506663	RPL8	ribosomal protein L8	3.565
564311685	PIKFYVE	phosphoinositide kinase, FYVE-type zinc finger containing	3.585
157823859	METTL27	methyltransferase like 27	3.622
147907212	ASAP2	ArfGAP with SH3 domain, ankyrin repeat and PH domain 2	3.782
672068318	PITPNM3	PITPNM family member 3	3.841
564324736	L3MBTL3	l(3)mbt-like 3 (Drosophila)	3.926
672040275	CPEB3	cytoplasmic polyadenylation element binding protein 3	4.000
564317997	N/A	N/A	4.080
672060766	N/A	N/A	4.358
149031942	N/A	N/A	4.409
164448680	HBB	hemoglobin subunit beta	4.465
564329920	EMSY	EMSY, BRCA2 interacting transcriptional repressor	4.585
564297371	LOC102556967	zinc finger protein 484-like	4.585
564395696	TANGO6	transport and golgi organization 6 homolog	4.585
672088045	N/A	N/A	4.636
149018624	DOCK3	dedicator of cytokinesis 3	4.644
148679862	SLC35F3	solute carrier family 35 member F3	4.858
672087664	N/A	N/A	4.858
880855761	N/A	N/A	4.907
521036477	N/A	N/A	4.907
148223355	ASAP2	ArfGAP with SH3 domain, ankyrin repeat and PH domain 2	4.943
50370130	PALLD	palladin, cytoskeletal associated protein	5.000
392338862	ATP8B2	ATPase phospholipid transporting 8B2	5.044
755783452	N/A	N/A	5.044
672020628	ATXN7L1	ataxin 7 like 1	5.129

149056134	Dzf17	zinc finger protein 17	5.129
45478072	N/A	N/A	5.129
293356488	RIC1	RIC1 homolog, RAB6A GEF complex partner 1	5.170
564329135	BLM	Bloom syndrome RecQ like helicase	5.248
149066939	PTPRB	protein tyrosine phosphatase, receptor type B	5.248
293349510	STAC	SH3 and cysteine rich domain	5.248
742171392	N/A	N/A	5.248
74184716	Kat6b	K(lysine) acetyltransferase 6B	5.392
392338425	FAM184A	family with sequence similarity 184 member A	5.459
564397808	HNRNPH3	heterogeneous nuclear ribonucleoprotein H3	5.459
293341054	IQCE	IQ motif containing E	5.492
564311685	PIKFYVE	phosphoinositide kinase, FYVE-type zinc finger containing	5.555
293346251	TMEM62	transmembrane protein 62	5.700
755566692	HUWE1	HECT, UBA and WWE domain containing 1, E3 ubiquitin protein ligase	5.728
564309734	IGSF9B	immunoglobulin superfamily member 9B	5.764
149038394	N/A	N/A	5.781
564329926	EMSY	EMSY, BRCA2 interacting transcriptional repressor	5.807
293352381	PAN3	PAN3 poly(A) specific ribonuclease subunit	5.807
149035182	SRP72	signal recognition particle 72	5.807
70778849	Abcg3	ATP-binding cassette, sub-family G (WHITE), member 3	5.833
62647202	KRBA1	KRAB-A domain containing 1	5.833
564301606	RIF1	replication timing regulatory factor 1	5.833
564296997	ZNF45	zinc finger protein 45	5.833
149059984	IL13RA1	interleukin 13 receptor subunit alpha 1	5.907
148687007	CEP128	centrosomal protein 128	5.977
158262015	LCAT	lecithin-cholesterol acyltransferase	5.977
672018101	N/A	N/A	6.000
663243313	N/A	N/A	6.087
564305188	FANCG	Fanconi anemia complementation group G	6.150
124487463	GPR161	G protein-coupled receptor 161	6.150
62656582	KIAA0100	KIAA0100	6.190
672035395	DMWD	dystrophia myotonica, WD repeat containing	6.209
568972626	BPTF	bromodomain PHD finger transcription factor	6.214
149046941	BEND3	BEN domain containing 3	6.229

564311685	PIKFYVE	phosphoinositide kinase, FYVE-type zinc finger containing	6.267
672017085	N/A	N/A	6.304
625198911	N/A	N/A	6.340
672070295	BAHCC1	BAH domain and coiled-coil containing 1	6.375
66730276	C7orf49	chromosome 7 open reading frame 49	6.375
672052120	RBM12B	RNA binding motif protein 12B	6.375
149038409	ACSF3	acyl-CoA synthetase family member 3	6.392
293344867	N/A	N/A	6.443
157821741	LY86	lymphocyte antigen 86	6.524
148682823	N/A	N/A	6.570
149064065	ZMYM5	zinc finger MYM-type containing 5	6.600
672027858	APBB2	amyloid beta precursor protein binding family B member 2	6.629
392342157	PHIP	pleckstrin homology domain interacting protein	6.629
392351087	HAGHL	hydroxyacylglutathione hydrolase-like	6.644
197384571	UBA2	ubiquitin like modifier activating enzyme 2	6.658
564355419	Nbas	neuroblastoma amplified sequence	6.687
755566690	HUWE1	HECT, UBA and WWE domain containing 1, E3 ubiquitin protein ligase	6.700
568914642	N/A	N/A	6.700
672028287	Kat6b	K(lysine) acetyltransferase 6B	6.714
564307792	TECPR2	tectonin beta-propeller repeat containing 2	6.768
6978675	CNTF	ciliary neurotrophic factor	6.794
672046728	N/A	N/A	6.820
569009290	TENM1	teneurin transmembrane protein 1	6.858
564321833	SLC26A8	solute carrier family 26 member 8	6.870
672014312	ZNF646	zinc finger protein 646	6.977
568914628	GARNL3	GTPase activating Rap/RanGAP domain like 3	6.989
149031942	N/A	N/A	6.989
149049553	A2M	alpha-2-macroglobulin	7.044
913507039	N/A	N/A	7.098
149034450	N/A	N/A	7.119
672063750	N/A	N/A	7.143
564297338	ZNF816	zinc finger protein 816	7.180
625182464	N/A	N/A	7.209
880863148	N/A	N/A	7.248
300797930	Tmem223	transmembrane protein 223	7.267
149054972	SLC26A11	solute carrier family 26 member 11	7.401
564316929	FRYL	FRY like transcription coactivator	7.484

568974167	SLC26A11	solute carrier family 26 member 11	7.524
149026222	N/A	N/A	7.531
564322493	RPGR	retinitis pigmentosa GTPase regulator	7.539
149050028	NAA16	N(alpha)-acetyltransferase 16, NatA auxiliary subunit	7.570
149057336	ZSCAN2	zinc finger and SCAN domain containing 2	7.570
148666118	N/A	N/A	7.577
672022615	N/A	N/A	7.585
6690510	CHD9	chromodomain helicase DNA binding protein 9	7.600
568931336	EPB41	erythrocyte membrane protein band 4.1	7.607
62650795	DACT1	dishevelled binding antagonist of beta catenin 1	7.615
672054770	LOC500584	similar to casein kinase 1, gamma 3 isoform 2	7.615
575403049	ERBIN	erbb2 interacting protein	7.622
672069572	KANSL1	KAT8 regulatory NSL complex subunit 1	7.658
564342627	TP53BP1	tumor protein p53 binding protein 1	7.687
568941582	IQSEC1	IQ motif and Sec7 domain 1	7.721
672019127	N/A	N/A	7.794
672013889	KIF7	kinesin family member 7	7.807
392342412	N/A	N/A	7.814
634841768	N/A	N/A	7.858
60360628	ATP8A1	ATPase phospholipid transporting 8A1	7.877
672029702	ZCCHC6	zinc finger CCHC-type containing 6	7.907
148708709	N/A	N/A	7.931
568979792	NPAS3	neuronal PAS domain protein 3	7.954
672032215	REPS2	RALBP1 associated Eps domain containing 2	7.994
28174920	RPL17	ribosomal protein L17	8.006
568979794	NPAS3	neuronal PAS domain protein 3	8.033
564312515	SH3PXD2B	SH3 and PX domains 2B	8.050
157819803	BACH1	BTB domain and CNC homolog 1	8.087
149015786	N/A	N/A	8.087
109472884	UBE3C	ubiquitin protein ligase E3C	8.155
124487463	GPR161	G protein-coupled receptor 161	8.165
440913556	N/A	N/A	8.185
148675846	FAM114A2	family with sequence similarity 114 member A2	8.190
608785644	NPAS3	neuronal PAS domain protein 3	8.195
921126126	N/A	N/A	8.195
672022290	KIF21A	kinesin family member 21A	8.219
293347435	PTPRD	protein tyrosine phosphatase, receptor type D	8.224
672022270	KIF21A	kinesin family member 21A	8.317
564378315	Zfp853	zinc finger protein 853	8.375

564297942	LOC103690302	AF4/FMR2 family member 2	8.379
149066285	PHF20L1	PHD finger protein 20-like 1	8.430
806549497	MYH9	myosin heavy chain 9	8.455
568979594	SYT16	synaptotagmin 16	8.551
392340959	ITSN2	intersectin 2	8.570
564380050	2410141K09Rik	RIKEN cDNA 2410141K09 gene	8.596
325530254	FAM109A	family with sequence similarity 109 member A	8.669
149064065	ZMYM5	zinc finger MYM-type containing 5	8.683
564317999	N/A	N/A	8.725
149051960	FLYWCH2	FLYWCH family member 2	8.762
672022290	KIF21A	kinesin family member 21A	8.814
564377502	EIF4G1	eukaryotic translation initiation factor 4 gamma 1	8.994
392341692	FMNL3	formin like 3	9.071
927135720	N/A	N/A	9.095
148675846	FAM114A2	family with sequence similarity 114 member A2	9.157
755541276	N/A	N/A	9.197
987404821	N/A	N/A	9.311
672033554	LOC102557335	uncharacterized LOC102557335	9.713
672035060	CIC	capicua transcriptional repressor	10.209

Supplementary Table S2. The lists of BPA-responsive genes that have been associated with ASD in SFARI database.

ASD-related DEGs in both sexes				
**ID**	**Symbol**	**Entrez Gene Name**	**Expr Log Ratio**	**Chromosome Location**
672035060	CIC	capicua transcriptional repressor	-8.114	1q21
755566690	HUWE1	HECT, UBA and WWE domain containing 1, E3 ubiquitin protein ligase	-7.864	Xq13
226698394	UNC80	unc-80 homolog, NALCN channel complex subunit	-7.349	9q32
564315185	CUX1	cut like homeobox 1	-7.209	12q12
564303135	KMT2C	lysine methyltransferase 2C	-6.870	4q11
149065087	CADPS2	calcium dependent secretion activator 2	-5.883	4q22
564329920	EMSY	EMSY, BRCA2 interacting transcriptional repressor	-5.585	1q32
148668227	GPC6	glypican 6	-5.248	15q24
672066092	UNC80	unc-80 homolog, NALCN channel complex subunit	-4.954	9q32
672035060	CIC	capicua transcriptional repressor	-4.863	1q21
564329926	EMSY	EMSY, BRCA2 interacting transcriptional repressor	-4.655	1q32
392334411	ANKRD11	ankyrin repeat domain 11	-4.358	19q12
54019438	PCDHAC1	protocadherin alpha subfamily C, 1	-3.170	18p11
124486586	AUTS2	AUTS2, activator of transcription and developmental regulator	-2.742	12q12
291327518	AVP	arginine vasopressin	-2.734	3q36
564303143	KMT2C	lysine methyltransferase 2C	-2.497	4q11
392334411	ANKRD11	ankyrin repeat domain 11	-2.301	19q12
755566692	HUWE1	HECT, UBA and WWE domain containing 1, E3 ubiquitin protein ligase	-2.222	Xq13
198278465	DYDC2	DPY30 domain containing 2	-2.190	16p14
25742828	SCN7A	sodium voltage-gated channel alpha subunit 7	-2.129	3q21
19424304	CHRNB3	cholinergic receptor nicotinic beta 3 subunit	-2.000	16q12.3
149066014	PVALB	parvalbumin	-1.939	7q34
226698394	UNC80	unc-80 homolog, NALCN channel complex subunit	-1.781	9q32

54019438	PCDHAC1	protocadherin alpha subfamily C, 1	-1.704	18p11
392332008	SLC38A10	solute carrier family 38 member 10	-1.568	10q32.3
158186711	F13A1	coagulation factor XIII A chain	-1.510	17p12
6981148	LEP	leptin	-1.436	4q22
672065933	DOCK10	dedicator of cytokinesis 10	-1.436	9q34
755566692	HUWE1	HECT, UBA and WWE domain containing 1, E3 ubiquitin protein ligase	-1.410	Xq13
672061705	KMT2A	lysine methyltransferase 2A	-1.326	8q22
300794534	WDR93	WD repeat domain 93	-1.322	1q31
158187533	ABCA13	ATP binding cassette subfamily A member 13	-1.322	14q21
148689488	SYN3	synapsin III	-1.308	7q13
564329920	EMSY	EMSY, BRCA2 interacting transcriptional repressor	-1.275	1q32
6981332	SERPINE1	serpin family E member 1	-1.184	12q12
25453414	ASS1	argininosuccinate synthase 1	-1.000	3p12
149025238	ESRRB	estrogen related receptor beta	-1.000	6q31
149053793	TSPOAP1	TSPO associated protein 1	-0.970	10q26
124486586	AUTS2	AUTS2, activator of transcription and developmental regulator	-0.941	12q12
564329916	EMSY	EMSY, BRCA2 interacting transcriptional repressor	-0.864	1q32
56605720	GADD45B	growth arrest and DNA damage inducible beta	-0.828	7q11
46485412	ABCA7	ATP binding cassette subfamily A member 7	-0.798	7q11
109480098	SMARCC2	SWI/SNF related, matrix associated, actin dependent regulator of chromatin subfamily c member 2	-0.795	7q11
18426812	ADA	adenosine deaminase	-0.787	3q42
157819203	TECTA	tectorin alpha	-0.778	8q22
55741827	TERT	telomerase reverse transcriptase	-0.771	1p11
58865632	ARHGAP24	Rho GTPase activating protein 24	-0.758	14p22
157819949	ITGA4	integrin subunit alpha 4	-0.748	3q24
46485412	ABCA7	ATP binding cassette subfamily A member 7	-0.739	7q11
157824208	NTNG1	netrin G1	-0.737	2q41
19173756	ERG	ERG, ETS transcription factor	-0.718	11q11
148671621	VIP	vasoactive intestinal peptide	-0.696	1q11

149062990	CUX1	cut like homeobox 1	-0.682	12q12
67514566	POLA2	DNA polymerase alpha 2, accessory subunit	-0.665	1q43
158262033	PAH	phenylalanine hydroxylase	-0.655	7q13
157822365	LAMC3	laminin subunit gamma 3	-0.651	3p12
13591940	DPYD	dihydropyrimidine dehydrogenase	-0.642	2q41
25742776	MC4R	melanocortin 4 receptor	-0.641	18q12.1
672076564	DDC	dopa decarboxylase	-0.632	14q21
157824208	NTNG1	netrin G1	-0.628	2q41
149053793	TSPOAP1	TSPO associated protein 1	-0.627	10q26
157819949	ITGA4	integrin subunit alpha 4	-0.620	3q24
108935976	DISC1	disrupted in schizophrenia 1	-0.590	19q12
110347493	PCDHA9	protocadherin alpha 9	-0.587	18p11
672035060	CIC	capicua transcriptional repressor	-0.579	1q21
293346999	MBD4	methyl-CpG binding domain 4, DNA glycosylase	-0.573	4q42
213513304	ATP10A	ATPase phospholipid transporting 10A (putative)	-0.554	1q22
6981180	MAOB	monoamine oxidase B	-0.518	Xq11
564313842	SLC38A10	solute carrier family 38 member 10	-0.503	10q32.3
564330609	SYT17	synaptotagmin 17	-0.494	1q35
6978867	GABRB1	gamma-aminobutyric acid type A receptor beta1 subunit	-0.487	14p11
392332443	PRKDC	protein kinase, DNA-activated, catalytic polypeptide	-0.475	11q23
312922379	TNN	tenascin N	-0.469	13q22
13562118	LRP2	LDL receptor related protein 2	-0.454	3q21
13027400	GUCY1A2	guanylate cyclase 1 soluble subunit alpha 2	-0.453	8q11
392341425	PTPRB	protein tyrosine phosphatase, receptor type B	-0.452	7q22
158138494	PTPRC	protein tyrosine phosphatase, receptor type C	-0.451	13q13
149026322	PTGER3	prostaglandin E receptor 3	-0.445	2q45
78126167	SLC1A2	solute carrier family 1 member 2	-0.445	3q32
51854227	GSN	gelsolin	-0.444	3p11
6978629	CD38	CD38 molecule	-0.440	14q21
198386351	TBC1D31	TBC1 domain family member 31	-0.439	7q33
51854227	GSN	gelsolin	-0.431	3p11
110347493	PCDHA9	protocadherin alpha 9	-0.429	18p11
157823279	CGNL1	cingulin like 1	-0.418	8q24
13994179	SLC24A2	solute carrier family 24 member 2	-0.416	5q32

748983333	PATJ	PATJ, crumbs cell polarity complex component	-0.415	5q33
672041704	NIPBL	NIPBL, cohesin loading factor	-0.412	2q16
71043794	CEP41	centrosomal protein 41	-0.410	4q22
13591949	GATM	glycine amidinotransferase	-0.409	3q35
78126167	SLC1A2	solute carrier family 1 member 2	-0.408	3q32
122065191	ABAT	4-aminobutyrate aminotransferase	-0.401	10q12
77695926	STAT1	signal transducer and activator of transcription 1	-0.399	9q22
451172073	CHRM3	cholinergic receptor muscarinic 3	-0.399	17q12.1
28972652	SLC12A5	solute carrier family 12 member 5	-0.394	3q42
11177892	KCNT1	potassium sodium-activated channel subfamily T member 1	-0.392	3p13
149052857	KCNJ12	potassium voltage-gated channel subfamily J member 12	-0.392	10q22
110347559	PCDHA13	protocadherin alpha 13	-0.391	18p11
166999225	GRM1	glutamate metabotropic receptor 1	-0.385	1p13
158303308	PCCA	propionyl-CoA carboxylase alpha subunit	-0.369	15q25
54019428	PCDHA5	protocadherin alpha 5	-0.366	18p11
13591949	GATM	glycine amidinotransferase	-0.365	3q35
449784888	ALDH5A1	aldehyde dehydrogenase 5 family member A1	-0.363	17p11
25453410	CACNA1B	calcium voltage-gated channel subunit alpha1 B	-0.357	3p13
157822187	WWOX	WW domain containing oxidoreductase	-0.355	19q12
348041395	DLGAP2	DLG associated protein 2	-0.354	16q12.5
197927216	TBC1D5	TBC1 domain family member 5	-0.351	9q11
755566690	HUWE1	HECT, UBA and WWE domain containing 1, E3 ubiquitin protein ligase	-0.351	Xq13
149053793	TSPOAP1	TSPO associated protein 1	-0.349	10q26
148747194	SLC16A7	solute carrier family 16 member 7	-0.349	7q22
451172073	CHRM3	cholinergic receptor muscarinic 3	-0.349	17q12.1
122065191	ABAT	4-aminobutyrate aminotransferase	-0.348	10q12
55741540	KATNAL1	katanin catalytic subunit A1 like 1	-0.345	12p11
568956384	ADAMTS18	ADAM metallopeptidase with thrombospondin type 1 motif 18	-0.345	19q12
300795140	TAF1	TATA-box binding protein associated factor 1	-0.342	Xq22

815891312	CACNA1G	calcium voltage-gated channel subunit alpha1 G	-0.340	10q26
6981208	NR3C2	nuclear receptor subfamily 3 group C member 2	-0.328	19q11
61557206	ZBTB16	zinc finger and BTB domain containing 16	-0.326	8q23
148705386	SLC30A3	solute carrier family 30 member 3	-0.318	6q14
672043401	POGZ	pogo transposable element derived with ZNF domain	-0.317	2q34
63101489	ACHE	acetylcholinesterase (Cartwright blood group)	-0.310	12q12
13994179	SLC24A2	solute carrier family 24 member 2	-0.310	5q32
6981168	LPL	lipoprotein lipase	-0.308	16p14
109480098	SMARCC2	SWI/SNF related, matrix associated, actin dependent regulator of chromatin subfamily c member 2	-0.307	7q11
77628037	PEX7	peroxisomal biogenesis factor 7	-0.307	1p12
564326692	ZC3H4	zinc finger CCCH-type containing 4	-0.306	1q21
115292425	KIRREL3	kirre like nephrin family adhesion molecule 3	-0.305	8q21
755566690	HUWE1	HECT, UBA and WWE domain containing 1, E3 ubiquitin protein ligase	-0.305	Xq13
6978789	SPARCL1	SPARC like 1	-0.295	14p22
149039905	TSPAN17	tetraspanin 17	-0.294	17p14
28972363	DOCK4	dedicator of cytokinesis 4	-0.292	6q21
51948412	ETFB	electron transfer flavoprotein beta subunit	-0.286	1q22
6981208	NR3C2	nuclear receptor subfamily 3 group C member 2	-0.286	19q11
28972652	SLC12A5	solute carrier family 12 member 5	-0.285	3q42
16758138	POMT1	protein O-mannosyltransferase 1	-0.285	3p12
50356003	SCP2	sterol carrier protein 2	-0.284	5q34
564319191	MCPH1	microcephalin 1	-0.284	16q12.5
261599034	IL1RAPL2	interleukin 1 receptor accessory protein like 2	-0.280	Xq32
16758716	CACNB2	calcium voltage-gated channel auxiliary subunit beta 2	-0.279	17q12.3
18959266	KHDRBS2	KH RNA binding domain containing, signal transduction associated 2	-0.274	9q21
157820049	LRFN5	leucine rich repeat and fibronectin type III domain containing 5	-0.271	6q23

158508684	BCAS1	breast carcinoma amplified sequence 1	-0.270	3q42
219804406	DOCK1	dedicator of cytokinesis 1	-0.269	1q41
392339847	CADPS2	calcium dependent secretion activator 2	-0.268	4q22
10242377	GRIK4	glutamate ionotropic receptor kainate type subunit 4	-0.267	8q22
13786142	SLIT3	slit guidance ligand 3	-0.266	10q12
564382316	HSD11B1	hydroxysteroid 11-beta dehydrogenase 1	-0.265	13q27
672037217	OTUD7A	OTU deubiquitinase 7A	-0.264	1q22
148668227	GPC6	glypican 6	-0.264	15q24
115292425	KIRREL3	kirre like nephrin family adhesion molecule 3	-0.263	8q21
140971205	GRIN2A	glutamate ionotropic receptor NMDA type subunit 2A	-0.255	10q11
157817710	FER	FER tyrosine kinase	-0.251	9q37
300795140	TAF1	TATA-box binding protein associated factor 1	-0.248	Xq22
158749644	MCM6	minichromosome maintenance complex component 6	-0.247	13q13
59858990	UNC13A	unc-13 homolog A	-0.246	16p14
54312094	DAGLA	diacylglycerol lipase alpha	-0.244	1q43
564391295	DUSP22	dual specificity phosphatase 22	-0.244	17p12
25742807	RASSF5	Ras association domain family member 5	-0.241	13q13
97537309	SYNJ1	synaptojanin 1	-0.240	11q11
449784888	ALDH5A1	aldehyde dehydrogenase 5 family member A1	-0.236	17p11
158749644	MCM6	minichromosome maintenance complex component 6	-0.231	13q13
157817839	SEMA5A	semaphorin 5A	-0.230	2q22
197927216	TBC1D5	TBC1 domain family member 5	-0.230	9q11
157817710	FER	FER tyrosine kinase	-0.225	9q37
13928806	P2RX4	purinergic receptor P2X 4	-0.223	12q16
672040941	ATRNL1	attractin like 1	-0.223	1q55
148695758	CAPRIN1	cell cycle associated protein 1	-0.221	3q32
19705535	PACS1	phosphofurin acidic cluster sorting protein 1	-0.220	1q43
54312094	DAGLA	diacylglycerol lipase alpha	-0.218	1q43
11560055	KHDRBS3	KH RNA binding domain containing, signal transduction associated 3	-0.217	7q34

157821397	SLC22A15	solute carrier family 22 member 15	-0.216	2q34
149066531	VPS13B	vacuolar protein sorting 13 homolog B	-0.212	7q22
157817839	SEMA5A	semaphorin 5A	-0.212	2q22
281306814	RPS6KA2	ribosomal protein S6 kinase A2	-0.210	1q12
19705535	PACS1	phosphofurin acidic cluster sorting protein 1	-0.208	1q43
157822271	DOLK	dolichol kinase	-0.207	3p12
37359962	PLPPR4	phospholipid phosphatase related 4	-0.207	2q41
39104626	CAMK2A	calcium/calmodulin dependent protein kinase II alpha	-0.206	18q12.1
39104626	CAMK2A	calcium/calmodulin dependent protein kinase II alpha	-0.206	18q12.1
402794666	NRG1	neuregulin 1	-0.205	16q12.3
54019432	PCDHA7	protocadherin alpha 7	-0.205	18p11
6978673	CNR1	cannabinoid receptor 1	-0.205	5q21
148747414	GDA	guanine deaminase	-0.203	1q51
149020512	PDE4A	phosphodiesterase 4A	-0.203	8q13
568971594	GABRA1	gamma-aminobutyric acid type A receptor alpha1 subunit	-0.203	10q21
564387864	CACNA1D	calcium voltage-gated channel subunit alpha1 D	-0.199	16p16
59858990	UNC13A	unc-13 homolog A	-0.199	16p14
77404395	SND1	staphylococcal nuclease and tudor domain containing 1	-0.199	4q22
148667088	ATP2B2	ATPase plasma membrane Ca2+ transporting 2	-0.199	4q42
109472884	UBE3C	ubiquitin protein ligase E3C	-0.198	4q11
26006243	KCND2	potassium voltage-gated channel subfamily D member 2	-0.197	4q22
672040941	ATRNL1	attractin like 1	-0.196	1q55
11560055	KHDRBS3	KH RNA binding domain containing, signal transduction associated 3	-0.195	7q34
56605990	LRPPRC	leucine rich pentatricopeptide repeat containing	-0.194	6q12
13591963	GRM7	glutamate metabotropic receptor 7	-0.191	4q41
56090379	POMGNT1	protein O-linked mannose N-acetylglucosaminyltransferase 1 (beta 1,2-)	-0.191	5q35
148695758	CAPRIN1	cell cycle associated protein 1	-0.188	3q32
169234826	ELP4	elongator acetyltransferase complex subunit 4	-0.186	3q33

300796855	PARD3B	par-3 family cell polarity regulator beta	-0.183	9q32
281306738	PCDH19	protocadherin 19	-0.176	Xq32
50356003	SCP2	sterol carrier protein 2	-0.170	5q34
9665227	DLG4	discs large MAGUK scaffold protein 4	-0.170	10q24
62945358	SCFD2	sec1 family domain containing 2	-0.165	14p11
77404395	SND1	staphylococcal nuclease and tudor domain containing 1	-0.165	4q22
672088848	PLXNA3	plexin A3	-0.163	Xq37
124486885	LRRC7	leucine rich repeat containing 7	-0.162	2q45
157820049	LRFN5	leucine rich repeat and fibronectin type III domain containing 5	-0.161	6q23
148673176	FABP7	fatty acid binding protein 7	-0.158	20q12
6981168	LPL	lipoprotein lipase	-0.158	16p14
194474054	EFR3A	EFR3 homolog A	-0.157	7q33
37359962	PLPPR4	phospholipid phosphatase related 4	-0.157	2q41
97537309	SYNJ1	synaptojanin 1	-0.152	11q11
41281619	PCDHA10	protocadherin alpha 10	-0.149	18p11
564388440	MYO9B	myosin IXB	-0.148	16p14
717324516	SCN8A	sodium voltage-gated channel alpha subunit 8	-0.147	7q36
6980978	GPD2	glycerol-3-phosphate dehydrogenase 2	-0.146	3q21
288806592	PRKCB	protein kinase C beta	-0.143	1q36
109472884	UBE3C	ubiquitin protein ligase E3C	-0.142	4q11
815891312	CACNA1G	calcium voltage-gated channel subunit alpha1 G	-0.142	10q26
40254595	DPYSL2	dihydropyrimidinase like 2	-0.141	15p12
16758736	NLGN1	neuroligin 1	-0.139	2q24
690969208	MCC	mutated in colorectal cancers	-0.134	18p11
690969208	MCC	mutated in colorectal cancers	-0.134	18p11
148747541	HNRNPU	heterogeneous nuclear ribonucleoprotein U	-0.130	13q25
6981520	SDC2	syndecan 2	-0.128	7q22
25453410	CACNA1B	calcium voltage-gated channel subunit alpha1 B	-0.128	3p13
114052913	CADM2	cell adhesion molecule 2	-0.126	11p12
149053938	SLC35B1	solute carrier family 35 member B1	-0.125	10q26
253683488	NTRK2	neurotrophic receptor tyrosine kinase 2	-0.124	17p14
564326713	ZC3H4	zinc finger CCCH-type containing 4	-0.121	1q21
166158339	REEP3	receptor accessory protein 3	-0.121	20p11

253683488	NTRK2	neurotrophic receptor tyrosine kinase 2	-0.121	17p14
476007242	EPS8	epidermal growth factor receptor pathway substrate 8	-0.117	4q44
8393390	GABRB3	gamma-aminobutyric acid type A receptor beta3 subunit	-0.115	1q22
56605990	LRPPRC	leucine rich pentatricopeptide repeat containing	-0.115	6q12
29789269	GRIA1	glutamate ionotropic receptor AMPA type subunit 1	-0.115	10q22
11560079	KIT	KIT proto-oncogene receptor tyrosine kinase	-0.114	14p11
148747541	HNRNPU	heterogeneous nuclear ribonucleoprotein U	-0.109	13q25
148673176	FABP7	fatty acid binding protein 7	-0.107	20q12
9665227	DLG4	discs large MAGUK scaffold protein 4	-0.101	10q24
157818589	TSPAN7	tetraspanin 7	-0.100	Xq12
300794843	IQGAP3	IQ motif containing GTPase activating protein 3	-0.100	2q34
224967068	PLCB1	phospholipase C beta 1	-0.098	3q36
40254595	DPYSL2	dihydropyrimidinase like 2	-0.096	15p12
281427192	CDH11	cadherin 11	-0.096	19p14
748585198	DLGAP1	DLG associated protein 1	-0.094	9q38
564350262	CHD7	chromodomain helicase DNA binding protein 7	-0.088	5q13
12408298	DPP6	dipeptidyl peptidase like 6	-0.086	4q11
149022387	NCKAP1	NCK associated protein 1	-0.084	3q24
148747194	SLC16A7	solute carrier family 16 member 7	-0.080	7q22
220777	STX1A	syntaxin 1A	-0.080	12q12
568986834	KCNMA1	potassium calcium-activated channel subfamily M alpha 1	-0.075	15p16
564375060	SLC39A11	solute carrier family 39 member 11	-0.070	10q32.1
672065682	AGAP1	ArfGAP with GTPase domain, ankyrin repeat and PH domain 1	-0.053	9q35-q36
29789269	GRIA1	glutamate ionotropic receptor AMPA type subunit 1	-0.052	10q22
83320121	RBM8A	RNA binding motif protein 8A	-0.052	2q34
953713397	AGO1	argonaute 1, RISC catalytic component	-0.045	5q36
6978673	CNR1	cannabinoid receptor 1	-0.023	5q21
672046314	AMBRA1	autophagy and beclin 1 regulator 1	0.034	3q24
9506875	SMAD4	SMAD family member 4	0.047	18q12.2

25742663	MYT1L	myelin transcription factor 1 like	0.052	6q16
20544149	CSNK1D	casein kinase 1 delta	0.053	10q32.3
50511039	GNB1L	G protein subunit beta 1 like	0.054	11q23
672073969	CLASP1	cytoplasmic linker associated protein 1	0.057	13q11
62078949	GPC4	glypican 4	0.063	Xq36
25742568	DPYSL3	dihydropyrimidinase like 3	0.069	18p11-q11
672044697	EHMT1	euchromatic histone lysine methyltransferase 1	0.070	3p13
89363040	PCDHGA11	protocadherin gamma subfamily A, 11	0.075	18p11
672075032	MARK1	microtubule affinity regulating kinase 1	0.089	13q26
112807209	EXT1	exostosin glycosyltransferase 1	0.090	7q31
149016466	NIPBL	NIPBL, cohesin loading factor	0.092	2q16
22024394	FABP5	fatty acid binding protein 5	0.092	2q23
164565398	RBFOX1	RNA binding fox-1 homolog 1	0.101	10q12
86477155	PER1	period circadian regulator 1	0.108	10q24
564320563	RBM27	RNA binding motif protein 27	0.109	18p11
30794434	SRRM4	serine/arginine repetitive matrix 4	0.112	12q16
47847438	EXOC3	exocyst complex component 3	0.118	1p11
18959272	KCNQ2	potassium voltage-gated channel subfamily Q member 2	0.119	3q43
300793721	TLK2	tousled like kinase 2	0.119	10q32.1
393716310	WAC	WW domain containing adaptor with coiled-coil	0.124	17q12.1
564361244	TCF20	transcription factor 20	0.128	7q34
51036652	SLC33A1	solute carrier family 33 member 1	0.128	2q31
672063411	SETD2	SET domain containing 2	0.131	8q32
157821015	KDM5B	lysine demethylase 5B	0.131	13q13
11177894	TSC1	TSC complex subunit 1	0.134	3p12
50510937	MBD5	methyl-CpG binding domain protein 5	0.134	3q12
8394405	SLC7A5	solute carrier family 7 member 5	0.134	19q12
148491097	DYNC1H1	dynein cytoplasmic 1 heavy chain 1	0.137	6q32
171543899	PLXNA4	plexin A4	0.139	4q22
213972547	KAT6A	lysine acetyltransferase 6A	0.143	16q12.5
157819885	SETD5	SET domain containing 5	0.144	4q42
291042494	MED13L	mediator complex subunit 13 like	0.145	12q16
157819885	SETD5	SET domain containing 5	0.150	4q42
404312698	CASC4	cancer susceptibility 4	0.150	3q35
171543899	PLXNA4	plexin A4	0.156	4q22

564393951	MBD1	methyl-CpG binding domain protein 1	0.157	18q12.2
300793740	TANC2	tetratricopeptide repeat, ankyrin repeat and coiled-coil containing 2	0.158	10q32.1
149038734	EIF4EBP2	eukaryotic translation initiation factor 4E binding protein 2	0.158	20q11
19705483	CLSTN2	calsyntenin 2	0.160	8q31
300793740	TANC2	tetratricopeptide repeat, ankyrin repeat and coiled-coil containing 2	0.160	10q32.1
124487247	PRICKLE2	prickle planar cell polarity protein 2	0.162	4q34
672042487	NBEA	neurobeachin	0.163	2q26
564386027	CHD8	chromodomain helicase DNA binding protein 8	0.163	15p14
8393104	CHKB	choline kinase beta	0.166	7q34
33356154	UBE2H	ubiquitin conjugating enzyme E2 H	0.172	4q22
568972665	TSPOAP1	TSPO associated protein 1	0.173	10q26
564303143	KMT2C	lysine methyltransferase 2C	0.176	4q11
209915609	PRICKLE1	prickle planar cell polarity protein 1	0.177	7q35
6978787	DYRK1A	dual specificity tyrosine phosphorylation regulated kinase 1A	0.179	11q11
61556927	EIF3G	eukaryotic translation initiation factor 3 subunit G	0.180	8q13
9506755	GRIK2	glutamate ionotropic receptor kainate type subunit 2	0.181	20q13
157821015	KDM5B	lysine demethylase 5B	0.182	13q13
568972475	NCOR1	nuclear receptor corepressor 1	0.185	10q23
564333605	BTAF1	B-TFIID TATA-box binding protein associated factor 1	0.189	1q53
157819605	EPC2	enhancer of polycomb homolog 2	0.190	3q12
403310664	KMT2E	lysine methyltransferase 2E	0.194	4q11
157818041	YEATS2	YEATS domain containing 2	0.195	11q23
119616373	MEF2C	myocyte enhancer factor 2C	0.200	2q11
291042494	MED13L	mediator complex subunit 13 like	0.202	12q16
672031398	ANKRD11	ankyrin repeat domain 11	0.203	19q12
145558904	EML1	echinoderm microtubule associated protein like 1	0.205	6q32
425384	CAMK4	calcium/calmodulin dependent protein kinase IV	0.207	18p12
19705483	CLSTN2	calsyntenin 2	0.214	8q31
24025618	DAB1	DAB1, reelin adaptor protein	0.217	5q33-q34

564386027	CHD8	chromodomain helicase DNA binding protein 8	0.219	15p14
2804296	CDH8	cadherin 8	0.221	19p13
2804296	CDH8	cadherin 8	0.221	19p13
564361244	TCF20	transcription factor 20	0.222	7q34
672026667	CUX1	cut like homeobox 1	0.225	12q12
148491097	DYNC1H1	dynein cytoplasmic 1 heavy chain 1	0.225	6q32
51871603	ST7	suppression of tumorigenicity 7	0.225	4q21
157819605	EPC2	enhancer of polycomb homolog 2	0.227	3q12
6978787	DYRK1A	dual specificity tyrosine phosphorylation regulated kinase 1A	0.228	11q11
11177894	TSC1	TSC complex subunit 1	0.230	3p12
157817961	PHF3	PHD finger protein 3	0.230	9q21
213972547	KAT6A	lysine acetyltransferase 6A	0.236	16q12.5
157817961	PHF3	PHD finger protein 3	0.254	9q21
157821435	RANBP17	RAN binding protein 17	0.256	10q12
564328896	CHD2	chromodomain helicase DNA binding protein 2	0.258	1q31
74180575	PPM1D	protein phosphatase, Mg2+/Mn2+ dependent 1D	0.260	10q26
425384	CAMK4	calcium/calmodulin dependent protein kinase IV	0.262	18p12
403310664	KMT2E	lysine methyltransferase 2E	0.263	4q11
8393652	KCNJ2	potassium voltage-gated channel subfamily J member 2	0.267	10q32.1
31543579	RELN	reelin	0.268	4q11
404312698	CASC4	cancer susceptibility 4	0.270	3q35
157819717	NIPA2	non imprinted in Prader-Willi/Angelman syndrome 2	0.270	1q22
149022827	PAX6	paired box 6	0.271	3q33
157823369	TERF2	telomeric repeat binding factor 2	0.272	19q12
9506755	GRIK2	glutamate ionotropic receptor kainate type subunit 2	0.274	20q13
29612542	H2AFZ	H2A histone family member Z	0.285	2q44
157817660	EBF3	early B cell factor 3	0.288	1q41
148687364	Cux1	cut-like homeobox 1	0.288	12q12
34328151	TBR1	T-box, brain 1	0.295	3q21
13929168	FAT1	FAT atypical cadherin 1	0.300	16q11
982920935	DLX6	distal-less homeobox 6	0.301	4q21
13929168	FAT1	FAT atypical cadherin 1	0.305	16q11
157818041	YEATS2	YEATS domain containing 2	0.309	11q23
672035060	CIC	capicua transcriptional repressor	0.325	1q21

157824124	NUAK1	NUAK family kinase 1	0.335	7q13
564303143	KMT2C	lysine methyltransferase 2C	0.336	4q11
29612542	H2AFZ	H2A histone family member Z	0.337	2q44
51871603	ST7	suppression of tumorigenicity 7	0.339	4q21
164518930	SDK1	sidekick cell adhesion molecule 1	0.347	12q11
157820017	FBXO33	F-box protein 33	0.359	6q23
672031398	ANKRD1 1	ankyrin repeat domain 11	0.367	19q12
564328896	CHD2	chromodomain helicase DNA binding protein 2	0.370	1q31
672061705	KMT2A	lysine methyltransferase 2A	0.372	8q22
157817845	KMT5B	lysine methyltransferase 5B	0.374	1q43
62078501	TTI2	TELO2 interacting protein 2	0.376	16q12.3
564333605	BTAF1	B-TFIID TATA-box binding protein associated factor 1	0.392	1q53
40789237	PCDHA4	protocadherin alpha 4	0.398	18p11
11560016	HTR1B	5-hydroxytryptamine receptor 1B	0.402	8q31
149016466	NIPBL	NIPBL, cohesin loading factor	0.407	2q16
148747270	PTGS2	prostaglandin-endoperoxide synthase 2	0.408	13q21
157817845	KMT5B	lysine methyltransferase 5B	0.413	1q43
16758574	CNTN5	contactin 5	0.414	8q11
40789237	PCDHA4	protocadherin alpha 4	0.419	18p11
293348214	CCDC88C	coiled-coil domain containing 88C	0.421	6q32
568940286	BRAF	B-Raf proto-oncogene, serine/threonine kinase	0.444	4q22
31543579	RELN	reelin	0.461	4q11
672061705	KMT2A	lysine methyltransferase 2A	0.498	8q22
11560065	GPR85	G protein-coupled receptor 85	0.502	4q21
564315667	CLASP1	cytoplasmic linker associated protein 1	0.555	13q11
62078501	TTI2	TELO2 interacting protein 2	0.561	16q12.3
13928942	PER2	period circadian regulator 2	0.562	9q36
157822673	HS3ST5	heparan sulfate-glucosamine 3-sulfotransferase 5	0.577	20q12
564315183	CUX1	cut like homeobox 1	0.585	12q12
672066092	UNC80	unc-80 homolog, NALCN channel complex subunit	0.589	9q32
564303143	KMT2C	lysine methyltransferase 2C	0.593	4q11
13928942	PER2	period circadian regulator 2	0.600	9q36
148689488	SYN3	synapsin III	0.627	7q13
28972363	DOCK4	dedicator of cytokinesis 4	0.647	6q21
564327667	TSHZ3	teashirt zinc finger homeobox 3	0.664	1q21

149058952	MEF2C	myocyte enhancer factor 2C	0.716	2q11
564389875	ARHGEF 10	Rho guanine nucleotide exchange factor 10	0.734	16q12.5
20302047	AMPD1	adenosine monophosphate deaminase 1	0.737	2q34
564315183	CUX1	cut like homeobox 1	0.748	12q12
755566692	HUWE1	HECT, UBA and WWE domain containing 1, E3 ubiquitin protein ligase	0.787	Xq13
300795362	PTCHD1	patched domain containing 1	0.820	Xq21
157818841	POGZ	pogo transposable element derived with ZNF domain	0.842	2q34
392332010	SLC38A1 0	solute carrier family 38 member 10	0.955	10q32.3
755515866	BRAF	B-Raf proto-oncogene, serine/threonine kinase	0.990	4q22
564303143	KMT2C	lysine methyltransferase 2C	0.997	4q11
672061705	KMT2A	lysine methyltransferase 2A	1.003	8q22
149058952	MEF2C	myocyte enhancer factor 2C	1.126	2q11
12083683	SRD5A2	steroid 5 alpha-reductase 2	1.128	6q14
755566692	HUWE1	HECT, UBA and WWE domain containing 1, E3 ubiquitin protein ligase	1.130	Xq13
157787081	WNT1	Wnt family member 1	1.222	7q36
293348214	CCDC88C	coiled-coil domain containing 88C	1.293	6q32
755566690	HUWE1	HECT, UBA and WWE domain containing 1, E3 ubiquitin protein ligase	1.299	Xq13
106879208	MYH4	myosin heavy chain 4	1.415	10q24
564329918	EMSY	EMSY, BRCA2 interacting transcriptional repressor	1.449	1q32
568972665	TSPOAP1	TSPO associated protein 1	1.480	10q26
155369646	AGBL4	ATP/GTP binding protein like 4	1.510	5q35
24308466	ITGB3	integrin subunit beta 3	1.596	10q32.1
575403049	ERBIN	erbb2 interacting protein	1.596	2q12
568990288	NIPBL	NIPBL, cohesin loading factor	1.663	2q16
755566690	HUWE1	HECT, UBA and WWE domain containing 1, E3 ubiquitin protein ligase	1.675	Xq13
157817658	VIL1	villin 1	1.700	9q33
564329926	EMSY	EMSY, BRCA2 interacting transcriptional repressor	1.703	1q32
568990288	NIPBL	NIPBL, cohesin loading factor	1.705	2q16

564329926	EMSY	EMSY, BRCA2 interacting transcriptional repressor	2.094	1q32
109480102	SMARCC2	SWI/SNF related, matrix associated, actin dependent regulator of chromatin subfamily c member 2	2.178	7q11
564329920	EMSY	EMSY, BRCA2 interacting transcriptional repressor	2.185	1q32
392342157	PHIP	pleckstrin homology domain interacting protein	2.226	8q31
564303135	KMT2C	lysine methyltransferase 2C	2.505	4q11
564329920	EMSY	EMSY, BRCA2 interacting transcriptional repressor	2.899	1q32
564329920	EMSY	EMSY, BRCA2 interacting transcriptional repressor	3.112	1q32
672048712	KMT2C	lysine methyltransferase 2C	3.209	4q11
564392795	MOCOS	molybdenum cofactor sulfurase	3.248	18p12
11024666	NTRK1	neurotrophic receptor tyrosine kinase 1	4.285	2q34
568906591	UNC80	unc-80 homolog, NALCN channel complex subunit	4.755	9q32
392333013	CEP135	centrosomal protein 135	4.858	14p11
8394516	PLAUR	plasminogen activator, urokinase receptor	5.229	1q21
564303143	KMT2C	lysine methyltransferase 2C	5.322	4q11
564329918	EMSY	EMSY, BRCA2 interacting transcriptional repressor	5.476	1q32
564315188	CUX1	cut like homeobox 1	6.267	12q12
564313845	SLC38A10	solute carrier family 38 member 10	6.807	10q32.3
755566690	HUWE1	HECT, UBA and WWE domain containing 1, E3 ubiquitin protein ligase	7.555	Xq13
564329920	EMSY	EMSY, BRCA2 interacting transcriptional repressor	7.728	1q32
109472884	UBE3C	ubiquitin protein ligase E3C	8.197	4q11
109472884	UBE3C	ubiquitin protein ligase E3C	8.238	4q11
755566692	HUWE1	HECT, UBA and WWE domain containing 1, E3 ubiquitin protein ligase	8.326	Xq13
109480102	SMARCC2	SWI/SNF related, matrix associated, actin dependent regulator of chromatin subfamily c member 2	10.050	7q11
**ASD-related DEGs in male**				

ID	Symbol	Entrez Gene Name	Expr Log Ratio	Chromosome Location
50510463	PRUNE2	prune homolog 2	-8.443	1q51
672035060	CIC	capicua transcriptional repressor	-8.114	1q21
755566690	HUWE1	HECT, UBA and WWE domain containing 1, E3 ubiquitin protein ligase	-7.864	Xq13
226698394	UNC80	unc-80 homolog, NALCN channel complex subunit	-7.349	9q32
564315185	CUX1	cut like homeobox 1	-7.209	12q12
564303135	KMT2C	lysine methyltransferase 2C	-6.870	4q11
564318843	PSD3	pleckstrin and Sec7 domain containing 3	-6.248	16p14
149065087	CADPS2	calcium dependent secretion activator 2	-5.883	4q22
564329920	EMSY	EMSY, BRCA2 interacting transcriptional repressor	-5.585	1q32
148668227	GPC6	glypican 6	-5.248	15q24
672066092	UNC80	unc-80 homolog, NALCN channel complex subunit	-4.954	9q32
564329926	EMSY	EMSY, BRCA2 interacting transcriptional repressor	-4.655	1q32
392334411	ANKRD11	ankyrin repeat domain 11	-4.358	19q12
54019438	PCDHAC1	protocadherin alpha subfamily C, 1	-3.170	18p11
747019224	SRCAP	Snf2 related CREBBP activator protein	-2.874	1q37
124486586	AUTS2	AUTS2, activator of transcription and developmental regulator	-2.742	12q12
564303143	KMT2C	lysine methyltransferase 2C	-2.497	4q11
755566692	HUWE1	HECT, UBA and WWE domain containing 1, E3 ubiquitin protein ligase	-2.222	Xq13
755566692	HUWE1	HECT, UBA and WWE domain containing 1, E3 ubiquitin protein ligase	-1.410	Xq13
672061705	KMT2A	lysine methyltransferase 2A	-1.326	8q22
293358436	FOXP2	forkhead box P2	-1.322	4q21
564329920	EMSY	EMSY, BRCA2 interacting transcriptional repressor	-1.275	1q32
157787062	GRID2IP	Grid2 interacting protein	-1.260	12p11
672012705	SYNE1	spectrin repeat containing nuclear envelope protein 1	-1.120	1q11

38322763	WNT2	Wnt family member 2	-0.943	4q21
564329916	EMSY	EMSY, BRCA2 interacting transcriptional repressor	-0.864	1q32
46485412	ABCA7	ATP binding cassette subfamily A member 7	-0.798	7q11
672012431	SASH1	SAM and SH3 domain containing 1	-0.756	1p13
157819949	ITGA4	integrin subunit alpha 4	-0.748	3q24
157824208	NTNG1	netrin G1	-0.737	2q41
149062990	CUX1	cut like homeobox 1	-0.682	12q12
12083595	GRM4	glutamate metabotropic receptor 4	-0.653	20p12
149053793	TSPOAP1	TSPO associated protein 1	-0.627	10q26
110347493	PCDHA9	protocadherin alpha 9	-0.587	18p11
672035060	CIC	capicua transcriptional repressor	-0.579	1q21
564313842	SLC38A10	solute carrier family 38 member 10	-0.503	10q32.3
672022667	ARHGAP32	Rho GTPase activating protein 32	-0.502	8q21
62543507	CIB2	calcium and integrin binding family member 2	-0.493	8q24
78126167	SLC1A2	solute carrier family 1 member 2	-0.445	3q32
51854227	GSN	gelsolin	-0.431	3p11
451172073	CHRM3	cholinergic receptor muscarinic 3	-0.399	17q12.1
13591949	GATM	glycine amidinotransferase	-0.365	3q35
449784888	ALDH5A1	aldehyde dehydrogenase 5 family member A1	-0.363	17p11
38322759	MET	MET proto-oncogene, receptor tyrosine kinase	-0.354	4q21
197927216	TBC1D5	TBC1 domain family member 5	-0.351	9q11
755566690	HUWE1	HECT, UBA and WWE domain containing 1, E3 ubiquitin protein ligase	-0.351	Xq13
122065191	ABAT	4-aminobutyrate aminotransferase	-0.348	10q12
6981208	NR3C2	nuclear receptor subfamily 3 group C member 2	-0.328	19q11
564310645	PLXNB1	plexin B1	-0.315	8q32
13994179	SLC24A2	solute carrier family 24 member 2	-0.310	5q32
6981168	LPL	lipoprotein lipase	-0.308	16p14
109480098	SMARCC2	SWI/SNF related, matrix associated, actin dependent regulator of chromatin subfamily c member 2	-0.307	7q11
564318875	PSD3	pleckstrin and Sec7 domain containing 3	-0.286	16p14
28972652	SLC12A5	solute carrier family 12 member 5	-0.285	3q42
50356003	SCP2	sterol carrier protein 2	-0.284	5q34

61556829	RIT2	Ras like without CAAX 2	-0.283	18p12
564394868	CC2D1A	coiled-coil and C2 domain containing 1A	-0.273	19q11
157820049	LRFN5	leucine rich repeat and fibronectin type III domain containing 5	-0.271	6q23
148678721	SOX5	SRY-box 5	-0.265	4q44
115292425	KIRREL3	kirre like nephrin family adhesion molecule 3	-0.263	8q21
747019224	SRCAP	Snf2 related CREBBP activator protein	-0.258	1q37
3043568	IQSEC2	IQ motif and Sec7 domain 2	-0.253	Xq13
157817710	FER	FER tyrosine kinase	-0.251	9q37
300795140	TAF1	TATA-box binding protein associated factor 1	-0.248	Xq22
158749644	MCM6	minichromosome maintenance complex component 6	-0.247	13q13
77627981	SHANK1	SH3 and multiple ankyrin repeat domains 1	-0.233	1q22
19705535	PACS1	phosphofurin acidic cluster sorting protein 1	-0.220	1q43
54312094	DAGLA	diacylglycerol lipase alpha	-0.218	1q43
11560055	KHDRBS3	KH RNA binding domain containing, signal transduction associated 3	-0.217	7q34
157817839	SEMA5A	semaphorin 5A	-0.212	2q22
149023410	SNAP25	synaptosome associated protein 25	-0.209	3q36
149015822	SLC9A6	solute carrier family 9 member A6	-0.209	Xq37
39104626	CAMK2A	calcium/calmodulin dependent protein kinase II alpha	-0.206	18q12.1
672036086	ARHGAP33	Rho GTPase activating protein 33	-0.205	1q21
71896543	SHANK3	SH3 and multiple ankyrin repeat domains 3	-0.204	7q34
8393490	GRM5	glutamate metabotropic receptor 5	-0.203	1q32
59858990	UNC13A	unc-13 homolog A	-0.199	16p14
109472884	UBE3C	ubiquitin protein ligase E3C	-0.198	4q11
13592131	DGKZ	diacylglycerol kinase zeta	-0.197	3q24
672040941	ATRNL1	attractin like 1	-0.196	1q55
56605990	LRPPRC	leucine rich pentatricopeptide repeat containing	-0.194	6q12
38454208	KCTD13	potassium channel tetramerization domain containing 13	-0.193	1q37
148695758	CAPRIN1	cell cycle associated protein 1	-0.188	3q32
8394328	SOD1	superoxide dismutase 1	-0.184	11q11

259155312	AGAP2	ArfGAP with GTPase domain, ankyrin repeat and PH domain 2	-0.184	7q22
83267872	ADNP	activity dependent neuroprotector homeobox	-0.183	3q42
148692349	ATP1A3	ATPase Na+/K+ transporting subunit alpha 3	-0.181	1q21
257796229	SYNGAP1	synaptic Ras GTPase activating protein 1	-0.181	20p12
9665227	DLG4	discs large MAGUK scaffold protein 4	-0.170	10q24
77404395	SND1	staphylococcal nuclease and tudor domain containing 1	-0.165	4q22
148673176	FABP7	fatty acid binding protein 7	-0.158	20q12
37359962	PLPPR4	phospholipid phosphatase related 4	-0.157	2q41
97537309	SYNJ1	synaptojanin 1	-0.152	11q11
564394830	NACC1	nucleus accumbens associated 1	-0.145	19q11
187469796	MBD3	methyl-CpG binding domain protein 3	-0.145	7q11
815891312	CACNA1G	calcium voltage-gated channel subunit alpha1 G	-0.142	10q26
40254595	DPYSL2	dihydropyrimidinase like 2	-0.141	15p12
407728599	CTNND2	catenin delta 2	-0.129	2q22
25453410	CACNA1B	calcium voltage-gated channel subunit alpha1 B	-0.128	3p13
254028210	CMIP	c-Maf inducing protein	-0.125	19q12
253683488	NTRK2	neurotrophic receptor tyrosine kinase 2	-0.124	17p14
68534276	NSMCE3	NSE3 homolog, SMC5-SMC6 complex component	-0.117	1q22
148747541	HNRNPU	heterogeneous nuclear ribonucleoprotein U	-0.109	13q25
149025239	VASH1	vasohibin 1	-0.088	6q31
148747194	SLC16A7	solute carrier family 16 member 7	-0.080	7q22
29789269	GRIA1	glutamate ionotropic receptor AMPA type subunit 1	-0.052	10q22
568931380	CHD5	chromodomain helicase DNA binding protein 5	-0.036	5q36
6978673	CNR1	cannabinoid receptor 1	-0.023	5q21
16758706	NRXN2	neurexin 2	0.005	1q43
83267872	ADNP	activity dependent neuroprotector homeobox	0.123	3q42
564390508	NSD1	nuclear receptor binding SET domain protein 1	0.128	17p14

300795060	SRGAP3	SLIT-ROBO Rho GTPase activating protein 3	0.128	4q42
115312278	MECP2	methyl-CpG binding protein 2	0.135	Xq37
672055431	SPAST	spastin	0.138	6q13
171543899	PLXNA4	plexin A4	0.139	4q22
924859455	NEXMIF	neurite extension and migration factor	0.139	Xq22
157819885	SETD5	SET domain containing 5	0.144	4q42
404312698	CASC4	cancer susceptibility 4	0.150	3q35
728864372	WDFY3	WD repeat and FYVE domain containing 3	0.153	14p22
59709464	PIK3R2	phosphoinositide-3-kinase regulatory subunit 2	0.159	16p14
167555091	CTTNBP2	cortactin binding protein 2	0.159	4q21
300793740	TANC2	tetratricopeptide repeat, ankyrin repeat and coiled-coil containing 2	0.160	10q32.1
10720132	NEO1	neogenin 1	0.164	8q24
24418849	KCNB1	potassium voltage-gated channel subfamily B member 1	0.167	3q42
564327171	ACTN4	actinin alpha 4	0.170	1q21
408387590	TRIP12	thyroid hormone receptor interactor 12	0.176	9q35
6978787	DYRK1A	dual specificity tyrosine phosphorylation regulated kinase 1A	0.179	11q11
148698133	AHDC1	AT-hook DNA binding motif containing 1	0.180	5q36
157821015	KDM5B	lysine demethylase 5B	0.182	13q13
149026331	SRSF11	serine and arginine rich splicing factor 11	0.185	2q45
42627759	SMC3	structural maintenance of chromosomes 3	0.186	1q55
404351649	CDK13	cyclin dependent kinase 13	0.191	17q11
157820255	MED13	mediator complex subunit 13	0.196	10q26
119616373	MEF2C	myocyte enhancer factor 2C	0.200	2q11
291042494	MED13L	mediator complex subunit 13 like	0.202	12q16
672042964	ASH1L	ASH1 like histone lysine methyltransferase	0.203	2q34
29789299	XPO1	exportin 1	0.204	14q22
425384	CAMK4	calcium/calmodulin dependent protein kinase IV	0.207	18p12
6981264	NF1	neurofibromin 1	0.213	10q25
19705483	CLSTN2	calsyntenin 2	0.214	8q31
62088168	ELAVL2	ELAV like RNA binding protein 2	0.216	5q32

564386027	CHD8	chromodomain helicase DNA binding protein 8	0.219	15p14
2804296	CDH8	cadherin 8	0.221	19p13
928135679	GAN	gigaxonin	0.222	19q12
564361244	TCF20	transcription factor 20	0.222	7q34
148491097	DYNC1H1	dynein cytoplasmic 1 heavy chain 1	0.225	6q32
13540699	NRP2	neuropilin 2	0.226	9q32
157819605	EPC2	enhancer of polycomb homolog 2	0.227	3q12
392348740	LAMB1	laminin subunit beta 1	0.228	6q16
403420604	PCDH11X	protocadherin 11 X-linked	0.228	Xq31
11177894	TSC1	TSC complex subunit 1	0.230	3p12
17864836	CACNA1C	calcium voltage-gated channel subunit alpha1 C	0.230	4q42
157817961	PHF3	PHD finger protein 3	0.230	9q21
145553966	CACNA1E	calcium voltage-gated channel subunit alpha1 E	0.231	13q21
213972547	KAT6A	lysine acetyltransferase 6A	0.236	16q12.5
2266994	OGT	O-linked N-acetylglucosamine (GlcNAc) transferase	0.238	Xq22
14091754	GRIP1	glutamate receptor interacting protein 1	0.239	7q22
403310691	RAI1	retinoic acid induced 1	0.242	10q22
300797458	UBE3A	ubiquitin protein ligase E3A	0.243	1q22
148697254	TBL1X	transducin beta like 1 X-linked	0.243	Xq21
672022994	NEO1	neogenin 1	0.248	8q24
290560930	CREBBP	CREB binding protein	0.255	10q12
157818967	KDM6B	lysine demethylase 6B	0.258	10q24
403310664	KMT2E	lysine methyltransferase 2E	0.263	4q11
157820313	GIGYF1	GRB10 interacting GYF protein 1	0.265	12q12
9506755	GRIK2	glutamate ionotropic receptor kainate type subunit 2	0.274	20q13
11067415	ERBB4	erb-b2 receptor tyrosine kinase 4	0.283	9q32-q33
149060466	ZBTB20	zinc finger and BTB domain containing 20	0.293	11q21
13929168	FAT1	FAT atypical cadherin 1	0.305	16q11
157818041	YEATS2	YEATS domain containing 2	0.309	11q23
672035060	CIC	capicua transcriptional repressor	0.325	1q21
149050659	BIRC6	baculoviral IAP repeat containing 6	0.331	6q13
564303143	KMT2C	lysine methyltransferase 2C	0.336	4q11
29612542	H2AFZ	H2A histone family member Z	0.337	2q44
51871603	ST7	suppression of tumorigenicity 7	0.339	4q21
198041672	TNRC6B	trinucleotide repeat containing 6B	0.345	7q34

149027091	GABRA3	gamma-aminobutyric acid type A receptor alpha3 subunit	0.357	Xq37
109467304	TRIM33	tripartite motif containing 33	0.363	2q34
564310645	PLXNB1	plexin B1	0.364	8q32
672031398	ANKRD11	ankyrin repeat domain 11	0.367	19q12
564328896	CHD2	chromodomain helicase DNA binding protein 2	0.370	1q31
672061705	KMT2A	lysine methyltransferase 2A	0.372	8q22
157817845	KMT5B	lysine methyltransferase 5B	0.374	1q43
564333605	BTAF1	B-TFIID TATA-box binding protein associated factor 1	0.392	1q53
149016466	NIPBL	NIPBL, cohesin loading factor	0.407	2q16
40789237	PCDHA4	protocadherin alpha 4	0.419	18p11
392353586	INTS6	integrator complex subunit 6	0.427	15p12
568940286	BRAF	B-Raf proto-oncogene, serine/threonine kinase	0.444	4q22
148705863	GABRA4	gamma-aminobutyric acid type A receptor alpha4 subunit	0.450	14p11
31543579	RELN	reelin	0.461	4q11
672087275	KDM5C	lysine demethylase 5C	0.558	Xq13
62078501	TTI2	TELO2 interacting protein 2	0.561	16q12.3
209364558	CEP290	centrosomal protein 290	0.563	7q13
672066092	UNC80	unc-80 homolog, NALCN channel complex subunit	0.589	9q32
13928942	PER2	period circadian regulator 2	0.600	9q36
28972363	DOCK4	dedicator of cytokinesis 4	0.647	6q21
564315183	CUX1	cut like homeobox 1	0.748	12q12
157818841	POGZ	pogo transposable element derived with ZNF domain	0.842	2q34
392332010	SLC38A10	solute carrier family 38 member 10	0.955	10q32.3
564303143	KMT2C	lysine methyltransferase 2C	0.997	4q11
672061705	KMT2A	lysine methyltransferase 2A	1.003	8q22
747019224	SRCAP	Snf2 related CREBBP activator protein	1.107	1q37
149058952	MEF2C	myocyte enhancer factor 2C	1.126	2q11
755566692	HUWE1	HECT, UBA and WWE domain containing 1, E3 ubiquitin protein ligase	1.130	Xq13
157822425	MFRP	membrane frizzled-related protein	1.264	8q22
293348214	CCDC88C	coiled-coil domain containing 88C	1.293	6q32

755566690	HUWE1	HECT, UBA and WWE domain containing 1, E3 ubiquitin protein ligase	1.299	Xq13
149016805	PSD3	pleckstrin and Sec7 domain containing 3	1.318	16p14
672087275	KDM5C	lysine demethylase 5C	1.411	Xq13
568972665	TSPOAP1	TSPO associated protein 1	1.480	10q26
568990288	NIPBL	NIPBL, cohesin loading factor	1.663	2q16
755566690	HUWE1	HECT, UBA and WWE domain containing 1, E3 ubiquitin protein ligase	1.675	Xq13
564329926	EMSY	EMSY, BRCA2 interacting transcriptional repressor	1.703	1q32
50510463	PRUNE2	prune homolog 2	2.470	1q51
564303135	KMT2C	lysine methyltransferase 2C	2.505	4q11
564329920	EMSY	EMSY, BRCA2 interacting transcriptional repressor	2.899	1q32
672048712	KMT2C	lysine methyltransferase 2C	3.209	4q11
568906591	UNC80	unc-80 homolog, NALCN channel complex subunit	4.755	9q32
564303143	KMT2C	lysine methyltransferase 2C	5.322	4q11
564329918	EMSY	EMSY, BRCA2 interacting transcriptional repressor	5.476	1q32
564315188	CUX1	cut like homeobox 1	6.267	12q12
564313845	SLC38A10	solute carrier family 38 member 10	6.807	10q32.3
755566690	HUWE1	HECT, UBA and WWE domain containing 1, E3 ubiquitin protein ligase	7.555	Xq13
564329920	EMSY	EMSY, BRCA2 interacting transcriptional repressor	7.728	1q32
109472884	UBE3C	ubiquitin protein ligase E3C	8.238	4q11
755566692	HUWE1	HECT, UBA and WWE domain containing 1, E3 ubiquitin protein ligase	8.326	Xq13
109480102	SMARCC2	SWI/SNF related, matrix associated, actin dependent regulator of chromatin subfamily c member 2	10.050	7q11
**ASD-related DEGs in female**				
**ID**	**Symbol**	**Entrez Gene Name**	**Expr Log Ratio**	**Chromosome Location**
755566690	HUWE1	HECT, UBA and WWE domain containing 1, E3 ubiquitin protein ligase	-7.570	Xq13

28972363	DOCK4	dedicator of cytokinesis 4	-7.468	6q21
755566692	HUWE1	HECT, UBA and WWE domain containing 1, E3 ubiquitin protein ligase	-7.238	Xq13
672065933	DOCK10	dedicator of cytokinesis 10	-5.907	9q34
392332008	SLC38A10	solute carrier family 38 member 10	-5.672	10q32.3
564303143	KMT2C	lysine methyltransferase 2C	-5.044	4q11
672035060	CIC	capicua transcriptional repressor	-3.861	1q21
564315188	CUX1	cut like homeobox 1	-3.459	12q12
663434101	CUX1	cut like homeobox 1	-3.222	12q12
274321177	MCM4	minichromosome maintenance complex component 4	-2.844	11q23
148689488	SYN3	synapsin III	-2.438	7q13
25742828	SCN7A	sodium voltage-gated channel alpha subunit 7	-2.202	3q21
158186711	F13A1	coagulation factor XIII A chain	-1.886	17p12
55741827	TERT	telomerase reverse transcriptase	-1.801	1p11
149053793	TSPOAP1	TSPO associated protein 1	-1.659	10q26
109480098	SMARCC2	SWI/SNF related, matrix associated, actin dependent regulator of chromatin subfamily c member 2	-1.490	7q11
768033504	KDM6A	lysine demethylase 6A	-1.442	Xq11
564325156	SYNE1	spectrin repeat containing nuclear envelope protein 1	-1.323	1q11
25742776	MC4R	melanocortin 4 receptor	-1.309	18q12.1
672066092	UNC80	unc-80 homolog, NALCN activator	-1.273	9q32
13591940	DPYD	dihydropyrimidine dehydrogenase	-1.272	2q41
672061705	KMT2A	lysine methyltransferase 2A	-1.236	8q22
6978773	DPP4	dipeptidyl peptidase 4	-1.161	3q21
392332010	SLC38A10	solute carrier family 38 member 10	-1.136	10q32.3
564389875	ARHGEF10	Rho guanine nucleotide exchange factor 10	-1.098	16q12.5
568977750	DOCK4	dedicator of cytokinesis 4	-0.983	6q21
18959222	SCN9A	sodium voltage-gated channel alpha subunit 9	-0.968	3q21
149053793	TSPOAP1	TSPO associated protein 1	-0.963	10q26
19424190	CX3CR1	C-X3-C motif chemokine receptor 1	-0.945	8q32
60360628	ATP8A1	ATPase phospholipid transporting 8A1	-0.941	14p11
157822365	LAMC3	laminin subunit gamma 3	-0.930	3p12
198442871	KANK1	KN motif and ankyrin repeat domains 1	-0.897	1q51
672012705	SYNE1	spectrin repeat containing nuclear envelope protein 1	-0.859	1q11

568940286	BRAF	B-Raf proto-oncogene, serine/threonine kinase	-0.852	4q22
406719604	SLC9A9	solute carrier family 9 member A9	-0.851	8q31
755566692	HUWE1	HECT, UBA and WWE domain containing 1, E3 ubiquitin protein ligase	-0.837	Xq13
11177892	KCNT1	potassium sodium-activated channel subfamily T member 1	-0.833	3p13
672073969	CLASP1	cytoplasmic linker associated protein 1	-0.817	13q11
226698394	UNC80	unc-80 homolog, NALCN activator	-0.814	9q32
755566682	HUWE1	HECT, UBA and WWE domain containing 1, E3 ubiquitin protein ligase	-0.788	Xq13
67514566	POLA2	DNA polymerase alpha 2, accessory subunit	-0.780	1q43
19173756	ERG	ERG, ETS transcription factor	-0.761	11q11
672041704	NIPBL	NIPBL, cohesin loading factor	-0.760	2q16
108935976	DISC1	disrupted in schizophrenia 1	-0.754	19q12
392332443	PRKDC	protein kinase, DNA-activated, catalytic polypeptide	-0.697	11q23
149053793	TSPOAP1	TSPO associated protein 1	-0.661	10q26
157823279	CGNL1	cingulin like 1	-0.645	8q24
564330609	SYT17	synaptotagmin 17	-0.639	1q35
392341425	PTPRB	protein tyrosine phosphatase, receptor type B	-0.637	7q22
18959236	PECR	peroxisomal trans-2-enoyl-CoA reductase	-0.629	9q33
77695926	STAT1	signal transducer and activator of transcription 1	-0.617	9q22
312147379	LAMA1	laminin subunit alpha 1	-0.610	9q38
568956384	ADAMTS18	ADAM metallopeptidase with thrombospondin type 1 motif 18	-0.604	19q12
25453410	CACNA1B	calcium voltage-gated channel subunit alpha1 B	-0.602	3p13
50510463	PRUNE2	prune homolog 2	-0.599	1q51
148747194	SLC16A7	solute carrier family 16 member 7	-0.597	7q22
13562118	LRP2	LDL receptor related protein 2	-0.589	3q21
348041395	DLGAP2	DLG associated protein 2	-0.582	16q12.5
815891312	CACNA1G	calcium voltage-gated channel subunit alpha1 G	-0.561	10q26
16758138	POMT1	protein O-mannosyltransferase 1	-0.560	3p12
149038513	SYNE1	spectrin repeat containing nuclear envelope protein 1	-0.555	1q11

16758716	CACNB2	calcium voltage-gated channel auxiliary subunit beta 2	-0.550	17q12.3
149060466	ZBTB20	zinc finger and BTB domain containing 20	-0.549	11q21
10242377	GRIK4	glutamate ionotropic receptor kainate type subunit 4	-0.546	8q22
6978867	GABRB1	gamma-aminobutyric acid type A receptor beta1 subunit	-0.536	14p11
157819687	TUBGCP5	tubulin gamma complex associated protein 5	-0.534	1q22
110347559	PCDHA13	protocadherin alpha 13	-0.531	18p11
157824208	NTNG1	netrin G1	-0.528	2q41
13994179	SLC24A2	solute carrier family 24 member 2	-0.524	5q32
219804406	DOCK1	dedicator of cytokinesis 1	-0.519	1q41
28972652	SLC12A5	solute carrier family 12 member 5	-0.519	3q42
149016805	PSD3	pleckstrin and Sec7 domain containing 3	-0.515	16p14
18959266	KHDRBS2	KH RNA binding domain containing, signal transduction associated 2	-0.513	9q21
55741540	KATNAL1	katanin catalytic subunit A1 like 1	-0.505	12p11
392343941	ZC3H4	zinc finger CCCH-type containing 4	-0.502	1q21
672035060	CIC	capicua transcriptional repressor	-0.499	1q21
403420604	PCDH11X	protocadherin 11 X-linked	-0.494	Xq31
13027400	GUCY1A2	guanylate cyclase 1 soluble subunit alpha 2	-0.493	8q11
157817205	USP45	ubiquitin specific peptidase 45	-0.492	5q21
6978789	SPARCL1	SPARC like 1	-0.491	14p22
13786142	SLIT3	slit guidance ligand 3	-0.477	10q12
158508684	BCAS1	breast carcinoma amplified sequence 1	-0.474	3q42
51854227	GSN	gelsolin	-0.457	3p11
61557206	ZBTB16	zinc finger and BTB domain containing 16	-0.457	8q23
122065191	ABAT	4-aminobutyrate aminotransferase	-0.455	10q12
140971205	GRIN2A	glutamate ionotropic receptor NMDA type subunit 2A	-0.452	10q11
392342157	PHIP	pleckstrin homology domain interacting protein	-0.452	8q31
13591949	GATM	glycine amidinotransferase	-0.451	3q35
201066395	MBOAT7	membrane bound O-acyltransferase domain containing 7	-0.442	1q12
16758746	CNTN4	contactin 4	-0.442	4q41
157821397	SLC22A15	solute carrier family 22 member 15	-0.432	2q34

300795140	TAF1	TATA-box binding protein associated factor 1	-0.426	Xq22
47155563	KIF13B	kinesin family member 13B	-0.423	15p12
11067415	ERBB4	erb-b2 receptor tyrosine kinase 4	-0.420	9q32-q33
672012705	SYNE1	spectrin repeat containing nuclear envelope protein 1	-0.419	1q11
672043401	POGZ	pogo transposable element derived with ZNF domain	-0.418	2q34
755566690	HUWE1	HECT, UBA and WWE domain containing 1, E3 ubiquitin protein ligase	-0.414	Xq13
166999225	GRM1	glutamate metabotropic receptor 1	-0.411	1p13
149066531	VPS13B	vacuolar protein sorting 13 homolog B	-0.406	7q22
12055542	SLC25A27	solute carrier family 25 member 27	-0.400	9q13
13540699	NRP2	neuropilin 2	-0.399	9q32
149039557	UTRN	utrophin	-0.387	1p13
672087275	KDM5C	lysine demethylase 5C	-0.386	Xq13
145553966	CACNA1E	calcium voltage-gated channel subunit alpha1 E	-0.382	13q21
149039905	TSPAN17	tetraspanin 17	-0.371	17p14
6978673	CNR1	cannabinoid receptor 1	-0.370	5q21
78126167	SLC1A2	solute carrier family 1 member 2	-0.366	3q32
149020512	PDE4A	phosphodiesterase 4A	-0.366	8q13
82617648	CSMD1	CUB and Sushi multiple domains 1	-0.363	16q12.5
568977996	PXDN	peroxidasin	-0.362	6q16
392339847	CADPS2	calcium dependent secretion activator 2	-0.361	4q22
148667088	ATP2B2	ATPase plasma membrane Ca2+ transporting 2	-0.357	4q42
115292425	KIRREL3	kin of IRRE like 3 (Drosophila)	-0.350	8q21
54019432	PCDHA7	protocadherin alpha 7	-0.345	18p11
690969206	MBD6	methyl-CpG binding domain protein 6	-0.343	7q22
281306814	RPS6KA2	ribosomal protein S6 kinase A2	-0.341	1q12
392348740	LAMB1	laminin subunit beta 1	-0.325	6q16
157817476	HECW2	HECT, C2 and WW domain containing E3 ubiquitin protein ligase 2	-0.325	9q31
8393490	GRM5	glutamate metabotropic receptor 5	-0.324	1q32
564394868	CC2D1A	coiled-coil and C2 domain containing 1A	-0.324	19q11
194473622	ADSL	adenylosuccinate lyase	-0.323	7q34
97537309	SYNJ1	synaptojanin 1	-0.321	11q11

50511316	AHI1	Abelson helper integration site 1	-0.316	1p12
67078434	SLC25A39	solute carrier family 25 member 39	-0.314	10q32.1
157818787	BBS4	Bardet-Biedl syndrome 4	-0.307	8q24
451172073	CHRM3	cholinergic receptor muscarinic 3	-0.304	17q12.1
149049163	GRIN2B	glutamate ionotropic receptor NMDA type subunit 2B	-0.304	4q43
28972363	DOCK4	dedicator of cytokinesis 4	-0.304	6q21
564387864	CACNA1D	calcium voltage-gated channel subunit alpha1 D	-0.299	16p16
59858990	UNC13A	unc-13 homolog A	-0.299	16p14
270288740	MAOA	monoamine oxidase A	-0.290	Xq11
124486885	LRRC7	leucine rich repeat containing 7	-0.283	2q45
148689929	CYFIP1	cytoplasmic FMR1 interacting protein 1	-0.281	1q22
672088848	PLXNA3	plexin A3	-0.277	Xq37
54312094	DAGLA	diacylglycerol lipase alpha	-0.276	1q43
672022994	NEO1	neogenin 1	-0.275	8q24
145312274	EPHA6	EPH receptor A6	-0.273	11q12
469469055	KCNQ3	potassium voltage-gated channel subfamily Q member 3	-0.273	7q34
148695071	STK39	serine/threonine kinase 39	-0.271	3q21
12018268	ADCY5	adenylate cyclase 5	-0.270	11q22
157818225	CNTNAP4	contactin associated protein like 4	-0.262	19q12
37359962	PLPPR4	phospholipid phosphatase related 4	-0.257	2q41
564388440	MYO9B	myosin IXB	-0.254	16p14
148695758	CAPRIN1	cell cycle associated protein 1	-0.252	3q32
157817839	SEMA5A	semaphorin 5A	-0.247	2q22
672040941	ATRNL1	attractin like 1	-0.246	1q55
6981208	NR3C2	nuclear receptor subfamily 3 group C member 2	-0.245	19q11
564327171	ACTN4	actinin alpha 4	-0.243	1q21
33413429	NTRK3	neurotrophic receptor tyrosine kinase 3	-0.243	1q31
294845709	OCRL	OCRL, inositol polyphosphate-5-phosphatase	-0.237	Xq35
77404395	SND1	staphylococcal nuclease and tudor domain containing 1	-0.235	4q22
26006243	KCND2	potassium voltage-gated channel subfamily D member 2	-0.230	4q22
717324516	SCN8A	sodium voltage-gated channel alpha subunit 8	-0.226	7q36
281306738	PCDH19	protocadherin 19	-0.220	Xq32
11559947	CASK	calcium/calmodulin dependent serine protein kinase	-0.215	Xq12

47846864	CADM1	cell adhesion molecule 1	-0.213	8q23
755533955	ANK3	ankyrin 3	-0.211	20p11
56090379	POMGNT1	protein O-linked mannose N-acetylglucosaminyltransferase 1 (beta 1,2-)	-0.209	5q35
149050844	DNMT3A	DNA methyltransferase 3 alpha	-0.208	6q14
39104626	CAMK2A	calcium/calmodulin dependent protein kinase II alpha	-0.206	18q12.1
148681975	NXPH1	neurexophilin 1	-0.205	4q21
747019224	SRCAP	Snf2 related CREBBP activator protein	-0.201	1q37
157817710	FER	FER tyrosine kinase	-0.200	9q37
19705535	PACS1	phosphofurin acidic cluster sorting protein 1	-0.194	1q43
564328626	HERC2	HECT and RLD domain containing E3 ubiquitin protein ligase 2	-0.193	1q22
564310645	PLXNB1	plexin B1	-0.192	8q32
568981376	DIP2C	disco interacting protein 2 homolog C	-0.191	17q12.1
71896543	SHANK3	SH3 and multiple ankyrin repeat domains 3	-0.188	7q34
288806592	PRKCB	protein kinase C beta	-0.187	1q36
115311606	MAPK3	mitogen-activated protein kinase 3	-0.185	1q36
37360128	CNTNAP2	contactin associated protein-like 2	-0.183	4q24
157818589	TSPAN7	tetraspanin 7	-0.180	Xq12
13928946	SMC1A	structural maintenance of chromosomes 1A	-0.179	Xq13
29789269	GRIA1	glutamate ionotropic receptor AMPA type subunit 1	-0.178	10q22
157823569	EXOC6B	exocyst complex component 6B	-0.178	4q34
149069395	CUL7	cullin 7	-0.178	9q12
198386334	KDM4B	lysine demethylase 4B	-0.178	9q12
11560079	KIT	KIT proto-oncogene receptor tyrosine kinase	-0.176	14p11
11560055	KHDRBS3	KH RNA binding domain containing, signal transduction associated 3	-0.174	7q34
14091754	GRIP1	glutamate receptor interacting protein 1	-0.170	7q22
17864836	CACNA1C	calcium voltage-gated channel subunit alpha1 C	-0.168	4q42
403224975	SBF1	SET binding factor 1	-0.168	7q34
149052114	CACNA1H	calcium voltage-gated channel subunit alpha1 H	-0.167	10q12

13592131	DGKZ	diacylglycerol kinase zeta	-0.167	3q24
114145542	ARHGAP5	Rho GTPase activating protein 5	-0.157	6q23
9507159	SYN1	synapsin I	-0.156	Xq11
148747541	HNRNPU	heterogeneous nuclear ribonucleoprotein U	-0.150	13q25
208973288	PREX1	phosphatidylinositol-3,4,5-trisphosphate dependent Rac exchange factor 1	-0.149	3q42
148682872	MTOR	mechanistic target of rapamycin	-0.148	5q36
148700348	ZMYND11	zinc finger MYND-type containing 11	-0.144	17q12.1
149017161	APC	APC, WNT signaling pathway regulator	-0.134	18p12
259155312	AGAP2	ArfGAP with GTPase domain, ankyrin repeat and PH domain 2	-0.134	7q22
224967068	PLCB1	phospholipase C beta 1	-0.127	3q36
149050659	BIRC6	baculoviral IAP repeat containing 6	-0.125	6q13
253683488	NTRK2	neurotrophic receptor tyrosine kinase 2	-0.118	17p14
149023410	SNAP25	synaptosome associated protein 25	-0.118	3q36
13929102	APBA2	amyloid beta precursor protein binding family A member 2	-0.116	1q22
77627981	SHANK1	SH3 and multiple ankyrin repeat domains 1	-0.107	1q22
149020473	SMARCA4	SWI/SNF related, matrix associated, actin dependent regulator of chromatin, subfamily a, member 4	-0.101	8q13
148694359	MYO5A	myosin VA	-0.086	8q24
38372401	NRCAM	neuronal cell adhesion molecule	-0.080	6q21
149022387	NCKAP1	NCK associated protein 1	-0.073	3q24
148692349	ATP1A3	ATPase Na+/K+ transporting subunit alpha 3	-0.064	1q21
148673176	FABP7	fatty acid binding protein 7	-0.057	20q12
40254595	DPYSL2	dihydropyrimidinase like 2	-0.051	15p12
2266994	OGT	O-linked N-acetylglucosamine (GlcNAc) transferase	-0.008	Xq22
7305363	PAFAH1B1	platelet activating factor acetylhydrolase 1b regulatory subunit 1	0.060	10q24
407728599	CTNND2	catenin delta 2	0.066	2q22
17978461	BRINP1	BMP/retinoic acid inducible neural specific 1	0.087	5q24
25742568	DPYSL3	dihydropyrimidinase like 3	0.101	18p11-q11
148672704	CSNK1E	casein kinase 1 epsilon	0.109	7q34

13929006	RAB2A	RAB2A, member RAS oncogene family	0.118	5q13
157818315	CUL3	cullin 3	0.139	9q34
564328896	CHD2	chromodomain helicase DNA binding protein 2	0.145	1q31
18959272	KCNQ2	potassium voltage-gated channel subfamily Q member 2	0.146	3q43
157820255	MED13	mediator complex subunit 13	0.147	10q26
672075032	MARK1	microtubule affinity regulating kinase 1	0.152	13q26
393716310	WAC	WW domain containing adaptor with coiled-coil	0.154	17q12.1
148688660	FEZF2	FEZ family zinc finger 2	0.155	15p16
157819885	SETD5	SET domain containing 5	0.155	4q42
300793740	TANC2	tetratricopeptide repeat, ankyrin repeat and coiled-coil containing 2	0.156	10q32.1
13928824	YWHAE	tyrosine 3-monooxygenase/tryptophan 5-monooxygenase activation protein epsilon	0.165	10q24
209447111	PDZD4	PDZ domain containing 4	0.166	Xq37
568921780	EIF4E	eukaryotic translation initiation factor 4E	0.167	2q44
171543899	PLXNA4	plexin A4	0.173	4q22
672050411	RAB11FIP5	RAB11 family interacting protein 5	0.173	4q34
22024394	FABP5	fatty acid binding protein 5	0.177	2q23
86477155	PER1	period circadian clock 1	0.185	10q24
8393415	GAP43	growth associated protein 43	0.191	11q21
672042487	NBEA	neurobeachin	0.196	2q26
10720132	NEO1	neogenin 1	0.198	8q24
197927410	SETBP1	SET binding protein 1	0.205	18q12.3
33356154	UBE2H	ubiquitin conjugating enzyme E2 H	0.210	4q22
564393951	MBD1	methyl-CpG binding domain protein 1	0.217	18q12.2
8394405	SLC7A5	solute carrier family 7 member 5	0.220	19q12
59709464	PIK3R2	phosphoinositide-3-kinase regulatory subunit 2	0.221	16p14
2804296	CDH8	cadherin 8	0.221	19p13
83267872	ADNP	activity dependent neuroprotector homeobox	0.227	3q42
42627759	SMC3	structural maintenance of chromosomes 3	0.230	1q55
11559976	EXOC5	exocyst complex component 5	0.234	15p14
27229298	ELAVL3	ELAV like RNA binding protein 3	0.238	8q13

157787066	SLC29A4	solute carrier family 29 member 4	0.239	12p11
29612542	H2AFZ	H2A histone family member Z	0.241	2q44
443906726	DLGAP3	DLG associated protein 3	0.245	5q36
145558904	EML1	echinoderm microtubule associated protein like 1	0.254	6q32
149038734	EIF4EBP2	eukaryotic translation initiation factor 4E binding protein 2	0.260	20q11
201066397	MPP6	membrane palmitoylated protein 6	0.264	4q24
124487247	PRICKLE2	prickle planar cell polarity protein 2	0.264	4q34
281332151	ROBO2	roundabout guidance receptor 2	0.266	11p11
61556927	EIF3G	eukaryotic translation initiation factor 3 subunit G	0.269	8q13
6978787	DYRK1A	dual specificity tyrosine phosphorylation regulated kinase 1A	0.273	11q11
157817961	PHF3	PHD finger protein 3	0.277	9q21
13929168	FAT1	FAT atypical cadherin 1	0.296	16q11
23097350	TOMM20	translocase of outer mitochondrial membrane 20	0.299	19q12
425384	CAMK4	calcium/calmodulin dependent protein kinase IV	0.313	18p12
564315672	CLASP1	cytoplasmic linker associated protein 1	0.336	13q11
672026667	CUX1	cut like homeobox 1	0.338	12q12
157818841	POGZ	pogo transposable element derived with ZNF domain	0.346	2q34
119616373	MEF2C	myocyte enhancer factor 2C	0.351	2q11
24025618	DAB1	DAB1, reelin adaptor protein	0.352	5q33-q34
40789237	PCDHA4	protocadherin alpha 4	0.377	18p11
564300780	TRIM33	tripartite motif containing 33	0.378	2q34
404312698	CASC4	cancer susceptibility 4	0.390	3q35
157819717	NIPA2	non imprinted in Prader-Willi/Angelman syndrome 2	0.429	1q22
149058952	MEF2C	myocyte enhancer factor 2C	0.441	2q11
157823369	TERF2	telomeric repeat binding factor 2	0.442	19q12
148687364	Cux1	cut-like homeobox 1	0.443	12q12
157817845	KMT5B	lysine methyltransferase 5B	0.450	1q43
40789241	PCDHA3	protocadherin alpha 3	0.450	18p11
564335688	TRIO	trio Rho guanine nucleotide exchange factor	0.485	2q22
157824124	NUAK1	NUAK family kinase 1	0.494	7q13
13928942	PER2	period circadian clock 2	0.513	9q36
34328151	TBR1	T-box, brain 1	0.518	3q21
157820017	FBXO33	F-box protein 33	0.542	6q23
16758574	CNTN5	contactin 5	0.636	8q11

11560065	GPR85	G protein-coupled receptor 85	0.656	4q21
124486586	AUTS2	AUTS2, activator of transcription and developmental regulator	0.660	12q12
293348214	CCDC88C	coiled-coil domain containing 88C	0.665	6q32
755566692	HUWE1	HECT, UBA and WWE domain containing 1, E3 ubiquitin protein ligase	0.776	Xq13
564329918	EMSY	EMSY, BRCA2 interacting transcriptional repressor	0.785	1q32
564322442	Kdm6a	lysine demethylase 6A	0.842	Xq11
564315667	CLASP1	cytoplasmic linker associated protein 1	0.851	13q11
741943159	OTX1	orthodenticle homeobox 1	0.947	14q22
109472884	UBE3C	ubiquitin protein ligase E3C	0.950	4q11
6679423	POU3F2	POU class 3 homeobox 2	0.956	5q21
564329920	EMSY	EMSY, BRCA2 interacting transcriptional repressor	0.991	1q32
109480102	SMARCC2	SWI/SNF related, matrix associated, actin dependent regulator of chromatin subfamily c member 2	1.042	7q11
564327667	TSHZ3	teashirt zinc finger homeobox 3	1.080	1q21
148689488	SYN3	synapsin III	1.225	7q13
755566692	HUWE1	HECT, UBA and WWE domain containing 1, E3 ubiquitin protein ligase	1.288	Xq13
672031995	Kdm6a	lysine demethylase 6A	1.326	Xq11
293358436	FOXP2	forkhead box P2	1.342	4q21
564313842	SLC38A10	solute carrier family 38 member 10	1.585	10q32.3
672012705	SYNE1	spectrin repeat containing nuclear envelope protein 1	1.617	1q11
568990288	NIPBL	NIPBL, cohesin loading factor	1.747	2q16
755515866	BRAF	B-Raf proto-oncogene, serine/threonine kinase	1.876	4q22
564326713	ZC3H4	zinc finger CCCH-type containing 4	1.881	1q21
24308466	ITGB3	integrin subunit beta 3	2.303	10q32.1
672061705	KMT2A	lysine methyltransferase 2A	2.760	8q22
564329920	EMSY	EMSY, BRCA2 interacting transcriptional repressor	4.585	1q32
149066939	PTPRB	protein tyrosine phosphatase, receptor type B	5.248	7q22
755566692	HUWE1	HECT, UBA and WWE domain containing 1, E3 ubiquitin protein ligase	5.728	Xq13

564329926	EMSY	EMSY, BRCA2 interacting transcriptional repressor	5.807	1q32
392342157	PHIP	pleckstrin homology domain interacting protein	6.629	8q31
755566690	HUWE1	HECT, UBA and WWE domain containing 1, E3 ubiquitin protein ligase	6.700	Xq13
575403049	ERBIN	erbb2 interacting protein	7.622	2q12
60360628	ATP8A1	ATPase phospholipid transporting 8A1	7.877	14p11
109472884	UBE3C	ubiquitin protein ligase E3C	8.155	4q11
672035060	CIC	capicua transcriptional repressor	10.209	1q21

Supplementary Table S3. Top biological functions and canonical pathways associated with dysregulated genes from RNA-seq identified by Ingenuity Pathway Analysis (IPA). Biological pathways and canonical pathways predicted by IPA were significantly associated with dysregulated genes in both sexes and in males and females separately. P-values were calculated using Fisher's exact test ($P < 0.05$). The numbers of genes involved in each biological function and pathway are shown.

Both Sexes	Males	Females
**Biological functions and pathways**		
**Physiological system development and function (P-values; number of genes)**		
Connective Tissue Development and Function (3.37E-04 - 3.37E-04; 8)	Nervous System Development and Function (6.56E-03 - 1.12E-03; 37)	Cardiovascular System Development and Function (9.84E-05 - 1.06E-08; 153)
Skeletal and Muscular System Development and Function (3.37E-04 - 3.37E-04; 8)	Organismal Development (8.40E-03 - 1.12E-03; 50)	Organismal Development (1.56E-05 - 1.06E-08; 126)
Tissue Development (5.82E-03 - 3.37E-04; 16)	Tissue Development (7.67E-03 - 1.12E-03; 71)	Tissue Development (1.61E-04 - 2.31E-07; 116)
Digestive System Development and Function (7.30E-03 - 6.71E-04; 32)	Connective Tissue Development and Function (5.82E-03 - 1.84E-03; 36)	Organismal Survival (3.95E-06 - 2.50E-06; 108)
Reproductive System Development and Function (5.85E-03 - 1.42E-03; 19)	Inflammatory Response (5.83E-03-5.83E-03; 4)	Nervous System Development and Function (1.86E-05 - 1.86E-05; 11)
**Top canonical pathways (P-values; number of genes)**		
Cholesterol Biosynthesis I (2.46E-04; 9)	Glutamate Receptor Signaling (5.86E-05; 14)	Axonal Guidance Signaling (1.92E-07; 97)
Cholesterol Biosynthesis II (via 24,25-dihydrolanosterol) (2.46E-04; 9)	Axonal Guidance Signaling (1.39E-03; 52)	Neuropathic Pain Signaling in Dorsal Horn Neurons (6.89E-07; 35)
Cholesterol Biosynthesis III (via Desmosterol) (2.46E-04; 9)	Amyotrophic Lateral Sclerosis Signaling (1.63E-03; 18)	Nitric Oxide Signaling in the Cardiovascular System (1.60E-06; 33)
Superpathway of Cholesterol Biosynthesis (3.89E-04; 14)	Circadian Rhythm Signaling (2.79E-03; 8)	Glutamate Receptor Signaling (1.39E-05; 20)
Ketogenesis (1.18E-03; 7)	Huntington's Disease Signaling (9.30E-03; 29)	Circadian Rhythm Signaling (6.31E-04; 12)

Supplementary Table S4. Comparison analysis using sets of DEGs in males and females showing sex-specific effects among neurological pathways. A comparison analysis between males and females was performed by IPA software using the lists of DEGs for each sex. The canonical pathways related to ASD that were shown to be significantly affected in males were not significantly affected in females, and vice versa. Statistical significance was determined using Fisher's exact test and is shown as P-values.

**Ingenuity canonical pathways**	**Males**	**Females**
DNA Methylation and Transcriptional Repression Signaling	**0.015**	0.264
4-Aminobutyrate Degradation I	**0.017**	0.344
Docosahexaenoic Acid (DHA) Signaling	**0.043**	0.072
Amyloid Processing	NS	**3.46E-05**
IGF-1 Signaling	0.060	**7.24E-05**
Synaptic Long-Term Potentiation	0.116	**2.34E-04**
CREB Signaling in Neurons	0.066	**3.72E-04**
Netrin Signaling	1	**9.77E-04**
Wnt/β-catenin Signaling	0.310	**1.20E-02**
Androgen Signaling	0.331	**1.51E-02**
eNOS Signaling	0.248	**5.89E-03**

Supplementary Table S5. The lists of overlapping genes between BPA-responsive genes in the hippocampus and the dysregulated genes in ASD post-mortem brain tissues

DEGs from Voineague, I. et al. (2011)	Overlapping Genes with DEGs in Both Sexes (206 genes)	Overlapping Genes with DEGs in Male (80 genes)	Overlapping genes with DEGs in Female (159 genes)
A2BP1	ABCB9	ACSBG1	ABCA1
A2BP1	ABCG4	AEBP1	ABCC5
A2BP1	ACSBG1	ANGPTL4	ABCC8
ABCA1	ADCYAP1	APOE	ABCG4
ABCB9	ADORA2B	AQP4	ABLIM2
ABCB9	ADPRHL1	ATP1A3	ACSBG1
ABCC5	AEBP1	ATP1B1	ACSL4
ABCC8	AGA	ATP8B2	ADORA2B
ABCG4	ALDH18A1	ATRNL1	AEBP1
ABLIM2	ANGPTL4	BRD2	AHI1
ABTB1	ANK1	BRPF1	ALDH18A1
ACOT7	ANKRD6	CABIN1	AMPH
ACSBG1	ANXA2	CABP1	ANAPC4
ACSL4	APOE	CADPS2	ANK1
ACSL5	AQP4	CDC25B	ANKRD6
ACTL6B	ARHGAP10	CHD5	ANXA2
ACTN1	ARMCX5	CKB	APOE
ACTR1B	ATP1B1	COBLL1	AQP4
ADCYAP1	ATP2B2	CPEB2	ATP1A1
ADFP	ATP2B3	CRYM	ATP1A3
ADM	ATRNL1	DFFA	ATP1B1
ADORA2B	BAG3	DHRS9	ATP2B2
ADPRHL1	BRD2	DTNA	ATP2B3
AEBP1	BRPF1	ERC1	ATP8B2
AES	C1QB	FBLN2	ATRNL1
AGA	CADPS	GPR68	BRD2
AGTRL1	CADPS2	GRIN3A	BRSK1
AHI1	CBLN4	HEY2	CABIN1
AKR1C2	CCDC90B	HLA-A	CACHD1
AKR1C3	CDC14B	HSPB1	CADPS
AKT1	CDC25B	IGFBP7	CADPS2
ALDH18A1	CDH11	INA	CAMK1G
ALDH1A3	CDS1	KCNB1	CELSR3
ALDH1L1	CHKB	KIF5A	CHRM1
ALDH4A1	CLDN5	LRP1	COL6A1
ALPL	CLYBL	LYSMD4	CPSF1
AMPH	COL6A1	MAL2	CYBA
AMT	COMMD2	MAP4	DDIT4

AMY2B	CPEB2	MET	DNAJB1
ANAPC4	CPSF1	MSI2	DPYSL3
ANGPTL4	CRYZ	NFIB	DTNA
ANK1	CXCL16	NOV	DUSP8
ANKRA2	CYB5D2	NRIP3	DYNC1I1
ANKRD15	DDIT4	NRP1	EEF2K
ANKRD20A1	DFFA	NSF	ELAVL4
ANKRD29	DNAJB1	OGDHL	EMP1
ANKRD6	DPP6	OSBPL8	EMP3
ANKRD9	DPYSL3	PALLD	EMX1
ANTXR1	DTNA	PCSK2	EPHX1
ANXA1	DYNC1I1	PDGFRA	EYA2
ANXA11	EAF1	PHYHIP	FAM89B
ANXA2	ELK1	PKD1	FDFT1
AP2A2	EMP3	PLOD3	GFAP
APBA2BP	EMX1	PPP1R13B	GLRB
APOC1	EPHX1	PPP1R16B	GRIN3A
APOE	ERC1	PTPRZ1	GYPC
AQP4	EYA2	RAB11FIP3	HARS
ARC	FAM3C	RHBDL3	HES6
ARFGAP1	FAM81A	RPS2	HIPK2
ARHGAP10	FDFT1	S100A10	HSP90AA1
ARMC10	FGFRL1	SH3D19	IDI1
ARMC8	GABRA1	SLC6A17	IGFBP4
ARMCX2	GABRB3	SNAP25	IGFBP5
ARMCX5	GADD45B	SOX4	IGFBP7
ARPC5	GAS8	SPARC	IGFBPL1
ARPP-21	GBP2	SPTBN1	KCNH4
ARRDC4	GFAP	STOX2	KCNT1
ASB13	GFRA2	SYT13	KIF1C
ASCC3L1	GLRB	TAGLN3	KIFC2
ATP1A1	GLS2	TIMP1	KIT
ATP1A1	GPR83	TNRC6A	LIME1
ATP1A1	GPX3	TRIM37	LRFN4
ATP1A3	GRIN3A	TSPYL2	LRP1
ATP1B1	GSTM3	TXNIP	LRP4
ATP2B2	GYPC	UBE2O	LYSMD4
ATP2B2	HARS	VPS13C	MAGI1
ATP2B3	HAVCR2	WASF1	MAL2
ATP6V0D1	HEY2	ZBTB40	MAP2K1
ATP6V1A	HLA-A	ZNF248	MAP3K12
ATP6V1C1	HSPB1	ZNF358	MAP7D2
ATP6V1G2	HSPB6		MCL1
ATP8B2	IER3		ME3

ATRNL1	IGFBP4		MFGE8
B3GNT6	IL17RB		MIB2
BAG3	ITGB1BP1		MRPS26
BAIAP2	KATNA1		MRPS30
BAIAP2L2	KCNB2		MYADM
BCAN	KCNMA1		NAPB
BCAS4	KCNT1		NCALD
BCHE	KIF1C		NCDN
BID	KIF5A		NCOA1
BLMH	KIFC2		NDRG2
BMS1L	KIT		NELL2
BRD2	LGI3		NEUROD6
BRPF1	MAL2		NFIB
BRSK1	MAOB		NGEF
BRSK2	MAP2K1		NOV
BST2	MAP3K12		NRIP3
BTBD11	MAP4		NRP1
BTG1	MAP7D2		NSDHL
BTG3	MAPK10		NSF
C10orf10	MCL1		PALLD
C10orf116	ME3		PCP4
C10orf54	MERTK		PDGFRA
C12orf24	MIB2		PDZD4
C13orf16	MRPS30		PFKP
C14orf106	MSX1		PGAP1
C1orf131	MYLIP		PLOD1
C1orf173	NAPB		PLXDC2
C1orf52	NDRG2		PPM1F
C1orf54	NELL2		PRKDC
C1orf59	NGB		PRMT2
C1QB	NGEF		PTPRR
C1QC	NOV		PTPRZ1
C20orf100	NOXA1		RAB11FIP3
C20orf103	NRIP3		RAB11FIP5
C20orf28	NSDHL		RAD23B
C20orf58	NSF		RASIP1
C20orf94	OAF		RDH10
C21orf2	OGDHL		REXO1
C21orf33	OLR1		RPS2
C3orf18	OPN3		RYR2
C5orf16	PAK6		SAMD14
C6orf117	PALLD		SDF2L1
C6orf190	PCDH20		SETBP1
C6orf32	PCP4		SEZ6

C6orf79	PCSK2		SH3D19
C9orf10OS	PDE7B		SLC15A3
C9orf45	PDE8B		SLC17A6
C9orf61	PDK4		SLC25A27
C9orf86	PELI3		SLC9A5
C9orf88	PLOD3		SLC9A9
CABIN1	PLXDC2		SMYD2
CABP1	PNMA3		SNAP25
CACHD1	PPP1R13B		SOX4
CACNA1A	PPP1R3C		SPARC
CACNA2D3	PRDX4		SPTBN1
CADPS	PREPL		SQLE
CADPS	PRICKLE1		STK36
CADPS2	PRKDC		SULF1
CALB2	PRMT2		SVOP
CAMK1G	PRRT2		TGM2
CAMK2G	PTGS2		THBS3
CAMK2N2	PTK2B		TLE2
CARHSP1	PTPRR		TNFRSF1A
CARM1	PTPRZ1		TNNT2
CART	PVALB		TNRC6A
CBLN2	RAB11FIP3		TOMM20
CBLN4	RAD23B		TRIM37
CCBL2	RANGAP1		TSPAN9
CCDC25	RASGRP1		TUBGCP5
CCDC34	REEP6		UCHL1
CCDC50	RGS12		UGCG
CCDC69	RGS2		VPS13C
CCDC90B	RHBDL3		WASF1
CCNB1IP1	RIMS4		WDR19
CCPG1	RND2		ZBTB16
CD14	RNF165		ZBTB40
CD14	RPN2		ZFYVE19
CD24	RPS2		
CD300LG	RYR2		
CD44	SCN4B		
CD74	SFT2D1		
CD99	SH3GL2		
CDC14B	SLC15A3		
CDC25B	SLC25A29		
CDH10	SLC4A8		
CDH11	SLC6A17		
CDH12	SLC7A8		
CDKN1A	SLC9A5		

CDS1	SOD2		
CEBPD	SORCS1		
CELSR3	SOX4		
CFLAR	SP110		
CGI-38	SPTBN1		
CHD5	SQLE		
CHGA	STAM		
CHGB	SYT13		
CHKB	TAGLN2		
CHN2	TAGLN3		
CHRD	TCERG1L		
CHRM1	TGM2		
CIRBP	THBS3		
CITED2	TIAM1		
CKB	TIMP1		
CKMT1A	TNFRSF1A		
CLCN3	TRIM37		
CLDN10	TRIP13		
CLDN5	TSPAN9		
CLIC1	TSPO		
CLTB	TTC17		
CLYBL	TYROBP		
CLYBL	UGCG		
CMKOR1	UPP1		
CMTM7	VAMP8		
CNN3	VAV3		
CNNM1	VHL		
COBLL1	VIP		
COL20A1	VPS13C		
COL4A1	WDR19		
COL6A1	ZBTB16		
COL7A1	ZBTB40		
COMMD2	ZFYVE19		
COPG	ZHX2		
CORO6	ZNF365		
CORT	ZSWIM4		
COX7A1			
CPEB2			
CPLX1			
CPLX2			
CPNE3			
CPSF1			
CRH			
CROCC			

CRYM			
CRYM			
CRYZ			
CSDA			
CSDC2			
CSEN			
CSMD2			
CSPG2			
CTNNAL1			
CTSH			
CUTL1			
CXCL16			
CYB5D2			
CYBA			
CYGB			
CYP2C8			
CYP2E1			
D4S234E			
DACH1			
DAK			
DBC1			
DCAMKL1			
DDIT4			
DDR1			
DFFA			
DGCR2			
DHRS9			
DIAPH1			
DIRAS1			
DIXDC1			
DIXDC1			
DKFZP564O0823			
DLX1			
DMAP1			
DNAJB1			
DNM1L			
DPP6			
DPYSL3			
DRD1IP			
DSCR1L1			
DSCR2			
DSCR2			
DSCR5			
DTNA			

DTNA			
DTNA			
DUSP5			
DUSP8			
DYNC1I1			
DYNLT1			
EAF1			
ECE2			
ECGF1			
EEF2K			
ELAVL4			
ELK1			
ELMOD1			
EMP1			
EMP3			
EMX1			
EMX1			
ENDOGL1			
ENO2			
ENTPD3			
EPHB6			
EPHX1			
ERC1			
ESRRA			
ESRRG			
ESTRRA			
ETS2			
EXTL2			
EYA2			
F12			
FAIM			
FAIM2			
FAM100A			
FAM102B			
FAM3C			
FAM3C			
FAM43A			
FAM46A			
FAM73B			
FAM81A			
FAM89B			
FBLN2			
FBLN2			
FBXO31			

FCGBP			
FDFT1			
FES			
FGF9			
FGFRL1			
FHL2			
FILIP1			
FKBP8			
FLJ11286			
FLJ20054			
FLJ20152			
FLJ20647			
FLJ25006			
FLJ25222			
FLJ25476			
FLJ30596			
FLJ30834			
FLJ37078			
FLJ37440			
FLJ39822			
FLJ40142			
FLJ90805			
FLRT3			
FNDC4			
FNDC5			
FOXO3A			
FREQ			
FRMPD2			
FRMPD2			
FSD1			
FSTL5			
FTHL11			
FTHL3			
G1P3			
G3BP2			
GABRA1			
GABRA5			
GABRB3			
GABRD			
GABRG2			
GABRG2			
GAD1			
GAD2			
GADD45B			

GADD45G			
GALGT			
GAS8			
GBP2			
GCA			
GFAP			
GFOD1			
GFRA2			
GLRB			
GLS2			
GNA12			
GNA13			
GOLGA8B			
GPNMB			
GPR56			
GPR56			
GPR68			
GPR83			
GPRASP2			
GPX3			
GRIN1			
GRIN3A			
GRK4			
GSDML			
GSDML			
GSTM3			
GSTT1			
GYPC			
H2AFY2			
HAMP			
HAPLN4			
HARS			
HAVCR2			
HCLS1			
HERC6			
HES6			
HEY2			
HIPK2			
HIST1H1C			
HIST1H2BD			
HIST1H2BK			
HIST2H2AA3			
HIST2H2AC			
HIST2H2BE			

HLA-A			
HLA-H			
HMFN0839			
HNRPU			
HPRT1			
HSP90AA1			
HSPA1A			
HSPA1B			
HSPB1			
HSPB3			
HSPB6			
HSPB8			
HTATIP2			
HTR4			
HYLS1			
ICA1			
ICA1			
ID3			
IDH3B			
IDI1			
IER3			
IFITM1			
IFITM2			
IFITM3			
IGFBP4			
IGFBP5			
IGFBP7			
IGFBPL1			
IGSF4			
IGSF4B			
IL17RB			
INA			
INPPL1			
INSM2			
INTS1			
INTU			
ISG20L1			
ISOC1			
ITGB1BP1			
ITGB2			
ITGB5			
ITPKA			
ITPR1			
JUN			

KATNA1			
KCNA5			
KCNB1			
KCNB2			
KCNG1			
KCNH3			
KCNH4			
KCNIP4			
KCNJ16			
KCNK12			
KCNMA1			
KCNMB4			
KCNN3			
KCNS1			
KCNT1			
KIAA0350			
KIAA0427			
KIAA0528			
KIAA0664			
KIAA1026			
KIAA1576			
KIAA1688			
KIAA1797			
KIF17			
KIF1C			
KIF2			
KIF5A			
KIFC2			
KIT			
KLF15			
KLHL8			
KNDC1			
LAMA5			
LAPTM5			
LARP2			
LCMT1			
LCP1			
LGALS3			
LGI3			
LHX6			
LIME1			
LIMK1			
LMO3			
LOC126208			

LOC144363			
LOC202134			
LOC283871			
LOC387856			
LOC387856			
LOC388419			
LOC400566			
LOC400566			
LOC649853			
LPHN2			
LRFN4			
LRP1			
LRP1B			
LRP4			
LRRC42			
LRRN6C			
LUZP1			
LY96			
LYN			
LYPD1			
LYPLAL1			
LYSMD4			
MAGI1			
MAL2			
MAOB			
MAP2K1			
MAP3K12			
MAP3K13			
MAP3K6			
MAP4			
MAP7D2			
MAPK10			
MAPK9			
MAPRE2			
MARCH2			
MASS1			
MATK			
MC1R			
MCL1			
ME3			
MEG3			
MEG3			
MERTK			
MET			

METRN			
MFGE8			
MGC11257			
MGC26718			
MGC33846			
MGC40405			
MGC4172			
MGC99813			
MGP			
MGST1			
MGST1			
MIB2			
MKNK2			
MLSTD1			
MOBKL2B			
MPP1			
MRCL3			
MRPS26			
MRPS30			
MSI2			
MSN			
MSX1			
MT1X			
MT2A			
MTP18			
MUM1L1			
MYADM			
MYBPC1			
MYBPHL			
MYLIP			
MYOZ3			
NAG6			
NAGPA			
NAP1L5			
NAPB			
NAPG			
NAT6			
NBPF10			
NBPF20			
NCALD			
NCALD			
NCDN			
NCOA1			
NCOA3			

NDRG2			
NDRG2			
NEDD4L			
NEFH			
NEFL			
NEFM			
NELL1			
NELL2			
NEUROD6			
NFIB			
NFKBIZ			
NGB			
NGEF			
NGFRAP1L1			
NLGN4Y			
NME5			
NMNAT2			
NOS2A			
NOV			
NOVA1			
NOXA1			
NP			
NPPA			
NPY			
NQO1			
NR2F1			
NR2F2			
NRIP3			
NRP1			
NSDHL			
NSF			
NSFL1C			
NTSR2			
NUDCD2			
NUDCD3			
NUDT14			
OAF			
OGDHL			
OLR1			
OPN3			
OPRK1			
OR2L13			
OSBP2			
OSBPL8			

OVGP1			
OXR1			
P4HA1			
PABPC1			
PAK1			
PAK6			
PALLD			
PANX2			
PCDH10			
PCDH20			
PCDH7			
PCNT2			
PCNXL2			
PCP4			
PCSK1			
PCSK2			
PDE1B			
PDE7B			
PDE8B			
PDGFRA			
PDK4			
PDYN			
PDZD4			
PELI3			
PFKP			
PFTK1			
PGAP1			
PHF16			
PHF17			
PHGDHL1			
PHKA2			
PHYHIP			
PIAS2			
PIB5PA			
PICK1			
PIR			
PITPNC1			
PKD1			
PKD1			
PLCXD3			
PLEKHA6			
PLEKHC1			
PLOD1			
PLOD2			

PLOD3			
PLSCR4			
PLTP			
PLXDC2			
PNKD			
PNMA3			
PNMA6A			
POPDC3			
PPARGC1A			
PPEF1			
PPFIA4			
PPGB			
PPM1F			
PPM2C			
PPP1R13B			
PPP1R16B			
PPP1R3C			
PPP2R1A			
PRDX4			
PREPL			
PRICKLE1			
PRKCB1			
PRKCB1			
PRKCE			
PRKCG			
PRKDC			
PRKRIP1			
PRMT2			
ProSAPiP1			
PRPSAP2			
PRRT2			
PRRX1			
PTBP1			
PTD004			
PTGS2			
PTHR2			
PTK2B			
PTPLA			
PTPRR			
PTPRT			
PTPRZ1			
PTTG1IP			
PUNC			
PVALB			

PVRL3			
PYGB			
RAB11FIP3			
RAB11FIP5			
RAB33A			
RAB37			
RAB37			
RAB40C			
RAD23B			
RAGE			
RAI14			
RALGDS			
RANGAP1			
RAPGEF6			
RAPGEFL1			
RARA			
RARRES3			
RASAL1			
RASD2			
RASGEF1C			
RASGRP1			
RASIP1			
RASSF4			
RBED1			
RBM9			
RDH10			
RECQL5			
REEP6			
REXO1			
RGS12			
RGS2			
RGS4			
RGS7			
RHBDF2			
RHBDL1			
RHBDL3			
RHPN2			
RIMS4			
RLTPR			
RN7SK			
RND2			
RNF157			
RNF165			
RORB			

RPIB9			
RPN2			
RPS15A			
RPS2			
RPS29			
RTN1			
RTN4			
RWDD2			
RXRA			
RXRB			
RYR2			
S100A10			
S100A16			
S100A8			
SAMD14			
SAPS3			
SAR1B			
SAT			
SBP1			
SC4MOL			
SCAMP5			
SCARA3			
SCARA3			
SCCPDH			
SCHIP1			
SCN1A			
SCN1B			
SCN2B			
SCN3B			
SCN4B			
SCNN1D			
SDF2L1			
SEMA3C			
SEMA5B			
SEP6			
SERPINA3			
SERTAD1			
SERTAD4			
SETBP1			
SETD1A			
SEZ6			
SF3A3			
SFMBT2			
SFRP1			

SFT2D1			
SGK			
SH3D19			
SH3GL2			
SHD			
SKIP			
SLC15A3			
SLC16A3			
SLC16A9			
SLC17A6			
SLC25A12			
SLC25A27			
SLC25A29			
SLC25A37			
SLC29A1			
SLC2A5			
SLC32A1			
SLC35C2			
SLC39A10			
SLC4A8			
SLC6A17			
SLC6A7			
SLC7A2			
SLC7A6			
SLC7A8			
SLC9A1			
SLC9A5			
SLC9A9			
SLN			
SMCY			
SMYD2			
SNAP25			
SNAPC4			
SNCB			
SNCB			
SNHG5			
SNTB1			
SOCS2			
SOD2			
SORCS1			
SOX2			
SOX4			
SOX9			
SP110			

SPARC			
SPTBN1			
SPTBN4			
SQLE			
SRC			
SRGN			
SSBP3			
SSBP4			
SST			
STAC2			
STAM			
STAMBPL1			
STARD10			
STAT4			
STEAP2			
STEAP2			
STK36			
STOX2			
STS-1			
STXBP1			
STXBP1			
STXBP5			
SULF1			
SVOP			
SYN2			
SYT13			
SYTL4			
TAC1			
TAC3			
TACC2			
TADA1L			
TADA3L			
TAGLN2			
TAGLN3			
TAP1			
TARBP1			
TCBA1			
TCEAL6			
TCEB2			
TCERG1L			
TCF4			
TESC			
TGFBRAP1			
TGM2			

THBS3			
TIAM1			
TIMP1			
TLE2			
TM4SF1			
TMBIM1			
TMED10P			
TMEFF2			
TMEM117			
TMEM25			
TMUB2			
TNFRSF1A			
TNFRSF25			
TNK2			
TNNT2			
TNPO1			
TNRC6A			
TOM1			
TOMM20			
TOX			
TRAK2			
TRIM24			
TRIM37			
TRIM37			
TRIP13			
TRPM2			
TSGA14			
TSPAN9			
TSPO			
TSPYL2			
TTC17			
TUBGCP5			
TWIST1			
TXNIP			
TYROBP			
UBA52			
UBE2L3			
UBE2O			
UBQLN4			
UCHL1			
UCHL3			
UCHL5			
UCRC			
UGCG			

UGCGL2			
ULK1			
ULK1			
UNG2			
UNQ2541			
UPF2			
UPP1			
UQCRC1			
USF2			
USP13			
VAMP1			
VAMP1			
VAMP8			
VAV3			
VAV3			
VGF			
VHL			
VIM			
VIP			
VIP			
VPS13C			
WAS			
WASF1			
WDR19			
WDR74			
WHDC1			
YAP1			
YBX1			
YPEL4			
YY1AP1			
ZADH2			
ZBTB16			
ZBTB16			
ZBTB40			
ZFP36			
ZFP36L1			
ZFYVE19			
ZHX2			
ZNF218			
ZNF248			
ZNF25			
ZNF265			
ZNF312			
ZNF358			

ZNF365			
ZNF385			
ZNF540			
ZNF659			
ZNF689			
ZSWIM4			
ZYX			
ZYX			
**DEGs from Parikshak, N. et al. (2016)**	**Overlapping Genes with DEGs in Both Sexes (1045 genes)**	**Overlapping Genes with DEGs in Male (393 genes)**	**Overlapping genes with DEGs in Female (690 genes)**
A1BG	ABCA5	ABCA3	AATK
AARS	ABCA9	ABHD3	ABCA1
AASDHPPT	ABCC12	ACAT2	ABCA2
AATK	ABCC4	ACP2	ABCA3
ABCA1	ABCC6	ACTN4	ABCA5
ABCA17P	ABCG4	ADAM11	ABCA9
ABCA2	ABHD16B	ADAM12	ABCC8
ABCA3	ABHD2	ADAMTSL1	ABCG4
ABCA5	ABHD6	ADCK2	ABHD2
ABCA9	ACAA2	ADCYAP1R1	ABLIM2
ABCB1	ACAT2	AEBP1	ACAT2
ABCC11	ACLY	AFG3L2	ACLY
ABCC12	ACOT13	AGTPBP1	ACP2
ABCC4	ACOX2	AKAP12	ACSS3
ABCC6	ACP2	AMER3	ACTN4
ABCC8	ACSS3	AMMECR1L	ADAM11
ABCD1	ADAM11	AMOT	ADAM12
ABCG2	ADAM12	ANGPTL4	ADAM9
ABCG4	ADAM23	APBB2	ADAMTS12
ABHD10	ADAM9	APMAP	ADARB2
ABHD12	ADAMTS15	APOE	ADCYAP1R1
ABHD16B	ADAMTS4	AREL1	ADD2
ABHD17C	ADAMTS9	ARHGAP20	ADD3
ABHD2	ADAMTSL1	ARHGAP44	ADORA2B
ABHD3	ADCK1	ARHGEF11	ADTRP
ABHD4	ADCK2	ARHGEF26	AEBP1
ABHD5	ADCYAP1R1	ARMC7	AGBL3
ABHD6	ADD2	ARMCX4	AGL
ABI1	ADH5	ARPC1B	AHNAK
ABLIM2	ADI1	ARPC5L	AIFM1
ABTB1	ADORA2B	ARRDC3	AK5
ABTB2	ADTRP	ASAP2	AKAP12
ACAA2	AEBP1	ATG14	AKAP6

ACACA	AFF1	ATG2B	ALDH7A1
ACAD9	AFG3L2	ATP1A3	AMOT
ACADS	AGK	ATP1B1	AMZ1
ACADSB	AGL	ATRNL1	ANGPT1
ACAP2	AHCYL2	B4GALNT1	ANK1
ACAT2	AIF1L	BCAS3	ANK3
ACBD3	AIFM1	BCL11B	ANKRD6
ACKR3	AKAP12	BEND3	ANKS1A
ACLY	AKAP6	BNIP2	ANXA2
ACOT13	AKR1A1	BRAF	ANXA5
ACOT4	ALDH7A1	BRPF1	AP5Z1
ACOT7	ALDH9A1	BTBD19	APBA2
ACOX1	AMER3	BUD13	APBB2
ACOX2	AMH	C15orf39	APOE
ACP2	AMIGO1	C16orf58	ARHGAP20
ACRC	AMOT	C1QTNF4	ARHGAP44
ACSL5	ANGPTL4	C3orf70	ARHGAP5
ACSL6	ANK1	C4orf46	ARHGEF11
ACSS2	ANKRD6	C8orf46	ARHGEF2
ACSS3	ANO6	CABP1	ARHGEF25
ACTL6A	ANXA2	CACTIN	ARHGEF40
ACTL6B	ANXA5	CADPS2	ARHGEF7
ACTN4	APBB2	CALR	ARIH2
ACTR1A	APMAP	CAMK4	ARL8A
ACTR1B	APOE	CANX	ARMC7
ACTR3C	AREL1	CAPZA1	ARMCX4
ADAM11	ARHGAP20	CBL	ARMCX6
ADAM12	ARHGAP42	CCDC85C	ARPC5L
ADAM17	ARHGEF11	CCK	ARRDC3
ADAM22	ARHGEF25	CCNI	ASAP2
ADAM23	ARHGEF26	CCSER2	ASNS
ADAM32	ARHGEF37	CDC42EP4	ATG4D
ADAM8	ARHGEF39	CENPL	ATP1A1
ADAM9	ARID5A	CHD2	ATP1A3
ADAMTS12	ARIH2	CHD8	ATP1B1
ADAMTS15	ARL6IP5	CLK4	ATP2B2
ADAMTS4	ARMC7	CNOT6	ATP2B3
ADAMTS9	ARMCX4	CNTN2	ATP8A2
ADAMTS9-AS2	ARPC1B	CNTNAP1	ATR
ADAMTSL1	ARRDC3	COBLL1	ATRN
ADARB2	ASNS	COL1A1	ATRNL1
ADAT2	ATG14	COL8A2	ATXN7L3B
ADCK1	ATP10A	COQ4	BCCIP
ADCK2	ATP1B1	COX4I1	BCL6

ADCK3	ATP2B2	CPE	BEND3
ADCY1	ATP2B3	CPEB3	BMPR1A
ADCY10	ATP2C2	CPNE4	BNIP2
ADCYAP1R1	ATP6AP1	CREB1	BRAF
ADD1	ATP6V0A1	CRMP1	BRSK1
ADD2	ATP8A2	CRY1	BTBD19
ADD3	ATRNL1	CRYM	C16orf58
ADH5	ATXN7L3B	CTTNBP2	C1orf216
ADI1	AZI2	CYTH1	C2CD5
ADIRF	B4GALNT1	DARS	C3orf70
ADK	B4GALT1	DCC	CA12
ADM	BAG3	DCHS1	CACHD1
ADO	BAZ1A	DDHD2	CACNG2
ADORA1	BCL2L2	DDX42	CADM1
ADORA2B	BDH1	DENND2A	CADM4
ADPRH	BHLHA15	DFFA	CADPS
ADRA1A	BICD1	DHX36	CADPS2
ADRA1D	BMP2K	DIRAS2	CALCOCO1
ADRBK2	BMP7	DMXL1	CALR
ADSS	BOLA3	DNALI1	CAMK4
ADTRP	BRAF	DNASE2	CAMKK1
AEBP1	BRD8	DNLZ	CAND2
AEN	BRPF1	DNMBP	CANX
AES	BTBD19	DOPEY1	CAPN1
AFF1	BTK	DPH7	CAPN2
AFF4	BUD13	DTD1	CBL
AFG3L2	C15orf39	DTNA	CBX7
AFTPH	C16orf58	DTX2	CCDC85C
AGBL3	C1orf228	DUSP19	CCK
AGK	C1orf74	DYNC1H1	CCSER2
AGL	C1QB	DYNLL2	CD163
AGPAT2	C1R	EFNB1	CD47
AGTPBP1	C20orf196	EGLN1	CD81
AGTRAP	C3orf70	ELAVL2	CD93
AHCY	C4orf19	EML4	CDC25A
AHCYL1	C5AR1	EPB41L2	CDC42EP4
AHCYL2	C5orf22	EPHA5	CDC42SE2
AHNAK	CA13	ERAP1	CDH1
AHNAK2	CACTIN	ERCC6	CDH6
AIF1L	CADM2	EZH1	CDHR2
AIFM1	CADPS	FABP7	CDK5
AK3	CADPS2	FAM114A2	CDKL2
AK4	CALR	FAM131B	CDKN2AIP
AK5	CAMK4	FAM171B	CEBPZ

AKAP11	CAMKK1	FANCM	CEND1
AKAP12	CAPN1	FASN	CENPL
AKAP13	CAPN2	FBXL17	CHD2
AKAP6	CAPS2	FBXO46	CHI3L1
AKNA	CASP4	FBXW9	CHORDC1
AKR1A1	CAV2	FCGR2A	CHPT1
AKR1C2	CBFB	FER	CHRM1
AKR1C3	CBLN4	FIBIN	CHST11
AKT3	CBX7	FLNA	CHST7
ALDH1L1	CCDC113	FLYWCH2	CHSY1
ALDH3B1	CCDC151	FOXJ1	CLASP2
ALDH7A1	CCDC40	FOXO1	CLMP
ALDH9A1	CCDC51	FRY	CLSTN1
ALG13	CCDC62	FTH1	CLTA
ALG5	CCDC81	FTL	CLU
ALKBH3	CCDC90B	FTSJ1	CNTNAP1
ALPK1	CCNE1	G3BP1	CNTNAP2
AMER3	CCNG1	GALNT11	CNTNAP4
AMH	CCSER2	GATM	COL12A1
AMIGO1	CD163	GDAP1	COL16A1
AMMECR1L	CD302	GHR	COL1A1
AMN1	CD320	GJA1	COL25A1
AMOT	CD4	GLCE	COL27A1
AMOTL2	CD40	GLT1D1	COL4A2
AMZ1	CD47	GLUD1	COL6A1
AMZ2P1	CD59	GPAM	COL9A3
ANAPC1	CD63	GPBP1	COMMD9
ANAPC13	CDC14A	GPD1	COPG1
ANAPC15	CDC25A	GPR158	COQ3
ANAPC5	CDC42EP4	GPSM1	COX19
ANAPC7	CDC42SE2	GRIA3	CP
ANGPT1	CDC7	GRIP1	CPE
ANGPTL4	CDCA3	HAGHL	CPEB3
ANK1	CDK10	HARBI1	CPNE4
ANK2	CDK14	HBB	CREB1
ANK3	CDKL2	HECTD1	CRMP1
ANKFN1	CDS1	HECW1	CRY1
ANKMY2	CEND1	HERC1	CSE1L
ANKRA2	CENPL	HERC3	CSF1
ANKRD18DP	CEP112	HID1	CSMD1
ANKRD29	CEP89	HMCN1	CSNK1A1
ANKRD32	CEP97	HNRNPU	CSTF2
ANKRD36	CFD	HSD3B7	CTSC
ANKRD36BP1	CGRRF1	HSP90B1	CX3CR1

ANKRD36C	CHD2	HSPA12A	CYBA
ANKRD45	CHD8	HSPA5	DAAM2
ANKRD6	CHPT1	HSPB1	DCAF17
ANKS1A	CHRDL1	HSPD1	DCC
ANKS1B	CHRNB3	IFT172	DCHS1
ANO6	CHST11	IGDCC4	DDIT4
ANP32E	CHSY1	IGLON5	DDR2
ANTXR2	CIAO1	INA	DDX24
ANXA2	CLK4	INTS5	DDX42
ANXA2P2	CLMP	IQSEC1	DENND1C
ANXA5	CLVS1	IQSEC3	DMXL1
ANXA6	CNDP1	ITGB8	DNAJB1
ANXA7	CNTNAP1	KANSL1L	DNAJC10
AP1AR	CNTNAP5	KBTBD11	DNAJC18
AP2S1	COL16A1	KCNB1	DNAJC2
AP3M1	COL1A1	KCTD1	DNM3
AP3M2	COL25A1	KCTD12	DOCK3
AP3S1	COL27A1	KDM5A	DOK5
AP4B1	COL4A2	KDM6B	DOPEY1
AP5Z1	COL6A1	KIAA0368	DOPEY2
APBA2	COMMD9	KIAA0513	DPH7
APBA3	COQ3	KIAA1755	DPYSL3
APBB1	COQ4	KIAA1841	DTNA
APBB2	COX19	KIF21A	DUSP3
APEX1	CP	KIF7	DYNC1LI1
APLP1	CPE	KIFAP3	DYNLRB1
APMAP	CPEB4	KLC4	ECM1
APOA1BP	CPNE4	KLF9	EFEMP1
APOBR	CREG2	KLHDC1	EGLN1
APOC1	CRMP1	KMT2C	EID2
APOE	CRY1	L3MBTL3	EIF4E
APOL6	CSF2RB	LAMB1	EIF4H
APP	CSRP2	LDB1	ELAVL4
APPBP2	CTBS	LIN54	EMILIN1
APPL1	CXCL16	LRCH2	EML1
AR	CYB5D2	LRP11	EML6
AREL1	CYTH1	LRRC40	EMP1
ARFGAP1	CYTH4	LSS	EMP3
ARFGAP3	DALRD3	LTN1	ENPP2
ARFGEF2	DARS	MAG	EPB41L2
ARFIP1	DARS2	MAL2	EPHX1
ARHGAP15	DBH	MAP1B	EPS15
ARHGAP18	DBX2	MCF2	ERAP1
ARHGAP20	DCLRE1C	ME1	ESYT1

ARHGAP26	DCP1A	MED13	ETV1
ARHGAP30	DCUN1D2	MEF2C	EXTL1
ARHGAP31	DDIT3	MEN1	EYA2
ARHGAP42	DDIT4	METTL22	EZH1
ARHGAP44	DDR2	MEX3D	FABP5
ARHGAP5	DDX24	MFN1	FABP7
ARHGAP5-AS1	DDX42	MFSD3	FADS3
ARHGAP6	DEF6	MIER3	FAM107A
ARHGDIG	DENND1C	MLC1	FAM114A2
ARHGEF11	DENND2C	MMAB	FAM161A
ARHGEF2	DEPTOR	MOAP1	FAM168B
ARHGEF25	DFFA	MSI2	FAM171B
ARHGEF26	DHX29	MTMR2	FAM184A
ARHGEF37	DLGAP1	MTMR4	FAM212B
ARHGEF39	DMXL1	NAA25	FAM217B
ARHGEF40	DNAH2	NAPEPLD	FAM220A
ARHGEF6	DNAJB1	NCAN	FAM57A
ARHGEF7	DNALI1	NDUFA10	FAM69A
ARHGEF9	DNLZ	NDUFS2	FAM69B
ARID3A	DNM3	NDUFS4	FASN
ARID5A	DNTTIP2	NF1	FBLN5
ARIH2	DOK3	NFIB	FBXO30
ARL1	DOK5	NHLRC3	FCGRT
ARL13B	DPH2	NHSL1	FER
ARL2BP	DPP6	NPAS3	FERMT2
ARL4D	DPYSL3	NPHP1	FEZ1
ARL6	DSC2	NREP	FLNA
ARL6IP5	DSE	NRIP3	FLYWCH2
ARL8A	DSN1	NRSN2	FNDC3A
ARL8B	DTNA	NSF	FNDC3B
ARMC1	DUSP12	NTN1	FOCAD
ARMC7	DUSP19	NTN3	FOXO1
ARMC8	DUSP3	NUDT21	FSTL4
ARMCX3	DYNC1H1	NUP98	FTL
ARMCX4	DYNLRB1	OGDH	FYN
ARMCX6	ECHDC3	OGDHL	FZD8
ARPC1A	ECM1	OSBPL11	GABRA2
ARPC1B	EDN3	OSTF1	GABRB1
ARPC5L	EDNRB	OTUD4	GABRG1
ARRDC3	EFEMP1	PABPC1L2A	GAP43
ARRDC4	EFNA5	PALLD	GARNL3
ARSG	EFNB1	PARP2	GARS
ASAH2B	EFR3A	PCDH18	GATM
ASAP2	EHD3	PCSK2	GDAP1

ASAP3	EIF2B5	PDE1A	GDI1
ASB13	EIF2D	PEX5L	GGCX
ASB8	EIF4E3	PFKM	GHITM
ASCC2	ELK1	PHYHIP	GJA1
ASIC2	ELK3	PIK3C2B	GJB2
ASIC4	ELMO1	PIP5K1A	GLCE
ASMTL	EMC9	PLD3	GLG1
ASNS	EMILIN1	PLEKHM3	GLRB
ASPHD1	EML1	PLIN2	GLT1D1
ASPHD2	EML6	POP7	GLUD1
ASTN2	EMP3	POSTN	GLYR1
ASXL2	ENPP5	PPA1	GMPR2
ATAD1	ENSA	PPM1H	GNB1
ATAD3C	EPB41L2	PPP1R14B	GNL1
ATF1	EPB41L3	PPP4R2	GPBP1
ATF7IP2	EPDR1	PRDM4	GPR85
ATG13	EPHX1	PREP	GPSM1
ATG14	EPS8	PRICKLE4	GRIA3
ATG16L1	ERAP1	PRUNE2	GRIN2A
ATG2B	ERCC6	PTPRD	GRIP1
ATG4D	ESYT1	PTPRZ1	GUCY1A2
ATHL1	ETV1	PYGL	GXYLT1
ATOH8	EVC	RALGAPA2	GYPC
ATP10A	EVC2	RBM12B	HACL1
ATP10B	EXOC1	RCN1	HAGHL
ATP10D	EXOC3	RCOR2	HAPLN1
ATP13A2	EXTL1	RCOR3	HARS
ATP1A1	EYA2	REPS2	HAS2
ATP1A3	EZH2	REV3L	HBB
ATP1B1	FA2H	RGS22	HDAC1
ATP2B1	FABP5	RHOBTB3	HECTD3
ATP2B2	FABP7	RHOQ	HECW1
ATP2B3	FAF1	RIMKLB	HECW2
ATP2C2	FAHD2B	RIOK1	HERC1
ATP5A1	FAM107A	RNASET2	HERC2
ATP5B	FAM114A2	RNF114	HERC3
ATP5C1	FAM118B	RNF169	HHIP
ATP5D	FAM131B	RNF182	HIC1
ATP5F1P5	FAM161A	RPH3A	HNRNPU
ATP5G3	FAM168B	RPL6	HS2ST1
ATP5O	FAM172A	RPLP0	HSP90B1
ATP6AP1	FAM184A	RPS2	HSPA5
ATP6AP1L	FAM20A	RPS6KL1	IAH1
ATP6AP2	FAM212B	RSRC1	ICAM1

ATP6V0A1	FAM3C	RTCB	IFNAR1
ATP6V0D1	FAM46B	RUSC1	IFT172
ATP6V0E1	FAM46C	RYK	IGDCC4
ATP6V1A	FAM69A	S100A10	IGFBP2
ATP6V1B2	FAM71E1	SAMD12	IGFBP5
ATP6V1C1	FAM81A	SASH1	IL6ST
ATP6V1D	FANCM	SCAI	IMP3
ATP6V1E1	FANK1	SDR39U1	IMPAD1
ATP8A2	FARSA	SEMA4C	INIP
ATP9A	FARSB	SERINC1	INTS10
ATPAF1	FBLN7	SERINC3	INTS12
ATR	FBXL17	SH2B2	IQSEC1
ATRN	FBXO4	SH3D19	IQSEC3
ATRNL1	FBXO44	SH3GL3	IRF2BP1
ATXN3	FBXO9	SLAIN2	ITFG1
ATXN7L3	FCGR2A	SLC10A4	ITGB8
ATXN7L3B	FCGRT	SLC12A5	ITIH3
AUH	FER	SLC16A7	ITPA
AVPI1	FERMT2	SLC1A3	ITPKC
AXL	FERMT3	SLC24A2	ITPR2
AZI2	FEZ1	SLC25A4	KANK1
B3GNT1	FGF11	SLC25A46	KANSL1L
B4GALNT1	FGFR4	SLC26A11	KATNAL1
B4GALT1	FGFRL1	SLC2A12	KBTBD3
B4GALT6	FIBP	SLC35F3	KCNA2
BACE2	FIGNL1	SLC39A7	KCNQ5
BAG2	FIS1	SLC4A4	KDELC2
BAG3	FLNA	SLC50A1	KDELR3
BANF1	FNIP2	SLC6A11	KIAA0368
BANP	FOCAD	SLC6A17	KIAA0513
BAZ1A	FRG1	SLC9A6	KIAA1324L
BBOX1	FSD2	SLITRK1	KIAA1551
BBS7	FSTL4	SLITRK3	KIAA1755
BCAM	FTH1	SLX4IP	KIF1B
BCAP29	FTL	SMC3	KIF21A
BCAR3	FTO	SMG1	KIF7
BCAS2	FUT10	SNAP91	KLHDC3
BCAS3	FXR2	SNX10	KMT2C
BCCIP	FYN	SOX4	L3MBTL3
BCDIN3D	FZD8	SPOCK2	LAMB1
BCL10	G3BP1	SRCIN1	LAMC1
BCL11A	GABPB1	SRGAP3	LAP3
BCL11B	GABRA1	SRSF11	LATS2
BCL2	GABRB1	STRA6	LDB1

BCL2L1	GABRG1	STX3	LHFPL2
BCL2L11	GADD45B	SUCLA2	LMNB2
BCL2L13	GALM	SUPT5H	LRP11
BCL2L2	GALNT11	SYNC	LRRC20
BCL3	GALNT3	SYNGR3	LSS
BCL6	GARNL3	SYNJ1	LTBP1
BCMO1	GATM	SYP	LTBP4
BDH1	GBP2	SYT13	LTN1
BECN1	GDI1	TAGLN3	LYNX1
BEND3	GFRA2	TBL1X	MAL2
BEND6	GGH	TCF12	MAOA
BEND7	GHR	TECPR2	MAP1B
BEX1	GJA1	TF	MAP3K12
BEX2	GJB2	TM6SF1	MAP3K5
BEX5	GK5	TMEM43	MAP3K9
BHLHA15	GLG1	TMEM63B	MAP7D2
BICD1	GLRB	TNKS1BP1	MAPRE1
BIRC3	GLS2	TRIM33	MARK3
BIVM	GLUD1	TRIM37	MCF2
BLMH	GLYR1	TRPS1	MCL1
BLVRA	GMPR2	TSC1	MCM4
BMF	GOT1	TSHR	MDK
BMP1	GPAM	TUBB	ME2
BMP2K	GPBP1	TUBGCP4	ME3
BMP7	GPC4	TXNDC9	MED13
BMPER	GPD1L	TXNIP	MEF2C
BMPR1A	GPD2	TYRO3	METTL22
BMPR1APS2	GPI	UBE3C	MFN1
BMPR1B	GPR156	UHRF2	MICU1
BNIP2	GPR158	UNC13A	MIIP
BNIP3L	GPR160	UNC13D	MKI67
BOLA3	GPR85	UNC80	MKNK1
BOP1	GRAMD1B	USP14	MLC1
BRAF	GRIK1	USP19	MMP11
BRAFP1	GRIN2A	USP38	MON2
BRD7	GSS	VCPIP1	MPPED1
BRD8	GTF3C6	WDR1	MPV17
BRPF1	GUCY1A2	WDR17	MRPS26
BRSK1	GXYLT1	WDR36	MTMR3
BST2	GYPC	WDR54	MVK
BTBD10	HACE1	WSB1	MYH9
BTBD19	HACL1	XPO1	MYO5A
BTF3L4	HAGHL	YBX3	MYOF
BTG1	HAPLN1	ZFAND3	NACC2

BTG2	HARS	ZFAND6	NALCN
BTG3	HAUS8	ZMYM2	NAMPT
BTK	HAVCR2	ZNF248	NAPB
BTN2A2	HBB	ZNF251	NBEA
BTN2A3P	HCK	ZNF316	NCALD
BTN3A1	HDAC1	ZNF426	NCAN
BTN3A3	HECW1	ZNF445	NDFIP1
BUD13	HERC1	ZSCAN21	NDUFA4
BZRAP1	HERC3		NDUFB9
C10orf111	HEYL		NDUFS1
C10orf118	HIBCH		NDUFS4
C10orf35	HIC1		NEBL
C10orf88	HID1		NEGR1
C11orf24	HINT1		NELL2
C11orf68	HIPK3		NEUROD6
C11orf74	HIST1H2AH		NFIB
C11orf83	HLA-DRB5		NGEF
C12orf10	HMG20A		NHLRC3
C12orf23	HMG20B		NHSL1
C12orf45	HNRNPU		NICN1
C12orf52	HP1BP3		NID1
C12orf68	HPS6		NIPA2
C14orf2	HS2ST1		NNT
C14orf23	HSD11B1		NOP58
C15orf39	HSF1		NOTCH2
C15orf61	HSP90B1		NPAS3
C16orf45	HSPA5		NRBP2
C16orf52	HSPB1		NREP
C16orf58	HSPD1		NRIP3
C16orf72	ICAM1		NSF
C17orf67	IDH1		NTN1
C19orf73	IFNAR1		NUAK1
C1orf106	IFT172		NUCKS1
C1orf112	IGDCC4		NUP98
C1orf162	IL10RA		NUPR1
C1orf174	IL17RB		NXPH1
C1orf192	IL33		ODC1
C1orf213	IL4R		OGDH
C1orf216	IMP3		OPA1
C1orf228	INIP		OSBPL11
C1orf56	INPP4A		OTUD4
C1orf74	INPP5K		OTX1
C1orf86	IPMK		PABPC1L2A
C1QA	IQSEC1		PABPN1

C1QB	IQSEC3		PAFAH1B1
C1QC	ITGB8		PAICS
C1QL1	ITPKC		PAIP2
C1QTNF4	ITPR2		PALLD
C1R	KANSL3		PAPD5
C1S	KATNAL1		PARP2
C2	KCNA1		PARP6
C20orf166-AS1	KCNA2		PBXIP1
C20orf196	KCNAB1		PCLO
C20orf203	KCNAB3		PCP4
C2CD4A	KCNB2		PCSK4
C2CD5	KCNE4		PDCD6IP
C2orf16	KCNH8		PDE4A
C2orf49	KCNJ3		PDIA6
C3AR1	KCNQ5		PEBP1
C3orf18	KCTD1		PEPD
C3orf49	KIAA0368		PEX5L
C3orf62	KIAA0513		PFKM
C3orf70	KIAA1551		PFKP
C3orf80	KIAA1755		PGAP1
C4A	KIF21A		PI4KA
C4orf19	KLC4		PIGK
C4orf27	KLF9		PIGL
C4orf46	KLHDC1		PIH1D2
C5AR1	KLHDC2		PINK1
C5orf22	KLHDC3		PKN1
C5orf42	KLHL11		PLCB1
C5orf51	KMT2C		PLCB4
C5orf64	KPNA6		PLD3
C6orf106	L3MBTL3		PLEC
C6orf118	LACTB		PLOD1
C6orf136	LAMC1		PLXDC2
C6orf211	LAP3		PLXNA2
C6orf62	LAT2		PNMA2
C6orf70	LATS2		PNP
C7orf26	LCA5		POLR2B
C7orf43	LCP2		POMGNT1
C7orf55-LUC7L2	LDB1		POR
C7orf61	LGI2		PPP1R14B
C8orf46	LGI3		PPP1R21
C8orf82	LGR4		PPP1R3B
C9orf37	LHFPL2		PPP1R9A
C9orf72	LIN54		PPP2R2D
C9orf84	LMNB2		PPP2R5A

CA10	LOXL2		PREP
CA12	LOXL3		PREX2
CA13	LPP		PRICKLE4
CA14	LRP11		PRKAR1A
CAB39	LRRC20		PRKAR2B
CABP1	LRRIQ1		PRKCB
CACHD1	LSP1		PRPF40A
CACNA2D2	LSR		PRPF4B
CACNA2D3	LSS		PRR18
CACNB1	LTBP1		PRUNE2
CACNB4	LTBR		PSMB4
CACNG2	LYNX1		PSMD1
CACTIN	MAD2L1BP		PTGES3
CADM1	MAG		PTPRD
CADM2	MAGEE1		PTPRZ1
CADM4	MAL2		PUS10
CADPS	MALL		QDPR
CADPS2	MAN1B1		RAB11FIP5
CAHM	MAOB		RAB2A
CALCOCO1	MAP2K5		RAB3GAP2
CALM2	MAP2K7		RAB3IL1
CALM3	MAP3K12		RAD23A
CALR	MAP3K5		RALGAPA1
CAMK2B	MAP3K8		RALGAPA2
CAMK2G	MAP6D1		RAMP2
CAMK4	MAP7D2		RANBP3
CAMKK1	MAPK10		RAP1B
CAMKK2	MAPKAPK3		RASGRF1
CAMTA1	MAS1		RB1
CAND1	MCC		RBBP6
CAND2	MCF2		RBM12B
CANX	MCL1		RCN1
CAP2	ME1		RCN3
CAPG	ME2		RCOR3
CAPN1	ME3		RDH10
CAPN10-AS1	MED21		REPS2
CAPN13	MEF2C		RET
CAPN15	MEN1		REV3L
CAPN2	MEOX2		RHBDF1
CAPNS1	MERTK		RIMKLB
CAPS2	METTL22		RNF112
CAPZA1	MFSD10		RNF14
CARD14	MFSD3		RNMT
CARD9	MID2		ROGDI

CARHSP1	MIIP		RPL24
CARKD	MKNK1		RPL6
CARNS1	MKX		RPS2
CASC10	MLC1		RPS6KL1
CASC5	MMAB		RTCB
CASD1	MMP11		RTN3
CASP1	MOAP1		RYR2
CASP4	MOB1B		S1PR1
CASP7	MORC3		SALL3
CASS4	MPPED1		SAMD12
CAV2	MRPL42		SAT1
CBFB	MRPL55		SCN8A
CBL	MRPS21		SCN9A
CBLN2	MRPS23		SDF2L1
CBLN4	MRPS9		SEMA4C
CBX7	MSANTD4		SEMA7A
CCBE1	MSX1		SERINC1
CCBL2	MTF2		SERINC3
CCDC102A	MTHFD2		SERPINH1
CCDC104	MTHFD2L		SESTD1
CCDC109B	MTMR2		SH3D19
CCDC11	MTMR3		SH3GLB1
CCDC113	MTMR4		SH3RF3
CCDC115	MXD1		SLAIN2
CCDC117	MYBBP1A		SLC12A5
CCDC120	MYH7		SLC15A3
CCDC126	MYO1F		SLC16A7
CCDC132	MYOF		SLC1A3
CCDC136	MYT1L		SLC1A5
CCDC151	N4BP3		SLC22A15
CCDC154	NAA25		SLC24A2
CCDC160	NAA50		SLC25A27
CCDC178	NADK		SLC25A39
CCDC18	NAP1L3		SLC25A46
CCDC25	NAPB		SLC26A11
CCDC36	NAPEPLD		SLC29A4
CCDC40	NBEA		SLC2A10
CCDC51	NBEAL2		SLC2A12
CCDC62	NDUFA10		SLC35B3
CCDC73	NDUFB2		SLC35B4
CCDC74B	NDUFS1		SLC35F3
CCDC8	NDUFS2		SLC36A4
CCDC81	NEBL		SLC38A5
CCDC85C	NEK3		SLC3A2

CCDC90B	NELL2		SLC41A1
CCIN	NET1		SLC4A4
CCK	NEXN		SLC50A1
CCM2	NFATC1		SLC6A11
CCNE1	NFATC2IP		SLC6A6
CCNF	NFE2L3		SLC7A11
CCNG1	NFKB2		SLC7A5
CCNI	NFU1		SLC8A1
CCNI2	NGEF		SLC9A9
CCR10	NICN1		SMC3
CCSER2	NID1		SMOX
CD14	NIPA2		SMTN
CD151	NIPAL3		SMYD2
CD163	NIT1		SNAP91
CD200	NLN		SNTG1
CD274	NMRAL1		SNX10
CD276	NNT		SOX4
CD300A	NPAS3		SPHK1
CD302	NPHP1		SRCIN1
CD320	NPL		SRF
CD34	NPY5R		SRGAP1
CD4	NREP		SRRT
CD40	NRG1		SS18
CD44	NRIP3		ST18
CD47	NSF		ST8SIA1
CD53	NTN3		STK39
CD58	NUAK1		STMN2
CD59	NUCKS1		STMN3
CD63	NUDT18		STRBP
CD81	NUSAP1		STXBP4
CD93	NXT2		SUMO3
CD99	OAF		SUSD4
CD99L2	ODC1		SVOP
CD99P1	OGDH		SYNGR1
CDC123	OGDHL		SYNGR3
CDC14A	OGG1		SYNJ1
CDC25A	OLFM3		SYNJ2
CDC42	OPA1		SYT16
CDC42EP4	OPALIN		SYT17
CDC42SE1	OPN3		SYT4
CDC42SE2	OSBPL11		TADA2B
CDC7	OSGEPL1		TBC1D10B
CDCA3	OSGIN1		TBC1D22B
CDCA5	OSMR		TBR1

CDCP1	OSTF1		TDRKH
CDH1	OTUD4		TEAD1
CDH18	OTUD7A		TECPR2
CDH6	PABPC1L2A		TEP1
CDHR2	PABPC4		TERF2IP
CDHR3	PAICS		TES
CDK10	PALLD		TFB1M
CDK12	PAPD5		TGFB2
CDK14	PARD3B		TGM2
CDK2AP1	PARL		THBD
CDK2AP2	PARP2		TIMP2
CDK4	PARP6		TLE3
CDK5	PARP9		TMC7
CDKAL1	PAX6		TMEM14A
CDKL2	PBX4		TMEM150C
CDKN1A	PBXIP1		TMEM55A
CDKN2AIP	PCDHB14		TMEM63B
CDKN2AIPNL	PCDHB2		TMX1
CDKN2D	PCGF5		TMX4
CDPF1	PCP4		TNFAIP8
CDS1	PCSK2		TNFRSF11B
CDS2	PCSK4		TNFRSF1A
CDT1	PCSK5		TNFRSF1B
CEBPB	PDE1A		TNNT2
CEBPD	PDE4A		TOMM20
CEBPZ	PDE6B		TOPBP1
CECR6	PDGFC		TOR1A
CELSR1	PDIA6		TOX2
CELSR2	PELI3		TRHDE
CEND1	PEX14		TRIM33
CENPL	PEX5L		TRIM36
CEP112	PFKM		TRIM37
CEP63	PIGA		TRIO
CEP68	PIGL		TRIP10
CEP70	PIK3AP1		TROVE2
CEP85	PIK3C2B		TSEN34
CEP89	PIK3R4		TTC28
CEP97	PIM1		TTC37
CERCAM	PIN1		TUBB
CERK	PIP5K1A		TUBB4A
CES3	PIRT		TUBGCP4
CFD	PITRM1		TUBGCP5
CFI	PKN1		TULP4
CFLAR	PKNOX1		TYK2

CGRRF1	PLA2G6		UBAP2
CH25H	PLAUR		UBE2H
CHCHD1	PLB1		UBE2QL1
CHCHD4	PLCB1		UBE2T
CHD1	PLCB4		UBE3C
CHD2	PLCD4		UBLCP1
CHD8	PLEKHG1		UBR3
CHGA	PLEKHG2		UBXN4
CHGB	PLEKHH1		UCHL1
CHI3L1	PLEKHM3		UFSP2
CHIC1	PLEKHO1		UGGT1
CHM	PLLP		UNC13A
CHMP3	PLSCR1		UNC5C
CHORDC1	PLXDC1		UNC80
CHPT1	PLXDC2		USO1
CHRDL1	PMS1		VEGFA
CHRM1	PNMA3		VGLL4
CHRM4	PNRC2		VPS41
CHRNB2	PODN		WDR1
CHRNB3	POFUT1		WDR17
CHST11	POLE3		WDR36
CHST3	POLR2B		WDR7
CHST7	POMGNT1		WDR75
CHSY1	PON2		WLS
CHUK	POP7		WSB1
CIAO1	POR		YPEL3
CIAPIN1	PPA1		ZBTB33
CIDEA	PPA2		ZC3H6
CIDECP	PPARA		ZCWPW2
CIITA	PPAT		ZEB1
CINP	PPIL6		ZFAND6
CIRBP	PPM1H		ZFYVE9
CISD1	PPM1L		ZMAT2
CKMT1B	PPP1R14C		ZMYM2
CLASP2	PPP1R21		ZNF180
CLCN3	PPP1R3B		ZNF251
CLCN4	PPP2R2D		ZNF518B
CLDN10	PPP2R5A		ZNF536
CLDN18	PREP		ZNF579
CLEC2L	PREX2		ZNF740
CLIC1	PRICKLE1		ZSCAN21
CLIP1	PRKAR1B		
CLK4	PRKCB		
CLMP	PRKD3		

CLP1	PRR22		
CLPX	PRSS35		
CLSPN	PSD4		
CLSTN1	PSMB8		
CLSTN3	PSMC3		
CLTA	PSMD1		
CLTC	PSPH		
CLU	PSTPIP1		
CLUHP3	PTH2R		
CLVS1	PTPN1		
CLVS2	PTPN22		
CMAS	PTPN6		
CMC2	PTPRC		
CMTM3	PTPRD		
CMTM5	PTPRZ1		
CMTM6	PVALB		
CNDP1	PYCR1		
CNIH3	PYGL		
CNN2	PYGO1		
CNN3	QRFPR		
CNNM1	RAB13		
CNOT6	RAB27A		
CNP	RAB3GAP2		
CNRIP1	RAB3IL1		
CNTLN	RALGAPA1		
CNTN2	RAMP2		
CNTNAP1	RANBP1		
CNTNAP2	RANBP3		
CNTNAP4	RANGAP1		
CNTNAP5	RAP1B		
COA1	RASAL3		
COBLL1	RASGRF1		
COL12A1	RASGRP1		
COL14A1	RASSF8		
COL16A1	RAVER2		
COL19A1	RB1		
COL1A1	RBFOX1		
COL25A1	RBM12B		
COL27A1	RBM47		
COL4A1	RCAN2		
COL4A2	RCN1		
COL4A3BP	RCN3		
COL4A5	RCOR2		
COL6A1	RCOR3		

COL7A1	RECQL4		
COL8A1	REPS2		
COL8A2	RET		
COL9A3	RFXANK		
COLGALT1	RGS14		
COMMD9	RGS16		
COPG1	RGS2		
COPS3	RGS22		
COPS4	RGS6		
COPS5	RHBDF1		
COPS6	RHOBTB3		
COPS7A	RILP		
COQ3	RIMKLB		
COQ4	RIN3		
COQ6	RINT1		
CORO2A	RIOK1		
CORO2B	RNF112		
COTL1	RNF114		
COX19	RNF144A		
COX4I1	RNF146		
COX5A	RNF167		
COX5B	RNF169		
COX6C	RNF34		
COX7A1	RNF5		
COX7A2	ROGDI		
COX7A2L	RPAIN		
CP	RPL24		
CPAMD8	RPL27A		
CPE	RPL28		
CPEB3	RPL6		
CPEB4	RPLP0		
CPLX1	RPN2		
CPLX2	RPRD1B		
CPNE3	RPS2		
CPNE4	RPS6KL1		
CPNE9	RRAS		
CPOX	RSU1		
CPT1A	RTCA		
CRADD	RTCB		
CREB1	RUFY2		
CREG2	RUNX1		
CREM	RWDD2B		
CRH	RYK		
CRHR2	RYR2		

CRK	S100A9		
CRKL	S1PR5		
CRMP1	SAMD12		
CRTAC1	SAMD5		
CRY1	SAMD8		
CRYM	SAMHD1		
CSE1L	SARDH		
CSF1	SBNO2		
CSF2RB	SCG5		
CSMD1	SCN4B		
CSNK1A1	SCN8A		
CSPP1	SCRN2		
CSRP2	SDC1		
CSRP2BP	SDC2		
CSTB	SDF4		
CSTF2	SDHA		
CTBS	SDS		
CTCF	SDSL		
CTH	SEMA4C		
CTNNBIP1	SENP2		
CTPS1	SERINC3		
CTR9	SERPINB9		
CTSC	SERPINE1		
CTSH	SERPINH1		
CTSS	SERTAD2		
CTTNBP2	SESN2		
CTXN3	SFXN4		
CUBN	SH2D4A		
CUEDC2	SH3GL2		
CUL1	SH3GL3		
CUL2	SHROOM4		
CUZD1	SIAE		
CX3CR1	SLAIN2		
CXCL16	SLC10A4		
CXCL5	SLC12A5		
CXCR4	SLC12A8		
CXorf22	SLC15A3		
CXorf40A	SLC16A7		
CXorf40B	SLC17A5		
CYB5B	SLC17A9		
CYB5D2	SLC1A3		
CYB5R2	SLC22A15		
CYBA	SLC24A2		
CYBRD1	SLC25A20		

CYC1	SLC25A40		
CYFIP2	SLC25A46		
CYP11A1	SLC26A11		
CYP26B1	SLC2A10		
CYP2C8	SLC2A4		
CYP4F11	SLC35B4		
CYP4X1	SLC35F3		
CYSTM1	SLC38A5		
CYTH1	SLC39A1		
CYTH2	SLC39A12		
CYTH4	SLC3A2		
DAAM2	SLC43A1		
DACH1	SLC43A3		
DACT2	SLC4A11		
DALRD3	SLC4A4		
DARS	SLC4A8		
DARS2	SLC50A1		
DBC1	SLC5A2		
DBH	SLC6A11		
DBI	SLC6A17		
DBX2	SLC6A6		
DCAF13	SLC7A5		
DCAF17	SLC7A8		
DCAF4L1	SLC8A1		
DCAF8	SLC9B2		
DCC	SLITRK1		
DCHS1	SMAD4		
DCLK1	SMAD5		
DCLRE1C	SMIM14		
DCP1A	SMNDC1		
DCTN1	SMTN		
DCTN1-AS1	SMUG1		
DCTN2	SMURF2		
DCTN6	SNAP91		
DCUN1D2	SNRPG		
DCUN1D4	SNTG1		
DDAH1	SNX10		
DDAH2	SNX18		
DDHD2	SOAT1		
DDIT3	SOCS5		
DDIT4	SOD2		
DDR2	SOX4		
DDX1	SP110		
DDX21	SPATS2L		

DDX24	SPDEF		
DDX3Y	SPHK1		
DDX42	SPTY2D1		
DEF6	SRD5A3		
DEGS2	SRPK2		
DENND1C	SRRM4		
DENND2A	SRRT		
DENND2C	SS18		
DEPDC5	SSX2IP		
DEPTOR	ST14		
DERL1	ST18		
DERL2	ST3GAL5		
DERL3	STAM		
DES	STARD8		
DFFA	STIP1		
DFNB59	STK16		
DGKI	STK17B		
DHCR24	STOM		
DHDDS	STPG1		
DHPS	STRA6		
DHRS11	STRN		
DHX29	STX1A		
DHX30	STXBP4		
DHX34	STYX		
DHX35	STYXL1		
DHX36	SUCLG1		
DHX57	SUMO3		
DIO1	SUPT5H		
DIRAS1	SUPV3L1		
DIRAS2	SUV39H1		
DLAT	SV2A		
DLEC1	SYNC		
DLG1	SYNGR1		
DLG3	SYNGR3		
DLGAP1	SYNJ1		
DLK2	SYNJ2		
DMPK	SYNPO2		
DMRTA1	SYP		
DMXL1	SYPL2		
DMXL2	SYT13		
DNAH10	SYT16		
DNAH11	SYT17		
DNAH2	TAB2		
DNAJA4	TADA2B		

DNAJB1	TAGLN2		
DNAJC1	TAGLN3		
DNAJC10	TBC1D10A		
DNAJC16	TBC1D22B		
DNAJC17	TBC1D25		
DNAJC18	TBC1D30		
DNAJC2	TBK1		
DNAJC27-AS1	TBR1		
DNAJC3	TBRG1		
DNAJC6	TBX15		
DNALI1	TECPR2		
DNASE1L2	TERF2IP		
DNASE2	TFB1M		
DND1P1	TG		
DNLZ	TGIF1		
DNM1	TGM2		
DNM1L	THAP1		
DNM3	THAP7		
DNMBP	THBD		
DNTTIP2	TIMM17A		
DOCK2	TIPARP		
DOCK3	TKT		
DOCK6	TLE3		
DOCK7	TLR5		
DOHH	TMC5		
DOK3	TMC7		
DOK5	TMCO4		
DOPEY1	TMED3		
DOPEY2	TMEM120A		
DPH2	TMEM144		
DPH7	TMEM167B		
DPP10	TMEM175		
DPP6	TMEM176A		
DPP8	TMEM178A		
DPP9	TMEM185B		
DPY19L2	TMEM255A		
DPY19L2P1	TMEM30A		
DPY19L2P2	TMEM38A		
DPYSL3	TMEM43		
DRAXIN	TMEM63A		
DRG1	TMX1		
DSC2	TNFRSF1A		
DSE	TNFRSF1B		
DSN1	TNIP1		

DSTN	TNKS1BP1		
DTD1	TOR1A		
DTNA	TOR1AIP2		
DTWD2	TPD52L2		
DTX2	TPI1		
DTX3	TPST1		
DTX3L	TRAIP		
DTX4	TRAM1L1		
DTYMK	TRAPPC10		
DUS2L	TRHDE		
DUSP12	TRIM14		
DUSP19	TRIM37		
DUSP3	TRIP13		
DYNC1H1	TRPC3		
DYNC1LI1	TRPS1		
DYNC2LI1	TSC1		
DYNLL2	TTC23		
DYNLRB1	TTC28		
DYNLT1	TTC37		
DYRK3	TTC38		
E2F1	TTL		
EBNA1BP2	TTPA		
ECE1	TUBB4A		
ECHDC3	TUBGCP4		
ECI2	TUSC3		
ECM1	TYRO3		
EDA	UBAP2		
EDN3	UBE2E3		
EDNRB	UBE2H		
EEF1A1P11	UBE2QL1		
EEF1A1P8	UBE3C		
EEFSEC	UBXN4		
EFCAB12	UFSP2		
EFEMP1	UGT8		
EFEMP2	UHRF1BP1L		
EFNA5	UNC13A		
EFNB1	UNC5C		
EFR3A	UNC80		
EGLN1	UPP1		
EGLN3	UROD		
EHBP1L1	USHBP1		
EHD1	USO1		
EHD3	USP38		
EHHADH	USP39		

EID2	USP42		
EID2B	USP48		
EIF2AK1	UVRAG		
EIF2AP4	VAMP8		
EIF2B1	VAPB		
EIF2B3	VASP		
EIF2B5	VCPIP1		
EIF2D	VHL		
EIF3E	VIP		
EIF3F	VPS33B		
EIF3J	VSTM5		
EIF3K	VWA2		
EIF4A2	VWA3B		
EIF4E	WDR1		
EIF4E2	WDR17		
EIF4E3	WDR36		
EIF4EBP1	WDR7		
EIF4G3	WDR77		
EIF4H	WDR91		
EIF5A2	WDYHV1		
ELAVL2	WFS1		
ELAVL4	XPO6		
ELF4	XRCC6		
ELK1	ZBBX		
ELK3	ZBED5		
ELMO1	ZBTB42		
ELMOD2	ZC3H12A		
ELMSAN1	ZC3H6		
ELOVL1	ZCCHC11		
ELOVL2	ZEB1		
ELOVL4	ZFAND6		
ELOVL5	ZMAT4		
ELP2	ZMYM2		
ELP6	ZNF133		
EMC8	ZNF157		
EMC9	ZNF251		
EME2	ZNF287		
EMILIN1	ZNF365		
EMILIN3	ZNF385B		
EML1	ZNF385D		
EML2	ZNF426		
EML4	ZNF445		
EML6	ZNF518B		
EMP1	ZSCAN21		

EMP3			
EMX2			
EMX2OS			
ENAH			
ENDOV			
ENKUR			
ENO2			
ENOPH1			
ENOSF1			
ENPP2			
ENPP5			
ENPP6			
ENSA			
ENTPD4			
ENY2			
EPB41L2			
EPB41L3			
EPCAM			
EPDR1			
EPHA5			
EPHA8			
EPHB6			
EPHX1			
EPHX4			
EPS15			
EPS8			
ERAL1			
ERAP1			
ERBB2			
ERBB3			
ERCC4			
ERCC6			
ERGIC3			
ERI1			
ERLIN1			
ERLIN2			
ERMN			
ERO1LB			
ESR1			
ESRRG			
ESYT1			
ETNK2			
ETS2			
ETV1			

ETV3			
EVC			
EVC2			
EXO5			
EXOC1			
EXOC3			
EXOSC6			
EXOSC9			
EXTL1			
EXTL2			
EXTL3			
EYA2			
EYA4			
EZH1			
EZH2			
EZR			
F2R			
F3			
FA2H			
FAAH			
FABP5			
FABP7			
FADS3			
FADS6			
FAF1			
FAF2			
FAH			
FAHD2A			
FAHD2B			
FAHD2CP			
FAM101A			
FAM101B			
FAM102B			
FAM105B			
FAM107A			
FAM110A			
FAM114A2			
FAM115B			
FAM117A			
FAM118B			
FAM120C			
FAM122C			
FAM126A			
FAM129A			

FAM129B			
FAM131B			
FAM131C			
FAM133A			
FAM134A			
FAM134B			
FAM13A-AS1			
FAM13C			
FAM150B			
FAM161A			
FAM168B			
FAM171B			
FAM172A			
FAM174A			
FAM175A			
FAM179B			
FAM181A			
FAM181A-AS1			
FAM184A			
FAM192A			
FAM203A			
FAM207A			
FAM20A			
FAM20C			
FAM212B			
FAM216A			
FAM217B			
FAM219A			
FAM220A			
FAM221A			
FAM228A			
FAM27D1			
FAM3C			
FAM3C2			
FAM43B			
FAM46A			
FAM46B			
FAM46C			
FAM49B			
FAM57A			
FAM58A			
FAM63B			
FAM65C			
FAM66B			

FAM66C			
FAM69A			
FAM69B			
FAM71E1			
FAM73A			
FAM76A			
FAM78B			
FAM81A			
FAM86A			
FAM86B3P			
FAM98B			
FANCC			
FANCM			
FANK1			
FARP1			
FARSA			
FARSB			
FAS			
FASN			
FAXC			
FBLN5			
FBLN7			
FBXL15			
FBXL17			
FBXL2			
FBXO17			
FBXO22			
FBXO25			
FBXO3			
FBXO30			
FBXO4			
FBXO44			
FBXO45			
FBXO46			
FBXO5			
FBXO8			
FBXO9			
FBXW2			
FBXW7			
FBXW9			
FCGBP			
FCGR2A			
FCGR3A			
FCGRT			

FECH			
FEM1B			
FEM1C			
FER			
FERMT1			
FERMT2			
FERMT3			
FES			
FEZ1			
FGD4			
FGF11			
FGF12			
FGF14			
FGF2			
FGF9			
FGFR1			
FGFR4			
FGFRL1			
FGL1			
FH			
FHL2			
FHL3			
FHOD1			
FIBIN			
FIBP			
FIGNL1			
FIS1			
FKBP10			
FKBP14			
FKBP8			
FLNA			
FLOT2			
FLT3			
FLYWCH2			
FMN1			
FMR1			
FNBP1L			
FNDC3A			
FNDC3B			
FNDC4			
FNDC5			
FNIP2			
FOCAD			
FOLH1			

FOPNL			
FOXE1			
FOXJ1			
FOXO1			
FOXRED1			
FPR1			
FRAS1			
FRG1			
FRG1B			
FRMPD4			
FRS3			
FRY			
FSCN2			
FSD1			
FSD2			
FSTL3			
FSTL4			
FTH1			
FTL			
FTLP3			
FTO			
FTSJ1			
FTSJ2			
FTSJD2			
FUT10			
FXR1			
FXR2			
FXYD3			
FYN			
FZD1			
FZD7			
FZD8			
FZD9			
G3BP1			
G3BP2			
GABARAPL1			
GABPA			
GABPB1			
GABPB2			
GABRA1			
GABRA2			
GABRB1			
GABRB2			
GABRD			

GABRE			
GABRG1			
GABRG2			
GABRQ			
GAD1			
GAD2			
GADD45B			
GADD45G			
GAK			
GALM			
GALNT10			
GALNT11			
GALNT12			
GALNT13			
GALNT3			
GALNT6			
GAP43			
GAPDHP32			
GAPDHP58			
GARNL3			
GARS			
GAS7			
GATAD1			
GATAD2A			
GATM			
GBA			
GBA2			
GBP1			
GBP2			
GBP3			
GBP4			
GCA			
GCFC2			
GCH1			
GDAP1			
GDE1			
GDF6			
GDI1			
GDPD1			
GEM			
GFPT2			
GFRA2			
GGCX			
GGH			

GGT7			
GHDC			
GHITM			
GHR			
GIPC2			
GIT2			
GJA1			
GJB1			
GJB2			
GJC2			
GK5			
GLCCI1			
GLCE			
GLG1			
GLI3			
GLIS3			
GLMN			
GLRB			
GLRX2			
GLS2			
GLT1D1			
GLTSCR1L			
GLUD1			
GLYR1			
GMCL1			
GMIP			
GMNN			
GMPR2			
GMPS			
GNA12			
GNA13			
GNA14			
GNAI1			
GNAI3			
GNAS			
GNB1			
GNB2L1			
GNB5			
GNG12			
GNG5			
GNL1			
GNS			
GOLGA2			
GOLGA8A			

GOLIM4			
GOLM1			
GOPC			
GOT1			
GOT2			
GOT2P2			
GPAA1			
GPAM			
GPATCH1			
GPBP1			
GPC4			
GPC5			
GPCPD1			
GPD1			
GPD1L			
GPD2			
GPI			
GPIHBP1			
GPKOW			
GPLD1			
GPR12			
GPR125			
GPR137B			
GPR137C			
GPR143			
GPR149			
GPR153			
GPR156			
GPR158			
GPR160			
GPR35			
GPR37			
GPR56			
GPR6			
GPR61			
GPR62			
GPR85			
GPR98			
GPRASP2			
GPRIN3			
GPSM1			
GPSM3			
GPT2			
GPX1			

GRAMD1B			
GRAMD2			
GREM2			
GRIA2			
GRIA3			
GRID1			
GRIK1			
GRIK3			
GRIN2A			
GRIP1			
GRPEL2			
GRTP1			
GSDMD			
GSK3B			
GSKIP			
GSPT1			
GSS			
GSTA4			
GSTM4			
GSTO2			
GSTZ1			
GTDC2			
GTF2F2			
GTF2IRD1			
GTF3C2			
GTF3C6			
GTPBP10			
GUCY1A2			
GUCY1B3			
GUSBP1			
GUSBP11			
GXYLT1			
GYPC			
GYPE			
GYS1			
H2AFJ			
H3F3A			
HABP4			
HACE1			
HACL1			
HADHB			
HAGH			
HAGHL			
HAPLN1			

HAR1A			
HARBI1			
HARS			
HAS2			
HAUS8			
HAVCR2			
HBB			
HBQ1			
HBS1L			
HCK			
HCLS1			
HCN1			
HCN4			
HCRTR2			
HDAC1			
HEATR5B			
HEBP2			
HECTD1			
HECTD3			
HECW1			
HECW2			
HELB			
HELLS			
HENMT1			
HERC1			
HERC2			
HERC2P9			
HERC3			
HERC5			
HEXA-AS1			
HEYL			
HGF			
HGSNAT			
HHAT			
HHIP			
HHIPL1			
HHLA3			
HIAT1			
HIBCH			
HIC1			
HID1			
HIF1A			
HINT1			
HIPK1			

HIPK3			
HIST1H1C			
HIST1H2AB			
HIST1H2AC			
HIST1H2AG			
HIST1H2AH			
HIST1H2AI			
HIST1H2BB			
HIST1H2BI			
HIST1H2BJ			
HIST1H2BM			
HIST1H3B			
HIST1H3C			
HIST1H4B			
HIST1H4E			
HIST2H2AC			
HIST2H2BA			
HIST2H2BB			
HIST2H2BE			
HIST2H2BF			
HIVEP2			
HKR1			
HLA-DRB1			
HLA-DRB5			
HLCS			
HLTF			
HLX			
HMBOX1			
HMCN1			
HMG20A			
HMG20B			
HMGB2			
HMGN1			
HMHA1			
HMOX1			
HMOX2			
HNRNPDL			
HNRNPH2			
HNRNPU			
HP1BP3			
HPRT1			
HPS6			
HR			
HRSP12			

HS2ST1			
HS3ST3B1			
HS3ST4			
HSD11B1			
HSD3B1			
HSD3B7			
HSDL2			
HSF1			
HSP90B1			
HSPA12A			
HSPA12B			
HSPA1A			
HSPA1B			
HSPA5			
HSPA6			
HSPB1			
HSPB8			
HSPBP1			
HSPD1			
HSPD1P5			
HTR1F			
HTR3B			
HYLS1			
IAH1			
IARS2			
ICA1			
ICAM1			
ICAM3			
ID4			
IDH1			
IDH3A			
IDH3B			
IDH3G			
IFI16			
IFIH1			
IFITM2			
IFNAR1			
IFNW1			
IFRD1			
IFT172			
IFT27			
IFT52			
IFT74			
IGDCC4			

IGF1R			
IGF2BP2			
IGFBP2			
IGFBP5			
IGFBP6			
IGLON5			
IGSF21			
IGSF8			
IKBKE			
IL10RA			
IL17RA			
IL17RB			
IL17RC			
IL17RD			
IL18BP			
IL1R1			
IL1RAPL1			
IL33			
IL4R			
IL6R			
IL6ST			
IMMT			
IMP3			
IMPA2			
IMPAD1			
INA			
INF2			
ING1			
INIP			
INO80D			
INPP4A			
INPP5F			
INPP5J			
INPP5K			
INPPL1			
INTS10			
INTS12			
INTS4			
INTS4L1			
INTS4L2			
INTS5			
INTU			
IPCEF1			
IPMK			

IPO13			
IQCB1			
IQGAP2			
IQSEC1			
IQSEC3			
IREB2			
IRF1			
IRF2BP1			
IRF2BPL			
IRF5			
IRF7			
IRGQ			
ISCA1			
ISCU			
ITFG1			
ITFG2			
ITGA7			
ITGAL			
ITGAV			
ITGB2			
ITGB5			
ITGB7			
ITGB8			
ITIH3			
ITIH5			
ITM2A			
ITM2C			
ITPA			
ITPK1			
ITPKC			
ITPR1			
ITPR2			
ITPRIP			
JAK3			
JAKMIP1			
KAL1			
KALP			
KALRN			
KANK1			
KANSL1L			
KANSL3			
KARS			
KAT7			
KATNAL1			

KATNB1			
KAZN			
KBTBD11			
KBTBD3			
KCNA1			
KCNA2			
KCNAB1			
KCNAB3			
KCNB1			
KCNB2			
KCNC2			
KCNE4			
KCNF1			
KCNG2			
KCNH1			
KCNH5			
KCNH8			
KCNIP4			
KCNJ11			
KCNJ16			
KCNJ3			
KCNJ4			
KCNJ9			
KCNK1			
KCNN3			
KCNN4			
KCNQ1			
KCNQ5			
KCNS1			
KCNS3			
KCNV1			
KCTD1			
KCTD12			
KCTD6			
KCTD9			
KDELC2			
KDELR3			
KDM1B			
KDM2B			
KDM3A			
KDM5A			
KDM5D			
KDM6B			
KIAA0040			

KIAA0196			
KIAA0226L			
KIAA0232			
KIAA0319L			
KIAA0368			
KIAA0430			
KIAA0513			
KIAA1045			
KIAA1107			
KIAA1109			
KIAA1143			
KIAA1217			
KIAA1279			
KIAA1324L			
KIAA1432			
KIAA1456			
KIAA1467			
KIAA1549L			
KIAA1551			
KIAA1755			
KIAA1841			
KIDINS220			
KIF1B			
KIF21A			
KIF22			
KIF23			
KIF3A			
KIF4B			
KIF7			
KIFAP3			
KLB			
KLC1			
KLC2			
KLC4			
KLF13			
KLF5			
KLF6			
KLF9			
KLHDC1			
KLHDC2			
KLHDC3			
KLHDC9			
KLHL11			
KLHL15			

KLHL21			
KLHL22			
KLHL3			
KLHL42			
KLHL6			
KLRAP1			
KMT2C			
KPNA6			
KRT5			
KRT8P12			
KRT8P26			
KTN1			
L2HGDH			
L3MBTL2			
L3MBTL3			
LACTB			
LAIR1			
LAMA5			
LAMB1			
LAMC1			
LAMTOR4			
LANCL1			
LAP3			
LAPTM5			
LARP4			
LARP6			
LAT2			
LATS2			
LCA5			
LCMT1			
LCP1			
LCP2			
LDB1			
LDB2			
LDB3			
LDLRAD4			
LECT1			
LEPREL1			
LETM2			
LETMD1			
LGALS9			
LGI2			
LGI3			
LGR4			

LHFPL1			
LHFPL2			
LHX2			
LHX6			
LIG4			
LILRB1			
LIMD1			
LIMK1			
LIMK2			
LIMS1			
LIN52			
LIN54			
LIN7C			
LINC00032			
LINC00342			
LINC00404			
LINC00470			
LINC00473			
LINC00507			
LINC00511			
LINC00599			
LINC00639			
LINC00667			
LINC00672			
LINC00683			
LINC00689			
LINC00693			
LINC00882			
LINC00886			
LINC00898			
LINC00963			
LINC00966			
LMAN2L			
LMBRD1			
LMNB2			
LMO4			
LNP1			
LOH12CR1			
LONRF3			
LOXL2			
LOXL3			
LPAR2			
LPCAT4			
LPGAT1			

LPIN1			
LPP			
LPP-AS2			
LRAT			
LRCH2			
LRIG1			
LRP10			
LRP11			
LRRC10B			
LRRC16A			
LRRC20			
LRRC3			
LRRC37A6P			
LRRC37B			
LRRC39			
LRRC40			
LRRC47			
LRRC58			
LRRC8A			
LRRC8B			
LRRIQ1			
LRRK2			
LSM11			
LSM14B			
LSM6			
LSP1			
LSR			
LSS			
LTBP1			
LTBP2			
LTBP4			
LTBR			
LTN1			
LY6E			
LYN			
LYNX1			
LYPD1			
LYPLAL1			
LYRM4			
LYSMD2			
LYSMD3			
LYZ			
LZTR1			
MACF1			

MAD2L1			
MAD2L1BP			
MAD2L2			
MADD			
MAFF			
MAG			
MAGEE1			
MAGEF1			
MAGOH			
MAL2			
MALL			
MAMDC4			
MAML2			
MAMLD1			
MAN1B1			
MAN2A2			
MANBAL			
MANSC1			
MAOA			
MAOB			
MAP10			
MAP1B			
MAP1LC3B2			
MAP2K3			
MAP2K4			
MAP2K5			
MAP2K7			
MAP3K12			
MAP3K19			
MAP3K2			
MAP3K5			
MAP3K8			
MAP3K9			
MAP4K2			
MAP4K3			
MAP6D1			
MAP7D2			
MAP7D3			
MAPK10			
MAPK8			
MAPK8IP2			
MAPK9			
MAPKAPK2			
MAPKAPK3			

MAPRE1			
MARCH3			
MARCH6			
MARK3			
MAS1			
MAT2B			
MATN2			
MBP			
MBTPS2			
MC1R			
MCAM			
MCC			
MCF2			
MCHR1			
MCHR2			
MCL1			
MCM4			
MCMBP			
MCMDC2			
MCOLN1			
MCTP1			
MCTS1			
MDFIC			
MDGA2			
MDH1B			
MDH2			
MDK			
ME1			
ME2			
ME3			
MEAF6			
MECR			
MED13			
MED21			
MED23			
MED31			
MEF2C			
MEFV			
MEN1			
MEOX2			
MERTK			
MESDC2			
METAP1			
METRN			

METRNL			
METTL13			
METTL15P1			
METTL21D			
METTL22			
METTL7B			
MEX3A			
MEX3D			
MFAP1			
MFN1			
MFN2			
MFSD10			
MFSD3			
MFSD6			
MGARP			
MGST1			
MICAL2			
MICU1			
MICU3			
MID1			
MID2			
MIER3			
MIIP			
MIPEP			
MIR181A1HG			
MIR210HG			
MIR24-2			
MKI67			
MKNK1			
MKNK2			
MKRN1			
MKX			
MLC1			
MLF1			
MLF1IP			
MLF2			
MLKL			
MLXIP			
MMAB			
MMP11			
MMP14			
MOAP1			
MOB1A			
MOB1B			

MOB3A			
MOB3B			
MOBP			
MOG			
MON2			
MORC3			
MOV10			
MPC2			
MPP1			
MPP2			
MPPED1			
MPV17			
MPZL1			
MR1			
MRAS			
MRFAP1L1			
MRI1			
MROH8			
MRPL19			
MRPL37			
MRPL42			
MRPL45			
MRPL55			
MRPS18B			
MRPS21			
MRPS23			
MRPS26			
MRPS35			
MRPS36			
MRPS9			
MSANTD4			
MSI1			
MSI2			
MSN			
MSR1			
MSRB1			
MSRB3			
MSX1			
MT1G			
MT2A			
MTCH2			
MTDH			
MTF2			
MTHFD2			

MTHFD2L			
MTM1			
MTMR11			
MTMR2			
MTMR3			
MTMR4			
MTMR7			
MTPAP			
MTR			
MTX2			
MTX3			
MUC1			
MUL1			
MUM1			
MVK			
MXD1			
MYBBP1A			
MYC			
MYCBP2			
MYD88			
MYH7			
MYH9			
MYO18A			
MYO1F			
MYO5A			
MYO7A			
MYOF			
MYRF			
MYRIP			
MYT1L			
N4BP2			
N4BP3			
NAA25			
NAA40			
NAA50			
NABP1			
NABP2			
NACC2			
NADK			
NADK2			
NAE1			
NALCN			
NALCN-AS1			
NAMPT			

NAMPTL			
NANOS1			
NAP1L2			
NAP1L3			
NAPA			
NAPB			
NAPEPLD			
NAPG			
NARS			
NAT8L			
NBEA			
NBEAL1			
NBEAL2			
NBPF10			
NBPF13P			
NBPF14			
NBPF20			
NCALD			
NCAN			
NCBP1			
NCBP2			
NCBP2-AS2			
NCKAP1L			
NCOA7			
NCR3LG1			
NDEL1			
NDFIP1			
NDFIP2			
NDN			
NDRG3			
NDRG4			
NDUFA10			
NDUFA4			
NDUFA9			
NDUFAF4			
NDUFAF5			
NDUFAF6			
NDUFB2			
NDUFB9			
NDUFS1			
NDUFS2			
NDUFS3			
NDUFS4			
NEBL			

NECAB1			
NECAP1			
NECAP2			
NEFH			
NEFL			
NEGR1			
NEK3			
NEK6			
NELL1			
NELL2			
NES			
NET1			
NEU3			
NEUROD6			
NEXN			
NF1			
NFAM1			
NFASC			
NFATC1			
NFATC2			
NFATC2IP			
NFE2L2			
NFE2L3			
NFIB			
NFIL3			
NFKB2			
NFKBID			
NFKBIL1			
NFKBIZ			
NFS1			
NFU1			
NFYA			
NFYB			
NGEF			
NGFRAP1			
NHLH1			
NHLH2			
NHLRC3			
NHP2L1			
NHS			
NHSL1			
NHSL2			
NICN1			
NID1			

NIF3L1			
NINJ1			
NIPA1			
NIPA2			
NIPAL1			
NIPAL2			
NIPAL3			
NIPSNAP1			
NIT1			
NIT2			
NKIRAS1			
NLGN4Y			
NLK			
NLN			
NLRC5			
NLRP9P			
NMB			
NME5			
NMI			
NMNAT2			
NMRAL1			
NNT			
NOL10			
NOMO1			
NONOP2			
NOP58			
NOP9			
NOS1			
NOS2			
NOTCH2			
NOVA1			
NPAS3			
NPHP1			
NPHP3-AS1			
NPL			
NPM2			
NPRL2			
NPTN			
NPY5R			
NQO1			
NR2E1			
NR3C1			
NRAP			
NRBP2			

NRD1			
NREP			
NRG1			
NRIP3			
NRM			
NRSN2			
NRXN3			
NSF			
NSMCE2			
NSUN5			
NSUN5P1			
NT5C2			
NT5DC1			
NT5E			
NT5M			
NTHL1			
NTN1			
NTN3			
NTN4			
NTPCR			
NTSR2			
NUAK1			
NUCKS1			
NUDCD3			
NUDT17			
NUDT18			
NUDT2			
NUDT21			
NUFIP2			
NUP133			
NUP155			
NUP210			
NUP210L			
NUP93			
NUP98			
NUPR1			
NUSAP1			
NXN			
NXPH1			
NXPH2			
NXPH3			
NXT2			
OAF			
OAT			

OBFC1			
OCIAD1			
ODC1			
OGDH			
OGDHL			
OGG1			
OIP5-AS1			
OLA1			
OLFM2			
OLFM3			
OPA1			
OPALIN			
OPCML			
OPHN1			
OPN3			
OPTN			
OR9A2			
ORC2			
ORC3			
OSBPL10			
OSBPL11			
OSBPL1A			
OSCAR			
OSGEPL1			
OSGIN1			
OSMR			
OSTF1			
OTUB1			
OTUD4			
OTUD7A			
OTX1			
OVGP1			
OXA1L			
OXLD1			
OXR1			
P4HA1			
P4HA2			
PAAF1			
PABPC1			
PABPC1L2A			
PABPC4			
PABPN1			
PACRG			
PACSIN2			

PACSIN3			
PAFAH1B1			
PAFAH1B2			
PAICS			
PAIP2			
PAK1			
PAK7			
PALLD			
PANK1			
PAPD5			
PAPOLA			
PAPSS1			
PARD3			
PARD3B			
PARD6A			
PARD6G			
PARG			
PARK2			
PARL			
PARN			
PARP10			
PARP2			
PARP6			
PARP9			
PART1			
PARVG			
PAX6			
PBDC1			
PBX4			
PBXIP1			
PCBD2			
PCBP4			
PCDH1			
PCDH11Y			
PCDH18			
PCDHA1			
PCDHB10			
PCDHB12			
PCDHB14			
PCDHB15			
PCDHB18			
PCDHB19P			
PCDHB2			
PCDHB3			

PCDHB6			
PCGF5			
PCLO			
PCMT1			
PCOLCE2			
PCP4			
PCP4L1			
PCSK2			
PCSK4			
PCSK5			
PCYOX1L			
PDCD10			
PDCD6IP			
PDCD7			
PDE10A			
PDE1A			
PDE1C			
PDE3B			
PDE4A			
PDE4DIP			
PDE6B			
PDE8A			
PDGFC			
PDHB			
PDIA6			
PDIK1L			
PDK3			
PDLIM3			
PDLIM5			
PDLIM7			
PDP1			
PEA15			
PEBP1			
PEF1			
PEG10			
PELI3			
PEPD			
PEX11B			
PEX14			
PEX16			
PEX3			
PEX5			
PEX5L			
PFDN1			

PFDN4			
PFKM			
PFKP			
PFN2			
PGA3			
PGAM1			
PGAP1			
PGAP2			
PGBD1			
PGBD4			
PGM3			
PGP			
PGS1			
PHACTR2			
PHF11			
PHF21B			
PHKG2			
PHLDA1			
PHLDB3			
PHRF1			
PHYHIP			
PHYHIPL			
PI4K2B			
PI4KA			
PIEZO1			
PIGA			
PIGK			
PIGL			
PIGT			
PIGU			
PIGV			
PIGX			
PIGZ			
PIH1D1			
PIH1D2			
PIK3AP1			
PIK3C2B			
PIK3CB			
PIK3CG			
PIK3R1			
PIK3R4			
PIK3R5			
PIM1			
PIN1			

PINK1			
PIP4K2C			
PIP5K1A			
PIRT			
PITHD1			
PITPNC1			
PITRM1			
PJA2			
PKD1P6			
PKM			
PKN1			
PKN2			
PKNOX1			
PKNOX2			
PLA2G4C			
PLA2G6			
PLAC9			
PLAUR			
PLB1			
PLBD1			
PLBD2			
PLCB1			
PLCB3			
PLCB4			
PLCD1			
PLCD3			
PLCD4			
PLCE1			
PLCL2			
PLD3			
PLEC			
PLEK			
PLEKHA4			
PLEKHA7			
PLEKHB2			
PLEKHF2			
PLEKHG1			
PLEKHG2			
PLEKHG3			
PLEKHH1			
PLEKHM3			
PLEKHO1			
PLIN2			
PLIN4			

PLK5			
PLLP			
PLOD1			
PLOD2			
PLP1			
PLSCR1			
PLSCR4			
PLTP			
PLVAP			
PLXDC1			
PLXDC2			
PLXNA2			
PLXNB2			
PM20D2			
PMEL			
PML			
PMPCB			
PMS1			
PMS2			
PMS2P1			
PNKD			
PNMA2			
PNMA3			
PNP			
PNPLA8			
PNRC2			
PODN			
POFUT1			
POFUT2			
POLD1			
POLDIP2			
POLE2			
POLE3			
POLR2B			
POLR2J			
POLR3E			
POLR3G			
POLR3K			
POLRMT			
POMGNT1			
PON2			
POP7			
POR			
PORCN			

POSTN			
POU6F1			
PPA1			
PPA2			
PPAP2B			
PPAPDC1A			
PPAPDC3			
PPARA			
PPARG			
PPARGC1B			
PPAT			
PPCS			
PPEF1			
PPIA			
PPIAP22			
PPIH			
PPIL6			
PPIP5K2			
PPM1AP1			
PPM1H			
PPM1L			
PPME1			
PPOX			
PPP1CA			
PPP1R11			
PPP1R12B			
PPP1R13L			
PPP1R14B			
PPP1R14C			
PPP1R16A			
PPP1R18			
PPP1R2			
PPP1R21			
PPP1R26-AS1			
PPP1R3B			
PPP1R3F			
PPP1R7			
PPP1R9A			
PPP2R1A			
PPP2R1B			
PPP2R2B			
PPP2R2D			
PPP2R3B			
PPP2R5A			

PPP2R5B			
PPP2R5D			
PPP3CA			
PPP3CB			
PPP3R1			
PPP4C			
PPP4R2			
PPP5C			
PPP5D1			
PRC1			
PRCP			
PRDM4			
PRDX6			
PREP			
PREX2			
PRICKLE1			
PRICKLE3			
PRICKLE4			
PRKAB2			
PRKACB			
PRKAG2-AS1			
PRKAR1A			
PRKAR1B			
PRKAR2B			
PRKCB			
PRKCE			
PRKCQ			
PRKCQ-AS1			
PRKD1			
PRKD2			
PRKD3			
PRKG1			
PRKX			
PROB1			
PROCA1			
PROS1			
PRPF19			
PRPF3			
PRPF38A			
PRPF40A			
PRPF4B			
PRPF8			
PRPS1			
PRPS2			

PRR18			
PRR22			
PRR4			
PRR5			
PRRT3			
PRRX1			
PRSS35			
PRSS51			
PRUNE2			
PSD4			
PSEN2			
PSG3			
PSG5			
PSMA4			
PSMA5			
PSMB2			
PSMB4			
PSMB5			
PSMB8			
PSMC1			
PSMC2			
PSMC3			
PSMC4			
PSMC6			
PSMD1			
PSMD12			
PSMD3			
PSMD4			
PSMD7			
PSMG3			
PSPH			
PSTPIP1			
PTAR1			
PTBP1			
PTBP3			
PTCHD2			
PTCHD4			
PTDSS2			
PTGES2			
PTGES3			
PTH2R			
PTP4A2P1			
PTPDC1			
PTPN1			

PTPN11			
PTPN12			
PTPN13			
PTPN22			
PTPN3			
PTPN6			
PTPN9			
PTPRC			
PTPRD			
PTPRZ1			
PTTG1			
PTTG1IP			
PUS10			
PVALB			
PVRL2			
PVT1			
PWP1			
PXDC1			
PXN			
PYCR1			
PYGL			
PYGO1			
QARS			
QDPR			
QRFPR			
QRICH1			
QTRT1			
R3HCC1			
R3HDM2			
RAB11A			
RAB11FIP1			
RAB11FIP5			
RAB13			
RAB14			
RAB20			
RAB21			
RAB27A			
RAB2A			
RAB31			
RAB33A			
RAB34			
RAB37			
RAB39A			
RAB39B			

RAB3A			
RAB3GAP2			
RAB3IL1			
RAB6B			
RAB7L1			
RAB8B			
RAB9B			
RABEPK			
RABGAP1L			
RABGGTA			
RABIF			
RAD1			
RAD23A			
RALB			
RALBP1			
RALGAPA1			
RALGAPA2			
RALGPS1			
RAMP1			
RAMP2			
RAMP3			
RANBP1			
RANBP3			
RANBP3L			
RANBP6			
RANGAP1			
RAP1B			
RAP1GDS1			
RAPGEF4-AS1			
RAPGEF5			
RAPGEF6			
RARRES3			
RASA1			
RASA4CP			
RASAL2-AS1			
RASAL3			
RASGEF1B			
RASGRF1			
RASGRP1			
RASSF10			
RASSF8			
RAVER1			
RAVER2			
RB1			

RBBP4			
RBBP6			
RBFOX1			
RBM12B			
RBM3			
RBM38			
RBM39			
RBM45			
RBM47			
RBMXP4			
RC3H1			
RCAN2			
RCC1			
RCN1			
RCN3			
RCOR2			
RCOR3			
RDH10			
RDH14			
RECQL4			
REEP1			
REEP2			
REL			
RELA			
RELL2			
REPS1			
REPS2			
RERGL			
RET			
RETSAT			
REV3L			
RFC3			
RFNG			
RFPL1S			
RFX2			
RFXANK			
RGP1			
RGS14			
RGS16			
RGS19			
RGS2			
RGS20			
RGS22			
RGS4			

RGS6			
RGS7			
RHBDF1			
RHBDF2			
RHOBTB1			
RHOBTB3			
RHOC			
RHOQ			
RHOQP3			
RHPN2			
RILP			
RIMKLB			
RIMS3			
RIN1			
RIN3			
RINT1			
RIOK1			
RIPK1			
RIPK2			
RIT1			
RMND5B			
RN7SL737P			
RNASE1			
RNASEH1			
RNASET2			
RNF112			
RNF113A			
RNF114			
RNF121			
RNF122			
RNF135			
RNF14			
RNF144A			
RNF146			
RNF149			
RNF157			
RNF167			
RNF169			
RNF175			
RNF182			
RNF185			
RNF187			
RNF215			
RNF217			

RNF220			
RNF26			
RNF34			
RNF5			
RNF5P1			
RNFT2			
RNMT			
ROBO3			
ROCK1			
ROCK2			
ROGDI			
ROM1			
ROS1			
RP2			
RP9			
RPAIN			
RPF1			
RPH3A			
RPL12			
RPL12P4			
RPL17P50			
RPL18A			
RPL23			
RPL23A			
RPL23P2			
RPL24			
RPL27A			
RPL28			
RPL34			
RPL37			
RPL5P34			
RPL6			
RPLP0			
RPLP1			
RPLP2			
RPN2			
RPP25			
RPRD1B			
RPS15A			
RPS19			
RPS2			
RPS20			
RPS24			
RPS27L			

RPS3			
RPS6			
RPS6KL1			
RPS7			
RPUSD2			
RPUSD4			
RRAD			
RRAGA			
RRAGD			
RRAS			
RRAS2			
RREB1			
RSBN1L-AS1			
RSL24D1			
RSPH4A			
RSRC1			
RSU1			
RTCA			
RTCB			
RTKN2			
RTN1			
RTN3			
RTN4			
RTN4IP1			
RTN4R			
RUFY2			
RUNDC3B			
RUNX1			
RUSC1			
RWDD1			
RWDD2B			
RXFP1			
RXRA			
RYK			
RYR1			
RYR2			
S100A10			
S100A16			
S100A9			
S1PR1			
S1PR3			
S1PR5			
SAE1			
SALL3			

SAMD12			
SAMD15			
SAMD4A			
SAMD5			
SAMD8			
SAMD9L			
SAMHD1			
SAMM50			
SAMSN1			
SAP30L			
SARDH			
SARS			
SASH1			
SASH3			
SAT1			
SATB2-AS1			
SBF2			
SBNO1			
SBNO2			
SCAI			
SCAMP1			
SCAMP5			
SCAND2P			
SCARA3			
SCARB2			
SCG5			
SCIN			
SCML1			
SCML2			
SCN1B			
SCN2A			
SCN2B			
SCN4B			
SCN8A			
SCN9A			
SCOC			
SCRG1			
SCRIB			
SCRN2			
SCRT1			
SDC1			
SDC2			
SDC4			
SDF2L1			

SDF4			
SDHA			
SDHAP3			
SDHB			
SDR39U1			
SDS			
SDSL			
SEC11A			
SEC14L5			
SEC23A			
SEC24A			
SEC61A1			
SECISBP2			
SELRC1			
SEMA3A			
SEMA3B			
SEMA3G			
SEMA4B			
SEMA4C			
SEMA7A			
SENP2			
SEPN1			
SEPT14			
SEPT7P2			
SEPT9			
SERBP1			
SERGEF			
SERINC1			
SERINC3			
SERP1			
SERP2			
SERPINA1			
SERPINA3			
SERPINB9			
SERPINE1			
SERPINE2			
SERPING1			
SERPINH1			
SERPINI1			
SERTAD2			
SERTAD4			
SERTAD4-AS1			
SESN2			
SESTD1			

SETD4			
SETD5-AS1			
SETD9			
SEZ6L2			
SFMBT2			
SFXN4			
SGCD			
SGIP1			
SGK2			
SGMS2			
SGPL1			
SGSH			
SGTB			
SH2B2			
SH2B3			
SH2D4A			
SH3BGRL2			
SH3BGRL3			
SH3D19			
SH3GL2			
SH3GL3			
SH3GLB1			
SH3GLB2			
SH3RF3			
SH3RF3-AS1			
SH3TC1			
SH3TC2			
SHC1			
SHD			
SHKBP1			
SHMT2			
SHROOM4			
SIAE			
SIPA1			
SIX4			
SKIV2L			
SKP1			
SLAIN2			
SLC10A1			
SLC10A4			
SLC11A1			
SLC12A5			
SLC12A7			
SLC12A8			

SLC15A3			
SLC16A10			
SLC16A3			
SLC16A7			
SLC16A9			
SLC17A5			
SLC17A9			
SLC19A1			
SLC1A3			
SLC1A4			
SLC1A5			
SLC22A15			
SLC22A17			
SLC22A24			
SLC22A3			
SLC24A2			
SLC24A6			
SLC25A11			
SLC25A12			
SLC25A14			
SLC25A15			
SLC25A17			
SLC25A20			
SLC25A27			
SLC25A3			
SLC25A30			
SLC25A33			
SLC25A36			
SLC25A39			
SLC25A4			
SLC25A40			
SLC25A41			
SLC25A46			
SLC25A5			
SLC25A53			
SLC26A11			
SLC27A4			
SLC29A1			
SLC29A4			
SLC2A10			
SLC2A11			
SLC2A12			
SLC2A4			
SLC2A4RG			

SLC2A5			
SLC2A8			
SLC2A9			
SLC30A1			
SLC30A10			
SLC30A5			
SLC30A7			
SLC30A9			
SLC32A1			
SLC35B3			
SLC35B4			
SLC35C2			
SLC35F3			
SLC35F5			
SLC35G1			
SLC36A1			
SLC36A4			
SLC38A1			
SLC38A11			
SLC38A5			
SLC39A1			
SLC39A10			
SLC39A12			
SLC39A7			
SLC3A2			
SLC41A1			
SLC43A1			
SLC43A3			
SLC44A1			
SLC44A3			
SLC45A1			
SLC4A10			
SLC4A11			
SLC4A4			
SLC4A8			
SLC50A1			
SLC5A11			
SLC5A2			
SLC6A11			
SLC6A15			
SLC6A17			
SLC6A6			
SLC6A7			
SLC7A11			

SLC7A11-AS1			
SLC7A2			
SLC7A5			
SLC7A8			
SLC8A1			
SLC8A1-AS1			
SLC9A6			
SLC9A9			
SLC9B2			
SLCO1A2			
SLFN11			
SLFNL1			
SLITRK1			
SLITRK3			
SLMO2			
SLU7			
SLX4IP			
SMAD1			
SMAD4			
SMAD5			
SMAD9			
SMAP2			
SMARCA1			
SMARCE1P5			
SMC3			
SMC5			
SMEK1			
SMEK2			
SMG1			
SMG6			
SMIM10			
SMIM13			
SMIM14			
SMIM15			
SMIM19			
SMIM20			
SMIM4			
SMNDC1			
SMO			
SMOX			
SMPX			
SMTN			
SMTNL1			
SMUG1			

SMURF2			
SMYD2			
SNAP23			
SNAP91			
SNCA			
SNCB			
SNHG11			
SNHG12			
SNHG16			
SNHG5			
SNN			
SNRK			
SNRNP25			
SNRPA			
SNRPC			
SNRPG			
SNRPN			
SNTA1			
SNTG1			
SNURF			
SNX10			
SNX14			
SNX17			
SNX18			
SNX20			
SNX22			
SNX30			
SNX4			
SNX5			
SOAT1			
SOCS3			
SOCS4			
SOCS5			
SOD2			
SOD3			
SORL1			
SOWAHB			
SOWAHC			
SOX4			
SOX6			
SP100			
SP110			
SPAG6			
SPAG7			

SPATA1			
SPATA17			
SPATA7			
SPATS2L			
SPDEF			
SPG20			
SPHK1			
SPHK2			
SPI1			
SPIN2A			
SPIN2B			
SPIN3			
SPINT1			
SPINT2			
SPOCD1			
SPOCK2			
SPOCK3			
SPON1			
SPPL2A			
SPR			
SPRED1			
SPRY1			
SPRYD3			
SPRYD7			
SPSB2			
SPTLC3			
SPTSSB			
SPTY2D1			
SQRDL			
SQSTM1			
SRCIN1			
SRCRB4D			
SRD5A1			
SRD5A3			
SREK1			
SRF			
SRGAP1			
SRGAP3			
SRGN			
SRI			
SRL			
SRP19			
SRPK2			
SRRM1			

SRRM4			
SRRT			
SRSF10			
SRSF11			
SS18			
SSBP3			
SSFA2			
SSR3			
SSSCA1-AS1			
SST			
SSX2IP			
ST13			
ST13P5			
ST13P6			
ST14			
ST18			
ST3GAL5			
ST5			
ST6GALNAC1			
ST8SIA1			
STAG3			
STAG3L4			
STAM			
STAMBPL1			
STARD4-AS1			
STARD8			
STARD9			
STAT4			
STAU2			
STEAP3			
STIP1			
STK16			
STK17A			
STK17B			
STK38			
STK38L			
STK39			
STMN1			
STMN2			
STMN3			
STOM			
STON2			
STPG1			
STRA6			

STRBP			
STRIP1			
STRN			
STS			
STX12			
STX1A			
STX1B			
STX3			
STXBP1			
STXBP2			
STXBP4			
STXBP5			
STXBP5-AS1			
STXBP5L			
STYX			
STYXL1			
SUCLA2			
SUCLG1			
SUCLG2			
SUCO			
SUDS3			
SUGP2			
SUGT1			
SUMO1			
SUMO3			
SUPT16H			
SUPT20H			
SUPT5H			
SUPV3L1			
SUSD4			
SUSD5			
SUV39H1			
SV2A			
SV2B			
SVEP1			
SVOP			
SWAP70			
SWT1			
SYBU			
SYDE1			
SYNC			
SYNDIG1			
SYNGR1			
SYNGR3			

SYNJ1			
SYNJ2			
SYNPO2			
SYP			
SYPL2			
SYT1			
SYT11			
SYT12			
SYT13			
SYT16			
SYT17			
SYT2			
SYT3			
SYT4			
TAB1			
TAB2			
TACR3			
TADA1			
TADA2A			
TADA2B			
TAF4B			
TAGLN2			
TAGLN3			
TARBP1			
TASP1			
TATDN2			
TBC1D10A			
TBC1D10B			
TBC1D22B			
TBC1D25			
TBC1D30			
TBC1D4			
TBC1D7			
TBC1D9			
TBCC			
TBK1			
TBL1X			
TBP			
TBR1			
TBRG1			
TBX15			
TCAP			
TCEA1			
TCEA3			

TCEAL1			
TCEAL7			
TCEB3			
TCERG1			
TCF12			
TCF3			
TCIRG1			
TCTEX1D1			
TDP2			
TDRKH			
TEAD1			
TEAD3			
TECPR2			
TECR			
TEP1			
TERF2IP			
TES			
TESC			
TESPA1			
TET2			
TEX14			
TEX15			
TF			
TFB1M			
TFE3			
TG			
TGFB1			
TGFB2			
TGFBRAP1			
TGIF1			
TGM2			
THAP1			
THAP2			
THAP7			
THBD			
THBS1			
THBS4			
THEM6			
THEMIS			
THOC5			
THYN1			
TICRR			
TIFA			
TIGD4			

TIMM17A			
TIMM17B			
TIMP2			
TIPARP			
TIPRL			
TKT			
TLCD1			
TLE1			
TLE3			
TLN1			
TLR2			
TLR4			
TLR5			
TM2D3			
TM4SF1			
TM6SF1			
TM7SF3			
TMA16			
TMBIM6			
TMC5			
TMC7			
TMC8			
TMCO1			
TMCO4			
TMED10			
TMED3			
TMED5			
TMEFF2			
TMEM100			
TMEM120A			
TMEM125			
TMEM133			
TMEM144			
TMEM14A			
TMEM150C			
TMEM151A			
TMEM155			
TMEM160			
TMEM161A			
TMEM164			
TMEM167B			
TMEM169			
TMEM17			
TMEM175			

TMEM176A			
TMEM176B			
TMEM177			
TMEM178A			
TMEM183A			
TMEM184A			
TMEM185B			
TMEM191C			
TMEM194B			
TMEM203			
TMEM235			
TMEM240			
TMEM25			
TMEM255A			
TMEM26			
TMEM30A			
TMEM38A			
TMEM43			
TMEM44-AS1			
TMEM47			
TMEM51			
TMEM55A			
TMEM55B			
TMEM63A			
TMEM63B			
TMEM72-AS1			
TMEM88B			
TMEM8A			
TMOD3			
TMSB4Y			
TMTC4			
TMUB1			
TMX1			
TMX4			
TNFAIP3			
TNFAIP8			
TNFRSF10B			
TNFRSF10D			
TNFRSF11B			
TNFRSF1A			
TNFRSF1B			
TNFRSF21			
TNFSF10			
TNFSF13B			

TNIP1			
TNIP2			
TNIP3			
TNKS1BP1			
TNNT2			
TNPO1			
TOB1			
TOMM20			
TOMM40			
TOMM40L			
TOMM70A			
TOP1			
TOP2B			
TOPBP1			
TOPORS			
TOR1A			
TOR1AIP2			
TOR1B			
TOR4A			
TOX2			
TP53			
TPBG			
TPD52L1			
TPD52L2			
TPI1			
TPM2			
TPP1			
TPP2			
TPRG1			
TPST1			
TPT1			
TRA2B			
TRABD			
TRAF3IP2			
TRAIP			
TRAM1L1			
TRAPPC10			
TRAPPC11			
TRAPPC13			
TRAPPC2L			
TRAPPC3			
TRAPPC6B			
TRAPPC9			
TRBC2			

TRHDE			
TRIM14			
TRIM2			
TRIM23			
TRIM25			
TRIM27			
TRIM3			
TRIM33			
TRIM36			
TRIM37			
TRIM4			
TRIM44			
TRIM5			
TRIM59			
TRIM60P18			
TRIM66			
TRIM69			
TRIM71			
TRIM72			
TRIM73			
TRIO			
TRIP10			
TRIP13			
TRIP6			
TRMT11			
TRO			
TROVE2			
TRPC1			
TRPC3			
TRPC4AP			
TRPS1			
TRUB1			
TRUB2			
TSC1			
TSC2			
TSEN34			
TSFM			
TSHR			
TSPAN12			
TSPAN15			
TSPAN33			
TSPAN4			
TSPAN5			
TSPAN6			

TSPYL5			
TSR2			
TSTD2			
TTBK2			
TTC1			
TTC13			
TTC19			
TTC21B			
TTC22			
TTC23			
TTC28			
TTC37			
TTC38			
TTC39B			
TTC9C			
TTF1			
TTL			
TTLL12			
TTLL7			
TTPA			
TTTY10			
TTTY15			
TTYH2			
TUBA1A			
TUBA4A			
TUBB			
TUBB1			
TUBB2A			
TUBB2B			
TUBB4A			
TUBB4B			
TUBB6			
TUBBP1			
TUBG1			
TUBG2			
TUBGCP3			
TUBGCP4			
TUBGCP5			
TUBGCP6			
TUG1			
TULP4			
TUSC2			
TUSC3			
TXLNA			

TXLNG2P			
TXNDC16			
TXNDC9			
TXNIP			
TXNL4B			
TYK2			
TYMP			
TYMS			
TYRO3			
TYSND1			
TYW1B			
UBA3			
UBAP1L			
UBAP2			
UBE2E3			
UBE2FP3			
UBE2H			
UBE2I			
UBE2J2			
UBE2L6			
UBE2N			
UBE2QL1			
UBE2T			
UBE2V2			
UBE3C			
UBL4A			
UBL7			
UBLCP1			
UBR3			
UBTD1			
UBXN4			
UCHL1			
UCHL5			
UCP2			
UFSP2			
UGDH			
UGGT1			
UGP2			
UGT8			
UHRF1BP1L			
UHRF2			
ULK3			
UNC13A			
UNC13D			

UNC50			
UNC5C			
UNC79			
UNC80			
UPF1			
UPP1			
UQCC			
UQCRC1			
UQCRC2			
UQCRFS1			
UQCRH			
URM1			
UROD			
UROS			
USF1			
USHBP1			
USO1			
USP13			
USP14			
USP19			
USP32			
USP33			
USP37			
USP38			
USP39			
USP42			
USP48			
USP49			
USP5			
USP53			
UST			
UTY			
UVRAG			
VAMP1			
VAMP5			
VAMP8			
VAPB			
VASP			
VCPIP1			
VDAC1			
VDAC1P1			
VDAC2			
VDAC3			
VEGFA			

VGLL4			
VHL			
VILL			
VIM			
VIMP			
VIP			
VIPR1			
VNN2			
VPS11			
VPS16			
VPS25			
VPS29			
VPS33B			
VPS35			
VPS36			
VPS37B			
VPS41			
VPS45			
VPS4B			
VPS52			
VRK1			
VSIG10			
VSIG4			
VSNL1			
VSTM5			
VTA1			
VWA2			
VWA3B			
VWC2			
WAC-AS1			
WARS2			
WAS			
WASH3P			
WBP1L			
WBP4			
WBP5			
WBSCR17			
WDFY3-AS2			
WDR1			
WDR12			
WDR17			
WDR36			
WDR47			
WDR49			

WDR52			
WDR54			
WDR62			
WDR7			
WDR70			
WDR73			
WDR75			
WDR77			
WDR78			
WDR91			
WDR92			
WDR96			
WDYHV1			
WEE1			
WEE2-AS1			
WFDC1			
WFS1			
WIBG			
WIZ			
WLS			
WNT7B			
WNT9B			
WSB1			
WSCD1			
WWC1			
WWTR1			
XBP1			
XPC			
XPO1			
XPO6			
XPOT			
XRCC1			
XRCC6			
XRN2			
YAP1			
YBEY			
YBX1			
YBX1P1			
YBX1P10			
YBX3			
YEATS4			
YIPF6			
YPEL2			
YPEL3			

YWHAB			
ZBBX			
ZBED5			
ZBTB1			
ZBTB24			
ZBTB3			
ZBTB33			
ZBTB42			
ZBTB43			
ZC2HC1A			
ZC3H12A			
ZC3H12C			
ZC3H6			
ZC3HAV1			
ZC4H2			
ZCCHC11			
ZCCHC2			
ZCWPW2			
ZDHHC15			
ZDHHC17			
ZDHHC18			
ZEB1			
ZEB1-AS1			
ZFAND2B			
ZFAND3			
ZFAND6			
ZFAS1			
ZFC3H1			
ZFP14			
ZFP2			
ZFP36			
ZFP36L1			
ZFR			
ZFY			
ZFYVE16			
ZFYVE27			
ZFYVE9			
ZGLP1			
ZIC5			
ZMAT2			
ZMAT4			
ZMPSTE24			
ZMYM1			
ZMYM2			

ZNF114			
ZNF117			
ZNF133			
ZNF14			
ZNF143			
ZNF157			
ZNF175			
ZNF18			
ZNF180			
ZNF217			
ZNF248			
ZNF25			
ZNF251			
ZNF254			
ZNF271			
ZNF28			
ZNF287			
ZNF316			
ZNF329			
ZNF33A			
ZNF33BP1			
ZNF347			
ZNF365			
ZNF366			
ZNF37BP			
ZNF385B			
ZNF385D			
ZNF398			
ZNF408			
ZNF426			
ZNF445			
ZNF449			
ZNF451			
ZNF460			
ZNF480			
ZNF516			
ZNF517			
ZNF518B			
ZNF536			
ZNF540			
ZNF554			
ZNF555			
ZNF568			
ZNF579			

ZNF586			
ZNF589			
ZNF607			
ZNF658			
ZNF674-AS1			
ZNF680			
ZNF701			
ZNF711			
ZNF740			
ZNF761			
ZNF789			
ZNF804B			
ZNF813			
ZNF815P			
ZNF826P			
ZNF835			
ZNF850			
ZNF862			
ZNRF3			
ZRANB1			
ZRANB2			
ZRANB2-AS2			
ZSCAN21			
ZSCAN31			
ZYG11B			

Supplementary Table S6. Previously published BPA transcriptome studies obtained from NCBI GEO DataSets that were used in the data-mining analysis of BPA-responsive genes.

Title	Author and Year	Sample Size	Sample Type
Transcriptomal profiling of C57BL/6 wild type and ER-alpha KO mice fetal mammary glands after fetal exposure to bisphenol A (BPA) and 17alpha-ethynylestradiol (EE2) (GSE44387)	Wadia et al., 2013	36 samples	Mouse mammary gland tissues
Toxicogenomic analysis of placenta samples from mice exposed to different doses of BPA (GSE63852)	Sabrina et al., 2014	11 samples	Mouse placental tissues
Transgenerational Gene Expression Changes Caused by Exposing Fetal Germ Cells to Endocrine Disruptors (GSE58642)	Piroska et al., 2015	32 samples	Mouse fetal germ cells
Transcriptome of human osteosarcoma (HOS) cells induced by bisphenol A, S and AF (GSE50527)	Fic et al., 2015	24 samples	HOS cell line
Effects of bisphenol A on gene expression in adipocytes from lean children: Association with metabolic disorders (GSE58516)	Menale et al., 2015	15 samples	Primary human adipocytes
Preferential epigenetic programming of the estrogen response after in utero xenoestrogen (bisphenol A) exposure (GSE86923)	Jorgensen et al., 2016	10 samples	Mouse uterine tissues

Supplementary Table S7. The list of differentially expressed genes in response to BPA identified by the reanalysis of previously published transcriptome profiling studies in NCBI GEO DataSets database.

GSE44387	GSE63852	GSE58642	GSE50527	GSE58516	GSE86923
AA407107	AAAS	Akr1cl	AATBC	AIM2	AAAS
AACS	ABCA4	Cd200r3	ABCA3	BRF2	AAGAB
ABCB9	ABHD14A	CLIC3	ABHD1	C7orf72	AARD
ABCC5	ABL2	CSGALNACT1	ABRA	DHRS1	AARS
ABHD17A	ACADM	DMRTA1	ABT1	FAM134C	AARSD1
ABL1	ACADSB	DOK7	ABTB1	FAM193B	AATF
ABTB1	ACSL6	FCER2	ACOXL	FOXO4	ABCA7
ACAA1	ACSS3	FFAR3	ACP1	GOLGA8DP	ABCB8
ACAA2	ACTA2	FRYL	ACSBG2	GOLGA8EP	ABCC10
ACACB	ADCY5	Gm10152	ACSM3	GOLGA8G	ABCD3
ACADM	ADD3	GPR15	ACSM5	HMOX2	ABCE1
ACADS	ADH1C	HS3ST3A1	ACSS2	KLHL36	ABCF2
ACLY	ADORA2B	ITGB7	ADAM28	LHX6	ABHD14B
ACOT9	ADRA1D	JAK3	ADAMTS17	MS4A6A	ABHD16A
ACSL1	AFDN	LGR5	ADAMTS7P1	NARFL	ABHD6
ACVR1	AGA	LPL	ADCY2	OR1B1	ACAA2
ADAL	AGXT2	mir-23	ADD3	OR51I1	ACAD9
ADAM17	Aia1	Olfr600	ADGRE3	PDDC1	ACADM
ADAMTS4	AIFM1	OR10G2	ADGRE4P	PEX26	ACADVL
ADHFE1	AKR1D1P1	PNMT	ADNP2	PIGH	ACER3
ADTRP	ALCAM	PPP1R3A	ADORA1	PPOX	ACOX3
AGL	ALDH18A1	RNF213	AFAP1-AS1	R3HCC1	ACOXL
AGPAT3	Aldh5	Sct	AFDN-AS1	RAB9BP1	ACP2
AI449212	ALDH5A1	SEP11	AGAP1	RPL13AP17	ACTA1
Airn	Aldr5	SERPINA11	AGAP3	RPL29P30	ACTB
AK1	ALOX12B	Serpina3b	AGK	RPS10P7	ACTN4
AKR1A1	AMT	Serpina3j	AGL	SIDT1	ACTR2
ALAS1	APBB3	SH3YL1	AHCTF1	SLAMF1	ACY1
ALDH16A1	APOC4	SP6	AHNAK2	SLC25A39	ADA
ALDOA	AQP7	SPOCK3	AIM1L	SNHG16	Adam26a
ALG8	ARFGAP3	TMEM82	AJAP1	SPATA16	Adam26b
ANAPC5	ARHGAP5	TSSK1B	AKAP1	STARD7-AS1	ADAMTSL3
ANKRD13D	ARID3A	Ube2d4	AKIRIN2	STRADA	ADAMTSL5
ANKRD17	ARL6IP1	UMODL1	AKR1C4	SYTL4	ADD1
ANKRD63	ASB9	ZAR1	AKR7L	TAS2R41	ADGRG2
Anp32a	ASCC3		ALAD	TLR10	Adh6a
ANXA8	ASGR1		ALDH1A2	TMEM185A	ADK
ANXA8L1	ASL		ALG12	ZNF212	ADM
Ap2b1	ASS1P5		ALKBH6	ZNF346	ADORA3

AP3M2	ASTL		ALOXE3	ZNF350	ADPRH
APCDD1	ATF1		AMN	ZNF419	ADRB2
APMAP	ATF6B		ANKH	ZNF821	ADSSL1
APOE	ATP2B3		ANKRD20A11F	ZSCAN25	AGFG2
APOO	ATRN		ANKRD20A8P		AGGF1
APOPT1	ATXN2		ANKRD30A		AGO2
ARAP1	AVPR1A		ANKRD37		AGPAT2
ARAP2	B4GAT1		ANXA8		AGTR1
ARHGEF25	BAG4		ANXA8L1		AHCTF1
ARID5B	BAIAP3		APLP1		AHCYL2
ARIH2	BBOX1		APOB		AHI1
ARL13B	BHLHA15		APOL4		AHNAK2
ARL4A	BLMH		APOOL		AHSA1
ARNT	BMP3		ARF3		AI413582
ARPC3	BMPR1B		ARHGAP11B		AIDA
ARSG	Bmyc		ARHGAP25		AIF1
ARX	BNIP3L		ARHGAP8		AIF1L
ASL	BUB1B		ARL2BP		AIM1L
ASPA	BUD31		ARMC10P1		AIP
ASTN1	C19orf53		ARMC6		AKAP1
ATG101	C1QBP		ARRDC2		AKAP8
ATG9A	C21orf2		ART4		Akap9
ATL3	CACFD1		ASB11		AKR1A1
ATP5J2-PTCD1	CAD		ASB12		AKR1B1
ATP6V0A1	CALB1		ASB16		Akr1c12
ATXN2	CALR		ASH1L-AS1		Akr1c13
B230214N19Rik	CANX		ASTN2-AS1		AKR1C3
B230307C23Rik	CAPSL		ATAD1		AKT3
B330016D10Rik	CCDC43		ATF4P4		ALCAM
BAD	CD34		ATL1		ALG5
BAIAP2	CD40LG		ATP1A4		ALKBH6
BARD1	CD7		ATP2A2		ALKBH7
BC026513	CDC42EP3		ATP5F1		ALKBH8
BCL2L2	CDH15		ATP6V0D1		Alox12e
BCL6	CDK20		ATP6V1E1		ALOX5
BCL9L	CDK6		ATP6V1G3		Alyref
BFAR	CDK8		AUP1		AMBRA1
BIRC6	CDS2		AXDND1		AMD1
BORCS8	CECR7		AZGP1P1		AMDHD2
BPGM	CLSTN2		B4GALNT3		AMOTL1
BPNT1	CLTA		B4GAT1		ANAPC5
BRF1	CLTC		BAAT		ANG
BTNL9	CMTR1		BAGE5		ANKH
BZW2	CNEP1R1		BCL2		ANKHD1

C030011I16Rik	CNGA3		BCL2L13		ANKHD1-EIF4EBP
C10orf11	CNPY2		BCL3		ANKMY2
C11orf54	COCH		BCLAF1		ANKRD11
C130075A20Rik	COL5A2		BCOR		ANKRD22
C18orf8	CPNE6		BCR		ANKS6
C19orf44	CREB5		BDNF-AS		Anp32b-ps1
C19orf53	CRHBP		BEND6		ANTXR2
C19orf70	Crisp3		BEX2		ANXA4
C1D	Crisp1		BEX4		ANXA7
C1orf123	CRX		BICDL2		AOC1
C22orf23	CSF2RA		BIN3		AP1S1
C2orf68	CTNNBL1		BMF		AP4M1
C3orf14	DCTN1		BMP1		AP5B1
C430003N24Rik	DDX21		BOD1		APBB1IP
C5orf30	DDX6		BRD7		APEH
C5orf63	Defa11		BTBD8		APOBEC1
C77438	Defa6		BZW1P2		APOBR
C9orf85	Defa-rs9		C10orf107		APOL6
CA11	DENR		C10orf53		APOPT1
CA5B	DIAPH1		C11orf65		APPL1
CASKIN2	DMP1		C11orf70		AQP9
CASP3	DNAJA1		C11orf95		AQR
CCDC107	DPCD		C12orf57		ARF3
CCDC167	EDF1		C12orf77		ARF5
CCDC47	EIF2A		C14orf132		ARFGEF3
CCDC81	EIF2B3		C16orf52		ARFIP2
CCDC9	EIF2S1		C16orf54		ARG1
CCND2	EIF3M		C18orf25		ARHGAP15
CCNJ	EIF5		C1orf147		ARHGAP30
Cd59a	EIF6		C1orf43		ARHGAP45
CD93	ELAVL3		C1orf53		ARHGAP6
CDC37	EPC1		C1QTNF1		ARHGDIB
CDC42BPG	ERBB4		C1R		ARHGEF6
CDHR4	ERCC4		C2orf70		ARID1B
CDIP1	ETHE1		C2orf81		ARID2
CDK5RAP2	EXO1		C3		ARL11
CDKL3	EXOSC2		C3orf35		ARL4C
CDKN1A	EYA3		C4orf3		ARL6IP1
CDS2	FAM20B		C6orf48		ARMC6
CEP55	FASLG		C8orf76		ARMCX3
CEP68	FBXL12		C9orf50		ARPC3
CES1	FDFT1		CA8		ARPC5
CFLAR	FER		CACNA1C-AS4		ARRDC1
CFP	FGFR2		CACNA1G		ARSA

CHID1	FH		CACNG2		ARSG
CHPF	FLT4		CACNG6		ARV1
CHPT1	FLVCR1		CACTIN		ASB1
CHRDL1	FOXN1		CACYBPP2		ASB6
CHRNA4	FTSJ1		CAMK1G		ASCC3
CHST4	G3BP2		CAMK2B		ASH1L
CIAO1	G6PD		CAMKV		ASNS
CIC	GALR3		CAP2		ASPH
CLEC16A	GATA2		CAPN7		ASXL1
CLEC6A	GGCT		CAPNS2		ATF4
CLSPN	GGPS1		CAPZB		ATF5
CMIP	GGT5		CARHSP1		ATG2B
CMPK2	GIF		CASC10		ATG9B
CMTM7	Glud-ps		CASK		ATIC
COA7	Glycam1		CAT		ATL3
COG2	GNAQ		CATSPER2		ATOX1
COG6	GOT1		CBLN3		ATP13A1
COPE	GPC1		CBX7		ATP2A1
CPTP	GPD1		CCDC103		ATP2A3
CRIM1	GPR19		CCDC113		ATP2B2
CRLS1	GRP		CCDC114		ATP5F1
CRTC1	GSTT1		CCDC115		ATP6AP1
CSAD	GTF2A1		CCDC153		ATP6AP2
CSDE1	GUCA2B		CCDC167		ATP6V1B2
CSF2RB	H32		CCDC191		ATP6V1C1
CSPG4	HEXIM1		CCDC78		ATP6V1D
CSTF1	HIPK2		CCDC85A		ATP6V1E1
CSTF2T	HNRNPA3P1		CCER2		ATXN1
CTBS	HNRNPC		CCL14		ATXN1L
CTSB	Hom1		CCL2		ATXN3
CTSD	HOMER3		CCL25		AUH
CTSZ	HOXB8		CCR1		AVEN
CUEDC1	HSPA9		CCR2		AVL9
CWC15	HUS1		CCT2		B2M
CWF19L2	Hya		CCT8L2		B3GALNT1
CX3CL1	IDH3G		CCZ1		B3GALNT2
CYB5B	IFIT3		CCZ1B		B4GALT3
CYHR1	IFITM3		CD1D		BABAM1
CYP1B1	IGHV1-69		CD207		BACE1
CYP2J2	IGHV3-33-2		CD28		BAG4
CYSRT1	IGHV3OR16-7		CD79B		BAIAP3
D630033A02Rik	IGKV1-5		CD99L2		BAP1
D630045M09Rik	IGKV2D-23		CDC25B		BBS7
DAPP1	IGLV1-36		CDC25C		BC085271

DAXX	IL17C		CDC37P1		BCAP29
DCAF5	IL17RA		CDC42BPB		BCCIP
DCAF7	IL1RAPL1		CDC6		BCKDHB
DCTN2	IL27RA		CDK15		BCL2L11
DCTN3	ING4		CDK5R1		BCL2L13
DDHD1	INHBA		CDK7		BCL2L15
DDX27	INHBB		CDKL2		BCL6
DDX41	Ins1		CDV3		BCS1L
DDX46	IVD		CEACAM21		BEX3
DDX60	Kap		CEACAM3		BEX4
DECR1	KBTBD2		CELF1		BHLHA15
DEDD	KCNA2		CERS2		BLCAP
DENND2A	KCNN3		CERS6		BMP1
DENND2D	KCNQ4		CFAP97		BMS1
DERL1	KDM5D		CFH		Bmyc
DGAT1	KHNYN		CGB1		BNC2
DHDH	KLF6		CGB2		BOD1L1
DHRS7	KLHL21		CHCHD10		BPNT1
DHX30	LIPT2		CHD3		Brip1os
DHX36	LMO7		CHD7		BRSK1
DISP1	LMX1B		CHKA		BTAF1
DLAT	LSM5		CHM		BTBD3
DMTF1	LUM		CHN1		BTD
DNAAF1	MAGEA1		CHORDC1		BTK
DNAJA3	MAP1B		CHRM2		BTRC
DNAJC17	MAP3K4		CHST4		BZW2
DNASE2	MAX		CHST9		C11orf98
DNM2	MBD2		CIDEC		C12orf10
DPH1	MIPEP		CLDN25		C12orf49
DR1	MITF		CLINT1		C14orf1
DSTYK	MLLT11		CNBD1		C16orf72
DTX4	MMEL1		CNN2		C18orf8
DUS1L	MOGS		CNNM2		C19orf53
DUSP28	MPV17L2		CNTN4		C1orf159
DYRK2	MRPL3		COA3		C1orf174
EAPP	MRPL38		COBLL1		C1orf198
EBPL	MSL3		COL5A1		C1orf54
ECHDC2	MSX2		COL9A1		C1QB
ECI1	MT-CYB		COLQ		C1QC
ECSIT	MTMR2		COMMD7		C1QTNF3
EDNRA	MTR		COX7A1		C1QTNF5
EEF2K	MUC3A		COX8A		C20orf24
EGLN2	MVK		CPNE7		C21orf33
Ehbp1l1	MYLIP		CRAMP1		C2CD3

EHD2	MYO9B		CROT		C2CD4A
EIF4A3	NBR1		CRYGN		C3
EIF4EBP2	NEURL1B		CSE1L		C3orf14
EIF4G1	NF1		CSF3		C4bp
EMC4	NFKBIB		CSTF1		C5orf22
EMC9	NIT1		CSTF3		C5orf24
EPB41L2	Nmyc2		CT55		C5orf51
ETF1	Nova2-rs2		CTAG1A		C6orf120
ETFA	NQO1		CTAG1B		C6orf62
ETFB	NUBP1		CTDSPL2		C8orf44-SGK3
ETNK1	NUP85		CTRB1		C9orf85
ETS2	OASL		CUX1		CA10
EXOSC10	OCIAD1		CWH43		CA13
EZH1	OPA3		CXCL1		CACNA1A
FADS1	OPRK1		CXCL17		CACNA1G
FAM105A	OR2A7		CXCL2		CACNB2
FAM114A2	OR2A4		CXCL8		CACNB3
FAM126B	OTX1		CYP2T1P		CACTIN
FAM131A	PAN2		CYP3A7		CACYBP
FAM149B1	PCMT1		CYP7A1		CADPS2
FAM168A	Pcnt		CYTH1		CALCOCO1
FAM193A	PDE3B		DACT1		Cald1
FAM219A	PDE7B		DAZ1		CALU
FAM50A	PDLIM5		DAZ4		CAMKK2
FAM63A	PEPD		DBIL5P		CAPZB
FAM71B	PFDN4		DCAF12L1		CARHSP1
FARSA	PHKA2		DCDC5		CASK
FASTK	PHYH		DCT		CASKIN1
FBXL14	PIAS1		DCUN1D5		CAT
FBXL7	PIN1		DDN		CAV1
FBXW5	PIP5K1C		DEFA1		CBFA2T3
FERMT2	PLA2R1		DEFB121		CBLN2
FEZF1	PLEC		DEGS1		CBX3
FGF14	PLK2		DEXI		CCDC106
FICD	Plk-ps1		DGAT1		CCDC137
FKBP11	PLS1		DHX35		CCDC186
FKBP15	PLTP		DIO1		CCDC3
FLAD1	PLXNA3		DIRAS2		CCDC59
FLII	PNOC		DISP1		CCDC6
FN3KRP	POLA2		DKFZp686K1684		Ccl6
FNBP1	POM121L9P		DKK1		Ccl7
FNBP4	POU3F3		DKK2		Ccl8
FNDC5	PPEF2		DLGAP1-AS1		CCND1
FNIP1	PPM1E		DLL3		CCND2

FOXM1	PRKAG1		DMD		CCNH
FUOM	PRKCI		DMP1		CCNL1
FZD1	PRKDC		DNAAF5		CCPG1
G6pd2	PRPF18		DNAH12		CD177
GABRB2	PSMA3		DNAJA3		CD180
GAD2	PSMB6		DNAJC5B		Cd209f
GALM	PSPN		DNASE2B		Cd33
GALNT2	PTGER1		DNHD1		CD47
GATB	PTPN7		DOCK2		Cd52
GCN1	RAD17		DOCK9-AS2		CD84
GFI1	RALGAPB		DPCD		CD86
GFPT1	RALGDS		DPM1		CDC23
GHITM	RAX		DPM3		CDC25B
GHR	RFXANK		DPPA2P3		CDC42SE1
GLT8D2	RGN		E2F1		CDC5L
GLTSCR1	RNF41		EBI3		CDCP1
GLTSCR2	RPL13		ECD		CDH3
GLUD1	RPL22		ECH1		CDK2
Gm10010	RPN1		EFEMP1		CDK2AP2
Gm12503	RPS15A		EFL1		CDK4
Gm15821	RRH		EGLN2		CDK5RAP1
Gm16845	S100A3		EHD2		CDT1
Gm31765	SCP2		EIF1AX		CEBPE
Gm33002	SCTR		EIF3CL		CELF2
Gm38785	SDCBP		EIF3M		CENPB
Gm41105	SERPINI1		EIF4E		CEP295
Gm41441	SLC10A1		EIF4ENIF1		CES1
Gm42151	SLC11A2		ELAVL2		CFB
GNA12	SLC12A4		ELF1		CFI
GOLGA3	SLC13A2		ELK4		CFP
GON4L	SLC16A4		ELMO1		CHCHD6
GPD2	SLC25A30		ELOCP3		CHD2
GPN1	SLC35B2		EMC3		CHD6
GPN2	SLCO3A1		EMC6		CHD7
GPR146	SLIT1		EN2		CHD9
GPRC5B	SLN		ENPP1		CHFR
GPRIN3	SMAD4		ENTPD1-AS1		CHI3L1
GPT2	SMTN		EP400NL		CHKA
GRAMD1B	SMYD3		EPB41L4B		CHMP2A
GRB10	SORD		EPC2		CHST15
GSN	SOX21		EPSTI1		Chtop
GSS	SPO11		EPX		CISD1
GSTT2	SPP1		ERC1		CKB
GSTT2B	SRD5A1		ERCC4		CLCA1

GTF2H5	STAT4		ERICH2		Clca3b
GTF3A	STX6		ERLEC1		CLDN10
GZF1	STXBP1		ESP33		CLDN12
H2AFJ	SUCLG1		ESR2		CLDN15
H2afy3	SULT1C4		ETFB		CLDN2
H2-T22	SUN2		EVPLL		CLDN4
HADHB	SYN1		EXD3		Clec2d
HAUS1	TAZ		EXOSC1		Clec4a3
HAUS7	TBC1D9B		EYA2		CLEC7A
HCAR1	TBCB		FAAH2		CLIP1
HDAC4	Tcp1-ps1		FAIM		CLN3
HEATR6	TCTE1		FAM106A		CLPP
HGS	TEKT1		FAM110B		CLTC
HIBCH	TIMM13		FAM120B		CLUAP1
HINT2	TIMM9		FAM127A		CLUH
HIP1	TLR4		FAM127B		CMA1
HIPK1	TMSB4XP8		FAM138A		CMPK1
HLA-A	TOP3B		FAM151A		CMSS1
HMG20A	TPI1		FAM168A		CMTR2
HOXB3	TPK1		FAM182B		CNDP1
HOXB4	TRAJ33		FAM189A1		CNN2
HSD17B4	TRAV2		FAM221A		CNOT4
HSD17B8	TRB		FAM27E5		CNOT6L
HSPA4L	TRBJ2-2		FAM41C		CNST
HSPBP1	TRBV20OR9-2		FAM69B		CNTN2
ICOS	TRDD3		FAM69C		CNTN4
IDUA	TRGV10		FAM74A1		Coa6
Ifi47	TRIM27		FAM86B3P		COASY
IFIT2	TRIM35		FAM90A26		COG3
IFT81	TRPC7		FAP		COL4A3BP
IGF2BP3	TSSK1B		FASTK		COL6A2
Igtp	TTC3		FAT1		COMMD3
ILVBL	TUBB1		FAT4		COPA
INO80	TUBG1		FBXL16		COPE
INTS8	TXN2		FBXO7		COPS7B
INTU	U2SURP		FDX1		COPS8
Irs3	UBE2G2		FERMT2		COPZ2
ITCH	UBE2R2		FES		COQ6
ITGA5	UBR2		FEZF1		CORO1A
ITGB3	UCN		FGF2		CORO1C
ITM2C	UCP2		FGF7P5		COX18
JMJD8	UGT2A3		FKBP1C		COX7A2
KAT14	Usf1-rs3		FKBP3		CP
KAT2B	USP9X		FLT4		CPA3

KAZN	VCX3A		FNDC4		CPE
KBTBD11	VCX		FOLR1		CPEB3
KCNB1	VDAC1P5		FRG1BP		CPNE4
KCNJ3	VIP		FRG2		CPSF6
KCNK3	VIPR2		FRG2B		CPT2
KDM4B	VNN1		FRMD4B		CPXM1
KDM5C	WDR1		FSD2		CREB3L2
KDSR	WDR62		FUOM		CREB5
KHDRBS2	WNT5B		FUT9		CRELD1
KIAA0100	WTAP		FXR1		CRELD2
KIAA1468	XPA		GABPAP		CRLS1
KIF13B	ZBTB8OS		GABPB1		CRNN
KIF1B	ZFP36L1		GABRA2		CRTC1
KLC2	ZNF214		GABRG2		CSE1L
KLC4	ZNF235		GAK		CSF1R
Klf16	ZNF337		GAS1		CSNK1G2
KLF6	ZNF608		GAS6-AS1		Csprs
KLHL25	ZNF79		GBP2		CSRP1
KMT5B	ZRSR1		GC		CSTF1
KSR1			GEM		CTBP2
L3MBTL3			GEMIN7		CTGF
LACTB			GFRA4		CTNS
LAMB1			GGA2		CTPS2
LAMC1			GHRL		CTSA
LAMP1			GIPC2		CTSE
LDLR			GJB3		CTSV
LDLRAP1			GJB7		CUL1
LEP			GLMN		CUL3
LMNA			GLRX2		Cux1
LMNB2			GLRXP3		CXADR
LOC102632594			GLT8D1		CXCL17
LOC545966			GMNN		CYB561D1
LONP2			GNB1L		Cyb5r3
LRRC18			GNG10		CYCS
LRRC24			GNG11		CYHR1
LRRN1			GOLGA2P5		CYP2S1
LSM6			GOLGA6L2		CYP39A1
LTBP3			GOLGA7B		CYP3A7
LYSMD3			GOLGA8DP		Cyp4f16
MALAT1			GOLGA8F		CYP4F2
MAP2K5			GPAT2		Cyp4f37
MAP3K10			GPC6		CYR61
MAP3K2			GPM6B		D030028A08Rik
MAPK14			GPR1		D130020L05Rik

MAPK9			GPR108		D17H6S56E-5
MARCH2			GPR173		D2Bwg1423e
MARCO			GPR183		DAB2IP
MARS			GPR39		DAG1
MASTL			GPR45		DAO
MCAM			GPR50-AS1		DAPK3
MCCC2			GPR88		Dazap1
MCEE			GPRC5C		DAZAP2
MCUR1			GRAP		DCAF6
MDH1			GRHPR		DCAKD
MDH2			GRIK1		DCP1A
MDN1			GRIN2A		DCTN4
MDP1			GRK6		DCTN6
MECP2			GSAP		DCTPP1
MECR			GSTA7P		DDB2
MED22			GSTTP2		DDIT3
Meg3			GTF2H1		DDR1
MEGF9			GTF2IP1		DDX24
METAP1			GTPBP10		DDX39B
METTL4			GULP1		DDX41
MFN1			GUSBP2		DDX49
MFSD12			GXYLT1P6		DDX6
MFSD4B			GZMH		DEK
MGRN1			H1FNT		DENND3
Mirt1			H3F3AP5		DEPTOR
MKLN1			HAO2		DERL2
MKRN2			HCFC1R1		DGAT2
MKS1			HCG27		DGKG
MLX			HCG4		DGKZ
MNS1			HDAC7		DHPS
MPDU1			HEATR3		DHX15
MRI1			HGH1		DHX16
MRPL2			HIC2		DHX37
MRPL20			HIRA		DLEU7
MRPL27			HIVEP2		DLGAP4
MRPL45			HK1		DLX2
MRPS14			HLA-A		DMAP1
MRPS15			HLA-DQB1		DMBT1
MRPS16			HLA-G		DNAJB4
MRPS5			HMG20B		DNAJB6
MSRB3			HMGN2P18		DNAJC1
MTMR9			HNF1A		DNAJC12
MVP			HNRNPA0		DNAJC13
MXD4			HNRNPH1		DNAJC24

MYCBP2			HOXA10		DNAJC3
MYL6			HOXB3		DNASE1L2
MYLIP			HPN		DOCK4
MYNN			HPS3		DOK3
MYO19			HRK		DOT1L
MYOD1			HSBP1L1		DPF1
MYSM1			HSD11B1		DPT
N4BP2L2			HSF2BP		DPY30
NAA25			HSPA7		DPYSL2
NAA30			HSPB2		DRAM2
NADK2			HSPB6		Dst
NAGK			HSPH1		DTYMK
NAP1L4			HTR2A		DUSP13
NAPRT			IDO1		Dync1i2
NAT2			IDS		DYNLL1
NAT8L			IFNL1		E030030I06Rik
NCF1			IGDCC3		E2F3
NCOA4			IGFBP2		ECD
NCOR1			IGHV5-78		ECE1
NDRG2			IL10RB		ECI1
NDUFA1			IL12A		EDN1
NDUFA4			IL12RB2		EDNRB
NDUFA9			IL1RL1		EDRF1
NDUFAF8			IL23A		EED
NDUFB11			IL2RA		EEF1D
NDUFB9			IL32		EFNA4
Neat1			IL36RN		EGFR
NEDD8			ILDR2		EGLN3
NEK8			ILK		EGR1
NFIA			IMMP1L		EGR3
NFIC			INHBA		Eif1
NFKBIA			INPP5J		EIF1B
NHEJ1			IPO5		EIF3B
NKTR			IQCF1		EIF3C
NOA1			IRAK3		EIF3D
NOL3			IRS4		EIF3I
Nos1ap			IRX2		EIF3K
NPC2			ISM2		EIF3L
NR1H3			ISOC2		EIF4A1
NR3C1			ITGA11		EIF4A2
NRM			ITGB1		EIF4A3
NRXN1			ITIH6		EIF4G1
NSD3			IZUMO1R		EIF5
NSRP1			KAT14		EIF5A

NUDT13			KCNAB1		ELAC2
NUP155			KCNJ12		ELAVL1
OARD1			KCNQ4		ELMO2
OAZ2			KCNT2		Elob
OGFOD2			KCP		ELOVL5
OLR1			KCTD14		EMC1
ONECUT2			KCTD20		EMD
OPHN1			KCTD9P6		EMG1
OR51B4			KDELR1		EMILIN1
OSBPL1A			KDF1		ENG
OSMR			KDM4B		ENTPD5
OSTC			KIAA1024L		EPHB2
OTULIN			KIAA1755		EPRS
OXSM			KIAA2022		ERCC5
PAIP2B			KIF1BP		ERCC8
PALD1			KIR2DS3		ERGIC3
PBRM1			KITLG		ERICH5
PBX2			KLHDC2		ERLIN2
PC			KLHL12		ERMARD
PCCB			KLHL29		ERO1B
PCDH1			KLK13		ESD
PDE1B			KLK8		ETFA
PDHB			KLLN		ETNPPL
PDPR			KMT2C		EVI2A
PECR			KNOP1		EXOC1
PER1			KPTN		EXOC4
PER2			KRBA1		EXOSC7
PEX13			KRCC1		EXOSC8
PEX16			KRT16P1		F11R
PEX6			KRT17P3		F2R
PGPEP1			KRT18P40		F2RL1
PHF20			KRT18P44		FAAH
PHF20L1			KRT2		FABP3
PIK3C3			KRT33A		FAHD2B
PITHD1			KRTAP10-10		FAM110B
Pitpnc1			KRTAP21-2		FAM110D
PLA2G16			L3MBTL1		FAM114A2
PLCB4			L3MBTL3		FAM118B
PLEKHA2			LAMP3		FAM135A
PLEKHN1			LARP7		FAM160A2
PLIN4			LATS1		FAM171A2
PLPP3			LBH		FAM180A
PLXNC1			LBP		Fam184b
POLR2H			LBX1		FAM185A

POLR3GL			LCAT		FAM199X
POMGNT2			LCN1P1		FAM207A
PON2			let-7		Fam25c
PON3			LGALS13		FAM45A
Poteg			LGALS2		FAM63A
PPA1			LGALS7		FAM63B
PPAT			LGALS7B		FAM78A
PPM1H			LGALS8		FAM8A1
PPP2R3A			LGMN		FAR1
PPP3R1			LHFPL4		FASTKD1
Ppp4r1l-ps			LHX6		FBLN1
PPP4R3B			LINC00052		FBXL6
PPT1			LINC00264		FBXO3
PQLC1			LINC00339		FBXO31
PRDX1			LINC00346		FCGBP
PREB			LINC00452		FCGR2A
PRELID1			LINC00543		FCHO2
PRKD3			LINC00574		Fcna
PRNP			LINC00623		Fcrls
PROK1			LINC00638		FEM1C
PRSS23			LINC00893		FGD4
PSMA7			LINC00938		FGF14
PSMB4			LINC00998		FGFRL1
PSMD13			LINC01001		FGL1
PSMD7			LINC01116		FHL1
PSME1			LINC01132		Fhod1
PTDSS2			LINC01293		Fip1l1
PTGER1			LINC01326		FKBP11
PTGR2			LINC01618		FKBP2
Ptprd			LINC01844		FKBPL
PTS			LINC01962		FMC1
PXMP2			LINC02076		Fnbp1l
QKI			LINC02180		FNDC10
RAB39B			LINS1		FNDC5
RAB3D			LLPH		FNIP1
RAB3GAP2			LMBRD2		FOLR2
RAB3IP			LOC100128164		FOXQ1
RAB5B			LOC100129027		FOXS1
RAB8B			LOC100129098		FRMD4B
RAD18			LOC100130345		FRMD6
RAE1			LOC100289495		FRY
RAF1			LOC100420587		FSCN1
RALGAPA2			LOC100507191		FST
RAPGEF1			LOC101928278		FUNDC1

RASSF8			LOC101929163		FURIN
RBFOX2			LOC101929523		FZD7
RBM42			LOC101929709		FZR1
RBM5			LOC105378397		G3BP1
RC3H2			LOC148696		G3BP2
RCOR1			LOC151760		GABARAP
RDH10			LOC171391		GABARAPL1
RDH16			LOC202181		GADD45A
REPS2			LOC285422		GADD45GIP1
RGS2			LOC339192		GALT
RIC8B			LOC389906		GAPT
RNASEL			LOC390705		GAPVD1
RNF10			LOC399886		Gar1
RNF114			LOC401589		GAREM2
RNF135			LOC440040		GARS
RNF146			LOC442028		GART
RNF19A			LOC643441		GAS2L1
RNF215			LOC645513		GATA6
RNF31			LOC646736		GATAD2B
RNH1			LOC647115		GBF1
RPAP1			LOC727978		GCAT
RPL9			LOC728026		GCNT2
RPLP2			LOC730268		GDPD1
RPS7			LOXL1		GDPD3
Rptoros			LRP10		GFM1
Rrp1			LRRC25		GGCT
RSC1A1			LRRC3		GIN1
S100A4			LRRC46		GINS3
S1PR3			LRRC57		GJB3
SAFB			LRRC58		GJB4
SAFB2			LRRC61		GKAP1
SAMM50			LRRC77P		GLB1
SAP130			LRRC9		GLIPR1
SAYSD1			LST1		GLO1
SBDS			LYG2		GLRB
SBNO1			LYN		GLRX
SBNO2			LYNX1		GLRX2
SCAF4			MAGEA10		GLUD1
SCAMP4			MAGEA13P		Gm10073
SCARB1			MAGEA8		Gm10075
SCCPDH			MAGEB18		Gm10125
SCLY			MAGEB6		Gm10171
SCRN3			MAGI1		Gm10408
SCYL1			MALRD1		Gm1070

SDC3			MAN2B2		Gm11425
SDHD			MANEA		Gm11942
SDK2			MAP2K5		Gm12091
SEC62			MAP3K4		Gm12258
SEC63			MAP6		Gm12814
SEP11			MAPK11		Gm12918
SERGEF			MAPRE1P1		Gm13078
SF3B2			MARCH2		Gm13328
SF3B5			MATN4		Gm13553
SFI1			MAX		Gm13981
SFXN1			MBD2		Gm13991
SH3GLB2			MBD5		Gm14407
SH3PXD2A			MBOAT2		Gm14439
SHANK3			MBP		Gm14686
SHB			MDK		Gm14964
SHD			MECOM		Gm2036
SHPK			MED24		Gm2174
SIK2			MED8		Gm2423
SIRT4			MEIOB		Gm2991
SLC17A3			METTL18		Gm3090
SLC1A7			METTL23		Gm3552
SLC22A14			METTL4		Gm41386
SLC25A4			MFAP3L		Gm44505
SLC25A44			MFF		Gm4799
SLC25A5			MFRP		Gm4832
SLC37A3			MFSD12		Gm4866
SLC39A11			MFSD2A		Gm4876
SLC45A4			MFSD8		Gm4909
SLC9A6			MGAM2		Gm4968
SLC9A7			MGAT5		Gm5135
SLFN13			MICA		Gm5321
SLK			MIGA2		Gm5446
SLMAP			mir-1		Gm5552
SMAD5			mir-1202		Gm5561
SMIM10L1			mir-142		Gm5564
SMPD1			mir-194		Gm5643
SMTN			mir-219		Gm5913
SMYD4			mir-290		Gm6368
SND1			mir-299		Gm6382
SNW1			mir-302		Gm6433
SNX27			mir-361		Gm6462
SNX33			mir-379		Gm6467
SOAT2			mir-548		Gm6483
SOD2			mir-602		Gm6745

SON			mir-630		Gm6776
SORBS1			mir-767		Gm6988
SPATA2			MLC1		Gm7729
SPATC1L			MMP16		Gm7901
SPECC1			MMP2		Gm8163
SPHK2			MPI		Gm8194
SPTLC2			MPP1		Gm8225
SRA1			MPZL2		Gm8228
SRP9			MRPL10		Gm8587
Srrm1			MRPL21		Gm9574
SSH1			MRPL42		GMCL1
SSR4			MRPS21		GMIP
ST3GAL3			MRPS7		GNA13
ST6GAL2			MS4A3		GNAQ
STIM2			MS4A7		GNAS
STK10			MSR1		GNG2
STK17B			MT1CP		GNL2
STK25			MT1F		GNL3L
STK38			MT1H		GNS
STK39			MTHFSD		GOLGA2
STX18			MTMR1		GOLGA5
STX1A			MTRR		GOLGB1
STX5			MUC12		GOLT1B
STXBP4			MUC19		GORASP1
Sult1a1			MUC3B		GORASP2
SURF6			MUM1L1		GOSR2
SYNGR1			MUSK		GPAT4
SYPL1			MXD1		GPATCH4
SZRD1			MYBPH		GPHB5
TACC1			MYH15		Gpr137b-ps
Taco1os			MYH6		Gprasp2
TAF1C			MYO1C		GPSM1
TAOK1			MYO1H		GPX2
TATDN2			NAB2		GPX4
TBC1D13			NACC1		GRAMD1B
TBC1D4			NASP		GSG2
TCF4			NAT16		GSTCD
TCF7L1			NAT2		GSTP1
TCP11L2			NBPF10		Gstp3
TDP1			NCKAP5		GTF2H4
TENM4			NCOA1		GTF2H5
TGS1			NCOR1		GTPBP6
THAP2			NDRG4		GUCA2B
TIMM9			NDUFAB1		GYG1

TIMP2			NDUFAF3		GZMH
TK2			NDUFAF4		H2AFZ
TKT			NDUFAF5		H2-K2
TLE1			NDUFAF6		H2-Q5
TLE6			NDUFAF7		H2-Q8
TLR3			NDUFB6		H2-Q9
TM4SF5			NDUFV1		H6PD
TM7SF3			NEK3		HAS1
TMC7			NEK6		HCFC2
TMCC3			NEPRO		HDAC7
TMED2			NEURL3		HELZ
TMEM177			NF1		HERPUD1
TMEM19			NFASC		HES6
TMEM201			NFKB2		HEXA
TMEM206			NFX1		HGFAC
TMEM263			NFXL1		HIF3A
TMEM57			NGF		HIGD2A
TMEM94			NHLRC4		HIST2H2AA3
TMEM9B			NKX3-1		HIST2H2AA4
TNFRSF10A			NME1		HK1
TNFRSF8			NME6		HLA-A
TNRC6B			NOMO1		HLA-DQA1
TOR1B			NPB		HLA-DQB1
TOR2A			NPEPL1		HLA-DRA
TP63			NPFFR1		HLCS
TP73			NPL		HM13
TPD52L2			NPM1P21		HMBS
TPRKB			NPM1P46		HMCN1
TPX2			NPTXR		HMCN2
TRAF3IP2			NR5A2		HMGN1
TRAIP			NRXN1		Hmgn2
TRAPPC9			NTM		Hnrnpa1
TRIM44			NTN1		HNRNPC
TRIM56			NTNG1		HNRNPDL
TRIP12			NUAK2		HNRNPL
TRNT1			NUB1		HNRNPM
TSPAN18			NUDT16P1		HOPX
TST			NUDT9		HOXB2
TTC14			NUP35		HOXB3
TTC38			NUP54		HOXB7
TTC39B			NUP85		HP1BP3
Ttf1			NUS1P2		HS6ST2
TUBG1			NUTF2P4		HSP90AA1
TXNDC11			NVL		HSP90AB1

UBALD1			NXF2		HSP90B1
UBE2B			NXF2B		HSPA1A
UBE2J1			NXPH4		HSPA1B
UBQLN1			OAT		HSPA2
UBQLN4			OC90		HSPA8
UBR3			OCIAD2		HSPB1
UBR4			OGDH		HSPBP1
UBXN4			OGFRP1		HSPD1
UHMK1			OLFM4		HSPE1
UQCC1			OLR1		HSPH1
UQCC3			OOSP1		HTATIP2
UQCRC1			OR12D3		HVCN1
UQCRC2			OR13H1		IAH1
URGCP			OR2A4		IARS
UROD			OR2A7		IARS2
USP12			OR2G6		ICA1
USP20			OR2L13		ICE1
USP46			OR2L8		ID4
UTP6			OR52N1		IDH1
VAC14			OR5C1		IFNA4
VAMP4			OR5H1		IFRD2
VASP			OR5K3		IFT81
VEGFB			OR7E14P		IGF1
VPS13C			OR8D2		IGFBP2
VPS25			OR8G5		IGFBP4
VPS45			OR8H1		Ighv5-4
WASHC2A			OR9A4		Ighv6-3
WASHC2C			OR9K2		Igkv14-100
Wasl			ORC2		Igkv15-103
WDR26			ORM1		Igkv3-2
WDR41			ORM2		Igkv4-58
WDR53			OSCP1		Igkv5-48
WHAMM			OSER1		Igkv6-23
WNK1			OTOGL		IGSF3
WRN			OTP		IKZF1
Xlr			OVCA2		IL11RA
XPR1			OVOL1		IL17RC
YKT6			OXSR1		IL1A
YME1L1			P2RY6		IL3RA
YWHAG			P4HA2		IL4I1
ZBTB16			P4HTM		ILVBL
ZBTB20			PAICS		IMMP1L
ZBTB7A			PALD1		IMP3
ZDHHC7			PAN2		IMP4

Zfp874a			PANK3		IMPAD1
Zfp874b			PAQR3		IMPDH2
ZHX3			PARD6G		ING3
ZMIZ1			PARP9		INHBA
ZMYM5			PARS2		INO80
ZNF106			PC		INSR
ZNF12			PCDH1		INTS13
ZNF236			PCDH10		INTS4
ZNF24			PCDHA7		INTS5
ZNF274			PCDHB10		IP6K1
ZNF318			PCDHGB5		IPMK
ZNF330			PCYOX1L		IPO4
ZNF398			PDE3B		IPP
ZNF560			PDE4DIP		IQGAP2
ZNF667			PDE6A		IRAK1
ZNF697			PDE6C		IRF2
ZNF775			PDE7B		IRF3
ZNF8			PDGFD		Irs3
ZNRF2			PDIA3		ITCH
ZSCAN12			PDIA3P1		ITGAE
ZSCAN20			PDLIM3		ITGAM
1810014B01Rik			PGLYRP2		ITGB8
1810073O08Rik			PHF13		ITGBL1
2010320M18Rik			PHIP		ITM2B
2610012C04Rik			PI4KA		IVD
2700080J24Rik			PICSAR		JADE1
2810047J09Rik			PIDD1		JAGN1
2810421E14Rik			PIK3CG		JAML
2900009J06Rik			PIK3R2		KALRN
3830403N18Rik			PINLYP		Kap
4833403J16Rik			PKD1L2		KARS
4930401B11Rik			PKIB		KAT2B
4930449I04Rik			PLAA		KAT7
4930465M20Rik			PLAT		KCNA2
4930484H19Rik			PLCG2		KCNAB1
4930507D05Rik			PLEC		KCNH1
4930515G01Rik			PLEK		KCNK2
4930544F09Rik			PLEKHA3		KCTD1
4930592A05Rik			PLEKHF2		KDM5A
5430406J06Rik			PLEKHG1		KDM5B
5730406G12Rik			PLGLA		KDM7A
5830433M15Rik			PLPP3		KDSR
6430500D05Rik			PMS2CL		KEAP1
6430501K19Rik			PNLIPRP3		KIAA0922

8030453O22Rik			POLM		KIAA1217
8030498B09Rik			POLR3G		KIAA1324
9330115C17Rik			POM121L2		KIAA1522
9430012M22Rik			POMC		KIAA1551
9430047L24Rik			POMT1		KIDINS220
9430064K01Rik			POT1		KIF1A
9430099H24Rik			POU2F1		KIF1B
A130019P10Rik			PP14571		KIF3A
A630035D09Rik			PPA2		KIF3B
			PPARGC1B		KIF3C
			PPIC		KLC1
			PPIP5K1		KLF6
			PPP2R3B		KLF7
			PPP3R2		KLHDC4
			PQLC2		KLHL21
			PRAC2		KLHL25
			PRAM1		KLHL5
			PRAMEF4		KLK3
			PRDM8		Klra7
			PRKX		KNG1
			PRMT9		KPNB1
			PRR22		KRBA1
			PRR23D1		KRR1
			PRR23D2		KRT6B
			PRR27		KSR1
			PRR29		LAIR1
			PRR30		LAMP1
			PRR5-ARHGAP8		LAMP2
			PSG6		LAPTM4A
			PSMA1		LAPTM5
			PSMA7		LARGE1
			PSMB1		LARP7
			PSMD12		LARS
			PSMF1		LBH
			PTER		LCN2
			PTF1A		LCP1
			PTGDR		LDAH
			PTGER3		LDHD
			PTGFRN		LDLR
			PTGIS		LEF1
			PTPMT1		LEFTY1
			PTPN14		LEPROT
			PTPN22		LGALS4
			PXMP4		LGALS8

			PYCARD		LGALSL
			QARS		LIAS
			RAB1B		LIF
			RAB27A		LIG3
			RAB44		LILRB4
			RAB4B		LIPG
			RABEP1		LMAN2L
			RABGGTB		LMNA
			RABL2A		LMNB2
			RANBP10		LOC102724023
			RAP1GDS1		LONRF3
			RAP2B		LOR
			RASAL2		LOX
			RASGRP3		LOXL3
			RASL11A		LPIN2
			RBM24		LPO
			RBM46		LRG1
			RBMX2		LRP1
			RBMY1A1		LRP10
			RBMY3AP		LRP2
			RCCD1		LRRC15
			RCHY1		LRRC59
			RDH14		LRRC8B
			REPS2		LRRFIP1
			RET		LSM6
			RGS7		Lst1
			RHOH		LTF
			RHOXF1		LUM
			RIOK1		Ly6a
			RNF126P1		LY6G6D
			RNF135		LYAR
			RNF138		LYL1
			RNF182		Lypd2
			RNPS1P1		LYSMD2
			RNU12-2P		MAN2B1
			RORA		MAN2B2
			RPL23AP12		MAN2C1
			RPL23AP42		MANBA
			RPL4P2		MAP1B
			RPL9		MAP1S
			RPP30		MAP2K4
			RPS21		MAP2K6
			RPS27A		MAP7
			RPS27AP11		MAPK6

			RPS28		MAPK8IP1
			RPS4X		MAPKAP1
			RPS6KC1		MAPRE1
			RRP15		Marcks
			RSAD1		MARVELD3
			RSC1A1		MASP2
			RUFY2		MAT2B
			RWDD1		MBD5
			S100PBP		MBNL2
			S1PR1		MBTD1
			SAA1		MCM2
			SAE1		MCMBP
			SAMD12		MCRIP2
			SBF2		MDH2
			SBF2-AS1		MDK
			SBK1		MED1
			SCARB1		MED15
			SCARB2		MED27
			SCFD2		MEIS3
			SCMH1		METTL1
			SCO1		MFAP3L
			SCRN1		MFSD11
			SDC4		MIA
			SDHAP1		MICALL1
			SDHC		MICU2
			SDHCP2		MID1
			SDR16C5		MID2
			SEC11C		MIER1
			SEC14L6		MIER2
			SEC23B		MINOS1-NBL1
			SEC31A		MKI67
			SELENOF		MKNK1
			SEMA5A		MLEC
			SEP3		MMP11
			SEP4		MMP14
			SEPHS1P4		MMP2
			SERINC5		MMP24
			SERPINB6		MOB4
			SERTAD2		MOGS
			SESN1		MOSPD1
			SETD3		MOSPD3
			SFXN4		MPHOSPH6
			SH3GL2		MRC1
			SH3KBP1		MRE11

			SH3RF2		MRPL15
			SHANK3		MRPL16
			SHISA2		MRPL17
			SHOX2		MRPL28
			SHROOM2		MRPL3
			SIAH1		MRPL38
			SIAH2		MRPL4
			SIDT1		MRPL52
			SIMC1		MRPS10
			SLAIN1		MRPS12
			SLC12A5		MRPS24
			SLC12A7		MRPS28
			SLC16A6P1		MRPS33
			SLC18B1		MRPS9
			SLC22A24		MSI2
			SLC24A4		MSMO1
			SLC25A5P8		MTCH2
			SLC26A6		MTHFD2
			SLC26A9		MTMR1
			SLC2A4RG		MTMR11
			SLC35B1		MTPN
			SLC38A7		MTX1
			SLC43A3		MUC13
			SLC44A3		Muc4
			SLC47A1		MUSTN1
			SLC4A1		MYADM
			SLC5A10		MYBBP1A
			SLC7A13		MYC
			SLC9A9		MYCBPAP
			SLC9B1P4		MYD88
			SLFN13		MYH10
			SMAD4		MYH9
			SMARCA2		MYL1
			SMPD4		MYL4
			SNHG3		MYLK2
			SNN		MYLPF
			SNORA16A		Myo15b
			SNORA55		MYO1D
			SNORA69		MYO1F
			SNORA70C		MYO9B
			SNORD105		MYOF
			SNORD109B		MYRIP
			SNORD119		N4BP2L1
			SNORD36C		N6AMT1

			SNORD37		NAA10
			SNORD4B		NACA
			SNORD68		NADK
			SNORD89		Naip1
			SNORD96B		NAP1L1
			SNRNP27		NARF
			SNRNP48		NARS
			SNRPA1		NBEAL1
			SNTB2		NBL1
			SNX32		NCAPG2
			SORBS1		NCAPH
			SOWAHC		NCAPH2
			SOX17		NCDN
			SP140		NCEH1
			SPAG8		NCKAP1L
			SPANXD		NCL
			SPARCL1		NCOA4
			SPATA2L		NDFIP2
			SPATA31C2		NDN
			SPDYA		NDOR1
			SPDYE2		NDRG1
			SPINK8		NDUFA12
			SPPL2B		NDUFA3
			SRC		NDUFAB1
			SRGAP2		NDUFAF2
			SRI		NDUFAF5
			SRP9P1		NDUFS3
			SRRM1P3		NDUFV1
			SRRM2		NECAP1
			SRSF9		NECAP2
			SSH1		NECTIN2
			SSPN		NEDD4L
			SSR1		NEDD9
			SSR2		NEK1
			SSX2		NELFE
			SSX2B		NEU1
			ST13P6		NEURL2
			ST20		NEUROD6
			STAG3L4		NFAT5
			STARD10		NFATC4
			STARD5		NFE2
			STARD7		NFIB
			STAT6		NFIC
			STK31		NFIX

			STN1		NFKB2
			STOML2		NFKBIE
			STOX2		NFYA
			STRADA		NFYC
			STRADB		NHLRC1
			STX7		NHLRC3
			SUGT1		NHP2
			SULF2		NHSL1
			SUMO2P6		NIPBL
			SUOX		NIPSNAP3A
			SYNC		Nisch
			SYNE3		NKX2-2
			SYNPO2		NME7
			SYPL2		NMRAL1
			SYT15		NNAT
			SYT4		Nnt
			TACR1		NOA1
			TAGLN		NOC2L
			TANC2		NOL11
			TAPBP		NOL12
			TBC1D28		NOL6
			TBKBP1		NOMO1
			TBX10		NOP14
			TBX5		NOSTRIN
			TCAIM		NOTCH1
			TCEA2		NOTCH4
			TCF21		NPC1
			TCF25		NPEPL1
			TCFL5		NPFFR2
			TCN2		NPHP4
			TCP11L1		NPR1
			TDGF1		NR1I3
			TDRD5		NR2F2
			TECPR2		NR5A2
			TERF2		NRXN2
			TESC		NSD2
			TET3		NSDHL
			TEX22		NSFL1C
			TFAMP2		NSUN5
			TFPI		NT5C2
			TFRC		NTAN1
			TGFB2		NTMT1
			TIGD7		NTN5
			TIMM44		NUBP1

			TINAG		NUBPL
			TKFC		NUDT21
			TLDC1		NUDT4
			TLE6		NUMB
			TLR4		NUPR1
			TMC8		NXN
			TMED10P1		NXT1
			TMED8		NXT2
			TMEM123		Oaz1-ps
			TMEM126B		OBP2B
			TMEM217		ODC1
			TMEM235		OGFRL1
			TMEM251		OLFM1
			TMEM255A		Olfr1113
			TMEM44		Olfr1115
			TMEM59		Olfr1329
			TMEM63B		Olfr458
			TMEM72		OPTN
			TMEM80		OR6A2
			TMEM91		OR6C6
			TMTC1		OR7G3
			TMTC4		ORC5
			TNFSF14		OSBP
			TNFSF4		OSTM1
			TNR		OTC
			TNS2		OTUD6B
			TOM1		OTUD7B
			TP53		OVOS2
			TP53TG3HP		P2RX4
			TPD52L3		P2RX5
			TPM1		P2RX7
			TPRXL		P2RY13
			TPTE2P3		P2RY6
			TRADD		P4HA1
			TRAPPC1		PABPC1
			TRHR		PABPC4
			TRIM37		PADI2
			TRIM49B		Pakap
			TRIM51FP		PAMR1
			TRIM62		PAPD7
			TRIML1		PAPLN
			TRMT2A		PAPSS1
			TRNAU1AP		PARD3
			TRPC6		PARM1

			TRPV4		PARP4
			TSG101		PARVG
			TSPAN1		PATJ
			TSPY1		PAWR
			TSPY6P		PBK
			TTC30B		PCBP1
			TTC34		PCBP2
			TTC38		PCCA
			TTTY13		PCDH17
			TTTY17A		PCDHB4
			TTTY5		PCDHGA4
			TTTY6B		PCIF1
			TVP23C		PCOLCE2
			TXN		PCSK4
			UAP1L1		PCYOX1
			UBA7		PCYT1B
			UBE2D3		PCYT2
			UBE2J1		PDAP1
			UBE2K		PDCD2
			UBE2L3		PDCD4
			UBE2L6		PDE1B
			UBL4B		PDE3A
			UBTFL11		PDE4DIP
			UBXN8		PDE7A
			UCA1		PDGFRB
			UGT2B17		PDIA6
			UMAD1		Pdlim3
			UMODL1-AS1		PDPK1
			UNC13D		PDXK
			UNC93B2		PDZK1IP1
			UNKL		PEAR1
			UPF2		PECAM1
			USP17L6P		PEF1
			USP2		PEG3
			USP24		PES1
			USP4		PEX6
			USP50		PFN1
			USP9Y		PGLYRP1
			USPL1		PHAX
			VBP1		PHF10
			VCL		PHF12
			VDR		PHF13
			VGLL2		PHF20L1
			VPS13A-AS1		PHGDH

			VPS35		PHKB
			VSTM2A		PHLPP1
			WAC		PHYKPL
			WASH5P		PI4KB
			WBP4		PIAS2
			WDPCP		PID1
			WDR12		PIGH
			WDR33		PIGL
			WDR83		PIK3CD
			WFDC10A		PIK3CG
			WNK2		PIK3R1
			XAGE3		PIK3R3
			XCL1		PIM3
			XKRX		PIP4K2A
			XYLT1		PIP4K2C
			YIPF2		Pira11
			YKT6		Pira4
			YY1AP1		Pisd-ps1
			ZAP70		Pisd-ps2
			ZBBX		PJA1
			ZBTB12		PJA2
			ZBTB45		PKDCC
			ZBTB8OS		PKN3
			ZCCHC18		PKNOX1
			ZDHHC1		PKP4
			ZFP82		PLA1A
			ZFR2		PLA2G4B
			ZGPAT		PLA2G4E
			ZKSCAN1		PLAU
			ZNF131		PLCD1
			ZNF132		PLCG2
			ZNF134		PLD3
			ZNF16		PLD4
			ZNF2		PLEC
			ZNF22		PLEKHA5
			ZNF225		PLEKHM3
			ZNF234		PLET1
			ZNF253		PLGRKT
			ZNF259P1		PLIN1
			ZNF274		PLIN2
			ZNF286A		PLPP3
			ZNF300		PLSCR4
			ZNF331		PLXNA2
			ZNF333		PMEPA1

			ZNF385B		PMM1
			ZNF407		PNISR
			ZNF416		PNPLA1
			ZNF417		PNPLA7
			ZNF468		PNPO
			ZNF493		POC1B
			ZNF563		POLD1
			ZNF581		POLD2
			ZNF587		POLE4
			ZNF589		POLR1C
			ZNF614		POLR2C
			ZNF623		POLR2F
			ZNF674-AS1		POLR3K
			ZNF677		PON1
			ZNF699		POP4
			ZNF706		POU6F1
			ZNF716		PPAN
			ZNF737		PPFIBP2
			ZNF799		PPIC
			ZNF844		PPIH
			ZNF93		PPM1A
			ZNRF1		PPM1B
			ZSCAN16-AS1		PPM1K
					PPM1M
					PPP1CB
					PPP1R14D
					Ppp2r5c
					PPP4R3B
					PPP5C
					PRAP1
					PRDX1
					PREPL
					PRKCD
					PRKG1
					PRMT5
					PRPF38A
					PRPF38B
					PRPF6
					PRPF8
					PRPS1
					PRR15L
					Prr33
					PRRC2C
					PRRG1

					Prrt1
					PRRX1
					PSAP
					PSAT1
					PSEN1
					PSMG2
					PTBP1
					PTCD2
					PTGES3
					PTGES3L
					PTN
					PTP4A1
					PTPN1
					PTPN14
					PTPN6
					Ptprd
					PTPRO
					PURB
					PUS1
					PVALB
					QPRT
					QSOX1
					QTRT1
					RAB23
					RAB9A
					RABGAP1
					RABL2A
					RABL6
					RAC3
					RAD50
					Raet1b
					RALB
					RALY
					RAMP1
					RANBP9
					RANGAP1
					RAP2C
					RAPGEF5
					RARG
					RARRES2
					RARS
					RASA4
					RASL12
					RASSF5

					RAVER1
					RAVER2
					RB1
					RBBP8
					RBM10
					RBM18
					RBM39
					RBM42
					RBM47
					RBM48
					RBM8A
					Rbm8a2
					RBPMS
					RCHY1
					RCL1
					RCOR1
					RCSD1
					REEP3
					RENBP
					Retnlg
					REXO2
					RFC4
					RFFL
					RGL3
					RGP1
					RHBDD1
					RHBDL2
					RHEB
					RHOF
					RHOJ
					RHOU
					RILPL2
					RING1
					RIOK1
					RIOX1
					RNASE2
					RNASE6
					RNASET2
					RND2
					RNF103
					RNF139
					RNF181
					RNF208
					RNF6

					RNGTT
					RNMT
					ROR2
					RPA2
					RPF1
					RPIA
					RPL12
					RPL18
					RPL18A
					Rpl21-ps4
					RPL24
					RPL26
					RPL27A
					Rpl27a-ps1
					RPL28
					Rpl31-ps15
					Rpl31-ps20
					RPL35
					RPL6
					RPL7L1
					RPLP0
					RPS10
					Rps12-ps24
					Rps13-ps5
					RPS15
					RPS16
					RPS21
					RPS24
					RPS29
					RPS3
					RPS5
					RPS6KB1
					Rpsa-ps1
					Rpsa-ps2
					RPTOR
					RPUSD4
					RRM1
					RRP8
					RRP9
					RSF1
					RSPH9
					RSRC1
					Rsrc2
					RTCB

					RTN3
					RUNX1T1
					RUVBL1
					RXFP3
					S100A8
					SAE1
					SAMHD1
					SAP130
					SARAF
					SART1
					SBF1
					SBF2
					SCAMP2
					SCAMP5
					SCAP
					SCFD1
					SCGB3A1
					SCGB3A2
					SCRIB
					SDC4
					SDCBP
					SDCBP2
					SDF2
					SDF2L1
					SDHAF1
					SEC11A
					SEC61B
					SEH1L
					SELENON
					SELENOP
					SELENOW
					SELPLG
					SENP1
					SEP8
					SEP9
					SERAC1
					SERINC1
					SERINC2
					SERINC3
					SERPINA1
					SERPINA6
					SERPINB6
					SERPING1
					SERPINH1

					SERTAD3
					SETD5
					SF3B2
					SFTPD
					SFXN1
					SGCA
					SGK3
					SGPL1
					SH3BGRL3
					SH3BP2
					SH3KBP1
					SH3PXD2B
					SH3TC2
					SHC1
					SHMT1
					SHMT2
					SIDT2
					SIN3A
					SIN3B
					SKI
					SKP2
					SLA
					SLC11A2
					SLC12A2
					SLC15A2
					SLC1A5
					SLC24A3
					SLC25A10
					SLC25A19
					SLC25A27
					SLC25A3
					SLC25A36
					SLC25A38
					SLC26A4
					SLC26A9
					SLC27A2
					SLC30A9
					SLC31A1
					SLC35B1
					SLC35B2
					SLC35B3
					SLC37A2
					SLC38A9
					SLC39A11

					SLC39A8
					SLC40A1
					SLC44A2
					SLC4A7
					SLC5A8
					SLC6A13
					SLC7A8
					SLC8A1
					SLC8B1
					SLCO4A1
					SLIRP
					SLTM
					SMAGP
					SMARCA4
					SMC1A
					SMC2
					SMC3
					SMCHD1
					SMIM15
					Smim4
					SMPD3
					SMPDL3A
					SMU1
					SNAP25
					SNN
					SNRNP48
					Snrpa
					SNRPB
					SNRPF
					SNU13
					SNX18
					SOD1
					SOX12
					SOX5
					SPATA9
					SPECC1
					SPINT1
					SPP1
					Sprr2a3
					Sprr2b
					Sprr2d
					Sprr2g
					SPSB2
					SPSB4

					SQLE
					SQSTM1
					SREBF2
					SRM
					SRP14
					SRP54
					SRP9
					SRSF10
					SRSF11
					SRSF2
					SRXN1
					SSBP4
					SSPN
					SSRP1
					SSSCA1
					ST3GAL3
					ST8SIA4
					STAB1
					STARD10
					STARD3NL
					STARD4
					STARD5
					STAT3
					STC2
					STEAP1
					STEAP2
					STIP1
					STK19
					STK25
					STK32A
					STRA6
					STRADB
					STT3B
					STX11
					STX17
					STX3
					STX7
					STX8
					SUGP2
					SULT1C2
					SUMO1
					SUN2
					SUPT3H
					Supt4b

					SUPT6H
					SVEP1
					SYAP1
					SYF2
					SYNE4
					SYNJ2
					SYNJ2BP
					SYNM
					SYPL1
					SYTL2
					SYTL3
					SYVN1
					SZRD1
					TAF10
					TAF7
					TANGO2
					TANK
					TAOK1
					TAPBP
					TARS
					Tas2r143
					TATDN3
					TAZ
					TBC1D10B
					TBC1D2B
					TBC1D7
					TBCB
					TBCD
					TBL1X
					TBL1XR1
					TBRG1
					TCF20
					TCF3
					TCIRG1
					TCN2
					TCOF1
					TEAD2
					TECPR2
					TERF2
					TES
					TFAM
					TFAP2B
					TFAP2C
					TFPT

					TGFB1I1
					TGFB2
					TGFBR1
					TGFBR2
					TGS1
					THAP4
					THBS2
					THNSL2
					THOC7
					THOP1
					THSD4
					THTPA
					TIA1
					Timd2
					TIMM10B
					TIMMDC1
					TIMP2
					TLCD2
					Tlr13
					TLR7
					TM6SF1
					TMC7
					TMCO1
					TMED10
					TMED4
					TMEM127
					TMEM139
					TMEM154
					TMEM167A
					TMEM176A
					TMEM181
					TMEM209
					Tmem223
					TMEM229B
					TMEM237
					TMEM255B
					TMEM260
					TMEM261
					TMEM68
					TMEM8A
					TMEM9
					TMPO
					TNC
					TNFAIP2

					TNFAIP8L2
					TNFRSF9
					TNIK
					TNNC2
					TNS1
					TNS3
					TOMM40
					TOMM6
					TOP1MT
					TOR1AIP1
					TOR1AIP2
					TP53INP1
					TP53INP2
					TPK1
					Tpm2
					TPR
					TPRG1L
					TRAF1
					TRAF3IP2
					TRAF4
					TRAF6
					TRAF7
					TRAM1
					TRAP1
					TRAPPC1
					TRAPPC2L
					Trdv2-2
					TREM2
					TRIB3
					TRIM10
					TRIM11
					TRIM15
					TRIM27
					TRIM36
					TRIM40
					TRIO
					TRIP12
					TRMT2B
					TRPM6
					TSC22D2
					TSEN2
					TSN
					TSPAN1
					TSPAN8

					TSR1
					TTC1
					TTC33
					Ttc41
					TTYH3
					TUBA3E
					TUBB2B
					TWISTNB
					TWSG1
					TXN2
					TXNL4A
					TYROBP
					U2SURP
					UAP1L1
					UBE2A
					UBE2G1
					UBE2Q1
					UBFD1
					UBL5
					UBOX5
					UBQLN1
					UBTF
					UCK2
					UCMA
					UGT1A6
					UHRF2
					ULK1
					ULK2
					Umad1
					UMPS
					UNC5A
					UNG
					Uox
					UPF1
					UPF2
					UPK3BL
					UQCRC1
					USP10
					USP48
					VAMP7
					VAMP8
					VANGL2
					VAPA
					VARS2

					VAT1
					VAV1
					VAV2
					VCP
					VEZT
					VILL
					VLDLR
					Vmn1r214
					VMP1
					VPS13A
					VPS13B
					VPS28
					VPS33A
					VPS33B
					VPS54
					VPS9D1
					VRK1
					VSIG10
					VTCN1
					VTI1B
					VWA8
					WARS
					WARS2
					WAS
					WASHC4
					WBP1
					WBP4
					WBSCR17
					WDR20
					WDR4
					WDR46
					WDR73
					WDYHV1
					Wfdc10
					Wfdc18
					WLS
					WNK2
					WNT16
					WNT7B
					WSB1
					WSB2
					WWP1
					XBP1
					XIST

					XPC
					YBX1
					YDJC
					YEATS4
					YIF1A
					YIF1B
					YIPF2
					YIPF4
					YPEL5
					YTHDF3
					YWHAB
					YWHAG
					YWHAH
					ZBTB18
					ZBTB24
					ZBTB7A
					ZC2HC1B
					ZC3H10
					ZC3H12A
					ZC3HC1
					ZCCHC17
					ZCCHC4
					ZCCHC6
					ZCRB1
					ZDHHC15
					ZEB2
					ZER1
					ZFAND6
					ZFC3H1
					ZFP28
					ZFP36L1
					Zfp60
					Zfp658
					Zfp763
					Zfp810
					Zfp87
					Zfp871
					Zfp950
					ZGPAT
					ZGRF1
					ZMAT1
					ZNF236
					ZNF326
					ZNF329

					ZNF428
					ZNF436
					ZNF445
					ZNF519
					ZNF777
					ZNF787
					ZNF8
					ZRSR2
					ZW10
					1700027J07Rik
					1700042G07Rik
					1700109H08Rik
					1700120B22Rik
					1810009N02Rik
					1810032O08Rik
					1810037I17Rik
					2210407C18Rik
					2310039H08Rik
					2700033N17Rik
					2700097O09Rik
					4921501E09Rik
					4930528A17Rik
					4933427G23Rik
					5033430I15Rik
					6820431F20Rik
					9130230L23Rik

Supplementary Table S8. The list of genes overlapping between DEGs in the hippocampus of rats prenatally exposed to BPA and the lists of BPA-responsive genes identified by the reanalysis of previously published transcriptome profiling studies in NCBI GEO DataSets database.

Overlap with	GSE44387	GSE63852	GSE58642	GSE50527	GSE58516	GSE86923		Combined lists
DEGs in both sexes	ABCB9	ABCA4	CSGALNACT1	ADAMTS17	NARFL	2310039H08Rik		ABCB9
	ACAA2	ABHD14A	FRYL	AGAP1	PIGH	AAGAB		ACAA2
	ACACB	ABL2	HS3ST3A1	AGK	SIDT1	AARD		ACACB
	ACLY	ACSS3	LGR5	AGL	STRADA	AATF		ACLY
	ACSL1	ADORA2B	LPL	ALAD	TMEM185A	ABCA7		ACSL1
	ADAMTS4	AGA		ALG12	ZNF212	ABCB8		ADAMTS4
	ADTRP	AIFM1		ALOXE3		ABHD6		ADTRP
	AGL	ALDH18A1		ARHGAP25		ACAA2		AGL
	AK1	ALDH5A1		ARRDC2		ACADVL		AK1
	AKR1A1	ASL		ATP1A4		ACP2		AKR1A1
	ALDH16A1	ATF6B		ATP2A2		ADA		ALDH16A1
	ALDOA	ATP2B3		B4GALNT3		ADGRG2		ALDOA
	APMAP	ATXN2		BAAT		AGTR1		APMAP
	APOE	BHLHA15		BCOR		AHCYL2		APOE
	ARHGEF25	BMP3		BOD1		AIDA		ARHGEF25
	ARIH2	BUB1B		C18orf25		AIF1L		ARIH2
	ASL	C1QBP		C1orf43		AKR1A1		ASL
	ASTN1	CALR		C1QTNF1		Akr1c13		ASTN1
	ATG101	CAPSL		C1R		ALOX5		ATG101
	ATP6V0A1	CCDC43		C2orf70		AMBRA1		ATP6V0A1
	ATXN2	CDC42EP3		CACNA1G		ANKRD11		ATXN2
	BAD	CDH15		CACTIN		ANKS6		BAD
	BCL2L2	CLSTN2		CAPNS2		AP4M1		BCL2L2
	BFAR	CMTR1		CATSPER2		APBB1IP		BFAR
	BORCS8	COL5A2		CBLN3		APEH		BORCS8
	BPGM	CTNNBL1		CBX7		AQR		BPGM
	C11orf54	DDX6		CCDC103		ARG1		C11orf54
	C22orf23	EPC1		CCDC113		ARSA		C22orf23
	C3orf14	ETHE1		CCDC153		ASNS		C3orf14
	CCDC167	EXO1		CCDC167		ASPH		CCDC167
	CCDC81	FASLG		CCER2		ATF4		CCDC81
	CDHR4	FDFT1		CD79B		ATIC		CDHR4
	CHID1	FER		CDC25B		ATP2A1		CHID1
	CHPT1	GGCT		CDC6		ATP2B2		CHPT1
	CHRDL1	GOT1		CDKL2		ATP6AP1		CHRDL1
	CHRNA4	HEXIM1		CERS6		ATXN1		CHRNA4
	CIAO1	HNRNPC		CHD7		AVEN		CIAO1
	CIC	IL27RA		CHKA		B3GALNT1		CIC
	CLEC16A	IVD		CHST9		B4GALT3		CLEC16A
	CMPK2	KCNA2		COL9A1		BAP1		CMPK2
	COA7	MAP3K4		CPNE7		BHLHA15		COA7
	COG2	MBD2		CROT		BMS1		COG2
	CPTP	MTMR2		CTDSPL2		BNC2		CPTP
	CSDE1	MYLIP		CUX1		BTAF1		CSDE1
	CSF2RB	MYO9B		CYTH1		BTD		CSF2RB
	CTBS	NFKBIB		DACT1		BTK		CTBS
	CTSB	NIT1		DGAT1		C11orf98		CTSB
	CYHR1	PDE7B		DKK2		C12orf49		CYHR1

	CYP1B1	PIAS1		EBI3		C1QB		CYP1B1
	CYP2J2	PIN1		EFEMP1		C20orf24		CYP2J2
	DCAF5	PLA2R1		EHD2		C3orf14		DCAF5
	DDX27	PLXNA3		EIF4ENIF1		C5orf22		DDX27
	DENND2D	PNOC		ELMO1		CA13		DENND2D
	DGAT1	POLA2		EPC2		CACNA1G		DGAT1
	DSTYK	PPM1E		EPSTI1		CACNB2		DSTYK
	EAPP	PRKAG1		ERC1		CACTIN		EAPP
	ECHDC2	PRKDC		ETFB		CADPS2		ECHDC2
	EHD2	RFXANK		EYA2		CCDC3		EHD2
	EIF4EBP2	RGN		FAM110B		Ccl7		EIF4EBP2
	EIF4G1	RNF41		FAM120B		CCND1		EIF4G1
	EMC9	RRH		FAM151A		CD47		EMC9
	EPB41L2	SCP2		FAM69C		Cd52		EPB41L2
	ETFB	SCTR		FAT1		CD84		ETFB
	ETNK1	SLC11A2		FDX1		CD86		ETNK1
	EXOSC10	SLC35B2		FERMT2		CDC25B		EXOSC10
	FADS1	SLCO3A1		FRMD4B		CDK2		FADS1
	FAM114A2	SMAD4		FSD2		CDK5RAP1		FAM114A2
	FAM193A	SMTN		GABPB1		CEBPE		FAM193A
	FARSA	SMYD3		GBP2		CFB		FARSA
	FERMT2	SUCLG1		GFRA4		CHD2		FERMT2
	FKBP15	SUN2		GLT8D1		CHD7		FKBP15
	FLAD1	TBC1D9B		GNB1L		CHD9		FLAD1
	FLII	TCTE1		GPC6		CHKA		FLII
	FN3KRP	TEKT1		GPR108		CLEC7A		FN3KRP
	FNBP1	TIMM9		GRHPR		CLPP		FNBP1
	FNBP4	TPI1		GRIK1		CLUH		FNBP4
	GALM	TPK1		GRIN2A		CMA1		GALM
	GHR	UBE2G2		GSAP		CNDP1		GHR
	GLUD1	UBR2		GULP1		CNST		GLUD1
	GPD2	VIP		HCFC1R1		COG3		GPD2
	GPN1	VNN1		HIC2		COL6A2		GPN1
	GPR146	WDR1		HLA-A		CP		GPR146
	GPRC5B	XPA		HMG20B		CPE		GPRC5B
	GRAMD1B	ZNF235		HPS3		CPNE4		GRAMD1B
	GRB10			HSD11B1		CREB3L2		GRB10
	GSN			HSF2BP		CRELD1		GSN
	GSS			HSPB6		CRELD2		GSS
	GTF3A			HSPH1		Cux1		GTF3A
	GZF1			IL12RB2		CYHR1		GZF1
	HIBCH			IL1RL1		CYP39A1		HIBCH
	HIP1			IL23A		DAO		HIP1
	HLA-A			ILDR2		DCP1A		HLA-A
	HMG20A			IRX2		DDIT3		HMG20A
	HSD17B4			KAT14		DDX24		HSD17B4
	HSPA4L			KCNAB1		DDX6		HSPA4L
	IDUA			KCNJ12		DEPTOR		IDUA
	IFT81			KCNT2		DGKG		IFT81
	ILVBL			KCP		DHX37		ILVBL
	INO80			KCTD14		DLEU7		INO80
	ITGA5			KIAA1755		DNAJB6		ITGA5
	ITGB3			KIF1BP		DNAJC13		ITGB3

	JMJD8			KLHDC2		DOCK4		JMJD8
	KAT14			KMT2C		DOK3		KAT14
	KCNJ3			L3MBTL3		DPYSL2		KCNJ3
	KDSR			LAMP3		Dync1i2		KDSR
	KHDRBS2			LATS1		EDN1		KHDRBS2
	KIAA0100			LBH		EDNRB		KIAA0100
	KLC4			LLPH		EED		KLC4
	KLHL25			LRRC57		EEF1D		KLHL25
	KMT5B			LRRC61		EIF3B		KMT5B
	L3MBTL3			LYNX1		EIF4G1		L3MBTL3
	LACTB			MAN2B2		EMD		LACTB
	LAMC1			MANEA		EMILIN1		LAMC1
	LDLR			MAP2K5		ENG		LDLR
	LDLRAP1			MAP3K4		EPRS		LDLRAP1
	LEP			MAPK11		EXOC1		LEP
	LMNB2			MBD2		EXOSC7		LMNB2
	MAP2K5			MBD5		EXOSC8		MAP2K5
	MARS			MED24		F2RL1		MARS
	MED22			METTL23		FAHD2B		MED22
	MEGF9			MLC1		FAM110B		MEGF9
	MFSD4B			MPZL2		FAM114A2		MFSD4B
	MKRN2			MRPL42		FAM118B		MKRN2
	MLX			MRPS21		FAM8A1		MLX
	MRPL20			MRPS7		FBLN1		MRPL20
	MRPS16			MTRR		FCGR2A		MRPS16
	MVP			MXD1		FGFRL1		MVP
	MYLIP			MYBPH		FRMD4B		MYLIP
	MYNN			MYH6		G3BP1		MYNN
	MYSM1			MYO1C		GADD45GIP1		MYSM1
	NAA25			NCOR1		GALT		NAA25
	NAA30			NDUFAF7		Gar1		NAA30
	NAGK			NEK3		GART		NAGK
	NAPRT			NEPRO		GATA6		NAPRT
	NCF1			NFKB2		GBF1		NCF1
	NCOR1			NFX1		GCNT2		NCOR1
	NDRG2			NME6		GGCT		NDRG2
	NEDD8			NPB		GIN1		NEDD8
	OGFOD2			NPL		GKAP1		OGFOD2
	OLR1			NR5A2		GLRB		OLR1
	OSMR			NTM		GLUD1		OSMR
	OXSM			NTNG1		GNL3L		OXSM
	PDPR			NUB1		GORASP2		PDPR
	PER1			NUP54		GPAT4		PER1
	PER2			NVL		GPATCH4		PER2
	PHF20L1			OGDH		Gprasp2		PHF20L1
	PIK3C3			OLFM4		GRAMD1B		PIK3C3
	PLCB4			OLR1		GSTP1		PLCB4
	PLPP3			OSER1		H2AFZ		PLPP3
	PON2			PAICS		HCFC2		PON2
	PPA1			PARP9		HEXA		PPA1
	PPAT			PCDHA7		HIGD2A		PPAT
	PPM1H			PDE7B		HLA-A		PPM1H
	PQLC1			PHIP		HLA-DQA1		PQLC1

	PRKD3			PLAA		HNRNPC		PRKD3
	PSMD13			PLAT		HOPX		PSMD13
	PTGR2			PLEKHG1		HP1BP3		PTGR2
	PTS			PLPP3		HSP90B1		PTS
	PXMP2			POMC		HSPA2		PXMP2
	RAB3GAP2			POMT1		HSPA8		RAB3GAP2
	RAE1			PPA2		HSPB1		RAE1
	RAF1			PPIP5K1		HSPD1		RAF1
	RASSF8			PRR22		HSPH1		RASSF8
	REPS2			PRR30		ICE1		REPS2
	RGS2			PTGDR		IDH1		RGS2
	RNF114			PTGER3		IFT81		RNF114
	RNF146			PTGFRN		IGFBP4		RNF146
	RNF19A			PTGIS		IGSF3		RNF19A
	RNH1			PTPN22		ILVBL		RNH1
	RPAP1			RAB27A		IMP3		RPAP1
	S100A4			RABGGTB		ING3		S100A4
	SAFB2			RBM46		INO80		SAFB2
	SAP130			RCHY1		INSR		SAP130
	SBNO2			REPS2		IP6K1		SBNO2
	SCAF4			RET		IPMK		SCAF4
	SCARB1			RIOK1		ITGB8		SCARB1
	SCLY			RPS6KC1		IVD		SCLY
	SCRN3			RUFY2		KCNA2		SCRN3
	SFI1			SAMD12		KCNAB1		SFI1
	SLC25A44			SCARB1		KCTD1		SLC25A44
	SLC39A11			SCFD2		KDM5B		SLC39A11
	SLFN13			SCRN1		KDSR		SLFN13
	SMAD5			SELENOF		KIAA1551		SMAD5
	SMPD1			SEMA5A		KIF3B		SMPD1
	SMTN			SERTAD2		KIF3C		SMTN
	SMYD4			SESN1		KLHL25		SMYD4
	SND1			SFXN4		KLHL5		SND1
	SOD2			SH3GL2		LARS		SOD2
	SORBS1			SIDT1		LBH		SORBS1
	SPATA2			SLAIN1		LDLR		SPATA2
	Srrm1			SLC12A5		LIAS		Srrm1
	ST6GAL2			SLC24A4		LIF		ST6GAL2
	STIM2			SLC35B1		LMNB2		STIM2
	STK17B			SLC43A3		LOXL3		STK17B
	STX1A			SLC47A1		LPIN2		STX1A
	STXBP4			SLC4A1		LRP2		STXBP4
	SYNGR1			SLFN13		LY6G6D		SYNGR1
	TGS1			SMAD4		Lypd2		TGS1
	TIMM9			SORBS1		MAN2B2		TIMM9
	TKT			SOX17		MAN2C1		TKT
	TLR3			SP140		MANBA		TLR3
	TMC7			SPARCL1		MAP7		TMC7
	TMEM206			SRGAP2		MAPKAP1		TMEM206
	TNFRSF10A			STRADA		MBD5		TNFRSF10A
	TPD52L2			STRADB		MBTD1		TPD52L2
	TRAIP			SYNC		MED1		TRAIP
	TRNT1			SYNPO2		MID2		TRNT1

	TTC38			SYPL2		MKNK1		TTC38
	Ttf1			TANC2		MLEC		Ttf1
	UBQLN1			TCF25		MMP11		UBQLN1
	UBXN4			TCFL5		MOSPD1		UBXN4
	UQCC1			TECPR2		MRE11		UQCC1
	UQCC3			TERF2		MRPL28		UQCC3
	UROD			TET3		MRPS9		UROD
	UTP6			TFPI		MSMO1		UTP6
	VASP			TINAG		MTHFD2		VASP
	VEGFB			TKFC		MTX1		VEGFB
	VPS13C			TLDC1		MYBBP1A		VPS13C
	WDR41			TMEM126B		MYO1F		WDR41
	WDR53			TMEM255A		MYO9B		WDR53
	WNK1			TMEM44		MYOF		WNK1
	YKT6			TMEM59		N4BP2L1		YKT6
	ZBTB16			TNFSF4		NAA10		ZBTB16
	ZNF106			TRADD		NADK		ZNF106
	ZNF330			TRIM37		NCAPG2		ZNF330
	ZNF560			TRIM62		NCEH1		ZNF560
	ZNF775			TTC30B		NDOR1		ZNF775
	ZNRF2			TTC38		NEU1		ZNRF2
				TXN		NEURL2		ABCA4
				UAP1L1		NFE2		ABHD14A
				UBA7		NFKB2		ABL2
				USP24		NHP2		ACSS3
				USP4		NIPBL		ADORA2B
				VDR		NIPSNAP3A		AGA
				WAC		NME7		AIFM1
				WDR33		NMRAL1		ALDH18A1
				WDR83		NOC2L		ALDH5A1
				XKRX		NOL6		ATF6B
				XYLT1		NOP14		ATP2B3
				YIPF2		NPHP4		BHLHA15
				YKT6		NR1I3		BMP3
				ZBBX		NR5A2		BUB1B
				ZCCHC18		NSDHL		C1QBP
				ZFR2		NXT2		CALR
				ZGPAT		ODC1		CAPSL
				ZNF131		OGFRL1		CCDC43
				ZNF22		ORC5		CDC42EP3
				ZNF286A		OSBP		CDH15
				ZNF385B		OTC		CLSTN2
				ZNF407		OTUD6B		CMTR1
				ZNF623		P2RX4		COL5A2
						PABPC4		CTNNBL1
						PAPD7		DDX6
						PATJ		EPC1
						PCCA		ETHE1
						PCSK4		EXO1
						PCYOX1		FASLG
						PDCD2		FDFT1
						PDCD4		FER
						PDGFRB		GGCT

						PDIA6		GOT1
						PDXK		HEXIM1
						PES1		HNRNPC
						PHF10		IL27RA
						PHF12		IVD
						PHF20L1		KCNA2
						PI4KB		MAP3K4
						PIGH		MBD2
						PIGL		MTMR2
						PIK3CD		MYO9B
						PIK3R3		NFKBIB
						PKDCC		NIT1
						PKNOX1		PDE7B
						PLA2G4B		PIAS1
						PLA2G4E		PIN1
						PLAU		PLA2R1
						PLEKHM3		PLXNA3
						PLET1		PNOC
						PLGRKT		POLA2
						PLPP3		PPM1E
						PMEPA1		PRKAG1
						PNPLA7		PRKDC
						PNPO		RFXANK
						POLE4		RGN
						PPM1M		RNF41
						PREPL		RRH
						PRKCD		SCP2
						PRMT5		SCTR
						PRRC2C		SLC11A2
						PSAT1		SLC35B2
						PSMG2		SLCO3A1
						PTCD2		SMAD4
						PTPN1		SMYD3
						PTPN6		SUCLG1
						PTPRO		SUN2
						PVALB		TBC1D9B
						QPRT		TCTE1
						RANBP9		TEKT1
						RANGAP1		TPI1
						RARRES2		TPK1
						RASSF5		UBE2G2
						RAVER2		UBR2
						RB1		VIP
						RBM10		VNN1
						RBM47		WDR1
						RBM48		XPA
						RBM8A		ZNF235
						RCHY1		CSGALNACT1
						REEP3		FRYL
						REXO2		HS3ST3A1
						RIOK1		LGR5
						RND2		LPL
						RNF6		ADAMTS17

						RPIA		AGAP1
						RPL18		AGK
						RPL24		ALAD
						RPL27A		ALG12
						RPL28		ALOXE3
						RPL6		ARHGAP25
						RPL7L1		ARRDC2
						RPLP0		ATP1A4
						Rps12-ps24		ATP2A2
						RRP8		B4GALNT3
						RSF1		BAAT
						RTCB		BCOR
						RUVBL1		BOD1
						RXFP3		C18orf25
						SAMHD1		C1orf43
						SAP130		C1QTNF1
						SDCBP2		C1R
						SELPLG		C2orf70
						SENP1		CACNA1G
						SERINC3		CACTIN
						SERPINH1		CAPNS2
						SERTAD3		CATSPER2
						SETD5		CBLN3
						SH3BP2		CBX7
						SH3PXD2B		CCDC103
						SHMT1		CCDC113
						SIN3B		CCDC153
						SKP2		CCER2
						SLC11A2		CD79B
						SLC12A2		CDC25B
						SLC24A3		CDC6
						SLC31A1		CDKL2
						SLC35B1		CERS6
						SLC35B2		CHD7
						SLC38A9		CHKA
						SLC39A11		CHST9
						SLC40A1		COL9A1
						SLC7A8		CPNE7
						SLC8A1		CROT
						SLC8B1		CTDSPL2
						SMAGP		CUX1
						SMC2		CYTH1
						SNX18		DACT1
						SQLE		DKK2
						STC2		EBI3
						STIP1		EFEMP1
						STRA6		EIF4ENIF1
						STRADB		ELMO1
						STT3B		EPC2
						SUN2		EPSTI1
						SUPT6H		ERC1
						SYF2		EYA2
						SYNJ2		FAM110B

						TARS		FAM120B
						TBCD		FAM151A
						TBRG1		FAM69C
						TCF20		FAT1
						TEAD2		FDX1
						TECPR2		FRMD4B
						TERF2		FSD2
						TFPT		GABPB1
						TGFB1I1		GBP2
						TGS1		GFRA4
						THBS2		GLT8D1
						THSD4		GNB1L
						THTPA		GPC6
						TLR7		GPR108
						TMC7		GRHPR
						TMED4		GRIK1
						TMEM176A		GRIN2A
						TMEM181		GSAP
						TMEM209		GULP1
						TMEM260		HCFC1R1
						TNFAIP8L2		HIC2
						TNFRSF9		HMG20B
						TNIK		HPS3
						TNS1		HSD11B1
						TOP1MT		HSF2BP
						TOR1AIP2		HSPB6
						TPK1		HSPH1
						Tpm2		IL12RB2
						TPRG1L		IL1RL1
						TRAP1		IL23A
						TSC22D2		ILDR2
						TSEN2		IRX2
						Ttc41		KCNAB1
						TYROBP		KCNJ12
						UAP1L1		KCNT2
						UBOX5		KCP
						UBQLN1		KCTD14
						USP10		KIAA1755
						USP48		KIF1BP
						VAMP8		KLHDC2
						VARS2		KMT2C
						VAV1		LAMP3
						VAV2		LATS1
						VPS13A		LBH
						VPS13B		LLPH
						VPS33A		LRRC57
						VPS33B		LRRC61
						WDYHV1		LYNX1
						YIF1A		MAN2B2
						YIPF2		MANEA
						YIPF4		MAPK11
						YWHAH		MBD5
						ZC3H12A		MED24

						ZC3HC1		METTL23
						ZCCHC4		MLC1
						ZCCHC6		MPZL2
						ZEB2		MRPL42
						ZFAND6		MRPS21
						Zfp658		MRPS7
						ZGPAT		MTRR
						ZNF445		MXD1
						ZNF777		MYBPH
						ZRSR2		MYH6
								MYO1C
								NDUFAF7
								NEK3
								NEPRO
								NFKB2
								NFX1
								NME6
								NPB
								NPL
								NR5A2
								NTM
								NTNG1
								NUB1
								NUP54
								NVL
								OGDH
								OLFM4
								OSER1
								PAICS
								PARP9
								PCDHA7
								PHIP
								PLAA
								PLAT
								PLEKHG1
								POMC
								POMT1
								PPA2
								PPIP5K1
								PRR22
								PRR30
								PTGDR
								PTGER3
								PTGFRN
								PTGIS
								PTPN22
								RAB27A
								RABGGTB
								RBM46
								RCHY1
								RET
								RIOK1
								RPS6KC1

								RUFY2
								SAMD12
								SCFD2
								SCRN1
								SELENOF
								SEMA5A
								SERTAD2
								SESN1
								SFXN4
								SH3GL2
								SIDT1
								SLAIN1
								SLC12A5
								SLC24A4
								SLC35B1
								SLC43A3
								SLC47A1
								SLC4A1
								SOX17
								SP140
								SPARCL1
								SRGAP2
								STRADA
								STRADB
								SYNC
								SYNPO2
								SYPL2
								TANC2
								TCF25
								TCFL5
								TECPR2
								TERF2
								TET3
								TFPI
								TINAG
								TKFC
								TLDC1
								TMEM126B
								TMEM255A
								TMEM44
								TMEM59
								TNFSF4
								TRADD
								TRIM37
								TRIM62
								TTC30B
								TXN
								UAP1L1
								UBA7
								USP24
								USP4
								VDR
								WAC

								WDR33
								WDR83
								XKRX
								XYLT1
								YIPF2
								ZBBX
								ZCCHC18
								ZFR2
								ZGPAT
								ZNF131
								ZNF22
								ZNF286A
								ZNF385B
								ZNF407
								ZNF623
								2310039H08Rik
								AAGAB
								AARD
								AATF
								ABCA7
								ABCB8
								ABHD6
								ACADVL
								ACP2
								ADA
								ADGRG2
								AGTR1
								AHCYL2
								AIDA
								AIF1L
								Akr1c13
								ALOX5
								AMBRA1
								ANKRD11
								ANKS6
								AP4M1
								APBB1IP
								APEH
								AQR
								ARG1
								ARSA
								ASNS
								ASPH
								ATF4
								ATIC
								ATP2A1
								ATP2B2
								ATP6AP1
								ATXN1
								AVEN
								B3GALNT1
								B4GALT3
								BAP1

								BMS1
								BNC2
								BTAF1
								BTD
								BTK
								C11orf98
								C12orf49
								C1QB
								C20orf24
								C5orf22
								CA13
								CACNB2
								CADPS2
								CCDC3
								Ccl7
								CCND1
								CD47
								Cd52
								CD84
								CD86
								CDK2
								CDK5RAP1
								CEBPE
								CFB
								CHD2
								CHD9
								CLEC7A
								CLPP
								CLUH
								CMA1
								CNDP1
								CNST
								COG3
								COL6A2
								CP
								CPE
								CPNE4
								CREB3L2
								CRELD1
								CRELD2
								CYP39A1
								DAO
								DCP1A
								DDIT3
								DDX24
								DEPTOR
								DGKG
								DHX37
								DLEU7
								DNAJB6
								DNAJC13
								DOCK4
								DOK3

								DPYSL2
								Dync1i2
								EDN1
								EDNRB
								EED
								EEF1D
								EIF3B
								EMD
								EMILIN1
								ENG
								EPRS
								EXOC1
								EXOSC7
								EXOSC8
								F2RL1
								FAHD2B
								FAM118B
								FAM8A1
								FBLN1
								FCGR2A
								FGFRL1
								G3BP1
								GADD45GIP1
								GALT
								Gar1
								GART
								GATA6
								GBF1
								GCNT2
								GIN1
								GKAP1
								GLRB
								GNL3L
								GORASP2
								GPAT4
								GPATCH4
								Gprasp2
								GSTP1
								H2AFZ
								HCFC2
								HEXA
								HIGD2A
								HLA-DQA1
								HOPX
								HP1BP3
								HSP90B1
								HSPA2
								HSPA8
								HSPB1
								HSPD1
								ICE1
								IDH1
								IGFBP4

								IGSF3
								IMP3
								ING3
								INSR
								IP6K1
								IPMK
								ITGB8
								KCTD1
								KDM5B
								KIAA1551
								KIF3B
								KIF3C
								KLHL5
								LARS
								LIAS
								LIF
								LOXL3
								LPIN2
								LRP2
								LY6G6D
								Lypd2
								MAN2C1
								MANBA
								MAP7
								MAPKAP1
								MBTD1
								MED1
								MID2
								MKNK1
								MLEC
								MMP11
								MOSPD1
								MRE11
								MRPL28
								MRPS9
								MSMO1
								MTHFD2
								MTX1
								MYBBP1A
								MYO1F
								MYOF
								N4BP2L1
								NAA10
								NADK
								NCAPG2
								NCEH1
								NDOR1
								NEU1
								NEURL2
								NFE2
								NHP2
								NIPBL
								NIPSNAP3A

								NME7
								NMRAL1
								NOC2L
								NOL6
								NOP14
								NPHP4
								NR1I3
								NSDHL
								NXT2
								ODC1
								OGFRL1
								ORC5
								OSBP
								OTC
								OTUD6B
								P2RX4
								PABPC4
								PAPD7
								PATJ
								PCCA
								PCSK4
								PCYOX1
								PDCD2
								PDCD4
								PDGFRB
								PDIA6
								PDXK
								PES1
								PHF10
								PHF12
								PI4KB
								PIGH
								PIGL
								PIK3CD
								PIK3R3
								PKDCC
								PKNOX1
								PLA2G4B
								PLA2G4E
								PLAU
								PLEKHM3
								PLET1
								PLGRKT
								PMEPA1
								PNPLA7
								PNPO
								POLE4
								PPM1M
								PREPL
								PRKCD
								PRMT5
								PRRC2C
								PSAT1

								PSMG2
								PTCD2
								PTPN1
								PTPN6
								PTPRO
								PVALB
								QPRT
								RANBP9
								RANGAP1
								RARRES2
								RASSF5
								RAVER2
								RB1
								RBM10
								RBM47
								RBM48
								RBM8A
								REEP3
								REXO2
								RND2
								RNF6
								RPIA
								RPL18
								RPL24
								RPL27A
								RPL28
								RPL6
								RPL7L1
								RPLP0
								Rps12-ps24
								RRP8
								RSF1
								RTCB
								RUVBL1
								RXFP3
								SAMHD1
								SDCBP2
								SELPLG
								SENP1
								SERINC3
								SERPINH1
								SERTAD3
								SETD5
								SH3BP2
								SH3PXD2B
								SHMT1
								SIN3B
								SKP2
								SLC12A2
								SLC24A3
								SLC31A1
								SLC38A9
								SLC40A1

								SLC7A8
								SLC8A1
								SLC8B1
								SMAGP
								SMC2
								SNX18
								SQLE
								STC2
								STIP1
								STRA6
								STT3B
								SUPT6H
								SYF2
								SYNJ2
								TARS
								TBCD
								TBRG1
								TCF20
								TEAD2
								TFPT
								TGFB1I1
								THBS2
								THSD4
								THTPA
								TLR7
								TMED4
								TMEM176A
								TMEM181
								TMEM209
								TMEM260
								TNFAIP8L2
								TNFRSF9
								TNIK
								TNS1
								TOP1MT
								TOR1AIP2
								Tpm2
								TPRG1L
								TRAP1
								TSC22D2
								TSEN2
								Ttc41
								TYROBP
								UBOX5
								USP10
								USP48
								VAMP8
								VARS2
								VAV1
								VAV2
								VPS13A
								VPS13B
								VPS33A

								VPS33B
								WDYHV1
								YIF1A
								YIPF4
								YWHAH
								ZC3H12A
								ZC3HC1
								ZCCHC4
								ZCCHC6
								ZEB2
								ZFAND6
								Zfp658
								ZNF445
								ZNF777
								ZRSR2
								NARFL
								TMEM185A
								ZNF212
**Overlap with**	**GSE44387**	**GSE63852**	**GSE58642**	**GSE50527**	**GSE58516**	**GSE86923**		**Combined lists**
DEGs in male	AGPAT3	AAAS	CSGALNACT1	ABCA3	PEX26	AAAS		AGPAT3
	ALDOA	ALDH5A1	FRYL	AGAP3		ABCA7		ALDOA
	ANKRD17	ARL6IP1	LPL	BCOR		ACP2		ANKRD17
	Anp32a	CAD		BOD1		ACTN4		Anp32a
	APMAP	CALR		C1orf43		AIDA		APMAP
	APOE	CANX		C1QTNF1		Akap9		APOE
	BCL9L	CLSTN2		CACNA1G		ANKRD11		BCL9L
	BIRC6	CNEP1R1		CACTIN		AQR		BIRC6
	CDHR4	COL5A2		CAMKV		ARL6IP1		CDHR4
	CDK5RAP2	EPC1		CDC25B		ASH1L		CDK5RAP2
	CIC	ERBB4		CLINT1		ATG2B		CIC
	CMIP	FER		COBLL1		AVL9		CMIP
	COG2	FGFR2		CROT		BMS1		COG2
	CSDE1	FTSJ1		CTDSPL2		BTAF1		CSDE1
	CYP1B1	GPD1		CUX1		CACNA1G		CYP1B1
	DENND2A	HNRNPC		CYTH1		CACTIN		DENND2A
	DHX36	INHBB		DACT1		CADPS2		DHX36
	DNASE2	MAP1B		DIRAS2		CASKIN1		DNASE2
	EIF4G1	MLLT11		ECH1		CCND1		EIF4G1
	EPB41L2	MTMR2		ELAVL2		CDC25B		EPB41L2
	EXOSC10	NEURL1B		EPC2		CDH3		EXOSC10
	EZH1	NF1		ERC1		CEP295		EZH1
	FAM114A2	POU3F3		ERICH2		CHD2		FAM114A2
	FASTK	SCP2		FAM110B		CHD6		FASTK
	FKBP15	SLC11A2		FASTK		CHD9		FKBP15
	GCN1	SUN2		FAT1		CKB		GCN1
	GHR	TIMM13		FRMD4B		CLDN12		GHR
	GLUD1	TTC3		FUT9		CLDN2		GLUD1
	GON4L	WDR1		GPC6		CNTN2		GON4L
	GSN	ZBTB8OS		HLA-A		CPE		GSN
	HIP1	ZNF235		HPS3		CPEB3		HIP1
	HLA-A			ILDR2		CPNE4		HLA-A
	HSD17B4			KCTD20		CYCS		HSD17B4
	IFT81			KIAA1755		DEK		IFT81

	KBTBD11			KITLG		DGKZ		KBTBD11
	KCNB1			KLHL29		DHX15		KCNB1
	KDM5C			KMT2C		DLEU7		KDM5C
	KIAA0100			KRBA1		DOCK4		KIAA0100
	KLC4			L3MBTL3		DPYSL2		KLC4
	KMT5B			LATS1		Dst		KMT5B
	L3MBTL3			LBH		Dync1i2		L3MBTL3
	LAMB1			LLPH		EDRF1		LAMB1
	MECP2			MAP6		EIF4G1		MECP2
	MFN1			MBOAT2		EXOSC7		MFN1
	MLX			MED24		FAM110B		MLX
	MPDU1			MFRP		FAM114A2		MPDU1
	MYNN			MLC1		FBLN1		MYNN
	MYSM1			MYH6		FCGR2A		MYSM1
	NAA25			NACC1		FRMD4B		NAA25
	NCF1			NASP		FRY		NCF1
	OGFOD2			NF1		FSCN1		OGFOD2
	PBRM1			NFX1		G3BP1		PBRM1
	PER2			NPTXR		GADD45A		PER2
	PHF20L1			NTN1		GAPVD1		PHF20L1
	PLXNC1			NTNG1		GATAD2B		PLXNC1
	PPA1			NUB1		GLUD1		PPA1
	PPM1H			NXPH4		Gprasp2		PPM1H
	PPP4R3B			OGDH		GPSM1		PPP4R3B
	RAF1			OTP		GSTP1		RAF1
	RALGAPA2			PHF13		GTF2H4		RALGAPA2
	RAPGEF1			PIK3R2		H2AFZ		RAPGEF1
	RBM5			PLAT		HELZ		RBM5
	REPS2			REPS2		HLA-A		REPS2
	RNF10			RIOK1		HMCN1		RNF10
	RNF114			RNF182		HNRNPC		RNF114
	RNF19A			SAMD12		HSP90B1		RNF19A
	RNF31			SEMA5A		HSPA8		RNF31
	SAP130			SESN1		HSPB1		SAP130
	SCAF4			SHANK3		HSPD1		SCAF4
	SHANK3			SLC12A5		IARS		SHANK3
	SLC25A4			SLFN13		ICE1		SLC25A4
	SLC45A4			SORBS1		IFT81		SLC45A4
	SLC9A6			STOX2		IGSF3		SLC9A6
	SLFN13			SYNC		INTS5		SLFN13
	SND1			TANC2		IP6K1		SND1
	SORBS1			TAPBP		ITGB8		SORBS1
	SPATA2			TCF25		ITM2B		SPATA2
	TAOK1			TECPR2		KCTD1		TAOK1
	TGS1			TET3		KDM5A		TGS1
	TMEM206			TMEM63B		KDM5B		TMEM206
	TMEM263			TMEM72		KRBA1		TMEM263
	TNRC6B			TRIM37		LARS		TNRC6B
	TRIP12			TRIM62		LBH		TRIP12
	TRNT1			TRPV4		LPIN2		TRNT1
	Ttf1			UNC13D		LRP1		Ttf1
	UBR4			USP24		MAP1B		UBR4
	VEGFB			USPL1		MBTD1		VEGFB

	VPS13C			WNK2		MED1		VPS13C
	YME1L1			XYLT1		MIER1		YME1L1
	ZBTB20			YIPF2		MOSPD1		ZBTB20
	ZMYM5			ZBTB8OS		MRPL4		ZMYM5
	ZNF12			ZNF131		MSI2		ZNF12
	ZNF274			ZNF274		N4BP2L1		ZNF274
	ZNF330			ZNF407		NCL		ZNF330
	ZNF560			ZNF623		NFIB		ZNF560
						NHLRC3		CSGALNACT1
						NHSL1		FRYL
						NIPBL		LPL
						NNAT		AAAS
						NRXN2		ALDH5A1
						NUDT21		ARL6IP1
						OTUD7B		CAD
						PEAR1		CALR
						PFN1		CANX
						PHF13		CLSTN2
						PHF20L1		CNEP1R1
						PHGDH		COL5A2
						PKP4		EPC1
						PLD3		ERBB4
						PLEKHM3		FER
						PLIN2		FGFR2
						PMEPA1		FTSJ1
						PPP4R3B		GPD1
						PRRC2C		HNRNPC
						Prrt1		INHBB
						PTPRO		MAP1B
						RBM10		MLLT11
						REXO2		MTMR2
						RIOK1		NEURL1B
						RNASET2		NF1
						RNF139		POU3F3
						RPL18		SCP2
						RPL6		SLC11A2
						RPL7L1		SUN2
						RPLP0		TIMM13
						RSF1		TTC3
						RSRC1		WDR1
						Rsrc2		ZBTB8OS
						RTCB		ZNF235
						SAP130		ABCA3
						SEH1L		AGAP3
						SELENOP		BCOR
						SERINC1		BOD1
						SERINC3		C1orf43
						SETD5		C1QTNF1
						SH3PXD2B		CACNA1G
						SIN3B		CACTIN
						SLC11A2		CAMKV
						SLC24A3		CDC25B
						SMC3		CLINT1

						SMCHD1		COBLL1
						SNAP25		CROT
						SOD1		CTDSPL2
						SOX5		CUX1
						SRM		CYTH1
						SRSF11		DACT1
						STRA6		DIRAS2
						STX3		ECH1
						SUN2		ELAVL2
						SYF2		EPC2
						TAOK1		ERC1
						TAPBP		ERICH2
						TBC1D2B		FAM110B
						TBL1X		FAT1
						TCF20		FRMD4B
						TECPR2		FUT9
						TGS1		GPC6
						THBS2		HPS3
						TM6SF1		ILDR2
						TMEM181		KCTD20
						TNC		KIAA1755
						TNIK		KITLG
						TOP1MT		KLHL29
						TP53INP2		KMT2C
						TPR		KRBA1
						TPRG1L		LATS1
						TRIP12		LBH
						TSC22D2		LLPH
						UHRF2		MAP6
						VARS2		MBOAT2
						VMP1		MED24
						VPS54		MFRP
						WASHC4		MLC1
						WNK2		MYH6
						WSB1		NACC1
						YIF1B		NASP
						YIPF2		NFX1
						YIPF4		NPTXR
						YTHDF3		NTN1
						YWHAH		NTNG1
						ZCCHC6		NUB1
						ZFAND6		NXPH4
						Zfp60		OGDH
						Zfp658		OTP
						ZMAT1		PHF13
						ZNF436		PIK3R2
						ZNF445		PLAT
						ZNF777		RIOK1
								RNF182
								SAMD12
								SEMA5A
								SESN1
								SLC12A5

								STOX2
								SYNC
								TANC2
								TAPBP
								TCF25
								TECPR2
								TET3
								TMEM63B
								TMEM72
								TRIM37
								TRIM62
								TRPV4
								UNC13D
								USP24
								USPL1
								WNK2
								XYLT1
								YIPF2
								ZNF131
								ZNF407
								ZNF623
								PEX26
								ABCA7
								ACP2
								ACTN4
								AIDA
								Akap9
								ANKRD11
								AQR
								ASH1L
								ATG2B
								AVL9
								BMS1
								BTAF1
								CADPS2
								CASKIN1
								CCND1
								CDH3
								CEP295
								CHD2
								CHD6
								CHD9
								CKB
								CLDN12
								CLDN2
								CNTN2
								CPE
								CPEB3
								CPNE4
								CYCS
								DEK
								DGKZ
								DHX15

								DLEU7
								DOCK4
								DPYSL2
								Dst
								Dync1i2
								EDRF1
								EXOSC7
								FBLN1
								FCGR2A
								FRY
								FSCN1
								G3BP1
								GADD45A
								GAPVD1
								GATAD2B
								Gprasp2
								GPSM1
								GSTP1
								GTF2H4
								H2AFZ
								HELZ
								HMCN1
								HSP90B1
								HSPA8
								HSPB1
								HSPD1
								IARS
								ICE1
								IGSF3
								INTS5
								IP6K1
								ITGB8
								ITM2B
								KCTD1
								KDM5A
								KDM5B
								LARS
								LPIN2
								LRP1
								MBTD1
								MED1
								MIER1
								MOSPD1
								MRPL4
								MSI2
								N4BP2L1
								NCL
								NFIB
								NHLRC3
								NHSL1
								NIPBL
								NNAT
								NRXN2

								NUDT21
								OTUD7B
								PEAR1
								PFN1
								PHGDH
								PKP4
								PLD3
								PLEKHM3
								PLIN2
								PMEPA1
								PRRC2C
								Prrt1
								PTPRO
								RBM10
								REXO2
								RNASET2
								RNF139
								RPL18
								RPL6
								RPL7L1
								RPLP0
								RSF1
								RSRC1
								Rsrc2
								RTCB
								SEH1L
								SELENOP
								SERINC1
								SERINC3
								SETD5
								SH3PXD2B
								SIN3B
								SLC24A3
								SMC3
								SMCHD1
								SNAP25
								SOD1
								SOX5
								SRM
								SRSF11
								STRA6
								STX3
								SYF2
								TBC1D2B
								TBL1X
								TCF20
								THBS2
								TM6SF1
								TMEM181
								TNC
								TNIK
								TOP1MT
								TP53INP2

								TPR
								TPRG1L
								TSC22D2
								UHRF2
								VARS2
								VMP1
								VPS54
								WASHC4
								WSB1
								YIF1B
								YIPF4
								YTHDF3
								YWHAH
								ZCCHC6
								ZFAND6
								Zfp60
								Zfp658
								ZMAT1
								ZNF436
								ZNF445
								ZNF777
**Overlap with**	**GSE44387**	**GSE63852**	**GSE58642**	**GSE50527**	**GSE58516**	**GSE86923**		**Combined lists**
DEGs in female	AACS	ABHD14A	FRYL	ABCA3	PEX26	ACP2		AACS
	ABCC5	ACSS3		ABT1	SIDT1	ACTN4		ABCC5
	ACLY	ADCY5		ADD3	SLC25A39	AHI1		ACLY
	ADTRP	ADD3		AGAP3		ALCAM		ADTRP
	AGL	ADORA2B		AGL		ANKS6		AGL
	ALDOA	AIFM1		ALDH1A2		APOPT1		ALDOA
	APOE	ALCAM		ATL1		ARPC3		APOE
	APOPT1	ALDH18A1		C11orf95		ASCC3		APOPT1
	ARAP2	ARHGAP5		C1orf43		ASNS		ARAP2
	ARHGEF25	ASCC3		CACNA1G		ATF4		ARHGEF25
	ARIH2	ATF6B		CACNG2		ATIC		ARIH2
	ARNT	ATP2B3		CAMK1G		ATP13A1		ARNT
	ARPC3	ATRN		CASK		ATP2B2		ARPC3
	ASTN1	CAD		CBX7		B4GALT3		ASTN1
	ATG101	CALR		CCDC153		BACE1		ATG101
	BCL6	CANX		CDK5R1		BAP1		BCL6
	BCL9L	CAPSL		CDKL2		BCCIP		BCL9L
	BIRC6	CLTA		CDV3		BCL6		BIRC6
	CA5B	COL5A2		CERS2		BNC2		CA5B
	CASKIN2	EIF2A		CFH		BRSK1		CASKIN2
	CCNJ	EIF5		CHCHD10		C2CD3		CCNJ
	CD93	ELAVL3		CHKA		CACNA1G		CD93
	CFP	EPC1		CHORDC1		CACNB2		CFP
	CHPF	ERBB4		CNTN4		CADPS2		CHPF
	CHPT1	FDFT1		COL5A1		CALCOCO1		CHPT1
	CIC	FER		CPNE7		Cald1		CIC
	CLEC16A	FGFR2		CROT		CASK		CLEC16A
	COPE	HIPK2		CSE1L		CASKIN1		COPE
	CRIM1	HNRNPC		CTDSPL2		CCDC3		CRIM1
	CSDE1	KCNA2		CUX1		CCND1		CSDE1
	CYHR1	KCNQ4		DACT1		CD47		CYHR1

	CYP1B1	LUM		DCUN1D5		CDH3		CYP1B1
	DCAF5	MAP1B		DDN		CDK2		DCAF5
	DDX46	MLLT11		DISP1		CELF2		DDX46
	DECR1	MRPL3		DKK2		CFB		DECR1
	DISP1	MVK		EFEMP1		CFP		DISP1
	EEF2K	MYO9B		EHD2		CHD2		EEF2K
	EHD2	OASL		EIF4E		CHD6		EHD2
	EIF4EBP2	OTX1		EIF4ENIF1		CHD9		EIF4EBP2
	EIF4G1	PAN2		EYA2		CHI3L1		EIF4G1
	EPB41L2	PEPD		FAM110B		CHKA		EPB41L2
	ETNK1	PHYH		FAM69B		CLUH		ETNK1
	EXOSC10	PLEC		FAT1		CMA1		EXOSC10
	EZH1	PLXNA3		FERMT2		CNST		EZH1
	FAM114A2	POLA2		GABRA2		CNTN4		FAM114A2
	FERMT2	POU3F3		GLT8D1		COL6A2		FERMT2
	FKBP11	PPM1E		GNG10		COPE		FKBP11
	FKBP15	PRKAG1		GPR108		CP		FKBP15
	FLAD1	PRKDC		GPR173		CPA3		FLAD1
	FLII	RNF41		GRIN2A		CPE		FLII
	FN3KRP	RPL13		HNRNPA0		CPEB3		FN3KRP
	GATB	SLC12A4		HSPH1		CPNE4		GATB
	GHITM	SLCO3A1		IGFBP2		CREB3L2		GHITM
	GLUD1	SLIT1		ILDR2		CRELD2		GLUD1
	GPRC5B	SMTN		ITGA11		CSE1L		GPRC5B
	GRB10	SPP1		ITGB1		CTSV		GRB10
	GSN	STX6		KAT14		CUL3		GSN
	HIP1	SYN1		KCNQ4		Cux1		HIP1
	HSD17B4	TBC1D9B		KCNT2		CYCS		HSD17B4
	IDUA	TCTE1		KCTD14		CYHR1		IDUA
	ITGA5	TPK1		KDM4B		DCAKD		ITGA5
	ITGB3	TTC3		KIAA1755		DDX24		ITGB3
	JMJD8	U2SURP		KITLG		DGKG		JMJD8
	KAT14	UBR2		KMT2C		DGKZ		KAT14
	KDM4B	USP9X		KRBA1		DHX37		KDM4B
	KDM5C	WDR1		KRCC1		DNAJC13		KDM5C
	KHDRBS2	ZNF235		L3MBTL3		DOCK4		KHDRBS2
	KIAA0100			LCAT		DOT1L		KIAA0100
	KIF13B			LGALS8		DPYSL2		KIF13B
	KIF1B			LHFPL4		DRAM2		KIF1B
	KLHL25			LINC00998		Dst		KLHL25
	KMT5B			LRRC57		Dync1i2		KMT5B
	L3MBTL3			LYNX1		EGFR		L3MBTL3
	LAMB1			MAGI1		EIF3B		LAMB1
	LAMC1			MAN2B2		EIF4A1		LAMC1
	LDLR			MAPK11		EIF4G1		LDLR
	LMNB2			MDK		EIF5		LMNB2
	MAP3K10			MED24		EIF5A		MAP3K10
	MARS			MFAP3L		ELAC2		MARS
	MFN1			MFSD12		EMILIN1		MFN1
	MFSD12			MLC1		ENG		MFSD12
	MYSM1			MPZL2		EPRS		MYSM1
	NAA30			MYBPH		FAM110B		NAA30
	NAGK			MYO1C		FAM114A2		NAGK

	NDRG2			NCOA1		FAM171A2		NDRG2
	NDUFA4			NDUFAF7		FAM8A1		NDUFA4
	NDUFB9			NME1		FAR1		NDUFB9
	NKTR			NTM		FBLN1		NKTR
	OSTC			NTN1		Fcna		OSTC
	PALD1			NTNG1		FKBP11		PALD1
	PBRM1			OGDH		Fnbp1l		PBRM1
	PECR			PAICS		FSCN1		PECR
	PER1			PALD1		GARS		PER1
	PER2			PAN2		GBF1		PER2
	PHF20L1			PAQR3		GLRB		PHF20L1
	PLCB4			PCDHA7		GLUD1		PLCB4
	PLPP3			PHIP		GOLGB1		PLPP3
	PQLC1			PI4KA		Gprasp2		PQLC1
	PSMA7			PIK3R2		GPSM1		PSMA7
	PSMB4			PLAA		H2AFZ		PSMB4
	PSMD13			PLEC		HES6		PSMD13
	QKI			PLPP3		HIGD2A		QKI
	RAB3GAP2			POMT1		HM13		RAB3GAP2
	RALGAPA2			PPIP5K1		HNRNPC		RALGAPA2
	RAPGEF1			PSMA7		HNRNPL		RAPGEF1
	RBM5			PTGFRN		HS6ST2		RBM5
	RCOR1			REPS2		HSP90AA1		RCOR1
	RDH10			RET		HSP90B1		RDH10
	REPS2			RNF138		HSPA8		REPS2
	RNASEL			S1PR1		HSPH1		RNASEL
	SAFB2			SAMD12		IAH1		SAFB2
	SCAF4			SBK1		ICE1		SCAF4
	SCRN3			SEMA5A		IGFBP2		SCRN3
	SCYL1			SERINC5		IGFBP4		SCYL1
	SDC3			SERPINB6		IMP3		SDC3
	SHANK3			SHANK3		IMP4		SHANK3
	SLC37A3			SIAH1		IMPAD1		SLC37A3
	SLC45A4			SIDT1		ING3		SLC45A4
	SMTN			SLAIN1		IP6K1		SMTN
	SND1			SLC12A5		IPO4		SND1
	SNX27			SLC24A4		ITGB8		SNX27
	SNX33			SLC47A1		ITGBL1		SNX33
	SORBS1			SLC4A1		KCNA2		SORBS1
	STK39			SLC9A9		KIAA0922		STK39
	STXBP4			SNRNP48		KIAA1551		STXBP4
	Sult1a1			SNTB2		KIF1B		Sult1a1
	SYNGR1			SORBS1		KIF3B		SYNGR1
	SZRD1			SPARCL1		KIF3C		SZRD1
	TAOK1			SSPN		KLHL25		TAOK1
	TENM4			SSR1		KLHL5		TENM4
	TIMP2			STAT6		KRBA1		TIMP2
	TMC7			STN1		LAMP2		TMC7
	TMEM57			STRADB		LARS		TMEM57
	TRNT1			STX7		LDLR		TRNT1
	TTC14			SYT4		LGALS8		TTC14
	Ttf1			TACR1		LGALSL		Ttf1
	UBR3			TANC2		LMNB2		UBR3

	UBR4			TAPBP		LOX		UBR4
	UBXN4			TCN2		LRP1		UBXN4
	UTP6			TECPR2		LRP2		UTP6
	VEGFB			TERF2		LUM		VEGFB
	VPS13C			TET3		MAN2B1		VPS13C
	WDR53			TFRC		MAN2B2		WDR53
	WNK1			TGFB2		MAN2C1		WNK1
	XPR1			TMEM63B		MAP1B		XPR1
	ZBTB16			TRIM37		MAP1S		ZBTB16
	ZBTB20			TRIM62		MAP7		ZBTB20
	ZMYM5			USP24		MAPRE1		ZMYM5
	ZNF106			VCL		MBTD1		ZNF106
	ZNF12			WAC		MDK		ZNF12
	ZNF274			ZCCHC18		MED1		ZNF274
	ZNRF2			ZFR2		MFAP3L		ZNRF2
				ZGPAT		MFSD11		ABHD14A
				ZNF274		MICU2		ACSS3
				ZNF623		MKI67		ADCY5
				ZNF706		MKNK1		ADD3
						MLEC		ADORA2B
						MMP11		AIFM1
						MRC1		ALCAM
						MRPL3		ALDH18A1
						MRPL52		ARHGAP5
						MSMO1		ASCC3
						MYADM		ATF6B
						MYH9		ATP2B3
						MYO9B		ATRN
						MYOF		CAD
						NARF		CALR
						NCDN		CANX
						NCEH1		CAPSL
						NCL		CLTA
						NECTIN2		COL5A2
						NEK1		EIF2A
						NEUROD6		EIF5
						NFIB		ELAVL3
						NFIX		EPC1
						NHLRC3		ERBB4
						NHSL1		FDFT1
						NIPBL		FER
						NIPSNAP3A		FGFR2
						NOL6		HIPK2
						NPHP4		HNRNPC
						NPR1		KCNA2
						NR1I3		KCNQ4
						NSDHL		LUM
						NUDT4		MAP1B
						NUPR1		MLLT11
						ODC1		MRPL3
						OGFRL1		MVK
						PAPD7		MYO9B
						PCDH17		OASL

						PCSK4		OTX1
						PCYOX1		PAN2
						PDAP1		PEPD
						PDCD4		PHYH
						PDIA6		PLEC
						PEAR1		PLXNA3
						PECAM1		POLA2
						PHF10		POU3F3
						PHF12		PPM1E
						PHF20L1		PRKAG1
						PHKB		PRKDC
						PIGL		RNF41
						PIK3CD		RPL13
						PIM3		SLC12A4
						PKDCC		SLCO3A1
						PKP4		SLIT1
						PLA2G4B		SPP1
						PLA2G4E		STX6
						PLD3		SYN1
						PLEC		TBC1D9B
						PLEKHA5		TCTE1
						PLPP3		TPK1
						PLXNA2		TTC3
						PNISR		U2SURP
						PRMT5		UBR2
						PRRG1		USP9X
						PSAP		WDR1
						PTGES3		ZNF235
						PTP4A1		FRYL
						RAB23		PEX26
						RB1		SIDT1
						RBM10		SLC25A39
						RCOR1		ABCA3
						REXO2		ABT1
						RFC4		AGAP3
						RNMT		ALDH1A2
						RPL18		ATL1
						RPL24		C11orf95
						RPL6		C1orf43
						RPL7L1		CACNA1G
						RPS16		CACNG2
						RPS6KB1		CAMK1G
						RSF1		CASK
						RTCB		CBX7
						RTN3		CCDC153
						SARAF		CDK5R1
						SBF1		CDKL2
						SDF2L1		CDV3
						SEH1L		CERS2
						SELENOP		CFH
						SELENOW		CHCHD10
						SERINC1		CHKA
						SERINC3		CHORDC1

						SERPINB6		CNTN4
						SERPINH1		COL5A1
						SETD5		CPNE7
						SH3BP2		CROT
						SH3PXD2B		CSE1L
						SLA		CTDSPL2
						SLC12A2		CUX1
						SLC1A5		DACT1
						SLC24A3		DCUN1D5
						SLC25A27		DDN
						SLC35B3		DKK2
						SLC6A13		EFEMP1
						SLC8A1		EIF4E
						SMARCA4		EIF4ENIF1
						SMC1A		EYA2
						SMC2		FAM110B
						SMC3		FAM69B
						SMCHD1		FAT1
						SNAP25		GABRA2
						SNRNP48		GLT8D1
						SNRPB		GNG10
						SOX12		GPR108
						SPP1		GPR173
						SQLE		GRIN2A
						SREBF2		HNRNPA0
						SRSF2		HSPH1
						SSPN		IGFBP2
						STRADB		ILDR2
						STT3B		ITGA11
						STX7		ITGB1
						SUPT6H		KCNT2
						SYNJ2		KCTD14
						SZRD1		KIAA1755
						TAOK1		KITLG
						TAPBP		KMT2C
						TBC1D10B		KRBA1
						TCN2		KRCC1
						TEAD2		LCAT
						TECPR2		LGALS8
						TERF2		LHFPL4
						TES		LINC00998
						TGFB1I1		LRRC57
						TGFB2		LYNX1
						THTPA		MAGI1
						TIMP2		MAN2B2
						TMC7		MAPK11
						TMEM127		MDK
						Tmem223		MED24
						TNIK		MFAP3L
						TNS1		MLC1
						TOP1MT		MPZL2
						TPK1		MYBPH
						Tpm2		MYO1C

						TPRG1L		NCOA1
						TRAF4		NDUFAF7
						TRIM36		NME1
						TRIO		NTM
						TSEN2		NTN1
						TTYH3		NTNG1
						U2SURP		OGDH
						UBTF		PAICS
						UNC5A		PAQR3
						USP10		PCDHA7
						VARS2		PHIP
						VAT1		PI4KA
						VPS13A		PIK3R2
						VPS13B		PLAA
						WBP1		POMT1
						WDR20		PPIP5K1
						WLS		PTGFRN
						WSB1		RET
						YIF1B		RNF138
						YWHAH		S1PR1
						ZC3HC1		SAMD12
						ZCCHC6		SBK1
						ZEB2		SEMA5A
						ZFAND6		SERINC5
						Zfp60		SERPINB6
						Zfp658		SIAH1
						ZGPAT		SLAIN1
						ZMAT1		SLC12A5
						ZNF777		SLC24A4
								SLC47A1
								SLC4A1
								SLC9A9
								SNRNP48
								SNTB2
								SPARCL1
								SSPN
								SSR1
								STAT6
								STN1
								STRADB
								STX7
								SYT4
								TACR1
								TANC2
								TAPBP
								TCN2
								TECPR2
								TERF2
								TET3
								TFRC
								TGFB2
								TMEM63B
								TRIM37

								TRIM62
								USP24
								VCL
								WAC
								ZCCHC18
								ZFR2
								ZGPAT
								ZNF623
								ZNF706
								ACP2
								ACTN4
								AHI1
								ANKS6
								ASNS
								ATF4
								ATIC
								ATP13A1
								ATP2B2
								B4GALT3
								BACE1
								BAP1
								BCCIP
								BNC2
								BRSK1
								C2CD3
								CACNB2
								CADPS2
								CALCOCO1
								Cald1
								CASKIN1
								CCDC3
								CCND1
								CD47
								CDH3
								CDK2
								CELF2
								CFB
								CHD2
								CHD6
								CHD9
								CHI3L1
								CLUH
								CMA1
								CNST
								COL6A2
								CP
								CPA3
								CPE
								CPEB3
								CPNE4
								CREB3L2
								CRELD2
								CTSV

								CUL3
								CYCS
								DCAKD
								DDX24
								DGKG
								DGKZ
								DHX37
								DNAJC13
								DOCK4
								DOT1L
								DPYSL2
								DRAM2
								Dst
								Dync1i2
								EGFR
								EIF3B
								EIF4A1
								EIF5A
								ELAC2
								EMILIN1
								ENG
								EPRS
								FAM171A2
								FAM8A1
								FAR1
								FBLN1
								Fcna
								Fnbp1l
								FSCN1
								GARS
								GBF1
								GLRB
								GOLGB1
								Gprasp2
								GPSM1
								H2AFZ
								HES6
								HIGD2A
								HM13
								HNRNPL
								HS6ST2
								HSP90AA1
								HSP90B1
								HSPA8
								IAH1
								ICE1
								IGFBP4
								IMP3
								IMP4
								IMPAD1
								ING3
								IP6K1
								IPO4

								ITGB8
								ITGBL1
								KIAA0922
								KIAA1551
								KIF3B
								KIF3C
								KLHL5
								LAMP2
								LARS
								LGALSL
								LOX
								LRP1
								LRP2
								MAN2B1
								MAN2C1
								MAP1S
								MAP7
								MAPRE1
								MBTD1
								MED1
								MFSD11
								MICU2
								MKI67
								MKNK1
								MLEC
								MMP11
								MRC1
								MRPL52
								MSMO1
								MYADM
								MYH9
								MYOF
								NARF
								NCDN
								NCEH1
								NCL
								NECTIN2
								NEK1
								NEUROD6
								NFIB
								NFIX
								NHLRC3
								NHSL1
								NIPBL
								NIPSNAP3A
								NOL6
								NPHP4
								NPR1
								NR1I3
								NSDHL
								NUDT4
								NUPR1
								ODC1

								OGFRL1
								PAPD7
								PCDH17
								PCSK4
								PCYOX1
								PDAP1
								PDCD4
								PDIA6
								PEAR1
								PECAM1
								PHF10
								PHF12
								PHKB
								PIGL
								PIK3CD
								PIM3
								PKDCC
								PKP4
								PLA2G4B
								PLA2G4E
								PLD3
								PLEKHA5
								PLXNA2
								PNISR
								PRMT5
								PRRG1
								PSAP
								PTGES3
								PTP4A1
								RAB23
								RB1
								RBM10
								REXO2
								RFC4
								RNMT
								RPL18
								RPL24
								RPL6
								RPL7L1
								RPS16
								RPS6KB1
								RSF1
								RTCB
								RTN3
								SARAF
								SBF1
								SDF2L1
								SEH1L
								SELENOP
								SELENOW
								SERINC1
								SERINC3
								SERPINH1

								SETD5
								SH3BP2
								SH3PXD2B
								SLA
								SLC12A2
								SLC1A5
								SLC24A3
								SLC25A27
								SLC35B3
								SLC6A13
								SLC8A1
								SMARCA4
								SMC1A
								SMC2
								SMC3
								SMCHD1
								SNAP25
								SNRPB
								SOX12
								SQLE
								SREBF2
								SRSF2
								STT3B
								SUPT6H
								SYNJ2
								TBC1D10B
								TEAD2
								TES
								TGFB1I1
								THTPA
								TMEM127
								Tmem223
								TNIK
								TNS1
								TOP1MT
								Tpm2
								TPRG1L
								TRAF4
								TRIM36
								TRIO
								TSEN2
								TTYH3
								UBTF
								UNC5A
								USP10
								VARS2
								VAT1
								VPS13A
								VPS13B
								WBP1
								WDR20
								WLS
								WSB1

								YIF1B
								YWHAH
								ZC3HC1
								ZCCHC6
								ZEB2
								ZFAND6
								Zfp60
								Zfp658
								ZMAT1
								ZNF777

Supplementary Table S9. The list of primers for qRT-PCR analyses.

Gene	Forward primer (5'-> 3')	Reverse primer (5'-> 3')
Auts2	GTCCTCCAGGCCCTAGTCTC	CACACTGGGGCTATCCTTGT
Foxp2	CCACAAGTTTGGGCTATGGA	ACTGCTGTTCCCATTGCTGT
Smarcc2	GGCTGAAGGAAGTTGCAGAG	ATCTGGGTCTCCACCAACAG
Dicer1	ACGAGATGCAAGGAATGGAC	GTACACCTGCCAGACCACCT
Rn18s	CTGGATACCGCAGCTAGGAA	GAATTTCACCTCTAGCGGCG
